# Multiscale Plant Defense Strategies against Ciprofloxacin Stress: From Chloroplast-Centered Adaptation to Microbiome Coordination

**DOI:** 10.34133/research.1082

**Published:** 2026-01-15

**Authors:** Chen Ling, Xiaohan Chen, Jing Yang, Xinhua Zhan, Jason C. White, Melanie Kah, Yu Shen, Baoshan Xing

**Affiliations:** ^1^ Co-Innovation Center for Sustainable Forestry in Southern China, College of Ecology and Environment, National Positioning Observation Station of Hung-tse Lake Wetland Ecosystem in Jiangsu Province, Nanjing Forestry University, Nanjing, Jiangsu 210037, China.; ^2^ Stockbridge School of Agriculture, University of Massachusetts, Amherst, MA 01003, USA.; ^3^ Electron Microscope Lab, Advance Analysis and Test Center, Nanjing Forestry University, Nanjing 210037, China.; ^4^ College of Resources and Environmental Sciences, Nanjing Agricultural University, Nanjing 210095, China.; ^5^ The Connecticut Agricultural Experiment Station, New Haven, CT 06504, USA.; ^6^ School of Environment, University of Auckland, Auckland 1010, New Zealand.

## Abstract

Biological stress responses operate across multiple scales, yet the coordination mechanisms remain poorly characterized. Here, we present comprehensive systems-level characterization of coordinated plant and endophytic microbiome responses during xenobiotic stress, integrating ultrastructural analysis, proteomics, and microbiome profiling in rice seedlings exposed to ciprofloxacin. We discovered a sophisticated multi-level defense system with chloroplasts as a key hub within a highly integrated cross-organellar network, comprising 36% of all differentially expressed proteins. The system operates through 3 integrated mechanisms: (a) differential cellular accumulation patterns showing 14-fold tissue-specific differences, (b) reactive-oxygen-species-associated metabolic processes with reduced toxicity of transformation products, and (c) restructuring of endophytic bacterial communities toward stress-resistant genera. This work indicates that biological systems deploy hierarchical, integrated responses spanning from organellar to ecosystem levels. The chloroplast-centered response represents a comprehensive characterization of multicompartmental responses with implications across multiple biological fields. Our findings illustrate how multidisciplinary systems approaches can uncover emergent properties of multiscale biological responses invisible to single-scale analyses, providing a framework for investigating multiscale responses across diverse biological systems.

## Introduction

Veterinary antibiotics, particularly fluoroquinolones (FQs), enter environmental matrices through wastewater irrigation and animal manure [1–3]. Their strong adsorption to soil particles and organic matter leads to a “pseudo-persistent” behavior, with frequent detection in agricultural water bodies and soils [4,5]. Similar to other environmental contaminants such as atrazine, these compounds can trigger complex cellular stress response pathways that extend beyond simple toxicity effects [6]. As critical accumulation vectors, plants can markedly influence FQ migration and transformation in ecosystems [7,8], raising concerns for both environmental and human health.

Despite extensive research on antibiotic resistance gene transmission in environmental settings, our understanding of how environmental antibiotics impact plant health remains limited [9]. FQ effects on crop safety, particularly at environmentally relevant concentrations, are especially unclear. Studies across major crop species have revealed notable phytotoxicity patterns [10]. In hydroponic systems, high-dose FQ exposure at 300 mg l^−1^ reduced tomato root and shoot length by over 50%, while even at a low concentration of 0.01 mg l^−1^, lettuce growth was inhibited by 15% to 27% [11]. In soil-based studies, ofloxacin at 40 mg l^−1^ reduced tomato shoot length by 22.3% and decreased chlorophyll content by 18.7% [12]. Similar effects were observed in onion crops [13]. Wheat seedlings also showed sensitivity to FQs, with exposure leading to substantial reductions in germination rates and biomass [14]. However, the molecular mechanisms underlying these observed effects remain largely unexplored, limiting our ability to accurately assess ecological risks and potential impacts on agricultural productivity.

The current understanding of FQ toxicity in plants largely focuses on phenotypic changes and basic stress responses such as reactive oxygen species (ROS) regulation [15]. Studies have demonstrated that FQ exposure disrupts cellular redox balance, as evidenced by elevated oxidative stress markers in tomato leaves exposed to ofloxacin [12] and altered H_2_O_2_ metabolism in *Lemna minor* L. under ciprofloxacin (CIP) stress [16]. However, emerging evidence suggests that plant responses to pharmaceutical stress operate through integrated networks spanning multiple biological scales. Chloroplasts serve as primary xenobiotic sensors, initiating cascading responses that extend beyond simple detoxification, while endophytic microbial communities contribute to stress tolerance through complementary mechanisms including xenobiotic transformation and enhanced stress resistance. The coordination between cellular and microbial responses in plant defense strategies represents a critical knowledge gap [17,18]. While antioxidant defense mechanisms and redox signaling networks have been characterized [19], protein-level responses to xenobiotic stress require further investigation. Given the complex nature of plant cellular systems and their response to xenobiotic stress, investigating protein-level changes is crucial not only for understanding plant-specific defense mechanisms against FQ exposure but also for revealing conserved biological principles that may be applicable across diverse organisms facing xenobiotic challenges.

Beyond cellular responses, the potential role of plant-associated microbiomes in antibiotic adaptation represents a critical yet underexplored dimension. Recent studies have highlighted how plant microbiomes contribute markedly to host fitness under various stress conditions [20], functioning as an extended phenotype that enhances adaptive capacity. Endophytic bacteria, as nonpathogenic microorganisms colonizing plant tissues and proliferating in intercellular spaces, are key components of this plant-associated microbiome [20]. These endophytes, analogous to beneficial bacteria in the human gut, perform specific biological functions particularly relevant to stress responses, enhancing plant tolerance to environmental challenges and contributing to xenobiotic transformation [21,22]. “Plant–microbiome responses” refer specifically to the temporally coordinated but mechanistically distinct responses between plant cellular systems and endophytic bacterial community restructuring, rather than treating the plant-associated microbiome as a single functional entity. This plant–microbiome partnership may be particularly relevant for antibiotic exposure, where microbial metabolic diversity could complement plant detoxification mechanisms. However, changes in the host’s growth environment can alter the microbial population, affecting host–microbe interactions and microbiome function [23]. For instance, exposure to imazethapyr has been found to reduce the abundance of *Pseudomonadaceae* in *Arabidopsis thaliana*, weakening plant resistance to pathogenic bacteria [24]. Traditional studies examining isolated cellular, metabolic, or microbial responses fail to capture the integrated nature of plant adaptation. This study employs a systems biology approach combining ultrastructural analysis, proteomics, and microbiome profiling to characterize multiscale defense coordination.

This research characterizes multiscale plant defense strategies during xenobiotic stress using CIP exposure in rice seedlings. CIP, a widely prescribed FQ antibiotic listed in World Health Organization (WHO) Essential Medicines, serves as our model compound. We hypothesize that plants coordinate defense strategies through (a) chloroplast-centered cellular adaptation as the primary response mechanism, (b) tissue-specific accumulation and metabolic transformation, and (c) endophytic microbial community restructuring providing complementary stress tolerance. CIP, a widely and currently prescribed FQ antibiotic listed in WHO Essential Medicines, has been selected as the model FQ. In this study, we employ a systems biology approach integrating ultrastructural, proteomic, and microbiome analyses to elucidate the molecular mechanisms underlying plant adaptation to antibiotic stress, thereby addressing the existing knowledge gap and providing a comprehensive multiscale understanding of plant–microbiome interactions under xenobiotic exposure.

## Results and Discussion

### Phenotyping and physiological changes with an increase in CIP

**Fig. 1.**
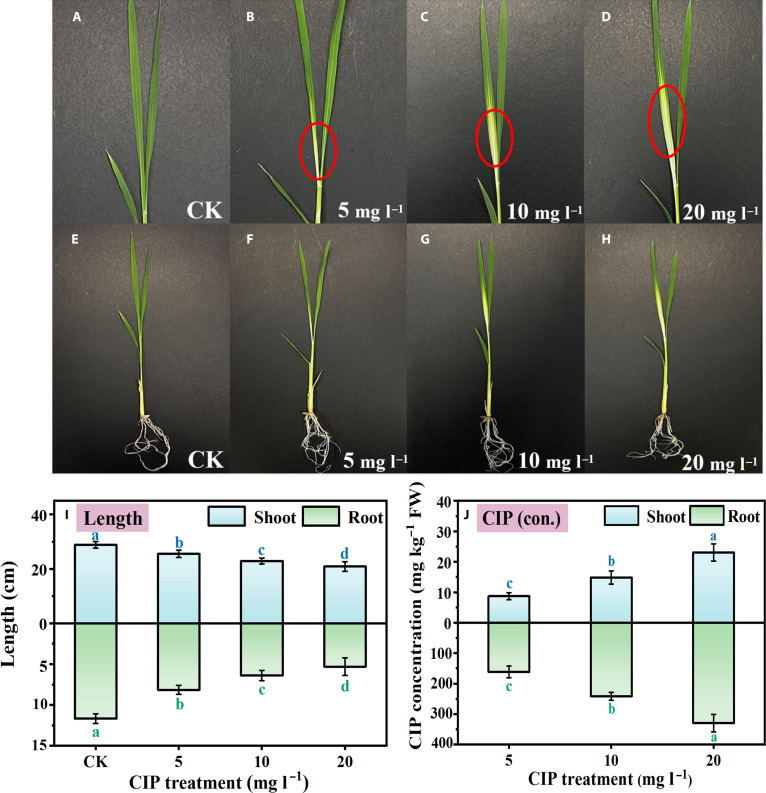
Effect of ciprofloxacin (CIP) exposure on rice seedlings. Shoots (A to D) and roots (E to H) of rice seedlings after 9-d exposure to CIP: (A and E) control (0 mg l^−1^) and (B and F) 5 mg l^−1^, (C and G) 10 mg l^−1^, and (D and H) 20 mg l^−1^ CIP. Shoot and root length (I) and CIP accumulation (J) changes after 9-d CIP exposure. Note: Data points and error bars represent mean ± standard deviation (SD), respectively. Different letters indicate significant differences (*P* < 0.05). CK, control group; FW, fresh weight.

Pronounced phenotypic changes were observed in rice seedlings following a 9-d hydroponic exposure to varying concentrations of CIP, with this timeframe capturing the critical window for early developmental effects (Fig. 1). The untreated control seedlings exhibited normal, healthy growth with uniformly green, straight leaves and well-developed root systems (Fig. 1A and E). In contrast, plants exposed to the highest CIP concentration of 20 mg l^−1^ displayed marked alterations, including pronounced leaf chlorosis at the base, leaf curling and deformation, and markedly reduced roots (Fig. 1D and H). As the CIP concentration increased from 5 to 20 mg l^−1^, gradual intensification of adverse effects was observed, including progressively more obvious leaf chlorosis, increasing leaf deformation, advanced reduction in root mass and branching complexity, a subtle but consistent decrease in overall plant size, and a gradual loss of green coloration in leaves (Fig. 1B to D and F to H).

Quantitative analysis further corroborated these observations, revealing substantial changes in plant growth parameters and dose-dependent CIP accumulation patterns (Fig. 1I and J). The 14-fold higher CIP accumulation in roots compared to that in shoots suggests active transport and compartmentalization mechanisms that may contribute to the observed differential stress responses across plant tissues. Shoot length decreased significantly from 30.2 ± 1.5 cm in untreated controls to 21.3 ± 1.2 cm at 20 mg l^−1^ CIP (*P* < 0.05). Root length also showed a statistically significant reduction, declining from 12.1 ± 0.8 to 5.2 ± 0.6 cm under the same conditions (*P* < 0.05). CIP accumulation increased significantly with increasing CIP dose. In shoots, the CIP content rose from 9.5 ± 0.7 mg kg^−1^ fresh weight (FW) at 5 mg l^−1^ exposure to 23.7 ± 1.3 mg kg^−1^ FW at 20 mg l^−1^ exposure (*P* < 0.05). Roots showed an even more pronounced increase, with the CIP content rising from 151.8 ± 7.2 to 336.5 ± 12.4 mg kg^−1^ FW over the same concentration range (*P* < 0.05), reaching levels 14-fold higher than that in shoots. These findings indicate that CIP exposure exerted dose-dependent phytotoxicity on rice seedling development, impacting both aboveground and belowground structures.

**Fig. 2.**
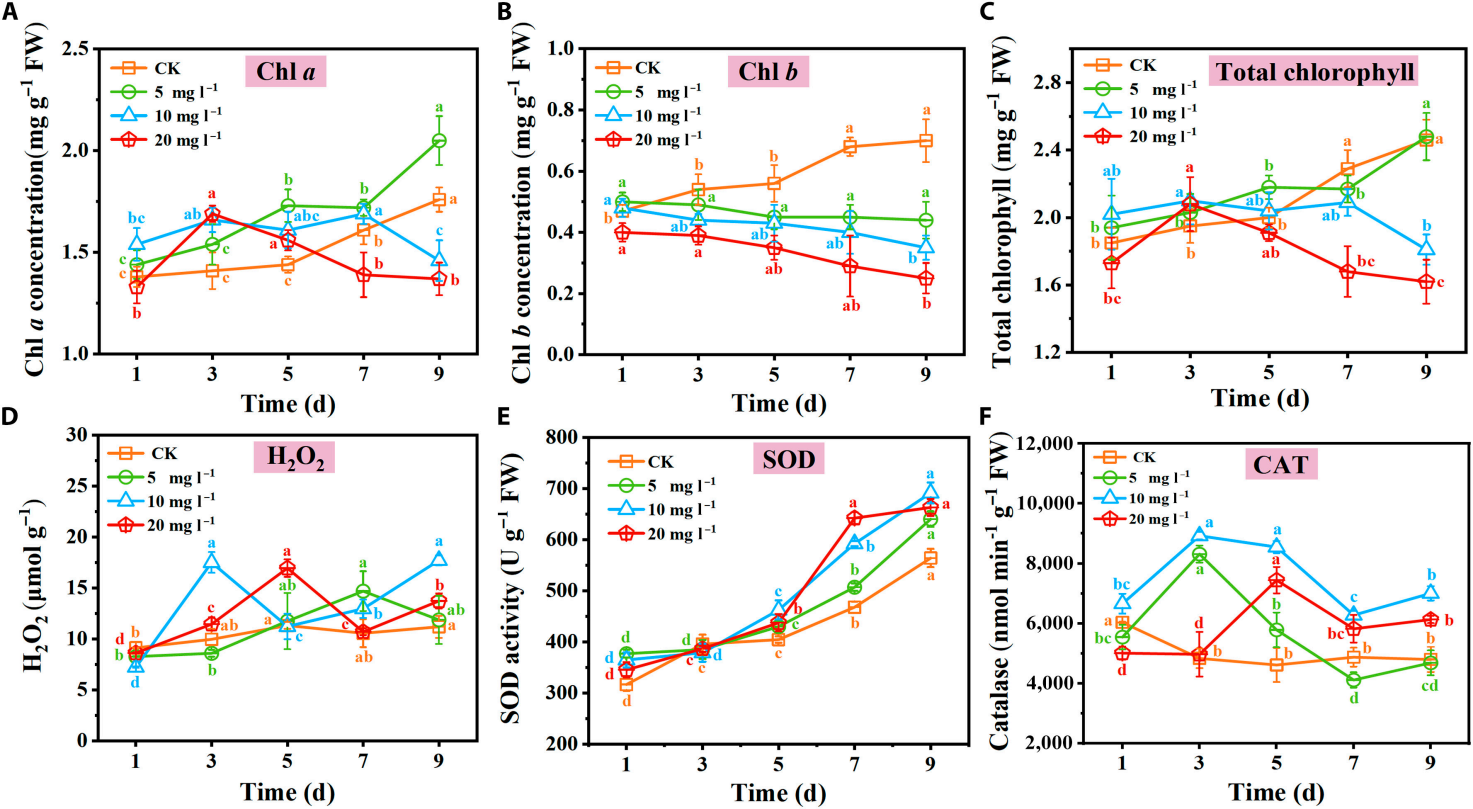
Concentrations of total chlorophyll (A), chlorophyll *a* (B), chlorophyll *b* (C), H_2_O_2_ (D), superoxide dismutase (SOD) (E), and catalase (CAT) (F) in rice plants following CIP treatment, measured on the 1st, 3rd, 5th, 7th, and 9th days. Note: Data points and error bars represent mean ± SD, respectively. Different letters (a, b, c, and d) indicate statistically significant differences among treatment groups (one-way analysis of variance followed by Tukey’s honestly significant difference [HSD] test, *P* < 0.05). Error bars represent SDs from 6 biological replicates (*n* = 6). Chl, chlorophyll.

Chlorophyll content analysis revealed contrasting trends between control and CIP-treated plants (Fig. 2A to C). In control plants, chlorophyll *a* increased from 1.38 mg g^−1^ on day 1 to 1.76 mg g^−1^ on day 9, while chlorophyll *b* rose from 0.47 to 0.70 mg g^−1^ over the same period. Under 20 mg l^−1^ CIP treatment, both chlorophyll *a* and *b* significantly decreased by day 9 (*P* < 0.05), showing 22.2% and 64.3% reductions compared to the control, respectively (Fig. 2A and B). For carotenoids, while control plants maintained stable levels, CIP-treated seedlings showed an elevated carotenoid content reaching 0.3671 mg g^−1^ (Fig. S1). The total chlorophyll content reflected these changes, with control levels increasing to 2.81 mg g^−1^ while CIP exposure led to a 30.6% decrease (Fig. 2C).

The physiological oxidative stress response of rice seedlings to CIP exposure showed distinct time- and dose-dependent patterns (Fig. 2D to F). H_2_O_2_ levels in control plants remained relatively stable, while 20 mg l^−1^ CIP induced rapid H_2_O_2_ accumulation, peaking at 16.97 μmol g^−1^ on day 5, representing an 85.7% increase compared to that of the control (Fig. 2D). Catalase (CAT) activity showed similar stress-responsive patterns, with 20 mg l^−1^ CIP triggering a 48.4% increase by day 9, while 10 mg l^−1^ CIP induced an even more pronounced response of 84.6% increase on day 3 (Fig. 2E). Superoxide dismutase (SOD) activity demonstrated unique kinetics under CIP stress—while control plants showed a baseline increase of 65.3% over 9 d, CIP-treated plants exhibited both an accelerated accumulation rate and higher final activity levels (Fig. 2F). These distinctive temporal patterns of antioxidant enzyme regulation, particularly the rapid CAT response and sustained SOD activation, reveal unique aspects of antibiotic-induced oxidative stress. The central role of ROS in this defense system extends beyond being merely stress indicators to serving as active agents in CIP transformation. Our findings reveal a dual function of ROS as signaling molecules that trigger defense responses and as direct chemical catalysts in xenobiotic transformation pathways. This functional duality may explain the observed upregulation of chloroplast-localized antioxidant enzymes, which are likely to modulate ROS levels for optimal signaling and transformation rather than simply minimizing oxidative damage. This regulated ROS production appears to be a cornerstone of the plant’s adaptation strategy, linking chloroplast function, metabolic transformation, and potentially even microbiome restructuring through altered cellular chemistry. Our quantitative data provide important perspective relative to existing plant antibiotic literature. Previous studies reported considerable biomass reductions in various crops under FQ exposure: 50% in tomato at 300 mg l^−1^ ofloxacin [12] and 22.3% shoot length reduction at 40 mg l^−1^ [13]. Our rice seedlings exhibited 29.4% shoot length and 57.0% root length reductions at 20 mg l^−1^ CIP, demonstrating substantial sensitivity to CIP stress even at relatively lower concentrations. While direct cross-species comparisons are constrained by differences in compounds, exposure conditions, and developmental stages, these findings contribute to the growing body of evidence documenting diverse plant responses to FQ contamination.

Additionally, our systems-level approach uncovered coordinated responses spanning from organellar adaptation to microbiome restructuring, revealing emergent properties not evident through single-scale analyses employed in previous studies. The quantitative correlations we established between cellular and microbial responses (*r* = 0.68 to 0.74) demonstrate sophisticated plant–microbiome communication mechanisms that collectively enhance stress tolerance.

These mechanistic insights establish a new paradigm for understanding plant pharmaceutical stress responses as integrated, multiscale phenomena. This framework provides a foundation for developing sustainable agricultural strategies to address antibiotic contamination while maintaining crop productivity.

### Chloroplasts as central response hubs in CIP stress adaptation

**Fig. 3.**
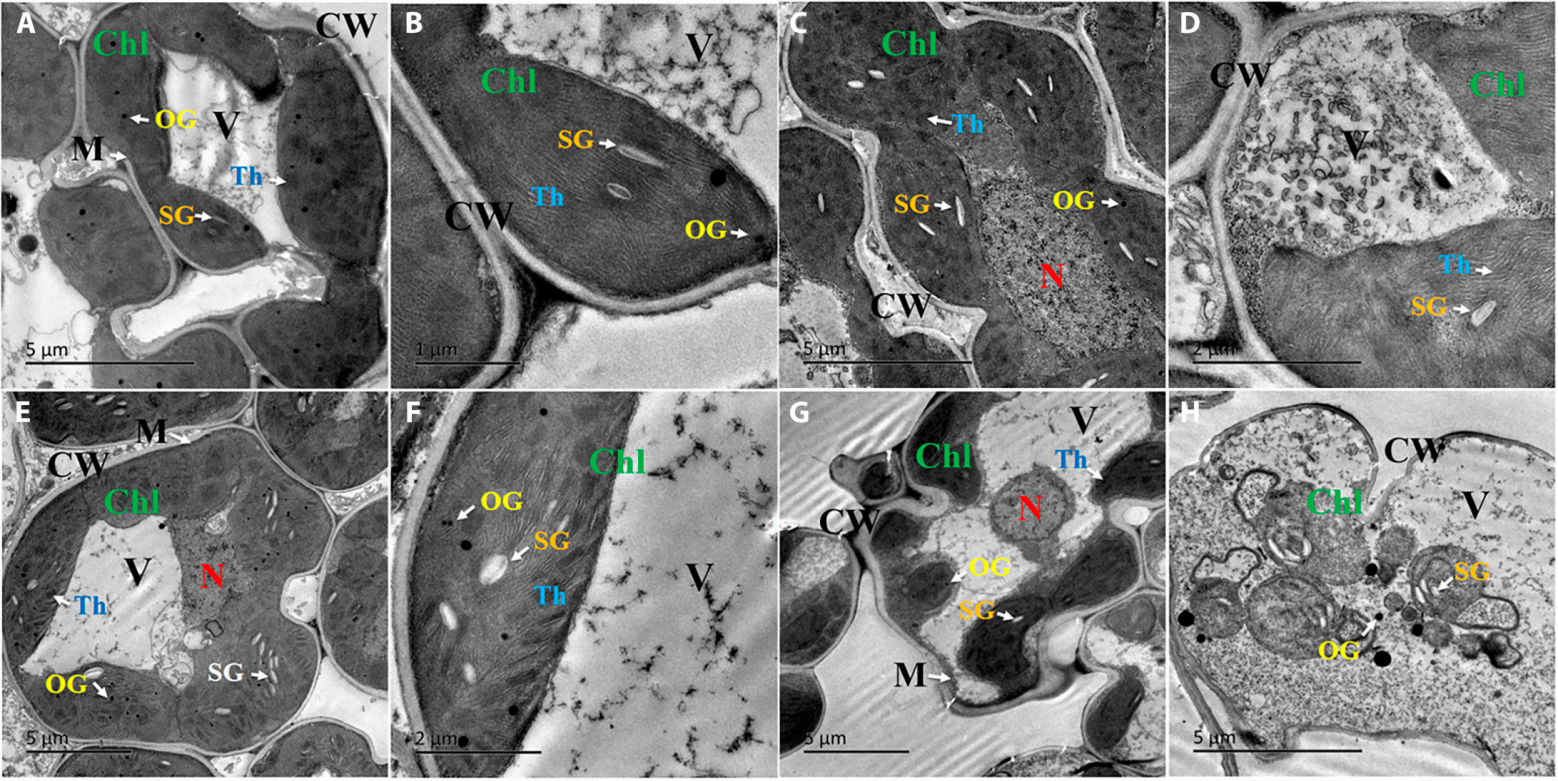
Transmission electron microscope (TEM) images of rice leaf subcellular structures after 9 d of CIP treatment. Control: (A) ×8,000 and (B) ×25,000; 5 mg l^−1^ CIP-treated samples: (C) ×8,000 and (D) ×15,000; 10 mg l^−1^ CIP-treated samples: (E) ×5,000 and (F) ×15,000; and 20 mg l^−1^ CIP-treated samples: (G) ×8,000 and (H) ×15,000. CW, cell wall; V, vacuole; Chl, chloroplast; Th, thylakoid; SG, starch grain; OG, osmiophilic globule; M, mitochondria; N, nucleus.

Chloroplasts emerged as central response hubs, showing both structural and functional alterations in response to CIP exposure. The predominance of chloroplast-related proteins (36% of differentially expressed proteins [DEPs]) represents a major advance over previous studies that focused on whole-cell stress markers, revealing chloroplasts as integrated stress sensing and processing centers. Ultrastructural analysis revealed progressive disruption of chloroplast architecture with increasing CIP concentration (Fig. 3C to H). In control plants, chloroplasts exhibited well-organized thylakoid membranes, distinct grana stacking, and well-defined starch grains (Fig. 3A and B). In contrast, CIP exposure led to chloroplast swelling, thylakoid membrane disorganization, and reduced starch grain accumulation, with the most severe alterations observed at 20 mg l^−1^ CIP (Fig. 3G and H).

**Fig. 4.**
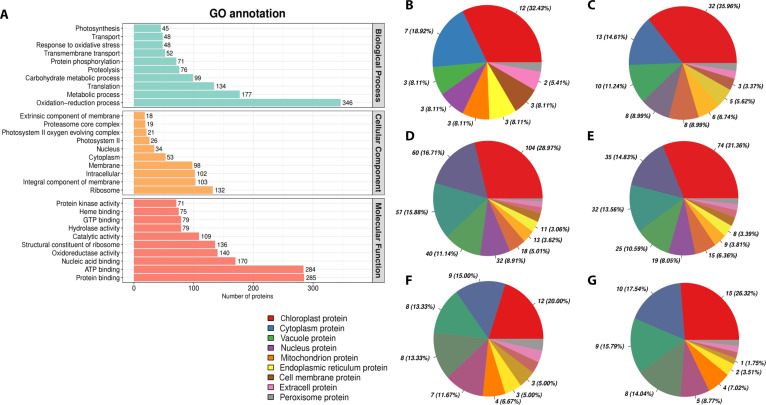
Analysis of differentially expressed proteins in rice leaves following CIP exposure at various concentrations. (A) Gene Ontology (GO) annotation of differentially expressed proteins categorized by Biological Process, Cellular Component, and Molecular Function. (B to G) Subcellular localization analysis of differentially expressed proteins: (B) 5 mg l^−1^ CIP vs. control, (C) 10 mg l^−1^ CIP vs. control, (D) 20 mg l^−1^ CIP vs. control, (E) 20 mg l^−1^ CIP vs. 5 mg l^−1^ CIP, (F) 20 mg l^−1^ CIP vs. 10 mg l^−1^ CIP, and (G) 10 mg l^−1^ CIP vs. 5 mg l^−1^ CIP. Pie charts show the percentage distribution of proteins across various cellular compartments. The color legend indicates the subcellular locations corresponding to each segment. This comprehensive analysis highlights the dose-dependent impacts of CIP on protein expression, function, and localization within rice leaf cells across different cellular compartments. ATP, adenosine triphosphate; GTP, guanosine-5′-triphosphate.

Following 9 d of CIP exposure, proteomic analysis revealed that chloroplast-related proteins constituted 36% of all DEPs, representing the predominant cellular response. Physiological monitoring throughout the exposure period showed that H_2_O_2_ accumulation peaked at day 5, while microbiome community restructuring was evident by day 9. These observations suggest that chloroplast responses, ROS dynamics, and microbiome changes represent coordinated components of the plant’s adaptive strategy, consistent with established chloroplast retrograde signaling mechanisms. Proteomic analysis revealed that chloroplast-related proteins constituted a substantial proportion of DEPs across all CIP treatments. This proportion showed a distinctive dose-dependent pattern, initially increasing from 32.43% at 5 mg l^−1^ to 35.96% at 10 mg l^−1^ CIP, followed by a decrease to 28.97% at 20 mg l^−1^ (Fig. 4B to D), suggesting potential threshold effects in chloroplast adaptation capacity. At the functional level, we observed concurrent regulation of 3 major chloroplast protein groups: photosynthetic apparatus components, redox regulation proteins, and metabolic enzymes.

Key upregulated photosynthetic proteins included the large subunit of ribulose-1,5-bisphosphate carboxylase/oxygenase (2.3-fold increase at 10 mg l^−1^ CIP), several light-harvesting complex proteins (1.8- to 2.7-fold increases), and photosystem II stability/assembly factor HCF136 (2.1-fold increase). This upregulation likely represents compensatory responses to maintain photosynthetic efficiency despite structural alterations. Concurrently, we observed enhanced expression of chloroplast-localized antioxidant enzymes including SOD (3.2-fold increase), ascorbate peroxidase (2.5-fold increase), and glutathione reductase (1.9-fold increase), indicating concurrent efforts to manage CIP-induced oxidative stress directly within the chloroplast [25]. This antioxidant upregulation aligns with recent findings that chloroplasts serve as primary sensors for xenobiotic stress and orchestrate subsequent cellular adaptation [26].

The chloroplast proteomic response also included substantial regulation of metabolic pathways, particularly those involved in carbon fixation, amino acid biosynthesis, and tetrapyrrole metabolism. Additionally, enzymes involved in chlorophyll biosynthesis showed differential regulation, with early biosynthetic steps (glutamyl-tRNA reductase and glutamate-1-semialdehyde aminotransferase) being upregulated while later steps (protochlorophyllide oxidoreductase and chlorophyll synthase) were down-regulated at higher CIP concentrations, consistent with the observed chlorosis in leaves (Fig. 1D). Interestingly, several chloroplast-localized proteins potentially involved in xenobiotic metabolism were also upregulated, including glutathione *S*-transferases (3.6-fold increase), cytochrome P450 enzymes (2.8-fold increase), and various esterases (1.7- to 2.4-fold increases). These enzymes could contribute directly to CIP transformation, as supported by our metabolite analysis and consistent with emerging evidence of chloroplast involvement in xenobiotic detoxification [27,28]. Convergent multi-omics evidence supports these mechanistic conclusions [29,30]. The predominant involvement of chloroplast proteins (36% of DEPs) provides compelling evidence for chloroplast-centered detoxification mechanisms. This finding aligns with emerging understanding of chloroplasts as cellular “chemical factories” capable of xenobiotic processing through photosystem-generated reducing power [26]. The concurrent upregulation of key detoxification enzymes (glutathione *S*-transferase, peroxidase, and CAT) and adenosine triphosphate synthase subunits suggests coordinated metabolic reprogramming to enhance detoxification capacity. The observed ultrastructural changes, including thylakoid membrane reorganization and increased plastoglobuli, provide morphological evidence supporting enhanced metabolic activity in chloroplasts under CIP stress. The assessment of reduced toxicity for CIP transformation products is based on structure–activity relationship analysis of the identified metabolites. CIP’s antimicrobial activity depends critically on its intact quinolone ring system, particularly the carboxylic acid at C-3 and the keto group at C-4, along with the cyclopropyl substituent at the N-1 position [31,32]. The transformation products identified in our study—phenylalanine, phenylpyruvic acid, 3-aminoisobutyric acid, 4-fluorocinnamic acid, and benzaldehyde—represent breakdown products lacking these essential structural features for antimicrobial activity. Phenylalanine and phenylpyruvic acid, in particular, are naturally occurring amino acid metabolites with well-documented low toxicity profiles in biological systems [33]. However, we acknowledge that direct toxicity testing of these transformation products on rice seedlings and microbial communities would provide more definitive evidence and represents an important direction for future research.

Metabolic analysis confirmed that seedlings actively transform CIP through 2 primary degradation pathways. The first pathway involves ROS-mediated oxidation of the piperazine ring, while the second proceeds through deamination. Both pathways generate metabolites with progressively decreased toxicity compared to that of the parent compound, demonstrating the plant’s capacity for xenobiotic transformation (Fig. S3). The identification of specific CIP transformation products, including phenylalanine, phenylpyruvic acid, 4-fluorocinnamic acid, and benzaldehyde, particularly through the ROS-mediated pathway as shown in Fig. S4, confirms the active role of these metabolic pathways in xenobiotic detoxification. These findings provide direct evidence that the upregulated chloroplast enzymes contribute to the systematic transformation of CIP within the plant.

Notably, the chloroplast response occurred alongside concomitant changes in mitochondria and cytoplasm, indicating an integrated cross-organellar response. Mitochondria showed marked proteome changes, particularly at higher CIP concentrations, with upregulation of key components of the electron transport chain, such as NADH-ubiquinone oxidoreductase and cytochrome c oxidase subunits, suggesting an increased energy demand (Table S6). Several mitochondrial heat shock proteins and their chaperones were also upregulated, indicating enhanced protein quality control. The concurrent upregulation of glycolytic enzymes in the cytoplasm (e.g., fructose-bisphosphate aldolase and pyruvate kinase) and electron transport components in mitochondria indicates synchronized enhancement of cellular energy production under stress. Recent research has highlighted such inter-organellar signaling networks as crucial for plant adaptation to environmental challenges [34,35].

Nuclear proteins showed progressive response to CIP exposure, with their proportion among DEPs increasing from 8.11% at 5 mg l^−1^ to 15.88% at 20 mg l^−1^ (Fig. 4B to D). Upregulated nuclear proteins involved in transcription regulation, chromatin remodeling, and stress response orchestrate the cellular adaptation to CIP stress [36]. The endoplasmic reticulum showed enhanced protein quality control through upregulation of heat shock proteins and protein disulfide isomerases, while peroxisomal proteins involved in fatty acid oxidation and ROS detoxification were also upregulated. Vacuolar proteins showed complex changes, with differential regulation of hydrolases and transporters, reflecting concurrent adjustments in both cellular detoxification processes and ion homeostasis. This collective evidence suggests that chloroplasts function not merely as targets of CIP toxicity but as active centers for both stress adaptation and potentially xenobiotic transformation. Recent studies demonstrate that chloroplast-derived ROS serve as retrograde signaling molecules triggering comprehensive cellular stress responses [37]. This chloroplast-centered response, coupled with coordinated changes across multiple cellular compartments and microbiome restructuring, indicates how plants deploy multitiered defense strategies against antibiotic stress. This chloroplast-centered adaptation response represents the plant’s first line of defense against CIP stress. However, as antibiotic concentrations increase, rice seedlings appear to deploy additional defense strategies beyond cellular adaptation. The restructuring of the endophytic bacterial community emerges as a complementary response that may contribute to both CIP tolerance and transformation, suggesting sophisticated coordination between plant metabolism and microbial partnerships.

### Microbiome restructuring complements cellular adaptation

**Fig. 5.**
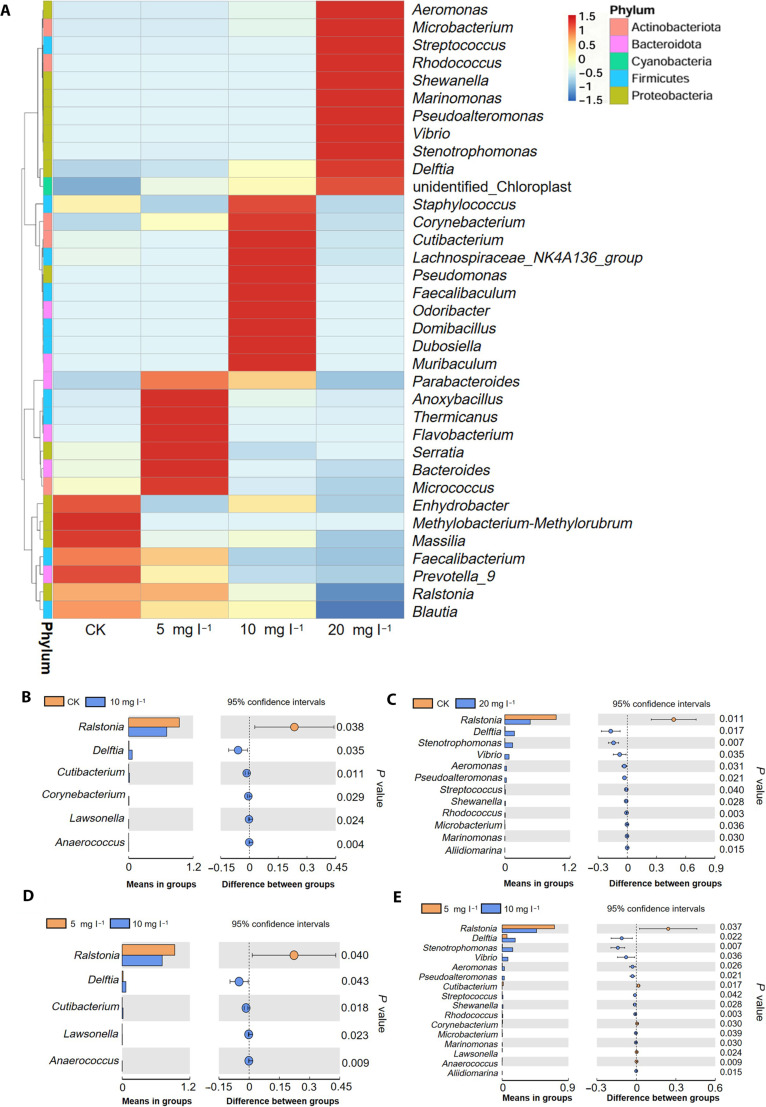
Analysis of bacterial community composition and differential abundance across treatments. (A) Heatmap showing relative abundance of bacterial genera across different treatments. The color scale indicates abundance levels from low (blue) to high (red). Genera are clustered based on abundance patterns. Colored bars on the left indicate phylum-level classification. Differential abundance analysis of select genera between treatments pairs (B to E): (B) 10 mg l^−1^ vs. CK, (C) 20 mg l^−1^ vs. CK, (D) 5 mg l^−1^ vs. 10 mg l^−1^, and (E) 10 mg l^−1^ vs. 20 mg l^−1^. Left panels show mean abundance in each group, while right panels display the difference in abundance between groups with 95% confidence intervals; genera with statistically significant differences are highlighted.

As CIP concentrations exceeded cellular adaptation capacity, rice seedlings deployed a complementary strategy through endophytic community restructuring. The 16S ribosomal RNA (rRNA) sequencing analysis of rice endophytes revealed notable shifts in bacterial community composition with increasing CIP exposure (Fig. 5A). In the controls, genera such as *Methylobacterium-Methylorubrum*, *Massilia*, and *Faecalibacterium* showed high abundance. As the CIP concentration increased to 5 mg l^−1^, these initially dominant genera began to decrease, while others such as *Anoxybacillus* and *Thermicanus* showed increased abundance. At 10 mg l^−1^ CIP, further reductions in *Methylobacterium-Methylorubrum* and *Massilia* were observed, coinciding with notable increases in *Staphylococcus* and *Corynebacterium*. The most dramatic changes occurred at 20 mg l^−1^ CIP, where *Aeromonas*, *Microbacterium*, *Streptococcus*, and *Rhodococcus* exhibited a marked increase in abundance, while the initially dominant genera continued to decline. Throughout this progression, some genera such as *Blautia* showed a consistent decrease in abundance as CIP levels rose. These observations indicate a progressive and complex restructuring of the microbial community in response to increasing CIP concentrations, where direct antibiotic pressure favors potentially resistant species while suppressing those dominant under normal conditions.

Detailed analysis revealed distinct thresholds in community response to CIP exposure (Fig. 5B to E). No substantial differences were observed between the control and 5 mg l^−1^ CIP treatment. At 10 mg l^−1^ CIP, the first notable shifts emerged, with significant decrease in *Ralstonia* abundance and increases in *Delftia*, *Cutibacterium*, *Corynebacterium*, *Lawsonella*, and *Anaerococcus* (*P* < 0.05), although the increase magnitude appears relatively small (Fig. 5B). The most dramatic changes occurred at 20 mg l^−1^ CIP, where *Ralstonia* exhibited a marked decrease compared to the control, while *Delftia*, *Stenotrophomonas*, and *Vibrio* showed marked increases (Fig. 5C). Other genera, including *Aeromonas*, *Pseudoalteromonas*, *Streptococcus*, *Shewanella*, *Rhodococcus*, *Microbacterium*, and *Marinomonas*, also displayed noticeable increases at 20 mg l^−1^ CIP (Fig. 5D). The comparison between 10 and 20 mg l^−1^ CIP further highlighted these trends, with *Ralstonia* continuing to decrease and genera such as *Delftia*, *Stenotrophomonas*, and *Vibrio* showing pronounced increases (Fig. 5E).

The selective enrichment of specific bacterial genera represents functionally driven recruitment rather than passive selection. The enrichment of *Rhodococcus* species is consistent with their reported xenobiotic degradation capabilities in the literature, suggesting potential contributions to CIP transformation through documented metabolic pathways [38]. *Aeromonas* strains contribute through multidrug efflux systems and β-lactamase production that directly reduce local antibiotic concentrations around plant tissues [39,40]. *Microbacterium* species are associated with oxidoreductase and deaminase activities that may contribute to CIP biotransformation based on reported metabolic capabilities [41]. Multi-omics correlation analysis revealed strong associations between bacterial community restructuring and plant molecular responses. The abundance of stress-resistant genera (*Aeromonas*, *Rhodococcus*, and *Microbacterium*) showed strong positive correlations with plant cytochrome P450 enzyme expression (*r* = 0.68 to 0.74, *P* < 0.01), while major facilitator superfamily (MFS) transporter protein levels correlated positively with *Aeromonas* abundance (*r* = 0.71, *P* < 0.01), indicating coordinated xenobiotic metabolism and synchronized efflux mechanisms [42]. These correlations reflect underlying signaling mechanisms coordinating plant–microbiome responses. Furthermore, multi-omics analysis revealed that oxidative stress markers correlated inversely with microbiome diversity (*r* = −0.72, *P* < 0.01), supporting ROS-mediated communication pathways [43,44]. Concurrently, secondary metabolism pathway activation indicates metabolite-mediated microbiome regulation, whereby CIP stress alters plant chemical signatures to selectively promote stress-tolerant taxa [45]. This coordinated plant–microbiome response represents an integrated defense strategy where bacterial metabolic capabilities complement plant cellular adaptations. The selective recruitment of antibiotic-resistant genera with demonstrated xenobiotic transformation abilities, coupled with the temporal synchronization of plant and microbial stress responses, indicates active biological coordination rather than coincidental changes. These findings suggest that endophytic bacteria function as an extended metabolic network that enhances plant tolerance to pharmaceutical contamination through shared detoxification mechanisms and metabolic complementarity.

Remarkably, despite CIP accumulation, the inner leaf pH remains relatively stable; this stability is crucial for maintaining cellular function under xenobiotic stress. Hydroponic experiments measuring leaf pH showed that CIP exposure did not markedly alter pH at lower concentrations within 5 to 10 mg l^−1^, with values remaining around 6.0 to 6.1 (Fig. S2B and C). At the highest CIP of 20 mg l^−1^, a slight decrease in pH to 5.9 ± 0.05 was observed, although this change was not statistically significant (*P* > 0.05) (Fig. S2C). These values remained within the typical range of 5.7 to 6.3 for a healthy inner leaf pH [46], suggesting that CIP exposure, even at higher concentrations, does not substantially disrupt the plant’s internal pH homeostasis.

**Fig. 6.**
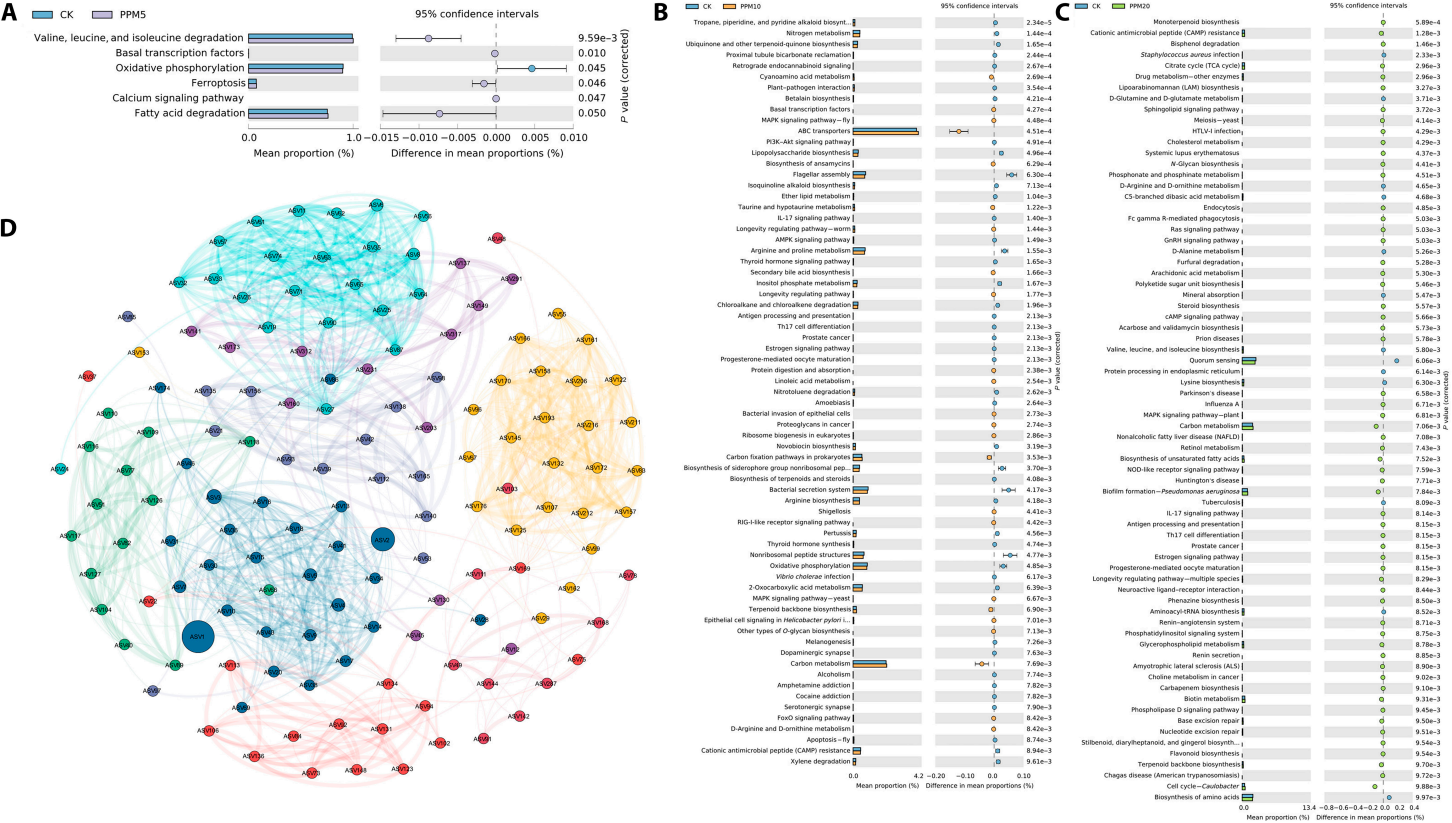
Multipanel analysis of biological pathways and network interactions. Differential expression analysis of key genes in biological processes. (A) CK vs. 5 mg l^−1^ CIP, (B) CK vs. 10 mg l^−1^ CIP, and (C) CK vs. 20 mg l^−1^ CIP. (D) Network visualization of gene interactions. The figure combines statistical analyses with pathway enrichment and network topology to provide a comprehensive view of the biological system under study. PPM5, 5 mg l^−1^ CIP; PPM10, 10 mg l^−1^ CIP; PPM20, 20 mg l^−1^ CIP; ASV, amplicon sequence variant.

The metabolic response to CIP exposure was characterized through transcriptome analysis (Fig. 6A to C). The activated pathways align with previously reported mechanisms of CIP biotransformation [47]. Key pathway changes included modifications in oxidative phosphorylation and tyrosine metabolism [48]. These metabolic alterations intensified with increasing CIP concentration from 5 to 20 mg l^−1^, suggesting enhanced engagement of transformation processes at higher exposure levels.

The formation of conjugates, a common detoxification strategy in many organisms, may be inferred from the alterations in glutathione metabolism pathways [49]. This could indicate the production of glutathione–CIP conjugates as part of the degradation process, although further metabolomic studies would be required to confirm this hypothesis. Of particular interest is the change observed in the “drug metabolism–cytochrome P450” pathway, which directly corresponds to the oxidation reactions catalyzed by cytochrome P450 enzymes, known to play a key role in xenobiotic metabolism [50]. This finding supports the involvement of these enzymes in CIP degradation, potentially contributing to various transformation processes such as hydroxylation and N-oxidation.

Examination of specific genes involved in CIP response revealed coordinated regulation of multiple defense mechanisms (Tables S8 and S9). The MerR (mercuric resistance regulator) family transcriptional regulator (K21902) showed substantial upregulation, consistent with its role in xenobiotic response [51]. Additionally, we observed enhanced expression of transport-related genes, including the MFS transporters of the DHA1 (drug:H⁺ antiporter 1) family (K08161 to K08164) and efflux pump components (K18899, K18902, and K18908). These transport system modifications suggest active cellular efforts to regulate CIP accumulation [52].

The involvement of the cytochrome P450 family (K21199 to K21201) in CIP degradation corresponds directly with the observed changes in the drug metabolism pathway [53,54]. These enzymes are known to catalyze various oxidation reactions, including hydroxylation and N-oxidation, which are key steps in CIP transformation [55]. Network analysis of the endophytic community reveals distinct functional modules that respond coordinately to CIP stress (Fig. 6D). The modular structure suggests that certain bacterial groups share resistance mechanisms or metabolic functions, with key taxa serving as connecting nodes across modules in the co-occurrence network [45]. Amplicon sequence variants (ASVs) representing *Aeromonas* and *Rhodococcus* occupy prominent positions with extensive connections within the network, indicating their potential roles as keystone species in community restructuring under antibiotic stress [56]. This network modularity aligns with functional coordination patterns documented in synthetic community studies, where microbial assemblages reflect biochemical cooperation rather than random selection [57]. As CIP concentrations increase, we observed changes in the connectivity and centrality of certain ASVs, indicating shifts in the community structure and potential keystone species. For instance, ASV1 and ASV2 show a high degree of centrality and betweenness, suggesting that they may play crucial roles in maintaining community structure across different CIP concentrations. The modularity of the network (visible as clusters in the image) suggests that certain groups of bacteria may respond to CIP stress in a coordinated manner, potentially sharing resistance mechanisms or metabolic functions.

### The fate of CIP in the micro universe

Our integrated analysis reveals that rice seedlings respond to CIP exposure not through isolated stress reactions but through a remarkably integrated, multi-level defense system that spans from subcellular machinery to microbial partnerships. This study provides the first comprehensive evidence of how plants orchestrate responses to antibiotic stress across biological scales, suggesting sophisticated adaptation mechanisms that intensify and coordinate as CIP concentrations increase from environmentally relevant to potentially phytotoxic levels. This response pattern aligns with established plant–microbiome signaling mechanisms [58].

This multi-level defense strategy follows a hierarchical progression corresponding to increasing CIP concentrations. At the foundation lies strategic compartmentalization, with roots accumulating substantially more CIP than shoots with 14-fold differences, representing a protective mechanism limiting antibiotic translocation to photosynthetic tissues. At the cellular level, chloroplasts function as major response centers, participating in both stress sensing and xenobiotic transformation through the upregulation of key proteins involved in ROS management and metabolic adaptation. The first pathway involves ROS-mediated oxidation of the piperazine ring, while the second proceeds through deamination processes. Both generate metabolites with progressively reduced toxicity, suggesting active xenobiotic detoxification rather than passive tolerance. The ROS pathway, in particular, generates intermediates including phenylalanine and phenylpyruvic acid, which serve as biomarkers of this detoxification process.

As CIP concentrations increase from 5–10 to 20 mg l^−1^ (higher levels), the response extends beyond cellular adaptation to include marked restructuring of the endophytic bacterial community. The shift toward CIP-resistant genera such as *Aeromonas*, *Microbacterium*, and *Rhodococcus* likely represents concurrent plant–microbiome responses to enhance both tolerance and transformation capacity. The network analysis of bacterial communities reveals distinct functional modules that respond coordinately to CIP stress, suggesting sophisticated interspecies interactions that complement plant metabolic responses. These findings establish plants as active bioremediation agents rather than passive victims of pharmaceutical pollution.

Cellular adaptations are evident from ultrastructural and proteomic analyses, revealing marked changes in organelle structure and function, particularly in chloroplasts and mitochondria [59]. Metabolic reprogramming is indicated by the upregulation of various pathways involved in CIP transformation, including lysine degradation and nitrogen metabolism [48]. Multiple detoxification mechanisms are activated, including alterations in glutathione metabolism pathways [50] and upregulation of cytochrome P450 enzymes for hydroxylation and N-oxidation processes [60]. The identification of specific CIP transformation products, particularly the formation of phenylalanine and phenylpyruvic acid intermediates, confirms the active role of these metabolic pathways in xenobiotic detoxification. Simultaneously, the endophytic bacterial community undergoes strategic restructuring toward antibiotic-tolerant species, with network analysis suggesting concurrent plant–microbe responses to CIP transformation.

**Fig. 7.**
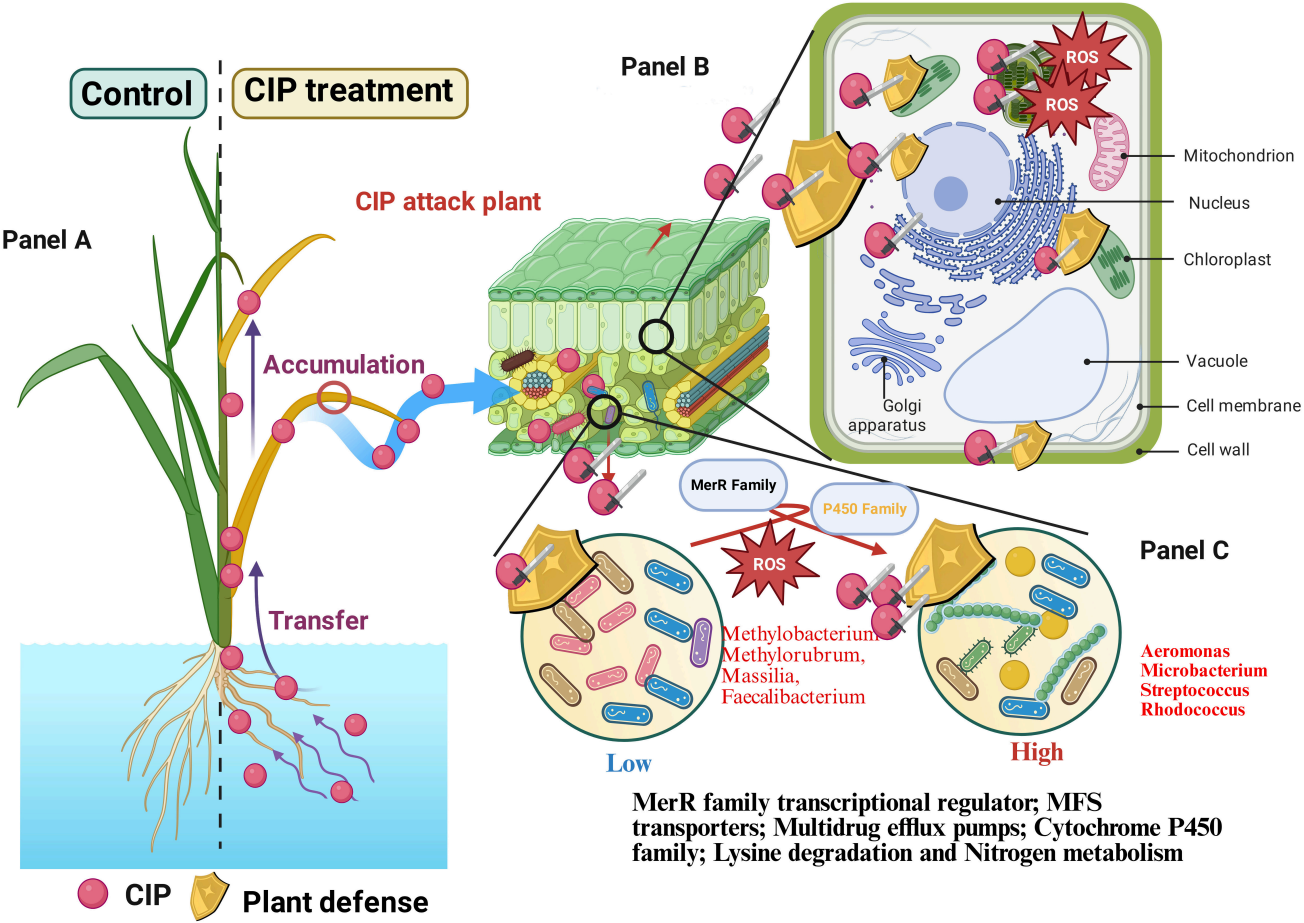
Schematic representation of CIP accumulation and its effects on coordinated plant cellular and endophytic bacterial responses. Note: The figure illustrates the transfer and accumulation of CIP in plant tissues, cellular responses, and shifts in microbial community composition under low and high CIP concentrations. Key functional genes and metabolic pathways involved in CIP response are listed. (A) CIP compartmentalization (root vs. shoot distribution), (B) chloroplast-centered cellular response (integrating ultrastructural and proteomic data), and (C) microbiome restructuring and CIP transformation pathways (showing parallel/complementary roles of plant and bacteria). ROS, reactive oxygen species; MerR, mercuric resistance regulator; MFS, major facilitator superfamily.

This integrated analysis, summarized in Fig. 7, indicates how plants deploy this multi-level defense strategy. At low CIP concentrations of 5 to 10 mg l^−1^, plants primarily respond through cellular adaptations, including enhanced membrane transport systems and activation of antioxidant mechanisms. As CIP levels increase from 10 to 20 mg l^−1^, a more comprehensive response emerges, involving both intensified cellular defenses and substantial microbiome restructuring. At the same time, chloroplasts orchestrate the plant’s adaptation response, as evidenced by the 35.96% of DEPs being chloroplast related, which contribute to both ROS defense and photosynthetic adjustments. The confirmed ROS-mediated CIP transformation pathway aligns with this chloroplast-centered response. Simultaneously, the endophytic community shifts toward CIP-tolerant species, particularly *Aeromonas*, *Microbacterium*, and *Rhodococcus*, that may contribute to xenobiotic transformation, creating an integrated multicompartmental defense system.

Importantly, our findings strongly suggest that biological stress responses operate as integrated, multiscale systems that integrate cellular, metabolic, and ecological mechanisms. This systems-level integration represents a fundamental property of biological adaptation that extends beyond individual organisms to encompass their associated microbiomes, indicating emergent properties invisible to single-scale approaches. The identification of specific CIP transformation products and pathways has important implications for environmental monitoring and remediation strategies. The ROS-mediated oxidation pathway targeting the piperazine ring appears particularly efficient at detoxification, as these metabolites show progressively reduced toxicity compared to the parent compound. The concurrent changes in gene expression patterns observed in our study support the emerging paradigm that transcriptional responses can serve as sensitive biomarkers for environmental stress, as demonstrated in plant–pathogen stress systems [61]. The identified transformation pathways could serve as biomarkers for monitoring antibiotic degradation in agricultural systems and as targets for enhancing natural remediation processes. The compartmentalization of CIP between roots and shoots further strongly suggests the plant’s strategic response, with differential accumulation patterns that may protect photosynthetic tissues while allowing for specialized degradation processes. These mechanisms have potential agricultural applications for managing pharmaceutical contamination.

This study focused on 21-d-old seedlings, and we acknowledge that plant antibiotic stress responses vary substantially across developmental stages [14,62]. Seedlings show heightened vulnerability due to developing defense mechanisms, a smaller biomass for compound dilution, and immature antioxidant systems [63]. Conversely, mature plants possess more robust detoxification systems, greater biomass ratios, and enhanced compartmentalization capabilities that may alter CIP accumulation and transformation efficiency. The differing physiological demands between rapidly growing seedlings and established plants may also affect resource allocation toward stress responses. Additional studies should examine CIP responses across developmental stages to provide comprehensive understanding throughout the plant life cycle.

While our study focused specifically on CIP stress, we acknowledge the limitation of single-stressor design in establishing response specificity. However, CIP’s unique molecular target through DNA gyrase inhibition and the distinctive chloroplast-centered response pattern that we observed suggest stressor-specific mechanisms distinct from other environmental stresses, such as heavy metal or salinity. The discovery of coordinated plant–microbiome responses provides a methodological framework and theoretical foundation for subsequent comparative studies across diverse environmental stressors. Our findings contribute to understanding both universal stress response principles and CIP-specific adaptation mechanisms. Our proposed signaling network—from chloroplast stress perception to nuclear gene expression to microbiome restructuring—integrates established retrograde signaling and plant–microbiome communication principles, representing a paradigm shift in plant stress biology. This multi-omics analysis provides mechanistic insights into coordinated chloroplast and microbiome responses to xenobiotic stress. The findings suggest that plants function as active transformation agents rather than passive stress recipients, with potential applications in developing pharmaceutical-resilient agricultural systems.

## Conclusion

This systems biology investigation reveals that plants deploy multiscale defense strategies against CIP stress through coordinated chloroplast-mediated adaptation and microbiome restructuring. Chloroplasts emerged as central response hubs orchestrating both cellular signaling and xenobiotic transformation, while endophytic communities shifted toward stress-resistant taxa that complement plant detoxification mechanisms. Rice seedlings progressively intensified defense responses with increasing antibiotic concentrations, progressing from cellular adaptations to comprehensive plant–microbiome coordination.

These findings establish plants as active bioremediation agents with implications for pharmaceutical-resilient agricultural systems. Chloroplast-centered mechanisms provide molecular targets for breeding or engineering crops with enhanced stress tolerance, while plant–microbiome coordination indicates opportunities for beneficial endophyte optimization. Future research should examine tissue-specific responses and validate findings across multiple plant species and xenobiotic compounds to advance the understanding of plant stress adaptation mechanisms.

## Materials and Methods

### Plant materials and CIP treatment

Rice seedlings (*Oryza sativa* L. cv. Nanjing 9108), obtained from the Jiangsu Academy of Agricultural Sciences Seed Station, were used in this study. Seeds were surface-sterilized with 3% H_2_O_2_ for 5 min, thoroughly rinsed with ultrapure water, and imbibed overnight. After germination on deionized-water-soaked gauze for 7 d, seedlings were transferred to black plastic containers with Hoagland’s nutrient solution for 14 d under controlled conditions (16/8 h light/dark photoperiod, 400 μmol m^−2^ s^−1^ light intensity, 25/20 °C (day/night) temperature, 60% relative humidity). The nutrient solution was renewed every 4 d.

Uniform seedlings were selected and divided into control and treatment groups. The control group (CK) was cultivated in Hoagland’s solution (pH 5.5) without CIP, while treatment groups were exposed to 5, 10, and 20 mg l^−1^ CIP in Hoagland’s solution (pH 5.5) for 9 d. Triplicates were established for each treatment (*n* = 3, providing 80% power to detect 2-fold changes at *α* = 0.05 based on preliminary variance estimates). Solutions were replenished every 2 d to maintain initial volumes and concentrations. CIP (98% purity) was procured from Aladdin Reagent Co., Ltd., China.

The 9-d exposure period was selected based on preliminary time-course experiments showing that this timeframe captured stable, measurable changes in plant physiology while allowing sufficient time for proteomic and metabolomic adaptations to develop. Previous studies on antibiotic effects in crop plants have demonstrated that major physiological responses typically stabilize within 7 to 10 d of exposure [64]. While earlier time points (1, 3, 5, and 7 d) would provide valuable information about transient responses, particularly for ROS dynamics and enzyme activities, our focus was on characterizing the established adaptive responses that represent the plant’s stable physiological adjustment to CIP stress.

The concentration range of 5 to 20 mg l^−1^ employed in this study reflects environmental relevance based on documented pharmaceutical contamination in agricultural systems. Recent investigations by Qi et al. [10] reported CIP concentrations reaching milligrams per liter levels in agricultural irrigation waters affected by livestock operations. Additionally, bioaccumulation factors in soil–plant systems can result in plant tissue concentrations considerably exceeding bulk environmental levels [6]. This concentration range enables mechanistic investigation while maintaining some environmental relevance for understanding plant responses to pharmaceutical contamination in agricultural settings.

The selected CIP concentrations (5, 10, and 20 mg l^−1^) were chosen to examine dose-dependent plant responses from moderate to high stress levels [61]. CIP (98% purity, Aladdin Reagent Co., Ltd., Shanghai, China) was dissolved directly in Hoagland’s solution without organic solvents due to its aqueous solubility (>30 mg l^−1^ at pH 5.5 to 6.0). Stock solutions (100 mg l^−1^) were prepared in amber bottles and stored at 4 °C to minimize photodegradation. Working solutions were freshly prepared from stock every 48 h and immediately applied to maintain consistent exposure concentrations throughout the 14-d experimental period under controlled light conditions (16-h photoperiod, 400 μmol m^−2^ s^−1^).

Leaf tissues were prioritized for molecular analysis to investigate chloroplast-centered adaptive signaling [26] and plant–endophyte coordination [22]. Although compartmentalization mechanisms limit CIP translocation to leaves [32], proteomic analysis revealed that leaves mount the predominant organellar response, validating this tissue selection for understanding systemic stress adaptation.

### Chloroplast ultrastructure observation

Leaf samples from control and CIP-treated (5, 10, and 20 mg l^−1^) rice seedlings were collected after 9 d of exposure. Leaf segments (1 mm^2^) were immediately fixed in 3% (w/v) glutaraldehyde in 0.05 mM phosphate buffer (pH 7.2) for 24 h at 4 °C, followed by postfixation in 2% osmium tetroxide for 2 h. Samples were dehydrated through an acetone/ethanol gradient series, infiltrated, and embedded in London Resin. Ultrathin sections (5.0 to 6.5 nm) were cut using a UCT-GA-D/E-1/00 ultramicrotome, stained with uranyl acetate, and examined using a Hitachi H-3000 N transmission electron microscope.

### Determination of CIP content in leaves

The acetone/dichloromethane extraction method was selected over conventional methanol-based protocols due to superior matrix cleanup efficiency for rice leaf tissues, which contain high levels of chlorophyll and lipophilic compounds that interfere with CIP quantification [12,13]. Method validation yielded recovery rates of 85.3% ± 4.2%, with limits of detection and quantification of 0.05 and 0.15 mg kg^−1^ FW, respectively. Matrix-matched calibration standards were employed to minimize matrix effects, achieving linearity with *r*^2^ > 0.999 and relative standard deviation (SD) < 5% for replicate analyses. CIP stability in hydroponic solutions was monitored throughout the cultivation period. CIP stability in hydroponic solutions was assessed throughout the cultivation period. Fresh nutrient solutions containing CIP were renewed every 2 d to maintain stable exposure conditions. Solution samples were collected immediately before each renewal and analyzed using the same high-performance liquid chromatography (HPLC) protocol described above. Results demonstrated that CIP concentrations retained 92.0% to 94.0% of initial values within each 2-d interval, confirming minimal degradation or adsorption losses and ensuring consistent exposure concentrations throughout the experimental period.

Approximately 1 g (FW) of rice leaves was homogenized and extracted thrice with 10 ml of an acetone/dichloromethane (1:1 v/v) mixture using ultrasonication for 30 min each time. The extract was filtered through anhydrous sodium sulfate and a silica gel column, followed by elution with 10 ml of hexane/dichloromethane (1:1 v/v). The combined eluate was evaporated to dryness at 40 °C using a vacuum rotary evaporator. The residue was reconstituted in 2 ml of HPLC-grade methanol and filtered through a 0.22-μm membrane for HPLC analysis. HPLC analysis was performed using a Thermo Fisher Scientific U3000 system equipped with an ultraviolet detector and an Agilent Zorbax SB-C18 column (250 mm × 4.6 mm × 5 μm). The mobile phase consisted of 20% acetonitrile and 80% water, with a flow rate of 1.0 ml min^−1^. The column temperature was maintained at 30 °C, the injection volume was 10 μl, and CIP was detected at 279 nm. The CIP concentration was calculated using a standard curve (*r*^2^ > 0.99).

For analyzing CIP transformation products, leaf samples from seedlings exposed to 20 mg l^−1^ CIP were collected after 9 d of treatment. Fresh leaves were immediately homogenized and extracted with 75% methanol. The extracts were analyzed using a Triple TOF 5600 system equipped with an Acquity UPLC HSS T3 column. Metabolites were identified using accurate mass measurements and tandem mass spectrometry (MS/MS) fragmentation patterns. The mass spectra of transformation products were obtained by subtracting the spectra of control samples from those of CIP-treated samples to eliminate background signals. Transformation products were characterized based on their mass-to-charge ratios (*m*/*z*) and relative abundances, with pathway analysis performed to determine the degradation routes.

### Oxidative stress response

#### H_2_O_2_ level

The H_2_O_2_ content in rice tissues was determined spectrophotometrically. Fresh shoot samples (0.5 g) were homogenized in 5 ml of 0.1% trichloroacetic acid solution. The homogenate was centrifuged (12,000 g, 4 °C, 10 min), and the supernatant was used for H_2_O_2_ determination. The reaction mixture contained 0.5 ml of the extract, 0.5 ml of phosphate buffer (0.1 M, pH 7.0), and 1 ml of 1 mM potassium iodide solution. After 1-h incubation in darkness, the absorbance was measured at 390 nm [65].

#### Antioxidant enzyme activity

Fresh leaf samples (500 mg) were homogenized in 4 ml of 50 mM phosphate buffer (pH 7.8) containing 0.2 mM ethylenediaminetetraacetic acid, 0.1 mM ascorbate, and 1% (w/v) polyvinylpyrrolidone. The homogenate was centrifuged at 14,800 g for 20 min at 4 °C. SOD activity was assayed by measuring its ability to inhibit the photochemical reduction of nitro blue tetrazolium at 560 nm. CAT activity was determined by monitoring the decrease in H_2_O_2_ absorbance at 240 nm according to Xu et al. [66].

#### Chlorophyll and carotenoids

Leaf pigments were extracted using a 95% ethanol–acetone (1:1 v/v) mixture. Absorbance was measured at 663, 645, 646, and 470 nm using a UV-6100 spectrophotometer (Shanghai Mapada Instruments Co., Ltd., China). Pigment concentrations were calculated using standard equations [67].

### Label-free quantitative proteomics

#### Total protein extraction

Samples were individually ground in liquid nitrogen and lysed with SDS–DTT–Tris (SDT) buffer (containing 100 mM NaCl) and 1/100 volume of dithiothreitol (DTT), followed by 5-min ultrasonication on ice. After incubation at 95 °C for 8 to 15 min and an ice bath for 2 min, lysates were centrifuged at 12,000 g for 15 min at 4 °C. Supernatants were alkylated with iodoacetamide for 1 h at room temperature in darkness. Samples were then mixed with 4 volumes of pre-cooled acetone, vortexed, and incubated at −20 °C for ≥2 h. After centrifugation (12,000 g, 15 min, 4 °C), precipitates were collected, washed with cold acetone, and dissolved in dissolution buffer. The protein extraction method was that of Xu et al. [66] and Marx et al. [68]. Three biological replicates were used, consistent with established proteomics protocols for controlled plant stress studies.

#### Protein quality assessment

Bovine serum albumin standard solutions (0 to 0.5 g l^−1^) were prepared according to Bradford protein quantification kit instructions. Standard and sample solutions were added to 96-well plates (20 μl per well) in triplicate. A G250 dye solution (180 μl) was added, and absorbance at 595 nm was measured after 5 min. Protein concentrations were calculated using a standard curve. In addition, sodium dodecyl sulfate–polyacrylamide gel electrophoresis (12%) was performed with 20 μg of the protein/sample (80 V for 20 min, 120 V for 90 min). Gels were stained with Coomassie Brilliant Blue R-250 and destained until bands were clearly visible.

#### Trypsin digestion

Protein samples were diluted to 100 μl with dissolution buffer (8 M urea, 100 mM triethylammonium bicarbonate [TEAB], pH 8.5). Trypsin and TEAB buffer were added for digestion at 37 °C (4 h), followed by overnight digestion with additional trypsin and CaCl_2_. Formic acid was added (pH < 3), and samples were centrifuged (12,000 g, 5 min, ambient temperature). The supernatants were desalted using C18 columns, washed (0.1% formic acid, 3% acetonitrile), and eluted (0.1% formic acid, 70% acetonitrile). Eluates were collected and lyophilized.

#### Fractionation for high-depth quantification

A gradient elution was developed using mobile phases A (2% acetonitrile, pH 10.0) and B (98% acetonitrile, pH 10.0). The lyophilized powder was dissolved in solution A and centrifuged at 12,000 g for 10 min at room temperature. Fractionation was performed on a Rigol L3000 HPLC system with a C18 column (Waters BEH C18, 4.6 × 250 mm, 5 μm) at 45 °C. Eluates were monitored at 214 nm, collected per minute, and combined into 10 fractions. All fractions were vacuum-dried and reconstituted in 0.1% formic acid.

#### Liquid chromatography–MS/MS analysis

Analyses were performed using an EASY-nLC 1200 UHPLC system coupled with a Q Exactive HF-X mass spectrometer (Thermo Fisher). The mobile phases were A (water, 0.1% formic acid) and B (80% acetonitrile, 0.1% formic acid). Samples were injected into a C18 Nano-Trap column (4.5 cm × 75 μm, 3 μm) at 55 °C. Peptides were separated on an analytical column (15 cm × 150 μm, 1.9 μm) using a linear gradient. The mass spectrometry parameters were as follows: Nanospray Flex (electrospray ionization) ion source, 2.1-kV spray voltage, 320 °C ion transport capillary temperature; full scan: *m*/*z* 350 to 1,500, resolution 60,000 (at *m*/*z* 200), automatic gain control (AGC) target 3 × 10^6^, and maximum injection time 20 ms. The top 40 precursors were selected for higher-energy collisional dissociation fragmentation and MS/MS analysis (resolution 15,000, AGC target 1 × 10^5^, maximum injection time 45 ms, normalized collision energy 27%, intensity threshold 2.2 × 10^4^, and dynamic exclusion 20 s).

#### Protein sequencing data analysis

Three biological replicates were used per treatment. The Proteome Discoverer software was used for database searches and protein quantification. DEPs were identified by a *t* test (*P* < 0.05, fold change > 1.5). Gene Ontology (GO) and InterPro (IPR) functional annotations were performed using InterProScan. Clusters of Orthologous Groups and Kyoto Encyclopedia of Genes and Genomes (KEGG) analyses were conducted for protein family and pathway analysis. Cluster analysis [69], GO/IPR/KEGG pathway enrichment [70], and protein–protein interaction predictions were performed for DEPs using appropriate software tools [71]. The details of elution gradients are shown in Table S7.

### Endophytic bacteria

#### DNA extraction and polymerase chain reaction amplification

Rice leaves were surface-sterilized by washing with distilled water (30 s), 75% ethanol (2 min), 2.5% NaClO (5 min), and 75% ethanol (30 s), followed by 5 to 6 rinses with sterile water. Bacterial DNA was extracted using the cetyltrimethylammonium bromide method [72]. The 16S rRNA gene V5 to V7 region was amplified using the primers 799F and 1193R. Polymerase chain reaction products were analyzed by 2% agarose gel electrophoresis, purified, and quantified. Equimolar amounts of purified amplicons were pooled and sequenced using NovaSeq 6000 (Illumina) by Novogene Co., Ltd.

#### Bioinformatic analysis of sequencing data

Raw sequencing data were processed to remove interference and obtain clean data. DADA2 was used for denoising to obtain ASVs. Taxonomic annotation was performed for representative ASV sequences. Alpha diversity indices were calculated, and shared/unique ASVs among samples were identified. Permutational multivariate analysis of variance (PERMANOVA) was used to statistically test community composition differences, and network analysis metrics (modularity and centrality) were employed to quantify community restructuring. *t* tests were used to analyze significant differences in community composition and structure between groups.

### Statistical analysis

All data were analyzed using pre-specified statistical protocols. Data normality was assessed using Shapiro–Wilk tests and Q–Q plots. Normally distributed data are presented as mean ± SD, and nonnormally distributed data, as median and interquartile range. For physiological and biochemical parameters, one-way analysis of variance (ANOVA) followed by Tukey’s honestly significant difference multiple comparison test was performed, with Bonferroni correction applied for planned comparisons (*P* < 0.05). For proteomics data, DEPs were identified using 2-sample *t* tests with Benjamini–Hochberg false discovery rate (FDR) correction (FDR < 0.05). Microbiome data were analyzed using PERMANOVA with 999 permutations. Effect sizes are reported as Cohen’s *d* for continuous variables where appropriate. All analyses were performed using SPSS 26.0 unless otherwise specified. Results represent 6 biological replicates, with sample sizes reported in individual figure legends. Missing data (<2% of total) were handled by complete case analysis.

## Data Availability

All data are available in the main text and the Supplementary Materials. Additional data are available from Yu Shen (sheyttmax@hotmail.com or yushen@njfu.edu.cn) upon reasonable request.

## Supplementary Materials

Supplementary 1 Description of Supplemental Methods Figs. S1 to S4 Tables S1 to S9
