# Supplementary material for: Multiscale Plant Defense Strategies against Ciprofloxacin Stress: From Chloroplast-Centered Adaptation to Microbiome Coordination
Source: Research (Wash D C). 2026 Jan 15;9:1082. doi: 10.34133/research.1082 (PMC12804602; doi:10.34133/research.1082)
Supplement: Supplementary 1 — Description of Supplemental Methods Figs. S1 to S4 Tables S1 to S9 [file research.1082.f1.zip › Supplemental Information Table S8.pdf]

Table S8. Gene

KO	CK	5 ppm	10 ppm	20 ppm	description
K00001	0.00044616	0.000443723	0.000444673	0.000418565	alcohol dehydrogenase [EC:1.1.1.1]
K00002	3.12816E-06	8.61912E-07	9.41332E-06	5.59051E-06	alcohol dehydrogenase (NADP+) [EC:1.1.1.2]
K00003	0.000222911	0.000222254	0.00022887	0.000211137	homoserine dehydrogenase [EC:1.1.1.3]
K00004	1.09285E-05	1.64361E-05	6.33841E-05	0.000172	(R,R)-butanediol dehydrogenase / meso-butanediol dehydrogenase / diacetyl reductase [EC:1.1.1.4 1.1.1.- 1.1.1.303]
K00005	1.23845E-06	7.15225E-07	4.60489E-06	7.8201E-06	glycerol dehydrogenase [EC:1.1.1.6]
K00007	1.28624E-06	3.3022E-06	1.62261E-05	3.85141E-05	D-arabinitol 4-dehydrogenase [EC:1.1.1.11]
K00008	1.33434E-05	1.5692E-05	7.35998E-05	0.000246387	L-iditol 2-dehydrogenase [EC:1.1.1.14]
K00009	4.5783E-07	6.73646E-07	1.76201E-06	1.55994E-05	mannitol-1-phosphate 5-dehydrogenase [EC:1.1.1.17]
K00010	0.000434046	0.000429013	0.000395042	0.000310553	myo-inositol 2-dehydrogenase / D-chiro-inositol 1-dehydrogenase [EC:1.1.1.18 1.1.1.369]
K00012	0.000223563	0.000223995	0.000210362	0.000214365	UDPglucose 6-dehydrogenase [EC:1.1.1.22]
K00013	0.000223116	0.000222952	0.00022747	0.000223681	histidinol dehydrogenase [EC:1.1.1.23]
K00014	0.000440839	0.000440917	0.000439021	0.000396893	shikimate dehydrogenase [EC:1.1.1.25]
K00015	0.000450294	0.000442881	0.000456647	0.000398911	glyoxylate reductase [EC:1.1.1.26]
K00016	5.02558E-06	5.67731E-06	3.19173E-05	1.21711E-05	L-lactate dehydrogenase [EC:1.1.1.27]
K00018	1.47643E-05	1.23222E-05	5.72868E-05	9.6534E-05	glycerate dehydrogenase [EC:1.1.1.29]
K00019	0.001531788	0.001526699	0.00148711	0.001429939	3-hydroxybutyrate dehydrogenase [EC:1.1.1.30]
K00020	0.000661152	0.000659804	0.00064551	0.000598479	3-hydroxyisobutyrate dehydrogenase [EC:1.1.1.31]
K00021	5.34814E-08	3.48385E-08	3.07299E-07	8.08538E-06	hydroxymethylglutaryl-CoA reductase (NADPH) [EC:1.1.1.34]
K00023	0.000881768	0.00087456	0.000863316	0.000922802	acetoacetyl-CoA reductase [EC:1.1.1.36]
K00024	0.000223714	0.000224102	0.00023537	0.000226523	malate dehydrogenase [EC:1.1.1.37]
K00027	0.000436316	0.000433296	0.000419909	0.000306475	malate dehydrogenase (oxaloacetate-decarboxylating) [EC:1.1.1.38]
K00029	0.000437613	0.000436826	0.000429991	0.000389203	malate dehydrogenase (oxaloacetate-decarboxylating)(NADP+) [EC:1.1.1.40]
K00030	0.000216461	0.000213973	0.00018977	0.000180082	isocitrate dehydrogenase (NAD+) [EC:1.1.1.41]
K00031	0.00043989	0.000440246	0.000427106	0.000411381	isocitrate dehydrogenase [EC:1.1.1.42]
K00032	1.50213E-06	4.99522E-07	9.39767E-07	1.08819E-05	phosphogluconate 2-dehydrogenase [EC:1.1.1.43]
K00033	6.13047E-06	6.83978E-06	1.64791E-05	8.14895E-05	6-phosphogluconate dehydrogenase [EC:1.1.1.44 1.1.1.343]
K00034	0.000452348	0.000449512	0.000451506	0.000562731	glucose 1-dehydrogenase [EC:1.1.1.47]
K00035	1.21906E-06	1.01649E-06	5.25438E-07	1.35208E-06	D-galactose 1-dehydrogenase [EC:1.1.1.48]
K00036	0.00022365	0.000225935	0.000221389	0.000221877	glucose-6-phosphate 1-dehydrogenase [EC:1.1.1.49 1.1.1.363]
K00038	0.00087258	0.000872053	0.000856886	0.000771689	3alpha(or 20beta)-hydroxysteroid dehydrogenase [EC:1.1.1.53]
K00039	0.000214048	0.000211996	0.000186966	0.000117572	ribitol 2-dehydrogenase [EC:1.1.1.56]
K00040	2.8926E-06	4.80716E-06	2.37511E-05	4.05118E-05	fructuronate reductase [EC:1.1.1.57]
K00041	5.77166E-07	1.07499E-06	2.8085E-06	5.47499E-06	tagaturonate reductase [EC:1.1.1.58]
K00042	0.000436936	0.000434798	0.000409992	0.000368163	2-hydroxy-3-oxopropionate reductase [EC:1.1.1.60]
K00043	0.000432586	0.00043096	0.000409494	0.000299472	4-hydroxybutyrate dehydrogenase [EC:1.1.1.61]
K00045	1.23311E-06	2.41672E-06	8.02361E-06	2.03809E-05	mannitol 2-dehydrogenase [EC:1.1.1.67]
K00046	0.000669304	0.000670093	0.000728263	0.000769269	gluconate 5-dehydrogenase [EC:1.1.1.69]
K00048	3.60787E-06	1.57892E-06	6.101E-06	1.24872E-05	lactaldehyde reductase [EC:1.1.1.77]
K00050	0.000216918	0.000215522	0.000203561	0.000148373	hydroxypyruvate reductase [EC:1.1.1.81]
K00052	0.000871093	0.000862915	0.0008163	0.000666504	3-isopropylmalate dehydrogenase [EC:1.1.1.85]
K00053	0.000223087	0.000222368	0.000227527	0.000226302	ketol-acid reductoisomerase [EC:1.1.1.86]
K00054	2.47327E-06	3.89349E-06	1.76994E-05	3.9959E-05	hydroxymethylglutaryl-CoA reductase [EC:1.1.1.88]
K00055	5.94574E-06	8.64585E-06	2.24901E-05	9.63079E-05	aryl-alcohol dehydrogenase [EC:1.1.1.90]
K00057	0.000224485	0.000227166	0.000228749	0.000228282	glycerol-3-phosphate dehydrogenase (NAD(P)+) [EC:1.1.1.94]
K00058	0.001119849	0.001112804	0.001168904	0.001091914	D-3-phosphoglycerate dehydrogenase / 2-oxoglutarate reductase [EC:1.1.1.95 1.1.1.399]
K00059	0.005745924	0.005742956	0.005769762	0.006092587	3-oxoacyl-[acyl-carrier protein] reductase [EC:1.1.1.100]
K00060	4.19536E-06	8.0672E-06	2.20567E-05	9.63628E-05	threonine 3-dehydrogenase [EC:1.1.1.103]
K00064	6.12628E-06	8.77815E-06	2.10871E-05	0.000141664	D-threo-aldose 1-dehydrogenase [EC:1.1.1.122]
K00065	0.000654236	0.000647169	0.000607822	0.000497937	2-dehydro-3-deoxy-D-gluconate 5-dehydrogenase [EC:1.1.1.127]
K00066	1.11297E-06	3.21713E-06	1.95455E-06	2.76811E-07	GDP-mannose 6-dehydrogenase [EC:1.1.1.132]
K00067	0.000221596	0.000223897	0.00022438	0.000230326	dTDP-4-dehydrorhamnose reductase [EC:1.1.1.133]
K00068	9.67928E-07	2.62551E-06	1.22E-05	2.4693E-05	sorbitol-6-phosphate 2-dehydrogenase [EC:1.1.1.140]
K00073	7.4572E-08	2.35864E-07	4.76294E-07	1.07689E-06	ureidoglycolate dehydrogenase (NAD+) [EC:1.1.1.350]
K00074	0.000449845	0.000454568	0.000465778	0.000446966	3-hydroxybutyryl-CoA dehydrogenase [EC:1.1.1.157]
K00075	0.000223668	0.0002246	0.000231086	0.000230212	UDP-N-acetylmuramate dehydrogenase [EC:1.3.1.98]
K00076	3.98393E-06	5.0877E-06	2.70871E-05	0.000101537	7-alpha-hydroxysteroid dehydrogenase [EC:1.1.1.159]
K00077	0.00065661	0.00065233	0.000647389	0.000557325	2-dehydropantoate 2-reductase [EC:1.1.1.169]
K00082	1.77107E-06	2.90479E-06	1.42058E-06	1.88989E-06	5-amino-6-(5-phosphoribosylamino)uracil reductase [EC:1.1.1.193]
K00086	3.77714E-06	1.45529E-06	3.75863E-06	7.97809E-06	1,3-propanediol dehydrogenase [EC:1.1.1.202]
K00087	2.41516E-06	2.22577E-06	9.87707E-06	2.75444E-05	xanthine dehydrogenase molybdenum-binding subunit [EC:1.17.1.4]
K00088	0.000229985	0.000234948	0.000261861	0.000294654	IMP dehydrogenase [EC:1.1.1.205]
K00090	0.000441296	0.000436367	0.00043854	0.000397057	glyoxylate/hydroxypyruvate/2-ketogluconate reductase [EC:1.1.1.79 1.1.1.81 1.1.1.215]
K00091	3.71041E-06	4.69988E-06	7.76219E-06	1.00538E-05	dihydroflavonol-4-reductase [EC:1.1.1.219]
K00094	2.85633E-08	1.73739E-08	7.08013E-08	4.53499E-07	galactitol-1-phosphate 5-dehydrogenase [EC:1.1.1.251]
K00096	1.13557E-06	1.0931E-06	1.21353E-05	2.19673E-07	glycerol-1-phosphate dehydrogenase [NAD(P)+] [EC:1.1.1.261]
K00097	0.000223313	0.000225566	0.000241699	0.000289194	4-hydroxythreonine-4-phosphate dehydrogenase [EC:1.1.1.262]
K00098	0.000215329	0.000213649	0.000196245	0.000126174	L-idonate 5-dehydrogenase [EC:1.1.1.264]
K00099	0.000222149	0.000222818	0.000224302	0.000222299	1-deoxy-D-xylulose-5-phosphate reductoisomerase [EC:1.1.1.267]
K00100	3.54183E-07	7.97619E-07	4.27363E-06	9.52602E-06	butanol dehydrogenase [EC:1.1.1.-]
K00101	1.26469E-05	1.82094E-05	8.49957E-05	0.000241761	L-lactate dehydrogenase (cytochrome) [EC:1.1.2.3]
K00102	0.000439504	0.000439851	0.000447787	0.000480785	D-lactate dehydrogenase (cytochrome) [EC:1.1.2.4]
K00103	2.73137E-07	6.93576E-07	1.94197E-06	1.11866E-05	L-gulonolactone oxidase [EC:1.1.3.8]
K00104	0.000660442	0.000664346	0.000678128	0.000683239	glycolate oxidase [EC:1.1.3.15]
K00105	6.10241E-08	8.03722E-08	2.65641E-07	3.23053E-06	alpha-glycerophosphate oxidase [EC:1.1.3.21]
K00108	0.000653306	0.00065419	0.000628207	0.000555373	choline dehydrogenase [EC:1.1.99.1]
K00109	0	1.52266E-07	2.01622E-07	1.15347E-07	2-hydroxyglutarate dehydrogenase [EC:1.1.99.2]
K00111	0.000226843	0.000232998	0.000288169	0.000404203	glycerol-3-phosphate dehydrogenase [EC:1.1.5.3]
K00112	1.43841E-06	6.27657E-07	8.86639E-06	1.3687E-05	glycerol-3-phosphate dehydrogenase subunit B [EC:1.1.5.3]
K00113	6.22742E-07	6.77887E-07	8.31417E-06	1.36522E-05	glycerol-3-phosphate dehydrogenase subunit C [EC:1.1.5.3]
K00114	4.40116E-06	1.17814E-06	3.26549E-06	1.19432E-06	alcohol dehydrogenase (cytochrome c) [EC:1.1.2.8]
K00116	0.000220837	0.000217476	0.000203526	0.00017282	malate dehydrogenase (quinone) [EC:1.1.5.4]
K00117	5.6002E-06	4.87889E-06	2.24136E-05	0.000111787	quinoprotein glucose dehydrogenase [EC:1.1.5.2]
K00118	0.000214632	0.000212549	0.00018881	0.000146531	glucose-fructose oxidoreductase [EC:1.1.99.28]
K00121	0.000234773	0.000239054	0.000262372	0.000395001	S-(hydroxymethyl)glutathione dehydrogenase / alcohol dehydrogenase [EC:1.1.1.284 1.1.1.1]
K00122	5.64294E-07	8.68407E-07	1.88321E-06	2.90273E-07	formate dehydrogenase [EC:1.17.1.9]

K00123	0.000448355	0.000444881	0.000452119	0.000510921	formate dehydrogenase major subunit [EC:1.17.1.9]
K00124	0.000439953	0.000438206	0.000432904	0.000377583	formate dehydrogenase iron-sulfur subunit
K00125	0	0	6.97714E-09	0	formate dehydrogenase (coenzyme F420) beta subunit [EC:1.17.98.3 1.8.98.6]
K00126	2.36771E-06	3.17633E-06	1.45141E-05	3.51754E-05	formate dehydrogenase subunit delta [EC:1.17.1.9]
K00127	0.000219631	0.000220217	0.000217715	0.000221562	formate dehydrogenase subunit gamma
K00128	0.002825122	0.002831902	0.002708218	0.002373532	aldehyde dehydrogenase (NAD+) [EC:1.2.1.3]
K00129	3.02691E-09	2.4556E-08	1.80196E-07	1.89115E-06	aldehyde dehydrogenase (NAD(P)+) [EC:1.2.1.5]
K00130	0.001091408	0.001095156	0.001078317	0.001126878	betaine-aldehyde dehydrogenase [EC:1.2.1.8]
K00131	1.27217E-06	1.79841E-06	5.74639E-06	9.08577E-06	glyceraldehyde-3-phosphate dehydrogenase (NADP+) [EC:1.2.1.9]
K00132	1.2423E-06	1.36264E-07	3.72454E-06	5.22422E-06	acetaldehyde dehydrogenase (acetylating) [EC:1.2.1.10]
K00133	0.000224322	0.000224454	0.000230226	0.00025219	aspartate-semialdehyde dehydrogenase [EC:1.2.1.11]
K00134	0.000227722	0.000231015	0.000239074	0.000276283	glyceraldehyde 3-phosphate dehydrogenase [EC:1.2.1.12]
K00135	0.001969638	0.001979735	0.00195936	0.001997863	succinate-semialdehyde dehydrogenase / glutarate-semialdehyde dehydrogenase [EC:1.2.1.16 1.2.1.79 1.2.1.20]
K00137	0.000218967	0.00022005	0.000213942	0.000199248	aminobutyraldehyde dehydrogenase [EC:1.2.1.19]
K00138	0.000228623	0.000232683	0.00026854	0.000385309	aldehyde dehydrogenase [EC:1.2.1.-]
K00140	0.001087893	0.001088217	0.001035573	0.000930342	malonate-semialdehyde dehydrogenase (acetylating) / methylmalonate-semialdehyde dehydrogenase [EC:1.2.1.18 1.2.
K00141	0.000432423	0.000431994	0.000404264	0.000334254	benzaldehyde dehydrogenase (NAD) [EC:1.2.1.28]
K00145	0.000225657	0.000225769	0.00024208	0.000256826	N-acetyl-gamma-glutamyl-phosphate reductase [EC:1.2.1.38]
K00146	0.00065946	0.000665208	0.00068185	0.000736689	phenylacetaldehyde dehydrogenase [EC:1.2.1.39]
K00147	0.000222904	0.00022308	0.000224641	0.000225276	glutamate-5-semialdehyde dehydrogenase [EC:1.2.1.41]
K00148	5.67553E-06	5.15212E-06	1.04884E-05	5.98711E-05	glutathione-independent formaldehyde dehydrogenase [EC:1.2.1.46]
K00150	1.42805E-06	3.36431E-06	9.07352E-07	8.95075E-07	glyceraldehyde-3-phosphate dehydrogenase (NAD(P)) [EC:1.2.1.59]
K00151	0.000653912	0.000657236	0.000644019	0.000583363	5-carboxymethyl-2-hydroxymuconic-semialdehyde dehydrogenase [EC:1.2.1.60]
K00152	8.83625E-09	9.07755E-08	5.83659E-08	2.4228E-08	salicylaldehyde dehydrogenase [EC:1.2.1.65]
K00153	7.6618E-06	1.41413E-05	5.02519E-05	0.000119069	S-(hydroxymethyl)mycothiol dehydrogenase [EC:1.1.1.306]
K00154	0.000433486	0.000434183	0.000405042	0.000352631	coniferyl-aldehyde dehydrogenase [EC:1.2.1.68]
K00156	7.1743E-06	1.17095E-05	3.06406E-05	0.000128145	pyruvate dehydrogenase (quinone) [EC:1.2.5.1]
K00158	3.07233E-06	3.24927E-06	8.17428E-06	2.8191E-06	pyruvate oxidase [EC:1.2.3.3]
K00161	0.0002233	0.000222812	0.000210931	0.00018648	pyruvate dehydrogenase E1 component alpha subunit [EC:1.2.4.1]
K00162	0.000226307	0.000225491	0.000216105	0.000192837	pyruvate dehydrogenase E1 component beta subunit [EC:1.2.4.1]
K00163	0.000222425	0.000224951	0.000233505	0.000291054	pyruvate dehydrogenase E1 component [EC:1.2.4.1]
K00164	0.000222102	0.00022233	0.000221446	0.000221758	2-oxoglutarate dehydrogenase E1 component [EC:1.2.4.2]
K00166	1.86432E-06	5.79365E-07	5.4881E-06	5.83461E-06	2-oxoisovalerate dehydrogenase E1 component alpha subunit [EC:1.2.4.4]
K00167	0.000220627	0.000221956	0.000205952	0.000148948	2-oxoisovalerate dehydrogenase E1 component beta subunit [EC:1.2.4.4]
K00169	1.24078E-06	8.63795E-07	7.95192E-06	1.18774E-07	pyruvate ferredoxin oxidoreductase alpha subunit [EC:1.2.7.1]
K00170	6.5236E-07	3.95045E-07	2.32828E-06	5.31346E-08	pyruvate ferredoxin oxidoreductase beta subunit [EC:1.2.7.1]
K00171	6.48389E-07	3.1839E-07	2.26531E-06	5.49191E-08	pyruvate ferredoxin oxidoreductase delta subunit [EC:1.2.7.1]
K00172	7.35188E-07	5.24251E-07	2.30247E-06	5.61416E-08	pyruvate ferredoxin oxidoreductase gamma subunit [EC:1.2.7.1]
K00174	2.34185E-06	2.45616E-06	1.59503E-05	1.93568E-06	2-oxoglutarate/2-oxoacid ferredoxin oxidoreductase subunit alpha [EC:1.2.7.3 1.2.7.11]
K00175	2.39693E-06	2.45567E-06	1.58849E-05	1.83564E-06	2-oxoglutarate/2-oxoacid ferredoxin oxidoreductase subunit beta [EC:1.2.7.3 1.2.7.11]
K00176	9.58029E-07	1.12895E-06	5.3599E-06	1.57904E-07	2-oxoglutarate ferredoxin oxidoreductase subunit delta [EC:1.2.7.3]
K00177	5.83679E-07	9.24651E-07	3.14301E-06	2.56876E-08	2-oxoglutarate ferredoxin oxidoreductase subunit gamma [EC:1.2.7.3]
K00179	0.000214314	0.000212716	0.00018905	0.000113457	indolepyruvate ferredoxin oxidoreductase, alpha subunit [EC:1.2.7.8]
K00180	0.000214196	0.000212675	0.000188828	0.000113873	indolepyruvate ferredoxin oxidoreductase, beta subunit [EC:1.2.7.8]
K00183	2.82321E-09	1.69162E-08	5.96804E-07	1.06745E-07	prokaryotic molybdopterin-containing oxidoreductase family, molybdopterin binding subunit
K00184	2.54837E-06	4.9802E-06	1.69271E-05	4.04588E-05	prokaryotic molybdopterin-containing oxidoreductase family, iron-sulfur binding subunit
K00185	1.13386E-06	1.0068E-06	7.4363E-07	8.7755E-08	prokaryotic molybdopterin-containing oxidoreductase family, membrane subunit
K00186	3.32167E-07	2.06608E-07	1.02817E-06	7.82206E-09	2-oxoisovalerate ferredoxin oxidoreductase, alpha subunit [EC:1.2.7.7]
K00187	3.31128E-07	1.34118E-07	3.12847E-07	7.82206E-09	2-oxoisovalerate ferredoxin oxidoreductase, beta subunit [EC:1.2.7.7]
K00188	9.9505E-08	2.17092E-07	0	0	2-oxoisovalerate ferredoxin oxidoreductase, delta subunit [EC:1.2.7.7]
K00196	5.8209E-08	6.74014E-08	6.35731E-07	1.46E-06	anaerobic carbon-monoxide dehydrogenase iron sulfur subunit
K00198	2.08165E-08	9.87999E-08	1.31622E-08	1.27188E-09	anaerobic carbon-monoxide dehydrogenase catalytic subunit [EC:1.2.7.4]
K00200	1.00317E-06	6.94065E-08	8.36939E-07	1.60403E-07	formylmethanofuran dehydrogenase subunit A [EC:1.2.7.12]
K00201	9.87446E-07	4.21631E-08	8.75913E-08	1.21796E-06	formylmethanofuran dehydrogenase subunit B [EC:1.2.7.12]
K00202	9.87446E-07	4.21631E-08	6.3322E-08	1.33207E-07	formylmethanofuran dehydrogenase subunit C [EC:1.2.7.12]
K00205	1.18375E-07	1.61696E-08	3.04155E-07	1.59212E-08	4Fe-4S ferredoxin
K00207	0	0	0	8.03716E-09	dihydropyrimidine dehydrogenase (NADP+) [EC:1.3.1.2]
K00208	0.00023188	0.000233729	0.000271628	0.00028962	enoyl-[acyl-carrier protein] reductase I [EC:1.3.1.9 1.3.1.10]
K00209	1.21832E-06	1.73367E-06	2.55443E-06	7.95034E-05	enoyl-[acyl-carrier protein] reductase / trans-2-enoyl-CoA reductase (NAD+) [EC:1.3.1.9 1.3.1.44]
K00210	1.86047E-06	1.31174E-06	4.43229E-06	4.17223E-07	prephenate dehydrogenase [EC:1.3.1.12]
K00211	0	0	0	1.53121E-08	prephenate dehydrogenase (NADP+) [EC:1.3.1.13]
K00213	0	0	1.04657E-08	2.67905E-09	7-dehydrocholesterol reductase [EC:1.3.1.21]
K00214	5.45116E-07	6.24417E-07	9.65717E-07	3.12162E-05	biliverdin reductase [EC:1.3.1.24]
K00215	0.00022383	0.000224156	0.000233212	0.000225621	4-hydroxy-tetrahydrodipicolinate reductase [EC:1.17.1.8]
K00216	0.000217858	0.000218198	0.000212709	0.000244937	2,3-dihydro-2,3-dihydroxybenzoate dehydrogenase [EC:1.3.1.28]
K00217	5.58637E-07	1.67089E-07	2.56554E-06	3.30444E-06	maleylacetate reductase [EC:1.3.1.32]
K00218	0.000214958	0.0002132	0.000189762	0.000118798	protochlorophyllide reductase [EC:1.3.1.33]
K00219	0.000660155	0.000661264	0.000640684	0.000613833	2,4-dienoyl-CoA reductase (NADPH2) [EC:1.3.1.34]
K00220	0.000217784	0.000213781	0.000190792	0.000114539	cyclohexadieny/prephenate dehydrogenase [EC:1.3.1.43 1.3.1.12]
K00221	1.4411E-07	5.6763E-07	2.52025E-07	2.11136E-06	alkylmercury lyase [EC:4.99.1.2]
K00222	0	0	1.04657E-08	0	Delta14-sterol reductase [EC:1.3.1.70]
K00226	2.01233E-06	1.26878E-06	1.30595E-05	3.79706E-06	dihydroorotate dehydrogenase (fumarate) [EC:1.3.98.1]
K00227	0	0	2.09314E-08	0	Delta7-sterol 5-desaturase [EC:1.14.19.20]
K00228	0.000219346	0.000217837	0.000207208	0.000217637	coproporphyrinogen III oxidase [EC:1.3.3.3]
K00230	9.95909E-07	3.08891E-06	1.0525E-06	1.79646E-05	menaquinone-dependent protoporphyrinogen oxidase [EC:1.3.5.3]
K00231	6.254E-06	1.13698E-05	4.81088E-05	8.08616E-05	protoporphyrinogen/coproporphyrinogen III oxidase [EC:1.3.3.4 1.3.3.15]
K00232	1.00421E-06	3.51938E-06	1.77094E-06	5.11897E-06	acyl-CoA oxidase [EC:1.3.3.6]
K00239	0.000225088	0.000225381	0.000234359	0.000238413	succinate dehydrogenase / fumarate reductase, flavoprotein subunit [EC:1.3.5.1 1.3.5.4]
K00240	0.00022305	0.000224117	0.000230059	0.0002299	succinate dehydrogenase / fumarate reductase, iron-sulfur subunit [EC:1.3.5.1 1.3.5.4]
K00241	0.00022284	0.000223423	0.000229597	0.000223448	succinate dehydrogenase / fumarate reductase, cytochrome b subunit
K00242	0.000220268	0.000220042	0.000208434	0.00022133	succinate dehydrogenase / fumarate reductase, membrane anchor subunit
K00243	1.8909E-06	2.34933E-06	6.9314E-06	2.29267E-05	uncharacterized protein
K00244	0.000218422	0.000214383	0.000216254	0.000169624	fumarate reductase flavoprotein subunit [EC:1.3.5.4]
K00245	1.10131E-07	3.46683E-07	7.02908E-07	1.37966E-05	fumarate reductase iron-sulfur subunit [EC:1.3.5.4]
K00246	1.10144E-07	3.46715E-07	6.34109E-07	1.52446E-05	fumarate reductase subunit C
K00247	1.10141E-07	3.46708E-07	4.05916E-07	1.23039E-05	fumarate reductase subunit D
K00248	0.001102849	0.001107316	0.001113944	0.001078485	butyryl-CoA dehydrogenase [EC:1.3.8.1]

K00249	0.001344534	0.001372295	0.001509188	0.001752958	acyl-CoA dehydrogenase [EC:1.3.8.7]
K00252	0.000455709	0.000484204	0.000572298	0.000772175	glutaryl-CoA dehydrogenase [EC:1.3.8.6]
K00253	0.000876279	0.0008794	0.000879362	0.000889409	isovaleryl-CoA dehydrogenase [EC:1.3.8.4]
K00254	0.000221688	0.000222221	0.000215278	0.000221643	dihydroorotate dehydrogenase [EC:1.3.5.2]
K00255	0.000216376	0.000216438	0.000205214	0.000161888	long-chain-acyl-CoA dehydrogenase [EC:1.3.8.8]
K00256	2.64698E-06	6.46655E-06	2.97028E-05	6.93969E-05	isoquinoline 1-oxidoreductase [EC:1.3.99.16]
K00257	0	5.09742E-07	5.09812E-07	2.13713E-06	acyl-ACP dehydrogenase [EC:1.3.99.-]
K00259	0.000219937	0.000219199	0.000214314	0.000148579	alanine dehydrogenase [EC:1.4.1.1]
K00260	0.000217356	0.000218869	0.000207428	0.000148261	glutamate dehydrogenase [EC:1.4.1.2]
K00261	0.000218862	0.000220325	0.000209035	0.000150832	glutamate dehydrogenase (NAD(P)+) [EC:1.4.1.3]
K00262	7.17286E-06	1.21571E-05	3.66664E-05	5.85142E-05	glutamate dehydrogenase (NADP+) [EC:1.4.1.4]
K00263	1.55222E-06	3.8213E-06	6.3118E-06	6.2853E-05	leucine dehydrogenase [EC:1.4.1.9]
K00265	0.000441516	0.000442385	0.000428136	0.000434183	glutamate synthase (NADPH/NADH) large chain [EC:1.4.1.13 1.4.1.14]
K00266	0.000229648	0.000234874	0.000280447	0.000322566	glutamate synthase (NADPH/NADH) small chain [EC:1.4.1.13 1.4.1.14]
K00270	0	9.5208E-10	5.60973E-07	5.18522E-08	phenylalanine dehydrogenase [EC:1.4.1.20]
K00271	7.34981E-07	1.52829E-06	7.82101E-06	4.9071E-06	valine dehydrogenase (NAD+) [EC:1.4.1.23]
K00273	8.78323E-08	1.98238E-08	2.50297E-07	1.01309E-07	D-amino-acid oxidase [EC:1.4.3.3]
K00274	0.00021742	0.000214142	0.000198023	0.000132525	monoamine oxidase [EC:1.4.3.4]
K00275	0.000434573	0.000431436	0.000395394	0.000336094	pyridoxamine 5-phosphate oxidase [EC:1.4.3.5]
K00276	7.51337E-08	2.14175E-07	8.52834E-07	1.30983E-06	primary-amine oxidase [EC:1.4.3.21]
K00278	0.000651923	0.000649022	0.000611104	0.00045864	L-aspartate oxidase [EC:1.4.3.16]
K00281	0.000222681	0.000223127	0.000225234	0.000221632	glycine dehydrogenase [EC:1.4.4.2]
K00282	0.000220704	0.00022182	0.00022095	0.000215255	glycine dehydrogenase subunit 1 [EC:1.4.4.2]
K00283	0.000218921	0.000217631	0.000202518	0.000166358	glycine dehydrogenase subunit 2 [EC:1.4.4.2]
K00284	0.00043976	0.000439657	0.00042725	0.000370937	glutamate synthase (ferredoxin) [EC:1.4.7.1]
K00285	0.000441234	0.000442589	0.000454458	0.000468871	D-amino-acid dehydrogenase [EC:1.4.5.1]
K00286	0.000224681	0.000226238	0.000229506	0.000240792	pyrroline-5-carboxylate reductase [EC:1.5.1.2]
K00287	0.0002247	0.000223626	0.000233444	0.000229343	dihydrofolate reductase [EC:1.5.1.3]
K00288	2.23408E-08	0	5.16189E-09	8.55783E-09	methylenetetrahydrofolate dehydrogenase (NADP+) / methenyltetrahydrofolate cyclohydrolase / formyltetrahydrofolat
K00290	1.64013E-06	2.82084E-06	5.69233E-06	8.21691E-06	saccharopine dehydrogenase (NAD+, L-lysine forming) [EC:1.5.1.7]
K00293	0	7.17609E-07	1.21979E-07	1.52139E-08	saccharopine dehydrogenase (NADP+, L-glutamate forming) [EC:1.5.1.10]
K00294	3.4449E-06	5.97507E-06	9.84401E-06	6.24845E-06	1-pyrroline-5-carboxylate dehydrogenase [EC:1.2.1.88]
K00297	0.000223464	0.000222153	0.000230778	0.00027886	methylenetetrahydrofolate reductase (NADPH) [EC:1.5.1.20]
K00298	5.89134E-08	1.66614E-09	4.63709E-08	1.126E-07	N5-(carboxyethyl)ornithine synthase [EC:1.5.1.24]
K00299	0.00022238	0.00022307	0.000227343	0.00029291	FMN reductase [EC:1.5.1.38]
K00301	2.79003E-07	2.48755E-07	6.42027E-07	1.43864E-07	sarcosine oxidase [EC:1.5.3.1]
K00302	0.000215105	0.000212766	0.000193294	0.000115885	sarcosine oxidase, subunit alpha [EC:1.5.3.1]
K00303	0.000430454	0.00042584	0.000381102	0.000231638	sarcosine oxidase, subunit beta [EC:1.5.3.1]
K00304	0.000214371	0.000211985	0.000187913	0.000114674	sarcosine oxidase, subunit delta [EC:1.5.3.1]
K00305	0.000213939	0.000211985	0.00018783	0.000114679	sarcosine oxidase, subunit gamma [EC:1.5.3.1]
K00311	0.000219766	0.000217276	0.000212207	0.000216624	electron-transferring-flavoprotein dehydrogenase [EC:1.5.5.1]
K00313	5.71833E-07	6.05032E-07	6.13118E-06	1.82881E-06	electron transfer flavoprotein-quinone oxidoreductase [EC:1.5.5.-]
K00314	0	7.63099E-08	6.86086E-08	1.90174E-09	sarcosine dehydrogenase [EC:1.5.8.3]
K00315	9.22781E-08	1.54097E-07	1.07511E-06	3.72818E-07	dimethylglycine dehydrogenase [EC:1.5.8.4]
K00316	3.2742E-07	9.03592E-07	3.0673E-06	9.48874E-06	spermidine dehydrogenase [EC:1.5.99.6]
K00317	0.000216299	0.000212621	0.000189111	0.000115482	dimethylamine/trimethylamine dehydrogenase [EC:1.5.8.1 1.5.8.2]
K00318	1.77251E-06	2.10533E-06	1.14135E-05	3.8071E-06	proline dehydrogenase [EC:1.5.-.-]
K00320	2.2172E-08	4.59298E-08	1.84047E-07	4.0059E-06	5,10-methylenetetrahydromethanopterin reductase [EC:1.5.98.2]
K00322	3.95709E-06	4.96084E-06	2.02502E-05	0.000131744	NAD(P) transhydrogenase [EC:1.6.1.1]
K00324	0.00086895	0.000861948	0.000799072	0.0006477	NAD(P) transhydrogenase subunit alpha [EC:1.6.1.2]
K00325	0.000433777	0.000429923	0.000399955	0.00032895	NAD(P) transhydrogenase subunit beta [EC:1.6.1.2]
K00329	0.000216233	0.000212619	0.000191494	0.000122585	NADH dehydrogenase [EC:1.6.5.3]
K00330	0.000220397	0.000219738	0.000218492	0.000206175	NADH-quinone oxidoreductase subunit A [EC:1.6.5.3]
K00331	0.000220917	0.000220133	0.00022474	0.000208236	NADH-quinone oxidoreductase subunit B [EC:1.6.5.3]
K00332	0.000219403	0.000218599	0.000220133	0.000203009	NADH-quinone oxidoreductase subunit C [EC:1.6.5.3]
K00333	0.00022057	0.000219476	0.000223787	0.000208232	NADH-quinone oxidoreductase subunit D [EC:1.6.5.3]
K00334	0.000220495	0.000219453	0.000216891	0.000206692	NADH-quinone oxidoreductase subunit E [EC:1.6.5.3]
K00335	0.000224111	0.000222148	0.000230619	0.000236658	NADH-quinone oxidoreductase subunit F [EC:1.6.5.3]
K00336	0.000221273	0.000220134	0.000218372	0.000206843	NADH-quinone oxidoreductase subunit G [EC:1.6.5.3]
K00337	0.000220364	0.000219754	0.000219012	0.000207091	NADH-quinone oxidoreductase subunit H [EC:1.6.5.3]
K00338	0.000221186	0.000220167	0.00022453	0.000206103	NADH-quinone oxidoreductase subunit I [EC:1.6.5.3]
K00339	0.000220359	0.000219735	0.000218876	0.000206078	NADH-quinone oxidoreductase subunit J [EC:1.6.5.3]
K00340	0.000220359	0.000219738	0.000218287	0.000206049	NADH-quinone oxidoreductase subunit K [EC:1.6.5.3]
K00341	0.00022333	0.000223683	0.000224072	0.000208588	NADH-quinone oxidoreductase subunit L [EC:1.6.5.3]
K00342	0.000220414	0.000219821	0.000219977	0.000208122	NADH-quinone oxidoreductase subunit M [EC:1.6.5.3]
K00343	0.000220442	0.000219923	0.000220443	0.000206365	NADH-quinone oxidoreductase subunit N [EC:1.6.5.3]
K00344	0.000904413	0.000914367	0.00101176	0.001148004	NADPH2:quinone reductase [EC:1.6.5.5]
K00346	4.00972E-07	1.44466E-06	4.02114E-06	1.8836E-05	Na+-transporting NADH:ubiquinone oxidoreductase subunit A [EC:1.6.5.8]
K00347	4.95602E-07	1.47698E-06	4.28535E-06	1.92775E-05	Na+-transporting NADH:ubiquinone oxidoreductase subunit B [EC:1.6.5.8]
K00348	8.29886E-07	2.07043E-06	6.25032E-06	1.89133E-05	Na+-transporting NADH:ubiquinone oxidoreductase subunit C [EC:1.6.5.8]
K00349	6.16749E-07	1.81174E-06	4.51279E-06	1.88617E-05	Na+-transporting NADH:ubiquinone oxidoreductase subunit D [EC:1.6.5.8]
K00350	9.35946E-07	1.69222E-06	5.02655E-06	2.19771E-05	Na+-transporting NADH:ubiquinone oxidoreductase subunit E [EC:1.6.5.8]
K00351	5.51982E-07	1.51556E-06	4.85718E-06	2.27014E-05	Na+-transporting NADH:ubiquinone oxidoreductase subunit F [EC:1.6.5.8]
K00354	0.00043687	0.000432344	0.00039154	0.000337185	NADPH2 dehydrogenase [EC:1.6.99.1]
K00355	0.000218193	0.000219785	0.000213708	0.000269653	NAD(P)H dehydrogenase (quinone) [EC:1.6.5.2]
K00356	0.000216233	0.000212619	0.000191494	0.000122585	NADH dehydrogenase [EC:1.6.99.3]
K00360	2.39245E-06	3.9518E-06	1.71795E-05	4.24852E-05	assimilatory nitrate reductase electron transfer subunit [EC:1.7.99.-]
K00362	0.000439218	0.000433558	0.00042078	0.000335187	nitrite reductase (NADH) large subunit [EC:1.7.1.15]
K00363	0.000221072	0.000220085	0.000224553	0.000204672	nitrite reductase (NADH) small subunit [EC:1.7.1.15]
K00364	3.68583E-06	6.55076E-06	2.14947E-05	5.18953E-05	GMP reductase [EC:1.7.1.7]
K00365	1.03021E-06	8.01493E-09	4.08248E-07	3.60974E-06	urate oxidase [EC:1.7.3.3]
K00366	1.05197E-06	3.11104E-07	6.21521E-06	2.22777E-07	ferredoxin-nitrite reductase [EC:1.7.7.1]
K00367	3.52848E-06	3.97417E-06	1.94501E-05	4.97919E-05	ferredoxin-nitrate reductase [EC:1.7.7.2]
K00368	0.000429273	0.000425844	0.000382109	0.000232804	nitrite reductase (NO-forming) [EC:1.7.2.1]
K00370	0.000218962	0.00021587	0.00021324	0.000183199	nitrate reductase / nitrite oxidoreductase, alpha subunit [EC:1.7.5.1 1.7.99.-]
K00371	0.000218852	0.000216457	0.000213283	0.000183088	nitrate reductase / nitrite oxidoreductase, beta subunit [EC:1.7.5.1 1.7.99.-]
K00372	0.000433015	0.000428014	0.000397804	0.000280579	assimilatory nitrate reductase catalytic subunit [EC:1.7.99.-]

K00373	0.000218839	0.000215809	0.00021319	0.000183155	nitrate reductase molybdenum cofactor assembly chaperone NarJ/NarW
K00374	0.000218936	0.000215809	0.000212987	0.000183055	nitrate reductase gamma subunit [EC:1.7.5.1 1.7.99.-]
K00375	0.001095464	0.001107073	0.001138764	0.00112612	GntR family transcriptional regulator / MocR family aminotransferase
K00376	0.0002141	0.000212308	0.00018704	0.000114375	nitrous-oxide reductase [EC:1.7.2.4]
K00380	1.23384E-05	1.02729E-05	4.25933E-05	0.000244696	sulfite reductase (NADPH) flavoprotein alpha-component [EC:1.8.1.2]
K00381	0.000433132	0.000428081	0.000397076	0.000279804	sulfite reductase (NADPH) hemoprotein beta-component [EC:1.8.1.2]
K00382	0.001108009	0.001106004	0.001086243	0.001067669	dihydrolipoamide dehydrogenase [EC:1.8.1.4]
K00383	0.000440304	0.000438765	0.000433027	0.000414937	glutathione reductase (NADPH) [EC:1.8.1.7]
K00384	0.000671308	0.000675498	0.000713841	0.0007055	thioredoxin reductase (NADPH) [EC:1.8.1.9]
K00385	1.18384E-07	1.46934E-07	4.10681E-07	8.96862E-08	anaerobic sulfite reductase subunit C
K00387	2.1928E-06	5.11502E-08	2.55371E-07	4.75661E-08	sulfite oxidase [EC:1.8.3.1]
K00389	1.59856E-06	3.83999E-06	3.21226E-06	8.27529E-06	putative membrane protein
K00390	0.000221454	0.000221919	0.0002168	0.000176362	phosphoadenosine phosphosulfate reductase [EC:1.8.4.8 1.8.4.10]
K00392	1.73947E-06	3.78889E-06	3.03454E-06	3.54131E-06	sulfite reductase (ferredoxin) [EC:1.8.7.1]
K00394	0	1.26847E-07	1.43658E-07	2.26312E-08	adenylylsulfate reductase, subunit A [EC:1.8.99.2]
K00395	0	1.26847E-07	5.57805E-08	2.26312E-08	adenylylsulfate reductase, subunit B [EC:1.8.99.2]
K00400	1.63519E-06	4.85719E-07	1.54032E-06	2.76305E-06	methyl coenzyme M reductase system, component A2
K00404	0.000431238	0.00042966	0.000394174	0.000278214	cytochrome c oxidase cbb3-type subunit I [EC:1.9.3.1]
K00405	0.000431262	0.000429219	0.000393281	0.000278635	cytochrome c oxidase cbb3-type subunit II
K00406	0.000435544	0.000432133	0.000407928	0.000380249	cytochrome c oxidase cbb3-type subunit III
K00407	0.000217242	0.000216092	0.00020721	0.00016477	cytochrome c oxidase cbb3-type subunit IV
K00410	3.0448E-06	1.96183E-07	8.19183E-07	2.71984E-07	ubiquinol-cytochrome c reductase cytochrome b/c1 subunit
K00411	0.00021935	0.000216887	0.000207616	0.000216484	ubiquinol-cytochrome c reductase iron-sulfur subunit [EC:1.10.2.2]
K00412	0.000219301	0.000216833	0.000208244	0.000217631	ubiquinol-cytochrome c reductase cytochrome b subunit
K00413	0.000219245	0.000216819	0.000207479	0.000216362	ubiquinol-cytochrome c reductase cytochrome c1 subunit
K00423	0.000213824	0.000211775	0.000187148	0.00011327	L-ascorbate oxidase [EC:1.10.3.3]
K00424	4.11007E-06	4.24006E-06	1.78681E-05	0.000107457	cytochrome bd-I ubiquinol oxidase subunit X [EC:1.10.3.14]
K00425	0.0004399	0.000435122	0.000411032	0.000408154	cytochrome bd ubiquinol oxidase subunit I [EC:1.10.3.14]
K00426	0.000439882	0.000435069	0.000410859	0.00040784	cytochrome bd ubiquinol oxidase subunit II [EC:1.10.3.14]
K00427	4.84303E-06	6.30221E-06	1.77888E-05	8.53651E-05	L-lactate permease
K00428	0.000429404	0.000430005	0.00038825	0.000277326	cytochrome c peroxidase [EC:1.11.1.5]
K00432	0.000439715	0.000433815	0.00041221	0.000443391	glutathione peroxidase [EC:1.11.1.9]
K00433	8.465E-06	7.53719E-06	1.18425E-05	0.000186658	non-heme chloroperoxidase [EC:1.11.1.10]
K00435	1.1026E-06	2.06813E-07	3.36432E-06	3.22644E-08	Fe-coproporphyrin III decarboxylase [EC:1.11.1.-]
K00436	4.55614E-08	6.7355E-08	2.4568E-07	4.90346E-07	NAD-reducing hydrogenase large subunit [EC:1.12.1.2]
K00437	0	7.55344E-09	2.49688E-07	1.22309E-06	[NiFe] hydrogenase large subunit [EC:1.12.2.1]
K00440	0	0	4.31646E-08	3.80731E-07	coenzyme F420 hydrogenase subunit alpha [EC:1.12.98.1]
K00441	4.35189E-11	7.9632E-08	2.94208E-06	2.19129E-07	coenzyme F420 hydrogenase subunit beta [EC:1.12.98.1]
K00442	0	0	0	5.35811E-09	coenzyme F420 hydrogenase subunit delta
K00443	9.07505E-11	2.25716E-10	6.53532E-10	2.68823E-09	coenzyme F420 hydrogenase subunit gamma [EC:1.12.98.1]
K00446	1.91997E-06	4.5309E-06	6.22944E-06	3.46298E-05	catechol 2,3-dioxygenase [EC:1.13.11.2]
K00448	0.00021436	0.000212285	0.000188657	0.000140006	protocatechuate 3,4-dioxygenase, alpha subunit [EC:1.13.11.3]
K00449	0.000214567	0.000212466	0.000189877	0.000140325	protocatechuate 3,4-dioxygenase, beta subunit [EC:1.13.11.3]
K00450	0.000429493	0.000427757	0.000389599	0.000262084	gentisate 1,2-dioxygenase [EC:1.13.11.4]
K00451	0.000215564	0.000215104	0.000191655	0.000183063	homogentisate 1,2-dioxygenase [EC:1.13.11.5]
K00452	1.08508E-06	2.05284E-06	1.83639E-06	5.35129E-05	3-hydroxyanthranilate 3,4-dioxygenase [EC:1.13.11.6]
K00453	0.000431774	0.000432456	0.000405979	0.000351233	tryptophan 2,3-dioxygenase [EC:1.13.11.11]
K00455	7.22104E-08	1.60697E-07	4.99497E-07	9.66175E-07	3,4-dihydroxyphenylacetate 2,3-dioxygenase [EC:1.13.11.15]
K00456	0	8.78843E-10	2.76019E-09	5.28572E-10	cysteine dioxygenase [EC:1.13.11.20]
K00457	0.000432068	0.000433043	0.000409254	0.000371771	4-hydroxyphenylpyruvate dioxygenase [EC:1.13.11.27]
K00459	0.000867681	0.000866753	0.000811907	0.000644966	nitronate monooxygenase [EC:1.13.12.16]
K00461	0	0	0	8.03716E-09	arachidonate 5-lipoxygenase [EC:1.13.11.34]
K00462	1.12039E-08	2.69919E-10	3.7812E-07	2.36895E-07	biphenyl-2,3-diol 1,2-dioxygenase [EC:1.13.11.39]
K00463	0	0	4.33942E-08	0	indoleamine 2,3-dioxygenase [EC:1.13.11.52]
K00464	0.000214244	0.000211967	0.00018682	0.000132402	all-trans-8-apo-beta-carotenal 15,15-oxygenase [EC:1.13.11.75]
K00466	1.34494E-07	7.98605E-07	6.94265E-07	2.61549E-06	tryptophan 2-monooxygenase [EC:1.13.12.3]
K00467	3.52957E-06	6.93997E-06	3.06009E-05	7.03813E-05	lactate 2-monooxygenase [EC:1.13.12.4]
K00469	4.23211E-08	2.55502E-09	6.23767E-08	1.53121E-08	inositol oxygenase [EC:1.13.99.1]
K00471	1.11031E-07	1.66283E-07	9.81863E-07	1.95266E-07	gamma-butyrobetaine dioxygenase [EC:1.14.11.1]
K00472	0.000215994	0.000215508	0.000203993	0.000154104	prolyl 4-hydroxylase [EC:1.14.11.2]
K00476	1.05589E-07	3.23107E-07	1.82795E-07	7.81874E-08	aspartate beta-hydroxylase [EC:1.14.11.16]
K00477	0.000213843	0.000211898	0.000186392	0.000114018	phytanoyl-CoA hydroxylase [EC:1.14.11.18]
K00479	7.9612E-07	6.94822E-07	3.19926E-06	2.794E-06	glycine betaine catabolism A
K00480	0.000220416	0.000222388	0.000228415	0.000231559	salicylate hydroxylase [EC:1.14.13.1]
K00481	0.000215884	0.000216078	0.000205292	0.000161573	p-hydroxybenzoate 3-monooxygenase [EC:1.14.13.2]
K00483	1.42558E-06	3.39413E-06	1.48781E-05	3.72541E-05	4-hydroxyphenylacetate 3-monooxygenase [EC:1.14.14.9]
K00484	2.86093E-06	4.8025E-07	1.94367E-06	3.69981E-06	flavin reductase (NADH) [EC:1.5.1.36]
K00485	0	0	5.76604E-08	2.67905E-09	dimethylaniline monooxygenase (N-oxide forming) [EC:1.14.13.8]
K00486	2.08919E-06	1.97882E-06	2.27196E-06	5.21017E-05	kynurenine 3-monooxygenase [EC:1.14.13.9]
K00491	1.02754E-06	2.06813E-07	3.57964E-06	1.71681E-06	nitric-oxide synthase, bacterial [EC:1.14.14.47]
K00493	0.000216416	0.000214761	0.000199148	0.000143404	unspecific monooxygenase [EC:1.14.14.1]
K00494	3.1635E-09	0	9.89572E-09	1.58716E-07	alkanal monooxygenase alpha chain [EC:1.14.14.3]
K00496	3.23058E-08	1.10729E-06	1.80888E-06	1.15934E-05	alkane 1-monooxygenase [EC:1.14.15.3]
K00499	0.000429021	0.000426765	0.000389808	0.00026191	choline monooxygenase [EC:1.14.15.7]
K00500	0.000216512	0.000217388	0.000205957	0.000215676	phenylalanine-4-hydroxylase [EC:1.14.16.1]
K00504	2.84943E-08	0	8.93407E-08	0	peptidylglycine monooxygenase [EC:1.14.17.3]
K00505	0.000428489	0.000425562	0.000381846	0.000259196	tyrosinase [EC:1.14.18.1]
K00507	0.000432615	0.000428714	0.000396401	0.000381446	stearoyl-CoA desaturase (Delta-9 desaturase) [EC:1.14.19.1]
K00508	1.11909E-06	2.33304E-06	4.06484E-06	6.24498E-05	linoleoyl-CoA desaturase [EC:1.14.19.3]
K00514	8.99101E-09	0	0	5.90766E-09	zeta-carotene desaturase [EC:1.3.5.6]
K00518	8.14197E-08	3.43967E-07	4.34098E-07	4.72035E-07	nickel superoxide dismutase [EC:1.15.1.1]
K00520	0.00022315	0.000222405	0.000236515	0.000195448	mercuric reductase [EC:1.16.1.1]
K00523	0.000217056	0.000219438	0.000222189	0.000197403	CDP-4-dehydro-6-deoxyglucose reductase, E3 [EC:1.17.1.1]
K00524	2.997E-09	0	1.91022E-07	6.78334E-09	ribonucleotide reductase, class II [EC:1.17.4.1]
K00525	0.000227897	0.000231975	0.000256449	0.000319864	ribonucleoside-diphosphate reductase alpha chain [EC:1.17.4.1]
K00526	0.000222605	0.000223677	0.00022708	0.000280347	ribonucleoside-diphosphate reductase beta chain [EC:1.17.4.1]
K00527	9.26252E-08	0	9.37395E-08	0	ribonucleoside-triphosphate reductase (thioredoxin) [EC:1.17.4.2]

K00528	0.000224742	0.000227201	0.000224744	0.000225423	ferredoxin/flavodoxin---NADP+ reductase [EC:1.18.1.2 1.19.1.1]
K00529	1.51736E-05	1.56263E-05	4.93689E-05	0.000107584	3-phenylpropionate/trans-cinnamate dioxygenase ferredoxin reductase component [EC:1.18.1.3]
K00531	2.04795E-08	2.16501E-08	1.41125E-08	0	nitrogenase delta subunit [EC:1.18.6.1]
K00532	4.19363E-07	1.91004E-07	6.89888E-07	1.14294E-07	ferredoxin hydrogenase [EC:1.12.7.2]
K00533	5.79574E-07	7.03753E-07	3.94915E-06	3.86482E-08	ferredoxin hydrogenase large subunit [EC:1.12.7.2]
K00534	2.01241E-07	2.49256E-08	8.86124E-08	1.15964E-07	ferredoxin hydrogenase small subunit [EC:1.12.7.2]
K00537	0.000443305	0.000441519	0.000440666	0.000522908	arsenate reductase [EC:1.20.4.1]
K00542	3.24676E-08	6.5978E-09	5.76604E-08	0	guanidinoacetate N-methyltransferase [EC:2.1.1.2]
K00544	0	0	8.30004E-08	3.36582E-08	betaine-homocysteine S-methyltransferase [EC:2.1.1.5]
K00545	0	1.06439E-08	3.70296E-08	2.14324E-08	catechol O-methyltransferase [EC:2.1.1.6]
K00547	2.41126E-06	1.26975E-06	8.83705E-06	1.91049E-05	homocysteine S-methyltransferase [EC:2.1.1.10]
K00548	0.000438344	0.000438565	0.00042641	0.000428001	5-methyltetrahydrofolate--homocysteine methyltransferase [EC:2.1.1.13]
K00549	0.000222138	0.000221683	0.000218283	0.000234222	5-methyltetrahydropteroyltriglutamate--homocysteine methyltransferase [EC:2.1.1.14]
K00554	0.000223471	0.000223749	0.000228248	0.000227254	tRNA (guanine37-N1)-methyltransferase [EC:2.1.1.228]
K00555	0	3.27506E-09	2.59514E-08	0	tRNA (guanine26-N2/guanine27-N2)-dimethyltransferase [EC:2.1.1.215 2.1.1.216]
K00556	1.53445E-07	1.41263E-06	1.35849E-06	1.19064E-05	tRNA (guanosine-2-O-)-methyltransferase [EC:2.1.1.34]
K00557	6.15632E-07	1.07071E-06	4.4666E-06	2.21157E-05	tRNA (uracil-5-)-methyltransferase [EC:2.1.1.35]
K00558	0.000441304	0.000439009	0.000430207	0.000361751	DNA (cytosine-5)-methyltransferase 1 [EC:2.1.1.37]
K00560	0.000223149	0.000223617	0.0002281	0.000236902	thymidylate synthase [EC:2.1.1.45]
K00561	2.96277E-08	2.95183E-07	1.84948E-06	4.26375E-07	23S rRNA (adenine-N6)-dimethyltransferase [EC:2.1.1.184]
K00563	3.09042E-06	2.05739E-06	1.53583E-05	2.44091E-05	23S rRNA (guanine745-N1)-methyltransferase [EC:2.1.1.187]
K00564	6.85185E-06	7.46927E-06	3.08403E-05	8.73555E-05	16S rRNA (guanine1207-N2)-methyltransferase [EC:2.1.1.172]
K00566	0.000225382	0.000225716	0.000231787	0.000225502	tRNA-uridine 2-sulfurtransferase [EC:2.8.1.13]
K00567	0.00065339	0.000653072	0.000619389	0.000527265	methylated-DNA-[protein]-cysteine S-methyltransferase [EC:2.1.1.63]
K00568	0.000437875	0.000430813	0.000401894	0.00039182	2-polyprenyl-6-hydroxyphenyl methylase / 3-demethylubiquinone-9 3-methyltransferase [EC:2.1.1.222 2.1.1.64]
K00569	1.38133E-06	9.16959E-07	2.1977E-06	1.3523E-05	thiopurine S-methyltransferase [EC:2.1.1.67]
K00570	4.1501E-06	2.40976E-07	8.22258E-07	8.91401E-07	phosphatidylethanolamine/phosphatidyl-N-methylethanolamine N-methyltransferase [EC:2.1.1.17 2.1.1.71]
K00571	0.000865435	0.000851936	0.000767881	0.000505496	site-specific DNA-methyltransferase (adenine-specific) [EC:2.1.1.72]
K00573	0.000439255	0.000435828	0.000418171	0.000467385	protein-L-isoaspartate(D-aspartate) O-methyltransferase [EC:2.1.1.77]
K00574	0.000440236	0.000441586	0.000447206	0.000587141	cyclopropane-fatty-acyl-phospholipid synthase [EC:2.1.1.79]
K00575	0.000439848	0.000434935	0.000421444	0.000468927	chemotaxis protein methyltransferase CheR [EC:2.1.1.80]
K00577	0	0	0	5.35811E-09	tetrahydromethanopterin S-methyltransferase subunit A [EC:2.1.1.86]
K00587	2.997E-09	0	1.36678E-07	1.35667E-08	protein-S-isoprenylcysteine O-methyltransferase [EC:2.1.1.100]
K00588	1.1216E-06	2.95834E-06	6.43352E-06	4.63087E-05	caffeoyl-CoA O-methyltransferase [EC:2.1.1.104]
K00590	6.97875E-07	8.55696E-07	3.48465E-06	7.10325E-06	site-specific DNA-methyltransferase (cytosine-N4-specific) [EC:2.1.1.113]
K00594	1.80721E-07	5.4256E-07	9.15454E-07	3.95958E-06	alditol oxidase [EC:1.1.3.41]
K00595	4.23818E-06	4.69611E-06	3.11853E-05	3.75874E-05	precorrin-6Y C5,15-methyltransferase (decarboxylating) [EC:2.1.1.132]
K00596	1.0702E-06	3.05844E-07	1.13858E-06	9.10913E-08	2,2-dialkylglycine decarboxylase (pyruvate) [EC:4.1.1.64]
K00598	5.90629E-06	1.063E-05	3.68674E-05	0.000127265	trans-aconitate 2-methyltransferase [EC:2.1.1.144]
K00600	0.000226677	0.000229779	0.000259193	0.000306256	glycine hydroxymethyltransferase [EC:2.1.2.1]
K00602	0.000223876	0.000224525	0.000230915	0.000225697	phosphoribosylaminoimidazolecarboxamide formyltransferase / IMP cyclohydrolase [EC:2.1.2.3 3.5.4.10]
K00603	1.04231E-06	7.60689E-07	1.82425E-06	1.97435E-06	glutamate formiminotransferase [EC:2.1.2.5]
K00604	0.000439768	0.000435757	0.000416433	0.000344137	methionyl-tRNA formyltransferase [EC:2.1.2.9]
K00605	0.000222185	0.000223389	0.000225188	0.000225553	aminomethyltransferase [EC:2.1.2.10]
K00606	0.000222527	0.000222651	0.00021782	0.000225608	3-methyl-2-oxobutanoate hydroxymethyltransferase [EC:2.1.2.11]
K00609	0.000224192	0.000224142	0.00023164	0.000234513	aspartate carbamoyltransferase catalytic subunit [EC:2.1.3.2]
K00610	1.40437E-06	1.54449E-06	3.09242E-06	2.07002E-05	aspartate carbamoyltransferase regulatory subunit
K00611	0.000223844	0.000222297	0.000229504	0.000186535	ornithine carbamoyltransferase [EC:2.1.3.3]
K00612	0.000215071	0.000212685	0.000190086	0.000114541	carbamoyltransferase [EC:2.1.3.-]
K00613	1.48887E-06	2.70844E-07	5.63164E-06	1.08998E-07	glycine amidinotransferase [EC:2.1.4.1]
K00615	0.0002329	0.000234805	0.000278266	0.000306521	transketolase [EC:2.2.1.1]
K00616	0.000223339	0.000224091	0.000224967	0.000227261	transaldolase [EC:2.2.1.2]
K00619	0.000215221	0.000215226	0.000190818	0.000120799	amino-acid N-acetyltransferase [EC:2.3.1.1]
K00620	0.000222034	0.000220632	0.000222072	0.000153606	glutamate N-acetyltransferase / amino-acid N-acetyltransferase [EC:2.3.1.35 2.3.1.1]
K00622	0	1.23941E-07	7.31057E-08	2.82158E-08	arylamine N-acetyltransferase [EC:2.3.1.5]
K00624	0	0	3.1232E-07	1.14104E-08	carnitine O-acetyltransferase [EC:2.3.1.7]
K00625	1.23014E-05	1.31086E-05	4.6185E-05	8.13805E-05	phosphate acetyltransferase [EC:2.3.1.8]
K00626	0.001114267	0.001128541	0.001176135	0.00133191	acetyl-CoA C-acetyltransferase [EC:2.3.1.9]
K00627	0.000667636	0.000666835	0.000650774	0.000734969	pyruvate dehydrogenase E2 component (dihydrolipoamide acetyltransferase) [EC:2.3.1.12]
K00630	0	0	0	2.67905E-09	glycerol-3-phosphate O-acyltransferase [EC:2.3.1.15]
K00631	2.4329E-06	1.7656E-06	4.13915E-06	7.19413E-05	glycerol-3-phosphate O-acyltransferase [EC:2.3.1.15]
K00632	0.000885631	0.000900428	0.000926321	0.00101119	acetyl-CoA acyltransferase [EC:2.3.1.16]
K00633	1.29632E-06	1.7866E-06	9.18337E-06	5.90491E-06	galactoside O-acetyltransferase [EC:2.3.1.18]
K00634	5.19717E-06	7.00468E-06	3.0216E-05	7.00299E-05	phosphate butyryltransferase [EC:2.3.1.19]
K00635	1.1922E-06	1.19889E-06	2.80023E-06	8.60956E-06	diacylglycerol O-acyltransferase [EC:2.3.1.20]
K00638	0.000218691	0.000216189	0.000202656	0.00015311	chloramphenicol O-acetyltransferase type B [EC:2.3.1.28]
K00639	0.000434329	0.000430688	0.00038665	0.000296782	glycine C-acetyltransferase [EC:2.3.1.29]
K00640	0.000445536	0.000443282	0.000452553	0.000369651	serine O-acetyltransferase [EC:2.3.1.30]
K00641	0.000441039	0.000434281	0.000407559	0.000371874	homoserine O-acetyltransferase/O-succinyltransferase [EC:2.3.1.31 2.3.1.46]
K00643	3.55589E-06	1.29464E-06	7.51396E-06	3.7067E-06	5-aminolevulinate synthase [EC:2.3.1.37]
K00645	0.000226998	0.000227272	0.000239613	0.000309755	[acyl-carrier-protein] S-malonyltransferase [EC:2.3.1.39]
K00646	3.54886E-08	1.65105E-08	2.20336E-07	1.97621E-08	malonyl-ACP decarboxylase
K00647	0.000655691	0.000651268	0.00062469	0.000655089	3-oxoacyl-[acyl-carrier-protein] synthase I [EC:2.3.1.41]
K00648	0.00022815	0.000233196	0.000257007	0.000343477	3-oxoacyl-[acyl-carrier-protein] synthase III [EC:2.3.1.180]
K00651	1.51462E-06	1.98062E-06	6.64724E-06	2.07408E-05	homoserine O-succinyltransferase/O-acetyltransferase [EC:2.3.1.46 2.3.1.31]
K00652	0.000223614	0.000222518	0.000228238	0.00022495	8-amino-7-oxononanoate synthase [EC:2.3.1.47]
K00655	0.000662189	0.000664871	0.000672395	0.000681021	1-acyl-sn-glycerol-3-phosphate acyltransferase [EC:2.3.1.51]
K00656	0.000217615	0.000217112	0.00020822	0.000158519	formate C-acetyltransferase [EC:2.3.1.54]
K00657	0.000434642	0.000434207	0.000412861	0.000386665	diamine N-acetyltransferase [EC:2.3.1.57]
K00658	0.000440502	0.000443276	0.000413169	0.000341838	2-oxoglutarate dehydrogenase E2 component (dihydrolipoamide succinyltransferase) [EC:2.3.1.61]
K00661	0.000441052	0.000441468	0.000435051	0.000332044	maltose O-acetyltransferase [EC:2.3.1.79]
K00662	2.43036E-07	1.51659E-07	3.93438E-06	1.18515E-06	aminoglycoside 3-N-acetyltransferase [EC:2.3.1.81]
K00663	8.2559E-08	6.99207E-08	1.0302E-06	7.63186E-06	aminoglycoside 6-N-acetyltransferase [EC:2.3.1.82]
K00666	0.00069389	0.000723541	0.000928903	0.00129753	fatty-acyl-CoA synthase [EC:6.2.1.-]
K00672	9.87446E-07	4.21631E-08	6.3322E-08	1.33207E-07	formylmethanofuran--tetrahydromethanopterin N-formyltransferase [EC:2.3.1.101]
K00673	1.38163E-06	4.69058E-07	3.73463E-06	2.74156E-05	arginine N-succinyltransferase [EC:2.3.1.109]
K00674	0.000222673	0.000222655	0.000225843	0.000228582	2,3,4,5-tetrahydropyridine-2,6-dicarboxylate N-succinyltransferase [EC:2.3.1.117]

K00675	3.10982E-06	3.94051E-06	2.02388E-05	8.95802E-05	N-hydroxyarylamine O-acetyltransferase [EC:2.3.1.118]
K00677	0.000437623	0.000433133	0.000404038	0.000342633	UDP-N-acetylglucosamine acyltransferase [EC:2.3.1.129]
K00680	9.37358E-07	2.33954E-09	2.69236E-06	4.06038E-08	uncharacterized N-acetyltransferase [EC:2.3.1.-]
K00681	0.000659976	0.000656309	0.00065282	0.000642835	gamma-glutamyltranspeptidase / glutathione hydrolase [EC:2.3.2.2 3.4.19.13]
K00684	0.000220198	0.000219238	0.000214135	0.000217869	leucyl/phenylalanyl-tRNA---protein transferase [EC:2.3.2.6]
K00686	5.54378E-08	9.5208E-10	7.95394E-08	0	protein-glutamine gamma-glutamyltransferase [EC:2.3.2.13]
K00687	7.94662E-07	3.59561E-07	6.75321E-06	3.62269E-06	penicillin-binding protein 2B
K00688	5.46572E-06	7.99618E-06	3.59308E-05	5.87842E-05	glycogen phosphorylase [EC:2.4.1.1]
K00689	1.33512E-07	1.78156E-08	4.62822E-07	3.68091E-07	dextransucrase [EC:2.4.1.5]
K00690	1.51212E-07	6.43842E-08	3.82351E-06	6.92438E-06	sucrose phosphorylase [EC:2.4.1.7]
K00691	1.02421E-06	3.59616E-06	9.59985E-07	8.97467E-07	maltose phosphorylase [EC:2.4.1.8]
K00692	9.90998E-08	1.72247E-08	4.20131E-07	4.68638E-08	levansucrase [EC:2.4.1.10]
K00693	2.43383E-07	7.03627E-07	2.586E-06	2.18841E-08	glycogen synthase [EC:2.4.1.11]
K00694	1.818E-06	1.72918E-06	5.44654E-06	2.75492E-05	cellulose synthase (UDP-forming) [EC:2.4.1.12]
K00695	0	3.98157E-08	2.09314E-08	0	sucrose synthase [EC:2.4.1.13]
K00696	2.05141E-06	7.53435E-07	4.7607E-07	1.26788E-07	sucrose-phosphate synthase [EC:2.4.1.14]
K00697	0.000434229	0.000431271	0.000404383	0.000332811	trehalose 6-phosphate synthase [EC:2.4.1.15 2.4.1.347]
K00698	0	0	2.442E-08	0	chitin synthase [EC:2.4.1.16]
K00700	0.000220834	0.000223972	0.000217076	0.000186171	1,4-alpha-glucan branching enzyme [EC:2.4.1.18]
K00701	1.01823E-06	5.78456E-07	1.68718E-06	5.17273E-05	cyclomaltodextrin glucanotransferase [EC:2.4.1.19]
K00702	5.62613E-07	2.97536E-07	1.69155E-07	9.21332E-06	cellobiose phosphorylase [EC:2.4.1.20]
K00703	0.000218105	0.000216109	0.000201364	0.000220422	starch synthase [EC:2.4.1.21]
K00705	0.000221346	0.000219553	0.000217357	0.000184079	4-alpha-glucanotransferase [EC:2.4.1.25]
K00709	0	2.46496E-07	1.73766E-06	3.06242E-08	histo-blood group ABO system transferase [EC:2.4.1.40 2.4.1.37]
K00712	5.42277E-06	1.32037E-06	1.62181E-05	3.78173E-06	poly(glycerol-phosphate) alpha-glucosyltransferase [EC:2.4.1.52]
K00713	6.21936E-09	7.59288E-09	1.52812E-08	1.12869E-07	UDP-glucose:(glucosyl)LPS alpha-1,2-glucosyltransferase [EC:2.4.1.-]
K00720	2.29558E-06	3.2963E-07	1.18613E-06	1.98804E-05	ceramide glucosyltransferase [EC:2.4.1.80]
K00721	9.42846E-06	1.06743E-05	2.35522E-05	8.72313E-05	dolichol-phosphate mannosyltransferase [EC:2.4.1.83]
K00728	1.53547E-06	4.14951E-06	1.01258E-05	4.07859E-06	dolichyl-phosphate-mannose-protein mannosyltransferase [EC:2.4.1.109]
K00737	4.0923E-10	2.85232E-09	1.25361E-08	2.00306E-08	beta-1,4-mannosyl-glycoprotein beta-1,4-N-acetylglucosaminyltransferase [EC:2.4.1.144]
K00748	0.000219732	0.000218764	0.000210961	0.000217739	lipid-A-disaccharide synthase [EC:2.4.1.182]
K00752	9.71301E-08	2.95742E-08	5.27837E-07	6.02613E-07	hyaluronan synthase [EC:2.4.1.212]
K00754	0.000218798	0.000216947	0.000194484	0.000153909	L-malate glycosyltransferase [EC:2.4.1.-]
K00756	4.77738E-06	4.59463E-06	1.27111E-05	2.14159E-05	pyrimidine-nucleoside phosphorylase [EC:2.4.2.2]
K00757	1.26133E-06	2.0281E-06	7.0312E-06	1.97904E-05	uridine phosphorylase [EC:2.4.2.3]
K00758	5.20507E-06	5.21754E-06	1.29774E-05	2.84634E-05	thymidine phosphorylase [EC:2.4.2.4]
K00759	0.000437699	0.000436354	0.000421632	0.000345596	adenine phosphoribosyltransferase [EC:2.4.2.7]
K00760	0.000222205	0.000221002	0.000211645	0.000191961	hypoxanthine phosphoribosyltransferase [EC:2.4.2.8]
K00761	0.000222766	0.000224026	0.000227993	0.000227482	uracil phosphoribosyltransferase [EC:2.4.2.9]
K00762	0.000224839	0.000225673	0.000234954	0.00028456	orotate phosphoribosyltransferase [EC:2.4.2.10]
K00763	0.000224222	0.000225029	0.000243138	0.000249273	nicotinate phosphoribosyltransferase [EC:6.3.4.21]
K00764	0.000223846	0.000224993	0.000236188	0.00023151	amidophosphoribosyltransferase [EC:2.4.2.14]
K00765	0.000223014	0.000222887	0.000226762	0.000221657	ATP phosphoribosyltransferase [EC:2.4.2.17]
K00766	0.000651568	0.000649695	0.000609143	0.000489906	anthranilate phosphoribosyltransferase [EC:2.4.2.18]
K00767	0.000221583	0.000224123	0.000220768	0.000234169	nicotinate-nucleotide pyrophosphorylase (carboxylating) [EC:2.4.2.19]
K00768	0.00021849	0.000217982	0.000223708	0.000164982	nicotinate-nucleotide--dimethylbenzimidazole phosphoribosyltransferase [EC:2.4.2.21]
K00769	4.33615E-07	4.62978E-07	6.54972E-07	1.27025E-05	xanthine phosphoribosyltransferase [EC:2.4.2.22]
K00771	0	4.42717E-08	1.70693E-07	3.39174E-06	protein xylosyltransferase [EC:2.4.2.26]
K00772	1.62617E-06	1.48231E-06	2.11669E-06	5.97312E-07	5-methylthioadenosine phosphorylase [EC:2.4.2.28]
K00773	0.000223519	0.000223492	0.00022709	0.000245732	queuine tRNA-ribosyltransferase [EC:2.4.2.29]
K00776	0	0	0	2.23002E-08	NAD-diphthamide ADP-ribosyltransferase [EC:2.4.2.36]
K00782	0.000216878	0.000217354	0.000198041	0.000120386	L-lactate dehydrogenase complex protein LldG
K00783	0.000222867	0.000222598	0.000218547	0.000221449	23S rRNA (pseudouridine1915-N3)-methyltransferase [EC:2.1.1.177]
K00784	0.000217239	0.000215475	0.000197172	0.000127857	ribonuclease Z [EC:3.1.26.11]
K00785	1.13869E-06	8.54442E-09	6.88115E-07	3.66938E-08	beta-galactosamide-alpha-2,3-sialyltransferase [EC:2.4.99.-]
K00786	0.000214047	0.000212445	0.000187521	0.000116337	glycosyltransferase [EC:2.4.-.-]
K00788	0.000442022	0.000437351	0.000423339	0.000339417	thiamine-phosphate pyrophosphorylase [EC:2.5.1.3]
K00789	0.000224353	0.000224323	0.000230736	0.000225604	S-adenosylmethionine synthetase [EC:2.5.1.6]
K00790	0.0002281	0.000231371	0.000248258	0.000283693	UDP-N-acetylglucosamine 1-carboxyvinyltransferase [EC:2.5.1.7]
K00791	0.000224134	0.000224676	0.000231883	0.0002286	tRNA dimethylallyltransferase [EC:2.5.1.75]
K00793	0.000223082	0.000223717	0.000224423	0.000233067	riboflavin synthase [EC:2.5.1.9]
K00794	0.000224349	0.000226938	0.000238408	0.000258543	6,7-dimethyl-8-ribityllumazine synthase [EC:2.5.1.78]
K00795	0.000221887	0.000218842	0.00021798	0.000221171	farnesyl diphosphate synthase [EC:2.5.1.1 2.5.1.10]
K00796	0.00022385	0.000227547	0.000227551	0.00022993	dihydropteroate synthase [EC:2.5.1.15]
K00797	0.000648145	0.000646277	0.000596393	0.000481071	spermidine synthase [EC:2.5.1.16]
K00798	0.000220762	0.000220208	0.000217942	0.000208073	cob(I)alamin adenosyltransferase [EC:2.5.1.17]
K00799	0.002836726	0.002814309	0.002696074	0.002596522	glutathione S-transferase [EC:2.5.1.18]
K00800	0.000228182	0.000228156	0.000235092	0.00027863	3-phosphoshikimate 1-carboxyvinyltransferase [EC:2.5.1.19]
K00801	2.7638E-07	3.33369E-07	3.36719E-07	3.50126E-08	farnesyl-diphosphate farnesyltransferase [EC:2.5.1.21]
K00803	5.10354E-06	4.45441E-06	2.35841E-05	7.98111E-05	alkyldihydroxyacetonephosphate synthase [EC:2.5.1.26]
K00805	5.14778E-06	5.83326E-06	2.34037E-05	6.98027E-06	heptaprenyl diphosphate synthase [EC:2.5.1.30]
K00806	0.00022451	0.000225284	0.000237813	0.000227714	undecaprenyl diphosphate synthase [EC:2.5.1.31]
K00808	1.30445E-06	1.93429E-07	4.22278E-07	3.09756E-07	homospermidine synthase [EC:2.5.1.44]
K00809	3.54067E-07	7.94864E-07	5.3464E-07	1.31507E-07	deoxyhypusine synthase [EC:2.5.1.46]
K00812	0.000875117	0.000872597	0.000807892	0.000631586	aspartate aminotransferase [EC:2.6.1.1]
K00813	0.000218147	0.000217259	0.000203805	0.000215809	aspartate aminotransferase [EC:2.6.1.1]
K00817	0.000659439	0.000660263	0.000660725	0.00058277	histidinol-phosphate aminotransferase [EC:2.6.1.9]
K00819	0.000860532	0.000856164	0.00078214	0.000554596	ornithine--oxo-acid transaminase [EC:2.6.1.13]
K00820	0.000226225	0.00022782	0.000246168	0.000311826	glucosamine---fructose-6-phosphate aminotransferase (isomerizing) [EC:2.6.1.16]
K00821	0.001090825	0.001091491	0.00105292	0.000847679	acetylornithine/N-succinyldiaminopimelate aminotransferase [EC:2.6.1.11 2.6.1.17]
K00822	0.000647207	0.000644351	0.000588034	0.000495703	beta-alanine--pyruvate transaminase [EC:2.6.1.18]
K00823	0.000439153	0.000440965	0.000438464	0.0004093	4-aminobutyrate aminotransferase [EC:2.6.1.19]
K00824	0.000216606	0.000212288	0.000191546	0.000113962	D-alanine transaminase [EC:2.6.1.21]
K00826	0.000225354	0.000224906	0.000231298	0.000234672	branched-chain amino acid aminotransferase [EC:2.6.1.42]
K00830	4.18375E-06	3.17616E-06	1.8191E-05	4.59002E-05	alanine-glyoxylate transaminase / serine-glyoxylate transaminase / serine-pyruvate transaminase [EC:2.6.1.44 2.6.1.45
K00831	0.000222123	0.000223205	0.000224848	0.000222626	phosphoserine aminotransferase [EC:2.6.1.52]
K00832	0.000220264	0.000221143	0.000225675	0.000268025	aromatic-amino-acid transaminase [EC:2.6.1.57]

K00833	0.000650378	0.000649012	0.0006133	0.000526566	adenosylmethionine---8-amino-7-oxononanoate aminotransferase [EC:2.6.1.62]
K00835	1.33637E-06	5.40113E-07	1.04497E-06	1.49456E-05	valine--pyruvate aminotransferase [EC:2.6.1.66]
K00836	0.000432068	0.000429614	0.0003838	0.000253129	diaminobutyrate-2-oxoglutarate transaminase [EC:2.6.1.76]
K00839	1.50761E-07	3.0206E-07	4.30847E-07	8.81952E-06	(S)-ureidoglycine---glyoxylate transaminase [EC:2.6.1.112]
K00840	0.000218858	0.000216462	0.000199852	0.000190445	succinylornithine aminotransferase [EC:2.6.1.81]
K00841	0.000219792	0.000217611	0.000196699	0.000118562	aminotransferase [EC:2.6.1.-]
K00842	0	0	1.78121E-07	8.41855E-09	aminotransferase [EC:2.6.1.-]
K00844	1.03837E-09	3.18986E-07	3.32623E-07	1.53089E-09	hexokinase [EC:2.7.1.1]
K00845	0.000442308	0.000437618	0.000428721	0.000429444	glucokinase [EC:2.7.1.2]
K00846	0	4.03924E-08	5.23286E-09	0	ketohexokinase [EC:2.7.1.3]
K00847	0.000227337	0.000227055	0.000236091	0.000315157	fructokinase [EC:2.7.1.4]
K00848	4.69752E-07	1.29741E-06	1.40642E-06	4.82151E-06	rhamnulokinase [EC:2.7.1.5]
K00849	2.89651E-06	3.20216E-06	2.24612E-05	2.5465E-05	galactokinase [EC:2.7.1.6]
K00850	4.57231E-06	8.03236E-06	2.11515E-05	2.19054E-05	6-phosphofructokinase 1 [EC:2.7.1.11]
K00851	0.000433311	0.000429707	0.000402958	0.000285268	gluconokinase [EC:2.7.1.12]
K00852	0.000223166	0.000219817	0.000224142	0.000252383	ribokinase [EC:2.7.1.15]
K00853	3.31087E-06	1.98658E-06	1.44671E-05	1.32414E-05	L-ribulokinase [EC:2.7.1.16]
K00854	5.58735E-06	5.22823E-06	2.87045E-05	3.81827E-05	xylulokinase [EC:2.7.1.17]
K00855	1.31343E-06	4.46935E-07	9.09606E-07	1.74282E-05	phosphoribulokinase [EC:2.7.1.19]
K00856	0.000217839	0.000217381	0.000207219	0.000201746	adenosine kinase [EC:2.7.1.20]
K00857	5.19764E-06	7.34738E-06	1.73142E-05	7.43958E-05	thymidine kinase [EC:2.7.1.21]
K00858	0.000223928	0.000225003	0.000229996	0.000228526	NAD+ kinase [EC:2.7.1.23]
K00859	0.000224598	0.000228192	0.000232881	0.000229639	dephospho-CoA kinase [EC:2.7.1.24]
K00860	0.000432333	0.000430791	0.000396336	0.000285587	adenylylsulfate kinase [EC:2.7.1.25]
K00862	5.20989E-08	1.39472E-07	8.82392E-07	8.20013E-09	erythritol kinase (D-erythritol 1-phosphate-forming) [EC:2.7.1.215]
K00863	4.05135E-06	4.88192E-06	2.80849E-05	4.49127E-05	triose/dihydroxyacetone kinase / FAD-AMP lyase (cyclizing) [EC:2.7.1.28 2.7.1.29 4.6.1.15]
K00864	0.000225414	0.000225822	0.000248003	0.000261335	glycerol kinase [EC:2.7.1.30]
K00865	4.53762E-06	3.4681E-06	2.32258E-05	2.46217E-05	glycerate 2-kinase [EC:2.7.1.165]
K00867	3.1543E-06	4.6602E-06	1.05932E-05	2.14698E-05	type I pantothenate kinase [EC:2.7.1.33]
K00868	8.95559E-06	1.16134E-05	4.33945E-05	0.000107879	pyridoxine kinase [EC:2.7.1.35]
K00869	1.3705E-06	8.59628E-07	3.58277E-06	5.03449E-06	mevalonate kinase [EC:2.7.1.36]
K00872	4.68994E-06	7.53642E-06	1.94246E-05	7.70004E-05	homoserine kinase [EC:2.7.1.39]
K00873	0.000439127	0.000439649	0.000420132	0.000358937	pyruvate kinase [EC:2.7.1.40]
K00874	0.000221223	0.000220019	0.000233263	0.000216843	2-dehydro-3-deoxygluconokinase [EC:2.7.1.45]
K00875	1.57581E-07	5.00489E-07	3.92731E-07	1.35343E-06	D-ribulokinase [EC:2.7.1.47]
K00876	6.84313E-06	7.3664E-06	2.15188E-05	2.39029E-05	uridine kinase [EC:2.7.1.48]
K00878	3.37143E-06	2.49763E-06	1.62598E-05	9.19058E-06	hydroxyethylthiazole kinase [EC:2.7.1.50]
K00879	1.27895E-07	2.3875E-07	4.45674E-08	3.50162E-07	L-fuculokinase [EC:2.7.1.51]
K00880	2.07304E-07	6.39146E-07	4.44366E-07	6.25726E-07	L-xylulokinase [EC:2.7.1.53]
K00881	7.70179E-08	2.96772E-07	9.88414E-08	2.167E-07	allose kinase [EC:2.7.1.55]
K00882	0.000218666	0.000218	0.000225308	0.000191212	1-phosphofructokinase [EC:2.7.1.56]
K00883	0.000215445	0.000212256	0.00019026	0.000132853	2-dehydro-3-deoxygalactonokinase [EC:2.7.1.58]
K00884	2.15774E-06	1.16025E-06	2.72505E-06	6.75735E-05	N-acetylglucosamine kinase [EC:2.7.1.59]
K00885	1.37557E-08	4.91407E-08	1.84341E-07	2.32717E-06	N-acylmannosamine kinase [EC:2.7.1.60]
K00886	1.82679E-06	4.22551E-06	1.28696E-05	7.62033E-06	polyphosphate glucokinase [EC:2.7.1.63]
K00887	1.26657E-06	3.77804E-07	7.01122E-06	3.63556E-06	undecaprenol kinase [EC:2.7.1.66]
K00891	0.000224582	0.000225556	0.000235243	0.000243852	shikimate kinase [EC:2.7.1.71]
K00892	9.82118E-08	3.28159E-07	3.90449E-07	2.56755E-05	inosine kinase [EC:2.7.1.73]
K00895	7.83412E-07	1.17245E-06	1.08829E-05	1.93984E-07	diphosphate-dependent phosphofructokinase [EC:2.7.1.90]
K00897	1.79759E-07	1.36845E-07	7.99267E-07	3.97161E-06	kanamycin kinase [EC:2.7.1.95]
K00899	5.07424E-07	8.41121E-07	1.10558E-06	3.11078E-06	5-methylthioribose kinase [EC:2.7.1.100]
K00901	0.000219848	0.000218987	0.000210211	0.000264143	diacylglycerol kinase (ATP) [EC:2.7.1.107]
K00903	3.32314E-07	2.55337E-07	1.03793E-06	3.80231E-07	protein-tyrosine kinase [EC:2.7.10.2]
K00906	0.000216244	0.000216171	0.00020599	0.000210405	isocitrate dehydrogenase kinase/phosphatase [EC:2.7.11.5 3.1.3.-]
K00912	0.000219733	0.000218788	0.000210462	0.000217708	tetraacyldisaccharide 4-kinase [EC:2.7.1.130]
K00917	1.05805E-06	1.53626E-07	3.77397E-06	7.25122E-06	tagatose 6-phosphate kinase [EC:2.7.1.144]
K00918	1.65487E-09	0	3.82026E-08	0	ADP-dependent phosphofructokinase/glucokinase [EC:2.7.1.146 2.7.1.147]
K00919	0.000223471	0.000223053	0.000228129	0.000233328	4-diphosphocytidyl-2-C-methyl-D-erythritol kinase [EC:2.7.1.148]
K00925	9.15104E-06	9.54143E-06	3.34998E-05	7.39442E-05	acetate kinase [EC:2.7.2.1]
K00926	2.57539E-06	1.5841E-06	1.47566E-05	2.61778E-05	carbamate kinase [EC:2.7.2.2]
K00927	0.000223838	0.000223811	0.000228218	0.000225495	phosphoglycerate kinase [EC:2.7.2.3]
K00928	0.000224958	0.000225309	0.000233321	0.000234164	aspartate kinase [EC:2.7.2.4]
K00929	3.53996E-07	9.49405E-07	1.36754E-06	5.04379E-07	butyrate kinase [EC:2.7.2.7]
K00930	0.000222296	0.000222128	0.000225061	0.000170936	acetylglutamate kinase [EC:2.7.2.8]
K00931	0.000222248	0.000222796	0.000225704	0.000228706	glutamate 5-kinase [EC:2.7.2.11]
K00932	2.33097E-07	9.48342E-07	1.16612E-06	2.82659E-06	propionate kinase [EC:2.7.2.15]
K00936	3.28597E-06	3.86761E-06	9.20443E-06	6.95739E-06	two-component system, sensor histidine kinase PdtaS [EC:2.7.13.3]
K00937	0.000222243	0.000221137	0.000222144	0.000219839	polyphosphate kinase [EC:2.7.4.1]
K00938	1.2938E-06	9.77477E-08	3.4134E-06	3.56213E-06	phosphomevalonate kinase [EC:2.7.4.2]
K00939	0.000223782	0.000224644	0.000228808	0.00022686	adenylate kinase [EC:2.7.4.3]
K00940	0.000222383	0.000223254	0.000223394	0.000222537	nucleoside-diphosphate kinase [EC:2.7.4.6]
K00941	0.000226755	0.000227133	0.000252385	0.000260742	hydroxymethylpyrimidine/phosphomethylpyrimidine kinase [EC:2.7.1.49 2.7.4.7]
K00942	0.000224765	0.000224269	0.000237174	0.000228837	guanylate kinase [EC:2.7.4.8]
K00943	0.000224073	0.000222997	0.000227377	0.000233726	dTMP kinase [EC:2.7.4.9]
K00945	0.000224346	0.000224493	0.00023523	0.000229049	CMP/dCMP kinase [EC:2.7.4.25]
K00946	0.000221327	0.000222939	0.00022066	0.000221628	thiamine-monophosphate kinase [EC:2.7.4.16]
K00947	6.99258E-08	7.35846E-09	2.03185E-08	0	molybdenum storage protein
K00948	0.000224295	0.000224191	0.000230731	0.000231459	ribose-phosphate pyrophosphokinase [EC:2.7.6.1]
K00949	2.85426E-06	2.59921E-06	8.94283E-06	3.9023E-06	thiamine pyrophosphokinase [EC:2.7.6.2]
K00950	0.000224123	0.000225202	0.000229883	0.000296271	2-amino-4-hydroxy-6-hydroxymethyldihydropteridine diphosphokinase [EC:2.7.6.3]
K00951	0.000442539	0.000442036	0.000437429	0.000443405	GTP pyrophosphokinase [EC:2.7.6.5]
K00952	1.5785E-06	6.35133E-08	9.27629E-07	3.56788E-08	nicotinamide-nucleotide adenylyltransferase [EC:2.7.7.1]
K00954	0.000223535	0.000223736	0.000228225	0.000225468	pantetheine-phosphate adenylyltransferase [EC:2.7.7.3]
K00955	0.000221443	0.000228034	0.000227362	0.000233472	bifunctional enzyme CysN/CysC [EC:2.7.7.4 2.7.1.25]
K00956	0.000218355	0.000222988	0.000209473	0.000174671	sulfate adenylyltransferase subunit 1 [EC:2.7.7.4]
K00957	0.000218864	0.000223272	0.000209661	0.000175389	sulfate adenylyltransferase subunit 2 [EC:2.7.7.4]
K00958	4.27224E-06	4.88339E-06	2.1609E-05	3.70076E-05	sulfate adenylyltransferase [EC:2.7.7.4]

K00960	1.44434E-10	2.06046E-07	4.87269E-08	2.64974E-09	DNA-directed RNA polymerase [EC:2.7.7.6]
K00962	0.000225227	0.000224552	0.000232965	0.00022772	polyribonucleotide nucleotidyltransferase [EC:2.7.7.8]
K00963	0.000437112	0.000435011	0.000412181	0.000350962	UTP--glucose-1-phosphate uridylyltransferase [EC:2.7.7.9]
K00965	1.18422E-06	1.21447E-06	7.60098E-06	2.61062E-05	UDPglucose--hexose-1-phosphate uridylyltransferase [EC:2.7.7.12]
K00966	1.60473E-06	2.05306E-06	7.18721E-06	4.20869E-06	mannose-1-phosphate guanylyltransferase [EC:2.7.7.13]
K00968	1.08316E-06	4.30756E-07	5.82351E-06	1.92811E-07	choline-phosphate cytidylyltransferase [EC:2.7.7.15]
K00969	0.000223387	0.000221186	0.000215852	0.000190797	nicotinate-nucleotide adenylyltransferase [EC:2.7.7.18]
K00970	0.000222424	0.000223443	0.000224129	0.0002219	poly(A) polymerase [EC:2.7.7.19]
K00971	7.77557E-06	1.19216E-05	2.93725E-05	8.84827E-05	mannose-1-phosphate guanylyltransferase [EC:2.7.7.13]
K00972	5.94393E-07	3.06799E-07	6.01104E-06	4.82287E-08	UDP-N-acetylglucosamine/UDP-N-acetylgalactosamine diphosphorylase [EC:2.7.7.23 2.7.7.83]
K00973	0.000223361	0.000225537	0.000225678	0.000251925	glucose-1-phosphate thymidylyltransferase [EC:2.7.7.24]
K00974	0.000437864	0.000435475	0.000412841	0.00035526	tRNA nucleotidyltransferase (CCA-adding enzyme) [EC:2.7.7.72 3.1.3.- 3.1.4.-]
K00975	5.29999E-06	1.04971E-05	2.42555E-05	4.23094E-05	glucose-1-phosphate adenylyltransferase [EC:2.7.7.27]
K00978	1.9375E-06	1.76958E-06	5.03361E-06	1.1381E-05	glucose-1-phosphate cytidylyltransferase [EC:2.7.7.33]
K00979	0.000219731	0.00021902	0.000211088	0.000218489	3-deoxy-manno-octulosonate cytidylyltransferase (CMP-KDO synthetase) [EC:2.7.7.38]
K00980	1.83743E-06	9.95801E-07	8.07832E-06	2.38477E-06	glycerol-3-phosphate cytidylyltransferase [EC:2.7.7.39]
K00981	0.000224696	0.000225102	0.000237168	0.000287226	phosphatidate cytidylyltransferase [EC:2.7.7.41]
K00982	0.000221046	0.00022128	0.000217109	0.000226233	[glutamine synthetase] adenylyltransferase / [glutamine synthetase]-adenylyl-L-tyrosine phosphorylase [EC:2.7.7.42 2.
K00983	1.78373E-06	1.21514E-06	5.64293E-06	1.35055E-05	N-acylneuraminate cytidylyltransferase [EC:2.7.7.43]
K00984	2.26845E-07	9.39033E-07	1.02965E-06	2.0527E-06	streptomycin 3\-adenylyltransferase [EC:2.7.7.47]
K00986	0.000221565	0.000219892	0.000305901	0.000198067	RNA-directed DNA polymerase [EC:2.7.7.49]
K00988	6.35144E-08	4.70051E-08	4.09739E-08	1.94044E-08	ATP adenylyltransferase [EC:2.7.7.53]
K00989	0.000220312	0.000218889	0.000204819	0.000187514	ribonuclease PH [EC:2.7.7.56]
K00990	0.000219725	0.000217688	0.000209324	0.000220324	[protein-PII] uridylyltransferase [EC:2.7.7.59]
K00991	0.000222931	0.000223054	0.000228729	0.000222705	2-C-methyl-D-erythritol 4-phosphate cytidylyltransferase [EC:2.7.7.60]
K00992	0.000219693	0.000218187	0.000210814	0.000210986	N-acetyl-alpha-D-muramate 1-phosphate uridylyltransferase [EC:2.7.7.99]
K00993	0	0	0	8.03716E-09	ethanolaminephosphotransferase [EC:2.7.8.1]
K00995	0.000227394	0.000228291	0.000244735	0.000279647	CDP-diacylglycerol---glycerol-3-phosphate 3-phosphatidyltransferase [EC:2.7.8.5]
K00996	0.000220196	0.000220142	0.000206085	0.000133844	undecaprenyl-phosphate galactose phosphotransferase [EC:2.7.8.6]
K00997	0.000434259	0.00043221	0.00040314	0.000282662	holo-[acyl-carrier protein] synthase [EC:2.7.8.7]
K00998	1.14511E-07	3.47662E-07	4.17848E-07	1.81772E-05	CDP-diacylglycerol---serine O-phosphatidyltransferase [EC:2.7.8.8]
K01000	0.000224008	0.000224392	0.000231766	0.000228315	phospho-N-acetylmuramoyl-pentapeptide-transferase [EC:2.7.8.13]
K01001	3.19681E-08	1.02051E-08	3.84612E-08	2.9957E-09	UDP-N-acetylglucosamine--dolichyl-phosphate N-acetylglucosaminephosphotransferase [EC:2.7.8.15]
K01002	1.147E-06	9.87276E-07	7.27864E-06	5.52374E-05	phosphoglycerol transferase [EC:2.7.8.20]
K01003	2.56407E-07	5.82597E-07	2.70909E-06	4.03356E-06	oxaloacetate decarboxylase [EC:4.1.1.3]
K01004	4.70265E-07	1.51948E-07	1.21674E-06	2.83359E-07	phosphatidylcholine synthase [EC:2.7.8.24]
K01006	4.45339E-06	2.52069E-06	1.69604E-05	3.98511E-06	pyruvate, orthophosphate dikinase [EC:2.7.9.1]
K01007	0.000217812	0.000218552	0.000208854	0.00022285	pyruvate, water dikinase [EC:2.7.9.2]
K01008	5.31552E-06	5.8511E-06	2.01429E-05	7.10875E-05	selenide, water dikinase [EC:2.7.9.3]
K01011	0.000441144	0.000448288	0.00045547	0.000491373	thiosulfate/3-mercaptopyruvate sulfurtransferase [EC:2.8.1.1 2.8.1.2]
K01012	0.000222512	0.000224241	0.000222987	0.000220673	biotin synthase [EC:2.8.1.6]
K01014	2.997E-09	0	1.50632E-07	2.1982E-09	aryl sulfotransferase [EC:2.8.2.1]
K01023	2.44979E-07	2.06574E-07	4.76602E-07	7.47113E-07	arylsulfate sulfotransferase [EC:2.8.2.22]
K01026	0.000217548	0.000214235	0.000199598	0.000138639	propionate CoA-transferase [EC:2.8.3.1]
K01027	2.37172E-06	4.44254E-06	4.96684E-06	6.12383E-05	3-oxoacid CoA-transferase [EC:2.8.3.5]
K01028	0.000222362	0.000225043	0.000227598	0.000275417	3-oxoacid CoA-transferase subunit A [EC:2.8.3.5]
K01029	0.000435316	0.000434251	0.000400001	0.000354498	3-oxoacid CoA-transferase subunit B [EC:2.8.3.5]
K01031	0.000217361	0.000215965	0.00020666	0.000174865	3-oxoadipate CoA-transferase, alpha subunit [EC:2.8.3.6]
K01032	0.000431154	0.000431137	0.000395035	0.000338343	3-oxoadipate CoA-transferase, beta subunit [EC:2.8.3.6]
K01034	7.31898E-07	1.2267E-06	3.46406E-06	1.14404E-05	acetate CoA/acetoacetate CoA-transferase alpha subunit [EC:2.8.3.8 2.8.3.9]
K01035	3.39561E-06	3.41249E-06	7.32528E-06	6.21066E-05	acetate CoA/acetoacetate CoA-transferase beta subunit [EC:2.8.3.8 2.8.3.9]
K01039	2.44075E-06	1.82299E-06	4.80666E-06	1.16421E-05	glutaconate CoA-transferase, subunit A [EC:2.8.3.12]
K01040	2.34285E-06	1.81972E-06	4.77875E-06	1.13031E-05	glutaconate CoA-transferase, subunit B [EC:2.8.3.12]
K01042	3.71611E-06	5.66961E-06	1.78085E-05	7.57486E-05	L-seryl-tRNA(Ser) seleniumtransferase [EC:2.9.1.1]
K01046	0.000221929	0.00021738	0.000236438	0.000198386	triacylglycerol lipase [EC:3.1.1.3]
K01048	6.35281E-06	2.90299E-06	2.07162E-05	2.57917E-05	lysophospholipase [EC:3.1.1.5]
K01051	0.000215926	0.000214796	0.00019656	0.000167906	pectinesterase [EC:3.1.1.11]
K01053	0.000222251	0.000218581	0.000223142	0.000187882	gluconolactonase [EC:3.1.1.17]
K01055	0.000433933	0.000434393	0.000418429	0.000360345	3-oxoadipate enol-lactonase [EC:3.1.1.24]
K01056	0.000223626	0.000224166	0.000229156	0.000225482	peptidyl-tRNA hydrolase, PTH1 family [EC:3.1.1.29]
K01057	0.000217745	0.000215965	0.000198747	0.000186225	6-phosphogluconolactonase [EC:3.1.1.31]
K01058	3.62734E-06	5.21181E-06	2.03279E-05	9.52726E-05	phospholipase A1/A2 [EC:3.1.1.32 3.1.1.4]
K01060	1.03766E-06	9.84367E-07	8.922E-06	4.95819E-06	cephalosporin-C deacetylase [EC:3.1.1.41]
K01061	0.000438649	0.000439576	0.000427732	0.000462087	carboxymethylenebutenolidase [EC:3.1.1.45]
K01066	0.001296248	0.001288304	0.001210848	0.000898943	acetyl esterase [EC:3.1.1.-]
K01067	1.58998E-06	5.16825E-06	1.90312E-05	3.80599E-05	acetyl-CoA hydrolase [EC:3.1.2.1]
K01069	0.000233582	0.000238864	0.000261522	0.000422023	hydroxyacylglutathione hydrolase [EC:3.1.2.6]
K01070	0.000219799	0.000217955	0.000208323	0.000220251	S-formylglutathione hydrolase [EC:3.1.2.12]
K01071	5.16613E-07	3.76239E-07	1.71657E-06	4.06958E-06	medium-chain acyl-[acyl-carrier-protein] hydrolase [EC:3.1.2.21]
K01073	3.86728E-07	6.77998E-07	3.25101E-06	1.08943E-05	acyl-CoA hydrolase [EC:3.1.2.20]
K01075	0.000430245	0.000427906	0.000389597	0.000261823	4-hydroxybenzoyl-CoA thioesterase [EC:3.1.2.23]
K01076	0	1.66614E-09	0	0	abhydrolase domain-containing protein 17 [EC:3.1.2.22]
K01077	0.000436566	0.000439042	0.000424003	0.0004218	alkaline phosphatase [EC:3.1.3.1]
K01079	0.000220778	0.000221067	0.000227338	0.000172697	phosphoserine phosphatase [EC:3.1.3.3]
K01081	8.74519E-06	1.40481E-05	4.27254E-05	0.000110352	5-nucleotidase [EC:3.1.3.5]
K01082	4.23299E-06	5.53365E-06	9.06763E-06	8.28937E-05	3(2), 5-bisphosphate nucleotidase [EC:3.1.3.7]
K01083	2.44788E-06	3.27899E-06	5.37349E-06	0.000112859	3-phytase [EC:3.1.3.8]
K01085	7.95156E-07	7.25474E-07	1.27053E-06	4.29169E-05	glucose-1-phosphatase [EC:3.1.3.10]
K01086	0.000218674	0.000216553	0.000206464	0.000196442	fructose-1,6-bisphosphatase I / sedoheptulose-1,7-bisphosphatase [EC:3.1.3.11 3.1.3.37]
K01087	0.00043679	0.000436126	0.000407564	0.000383139	trehalose 6-phosphate phosphatase [EC:3.1.3.12]
K01089	3.68438E-06	3.31042E-06	1.41917E-05	6.84457E-05	imidazoleglycerol-phosphate dehydratase / histidinol-phosphatase [EC:4.2.1.19 3.1.3.15]
K01090	1.77553E-06	3.60003E-06	1.59808E-05	3.84761E-05	protein phosphatase [EC:3.1.3.16]
K01091	0.000879373	0.000868819	0.000823715	0.000776727	phosphoglycolate phosphatase [EC:3.1.3.18]
K01092	0.000229952	0.000229908	0.00023802	0.000245067	myo-inositol-1(or 4)-monophosphatase [EC:3.1.3.25]
K01093	9.39541E-08	4.30406E-08	8.08122E-07	7.07452E-07	4-phytase / acid phosphatase [EC:3.1.3.26 3.1.3.2]
K01095	0.000216667	0.000216209	0.000206365	0.000166732	phosphatidylglycerophosphatase A [EC:3.1.3.27]
K01096	9.82312E-08	3.14629E-07	3.85911E-07	1.33185E-05	phosphatidylglycerophosphatase B [EC:3.1.3.27 3.1.3.81 3.1.3.4 3.6.1.27]

K01101	2.44902E-06	4.2702E-06	1.057E-05	9.14655E-06	4-nitrophenyl phosphatase [EC:3.1.3.41]
K01103	0	0	1.04657E-08	0	6-phosphofructo-2-kinase / fructose-2,6-biphosphatase 3 [EC:2.7.1.105 3.1.3.46]
K01104	0.000228621	0.000235277	0.000263503	0.00035877	protein-tyrosine phosphatase [EC:3.1.3.48]
K01113	9.93549E-06	1.61359E-05	4.79914E-05	0.000171721	alkaline phosphatase D [EC:3.1.3.1]
K01114	0.000644206	0.0006372	0.000570062	0.000393633	phospholipase C [EC:3.1.4.3]
K01115	1.23814E-06	5.6114E-07	1.89241E-06	2.82549E-05	phospholipase D1/2 [EC:3.1.4.4]
K01117	7.90181E-07	0	2.92298E-06	2.91551E-08	sphingomyelin phosphodiesterase [EC:3.1.4.12]
K01118	0.000217904	0.000218018	0.000214684	0.000226078	FMN-dependent NADH-azoreductase [EC:1.7.1.17]
K01119	0.000219025	0.000217124	0.000216958	0.000175634	2,3-cyclic-nucleotide 2-phosphodiesterase / 3-nucleotidase [EC:3.1.4.16 3.1.3.6]
K01120	6.98644E-08	3.31656E-07	1.37585E-07	2.69118E-08	3,5-cyclic-nucleotide phosphodiesterase [EC:3.1.4.17]
K01126	0.000447105	0.000452118	0.000486729	0.000439823	glycerophosphoryl diester phosphodiesterase [EC:3.1.4.46]
K01127	0	0	9.62333E-08	0	glycosylphosphatidylinositol phospholipase D [EC:3.1.4.50]
K01129	0.000220997	0.000222592	0.000219589	0.000178892	dGTPase [EC:3.1.5.1]
K01130	7.61412E-06	1.85868E-05	5.34784E-05	0.000111634	arylsulfatase [EC:3.1.6.1]
K01133	9.4266E-07	2.21839E-06	5.46882E-06	3.23625E-06	choline-sulfatase [EC:3.1.6.6]
K01134	5.33703E-07	2.07818E-06	5.13281E-07	1.07854E-07	arylsulfatase A [EC:3.1.6.8]
K01135	1.21323E-06	2.96022E-06	1.40337E-05	3.46132E-05	arylsulfatase B [EC:3.1.6.12]
K01136	3.38731E-07	9.5804E-07	1.77521E-06	5.1431E-08	iduronate 2-sulfatase [EC:3.1.6.13]
K01137	0	0	1.38151E-07	1.1358E-08	N-acetylglucosamine-6-sulfatase [EC:3.1.6.14]
K01138	9.54019E-07	3.34195E-06	6.30362E-06	2.33366E-06	uncharacterized sulfatase [EC:3.1.6.-]
K01139	0.000437254	0.000434317	0.000409592	0.000338125	GTP diphosphokinase / guanosine-3,5-bis(diphosphate) 3-diphosphatase [EC:2.7.6.5 3.1.7.2]
K01141	0.000216379	0.000216759	0.000205244	0.000217054	exodeoxyribonuclease I [EC:3.1.11.1]
K01142	0.000443522	0.000447593	0.000445745	0.000484036	exodeoxyribonuclease III [EC:3.1.11.2]
K01143	5.56136E-07	3.26143E-08	1.10472E-07	3.70781E-07	exodeoxyribonuclease (lambda-induced) [EC:3.1.11.3]
K01144	2.00992E-06	1.62507E-06	2.98395E-06	2.59685E-06	exodeoxyribonuclease V [EC:3.1.11.5]
K01146	1.59097E-07	4.39552E-07	6.91037E-07	1.70162E-05	protein Xni
K01147	0.000215438	0.00021547	0.000203625	0.000160236	exoribonuclease II [EC:3.1.13.1]
K01150	4.52807E-07	7.7368E-07	2.45533E-06	1.82378E-05	deoxyribonuclease I [EC:3.1.21.1]
K01151	2.85503E-06	2.38723E-06	1.65601E-05	1.45159E-05	deoxyribonuclease IV [EC:3.1.21.2]
K01153	0.000650902	0.000648194	0.000595806	0.000435742	type I restriction enzyme, R subunit [EC:3.1.21.3]
K01154	0.000221358	0.000224394	0.0002207	0.000190686	type I restriction enzyme, S subunit [EC:3.1.21.3]
K01155	2.49805E-06	2.38894E-06	2.25986E-06	2.77871E-06	type II restriction enzyme [EC:3.1.21.4]
K01156	3.10773E-06	2.76554E-06	8.82859E-06	1.67623E-05	type III restriction enzyme [EC:3.1.21.5]
K01158	0	7.49763E-09	8.92996E-08	3.42313E-09	deoxyribonuclease II [EC:3.1.22.1]
K01159	0.000223283	0.000225187	0.000230957	0.000242871	crossover junction endodeoxyribonuclease RuvC [EC:3.1.22.4]
K01160	3.84257E-07	1.74467E-07	6.38637E-07	5.62985E-06	crossover junction endodeoxyribonuclease RusA [EC:3.1.22.4]
K01161	0	0	3.27523E-08	0	deoxyribonuclease (pyrimidine dimer) [EC:3.1.25.1]
K01163	6.74483E-07	9.64923E-07	4.74474E-06	1.5401E-07	uncharacterized protein
K01166	2.70224E-06	1.78539E-07	2.35459E-06	3.62987E-06	ribonuclease T2 [EC:3.1.27.1]
K01167	0.000216647	0.00021735	0.000211299	0.000199636	ribonuclease T1 [EC:3.1.27.3]
K01169	2.83781E-08	1.95821E-08	5.51403E-08	7.36361E-07	ribonuclease I (enterobacter ribonuclease) [EC:3.1.27.6]
K01173	0.000220163	0.000216599	0.000208888	0.000195682	endonuclease G, mitochondrial
K01174	3.79905E-06	5.19211E-06	1.27292E-05	1.2408E-05	micrococcal nuclease [EC:3.1.31.1]
K01175	3.75621E-06	5.97623E-06	9.59314E-06	6.96004E-05	esterase [EC:3.1.-.-]
K01176	2.61193E-06	5.36346E-06	2.31599E-05	4.48596E-05	alpha-amylase [EC:3.2.1.1]
K01178	0.00021759	0.000216197	0.000189624	0.000120324	glucoamylase [EC:3.2.1.3]
K01179	0.000431292	0.000426887	0.000392783	0.000239487	endoglucanase [EC:3.2.1.4]
K01181	2.46383E-06	1.08088E-06	3.78875E-06	1.99318E-05	endo-1,4-beta-xylanase [EC:3.2.1.8]
K01182	5.52202E-06	7.12776E-06	2.87732E-05	4.23769E-05	oligo-1,6-glucosidase [EC:3.2.1.10]
K01183	0.000218553	0.000215077	0.000201468	0.000279023	chitinase [EC:3.2.1.14]
K01184	0.000427628	0.000423567	0.000372405	0.000226502	polygalacturonase [EC:3.2.1.15]
K01185	0.000434708	0.000432974	0.000415441	0.000385178	lysozyme [EC:3.2.1.17]
K01186	0.000216318	0.00021496	0.000210525	0.00011636	sialidase-1 [EC:3.2.1.18]
K01187	0.000443888	0.000442162	0.000433458	0.000452415	alpha-glucosidase [EC:3.2.1.20]
K01188	3.19681E-08	1.66614E-09	0	1.90174E-09	beta-glucosidase [EC:3.2.1.21]
K01190	8.57387E-06	1.63585E-05	3.04061E-05	2.55274E-05	beta-galactosidase [EC:3.2.1.23]
K01191	1.22409E-06	8.41893E-07	1.35933E-05	3.95697E-06	alpha-mannosidase [EC:3.2.1.24]
K01192	3.05382E-06	2.17849E-06	1.05964E-05	6.15458E-05	beta-mannosidase [EC:3.2.1.25]
K01193	0.000215944	0.000214678	0.00019958	0.00013372	beta-fructofuranosidase [EC:3.2.1.26]
K01194	0.000215643	0.00021627	0.000196465	0.00012961	alpha,alpha-trehalase [EC:3.2.1.28]
K01195	1.20669E-06	1.01083E-06	8.7183E-06	9.09045E-07	beta-glucuronidase [EC:3.2.1.31]
K01197	5.10629E-07	1.36887E-06	3.05266E-06	3.41433E-06	hyaluronoglucosaminidase [EC:3.2.1.35]
K01198	3.32885E-06	5.42212E-06	9.68232E-06	3.46402E-05	xylan 1,4-beta-xylosidase [EC:3.2.1.37]
K01200	1.64416E-06	2.15879E-06	1.25395E-05	3.25481E-05	pullulanase [EC:3.2.1.41]
K01201	5.75108E-07	8.15102E-07	3.38935E-06	1.25522E-05	glucosylceramidase [EC:3.2.1.45]
K01205	3.87053E-07	2.19276E-06	2.23247E-06	7.83466E-08	alpha-N-acetylglucosaminidase [EC:3.2.1.50]
K01206	4.42409E-06	1.1294E-05	1.3148E-05	4.0716E-06	alpha-L-fucosidase [EC:3.2.1.51]
K01207	0.000222418	0.000221885	0.000230564	0.000219798	beta-N-acetylhexosaminidase [EC:3.2.1.52]
K01208	4.86902E-06	3.47129E-06	1.83171E-05	2.48184E-05	cyclomaltodextrinase / maltogenic alpha-amylase / neopullulanase [EC:3.2.1.54 3.2.1.133 3.2.1.135]
K01209	3.03446E-06	4.76101E-06	1.34785E-05	6.98282E-06	alpha-N-arabinofuranosidase [EC:3.2.1.55]
K01210	8.12435E-07	1.0875E-07	3.68633E-07	2.22598E-07	glucan 1,3-beta-glucosidase [EC:3.2.1.58]
K01212	1.58404E-07	2.56197E-07	2.90274E-07	9.08342E-08	levanase [EC:3.2.1.65]
K01214	0.00022034	0.000217699	0.000206482	0.000188222	isoamylase [EC:3.2.1.68]
K01215	2.78749E-06	1.20128E-06	7.1391E-06	2.50481E-05	glucan 1,6-alpha-glucosidase [EC:3.2.1.70]
K01216	1.03837E-09	3.18986E-07	4.71545E-08	1.53121E-08	licheninase [EC:3.2.1.73]
K01218	5.35755E-07	1.79301E-06	3.24577E-06	2.20637E-06	mannan endo-1,4-beta-mannosidase [EC:3.2.1.78]
K01219	1.32724E-08	2.54415E-08	2.01732E-08	3.15541E-06	beta-agarase [EC:3.2.1.81]
K01220	1.17578E-06	6.64199E-08	5.86536E-06	3.30769E-06	6-phospho-beta-galactosidase [EC:3.2.1.85]
K01222	1.11278E-06	9.71109E-07	9.54452E-06	3.0574E-06	6-phospho-beta-glucosidase [EC:3.2.1.86]
K01223	4.60731E-06	3.56302E-06	2.74055E-05	4.24198E-05	6-phospho-beta-glucosidase [EC:3.2.1.86]
K01224	7.85404E-07	1.49194E-06	1.01602E-06	3.60379E-06	arabinogalactan endo-1,4-beta-galactosidase [EC:3.2.1.89]
K01226	5.21212E-06	7.69869E-06	1.69567E-05	3.72338E-05	trehalose-6-phosphate hydrolase [EC:3.2.1.93]
K01227	3.28359E-06	4.50812E-07	1.26012E-05	4.02993E-06	mannosyl-glycoprotein endo-beta-N-acetylglucosaminidase [EC:3.2.1.96]
K01232	1.93045E-07	1.48186E-07	4.48739E-07	2.10616E-06	maltose-6-phosphate glucosidase [EC:3.2.1.122]
K01233	0	1.0765E-08	5.82801E-07	6.98357E-08	chitosanase [EC:3.2.1.132]
K01235	4.30717E-07	6.33131E-07	8.66541E-07	4.23432E-06	alpha-glucuronidase [EC:3.2.1.139]
K01236	0.000219109	0.000220005	0.000201163	0.000162053	maltooligosyltrehalose trehalohydrolase [EC:3.2.1.141]

K01239	6.84926E-06	7.39735E-06	2.19953E-05	0.000117303	purine nucleosidase [EC:3.2.2.1]
K01241	0.000217687	0.00021809	0.000207594	0.000200674	AMP nucleosidase [EC:3.2.2.4]
K01243	0.000219667	0.000217921	0.000221737	0.000174759	adenosylhomocysteine nucleosidase [EC:3.2.2.9]
K01246	0.000222791	0.00022315	0.000220234	0.000226528	DNA-3-methyladenine glycosylase I [EC:3.2.2.20]
K01247	0.000434335	0.000432041	0.000408038	0.000343773	DNA-3-methyladenine glycosylase II [EC:3.2.2.21]
K01250	2.89091E-06	4.48654E-06	1.22223E-05	8.28161E-06	pyrimidine-specific ribonucleoside hydrolase [EC:3.2.-.-]
K01251	0.000220568	0.000222118	0.000213768	0.00020774	adenosylhomocysteinase [EC:3.3.1.1]
K01252	1.93021E-06	1.28494E-06	3.34363E-06	0.000118929	bifunctional isochorismate lyase / aryl carrier protein [EC:3.3.2.1 6.3.2.14]
K01253	1.65968E-06	7.23802E-07	1.31215E-06	2.56891E-06	microsomal epoxide hydrolase [EC:3.3.2.9]
K01255	0.00022603	0.000226554	0.000235985	0.000335752	leucyl aminopeptidase [EC:3.4.11.1]
K01256	0.000436021	0.000438096	0.000412846	0.000324107	aminopeptidase N [EC:3.4.11.2]
K01258	4.35699E-06	3.27262E-06	1.54883E-05	2.61223E-05	tripeptide aminopeptidase [EC:3.4.11.4]
K01259	0.000435639	0.000431699	0.000399413	0.00038473	proline iminopeptidase [EC:3.4.11.5]
K01261	2.66967E-06	1.31934E-06	7.8006E-06	4.3412E-06	glutamyl aminopeptidase [EC:3.4.11.7]
K01262	0.000226865	0.000227111	0.000240417	0.000249382	Xaa-Pro aminopeptidase [EC:3.4.11.9]
K01263	7.18254E-08	2.23385E-07	7.71278E-07	5.11612E-06	cytosol alanyl aminopeptidase [EC:3.4.11.14]
K01265	0.000228801	0.000233396	0.000260045	0.000319952	methionyl aminopeptidase [EC:3.4.11.18]
K01266	0.00021734	0.000217771	0.000209428	0.00015036	D-aminopeptidase [EC:3.4.11.19]
K01267	2.68259E-06	5.05292E-06	1.4562E-05	2.71474E-06	aspartyl aminopeptidase [EC:3.4.11.21]
K01269	1.70241E-06	7.5441E-07	4.2087E-06	3.45489E-06	aminopeptidase [EC:3.4.11.-]
K01270	0.000215508	0.000214676	0.00019253	0.000135953	dipeptidase D [EC:3.4.13.-]
K01271	0.000222821	0.000220666	0.000213076	0.00020868	Xaa-Pro dipeptidase [EC:3.4.13.9]
K01273	3.82964E-06	3.65439E-06	9.85544E-06	6.19429E-05	membrane dipeptidase [EC:3.4.13.19]
K01274	2.74357E-07	6.67879E-07	2.25678E-06	5.49707E-06	D-alanyl-D-alanine dipeptidase [EC:3.4.13.-]
K01277	2.52551E-07	7.03627E-07	2.57884E-06	2.18841E-08	dipeptidyl-peptidase III [EC:3.4.14.4]
K01278	4.57285E-06	9.15185E-06	2.11226E-05	0.000105234	dipeptidyl-peptidase 4 [EC:3.4.14.5]
K01281	2.20424E-07	1.23852E-07	1.431E-06	7.72906E-06	X-Pro dipeptidyl-peptidase [EC:3.4.14.11]
K01283	1.26143E-06	1.24592E-06	2.24148E-06	5.4448E-05	peptidyl-dipeptidase A [EC:3.4.15.1]
K01284	0.000223063	0.000224095	0.000209723	0.000303872	peptidyl-dipeptidase Dcp [EC:3.4.15.5]
K01286	1.22391E-05	1.29749E-05	2.4285E-05	0.000277168	D-alanyl-D-alanine carboxypeptidase [EC:3.4.16.4]
K01295	0.00043381	0.000433227	0.000420813	0.000336445	glutamate carboxypeptidase [EC:3.4.17.11]
K01297	0.000218853	0.000219125	0.000217925	0.000172985	muramoyltetrapeptide carboxypeptidase [EC:3.4.17.13]
K01299	1.5683E-06	7.0827E-07	1.81575E-06	1.73395E-05	carboxypeptidase Taq [EC:3.4.17.19]
K01301	1.62699E-07	4.10544E-08	1.0306E-07	2.09243E-08	N-acetylated-alpha-linked acidic dipeptidase [EC:3.4.17.21]
K01303	4.29485E-06	6.47391E-06	1.92767E-05	0.000104656	acylaminoacyl-peptidase [EC:3.4.19.1]
K01304	0.000431689	0.000429939	0.000391746	0.000266185	pyroglutamyl-peptidase [EC:3.4.19.3]
K01305	4.73301E-09	9.80703E-08	1.26135E-06	5.64091E-06	beta-aspartyl-dipeptidase (metallo-type) [EC:3.4.19.-]
K01308	1.17551E-07	1.04912E-06	1.48411E-07	1.89155E-06	g-D-glutamyl-meso-diaminopimelate peptidase [EC:3.4.19.11]
K01312	3.97652E-07	2.79232E-07	1.59579E-06	3.24905E-05	trypsin [EC:3.4.21.4]
K01318	3.17144E-06	3.5778E-06	6.96903E-06	0.000104351	glutamyl endopeptidase [EC:3.4.21.19]
K01322	2.93144E-06	4.22272E-06	8.38278E-06	6.25631E-05	prolyl oligopeptidase [EC:3.4.21.26]
K01337	1.33639E-06	3.77371E-06	2.02683E-05	4.25418E-05	lysyl endopeptidase [EC:3.4.21.50]
K01338	0.000229466	0.000224528	0.000230726	0.000238784	ATP-dependent Lon protease [EC:3.4.21.53]
K01342	6.93343E-07	2.39919E-06	1.10975E-05	1.83584E-05	subtilisin [EC:3.4.21.62]
K01347	4.61855E-10	1.14873E-09	1.82618E-08	4.94158E-08	IgA-specific serine endopeptidase [EC:3.4.21.72]
K01354	3.91165E-06	7.0268E-06	7.93811E-06	8.07014E-05	oligopeptidase B [EC:3.4.21.83]
K01355	3.35772E-09	1.69551E-08	3.46461E-08	1.6504E-07	omptin [EC:3.4.23.49]
K01356	0.00022456	0.000224535	0.000230461	0.000231317	repressor LexA [EC:3.4.21.88]
K01358	0.000229621	0.000234472	0.000262436	0.000314471	ATP-dependent Clp protease, protease subunit [EC:3.4.21.92]
K01361	1.36699E-06	1.0493E-06	4.69038E-06	1.84559E-05	lactocepin [EC:3.4.21.96]
K01364	0	0	1.91871E-08	1.63347E-08	streptopain [EC:3.4.22.10]
K01372	1.91998E-06	3.10426E-06	6.93068E-06	3.86862E-06	bleomycin hydrolase [EC:3.4.22.40]
K01387	2.89403E-07	1.81811E-07	2.85765E-06	3.68742E-05	microbial collagenase [EC:3.4.24.3]
K01389	0	1.06439E-08	0	6.78334E-09	neprilysin [EC:3.4.24.11]
K01390	1.09932E-07	1.5884E-07	2.86124E-07	4.4734E-07	IgA-specific metalloendopeptidase [EC:3.4.24.13]
K01392	1.26753E-07	8.97913E-08	2.18946E-06	1.4257E-06	thimet oligopeptidase [EC:3.4.24.15]
K01399	6.38821E-08	4.7573E-07	6.73179E-07	6.89286E-06	pseudolysin [EC:3.4.24.26]
K01400	1.55488E-06	1.01478E-06	4.35044E-06	3.28435E-06	bacillolysin [EC:3.4.24.28]
K01401	1.0261E-06	4.32978E-07	3.08257E-06	1.01824E-06	aureolysin [EC:3.4.24.29]
K01406	0.000857837	0.000851461	0.000763156	0.000494613	serralysin [EC:3.4.24.40]
K01407	2.33087E-07	3.58877E-07	4.22195E-07	1.96917E-05	protease III [EC:3.4.24.55]
K01408	1.55345E-07	4.49486E-07	1.05673E-06	7.59754E-06	insulysin [EC:3.4.24.56]
K01409	0.00022465	0.000225689	0.00023409	0.000238241	N6-L-threonylcarbamoyladenine synthase [EC:2.3.1.234]
K01414	0.000220774	0.000220624	0.000215363	0.000272901	oligopeptidase A [EC:3.4.24.70]
K01415	2.45717E-07	1.55594E-06	1.03402E-06	1.32749E-05	endothelin-converting enzyme [EC:3.4.24.71]
K01416	0	0	6.15299E-07	3.57207E-09	snapalysin [EC:3.4.24.77]
K01417	9.16819E-09	4.42717E-08	1.76926E-07	1.21072E-08	extracellular elastinolytic metalloproteinase [EC:3.4.24.-]
K01419	0.00021937	0.000217355	0.000210493	0.000218288	ATP-dependent HslUV protease, peptidase subunit HslV [EC:3.4.25.2]
K01420	0.00022263	0.000220287	0.000211068	0.000259349	CRP/FNR family transcriptional regulator, anaerobic regulatory protein
K01421	4.73053E-06	5.23703E-06	2.91318E-05	1.1776E-05	putative membrane protein
K01423	0.000218975	0.00021777	0.000211897	0.000223724	beta-barrel assembly-enhancing protease [EC:3.4.-.-]
K01424	0.00022674	0.000230835	0.00025969	0.000299164	L-asparaginase [EC:3.5.1.1]
K01425	0.000217994	0.000221154	0.000193136	0.000137034	glutaminase [EC:3.5.1.2]
K01426	0.000452865	0.000449314	0.000493706	0.000539054	amidase [EC:3.5.1.4]
K01427	0	0	1.23172E-08	5.4955E-10	urease [EC:3.5.1.5]
K01428	0.000221735	0.000222427	0.000226608	0.000236253	urease subunit alpha [EC:3.5.1.5]
K01429	0.000219527	0.000218683	0.000210335	0.000152928	urease subunit beta [EC:3.5.1.5]
K01430	0.000219526	0.000218555	0.000210597	0.000152941	urease subunit gamma [EC:3.5.1.5]
K01431	1.09029E-06	9.59289E-07	1.17765E-06	1.94146E-06	beta-ureidopropionase [EC:3.5.1.6]
K01432	6.3777E-06	4.84936E-06	2.25456E-05	3.72649E-05	arylformamidase [EC:3.5.1.9]
K01433	0.000434829	0.000435541	0.000407422	0.000336053	formyltetrahydrofolate deformylase [EC:3.5.1.10]
K01434	0.000217598	0.00021706	0.000207112	0.000205715	penicillin amidase [EC:3.5.1.11]
K01436	0.000666592	0.000671268	0.000693449	0.000712621	amidohydrolase [EC:3.5.1.-]
K01437	0	0	1.01987E-08	3.73779E-07	aspartoacylase [EC:3.5.1.15]
K01438	0.000223666	0.000220873	0.000230944	0.000248808	acetylornithine deacetylase [EC:3.5.1.16]
K01439	0.0004455	0.000447163	0.000460535	0.000429883	succinyl-diaminopimelate desuccinylase [EC:3.5.1.18]
K01442	4.64204E-06	5.3925E-06	2.9512E-05	9.79462E-05	choloylglycine hydrolase [EC:3.5.1.24]

K01443	7.62158E-06	1.15098E-05	2.85862E-05	0.000137678	N-acetylglucosamine-6-phosphate deacetylase [EC:3.5.1.25]
K01444	2.69632E-06	3.65105E-06	1.8494E-05	8.70909E-05	N4-(beta-N-acetylglucosaminyl)-L-asparaginase [EC:3.5.1.26]
K01446	4.08517E-08	3.33228E-09	2.18751E-07	1.0845E-07	peptidoglycan recognition protein
K01447	6.5447E-06	7.5252E-06	8.48905E-06	0.000105783	N-acetylmuramoyl-L-alanine amidase [EC:3.5.1.28]
K01448	0.000224328	0.000225857	0.000236944	0.000231399	N-acetylmuramoyl-L-alanine amidase [EC:3.5.1.28]
K01449	2.93352E-06	1.49466E-06	4.0255E-06	2.89593E-07	N-acetylmuramoyl-L-alanine amidase [EC:3.5.1.28]
K01451	0.000663695	0.000667105	0.000684995	0.000660005	hippurate hydrolase [EC:3.5.1.32]
K01452	0.000215386	0.000215517	0.000204198	0.000153804	chitin deacetylase [EC:3.5.1.41]
K01453	0	1.44985E-08	2.20645E-08	5.02891E-08	6-aminohexanoate-oligomer exohydrolase [EC:3.5.1.46]
K01455	7.45029E-06	1.00009E-05	4.59391E-05	0.000108003	formamidase [EC:3.5.1.49]
K01457	0.000219628	0.000215348	0.000203784	0.000155213	allophanate hydrolase [EC:3.5.1.54]
K01458	6.24641E-06	1.12838E-05	5.25981E-05	0.000122215	N-formylglutamate deformylase [EC:3.5.1.68]
K01459	1.04336E-07	4.678E-08	2.1218E-06	1.91935E-07	N-carbamoyl-D-amino-acid hydrolase [EC:3.5.1.77]
K01460	6.14906E-08	2.45914E-08	9.7036E-08	2.91602E-06	glutathionylspermidine amidase/synthetase [EC:3.5.1.78 6.3.1.8]
K01461	8.46938E-08	1.64246E-07	2.80544E-07	9.66745E-07	N-acyl-D-glutamate deacylase [EC:3.5.1.82]
K01462	0.000442391	0.000443436	0.000444022	0.00038676	peptide deformylase [EC:3.5.1.88]
K01463	3.0613E-06	4.64336E-06	3.32786E-06	6.10622E-05	N-acetylglucosamine malate deacetylase 1 [EC:3.5.1.-]
K01464	3.87578E-06	3.65785E-06	2.1439E-05	4.10732E-05	dihydropyrimidinase [EC:3.5.2.2]
K01465	0.000447971	0.000442439	0.000438243	0.000390366	dihydroorotase [EC:3.5.2.3]
K01466	6.9269E-07	3.90241E-07	5.15957E-06	3.41105E-06	allantoinase [EC:3.5.2.5]
K01467	1.913E-06	3.11715E-06	4.9859E-06	8.69176E-05	beta-lactamase class C [EC:3.5.2.6]
K01468	0.000222808	0.000227273	0.000223155	0.000351611	imidazolonepropionase [EC:3.5.2.7]
K01469	1.40057E-06	3.28952E-06	1.77485E-05	3.50202E-05	5-oxoprolinase (ATP-hydrolysing) [EC:3.5.2.9]
K01470	3.80147E-06	5.39268E-06	1.8766E-05	3.72076E-05	creatinine amidohydrolase [EC:3.5.2.10]
K01473	1.48261E-06	4.24373E-06	1.89138E-05	3.80992E-05	N-methylhydantoinase A [EC:3.5.2.14]
K01474	1.52259E-06	3.90112E-06	2.01037E-05	3.74447E-05	N-methylhydantoinase B [EC:3.5.2.14]
K01476	0.000431479	0.000428558	0.000392564	0.000318971	arginase [EC:3.5.3.1]
K01477	0.000214805	0.000211996	0.000186991	0.000115038	allantoicase [EC:3.5.3.4]
K01478	2.33152E-06	1.21436E-06	1.25459E-05	2.84183E-05	arginine deiminase [EC:3.5.3.6]
K01479	0.000218929	0.000221087	0.000211635	0.000222754	formiminoglutamase [EC:3.5.3.8]
K01480	0.00021766	0.000220404	0.000205909	0.000169949	agmatinase [EC:3.5.3.11]
K01481	0	0	9.96802E-08	4.46004E-08	protein-arginine deiminase [EC:3.5.3.15]
K01482	4.92672E-08	9.92051E-08	3.01844E-07	1.39565E-07	dimethylargininase [EC:3.5.3.18]
K01483	0.000217139	0.000215662	0.000204401	0.00014838	ureidoglycolate lyase [EC:4.3.2.3]
K01484	1.13771E-06	4.67832E-07	2.17537E-06	1.16578E-05	succinylarginine dihydrolase [EC:3.5.3.23]
K01485	0.000220635	0.00022094	0.000225344	0.000297457	cytosine deaminase [EC:3.5.4.1]
K01486	2.08489E-06	1.14675E-06	5.13041E-06	3.34492E-06	adenine deaminase [EC:3.5.4.2]
K01487	0.000435932	0.000436808	0.00042416	0.00038367	guanine deaminase [EC:3.5.4.3]
K01488	0.000218024	0.00021933	0.000211457	0.000175504	adenosine deaminase [EC:3.5.4.4]
K01489	5.35442E-06	6.32183E-06	2.01641E-05	2.56935E-05	cytidine deaminase [EC:3.5.4.5]
K01491	0.000222962	0.000224023	0.000229588	0.000227792	methylenetetrahydrofolate dehydrogenase (NADP+) / methenyltetrahydrofolate cyclohydrolase [EC:1.5.1.5 3.5.4.9]
K01492	9.00703E-07	3.00438E-06	4.64896E-07	1.92103E-07	phosphoribosylglycinamide/phosphoribosylaminoimidazolecarboxamide formyltransferase [EC:2.1.2.2 2.1.2.3]
K01493	0.000217201	0.000216023	0.000208778	0.00014265	dCMP deaminase [EC:3.5.4.12]
K01494	0.000220641	0.000221002	0.000214388	0.000213268	dCTP deaminase [EC:3.5.4.13]
K01495	0.000222443	0.000223297	0.000223584	0.000225183	GTP cyclohydrolase IA [EC:3.5.4.16]
K01496	0.000222082	0.000221599	0.00022526	0.00018527	phosphoribosyl-AMP cyclohydrolase [EC:3.5.4.19]
K01497	5.97532E-06	6.69835E-06	2.00542E-05	7.45457E-05	GTP cyclohydrolase II [EC:3.5.4.25]
K01498	0	0	6.13695E-09	7.53847E-09	diaminohydroxyphosphoribosylaminopyrimidine deaminase [EC:3.5.4.26]
K01499	9.87446E-07	4.21631E-08	6.3322E-08	1.33207E-07	methenyltetrahydromethanopterin cyclohydrolase [EC:3.5.4.27]
K01501	1.38606E-06	1.26716E-06	3.01714E-06	2.62639E-06	nitrilase [EC:3.5.5.1]
K01502	0.000215253	0.000212696	0.000188242	0.000113463	aliphatic nitrilase [EC:3.5.5.7]
K01505	0.000215372	0.000213484	0.000190571	0.000132251	1-aminocyclopropane-1-carboxylate deaminase [EC:3.5.99.7]
K01507	0.000222851	0.000222885	0.000223027	0.000268805	inorganic pyrophosphatase [EC:3.6.1.1]
K01512	0.000430986	0.000426393	0.000379242	0.000246207	acylphosphatase [EC:3.6.1.7]
K01514	0	0	0	2.67905E-09	exopolyphosphatase [EC:3.6.1.11]
K01515	0.000223783	0.000222891	0.000221174	0.000205328	ADP-ribose pyrophosphatase [EC:3.6.1.13]
K01519	0	0	0	8.03716E-09	inosine triphosphate pyrophosphatase [EC:3.6.1.-]
K01520	0.000222596	0.000223438	0.000224984	0.000213665	dUTP pyrophosphatase [EC:3.6.1.23]
K01521	3.08221E-06	1.41195E-06	1.82401E-06	5.2258E-05	CDP-diacylglycerol pyrophosphatase [EC:3.6.1.26]
K01523	0.000221064	0.000220443	0.000220246	0.000181569	phosphoribosyl-ATP pyrophosphohydrolase [EC:3.6.1.31]
K01524	0.000227547	0.000233167	0.000243907	0.000280797	exopolyphosphatase / guanosine-5-triphosphate,3-diphosphate pyrophosphatase [EC:3.6.1.11 3.6.1.40]
K01525	0.00021786	0.000217237	0.000208143	0.000222509	bis(5-nucleosyl)-tetraphosphatase (symmetrical) [EC:3.6.1.41]
K01531	5.03206E-06	5.19326E-06	1.89857E-05	8.78805E-05	Mg2+-importing ATPase [EC:3.6.3.2]
K01533	0.00045203	0.000455986	0.000476717	0.000500151	Cu2+-exporting ATPase [EC:3.6.3.4]
K01534	0.000454097	0.000455063	0.000479853	0.000506987	Cd2+/Zn2+-exporting ATPase [EC:3.6.3.3 3.6.3.5]
K01535	2.77316E-06	9.29026E-07	2.99677E-06	2.76987E-06	H+-transporting ATPase [EC:3.6.3.6]
K01537	8.13967E-06	7.13771E-06	2.66903E-05	7.37563E-05	Ca2+-transporting ATPase [EC:3.6.3.8]
K01539	1.57612E-07	8.22929E-08	2.85773E-06	2.70296E-06	sodium/potassium-transporting ATPase subunit alpha [EC:3.6.3.9]
K01541	5.29887E-08	7.94201E-08	5.00404E-08	2.24847E-06	H+/K+-exchanging ATPase [EC:3.6.3.10]
K01545	0	2.72195E-09	1.91031E-07	1.95069E-08	K+-transporting ATPase ATPase F chain
K01546	0.000219518	0.000217769	0.000217348	0.000205954	K+-transporting ATPase ATPase A chain [EC:3.6.3.12]
K01547	0.000221966	0.000218946	0.000219497	0.000241789	K+-transporting ATPase ATPase B chain [EC:3.6.3.12]
K01548	0.000219566	0.000217691	0.00021604	0.000205921	K+-transporting ATPase ATPase C chain [EC:3.6.3.12]
K01551	3.34391E-06	2.60021E-06	6.90095E-06	7.23939E-05	arsenite/tail-anchored protein-transporting ATPase [EC:3.6.3.16 3.6.3.-]
K01555	0.00021534	0.000216228	0.00020411	0.000153678	fumarylacetoacetase [EC:3.7.1.2]
K01556	0.00021637	0.000217608	0.000206351	0.000209027	kynureninase [EC:3.7.1.3]
K01560	0.000647264	0.000637519	0.000576308	0.000353804	2-haloacid dehalogenase [EC:3.8.1.2]
K01561	0.000432306	0.000428379	0.000390965	0.000295931	haloacetate dehalogenase [EC:3.8.1.3]
K01563	0.000434942	0.000432607	0.000398645	0.000327186	haloalkane dehalogenase [EC:3.8.1.5]
K01565	1.21255E-06	2.93338E-06	1.45931E-05	3.63202E-05	N-sulfoglucosamine sulfohydrolase [EC:3.10.1.1]
K01567	8.98698E-07	6.27192E-07	2.22583E-06	4.64249E-07	peptidoglycan-N-acetylmuramic acid deacetylase [EC:3.5.1.-]
K01568	9.82047E-08	3.4017E-07	7.84148E-07	2.38278E-08	pyruvate decarboxylase [EC:4.1.1.1]
K01569	2.27542E-06	6.08222E-07	5.0177E-07	1.29775E-05	oxalate decarboxylase [EC:4.1.1.2]
K01571	2.37407E-06	3.56416E-06	1.36715E-05	2.10009E-05	oxaloacetate decarboxylase, alpha subunit [EC:4.1.1.3]
K01572	2.58664E-06	3.72645E-06	5.23445E-06	1.89319E-05	oxaloacetate decarboxylase, beta subunit [EC:4.1.1.3]
K01573	1.94416E-07	7.87741E-07	1.1364E-06	1.76765E-05	oxaloacetate decarboxylase, gamma subunit [EC:4.1.1.3]
K01574	0.000215051	0.000211986	0.000188464	0.000113382	acetoacetate decarboxylase [EC:4.1.1.4]

K01575	1.1849E-06	8.1377E-07	4.88977E-06	1.18293E-05	acetolactate decarboxylase [EC:4.1.1.5]
K01576	2.56603E-06	3.68179E-06	1.7108E-05	4.21208E-05	benzoylformate decarboxylase [EC:4.1.1.7]
K01577	1.56888E-06	7.6983E-08	2.13908E-06	3.04655E-07	oxalyl-CoA decarboxylase [EC:4.1.1.8]
K01578	2.80313E-06	2.98573E-06	1.62874E-05	3.52377E-05	malonyl-CoA decarboxylase [EC:4.1.1.9]
K01579	0.000220521	0.000222776	0.000214943	0.000210141	aspartate 1-decarboxylase [EC:4.1.1.11]
K01580	4.30737E-07	1.13802E-06	2.07854E-06	2.14936E-05	glutamate decarboxylase [EC:4.1.1.15]
K01581	4.37713E-06	1.46314E-06	3.87834E-06	1.03363E-05	ornithine decarboxylase [EC:4.1.1.17]
K01582	2.33439E-06	1.98555E-06	5.62546E-06	4.88481E-05	lysine decarboxylase [EC:4.1.1.18]
K01583	0	3.98157E-08	2.09314E-08	0	arginine decarboxylase [EC:4.1.1.19]
K01584	0.000217364	0.000218653	0.000214664	0.000220758	arginine decarboxylase [EC:4.1.1.19]
K01585	0.000218875	0.000220138	0.000212058	0.000224601	arginine decarboxylase [EC:4.1.1.19]
K01586	0.000652445	0.000649848	0.000609373	0.000476893	diaminopimelate decarboxylase [EC:4.1.1.20]
K01587	0	9.82517E-09	0	0	phosphoribosylaminoimidazole carboxylase / phosphoribosylaminoimidazole-succinocarboxamide synthase [EC:4.1.1.
K01588	0.000223494	0.000223816	0.000227697	0.000226517	5-(carboxyamino)imidazole ribonucleotide mutase [EC:5.4.99.18]
K01589	0.000222319	0.000222279	0.000222037	0.000225149	5-(carboxyamino)imidazole ribonucleotide synthase [EC:6.3.4.18]
K01590	6.05838E-08	1.12526E-07	1.79022E-07	2.34407E-06	histidine decarboxylase [EC:4.1.1.22]
K01591	0.000223572	0.000223713	0.000227612	0.000227581	orotidine-5-phosphate decarboxylase [EC:4.1.1.23]
K01593	1.33229E-07	3.47833E-07	1.57784E-06	2.22476E-06	aromatic-L-amino-acid/L-tryptophan decarboxylase [EC:4.1.1.28 4.1.1.105]
K01595	0.000220778	0.000218031	0.000209832	0.000223951	phosphoenolpyruvate carboxylase [EC:4.1.1.31]
K01596	0.000216725	0.000219331	0.000207474	0.00015273	phosphoenolpyruvate carboxykinase (GTP) [EC:4.1.1.32]
K01597	1.2938E-06	8.59628E-07	3.55275E-06	3.57059E-06	diphosphomevalonate decarboxylase [EC:4.1.1.33]
K01598	7.18863E-07	7.44702E-07	7.40708E-07	3.52714E-06	phosphopantothenoylcysteine decarboxylase [EC:4.1.1.36]
K01599	0.000222638	0.000222876	0.000226271	0.000225569	uroporphyrinogen decarboxylase [EC:4.1.1.37]
K01601	1.31733E-06	1.54145E-06	6.8586E-06	1.43862E-05	ribulose-bisphosphate carboxylase large chain [EC:4.1.1.39]
K01602	2.43449E-07	8.27298E-08	4.71344E-07	1.33999E-07	ribulose-bisphosphate carboxylase small chain [EC:4.1.1.39]
K01607	0.000867179	0.000859361	0.000799176	0.000657781	4-carboxymuconolactone decarboxylase [EC:4.1.1.44]
K01608	0.000216424	0.00021633	0.000202254	0.000149665	tartronate-semialdehyde synthase [EC:4.1.1.47]
K01609	0.000436837	0.000434625	0.000413141	0.000332762	indole-3-glycerol phosphate synthase [EC:4.1.1.48]
K01610	3.5593E-06	3.32081E-06	8.67251E-06	1.88293E-05	phosphoenolpyruvate carboxykinase (ATP) [EC:4.1.1.49]
K01611	0.000217228	0.000216932	0.000206003	0.00020541	S-adenosylmethionine decarboxylase [EC:4.1.1.50]
K01612	2.84961E-07	1.15257E-07	1.80258E-07	5.50219E-07	vanillate/4-hydroxybenzoate decarboxylase subunit C [EC:4.1.1.- 4.1.1.61]
K01613	0.000434537	0.00043147	0.000398026	0.000336238	phosphatidylserine decarboxylase [EC:4.1.1.65]
K01615	7.43426E-07	4.83663E-07	9.93934E-07	5.92522E-07	glutaconyl-CoA decarboxylase [EC:4.1.1.70]
K01616	2.29023E-06	5.85719E-06	1.2349E-05	5.68128E-05	2-oxoglutarate decarboxylase [EC:4.1.1.71]
K01617	0.00021487	0.000212047	0.000187952	0.000116064	2-oxo-3-hexenedioate decarboxylase [EC:4.1.1.77]
K01619	6.02035E-06	7.24263E-06	3.02692E-05	2.68324E-05	deoxyribose-phosphate aldolase [EC:4.1.2.4]
K01620	0.000434902	0.000432862	0.000402703	0.000285943	threonine aldolase [EC:4.1.2.48]
K01621	2.10727E-06	8.36961E-07	1.73358E-06	5.88586E-06	xylulose-5-phosphate/fructose-6-phosphate phosphoketolase [EC:4.1.2.9 4.1.2.22]
K01622	8.56287E-09	0	7.3146E-08	0	fructose 1,6-bisphosphate aldolase/phosphatase [EC:4.1.2.13 3.1.3.11]
K01623	3.9113E-06	5.18946E-06	1.47176E-05	5.22753E-05	fructose-bisphosphate aldolase, class I [EC:4.1.2.13]
K01624	0.000222912	0.000223527	0.000227305	0.0001806	fructose-bisphosphate aldolase, class II [EC:4.1.2.13]
K01625	0.000221654	0.000219896	0.00023169	0.000299706	2-dehydro-3-deoxyphosphogluconate aldolase / (4S)-4-hydroxy-2-oxoglutarate aldolase [EC:4.1.2.14 4.1.3.42]
K01626	0.000438119	0.000438789	0.000429142	0.000467153	3-deoxy-7-phosphoheptulonate synthase [EC:2.5.1.54]
K01627	0.000220061	0.000219063	0.000211129	0.000220142	2-dehydro-3-deoxyphosphooctonate aldolase (KDO 8-P synthase) [EC:2.5.1.55]
K01628	0.000430349	0.00042508	0.000383473	0.000238547	L-fuculose-phosphate aldolase [EC:4.1.2.17]
K01629	2.94959E-07	8.77318E-07	7.00477E-07	2.53633E-06	rhamnulose-1-phosphate aldolase [EC:4.1.2.19]
K01630	4.54995E-06	8.61634E-06	3.10014E-05	6.59069E-05	2-dehydro-3-deoxyglucarate aldolase [EC:4.1.2.20]
K01631	0.000215665	0.000212288	0.000190195	0.000135933	2-dehydro-3-deoxyphosphogalactonate aldolase [EC:4.1.2.21]
K01633	0.00043616	0.000434823	0.000410214	0.000338085	7,8-dihydroneopterin aldolase/epimerase/oxygenase [EC:4.1.2.25 5.1.99.8 1.13.11.81]
K01634	3.91036E-08	1.70006E-07	7.05693E-07	2.46464E-06	sphinganine-1-phosphate aldolase [EC:4.1.2.27]
K01635	1.07051E-06	2.09856E-07	3.47514E-06	7.0167E-06	tagatose 1,6-diphosphate aldolase [EC:4.1.2.40]
K01637	0.000219561	0.000220814	0.000209133	0.000221601	isocitrate lyase [EC:4.1.3.1]
K01638	0.000220657	0.000220758	0.000210693	0.000229875	malate synthase [EC:2.3.3.9]
K01639	7.50797E-07	1.47205E-06	7.71136E-06	4.89186E-06	N-acetylneuraminate lyase [EC:4.1.3.3]
K01640	0.00022344	0.000228109	0.000257667	0.000319775	hydroxymethylglutaryl-CoA lyase [EC:4.1.3.4]
K01641	1.30628E-06	1.01588E-06	3.92082E-06	3.76479E-06	hydroxymethylglutaryl-CoA synthase [EC:2.3.3.10]
K01643	2.99911E-07	3.1785E-07	7.00872E-07	4.99809E-06	citrate lyase subunit alpha / citrate CoA-transferase [EC:2.8.3.10]
K01644	0.000227799	0.000234101	0.000256229	0.000262214	citrate lyase subunit beta / citryl-CoA lyase [EC:4.1.3.34]
K01646	2.94827E-07	2.97366E-07	5.27582E-07	4.99706E-06	citrate lyase subunit gamma (acyl carrier protein)
K01647	0.000231349	0.000238944	0.0002846	0.000395847	citrate synthase [EC:2.3.3.1]
K01649	0.000441905	0.000442414	0.000446756	0.000416681	2-isopropylmalate synthase [EC:2.3.3.13]
K01652	0.000451229	0.000454743	0.000477109	0.000533804	acetolactate synthase I/II/III large subunit [EC:2.2.1.6]
K01653	0.000222112	0.000222094	0.000225206	0.000173514	acetolactate synthase I/III small subunit [EC:2.2.1.6]
K01654	1.8105E-06	9.40343E-07	6.2001E-06	1.74987E-05	N-acetylneuraminate synthase [EC:2.5.1.56]
K01655	0	0	9.34485E-08	0	homocitrate synthase [EC:2.3.3.14]
K01657	0.000227185	0.000228927	0.000236534	0.000322467	anthranilate synthase component I [EC:4.1.3.27]
K01658	0.000226635	0.000224098	0.000231122	0.00024262	anthranilate synthase component II [EC:4.1.3.27]
K01659	0.000219399	0.000220547	0.00020873	0.000222377	2-methylcitrate synthase [EC:2.3.3.5]
K01661	3.58462E-06	7.4892E-06	2.06649E-05	2.04544E-05	naphthoate synthase [EC:4.1.3.36]
K01662	0.000225498	0.00022593	0.000229733	0.00023506	1-deoxy-D-xylulose-5-phosphate synthase [EC:2.2.1.7]
K01663	2.97877E-09	1.63753E-08	5.33176E-08	6.64933E-08	glutamine amidotransferase / cyclase [EC:2.4.2.- 4.1.3.-]
K01664	0.00022346	0.000220805	0.000222252	0.000223371	para-aminobenzoate synthetase component II [EC:2.6.1.85]
K01665	0.000226158	0.00022764	0.000231589	0.000295566	para-aminobenzoate synthetase component I [EC:2.6.1.85]
K01666	0.000214231	0.000213202	0.000188962	0.000119537	4-hydroxy 2-oxovalerate aldolase [EC:4.1.3.39]
K01667	8.48928E-07	6.29182E-07	8.58984E-06	1.20826E-05	tryptophanase [EC:4.1.99.1]
K01668	6.50873E-07	5.12579E-07	5.74753E-06	3.70786E-06	tyrosine phenol-lyase [EC:4.1.99.2]
K01669	7.40564E-06	7.31821E-06	2.63056E-05	0.000115351	deoxyribodipyrimidine photo-lyase [EC:4.1.99.3]
K01671	1.4079E-08	4.03172E-08	1.04463E-07	4.6176E-07	sulfofructosephosphate aldolase [EC:4.1.2.57]
K01673	0.000654895	0.000652471	0.000611613	0.00049732	carbonic anhydrase [EC:4.2.1.1]
K01674	2.64832E-07	2.08869E-07	1.74086E-06	1.35677E-05	carbonic anhydrase [EC:4.2.1.1]
K01676	0.000218887	0.000218473	0.000211997	0.000220492	fumarate hydratase, class I [EC:4.2.1.2]
K01677	2.09002E-06	4.45091E-06	1.58569E-05	3.69426E-05	fumarate hydratase subunit alpha [EC:4.2.1.2]
K01678	7.57323E-07	1.02517E-06	9.98295E-07	1.72549E-07	fumarate hydratase subunit beta [EC:4.2.1.2]
K01679	0.000226191	0.000227246	0.000250666	0.000264525	fumarate hydratase, class II [EC:4.2.1.2]
K01681	0.00108683	0.001082876	0.001020668	0.000882478	aconitate hydratase [EC:4.2.1.3]
K01682	0.000217548	0.000216911	0.000206992	0.000221133	aconitate hydratase 2 / 2-methylisocitrate dehydratase [EC:4.2.1.3 4.2.1.99]
K01683	9.70647E-07	2.37526E-06	1.10717E-05	2.7686E-05	D-arabinonate dehydratase [EC:4.2.1.5]

K01684	0.000216952	0.000217879	0.000215436	0.000193339	galactonate dehydratase [EC:4.2.1.6]
K01685	0.000219678	0.000219412	0.000223216	0.000190521	altronate hydrolase [EC:4.2.1.7]
K01686	1.83555E-06	2.32768E-06	9.16553E-06	3.83015E-06	mannonate dehydratase [EC:4.2.1.8]
K01687	0.000233878	0.000234186	0.000283428	0.000363029	dihydroxy-acid dehydratase [EC:4.2.1.9]
K01689	0.000224922	0.000227459	0.000242809	0.000260433	enolase [EC:4.2.1.11]
K01690	0.000218412	0.000216864	0.000206746	0.000217968	phosphogluconate dehydratase [EC:4.2.1.12]
K01692	0.002179448	0.002187416	0.002123724	0.001847175	enoyl-CoA hydratase [EC:4.2.1.17]
K01693	0.000223032	0.000222859	0.000226768	0.000222048	imidazoleglycerol-phosphate dehydratase [EC:4.2.1.19]
K01695	0.000223149	0.00022285	0.000226784	0.000227583	tryptophan synthase alpha chain [EC:4.2.1.20]
K01696	0.000224224	0.000225928	0.000242421	0.00025547	tryptophan synthase beta chain [EC:4.2.1.20]
K01697	0.0002244	0.000227721	0.000243932	0.000263768	cystathionine beta-synthase [EC:4.2.1.22]
K01698	0.000222852	0.000223028	0.00022283	0.000266302	porphobilinogen synthase [EC:4.2.1.24]
K01699	1.03604E-07	4.48864E-07	1.04068E-06	1.04636E-06	propanediol dehydratase large subunit [EC:4.2.1.28]
K01703	0.000224814	0.000223244	0.000238313	0.000224907	3-isopropylmalate/(R)-2-methylmalate dehydratase large subunit [EC:4.2.1.33 4.2.1.35]
K01704	0.000223882	0.000222878	0.000228739	0.000224835	3-isopropylmalate/(R)-2-methylmalate dehydratase small subunit [EC:4.2.1.33 4.2.1.35]
K01706	0.000431556	0.000429996	0.000406482	0.000298213	glucarate dehydratase [EC:4.2.1.40]
K01707	0.000217584	0.000215233	0.000204067	0.000150211	5-dehydro-4-deoxyglucarate dehydratase [EC:4.2.1.41]
K01708	0.000218652	0.000217984	0.00021797	0.000183611	galactarate dehydratase [EC:4.2.1.42]
K01709	9.54956E-07	1.7242E-06	4.46493E-06	1.11852E-05	CDP-glucose 4,6-dehydratase [EC:4.2.1.45]
K01710	0.000225328	0.00022916	0.000234807	0.000280716	dTDP-glucose 4,6-dehydratase [EC:4.2.1.46]
K01711	3.99826E-06	3.50781E-06	1.05167E-05	1.70264E-05	GDPmannose 4,6-dehydratase [EC:4.2.1.47]
K01712	0.000219791	0.00022454	0.000217761	0.000220999	urocanate hydratase [EC:4.2.1.49]
K01713	0.000213941	0.000212173	0.000189251	0.000116923	cyclohexadienyl dehydratase [EC:4.2.1.51 4.2.1.91]
K01714	0.000661098	0.000659448	0.000648886	0.000561276	4-hydroxy-tetrahydrodipicolinate synthase [EC:4.3.3.7]
K01715	0.00066753	0.000675212	0.000692866	0.00073868	enoyl-CoA hydratase [EC:4.2.1.17]
K01716	4.23103E-06	5.10339E-06	1.9546E-05	0.000105478	3-hydroxyacyl-[acyl-carrier protein] dehydratase / trans-2-decenoyl-[acyl-carrier protein] isomerase [EC:4.2.1.59 5.3.3
K01719	8.59449E-06	1.17778E-05	3.77861E-05	0.00010796	uroporphyrinogen-III synthase [EC:4.2.1.75]
K01720	0.000216002	0.000216616	0.000189873	0.000158788	2-methylcitrate dehydratase [EC:4.2.1.79]
K01721	1.58375E-06	3.07786E-07	1.55298E-06	1.86952E-06	nitrile hydratase subunit alpha [EC:4.2.1.84]
K01724	0.000219052	0.000221065	0.000208041	0.000219012	4a-hydroxytetrahydrobiopterin dehydratase [EC:4.2.1.96]
K01725	3.13526E-06	1.05744E-06	2.26826E-06	4.23188E-06	cyanate lyase [EC:4.2.1.104]
K01726	2.22622E-06	1.5125E-06	3.90901E-06	5.59418E-05	gamma-carbonic anhydrase [EC:4.2.1.-]
K01727	5.72908E-07	5.17579E-07	5.70263E-06	1.22549E-06	hyaluronate lyase [EC:4.2.2.1]
K01728	4.45001E-07	1.67098E-07	3.08293E-06	1.14747E-06	pectate lyase [EC:4.2.2.2]
K01729	9.29665E-07	6.3805E-07	3.22641E-06	6.76047E-05	poly(beta-D-mannuronate) lyase [EC:4.2.2.3]
K01730	4.38767E-07	4.41388E-07	4.61847E-07	3.8209E-06	oligogalacturonide lyase [EC:4.2.2.6]
K01731	0	0	0	2.84234E-06	pectate disaccharide-lyase [EC:4.2.2.9]
K01732	0	0	0	3.98855E-08	pectin lyase [EC:4.2.2.10]
K01733	0.000224828	0.00022246	0.000231324	0.000230654	threonine synthase [EC:4.2.3.1]
K01734	0.000218961	0.000221055	0.000219519	0.000250297	methylglyoxal synthase [EC:4.2.3.3]
K01735	0.000223513	0.000224008	0.000228059	0.000226463	3-dehydroquinate synthase [EC:4.2.3.4]
K01736	0.000223375	0.000223743	0.000227824	0.000225874	chorismate synthase [EC:4.2.3.5]
K01737	0.000221387	0.000219832	0.000215175	0.00021346	6-pyruvoyltetrahydropterin/6-carboxytetrahydropterin synthase [EC:4.2.3.12 4.1.2.50]
K01738	0.000663991	0.000663244	0.000636594	0.000620134	cysteine synthase A [EC:2.5.1.47]
K01739	0.000662259	0.000668138	0.000623341	0.000574916	cystathionine gamma-synthase [EC:2.5.1.48]
K01740	0.000454099	0.000466258	0.000477575	0.000570372	O-acetylhomoserine (thiol)-lyase [EC:2.5.1.49]
K01744	0.000223118	0.000228294	0.000249785	0.000288242	aspartate ammonia-lyase [EC:4.3.1.1]
K01745	0.00043444	0.000435882	0.000407469	0.000396919	histidine ammonia-lyase [EC:4.3.1.3]
K01746	5.88727E-07	5.17093E-07	7.22492E-07	1.93998E-06	formiminotetrahydrofolate cyclodeaminase [EC:4.3.1.4]
K01749	0.000222417	0.000223194	0.000224607	0.000230086	hydroxymethylbilane synthase [EC:2.5.1.61]
K01750	0.000647715	0.000646242	0.000604691	0.000419672	ornithine cyclodeaminase [EC:4.3.1.12]
K01751	0.00021411	0.000211872	0.000187464	0.000115184	diaminopropionate ammonia-lyase [EC:4.3.1.15]
K01752	0.000439211	0.000433584	0.0004022	0.000323666	L-serine dehydratase [EC:4.3.1.17]
K01753	0.000217126	0.000215548	0.000205572	0.000158995	D-serine dehydratase [EC:4.3.1.18]
K01754	0.000658993	0.000657504	0.000652953	0.000621052	threonine dehydratase [EC:4.3.1.19]
K01755	0.000224507	0.000223205	0.0002344	0.000223907	argininosuccinate lyase [EC:4.3.2.1]
K01756	0.00022471	0.000226864	0.000244337	0.000266035	adenylosuccinate lyase [EC:4.3.2.2]
K01758	1.34071E-05	2.16254E-05	4.46421E-05	0.00014146	cystathionine gamma-lyase [EC:4.4.1.1]
K01759	0.00044135	0.000441733	0.000447033	0.000527764	lactoylglutathione lyase [EC:4.4.1.5]
K01760	0.000239499	0.000251702	0.000310879	0.000497216	cystathionine beta-lyase [EC:4.4.1.8]
K01761	0.00022934	0.000226941	0.000243408	0.000310777	methionine-gamma-lyase [EC:4.4.1.11]
K01766	1.71476E-06	8.9703E-07	4.06607E-06	1.71109E-05	cysteine sulfinate desulfinase [EC:4.4.1.-]
K01768	0.000239337	0.000233991	0.000263173	0.000371993	adenylate cyclase [EC:4.6.1.1]
K01769	3.97169E-09	0	3.43043E-08	1.0175E-08	guanylate cyclase, other [EC:4.6.1.2]
K01770	0.00022195	0.000222888	0.000223907	0.000222622	2-C-methyl-D-erythritol 2,4-cyclodiphosphate synthase [EC:4.6.1.12]
K01771	2.72447E-08	7.1296E-09	7.49316E-08	2.4745E-08	1-phosphatidylinositol phosphodiesterase [EC:4.6.1.13]
K01772	0.000222353	0.000223393	0.000223524	0.000229877	protoporphyrin/coproporphyrin ferrochelatase [EC:4.99.1.1 4.99.1.9]
K01775	0.000226955	0.000226145	0.000236397	0.000253585	alanine racemase [EC:5.1.1.1]
K01776	0.000438946	0.000438273	0.000434427	0.000352228	glutamate racemase [EC:5.1.1.3]
K01777	2.49517E-07	3.9137E-07	7.83555E-07	3.04638E-06	proline racemase [EC:5.1.1.4]
K01778	0.000222634	0.000223789	0.000225202	0.000222695	diaminopimelate epimerase [EC:5.1.1.7]
K01779	0.000219437	0.000219229	0.000224246	0.000250211	aspartate racemase [EC:5.1.1.13]
K01781	5.90209E-08	4.92284E-08	1.93427E-07	8.78124E-09	mandelate racemase [EC:5.1.2.2]
K01782	8.38296E-06	1.06456E-05	1.9698E-05	0.000116073	3-hydroxyacyl-CoA dehydrogenase / enoyl-CoA hydratase / 3-hydroxybutyryl-CoA epimerase [EC:1.1.1.35 4.2.1.17 5
K01783	0.000223996	0.000224439	0.000228783	0.000228368	ribulose-phosphate 3-epimerase [EC:5.1.3.1]
K01784	0.00023461	0.000240427	0.000268559	0.000329759	UDP-glucose 4-epimerase [EC:5.1.3.2]
K01785	0.000435659	0.000431937	0.000398101	0.000267105	aldose 1-epimerase [EC:5.1.3.3]
K01787	3.42888E-07	9.22385E-07	2.88539E-06	2.88987E-08	N-acylglucosamine 2-epimerase [EC:5.1.3.8]
K01788	1.66635E-06	1.65665E-06	1.8567E-05	1.09844E-05	N-acylglucosamine-6-phosphate 2-epimerase [EC:5.1.3.9]
K01790	0.000220818	0.000222286	0.000219557	0.000225987	dTDP-4-dehydrorhamnose 3,5-epimerase [EC:5.1.3.13]
K01791	0.000434422	0.000436753	0.000417213	0.000333575	UDP-N-acetylglucosamine 2-epimerase (non-hydrolysing) [EC:5.1.3.14]
K01792	6.13201E-06	6.12926E-08	2.77427E-05	0.000130735	glucose-6-phosphate 1-epimerase [EC:5.1.3.15]
K01795	2.04795E-08	4.35878E-08	1.27031E-06	1.84069E-07	mannuronan 5-epimerase [EC:5.1.3.37]
K01796	0.000450447	0.000465223	0.000574498	0.000693933	alpha-methylacyl-CoA racemase [EC:5.1.99.4]
K01799	0.000217614	0.000219622	0.000217819	0.000183079	maleate isomerase [EC:5.2.1.1]
K01800	0.000432813	0.000431372	0.000405739	0.000365921	maleylacetoacetate isomerase [EC:5.2.1.2]
K01801	0.000431664	0.000431233	0.000405269	0.000357727	maleylpyruvate isomerase [EC:5.2.1.4]

K01802	0.000447745	0.000449756	0.000450157	0.000503107	peptidylprolyl isomerase [EC:5.2.1.8]
K01803	0.000223903	0.000224603	0.000231403	0.00022943	triosephosphate isomerase (TIM) [EC:5.3.1.1]
K01804	8.50535E-07	8.65883E-07	1.71571E-06	1.31221E-05	L-arabinose isomerase [EC:5.3.1.4]
K01805	9.51965E-07	1.76402E-06	5.4834E-06	2.85072E-05	xylose isomerase [EC:5.3.1.5]
K01807	0.000220809	0.000217729	0.000214798	0.000223239	ribose 5-phosphate isomerase A [EC:5.3.1.6]
K01808	5.34357E-06	1.05656E-05	3.78471E-05	4.37594E-05	ribose 5-phosphate isomerase B [EC:5.3.1.6]
K01809	0.000218921	0.000218882	0.000223856	0.000138098	mannose-6-phosphate isomerase [EC:5.3.1.8]
K01810	0.000223955	0.000226738	0.000243288	0.000263721	glucose-6-phosphate isomerase [EC:5.3.1.9]
K01811	0.00021497	0.000214869	0.000191408	0.000117445	alpha-D-xyloside xylohydrolase [EC:3.2.1.177]
K01812	1.5312E-06	1.76534E-06	8.9895E-06	7.43281E-06	glucuronate isomerase [EC:5.3.1.12]
K01813	3.24406E-07	1.05729E-06	5.26967E-07	2.52514E-06	L-rhamnose isomerase [EC:5.3.1.14]
K01814	0.000226386	0.000227185	0.00023446	0.000232339	phosphoribosylformimino-5-aminoimidazole carboxamide ribotide isomerase [EC:5.3.1.16]
K01815	1.53042E-06	2.06397E-06	3.90473E-06	2.74669E-05	4-deoxy-L-threo-5-hexosulose-uronate ketol-isomerase [EC:5.3.1.17]
K01816	0.000433662	0.000431987	0.000407275	0.000356315	hydroxypyruvate isomerase [EC:5.3.1.22]
K01817	0.000223087	0.000222708	0.000231267	0.00022114	phosphoribosylanthranilate isomerase [EC:5.3.1.24]
K01818	1.19959E-06	1.39066E-06	9.97084E-06	5.4287E-07	L-fucose/D-arabinose isomerase [EC:5.3.1.25 5.3.1.3]
K01819	2.4373E-06	5.07707E-07	6.61545E-06	1.4312E-05	galactose-6-phosphate isomerase [EC:5.3.1.26]
K01820	1.67296E-07	2.39459E-07	7.71443E-07	2.20794E-06	L-rhamnose isomerase / sugar isomerase [EC:5.3.1.14 5.3.1.-]
K01821	0.000645305	0.000639923	0.000586869	0.000386434	4-oxalocrotonate tautomerase [EC:5.3.2.6]
K01822	5.56472E-07	1.77652E-06	5.97271E-06	1.94551E-05	steroid Delta-isomerase [EC:5.3.3.1]
K01823	3.56664E-06	5.99861E-06	1.2319E-05	6.9951E-06	isopentenyl-diphosphate Delta-isomerase [EC:5.3.3.2]
K01825	4.83016E-06	3.52269E-06	1.11478E-05	8.59148E-05	3-hydroxyacyl-CoA dehydrogenase / enoyl-CoA hydratase / 3-hydroxybutyryl-CoA epimerase / enoyl-CoA isomerase
K01826	0.000217874	0.000218248	0.000219585	0.000197536	5-carboxymethyl-2-hydroxymuconate isomerase [EC:5.3.3.10]
K01829	2.62203E-06	3.78155E-06	4.12309E-06	8.52637E-05	protein disulfide-isomerase [EC:5.3.4.1]
K01834	0.000224186	0.000222132	0.000238564	0.000209187	2,3-bisphosphoglycerate-dependent phosphoglycerate mutase [EC:5.4.2.11]
K01835	7.45693E-06	1.1069E-05	2.65137E-05	4.56574E-05	phosphoglucomutase [EC:5.4.2.2]
K01838	5.50285E-06	9.15488E-06	1.58598E-05	5.83761E-05	beta-phosphoglucomutase [EC:5.4.2.6]
K01839	2.89848E-06	1.08711E-06	7.81171E-06	2.20873E-05	phosphopentomutase [EC:5.4.2.7]
K01840	0.000438677	0.000442554	0.000422288	0.000353898	phosphomannomutase [EC:5.4.2.8]
K01841	1.42707E-07	1.98949E-07	1.67949E-06	2.68681E-07	phosphoenolpyruvate phosphomutase [EC:5.4.2.9]
K01843	0.000217937	0.000213966	0.00019119	0.000164973	lysine 2,3-aminomutase [EC:5.4.3.2]
K01844	0	4.19867E-08	5.58662E-07	7.81044E-08	beta-lysine 5,6-aminomutase alpha subunit [EC:5.4.3.3]
K01845	0.000869648	0.000868176	0.000803779	0.000634446	glutamate-1-semialdehyde 2,1-aminomutase [EC:5.4.3.8]
K01846	9.91187E-08	7.42606E-08	2.50845E-07	5.90615E-08	methylaspartate mutase sigma subunit [EC:5.4.99.1]
K01847	5.82107E-06	8.83853E-06	3.80479E-05	3.98423E-05	methylmalonyl-CoA mutase [EC:5.4.99.2]
K01848	3.34464E-06	5.46145E-06	2.59759E-05	3.78165E-05	methylmalonyl-CoA mutase, N-terminal domain [EC:5.4.99.2]
K01849	1.0331E-07	9.38463E-07	1.36321E-06	4.96206E-07	methylmalonyl-CoA mutase, C-terminal domain [EC:5.4.99.2]
K01851	6.28264E-09	2.27009E-07	6.40391E-07	1.80181E-07	salicylate biosynthesis isochorismate synthase [EC:5.4.4.2]
K01852	0	0	1.04657E-08	0	lanosterol synthase [EC:5.4.99.7]
K01854	2.32786E-06	4.01883E-06	1.26261E-05	2.51573E-05	UDP-galactopyranose mutase [EC:5.4.99.9]
K01856	1.52435E-06	3.97822E-06	2.00065E-05	4.51418E-05	muconate cycloisomerase [EC:5.5.1.1]
K01857	0.000217282	0.000218499	0.00022383	0.000210226	3-carboxy-cis,cis-muconate cycloisomerase [EC:5.5.1.2]
K01858	2.40686E-06	4.042E-06	4.80572E-06	2.05305E-06	myo-inositol-1-phosphate synthase [EC:5.5.1.4]
K01860	3.30974E-09	0	0	0	chloromuconate cycloisomerase [EC:5.5.1.7]
K01865	7.9677E-09	0	0	1.15405E-08	(hydroxyamino)benzene mutase [EC:5.4.4.1]
K01866	0.000223856	0.000224296	0.000229156	0.000236349	tyrosyl-tRNA synthetase [EC:6.1.1.1]
K01867	0.00022412	0.000224406	0.00022954	0.000230729	tryptophanyl-tRNA synthetase [EC:6.1.1.2]
K01868	0.000223584	0.000224022	0.000228448	0.000227188	threonyl-tRNA synthetase [EC:6.1.1.3]
K01869	0.000223798	0.000224269	0.000229011	0.00022564	leucyl-tRNA synthetase [EC:6.1.1.4]
K01870	0.000224695	0.000224157	0.000232754	0.000225984	isoleucyl-tRNA synthetase [EC:6.1.1.5]
K01872	0.000439492	0.000438876	0.000425844	0.000372878	alanyl-tRNA synthetase [EC:6.1.1.7]
K01873	0.000438136	0.000436346	0.00041597	0.000340596	valyl-tRNA synthetase [EC:6.1.1.9]
K01874	0.000438164	0.000436925	0.000418169	0.000344533	methionyl-tRNA synthetase [EC:6.1.1.10]
K01875	0.000223495	0.000223753	0.000227553	0.000226656	seryl-tRNA synthetase [EC:6.1.1.11]
K01876	0.000224646	0.000227738	0.000233256	0.000225876	aspartyl-tRNA synthetase [EC:6.1.1.12]
K01878	0.00021978	0.000217217	0.000209277	0.000220953	glycyl-tRNA synthetase alpha chain [EC:6.1.1.14]
K01879	0.000219805	0.000217237	0.000209586	0.000223414	glycyl-tRNA synthetase beta chain [EC:6.1.1.14]
K01880	3.71195E-06	6.53027E-06	1.93535E-05	4.50974E-06	glycyl-tRNA synthetase [EC:6.1.1.14]
K01881	0.000223511	0.000223777	0.000228577	0.000225474	prolyl-tRNA synthetase [EC:6.1.1.15]
K01883	0.000225132	0.00022685	0.000237409	0.000229739	cysteinyl-tRNA synthetase [EC:6.1.1.16]
K01884	0.000213778	0.000211793	0.000186388	0.000113218	cysteinyl-tRNA synthetase, unknown class [EC:6.1.1.16]
K01885	0.000227607	0.000228362	0.000243706	0.000233434	glutamyl-tRNA synthetase [EC:6.1.1.17]
K01886	0.000219771	0.000219362	0.000216779	0.000217633	glutaminyl-tRNA synthetase [EC:6.1.1.18]
K01887	0.000223549	0.000223849	0.000228841	0.000232837	arginyl-tRNA synthetase [EC:6.1.1.19]
K01889	0.000223495	0.000223749	0.000228321	0.000226098	phenylalanyl-tRNA synthetase alpha chain [EC:6.1.1.20]
K01890	0.000224596	0.000223923	0.000231954	0.000228809	phenylalanyl-tRNA synthetase beta chain [EC:6.1.1.20]
K01892	0.000223583	0.000223841	0.000228766	0.000225797	histidyl-tRNA synthetase [EC:6.1.1.21]
K01893	3.74468E-06	4.21708E-06	1.27545E-05	7.23463E-05	asparaginyl-tRNA synthetase [EC:6.1.1.22]
K01894	0.000221188	0.00021823	0.000218773	0.000247895	glutamyl-Q tRNA(Asp) synthetase [EC:6.1.1.-]
K01895	0.000885425	0.000887758	0.000876258	0.000815952	acetyl-CoA synthetase [EC:6.2.1.1]
K01896	8.65702E-07	2.76853E-06	5.4122E-07	0	medium-chain acyl-CoA synthetase [EC:6.2.1.2]
K01897	0.000909731	0.000939167	0.001119887	0.001354474	long-chain acyl-CoA synthetase [EC:6.2.1.3]
K01902	0.000223695	0.000222373	0.000220265	0.000221895	succinyl-CoA synthetase alpha subunit [EC:6.2.1.5]
K01903	0.000223718	0.00022236	0.000220078	0.000221916	succinyl-CoA synthetase beta subunit [EC:6.2.1.5]
K01904	3.50005E-08	1.34585E-07	7.11462E-07	1.81761E-06	4-coumarate--CoA ligase [EC:6.2.1.12]
K01905	8.27236E-06	1.72261E-05	6.62826E-05	0.000170572	acetate---CoA ligase (ADP-forming) subunit alpha [EC:6.2.1.13]
K01906	5.19908E-07	3.58428E-07	7.03966E-06	6.26308E-08	6-carboxyhexanoate--CoA ligase [EC:6.2.1.14]
K01907	3.59991E-06	8.95911E-06	4.56725E-05	0.000109717	acetoacetyl-CoA synthetase [EC:6.2.1.16]
K01908	0.000443254	0.000441392	0.000437482	0.000461164	propionyl-CoA synthetase [EC:6.2.1.17]
K01909	1.206E-06	3.18982E-06	1.42338E-05	3.46999E-05	long-chain-fatty-acid--[acyl-carrier-protein] ligase [EC:6.2.1.20]
K01910	2.97151E-07	3.06709E-07	3.8256E-07	5.05898E-06	[citrate (pro-3S)-lyase] ligase [EC:6.2.1.22]
K01911	9.52883E-06	1.64342E-05	6.27253E-05	0.000175507	O-succinylbenzoic acid---CoA ligase [EC:6.2.1.26]
K01912	0.000220849	0.000228506	0.000240653	0.000242281	phenylacetate-CoA ligase [EC:6.2.1.30]
K01914	1.26341E-06	1.50102E-06	6.53625E-06	1.02715E-05	aspartate--ammonia ligase [EC:6.3.1.1]
K01915	0.000443921	0.000446245	0.000440883	0.000410382	glutamine synthetase [EC:6.3.1.2]
K01916	0.000222781	0.000228088	0.000232772	0.000262534	NAD+ synthase [EC:6.3.1.5]
K01918	0.000221882	0.000222823	0.000219787	0.000221881	pantoate--beta-alanine ligase [EC:6.3.2.1]

K01919	0.000434889	0.000432197	0.000414106	0.000370829	glutamate--cysteine ligase [EC:6.3.2.2]
K01920	0.000219348	0.000218127	0.00020782	0.000219496	glutathione synthase [EC:6.3.2.3]
K01921	0.000225726	0.000225515	0.000237991	0.000282888	D-alanine-D-alanine ligase [EC:6.3.2.4]
K01923	0.000224515	0.000223781	0.000227795	0.00022541	phosphoribosylaminoimidazole-succinocarboxamide synthase [EC:6.3.2.6]
K01924	0.000227205	0.000227216	0.000236433	0.000324803	UDP-N-acetylmuramate--alanine ligase [EC:6.3.2.8]
K01925	0.000225605	0.000225538	0.000241963	0.000272499	UDP-N-acetylmuramoylalanine--D-glutamate ligase [EC:6.3.2.9]
K01926	2.42066E-06	1.70095E-06	1.0574E-05	6.2535E-06	redox-sensing transcriptional repressor
K01928	0.000226158	0.000225651	0.000240314	0.000233826	UDP-N-acetylmuramoyl-L-alanyl-D-glutamate--2,6-diaminopimelate ligase [EC:6.3.2.13]
K01929	0.000439827	0.000438407	0.000421362	0.00039336	UDP-N-acetylmuramoyl-tripeptide--D-alanyl-D-alanine ligase [EC:6.3.2.10]
K01932	1.24618E-06	1.80759E-07	3.11756E-06	6.62022E-08	gamma-polyglutamate synthase [EC:6.3.2.-]
K01933	0.000223496	0.000223735	0.000227885	0.000225529	phosphoribosylformylglycinamidine cyclo-ligase [EC:6.3.3.1]
K01934	0.000223513	0.000223796	0.00022792	0.000225599	5-formyltetrahydrofolate cyclo-ligase [EC:6.3.3.2]
K01935	0.00021994	0.000220774	0.000218212	0.00022008	dethiobiotin synthetase [EC:6.3.3.3]
K01937	0.000223768	0.00022372	0.000227707	0.000227051	CTP synthase [EC:6.3.4.2]
K01938	5.28624E-06	3.28306E-06	1.69541E-05	1.87727E-05	formate--tetrahydrofolate ligase [EC:6.3.4.3]
K01939	0.000223896	0.000224729	0.000231359	0.000237527	adenylosuccinate synthase [EC:6.3.4.4]
K01940	0.000223529	0.000223238	0.000227918	0.000222986	argininosuccinate synthase [EC:6.3.4.5]
K01941	0.000659897	0.000671717	0.0006847	0.000658206	urea carboxylase [EC:6.3.4.6]
K01945	0.000223574	0.000223749	0.000233261	0.000225851	phosphoribosylamine---glycine ligase [EC:6.3.4.13]
K01947	4.04966E-08	0	8.72143E-09	1.07555E-07	biotin---[acetyl-CoA-carboxylase] ligase / type III pantothenate kinase [EC:6.3.4.15 2.7.1.33]
K01949	4.34659E-07	4.10737E-08	5.72521E-08	5.06588E-08	glutamate---methylamine ligase [EC:6.3.4.12]
K01950	0.000222715	0.000221977	0.000241423	0.000246547	NAD+ synthase (glutamine-hydrolysing) [EC:6.3.5.1]
K01951	0.000447661	0.000453519	0.000462746	0.000528087	GMP synthase (glutamine-hydrolysing) [EC:6.3.5.2]
K01952	0.000232624	0.000233344	0.000257188	0.000236607	phosphoribosylformylglycinamidine synthase [EC:6.3.5.3]
K01953	1.00254E-05	1.19619E-05	3.11858E-05	0.000174989	asparagine synthase (glutamine-hydrolysing) [EC:6.3.5.4]
K01955	0.00022495	0.000226183	0.000232471	0.000228913	carbamoyl-phosphate synthase large subunit [EC:6.3.5.5]
K01956	0.000223998	0.000224204	0.000229237	0.000225642	carbamoyl-phosphate synthase small subunit [EC:6.3.5.5]
K01958	0.000665731	0.000676833	0.000702292	0.000709904	pyruvate carboxylase [EC:6.4.1.1]
K01959	0.000227151	0.000237157	0.000272934	0.000289012	pyruvate carboxylase subunit A [EC:6.4.1.1]
K01960	2.7991E-06	3.86354E-06	1.79442E-05	2.16473E-05	pyruvate carboxylase subunit B [EC:6.4.1.1]
K01961	0.000668271	0.000679508	0.000706861	0.000740142	acetyl-CoA carboxylase, biotin carboxylase subunit [EC:6.4.1.2 6.3.4.14]
K01962	0.00022408	0.000220833	0.000219194	0.000280821	acetyl-CoA carboxylase carboxyl transferase subunit alpha [EC:6.4.1.2 2.1.3.15]
K01963	0.000224047	0.000221111	0.000219076	0.000286953	acetyl-CoA carboxylase carboxyl transferase subunit beta [EC:6.4.1.2 2.1.3.15]
K01964	5.68427E-09	5.04174E-07	7.53796E-07	1.72088E-06	acetyl-CoA/propionyl-CoA carboxylase [EC:6.4.1.2 6.4.1.3]
K01965	0.000446249	0.000457693	0.000483617	0.000501686	propionyl-CoA carboxylase alpha chain [EC:6.4.1.3]
K01966	7.40048E-06	1.7252E-05	5.68945E-05	7.93628E-05	propionyl-CoA carboxylase beta chain [EC:6.4.1.3 2.1.3.15]
K01968	0.00066417	0.000673324	0.000691068	0.000680603	3-methylcrotonyl-CoA carboxylase alpha subunit [EC:6.4.1.4]
K01969	0.000221322	0.000225082	0.000237074	0.00025995	3-methylcrotonyl-CoA carboxylase beta subunit [EC:6.4.1.4]
K01971	7.12935E-06	1.13538E-05	3.14455E-05	0.000146949	bifunctional non-homologous end joining protein LigD [EC:6.5.1.1]
K01972	0.000224179	0.000224636	0.000231625	0.000226664	DNA ligase (NAD+) [EC:6.5.1.2]
K01974	0.00021531	0.000214962	0.000202573	0.000168703	RNA 3-terminal phosphate cyclase (ATP) [EC:6.5.1.4]
K01975	0.000429961	0.000425711	0.00038023	0.000235172	RNA 2,3-cyclic 3-phosphodiesterase [EC:3.1.4.58]
K01989	0.000222175	0.000220384	0.00021666	0.000171185	putative ABC transport system substrate-binding protein
K01990	0.003359382	0.003384115	0.003608727	0.003584883	ABC-2 type transport system ATP-binding protein
K01991	0.000223638	0.000218329	0.000196207	0.000144638	polysaccharide biosynthesis/export protein
K01992	0.000698746	0.000706705	0.000824386	0.001191801	ABC-2 type transport system permease protein
K01993	0.000222465	0.000224548	0.000225071	0.000299624	HlyD family secretion protein
K01994	6.48858E-08	2.06813E-07	6.02021E-07	5.28038E-08	LuxR family transcriptional regulator, transcriptional regulator of spore coat protein
K01995	0.003734923	0.003722469	0.003740901	0.003229457	branched-chain amino acid transport system ATP-binding protein
K01996	0.002865596	0.002859406	0.002892946	0.002506818	branched-chain amino acid transport system ATP-binding protein
K01997	0.001751643	0.001749987	0.001759859	0.00150417	branched-chain amino acid transport system permease protein
K01998	0.002399482	0.002387849	0.002334618	0.0018649	branched-chain amino acid transport system permease protein
K01999	0.002619196	0.002614942	0.002586772	0.002138906	branched-chain amino acid transport system substrate-binding protein
K02000	2.26253E-05	2.78375E-05	0.000101096	0.000352259	glycine betaine/proline transport system ATP-binding protein [EC:3.6.3.32]
K02001	4.58647E-06	6.42285E-06	3.5637E-05	6.2859E-05	glycine betaine/proline transport system permease protein
K02002	5.81616E-06	5.74348E-06	4.46499E-05	6.44697E-05	glycine betaine/proline transport system substrate-binding protein
K02003	0.004244404	0.004263975	0.004563207	0.004637638	putative ABC transport system ATP-binding protein
K02004	0.000471312	0.000485441	0.000559979	0.001007532	putative ABC transport system permease protein
K02005	8.70709E-06	1.308E-05	2.78272E-05	0.000129332	HlyD family secretion protein
K02006	0.000442831	0.000444452	0.00044609	0.000406875	cobalt/nickel transport system ATP-binding protein
K02007	1.01068E-06	1.24482E-06	9.96003E-06	2.29382E-06	cobalt/nickel transport system permease protein
K02008	7.94899E-07	1.21415E-06	9.70311E-06	2.27065E-06	cobalt/nickel transport system permease protein
K02009	8.54373E-07	7.00904E-07	7.893E-06	2.11308E-06	cobalt/nickel transport protein
K02010	0.002001894	0.002014318	0.002091357	0.002078612	iron(III) transport system ATP-binding protein [EC:3.6.3.30]
K02011	0.000223537	0.000230486	0.000248021	0.000259346	iron(III) transport system permease protein
K02012	0.00043652	0.00044141	0.000416617	0.000390409	iron(III) transport system substrate-binding protein
K02013	0.001800697	0.001817322	0.001948723	0.001940333	iron complex transport system ATP-binding protein [EC:3.6.3.34]
K02014	0.002243911	0.002242713	0.00239632	0.004112816	iron complex outermembrane recepter protein
K02015	0.000255809	0.000274488	0.000443046	0.000679888	iron complex transport system permease protein
K02016	0.000465323	0.000476172	0.000617857	0.00078319	iron complex transport system substrate-binding protein
K02017	0.001548532	0.001550129	0.001579492	0.001437775	molybdate transport system ATP-binding protein [EC:3.6.3.29]
K02018	0.000654836	0.000652998	0.00063213	0.000519931	molybdate transport system permease protein
K02019	0.000221439	0.000223088	0.000238323	0.000254941	molybdate transport system regulatory protein
K02020	0.000222994	0.000222021	0.000234299	0.000246653	molybdate transport system substrate-binding protein
K02021	3.89042E-06	1.49469E-06	7.55211E-06	4.02772E-06	putative ABC transport system ATP-binding protein
K02022	1.7682E-06	1.27888E-06	5.6406E-06	1.3714E-05	HlyD family secretion protein
K02024	0.000214341	0.000213021	0.000188144	0.000135746	maltoporin
K02025	0.000230597	0.000232331	0.000305829	0.000278576	multiple sugar transport system permease protein
K02026	1.99121E-05	2.65097E-05	0.000132336	0.000188903	multiple sugar transport system permease protein
K02027	1.11627E-05	1.19317E-05	6.44312E-05	7.94895E-05	multiple sugar transport system substrate-binding protein
K02028	0.002676562	0.00268117	0.002809485	0.002863636	polar amino acid transport system ATP-binding protein [EC:3.6.3.21]
K02029	0.003051019	0.003034489	0.002890246	0.002282313	polar amino acid transport system permease protein
K02030	0.001750234	0.001749199	0.001718569	0.00156762	polar amino acid transport system substrate-binding protein
K02031	0.003793719	0.00379025	0.003941447	0.003607741	peptide/nickel transport system ATP-binding protein
K02032	0.003773708	0.003768831	0.003842242	0.003404495	peptide/nickel transport system ATP-binding protein
K02033	0.001101595	0.001093249	0.00108897	0.000836066	peptide/nickel transport system permease protein
K02034	0.001540779	0.001525413	0.001486697	0.001127401	peptide/nickel transport system permease protein

K02035	0.001538936	0.00152328	0.001473449	0.001133448	peptide/nickel transport system substrate-binding protein
K02036	0.000671225	0.000678926	0.000721596	0.000758927	phosphate transport system ATP-binding protein [EC:3.6.3.27]
K02037	0.000226578	0.000225915	0.000248782	0.000257736	phosphate transport system permease protein
K02038	0.000227053	0.000227908	0.000235284	0.000246571	phosphate transport system permease protein
K02039	0.000224759	0.000223863	0.000222663	0.000229593	phosphate transport system protein
K02040	0.000443491	0.000440005	0.00042233	0.000422251	phosphate transport system substrate-binding protein
K02041	0.001106459	0.001119041	0.001133989	0.001158305	phosphonate transport system ATP-binding protein [EC:3.6.3.28]
K02042	1.16677E-05	9.02728E-06	4.48331E-05	8.29222E-05	phosphonate transport system permease protein
K02043	2.38643E-06	3.66128E-06	1.61444E-05	3.75721E-05	GntR family transcriptional regulator, phosphonate transport system regulatory protein
K02044	1.21259E-05	1.28683E-05	5.96513E-05	0.000163463	phosphonate transport system substrate-binding protein
K02045	0.001313987	0.001313286	0.001303069	0.001141451	sulfate transport system ATP-binding protein [EC:3.6.3.25]
K02046	0.000219681	0.000217216	0.000208564	0.000161122	sulfate transport system permease protein
K02047	0.000218917	0.000217232	0.000213254	0.000171353	sulfate transport system permease protein
K02048	0.000221136	0.000219262	0.000224661	0.00019868	sulfate transport system substrate-binding protein
K02049	0.00331703	0.003324323	0.003387759	0.003176562	NitT/TauT family transport system ATP-binding protein
K02050	0.000663643	0.00066746	0.000680631	0.000618216	NitT/TauT family transport system permease protein
K02051	0.000878193	0.00087724	0.000877582	0.000763791	NitT/TauT family transport system substrate-binding protein
K02052	0.00113223	0.001144479	0.001271279	0.001502402	putative spermidine/putrescine transport system ATP-binding protein
K02053	0.000652645	0.000652288	0.000621021	0.000466097	putative spermidine/putrescine transport system permease protein
K02054	0.000220355	0.00021695	0.000204869	0.000136819	putative spermidine/putrescine transport system permease protein
K02055	0.000434765	0.000432921	0.000416303	0.000319763	putative spermidine/putrescine transport system substrate-binding protein
K02056	0.001314367	0.001311788	0.001286514	0.001101439	simple sugar transport system ATP-binding protein [EC:3.6.3.17]
K02057	0.000447773	0.000456128	0.000494719	0.000471229	simple sugar transport system permease protein
K02058	0.000220306	0.000227118	0.000259165	0.000265206	simple sugar transport system substrate-binding protein
K02062	0.000232492	0.000244074	0.00030629	0.000463583	thiamine transport system ATP-binding protein
K02063	2.32591E-06	4.23832E-06	3.23901E-06	1.48258E-05	thiamine transport system permease protein
K02064	1.12476E-06	3.51008E-06	1.33661E-06	1.30283E-05	thiamine transport system substrate-binding protein
K02065	0.00045734	0.000464334	0.00052138	0.000678885	phospholipid/cholesterol/gamma-HCH transport system ATP-binding protein
K02066	0.000436692	0.000440318	0.000436532	0.000480854	phospholipid/cholesterol/gamma-HCH transport system permease protein
K02067	0.000436414	0.000444349	0.000438702	0.000514896	phospholipid/cholesterol/gamma-HCH transport system substrate-binding protein
K02068	0.000224714	0.000223907	0.000202749	0.000155396	putative ABC transport system ATP-binding protein
K02069	0.000217109	0.000213412	0.000197471	0.000121069	putative ABC transport system permease protein
K02071	0.001761406	0.001759871	0.001782615	0.001613872	D-methionine transport system ATP-binding protein
K02072	0.000228821	0.000231014	0.000272429	0.000331622	D-methionine transport system permease protein
K02073	0.000449278	0.000456501	0.000516901	0.000593756	D-methionine transport system substrate-binding protein
K02074	0.000226199	0.000229139	0.000251999	0.000193059	zinc/manganese transport system ATP-binding protein
K02075	5.89357E-06	4.81458E-06	2.21311E-05	7.19773E-06	zinc/manganese transport system permease protein
K02076	1.1299E-06	2.63795E-07	3.56241E-06	7.09315E-07	Fur family transcriptional regulator, zinc uptake regulator
K02077	5.13497E-06	5.53443E-06	1.41492E-05	6.9604E-06	zinc/manganese transport system substrate-binding protein
K02078	0.001084214	0.0010796	0.001002402	0.000834639	acyl carrier protein
K02079	2.22946E-08	9.81061E-08	6.62975E-07	3.06802E-06	N-acetylgalactosamine-6-phosphate deacetylase [EC:3.5.1.25]
K02080	4.44141E-09	5.12806E-08	2.32256E-07	1.97799E-07	galactosamine-6-phosphate isomerase [EC:5.3.1.-]
K02081	4.04622E-06	6.70148E-06	3.37205E-05	0.000117881	DeoR family transcriptional regulator, aga operon transcriptional repressor
K02082	1.2021E-06	1.03254E-06	2.41462E-06	5.78744E-05	tagatose-6-phosphate ketose/aldose isomerase [EC:5.-.-.-]
K02083	4.76919E-06	2.40469E-06	1.24527E-05	2.78407E-05	allantoate deiminase [EC:3.5.3.9]
K02086	1.30797E-06	3.44262E-07	4.04484E-06	3.93598E-06	DNA replication protein
K02099	7.55243E-07	7.94734E-07	6.41215E-07	1.12974E-05	AraC family transcriptional regulator, arabinose operon regulatory protein
K02100	2.67773E-06	5.31755E-06	7.47842E-06	5.68831E-05	MFS transporter, SP family, arabinose:H+ symporter
K02101	0	9.5208E-10	2.9902E-09	0	arabinose operon protein AraL
K02103	0.000215304	0.000214907	0.000200999	0.000153177	GntR family transcriptional regulator, arabinose operon transcriptional repressor
K02106	2.9273E-06	6.99253E-06	6.49972E-06	5.69091E-05	short-chain fatty acids transporter
K02107	2.21445E-08	0	3.75833E-07	2.68018E-06	V/A-type H+/Na+-transporting ATPase subunit G/H
K02108	0.000437225	0.000435898	0.000413322	0.000341793	F-type H+-transporting ATPase subunit a
K02109	0.000225186	0.000224392	0.000228066	0.000228947	F-type H+-transporting ATPase subunit b
K02110	0.000223487	0.000224058	0.000227467	0.000228653	F-type H+-transporting ATPase subunit c
K02111	0.000226236	0.00022623	0.000237607	0.000287697	F-type H+-transporting ATPase subunit alpha [EC:3.6.3.14]
K02112	0.000223546	0.000224257	0.000229328	0.000228992	F-type H+-transporting ATPase subunit beta [EC:3.6.3.14]
K02113	0.000223081	0.000223756	0.000226947	0.000228268	F-type H+-transporting ATPase subunit delta
K02114	0.000437409	0.000435927	0.000415336	0.000351232	F-type H+-transporting ATPase subunit epsilon
K02115	0.000223516	0.000224125	0.00022722	0.000228578	F-type H+-transporting ATPase subunit gamma
K02116	0.000219426	0.000216193	0.000209625	0.000166478	ATP synthase protein I
K02117	9.77183E-07	1.07018E-06	2.60267E-06	2.83743E-06	V/A-type H+/Na+-transporting ATPase subunit A [EC:3.6.3.14 3.6.3.15]
K02118	0.000214437	0.000212778	0.000188367	0.000116037	V/A-type H+/Na+-transporting ATPase subunit B
K02119	4.8482E-07	1.93776E-07	1.42266E-06	2.82112E-06	V/A-type H+/Na+-transporting ATPase subunit C
K02120	6.55223E-07	9.37724E-07	2.25092E-06	2.83568E-06	V/A-type H+/Na+-transporting ATPase subunit D
K02121	6.55223E-07	8.383E-07	1.92256E-06	2.77566E-06	V/A-type H+/Na+-transporting ATPase subunit E
K02122	4.8482E-07	2.32431E-07	1.4155E-06	2.82112E-06	V/A-type H+/Na+-transporting ATPase subunit F
K02123	6.55223E-07	9.37724E-07	2.24853E-06	2.83469E-06	V/A-type H+/Na+-transporting ATPase subunit I
K02124	6.55223E-07	9.37724E-07	2.24853E-06	2.83363E-06	V/A-type H+/Na+-transporting ATPase subunit K
K02160	0.000652732	0.000649701	0.000612852	0.000505543	acetyl-CoA carboxylase biotin carboxyl carrier protein
K02164	2.58348E-08	8.25818E-07	4.46038E-07	1.04762E-07	nitric oxide reductase NorE protein
K02167	1.13069E-06	1.23934E-06	2.79754E-06	6.17485E-05	TetR/AcrR family transcriptional regulator, transcriptional repressor of bet genes
K02168	1.218E-05	1.90979E-05	4.02963E-05	0.000229018	choline/glycine/proline betaine transport protein
K02169	0.000221306	0.000223716	0.00023027	0.000276558	malonyl-CoA O-methyltransferase [EC:2.1.1.197]
K02170	1.63606E-06	2.49993E-06	4.54627E-06	7.37396E-05	pimeloyl-[acyl-carrier protein] methyl ester esterase [EC:3.1.1.85]
K02171	2.80993E-06	1.35427E-06	7.18308E-06	7.99776E-06	BlaI family transcriptional regulator, penicillinase repressor
K02172	2.37951E-06	8.6084E-07	6.97766E-06	1.19309E-06	bla regulator protein blaR1
K02173	4.39441E-09	3.88613E-08	1.55259E-07	4.46146E-07	putative kinase
K02182	7.85551E-07	8.97067E-07	4.85508E-06	1.03531E-05	carnitine-CoA ligase [EC:6.2.1.48]
K02188	0.000214623	0.000212708	0.000189753	0.000114984	cobalt-precorrin-5B (C1)-methyltransferase [EC:2.1.1.195]
K02189	0.000217366	0.00021604	0.000213023	0.000151695	cobalt-precorrin 5A hydrolase [EC:3.7.1.12]
K02190	7.50916E-07	8.88711E-07	1.23561E-06	1.1858E-06	sirohydrochlorin cobaltochelatase [EC:4.99.1.3]
K02191	5.41075E-07	8.07765E-07	5.08091E-07	1.15127E-06	cobalt-precorrin-6B (C15)-methyltransferase [EC:2.1.1.196]
K02192	0.000217691	0.000216992	0.000207025	0.000212218	bacterioferritin-associated ferredoxin
K02193	1.43958E-05	1.65889E-05	3.9819E-05	9.04499E-05	heme exporter protein A [EC:3.6.3.41]
K02194	4.2696E-06	2.34033E-06	5.76499E-06	6.94482E-05	heme exporter protein B
K02195	4.27657E-06	2.72865E-06	6.3749E-06	7.34345E-05	heme exporter protein C

K02196	3.7986E-06	1.60863E-06	5.03643E-06	5.81751E-05	heme exporter protein D
K02197	4.28199E-06	2.34976E-06	6.26837E-06	6.96376E-05	cytochrome c-type biogenesis protein CcmE
K02198	4.32729E-06	2.37833E-06	7.12411E-06	7.53912E-05	cytochrome c-type biogenesis protein CcmF
K02199	0.00022577	0.000226567	0.000232007	0.000336829	cytochrome c biogenesis protein CcmG, thiol:disulfide interchange protein DsbE
K02200	8.56269E-06	5.05365E-06	1.26193E-05	0.000133313	cytochrome c-type biogenesis protein CcmH
K02203	7.77786E-08	5.447E-07	1.39841E-06	2.20001E-07	phosphoserine / homoserine phosphotransferase [EC:3.1.3.3 2.7.1.39]
K02204	0.000218195	0.000215745	0.00020613	0.000149092	homoserine kinase type II [EC:2.7.1.39]
K02205	4.81136E-06	9.79887E-06	4.81245E-05	0.000106655	arginine/ornithine permease
K02217	4.52833E-06	5.56072E-06	1.47452E-05	1.43034E-05	ferritin [EC:1.16.3.2]
K02221	0.000221856	0.000220711	0.000225762	0.000188917	YggT family protein
K02224	0.00021759	0.000216579	0.000217403	0.00015388	cobyrinic acid a,c-diamide synthase [EC:6.3.5.9 6.3.5.11]
K02225	0.00021502	0.00021206	0.000188113	0.000113914	cobalamin biosynthetic protein CobC
K02226	0.000218758	0.000218668	0.000216779	0.000223511	alpha-ribazole phosphatase [EC:3.1.3.73]
K02227	0.000432764	0.000431698	0.000415593	0.000317037	adenosylcobinamide-phosphate synthase [EC:6.3.1.10]
K02228	3.05827E-06	3.62885E-06	2.22738E-05	3.66193E-05	precorrin-6A synthase [EC:2.1.1.152]
K02229	2.47577E-06	3.37877E-06	1.6057E-05	3.67058E-05	precorrin-3B synthase [EC:1.14.13.83]
K02230	0.000218952	0.000216679	0.000216723	0.00015301	cobaltochelatase CobN [EC:6.6.1.2]
K02231	0.000217946	0.000217479	0.00021365	0.000164333	adenosylcobinamide kinase / adenosylcobinamide-phosphate guanylyltransferase [EC:2.7.1.156 2.7.7.62]
K02232	0.000217626	0.000216751	0.000214307	0.000167792	adenosylcobyric acid synthase [EC:6.3.5.10]
K02233	0.000218013	0.000217402	0.000213608	0.000164362	adenosylcobinamide-GDP ribazoletransferase [EC:2.7.8.26]
K02234	0.000658596	0.000658518	0.000665023	0.000637332	cobalamin biosynthesis protein CobW
K02236	1.32921E-06	3.64636E-07	3.99102E-06	3.68812E-06	leader peptidase (prepilin peptidase) / N-methyltransferase [EC:3.4.23.43 2.1.1.-]
K02237	0.00022297	0.000223169	0.000223421	0.000203249	competence protein ComEA
K02238	0.000225748	0.000224864	0.000230277	0.000226774	competence protein ComEC
K02239	0	2.06813E-07	7.1458E-08	0	competence protein ComER
K02240	1.30797E-06	3.44262E-07	6.86494E-06	3.75797E-06	competence protein ComFA
K02241	0	4.23898E-08	5.01189E-08	1.09396E-09	competence protein ComFB
K02242	4.66216E-06	5.83988E-06	1.29835E-05	6.96799E-06	competence protein ComFC
K02243	3.09896E-06	1.94519E-06	1.05391E-05	5.656E-05	competence protein ComGA
K02244	1.31755E-06	3.07615E-07	3.9801E-06	3.67919E-06	competence protein ComGB
K02245	1.21886E-06	3.20936E-07	6.71557E-06	3.46299E-06	competence protein ComGC
K02246	2.35834E-06	5.16557E-07	7.20109E-06	6.3376E-06	competence protein ComGD
K02247	1.04582E-08	2.05861E-07	2.36988E-08	0	competence protein ComGE
K02248	4.94862E-07	2.86233E-07	1.17465E-06	3.72002E-06	competence protein ComGF
K02249	1.04582E-08	2.05861E-07	2.36988E-08	0	competence protein ComGG
K02250	1.28033E-06	6.59188E-07	4.22579E-06	3.41661E-08	competence protein ComK
K02251	0	2.05861E-07	3.92124E-08	0	competence protein ComQ
K02253	0	2.05861E-07	3.35017E-08	0	competence protein ComX
K02254	0	9.5208E-10	3.99483E-07	0	competence protein ComZ
K02255	2.83571E-08	1.95297E-08	4.4334E-08	7.20944E-07	ferritin-like protein 2
K02257	0.000220935	0.000222423	0.000219409	0.000228065	heme o synthase [EC:2.5.1.141]
K02258	0.000217832	0.000216706	0.000206498	0.000210863	cytochrome c oxidase assembly protein subunit 11
K02259	0.000221099	0.000222374	0.000223469	0.000214934	cytochrome c oxidase assembly protein subunit 15
K02274	0.000869466	0.000865034	0.000807175	0.000701062	cytochrome c oxidase subunit I [EC:1.9.3.1]
K02275	0.00086522	0.000860348	0.000784937	0.000576725	cytochrome c oxidase subunit II [EC:1.9.3.1]
K02276	0.000649465	0.000646576	0.000591059	0.000443417	cytochrome c oxidase subunit III [EC:1.9.3.1]
K02277	1.98665E-07	2.06813E-07	7.5376E-07	3.41139E-06	cytochrome c oxidase subunit IV [EC:1.9.3.1]
K02278	0.000431823	0.000427655	0.000389213	0.000327666	prepilin peptidase CpaA [EC:3.4.23.43]
K02279	0.000433029	0.000429095	0.000397061	0.000339697	pilus assembly protein CpaB
K02280	0.000646391	0.000641669	0.000584386	0.000460112	pilus assembly protein CpaC
K02281	1.73084E-06	1.27686E-07	5.69933E-07	5.2428E-07	pilus assembly protein CpaD
K02282	0.000431872	0.000428556	0.000394183	0.000325783	pilus assembly protein CpaE
K02283	0.000653659	0.000653812	0.000619954	0.000512245	pilus assembly protein CpaF
K02288	0	0	1.39543E-08	0	phycocyanobilin lyase subunit alpha [EC:4.4.1.32]
K02291	0.000220086	0.000217207	0.000209126	0.000152697	15-cis-phytoene synthase [EC:2.5.1.32]
K02292	1.08899E-06	1.21426E-06	1.83666E-06	5.14349E-05	beta-carotene ketolase (CrtO type)
K02293	2.70408E-08	3.36261E-09	5.31254E-07	9.53116E-08	15-cis-phytoene desaturase [EC:1.3.5.5]
K02294	1.5984E-08	1.13298E-07	5.36711E-08	1.90174E-09	beta-carotene hydroxylase [EC:1.14.13.-]
K02297	0.000432931	0.000428196	0.000393967	0.000354836	cytochrome o ubiquinol oxidase subunit II [EC:1.10.3.10]
K02298	0.000436002	0.00043347	0.000399434	0.000364319	cytochrome o ubiquinol oxidase subunit I [EC:1.10.3.10]
K02299	0.000433363	0.000428601	0.000396222	0.000354845	cytochrome o ubiquinol oxidase subunit III
K02300	0.000432194	0.000428188	0.000392799	0.000354813	cytochrome o ubiquinol oxidase subunit IV
K02302	0.000223494	0.000222071	0.000233648	0.000196556	uroporphyrin-III C-methyltransferase / precorrin-2 dehydrogenase / sirohydrochlorin ferrochelatase [EC:2.1.1.107 1.3.[illegible]
K02303	0.000226061	0.000222698	0.000239004	0.000189312	uroporphyrin-III C-methyltransferase [EC:2.1.1.107]
K02304	1.51459E-06	6.08296E-07	7.97096E-06	1.44083E-05	precorrin-2 dehydrogenase / sirohydrochlorin ferrochelatase [EC:1.3.1.76 4.99.1.4]
K02305	1.81687E-08	1.2386E-06	7.08571E-07	1.03581E-07	nitric oxide reductase subunit C
K02313	0.000224007	0.000223932	0.000228368	0.000228733	chromosomal replication initiator protein
K02314	0.000226083	0.000228496	0.000244331	0.000269835	replicative DNA helicase [EC:3.6.4.12]
K02315	1.09878E-05	1.05138E-05	6.04756E-05	2.92493E-05	DNA replication protein DnaC
K02316	0.000225226	0.000225725	0.00023311	0.000234096	DNA primase [EC:2.7.7.-]
K02317	6.01475E-08	5.03683E-08	6.01284E-08	7.85184E-07	DNA replication protein DnaT
K02319	0	0	1.26165E-08	0	DNA polymerase, archaea type [EC:2.7.7.7]
K02323	0	0	1.89269E-08	0	DNA polymerase II small subunit [EC:2.7.7.7]
K02334	2.7953E-06	2.06671E-06	9.94336E-06	1.44412E-05	DNA polymerase bacteriophage-type [EC:2.7.7.7]
K02335	0.0002279	0.000232528	0.000236196	0.000301631	DNA polymerase I [EC:2.7.7.7]
K02336	3.85891E-07	8.47856E-07	1.50236E-06	1.85216E-05	DNA polymerase II [EC:2.7.7.7]
K02337	0.000223806	0.00022445	0.000231252	0.000226504	DNA polymerase III subunit alpha [EC:2.7.7.7]
K02338	0.000223737	0.00022392	0.000229594	0.000225678	DNA polymerase III subunit beta [EC:2.7.7.7]
K02339	0.000219343	0.00021712	0.000207365	0.00021725	DNA polymerase III subunit chi [EC:2.7.7.7]
K02340	0.000223362	0.000223745	0.000228193	0.000227784	DNA polymerase III subunit delta [EC:2.7.7.7]
K02341	0.000223206	0.000223677	0.000229777	0.000225511	DNA polymerase III subunit delta [EC:2.7.7.7]
K02342	0.000235884	0.000241426	0.000293724	0.000435079	DNA polymerase III subunit epsilon [EC:2.7.7.7]
K02343	0.000223484	0.000224895	0.000225952	0.000227494	DNA polymerase III subunit gamma/tau [EC:2.7.7.7]
K02344	9.82254E-08	3.14615E-07	3.48655E-07	1.2293E-05	DNA polymerase III subunit psi [EC:2.7.7.7]
K02345	2.36621E-07	6.65512E-07	1.08573E-07	1.37229E-06	DNA polymerase III subunit theta [EC:2.7.7.7]
K02346	0.000437628	0.000436522	0.00041811	0.000345898	DNA polymerase IV [EC:2.7.7.7]
K02347	1.37011E-06	3.43121E-07	4.12572E-06	1.83983E-06	DNA polymerase (family X)

K02348	2.49972E-06	5.67729E-06	1.46832E-05	2.77861E-05	ElaA protein
K02351	2.97202E-06	8.64346E-06	1.09468E-05	1.39958E-05	putative membrane protein
K02352	9.60631E-07	3.54778E-09	2.78823E-06	1.0957E-07	lactonase [EC:3.1.1.-]
K02355	0.000442578	0.000442855	0.000445748	0.000445613	elongation factor G
K02356	0.000225115	0.000225365	0.000229395	0.000290228	elongation factor P
K02357	0.000223539	0.000223784	0.000228242	0.00022547	elongation factor Ts
K02358	0.000442655	0.000440935	0.000436146	0.000445514	elongation factor Tu
K02361	2.32318E-06	3.29924E-06	1.01976E-05	7.16739E-05	isochorismate synthase [EC:5.4.4.2]
K02362	3.27295E-07	8.46528E-07	3.0352E-06	1.36289E-05	enterobactin synthetase component D [EC:6.3.2.14 2.7.8.-]
K02363	2.53409E-06	1.44155E-06	5.50519E-06	7.41224E-05	2,3-dihydroxybenzoate-AMP ligase [EC:6.3.2.14 2.7.7.58]
K02364	0.001295774	0.001286523	0.001202779	0.000915111	enterobactin synthetase component F [EC:6.3.2.14]
K02371	0.000218249	0.00021677	0.00019708	0.000183467	enoyl-[acyl-carrier protein] reductase II [EC:1.3.1.9]
K02372	0.000438729	0.000433556	0.000409114	0.000338147	3-hydroxyacyl-[acyl-carrier-protein] dehydratase [EC:4.2.1.59]
K02377	2.89028E-06	2.47057E-06	5.54312E-06	4.95462E-06	GDP-L-fucose synthase [EC:1.1.1.271]
K02379	0.000432732	0.000430098	0.00039863	0.000329967	FdhD protein
K02380	3.36905E-06	5.21161E-06	1.58921E-05	6.47256E-05	FdhE protein
K02381	5.80654E-08	1.99734E-07	3.56185E-07	2.14973E-06	FdrA protein
K02385	5.62595E-07	2.06952E-07	1.3585E-06	1.75722E-07	flagellar protein FlbD
K02386	0.000432912	0.00042934	0.000392852	0.000338216	flagella basal body P-ring formation protein FlgA
K02387	0.000434848	0.000432293	0.000406205	0.000366263	flagellar basal-body rod protein FlgB
K02388	0.000649334	0.000642802	0.000588411	0.000465711	flagellar basal-body rod protein FlgC
K02389	0.000433902	0.000431168	0.000398258	0.000350604	flagellar basal-body rod modification protein FlgD
K02390	0.000652932	0.000647216	0.000606734	0.000541296	flagellar hook protein FlgE
K02391	0.000221864	0.000220572	0.000217212	0.000256933	flagellar basal-body rod protein FlgF
K02392	0.000865988	0.000859585	0.00079315	0.000677356	flagellar basal-body rod protein FlgG
K02393	0.000433336	0.000429969	0.000396332	0.000345209	flagellar L-ring protein precursor FlgH
K02394	0.000433642	0.000429926	0.000396419	0.000345205	flagellar P-ring protein precursor FlgI
K02395	0.000217327	0.000218634	0.000209335	0.00023427	flagellar protein FlgJ
K02396	0.000220165	0.000218506	0.000212641	0.000257675	flagellar hook-associated protein 1 FlgK
K02397	0.000218457	0.0002186	0.000208426	0.000225242	flagellar hook-associated protein 3 FlgL
K02398	0.000217133	0.000217735	0.000207025	0.000224148	negative regulator of flagellin synthesis FlgM
K02399	0.000214309	0.000213157	0.000188806	0.000137819	flagella synthesis protein FlgN
K02400	0.000647634	0.000642946	0.00058796	0.000458961	flagellar biosynthesis protein FlhA
K02401	0.000648173	0.000642984	0.000588033	0.000459135	flagellar biosynthetic protein FlhB
K02402	0.000215315	0.000215298	0.00020295	0.000149338	flagellar transcriptional activator FlhC
K02403	0.000215301	0.000215321	0.000203112	0.000149348	flagellar transcriptional activator FlhD
K02404	0.000216619	0.000216908	0.000206522	0.00021704	flagellar biosynthesis protein FlhF
K02405	0.000645169	0.0006424	0.000584708	0.000458904	RNA polymerase sigma factor for flagellar operon FliA
K02406	0.000223646	0.000223971	0.000238743	0.000417489	flagellin
K02407	0.0002182	0.000217958	0.000211135	0.000225121	flagellar hook-associated protein 2
K02408	0.00043378	0.000430381	0.000397656	0.000344705	flagellar hook-basal body complex protein FliE
K02409	0.000434149	0.000431483	0.000398111	0.000347405	flagellar M-ring protein FliF
K02410	0.000219839	0.000218007	0.000208587	0.00022465	flagellar motor switch protein FliG
K02411	0.000432684	0.000429772	0.000394997	0.000336401	flagellar assembly protein FliH
K02412	0.000648827	0.000643588	0.000589872	0.000510431	flagellum-specific ATP synthase [EC:3.6.3.14]
K02413	0.000216996	0.000217307	0.000206383	0.000217146	flagellar FliJ protein
K02414	0.000218006	0.000217797	0.000207836	0.00022432	flagellar hook-length control protein FliK
K02415	0.000219595	0.000218252	0.000209426	0.000240671	flagellar FliL protein
K02416	0.000219839	0.000218008	0.000208417	0.000218459	flagellar motor switch protein FliM
K02417	0.000435834	0.000431091	0.000400581	0.000351771	flagellar motor switch protein FliN/FliY
K02418	0.000217756	0.000218147	0.000207315	0.00021753	flagellar protein FliO/FliZ
K02419	0.000433959	0.000431109	0.000399108	0.000345034	flagellar biosynthetic protein FliP
K02420	0.000433774	0.000431047	0.000398849	0.00034491	flagellar biosynthetic protein FliQ
K02421	0.000433771	0.000430373	0.000398528	0.000344862	flagellar biosynthetic protein FliR
K02422	0.000217255	0.000218421	0.000209273	0.000224659	flagellar protein FliS
K02423	0.000429092	0.000427213	0.000389031	0.000262071	flagellar protein FliT
K02424	6.31825E-06	2.76325E-06	8.01499E-06	1.39736E-05	cystine transport system substrate-binding protein
K02425	7.4128E-08	3.00735E-07	6.83387E-08	3.4439E-07	regulator of sigma S factor FliZ
K02426	4.43211E-06	7.11073E-06	7.31177E-06	7.23237E-05	cysteine desulfuration protein SufE
K02427	0.000219344	0.000217755	0.00020793	0.000217646	23S rRNA (uridine2552-2-O)-methyltransferase [EC:2.1.1.166]
K02428	0.000227901	0.000230992	0.000243148	0.000249782	XTP/dITP diphosphohydrolase [EC:3.6.1.66]
K02429	6.52541E-06	6.58307E-06	1.73868E-05	0.000130439	MFS transporter, FHS family, L-fucose permease
K02430	1.92953E-07	4.54654E-07	2.37617E-07	4.53935E-06	DeoR family transcriptional regulator, L-fucose operon activator
K02431	3.04245E-07	7.87073E-07	9.44689E-07	1.28847E-06	L-fucose mutarotase [EC:5.1.3.29]
K02433	0.000456371	0.000457905	0.000524694	0.000568647	aspartyl-tRNA(Asn)/glutamyl-tRNA(Gln) amidotransferase subunit A [EC:6.3.5.6 6.3.5.7]
K02434	0.000221532	0.000220427	0.000222835	0.00015687	aspartyl-tRNA(Asn)/glutamyl-tRNA(Gln) amidotransferase subunit B [EC:6.3.5.6 6.3.5.7]
K02435	0.000221432	0.000220396	0.000222765	0.000156846	aspartyl-tRNA(Asn)/glutamyl-tRNA(Gln) amidotransferase subunit C [EC:6.3.5.6 6.3.5.7]
K02436	1.40978E-07	1.88252E-08	8.81925E-08	4.64079E-07	DeoR family transcriptional regulator, galactitol utilization operon repressor
K02437	0.000222579	0.000223576	0.000229467	0.000222293	glycine cleavage system H protein
K02438	0.000218913	0.000217456	0.000204824	0.000186877	glycogen debranching enzyme [EC:3.2.1.196]
K02439	2.94518E-07	8.26436E-07	1.51108E-06	1.79153E-05	thiosulfate sulfurtransferase [EC:2.8.1.1]
K02440	4.88113E-06	4.40044E-06	2.01189E-05	3.43187E-05	glycerol uptake facilitator protein
K02441	4.42151E-07	1.49886E-06	4.66668E-06	1.9559E-05	GlpG protein
K02442	3.61336E-07	3.13406E-07	4.20676E-07	9.13646E-06	membrane protein GlpM
K02443	2.69877E-06	3.39117E-06	1.83116E-05	3.49401E-05	glycerol uptake operon antiterminator
K02444	0.000219335	0.000217214	0.000215001	0.000205815	DeoR family transcriptional regulator, glycerol-3-phosphate regulon repressor
K02445	1.18943E-06	3.48323E-07	8.42867E-06	1.33564E-05	MFS transporter, OPA family, glycerol-3-phosphate transporter
K02446	2.7532E-06	4.48545E-06	5.28051E-06	1.65595E-05	fructose-1,6-bisphosphatase II [EC:3.1.3.11]
K02448	2.73515E-07	1.87804E-06	3.53232E-06	7.09693E-06	nitric oxide reductase NorD protein
K02450	7.44263E-07	3.14368E-07	4.54551E-06	1.82369E-05	general secretion pathway protein A
K02451	1.3627E-07	6.87293E-08	2.61022E-06	1.79149E-05	general secretion pathway protein B
K02452	0.000217227	0.000215939	0.000205259	0.000198293	general secretion pathway protein C
K02453	0.000650015	0.000648618	0.000611823	0.000558083	general secretion pathway protein D
K02454	0.000656544	0.000655383	0.000641913	0.000721151	general secretion pathway protein E
K02455	0.000434342	0.000433747	0.000410708	0.000400979	general secretion pathway protein F
K02456	0.001726906	0.001710208	0.001563185	0.001225704	general secretion pathway protein G
K02457	0.000221442	0.000226981	0.000258723	0.000365012	general secretion pathway protein H

K02458	0.000219784	0.000220968	0.000224151	0.000286591	general secretion pathway protein I
K02459	0.000223742	0.000227333	0.000255048	0.000378938	general secretion pathway protein J
K02460	0.000219623	0.000220826	0.000223594	0.00028464	general secretion pathway protein K
K02461	0.000219156	0.00022012	0.000224142	0.000283967	general secretion pathway protein L
K02462	0.000217978	0.000220038	0.000222807	0.00028384	general secretion pathway protein M
K02463	0.000217321	0.000216145	0.000205699	0.000216052	general secretion pathway protein N
K02464	1.23306E-06	6.25466E-07	1.96821E-06	1.44464E-06	general secretion pathway protein O [EC:3.4.23.43 2.1.1.-]
K02465	1.07328E-07	4.32047E-07	3.83298E-08	7.22121E-08	general secretion pathway protein S
K02466	1.27617E-07	3.77184E-07	5.43814E-07	6.35154E-06	glucitol operon activator protein
K02467	0.000214133	0.000212143	0.000186544	0.000122363	arabinose 5-phosphate isomerase [EC:5.3.1.13]
K02468	2.69833E-07	8.71883E-07	4.05631E-06	2.40723E-05	DeoR family transcriptional regulator, glucitol operon repressor
K02469	0.000446969	0.000445425	0.000451131	0.000450854	DNA gyrase subunit A [EC:5.99.1.3]
K02470	0.0004465	0.000445352	0.000450954	0.000449138	DNA gyrase subunit B [EC:5.99.1.3]
K02471	0.000221638	0.000218438	0.000219317	0.000214976	vitamin B12/bleomycin/antimicrobial peptide transport system ATP-binding/permease protein
K02472	0.000217428	0.000219496	0.000198058	0.000126501	UDP-N-acetyl-D-mannosaminuronic acid dehydrogenase [EC:1.1.1.336]
K02473	4.07313E-07	2.22342E-06	1.74104E-06	3.51994E-06	UDP-N-acetylglucosamine 4-epimerase [EC:5.1.3.7]
K02474	4.98488E-06	6.14413E-06	9.50124E-06	5.5037E-05	UDP-N-acetyl-D-galactosamine dehydrogenase [EC:1.1.1.-]
K02475	5.42152E-07	6.02214E-07	2.5149E-06	1.79404E-05	two-component system, CitB family, response regulator
K02476	1.11333E-06	3.65654E-06	5.39219E-06	2.58289E-05	two-component system, CitB family, sensor kinase [EC:2.7.13.3]
K02477	1.31586E-05	1.89283E-05	5.80555E-05	0.000249389	two-component system, LytTR family, response regulator
K02478	3.24015E-06	3.47796E-06	1.34932E-05	7.84903E-05	two-component system, LytTR family, sensor kinase [EC:2.7.13.3]
K02479	6.60788E-06	9.10797E-06	2.74175E-05	0.000207197	two-component system, NarL family, response regulator
K02480	0.000434482	0.000434178	0.0004265	0.000346904	two-component system, NarL family, sensor kinase [EC:2.7.13.3]
K02481	0.001741561	0.001738156	0.001664468	0.001593792	two-component system, NtrC family, response regulator
K02482	0.000245876	0.000229331	0.000261425	0.000538106	two-component system, NtrC family, sensor kinase [EC:2.7.13.3]
K02483	0.005475989	0.00545929	0.005340594	0.005377799	two-component system, OmpR family, response regulator
K02484	0.001752779	0.001749548	0.00169619	0.001680557	two-component system, OmpR family, sensor kinase [EC:2.7.13.3]
K02485	4.73142E-06	3.65111E-06	6.82546E-06	0.000110231	two-component system, response regulator
K02486	1.30122E-07	1.44645E-07	4.25742E-07	2.02694E-06	two-component system, sensor kinase [EC:2.7.13.3]
K02487	0.000435131	0.000432111	0.00040919	0.000353455	type IV pili sensor histidine kinase and response regulator
K02488	0.001766177	0.001762031	0.001794042	0.002255319	two-component system, cell cycle response regulator [EC:2.7.7.65]
K02489	4.18901E-08	4.3943E-07	4.77202E-07	9.29222E-06	two-component system, glycerol uptake and utilization sensor kinase [EC:2.7.13.3]
K02490	1.9438E-07	8.27702E-07	1.50315E-06	1.58933E-06	two-component system, response regulator, stage 0 sporulation protein F
K02491	3.02566E-07	9.05237E-07	2.55097E-06	5.01918E-06	two-component system, sporulation sensor kinase A [EC:2.7.13.3]
K02492	0.000221852	0.000225258	0.000223236	0.000223262	glutamyl-tRNA reductase [EC:1.2.1.70]
K02493	0.000225299	0.000229089	0.000237241	0.000236189	release factor glutamine methyltransferase [EC:2.1.1.297]
K02494	0.000217642	0.00021692	0.000207106	0.000217512	outer membrane lipoprotein LolB
K02495	0.000438343	0.000437583	0.000426325	0.000409219	oxygen-independent coproporphyrinogen III oxidase [EC:1.3.98.3]
K02496	0.000216478	0.000216238	0.000206096	0.000214022	uroporphyrin-III C-methyltransferase [EC:2.1.1.107]
K02497	1.05101E-06	2.06813E-07	3.36174E-06	3.03626E-08	HemX protein
K02498	0.000217954	0.000217071	0.000206497	0.000217509	HemY protein
K02499	5.73898E-06	6.68562E-06	1.76812E-05	4.35636E-05	tetrapyrrole methylase family protein / MazG family protein
K02500	0.000439238	0.000435448	0.000420233	0.000343245	cyclase [EC:4.1.3.-]
K02501	0.000224021	0.000223881	0.000237659	0.00022959	glutamine amidotransferase [EC:2.4.2.-]
K02502	0.000218489	0.000216249	0.000209641	0.000149476	ATP phosphoribosyltransferase regulatory subunit
K02503	0.000225963	0.000231023	0.00024487	0.000264829	histidine triad (HIT) family protein
K02504	4.33495E-06	7.80163E-06	3.44696E-05	0.000140397	protein transport protein HofB
K02505	1.34005E-06	4.87923E-07	1.54672E-06	2.17502E-05	protein transport protein HofC
K02506	7.17006E-08	9.05133E-08	8.12481E-08	6.75376E-07	leader peptidase HopD [EC:3.4.23.43]
K02507	2.37565E-07	3.45466E-07	1.06854E-07	3.04082E-06	protein transport protein HofQ
K02508	1.13469E-07	2.894E-07	2.65719E-07	9.001E-07	AraC family transcriptional regulator, 4-hydroxyphenylacetate 3-monooxygenase operon regulatory protein
K02509	4.70137E-06	1.00253E-05	3.84236E-05	8.01578E-05	2-oxo-hept-3-ene-1,7-dioate hydratase [EC:4.2.1.-]
K02510	0.000649944	0.000651044	0.000628729	0.000486745	4-hydroxy-2-oxoheptanedioate aldolase [EC:4.1.2.52]
K02511	0.000223233	0.000223919	0.000237839	0.000240986	MFS transporter, ACS family, 4-hydroxyphenylacetate permease
K02517	0.000652378	0.000647998	0.000604184	0.000533362	Kdo2-lipid IVA lauroyltransferase [EC:2.3.1.241]
K02518	0.000652546	0.000650423	0.000617717	0.000486932	translation initiation factor IF-1
K02519	0.000224788	0.000224332	0.000228527	0.000230007	translation initiation factor IF-2
K02520	0.000223494	0.00022382	0.000228107	0.000225017	translation initiation factor IF-3
K02521	1.52823E-06	3.00957E-06	1.61957E-05	6.53568E-05	LysR family transcriptional regulator, positive regulator for ilvC
K02523	0.000220185	0.000220277	0.000217684	0.00022092	octaprenyl-diphosphate synthase [EC:2.5.1.90]
K02525	7.74094E-07	1.07198E-06	3.27143E-06	1.01782E-05	LacI family transcriptional regulator, kdg operon repressor
K02526	1.44446E-06	1.30631E-07	1.77754E-06	1.14715E-06	2-keto-3-deoxygluconate permease
K02527	0.000219758	0.000218764	0.000210919	0.000217748	3-deoxy-D-manno-octulosonic-acid transferase [EC:2.4.99.12 2.4.99.13 2.4.99.14 2.4.99.15]
K02528	0.000224035	0.000224213	0.000229325	0.000234775	16S rRNA (adenine1518-N6/adenine1519-N6)-dimethyltransferase [EC:2.1.1.182]
K02529	0.001751738	0.001750337	0.001729331	0.00177719	LacI family transcriptional regulator
K02530	1.11374E-06	1.71414E-07	9.56936E-06	5.45213E-06	DeoR family transcriptional regulator, lactose phosphotransferase system repressor
K02531	6.24792E-08	6.10918E-09	2.44141E-07	3.36346E-06	transcriptional antiterminator
K02532	4.56537E-07	3.3605E-07	4.15616E-07	3.47678E-06	MFS transporter, OHS family, lactose permease
K02533	0.000217656	0.000216668	0.000205134	0.000168563	tRNA/rRNA methyltransferase [EC:2.1.1.-]
K02535	0.000220617	0.000218289	0.000210703	0.000217714	UDP-3-O-[3-hydroxymyristoyl] N-acetylglucosamine deacetylase [EC:3.5.1.108]
K02536	0.000223928	0.000222024	0.000219543	0.000235965	UDP-3-O-[3-hydroxymyristoyl] glucosamine N-acyltransferase [EC:2.3.1.191]
K02538	1.03828E-06	2.97769E-07	2.82213E-06	1.63959E-06	activator of the mannose operon, transcriptional antiterminator
K02545	1.24386E-08	0	8.95526E-08	1.07162E-08	penicillin-binding protein 2 prime [EC:3.4.16.4]
K02546	0	0	8.78778E-08	0	BlaI family transcriptional regulator, methicillin resistance regulatory protein
K02547	0	0	8.78778E-08	0	methicillin resistance protein
K02548	4.32968E-06	6.72489E-06	1.78114E-05	1.98591E-05	1,4-dihydroxy-2-naphthoate octaprenyltransferase [EC:2.5.1.74 2.5.1.-]
K02549	3.86106E-06	5.00024E-06	1.3988E-05	2.40229E-05	O-succinylbenzoate synthase [EC:4.2.1.113]
K02550	3.81575E-07	8.2292E-08	2.40292E-07	8.83594E-07	glycolate permease
K02551	3.38228E-06	7.08739E-06	1.59485E-05	1.95381E-05	2-succinyl-5-enolpyruvyl-6-hydroxy-3-cyclohexene-1-carboxylate synthase [EC:2.2.1.9]
K02552	4.39842E-06	5.57036E-06	2.00592E-05	7.26313E-05	menaquinone-specific isochorismate synthase [EC:5.4.4.2]
K02553	0.000217025	0.00021594	0.000203701	0.000184667	regulator of ribonuclease activity A
K02554	0.000216109	0.000215458	0.000201685	0.000174071	2-keto-4-pentenoate hydratase [EC:4.2.1.80]
K02556	0.000222322	0.000219335	0.000213125	0.00028668	chemotaxis protein MotA
K02557	0.000659197	0.00065796	0.000638227	0.000735322	chemotaxis protein MotB
K02558	0.000219101	0.00022144	0.000211206	0.000217395	UDP-N-acetylmuramate: L-alanyl-gamma-D-glutamyl-meso-diaminopimelate ligase [EC:6.3.2.45]
K02560	0.000213936	0.000212381	0.000187162	0.000133505	lauroyl-Kdo2-lipid IVA myristoyltransferase [EC:2.3.1.243]
K02562	9.82183E-08	3.14597E-07	3.19761E-07	1.18223E-05	mannitol operon repressor

K02563	0.000223808	0.000223917	0.000228491	0.000225482	UDP-N-acetylglucosamine--N-acetylmuramyl-(pentapeptide) pyrophosphoryl-undecaprenol N-acetylglucosamine tran:
K02564	4.54743E-06	6.74172E-06	2.47175E-05	2.37258E-05	glucosamine-6-phosphate deaminase [EC:3.5.99.6]
K02565	9.82588E-08	3.14698E-07	3.44169E-07	1.23123E-05	N-acetylglucosamine repressor
K02566	1.12707E-06	1.55252E-06	1.22844E-05	9.41552E-06	NagD protein
K02567	3.90902E-07	1.30186E-06	2.57189E-06	1.94801E-05	periplasmic nitrate reductase NapA [EC:1.7.99.-]
K02568	3.67494E-08	1.22407E-06	1.81716E-06	1.43644E-05	cytochrome c-type protein NapB
K02569	1.12705E-07	1.77904E-06	2.08046E-06	2.52225E-05	cytochrome c-type protein NapC
K02570	3.05784E-08	1.19968E-06	1.81575E-06	1.43647E-05	periplasmic nitrate reductase NapD
K02571	2.93319E-08	1.18135E-06	1.2909E-06	1.00261E-05	periplasmic nitrate reductase NapE
K02572	1.2683E-08	7.28061E-07	7.33208E-07	1.61846E-05	ferredoxin-type protein NapF
K02573	9.68604E-09	6.31628E-08	1.1526E-06	4.94775E-06	ferredoxin-type protein NapG
K02574	2.67071E-07	2.47038E-07	1.94305E-06	8.02741E-06	ferredoxin-type protein NapH
K02575	0.000655433	0.000649782	0.000636568	0.000555766	MFS transporter, NNP family, nitrate/nitrite transporter
K02584	0.001742429	0.001738999	0.001674037	0.00161225	Nif-specific regulatory protein
K02585	5.85466E-08	2.80616E-07	3.90658E-07	1.16755E-07	nitrogen fixation protein NifB
K02586	7.24339E-08	3.06499E-07	2.83997E-07	2.02907E-07	nitrogenase molybdenum-iron protein alpha chain [EC:1.18.6.1]
K02587	2.00394E-07	2.76652E-07	3.03231E-07	1.34169E-07	nitrogenase molybdenum-cofactor synthesis protein NifE
K02588	1.31159E-06	7.64338E-07	1.24225E-06	3.00446E-07	nitrogenase iron protein NifH [EC:1.18.6.1]
K02589	0	1.41012E-07	2.8053E-06	4.22927E-09	nitrogen regulatory protein PII 1
K02590	6.09192E-08	1.23529E-07	9.34963E-07	3.99843E-06	nitrogen regulatory protein PII 2
K02591	5.85466E-08	2.43457E-07	3.9078E-07	1.15638E-07	nitrogenase molybdenum-iron protein beta chain [EC:1.18.6.1]
K02592	6.02592E-08	9.4519E-08	2.07621E-07	1.15638E-07	nitrogenase molybdenum-iron protein NifN
K02593	3.80671E-08	8.06108E-08	1.85386E-07	1.14544E-07	nitrogen fixation protein NifT
K02594	5.055E-07	2.90363E-07	4.95238E-07	3.26835E-07	homocitrate synthase NifV [EC:2.3.3.14]
K02595	3.80671E-08	8.06108E-08	1.73757E-07	1.14544E-07	nitrogenase-stabilizing/protective protein
K02596	1.19985E-07	1.50559E-07	2.31602E-07	1.38699E-07	nitrogen fixation protein NifX
K02597	4.70918E-08	1.33202E-07	2.86185E-07	2.19042E-07	nitrogen fixation protein NifZ
K02598	3.25532E-07	8.40863E-07	6.27194E-06	1.6826E-05	nitrite transporter
K02599	0	0	8.34781E-08	0	Notch 1
K02600	0.000223643	0.000223749	0.000227871	0.000225456	N utilization substance protein A
K02601	0.000223553	0.000223944	0.000228179	0.00022546	transcriptional antiterminator NusG
K02609	0.000216294	0.000218367	0.000202561	0.000150818	ring-1,2-phenylacetyl-CoA epoxidase subunit PaaA [EC:1.14.13.149]
K02610	0.000216294	0.000218367	0.000202539	0.000150818	ring-1,2-phenylacetyl-CoA epoxidase subunit PaaB
K02611	0.000216293	0.000218367	0.000202535	0.000150819	ring-1,2-phenylacetyl-CoA epoxidase subunit PaaC [EC:1.14.13.149]
K02612	0.000216294	0.000218367	0.000202535	0.000150799	ring-1,2-phenylacetyl-CoA epoxidase subunit PaaD
K02613	0.000216419	0.000220382	0.000203805	0.000156306	ring-1,2-phenylacetyl-CoA epoxidase subunit PaaE
K02614	0.000218278	0.00022264	0.000207404	0.000155237	acyl-CoA thioesterase [EC:3.1.2.-]
K02615	0.000217295	0.000220582	0.000211243	0.000185912	3-oxo-5,6-didehydrosuberyl-CoA/3-oxoadipyl-CoA thiolase [EC:2.3.1.223 2.3.1.174]
K02616	4.87148E-07	6.1211E-07	2.41197E-06	5.14917E-06	phenylacetic acid degradation operon negative regulatory protein
K02617	6.59199E-06	8.11918E-06	2.30181E-05	4.07716E-05	phenylacetic acid degradation protein
K02618	1.25228E-06	3.81344E-06	2.67444E-06	3.05694E-06	oxepin-CoA hydrolase / 3-oxo-5,6-dehydrosuberyl-CoA semialdehyde dehydrogenase [EC:3.3.2.12 1.2.1.91]
K02619	4.55846E-06	5.76001E-06	1.49681E-05	2.19503E-05	4-amino-4-deoxychorismate lyase [EC:4.1.3.38]
K02621	0.000444353	0.000441595	0.000441997	0.00044726	topoisomerase IV subunit A [EC:5.99.1.-]
K02622	0.000231363	0.000231867	0.000249644	0.000300837	topoisomerase IV subunit B [EC:5.99.1.-]
K02623	0.000649885	0.000649061	0.000621347	0.000518201	LysR family transcriptional regulator, pca operon transcriptional activator
K02624	0.00043881	0.00045162	0.000488403	0.000521251	IclR family transcriptional regulator, pca regulon regulatory protein
K02625	4.39996E-07	2.52563E-07	3.1859E-06	2.09276E-05	MFS transporter, MHS family, dicarboxylic acid transporter PcaT
K02626	0	0	2.47154E-07	3.2749E-08	arginine decarboxylase [EC:4.1.1.19]
K02635	9.16819E-09	0	1.54857E-08	2.67905E-09	cytochrome b6
K02636	5.63114E-07	8.07055E-07	2.9055E-06	2.21641E-05	cytochrome b6-f complex iron-sulfur subunit [EC:1.10.9.1]
K02637	0	0	4.35362E-08	8.47917E-09	cytochrome b6-f complex subunit 4
K02639	3.97169E-09	7.83562E-08	2.24949E-06	6.51098E-07	ferredoxin
K02641	2.15678E-09	8.69011E-09	6.1752E-10	9.11566E-09	ferredoxin--NADP+ reductase [EC:1.18.1.2]
K02647	1.54735E-06	1.47224E-06	5.46743E-06	1.83378E-05	carbohydrate diacid regulator
K02650	0.000438699	0.000438833	0.000428273	0.000548195	type IV pilus assembly protein PilA
K02651	0.000861236	0.000854226	0.000776098	0.000580554	pilus assembly protein Flp/PilA
K02652	0.000657871	0.00065716	0.000652743	0.000787579	type IV pilus assembly protein PilB
K02653	0.000221893	0.000222061	0.000226505	0.000306406	type IV pilus assembly protein PilC
K02654	0.000220619	0.000221956	0.000213588	0.000222692	leader peptidase (prepilin peptidase) / N-methyltransferase [EC:3.4.23.43 2.1.1.-]
K02655	0.001296597	0.001287948	0.001191447	0.00104031	type IV pilus assembly protein PilE
K02656	0.00021764	0.000216989	0.000206659	0.000216144	type IV pilus assembly protein PilF
K02657	0.00021749	0.000216818	0.000207924	0.000206755	twitching motility two-component system response regulator PilG
K02658	0.000220416	0.000221004	0.000211103	0.000263825	twitching motility two-component system response regulator PilH
K02659	0.000218137	0.000218211	0.000214282	0.000220513	twitching motility protein PilI
K02660	0.000220773	0.000219906	0.00022346	0.000337682	twitching motility protein PilJ
K02661	5.77523E-07	4.36877E-07	1.37477E-06	1.64929E-07	type IV pilus assembly protein PilK
K02662	0.000217772	0.000217042	0.000207337	0.000216519	type IV pilus assembly protein PilM
K02663	0.000217567	0.000216753	0.000206707	0.00021623	type IV pilus assembly protein PilN
K02664	0.000217526	0.000216749	0.00020684	0.000216231	type IV pilus assembly protein PilO
K02665	0.000217455	0.000216465	0.000206351	0.000216214	type IV pilus assembly protein PilP
K02666	0.000219366	0.000219324	0.000218669	0.000250804	type IV pilus assembly protein PilQ
K02667	0.001742424	0.001741789	0.001695123	0.001596607	two-component system, NtrC family, response regulator PilR
K02668	0.000654835	0.000650587	0.000629201	0.000564167	two-component system, NtrC family, sensor histidine kinase PilS [EC:2.7.13.3]
K02669	0.000446157	0.000451253	0.000489248	0.000672239	twitching motility protein PilT
K02670	0.000221212	0.000222875	0.000232911	0.000313898	twitching motility protein PilU
K02671	0.000649417	0.000646455	0.000608744	0.000556637	type IV pilus assembly protein PilV
K02672	0.000863273	0.000858726	0.000797622	0.000655225	type IV pilus assembly protein PilW
K02673	0.000433017	0.00043477	0.000421238	0.000403889	type IV pilus assembly protein PilX
K02674	0.000861759	0.000858381	0.000795987	0.000639315	type IV pilus assembly protein PilY1
K02676	0.000217455	0.000216438	0.000206403	0.000204719	type IV pilus assembly protein PilZ
K02679	1.54161E-07	3.06837E-07	9.68427E-08	8.8838E-07	prepilin peptidase dependent protein A
K02680	1.56046E-07	3.51779E-07	2.17804E-07	4.58989E-06	prepilin peptidase dependent protein B
K02681	9.82118E-08	3.01003E-07	9.72084E-08	8.0882E-07	prepilin peptidase dependent protein C
K02682	0.000218825	0.000220478	0.000227211	0.000234285	prepilin peptidase dependent protein D
K02686	0.000214946	0.000214341	0.000200579	0.000155473	primosomal replication protein N
K02687	0.000222051	0.000220494	0.000218788	0.000223479	ribosomal protein L11 methyltransferase [EC:2.1.1.-]
K02688	0.001300344	0.00129698	0.001233715	0.00109435	transcriptional regulator, propionate catabolism operon regulatory protein

K02742	2.53557E-07	7.51932E-07	1.50056E-06	1.42574E-05	SprT protein
K02744	1.34186E-07	4.16527E-07	5.87812E-06	6.48408E-06	PTS system, N-acetylgalactosamine-specific IIA component [EC:2.7.1.-]
K02745	1.12593E-07	4.00967E-07	3.02175E-06	4.69944E-06	PTS system, N-acetylgalactosamine-specific IIB component [EC:2.7.1.-]
K02746	1.33536E-07	4.29572E-07	2.50857E-07	4.18829E-06	PTS system, N-acetylgalactosamine-specific IIC component
K02747	1.35805E-07	4.30835E-07	2.50676E-07	4.31712E-06	PTS system, N-acetylgalactosamine-specific IID component
K02749	2.17419E-06	7.28481E-07	8.05734E-06	2.05056E-05	PTS system, alpha-glucoside-specific IIB component [EC:2.7.1.208 2.7.1.-]
K02750	2.17419E-06	7.28481E-07	8.05734E-06	2.05056E-05	PTS system, alpha-glucoside-specific IIC component
K02752	1.10857E-07	3.48459E-07	4.34204E-07	4.88118E-06	PTS system, beta-glucoside (arbutin/salicin/cellobiose)-specific IIB component [EC:2.7.1.-]
K02753	1.10857E-07	3.48459E-07	4.34204E-07	4.88118E-06	PTS system, beta-glucoside (arbutin/salicin/cellobiose)-specific IIC component
K02755	0.000221974	0.000216394	0.000233289	0.000151451	PTS system, beta-glucoside-specific IIA component [EC:2.7.1.-]
K02756	0.000221898	0.000216442	0.000236126	0.000149787	PTS system, beta-glucoside-specific IIB component [EC:2.7.1.-]
K02757	0.000221934	0.000216442	0.000236157	0.000149813	PTS system, beta-glucoside-specific IIC component
K02759	1.30118E-06	1.20185E-06	1.75358E-05	9.90753E-06	PTS system, cellobiose-specific IIA component [EC:2.7.1.196 2.7.1.205]
K02760	1.31704E-06	1.3323E-06	1.77833E-05	1.37836E-05	PTS system, cellobiose-specific IIB component [EC:2.7.1.196 2.7.1.205]
K02761	4.03054E-06	4.30308E-06	4.47027E-05	5.7617E-05	PTS system, cellobiose-specific IIC component
K02763	8.54206E-09	0	2.22133E-08	0	PTS system, D-glucosamine-specific IIA component [EC:2.7.1.-]
K02764	8.54206E-09	0	2.22133E-08	0	PTS system, D-glucosamine-specific IIB component [EC:2.7.1.-]
K02765	8.54206E-09	0	2.22133E-08	0	PTS system, D-glucosamine-specific IIC component
K02768	0.000655591	0.000649504	0.000659183	0.00061241	PTS system, fructose-specific IIA component [EC:2.7.1.202]
K02769	0.000442562	0.00043724	0.000481229	0.000461103	PTS system, fructose-specific IIB component [EC:2.7.1.202]
K02770	0.000442376	0.000437115	0.000476239	0.000449747	PTS system, fructose-specific IIC component
K02771	2.75204E-08	6.62726E-08	2.91761E-06	2.61741E-06	PTS system, fructose-specific IID component
K02773	1.12281E-06	8.07934E-07	1.46263E-05	1.997E-06	PTS system, galactitol-specific IIA component [EC:2.7.1.200]
K02774	1.61018E-06	1.10358E-06	1.97449E-05	1.16271E-06	PTS system, galactitol-specific IIB component [EC:2.7.1.200]
K02775	9.70838E-07	1.27106E-06	1.21301E-05	1.33752E-06	PTS system, galactitol-specific IIC component
K02777	0.000219001	0.000215591	0.000224132	0.000147373	PTS system, sugar-specific IIA component [EC:2.7.1.-]
K02778	3.11679E-06	2.18173E-06	2.0529E-05	2.83673E-05	PTS system, glucose-specific IIB component [EC:2.7.1.199]
K02779	3.0608E-06	2.06833E-06	2.0458E-05	2.72658E-05	PTS system, glucose-specific IIC component
K02781	1.29448E-06	1.0473E-06	1.1733E-05	1.43502E-05	PTS system, glucitol/sorbitol-specific IIA component [EC:2.7.1.198]
K02782	6.50114E-07	7.75931E-07	6.07011E-06	1.39876E-05	PTS system, glucitol/sorbitol-specific IIB component [EC:2.7.1.198]
K02783	1.29997E-06	1.53486E-06	1.21326E-05	2.75072E-05	PTS system, glucitol/sorbitol-specific IIC component
K02784	3.02089E-07	3.15533E-07	1.04243E-06	1.3793E-05	phosphocarrier protein HPr
K02786	1.02958E-06	3.98233E-09	5.88192E-06	3.12642E-06	PTS system, lactose-specific IIA component [EC:2.7.1.207]
K02787	1.17808E-06	2.05796E-08	5.95685E-06	4.40808E-06	PTS system, lactose-specific IIB component [EC:2.7.1.207]
K02788	1.17808E-06	2.05796E-08	5.95685E-06	4.40808E-06	PTS system, lactose-specific IIC component
K02790	4.79756E-06	3.34311E-06	2.25445E-05	3.4889E-05	PTS system, maltose/glucose-specific IIB component [EC:2.7.1.199 2.7.1.208]
K02791	4.79756E-06	3.34311E-06	2.25445E-05	3.4889E-05	PTS system, maltose/glucose-specific IIC component
K02793	2.97219E-06	1.76714E-06	3.20654E-06	4.24007E-05	PTS system, mannose-specific IIA component [EC:2.7.1.191]
K02794	8.49402E-07	1.58385E-06	4.76068E-06	1.61003E-05	PTS system, mannose-specific IIB component [EC:2.7.1.191]
K02795	7.68116E-07	1.19155E-06	4.40402E-06	1.65868E-05	PTS system, mannose-specific IIC component
K02796	6.28691E-07	1.24882E-06	6.89784E-06	1.48112E-05	PTS system, mannose-specific IID component
K02798	1.83871E-06	1.81647E-06	1.81273E-05	2.63631E-05	PTS system, mannitol-specific IIA component [EC:2.7.1.197]
K02799	2.47917E-06	2.38755E-06	2.37827E-05	3.57272E-05	PTS system, mannitol-specific IIB component [EC:2.7.1.197]
K02800	2.47018E-06	2.38348E-06	2.3659E-05	3.50236E-05	PTS system, mannitol-specific IIC component
K02802	5.74887E-06	3.47068E-06	2.67413E-05	3.42239E-05	PTS system, N-acetylglucosamine-specific IIA component [EC:2.7.1.193]
K02803	1.17181E-05	6.95169E-06	5.68696E-05	7.45535E-05	PTS system, N-acetylglucosamine-specific IIB component [EC:2.7.1.193]
K02804	1.49266E-05	8.70923E-06	6.55568E-05	0.000101156	PTS system, N-acetylglucosamine-specific IIC component
K02805	0.000219115	0.000218261	0.000207061	0.000169315	dTDP-4-amino-4,6-dideoxygalactose transaminase [EC:2.6.1.59]
K02806	0.000219844	0.000216929	0.000208654	0.000227517	PTS system, nitrogen regulatory IIA component [EC:2.7.1.-]
K02808	0.000216512	0.000213083	0.000207179	0.000121701	PTS system, sucrose-specific IIA component [EC:2.7.1.211]
K02809	0.0004336	0.000427022	0.000410472	0.000262055	PTS system, sucrose-specific IIB component [EC:2.7.1.211]
K02810	0.0004336	0.000427022	0.000410472	0.000262055	PTS system, sucrose-specific IIC component
K02812	2.57316E-08	8.57464E-09	1.84493E-08	1.03046E-07	PTS system, sorbose-specific IIA component [EC:2.7.1.206]
K02813	2.57413E-08	1.26716E-08	1.85194E-08	3.14298E-07	PTS system, sorbose-specific IIB component [EC:2.7.1.206]
K02814	1.34408E-07	3.63573E-07	1.59268E-07	9.52016E-07	PTS system, sorbose-specific IIC component
K02815	1.34593E-07	3.30391E-07	1.51847E-07	1.02627E-06	PTS system, sorbose-specific IID component
K02817	9.99092E-07	4.39378E-07	6.2794E-06	2.69542E-06	PTS system, trehalose-specific IIA component [EC:2.7.1.201]
K02818	0.000216609	0.000213379	0.000198261	0.000140697	PTS system, trehalose-specific IIB component [EC:2.7.1.201]
K02819	0.000216609	0.000213379	0.000198261	0.000140697	PTS system, trehalose-specific IIC component
K02821	0.000217304	0.000216903	0.000218816	0.000215174	PTS system, ascorbate-specific IIA component [EC:2.7.1.194]
K02822	6.62058E-07	7.87606E-07	4.25059E-06	2.0208E-05	PTS system, ascorbate-specific IIB component [EC:2.7.1.194]
K02823	2.15725E-06	2.54884E-06	1.36598E-05	4.21009E-06	dihydroorotate dehydrogenase electron transfer subunit
K02824	8.03855E-06	1.18902E-05	4.46039E-05	9.79835E-05	uracil permease
K02825	0.00021864	0.000221569	0.000219122	0.000156515	pyrimidine operon attenuation protein / uracil phosphoribosyltransferase [EC:2.4.2.9]
K02826	1.00328E-06	2.44968E-07	2.8418E-06	5.75621E-08	cytochrome aa3-600 menaquinol oxidase subunit II [EC:1.10.3.12]
K02827	0.000648749	0.000644206	0.000602025	0.000503878	cytochrome aa3-600 menaquinol oxidase subunit I [EC:1.10.3.12]
K02828	1.18396E-06	4.35696E-07	3.5339E-06	1.3982E-07	cytochrome aa3-600 menaquinol oxidase subunit III [EC:1.10.3.12]
K02829	1.00328E-06	2.06813E-07	2.83698E-06	3.03626E-08	cytochrome aa3-600 menaquinol oxidase subunit IV [EC:1.10.3.12]
K02834	0.000223494	0.000223748	0.000227837	0.000225464	ribosome-binding factor A
K02835	0.000449131	0.000449716	0.000471854	0.000486064	peptide chain release factor 1
K02836	0.00044803	0.000447057	0.000455773	0.000451454	peptide chain release factor 2
K02837	0.000221704	0.000219828	0.00021795	0.000225118	peptide chain release factor 3
K02838	0.000223492	0.000223779	0.000228122	0.000225472	ribosome recycling factor
K02839	0.000218787	0.000216763	0.000207354	0.000150794	peptide chain release factor
K02840	9.19249E-07	8.96257E-07	1.45434E-06	9.39725E-07	UDP-D-galactose:(glucosyl)LPS alpha-1,6-D-galactosyltransferase [EC:2.4.1.-]
K02841	0.000428618	0.000425179	0.000375095	0.000230791	heptosyltransferase I [EC:2.4.-.-]
K02843	0.000429295	0.00042588	0.000377943	0.000243342	heptosyltransferase II [EC:2.4.-.-]
K02844	1.06146E-06	1.89261E-06	3.41444E-06	1.73021E-06	UDP-glucose:(heptosyl)LPS alpha-1,3-glucosyltransferase [EC:2.4.1.-]
K02846	1.17045E-06	3.85069E-07	3.4497E-07	5.27394E-06	N-methyl-L-tryptophan oxidase [EC:1.5.3.-]
K02847	0.000216435	0.000216312	0.00020765	0.000217339	O-antigen ligase [EC:2.4.1.-]
K02848	1.59757E-07	4.57297E-07	1.1761E-06	3.98261E-07	heptose I phosphotransferase [EC:2.7.1.-]
K02849	6.81077E-07	1.18196E-06	4.14698E-07	8.68981E-07	heptosyltransferase III [EC:2.4.-.-]
K02850	4.39368E-09	1.84114E-08	3.7531E-07	2.43127E-07	heptose II phosphotransferase [EC:2.7.1.-]
K02851	6.22544E-06	7.51081E-06	3.36058E-05	6.76162E-05	UDP-GlcNAc:undecaprenyl-phosphate/decaprenyl-phosphate GlcNAc-1-phosphate transferase [EC:2.7.8.33 2.7.8.35]
K02852	2.58996E-07	4.92544E-07	4.83317E-07	1.7852E-06	UDP-N-acetyl-D-mannosaminouronate:lipid I N-acetyl-D-mannosaminouronosyltransferase [EC:2.4.1.180]
K02853	9.82118E-08	3.01003E-07	7.49218E-08	7.99764E-07	enterobacterial common antigen polymerase [EC:2.4.1.-]
K02854	8.23907E-08	5.76411E-08	1.07505E-07	4.23661E-06	AraC family transcriptional regulator, L-rhamnose operon transcriptional activator RhaR

K02855	7.64838E-08	3.80054E-08	2.24905E-07	4.2201E-06	AraC family transcriptional regulator, L-rhamnose operon regulatory protein RhaS
K02856	2.44236E-07	6.97985E-07	6.12741E-07	1.4542E-06	L-rhamnose-H+ transport protein
K02858	0.000218334	0.000218218	0.00020345	0.000178538	3,4-dihydroxy 2-butanone 4-phosphate synthase [EC:4.1.99.12]
K02859	2.62557E-07	2.39461E-07	1.21949E-06	1.87659E-07	riboflavin biosynthesis RibT protein
K02860	0.000223381	0.000223531	0.000227376	0.000225425	16S rRNA processing protein RimM
K02862	1.46319E-07	2.10556E-07	8.34623E-07	1.90174E-09	putative membrane protein
K02863	0.000223481	0.000224146	0.00022825	0.000225453	large subunit ribosomal protein L1
K02864	0.000223732	0.000224343	0.000230825	0.00023421	large subunit ribosomal protein L10
K02867	0.000223451	0.000224143	0.000228394	0.000227131	large subunit ribosomal protein L11
K02871	0.000223492	0.000223748	0.000228032	0.00022441	large subunit ribosomal protein L13
K02874	0.000223481	0.000223748	0.000227864	0.000225444	large subunit ribosomal protein L14
K02875	8.67992E-08	0	0	0	large subunit ribosomal protein L14e
K02876	0.00022346	0.000223758	0.000227865	0.000225449	large subunit ribosomal protein L15
K02878	0.000223451	0.000223748	0.000228087	0.000226147	large subunit ribosomal protein L16
K02879	0.000223966	0.000224013	0.000233474	0.000224851	large subunit ribosomal protein L17
K02881	0.000223408	0.000223533	0.000227775	0.00022244	large subunit ribosomal protein L18
K02884	0.000223507	0.000223748	0.000227867	0.000225443	large subunit ribosomal protein L19
K02886	0.000223499	0.000223748	0.000228097	0.000225457	large subunit ribosomal protein L2
K02887	0.000224357	0.000226505	0.000228888	0.000225017	large subunit ribosomal protein L20
K02888	0.000223516	0.000223824	0.000228066	0.000225481	large subunit ribosomal protein L21
K02890	0.000223568	0.000223886	0.000228095	0.000225461	large subunit ribosomal protein L22
K02892	0.000223492	0.000223748	0.000228116	0.000225808	large subunit ribosomal protein L23
K02895	0.00022349	0.000223738	0.000227867	0.000225444	large subunit ribosomal protein L24
K02897	0.000222792	0.00022336	0.00022408	0.000222277	large subunit ribosomal protein L25
K02899	0.000223501	0.000223748	0.000228041	0.000225458	large subunit ribosomal protein L27
K02902	0.000224377	0.000224244	0.000234031	0.000227276	large subunit ribosomal protein L28
K02904	0.000223451	0.000223748	0.000228087	0.000224767	large subunit ribosomal protein L29
K02906	0.000223492	0.000223716	0.000228095	0.000225464	large subunit ribosomal protein L3
K02907	0.000223282	0.000223198	0.000225104	0.000225129	large subunit ribosomal protein L30
K02909	0.000224271	0.000224826	0.000235059	0.000241994	large subunit ribosomal protein L31
K02911	0.000224007	0.000223819	0.000233183	0.000227555	large subunit ribosomal protein L32
K02913	0.000225308	0.000224576	0.000240588	0.000230938	large subunit ribosomal protein L33
K02914	0.000222858	0.000222889	0.000223936	0.000221951	large subunit ribosomal protein L34
K02916	0.000223492	0.000223748	0.000228094	0.000225016	large subunit ribosomal protein L35
K02919	0.000224333	0.000224705	0.000246257	0.000264141	large subunit ribosomal protein L36
K02926	0.000223492	0.000224083	0.000228226	0.000225457	large subunit ribosomal protein L4
K02927	1.53799E-07	1.57232E-09	2.31539E-06	1.81305E-07	large subunit ribosomal protein L40e
K02931	0.000223492	0.000224136	0.00022803	0.000225448	large subunit ribosomal protein L5
K02933	0.000223492	0.000223748	0.000227929	0.000225444	large subunit ribosomal protein L6
K02935	0.000223577	0.000223479	0.000228238	0.000227294	large subunit ribosomal protein L7/L12
K02939	0.000223492	0.000223746	0.000228139	0.000225444	large subunit ribosomal protein L9
K02945	0.000227958	0.000225761	0.000233314	0.000233001	small subunit ribosomal protein S1
K02946	0.000223492	0.000223748	0.000228177	0.000225457	small subunit ribosomal protein S10
K02948	0.000223275	0.000223442	0.000227832	0.00022384	small subunit ribosomal protein S11
K02950	0.000223514	0.000223772	0.000228136	0.000227314	small subunit ribosomal protein S12
K02952	0.000223453	0.000223724	0.000228082	0.00022552	small subunit ribosomal protein S13
K02954	0.000225002	0.000223893	0.000237429	0.000228528	small subunit ribosomal protein S14
K02956	0.000223494	0.000223748	0.000228308	0.000223798	small subunit ribosomal protein S15
K02959	0.000223468	0.000223747	0.000227851	0.000225443	small subunit ribosomal protein S16
K02961	0.000223451	0.000223748	0.000228087	0.000225457	small subunit ribosomal protein S17
K02963	0.000223492	0.000223872	0.000228351	0.000227157	small subunit ribosomal protein S18
K02965	0.000223451	0.000223748	0.000228087	0.000225457	small subunit ribosomal protein S19
K02966	3.97169E-09	0	9.0419E-08	0	small subunit ribosomal protein S19e
K02967	0.000223492	0.000223788	0.000228108	0.000225515	small subunit ribosomal protein S2
K02968	0.000223391	0.000223464	0.000228111	0.000224282	small subunit ribosomal protein S20
K02970	0.000221954	0.000219671	0.000218132	0.000221417	small subunit ribosomal protein S21
K02972	2.83247E-08	1.94491E-08	5.47552E-08	7.28609E-07	stationary-phase-induced ribosome-associated protein
K02982	0.000223492	0.000223749	0.000228187	0.000225459	small subunit ribosomal protein S3
K02986	0.000223525	0.000223794	0.000227961	0.000225745	small subunit ribosomal protein S4
K02988	0.000223492	0.000223748	0.000227864	0.000225444	small subunit ribosomal protein S5
K02990	0.000223492	0.000223746	0.000228087	0.000225457	small subunit ribosomal protein S6
K02992	0.000223492	0.000223758	0.000228093	0.00022546	small subunit ribosomal protein S7
K02994	0.000223492	0.000223748	0.000227864	0.000225444	small subunit ribosomal protein S8
K02996	0.000223492	0.000223748	0.000228032	0.000225466	small subunit ribosomal protein S9
K03006	0	0	1.38479E-08	1.56369E-08	DNA-directed RNA polymerase II subunit RPB1 [EC:2.7.7.6]
K03040	0.000223517	0.000223739	0.000230648	0.000227325	DNA-directed RNA polymerase subunit alpha [EC:2.7.7.6]
K03041	1.10889E-07	1.99937E-08	2.09314E-08	0	DNA-directed RNA polymerase subunit A [EC:2.7.7.6]
K03042	2.62732E-06	5.45533E-06	1.15767E-05	5.84284E-06	DNA-directed RNA polymerase subunit A\ [EC:2.7.7.6]
K03043	0.000223517	0.000223851	0.000228217	0.0002258	DNA-directed RNA polymerase subunit beta [EC:2.7.7.6]
K03046	0.000223511	0.000223824	0.000228142	0.000226842	DNA-directed RNA polymerase subunit beta [EC:2.7.7.6]
K03048	1.30968E-06	3.44262E-07	6.79471E-06	3.64352E-06	DNA-directed RNA polymerase subunit delta
K03049	0	0	0	1.45357E-09	DNA-directed RNA polymerase subunit E [EC:2.7.7.6]
K03060	0.000222838	0.000222041	0.000222329	0.000225082	DNA-directed RNA polymerase subunit omega [EC:2.7.7.6]
K03070	0.000228604	0.000229325	0.00025729	0.000280123	preprotein translocase subunit SecA
K03071	0.000219358	0.000217129	0.000208023	0.000217748	preprotein translocase subunit SecB
K03072	0.000224518	0.000223786	0.000232842	0.000236817	preprotein translocase subunit SecD
K03073	0.000223451	0.000223681	0.000226735	0.000225426	preprotein translocase subunit SecE
K03074	0.00022288	0.000223158	0.000224107	0.000236586	preprotein translocase subunit SecF
K03075	0.000223213	0.000223333	0.000227644	0.000223534	preprotein translocase subunit SecG
K03076	0.000224489	0.000223914	0.000230942	0.000227477	preprotein translocase subunit SecY
K03077	2.2541E-06	2.19646E-06	1.52726E-05	2.05673E-05	L-ribulose-5-phosphate 4-epimerase [EC:5.1.3.4]
K03078	6.80969E-07	6.27611E-07	5.7067E-06	7.27805E-06	3-dehydro-L-gulonate-6-phosphate decarboxylase [EC:4.1.1.85]
K03079	8.43123E-07	9.21364E-07	5.9073E-06	7.31106E-06	L-ribulose-5-phosphate 3-epimerase [EC:5.1.3.22]
K03086	0.000440262	0.000439155	0.00042939	0.000370613	RNA polymerase primary sigma factor
K03087	0.000217384	0.000218538	0.000208746	0.000140818	RNA polymerase nonessential primary-like sigma factor
K03088	0.002248554	0.002286352	0.00248102	0.003210642	RNA polymerase sigma-70 factor, ECF subfamily
K03089	0.000434651	0.000429999	0.000396779	0.000340679	RNA polymerase sigma-32 factor

K03090	1.42418E-06	7.31931E-07	6.20288E-06	5.61606E-06	RNA polymerase sigma-B factor
K03091	5.70805E-06	4.09551E-06	1.26353E-05	9.83849E-07	RNA polymerase sporulation-specific sigma factor
K03092	0.000436339	0.000435357	0.000418415	0.00041916	RNA polymerase sigma-54 factor
K03093	1.14351E-07	5.35469E-08	4.78572E-07	1.09396E-09	RNA polymerase sigma factor
K03095	1.24018E-06	3.04561E-07	3.91216E-06	3.57361E-06	SprT-like protein
K03098	9.28536E-06	1.00248E-05	3.93362E-05	0.000295541	apolipoprotein D and lipocalin family protein
K03100	0.000447453	0.000448786	0.000459032	0.000454867	signal peptidase I [EC:3.4.21.89]
K03101	0.000224965	0.000226185	0.000238193	0.000271768	signal peptidase II [EC:3.4.23.36]
K03106	0.000225211	0.000228438	0.000238668	0.000228863	signal recognition particle subunit SRP54 [EC:3.6.5.4]
K03110	0.000223975	0.000225436	0.000230711	0.000232094	fused signal recognition particle receptor
K03111	0.000230671	0.000232684	0.000269601	0.000277163	single-strand DNA-binding protein
K03112	2.53557E-07	7.51755E-07	1.52972E-06	1.78285E-05	DamX protein
K03113	5.37556E-07	2.31052E-06	6.9487E-06	1.74359E-05	translation initiation factor 1
K03116	0.000223545	0.000225362	0.000224453	0.000243605	sec-independent protein translocase protein TatA
K03117	0.000222298	0.000221614	0.000218712	0.000221612	sec-independent protein translocase protein TatB
K03118	0.000223294	0.000223808	0.000222543	0.000224135	sec-independent protein translocase protein TatC
K03119	0.000219904	0.000224783	0.000248794	0.000269647	taurine dioxygenase [EC:1.14.11.17]
K03147	0.000221388	0.000222356	0.000213596	0.00021884	phosphomethylpyrimidine synthase [EC:4.1.99.17]
K03148	2.54582E-06	4.84328E-06	2.0134E-05	9.63997E-05	sulfur carrier protein ThiS adenylyltransferase [EC:2.7.7.73]
K03149	0.000222185	0.000222094	0.00022237	0.000220718	thiazole synthase [EC:2.8.1.10]
K03150	6.2531E-07	2.26997E-06	2.96665E-06	1.42085E-05	2-iminoacetate synthase [EC:4.1.99.19]
K03151	2.43384E-06	1.5994E-06	9.13535E-06	2.13933E-05	tRNA uracil 4-sulfurtransferase [EC:2.8.1.4]
K03152	1.26288E-06	1.89433E-06	1.35865E-05	1.94061E-05	protein deglycase [EC:3.5.1.124]
K03153	0.000219675	0.000219578	0.000217725	0.000158115	glycine oxidase [EC:1.4.3.19]
K03154	0.000222251	0.000222217	0.000223516	0.00021993	sulfur carrier protein
K03166	0	0	9.14913E-08	0	DNA topoisomerase VI subunit A [EC:5.99.1.3]
K03167	8.65761E-09	5.77828E-09	1.05446E-07	0	DNA topoisomerase VI subunit B [EC:5.99.1.3]
K03168	1.27171E-05	1.40441E-05	4.57354E-05	0.000179993	DNA topoisomerase I [EC:5.99.1.2]
K03169	0.000223066	0.000225721	0.000244456	0.000228061	DNA topoisomerase III [EC:5.99.1.2]
K03170	0	0	1.52294E-07	1.29419E-08	reverse gyrase [EC:5.99.1.3 3.6.4.12]
K03177	0.00022351	0.000223769	0.000227893	0.000225452	tRNA pseudouridine55 synthase [EC:5.4.99.25]
K03179	0.000219541	0.000219472	0.000208869	0.0002178	4-hydroxybenzoate polyprenyltransferase [EC:2.5.1.39]
K03181	0.000215373	0.000212717	0.000188979	0.000131085	chorismate--pyruvate lyase [EC:4.1.3.40]
K03182	0.00021621	0.000213741	0.000190631	0.000133524	4-hydroxy-3-polyprenylbenzoate decarboxylase [EC:4.1.1.98]
K03183	0.000873498	0.000865483	0.000805382	0.000616265	demethylmenaquinone methyltransferase / 2-methoxy-6-polyprenyl-1,4-benzoquinol methylase [EC:2.1.1.163 2.1.1.20
K03184	0.000216464	0.000214248	0.000190719	0.000193018	3-demethoxyubiquinol 3-hydroxylase [EC:1.14.99.60]
K03185	0.000439448	0.000435758	0.000409913	0.000459733	2-octaprenyl-6-methoxyphenol hydroxylase [EC:1.14.13.-]
K03186	0.000216472	0.00021332	0.000190267	0.000133647	flavin prenyltransferase [EC:2.5.1.129]
K03187	0.000220294	0.000218998	0.000210872	0.000151292	urease accessory protein
K03188	0.00021953	0.000218684	0.000210574	0.000152939	urease accessory protein
K03189	0.00021956	0.000218737	0.000210833	0.000157498	urease accessory protein
K03190	0.00021945	0.00021867	0.000210938	0.000153628	urease accessory protein
K03191	4.76303E-08	0	1.31208E-07	2.56735E-08	acid-activated urea channel
K03192	0.000217358	0.000215383	0.000203745	0.000151779	urease accessory protein
K03194	0.000214926	0.000213005	0.000190988	0.000159139	type IV secretion system protein VirB1
K03195	0.000215536	0.000212446	0.000188385	0.000153298	type IV secretion system protein VirB10
K03196	0.000431479	0.000428337	0.000391141	0.000355977	type IV secretion system protein VirB11
K03197	0.000215	0.000212411	0.000188008	0.000153251	type IV secretion system protein VirB2
K03198	0.000214972	0.000212417	0.000188016	0.000152142	type IV secretion system protein VirB3
K03199	0.000215357	0.000212484	0.000188552	0.000153366	type IV secretion system protein VirB4
K03200	0.000214067	0.000211985	0.000186855	0.000114471	type IV secretion system protein VirB5
K03201	0.000215343	0.000212631	0.000189933	0.000171524	type IV secretion system protein VirB6
K03202	0	7.63099E-08	3.29181E-08	0	type IV secretion system protein VirB7
K03203	0.000215454	0.000212444	0.000188223	0.000153297	type IV secretion system protein VirB8
K03204	0.000215531	0.000212441	0.000188384	0.000153308	type IV secretion system protein VirB9
K03205	1.12781E-05	8.0418E-06	3.25493E-05	9.48597E-05	type IV secretion system protein VirD4
K03206	9.88676E-07	2.18236E-09	2.70363E-06	2.23255E-08	azobenzene reductase [EC:1.7.1.6]
K03207	2.87664E-08	7.34922E-08	1.53625E-07	6.92536E-07	colanic acid biosynthesis protein WcaH [EC:3.6.1.-]
K03208	4.39235E-08	7.80563E-08	6.19215E-07	2.14468E-06	colanic acid biosynthesis glycosyl transferase WcaI
K03210	0.000223526	0.00022336	0.000224701	0.000225668	preprotein translocase subunit YajC
K03212	1.08034E-06	3.53459E-06	6.2231E-07	1.02387E-05	23S rRNA (uracil747-C5)-methyltransferase [EC:2.1.1.189]
K03214	1.09847E-06	1.50354E-06	1.78708E-06	5.25063E-05	RNA methyltransferase, TrmH family [EC:2.1.1.-]
K03215	0.000225932	0.000225174	0.000235818	0.000233806	23S rRNA (uracil1939-C5)-methyltransferase [EC:2.1.1.190]
K03216	0.00022273	0.000221588	0.000218667	0.000223303	tRNA (cytidine/uridine-2-O-)-methyltransferase [EC:2.1.1.207]
K03217	0.000224854	0.000224511	0.000234087	0.000239252	YidC/Oxa1 family membrane protein insertase
K03218	0.000227022	0.000231103	0.000244321	0.000277992	23S rRNA (guanosine2251-2-O)-methyltransferase [EC:2.1.1.185]
K03219	0.000214088	0.000211993	0.000189361	0.000116281	type III secretion protein C
K03220	0.00021383	0.000211852	0.00018902	0.00011372	type III secretion protein D
K03221	3.4306E-09	4.49674E-08	2.25999E-07	3.36213E-07	type III secretion protein F
K03222	0.000213851	0.000211909	0.000189283	0.000113746	type III secretion protein J
K03223	0.000213804	0.000212519	0.000189196	0.000113673	type III secretion protein L
K03224	0.000428738	0.000425782	0.000381379	0.000240052	ATP synthase in type III secretion protein N [EC:3.6.3.14]
K03225	0.000213814	0.000212567	0.000189797	0.00011392	type III secretion protein Q
K03226	0.000429576	0.00042626	0.000379592	0.000293676	type III secretion protein R
K03227	0.000215129	0.000212568	0.000189655	0.000115943	type III secretion protein S
K03228	0.000213851	0.000212544	0.000189531	0.000113786	type III secretion protein T
K03229	0.000215562	0.000213512	0.000190874	0.00011462	type III secretion protein U
K03230	0.000431268	0.000429296	0.00039626	0.000294108	type III secretion protein V
K03231	4.32763E-07	6.43307E-07	5.16585E-07	3.53945E-06	elongation factor 1-alpha
K03234	1.34865E-08	2.28391E-08	9.66106E-08	5.12435E-08	elongation factor 2
K03237	2.57365E-07	2.01453E-07	8.60314E-09	0	translation initiation factor 2 subunit 1
K03239	2.44485E-08	2.14069E-09	2.74136E-08	0	translation initiation factor eIF-2B subunit alpha
K03242	7.2419E-09	3.2516E-09	1.00868E-07	2.57382E-08	translation initiation factor 2 subunit 3
K03243	1.86304E-07	3.32617E-07	1.06841E-07	2.2042E-08	translation initiation factor 5B
K03265	0	0	0	2.82158E-08	peptide chain release factor subunit 1
K03268	7.94338E-09	1.77569E-10	1.47507E-07	8.14E-10	benzene/toluene/chlorobenzene dioxygenase subunit alpha [EC:1.14.12.3 1.14.12.11 1.14.12.-]
K03269	0.000217899	0.000218364	0.000209932	0.000217773	UDP-2,3-diacylglucosamine hydrolase [EC:3.6.1.54]

K03270	0.000217932	0.000218476	0.000210505	0.000218962	3-deoxy-D-manno-octulosonate 8-phosphate phosphatase (KDO 8-P phosphatase) [EC:3.1.3.45]
K03271	3.7661E-06	4.41331E-06	2.23057E-05	6.79963E-05	D-sedoheptulose 7-phosphate isomerase [EC:5.3.1.28]
K03272	0.000429459	0.000425147	0.000376952	0.000244752	D-beta-D-heptose 7-phosphate kinase / D-beta-D-heptose 1-phosphate adenosyltransferase [EC:2.7.1.167 2.7.7.70]
K03273	0.000217675	0.000216278	0.000207357	0.000168374	D-glycero-D-manno-heptose 1,7-bisphosphate phosphatase [EC:3.1.3.82 3.1.3.83]
K03274	0.000215899	0.000213908	0.000187849	0.000128606	ADP-L-glycero-D-manno-heptose 6-epimerase [EC:5.1.3.20]
K03275	1.52297E-07	7.87132E-08	7.47577E-08	6.38586E-07	UDP-glucose:(glucosyl)LPS alpha-1,3-glucosyltransferase [EC:2.4.1.-]
K03276	6.25341E-09	2.72452E-08	2.45305E-07	2.81079E-07	UDP-glucose/galactose:(glucosyl)LPS alpha-1,2-glucosyl/galactosyltransferase [EC:2.4.1.-]
K03277	1.17435E-09	2.92087E-09	8.45701E-09	6.05251E-08	heptosyltransferase IV [EC:2.4.-.-]
K03278	2.31586E-08	2.25892E-08	3.45199E-08	1.70561E-07	UDP-D-galactose:(glucosyl)LPS alpha-1,3-D-galactosyltransferase [EC:2.4.1.44]
K03279	2.85202E-09	1.03686E-08	2.05386E-08	1.71968E-07	UDP-glucose:(galactosyl)LPS alpha-1,2-glucosyltransferase [EC:2.4.1.58]
K03280	4.64547E-09	7.48808E-09	2.57016E-08	1.1162E-07	UDP-N-acetylglucosamine:(glucosyl)LPS alpha-1,2-N-acetylglucosaminyltransferase [EC:2.4.1.56]
K03281	0.00043488	0.000431971	0.00040513	0.000394089	chloride channel protein, ClC family
K03282	0.000223375	0.000223665	0.000225758	0.000212015	large conductance mechanosensitive channel
K03284	0.000443816	0.000442401	0.00046278	0.000408286	magnesium transporter
K03285	0.003219404	0.003206614	0.002937666	0.002025796	general bacterial porin, GBP family
K03286	0.000659407	0.000664784	0.000656252	0.000724625	OmpA-OmpF porin, OOP family
K03288	0.000430357	0.000425652	0.000377508	0.000235637	MFS transporter, MHS family, citrate/tricarballylate:H+ symporter
K03289	2.92316E-07	1.41959E-06	4.28202E-06	9.68391E-07	MFS transporter, NHS family, nucleoside permease
K03290	1.10619E-06	6.01655E-07	1.12909E-05	1.65574E-06	MFS transporter, SHS family, sialic acid transporter
K03291	2.04316E-07	6.25187E-07	5.88019E-07	4.84197E-06	MFS transporter, SET family, sugar efflux transporter
K03292	4.45096E-06	3.40837E-06	1.78467E-05	1.48273E-05	glycoside/pentoside/hexuronide:cation symporter, GPH family
K03293	0.000450476	0.000454739	0.000540032	0.000568436	amino acid transporter, AAT family
K03294	0.000442504	0.000434575	0.000405956	0.000458332	basic amino acid/polyamine antiporter, APA family
K03296	0.001760897	0.001758998	0.001703622	0.00192037	hydrophobic/amphiphilic exporter-1 (mainly G- bacteria), HAE1 family
K03297	7.07435E-06	8.08565E-06	2.63295E-05	0.000159651	small multidrug resistance pump
K03298	1.30039E-06	3.01189E-06	1.74239E-05	3.96075E-05	drug/metabolite transporter, DME family
K03299	0.000218827	0.000218388	0.000217322	0.000187381	gluconate:H+ symporter, GntP family
K03300	2.39882E-06	9.47591E-07	4.36014E-06	5.47111E-05	citrate-Mg2+:H+ or citrate-Ca2+:H+ symporter, CitMHS family
K03301	2.29421E-06	2.35756E-06	3.29374E-06	5.17621E-05	ATP:ADP antiporter, AAA family
K03303	0.000225869	0.000230466	0.000250491	0.000280788	lactate permease
K03304	1.2512E-07	1.59986E-07	3.63518E-07	8.07213E-07	tellurite resistance protein
K03305	8.37795E-06	1.29179E-05	2.26053E-05	0.000179713	proton-dependent oligopeptide transporter, POT family
K03306	0.000222235	0.000222541	0.000219555	0.00022571	inorganic phosphate transporter, PiT family
K03307	0.000442059	0.000447146	0.000456281	0.00040327	solute:Na+ symporter, SSS family
K03308	5.59666E-06	7.17798E-06	2.60741E-05	5.43022E-05	neurotransmitter:Na+ symporter, NSS family
K03309	0.000215768	0.000213744	0.00019104	0.000193479	dicarboxylate/amino acid:cation (Na+ or H+) symporter, DAACS family
K03310	1.45313E-05	2.0219E-05	3.24338E-05	0.000138434	alanine or glycine:cation symporter, AGCS family
K03311	5.46521E-06	8.81872E-06	3.47792E-05	5.70233E-05	branched-chain amino acid:cation transporter, LIVCS family
K03312	5.05583E-06	9.21559E-06	2.14445E-05	5.11363E-05	glutamate:Na+ symporter, ESS family
K03313	7.62921E-06	6.53898E-06	3.11675E-05	6.49175E-05	Na+:H+ antiporter, NhaA family
K03314	9.82928E-08	3.17605E-07	6.51836E-07	1.57721E-05	Na+:H+ antiporter, NhaB family
K03315	3.28319E-06	3.09402E-06	9.90899E-06	2.10452E-05	Na+:H+ antiporter, NhaC family
K03316	1.28148E-05	1.11967E-05	3.59762E-05	0.00013195	monovalent cation:H+ antiporter, CPA1 family
K03317	4.10765E-06	2.97634E-06	8.2166E-06	9.48455E-05	concentrative nucleoside transporter, CNT family
K03319	2.76828E-06	3.68557E-06	1.7818E-05	4.78226E-05	divalent anion:Na+ symporter, DASS family
K03320	0.000677398	0.000668858	0.000688476	0.000845804	ammonium transporter, Amt family
K03321	0.000228444	0.000227935	0.000238822	0.000313649	sulfate permease, SulP family
K03322	0.000220232	0.000220224	0.000215388	0.000215231	manganese transport protein
K03324	4.04832E-06	7.91424E-06	3.20395E-05	4.42281E-05	phosphate:Na+ symporter
K03325	5.94405E-06	1.13019E-05	1.90425E-05	0.000102349	arsenite transporter
K03326	1.09231E-07	7.36682E-07	6.21579E-06	1.63676E-05	C4-dicarboxylate transporter, DcuC family
K03327	0.000436864	0.000436575	0.000417053	0.000384052	multidrug resistance protein, MATE family
K03328	1.46194E-06	1.62459E-06	6.9906E-06	7.7206E-06	polysaccharide transporter, PST family
K03329	4.18334E-09	1.78882E-08	6.35382E-08	1.93635E-07	amino acid exporter
K03330	0	0	6.36425E-09	0	glutamyl-tRNA(Gln) amidotransferase subunit E [EC:6.3.5.7]
K03332	1.21389E-06	2.29558E-06	1.45125E-05	1.25249E-06	fructan beta-fructosidase [EC:3.2.1.80]
K03333	0.000216304	0.00021243	0.000188672	0.000122405	cholesterol oxidase [EC:1.1.3.6]
K03335	0.000429148	0.000426557	0.000388045	0.000254788	inosose dehydratase [EC:4.2.1.44]
K03336	0.000430595	0.000428624	0.000395366	0.000274236	3D-(3,5/4)-trihydroxycyclohexane-1,2-dione acylhydrolase (decyclizing) [EC:3.7.1.22]
K03337	0.000429511	0.000427533	0.000395003	0.00026669	5-deoxy-glucuronate isomerase [EC:5.3.1.30]
K03338	0.000430694	0.000428505	0.000395177	0.00031835	5-dehydro-2-deoxygluconokinase [EC:2.7.1.92]
K03339	1.20462E-06	2.35537E-07	3.56872E-06	5.15818E-07	6-phospho-5-dehydro-2-deoxy-D-gluconate aldolase [EC:4.1.2.29]
K03340	2.54183E-06	7.16016E-06	1.7594E-05	3.46559E-05	diaminopimelate dehydrogenase [EC:1.4.1.16]
K03342	0.000221032	0.000222736	0.000231451	0.000195944	para-aminobenzoate synthetase / 4-amino-4-deoxychorismate lyase [EC:2.6.1.85 4.1.3.38]
K03343	3.31846E-06	2.83458E-06	9.73452E-07	3.70464E-06	putrescine oxidase [EC:1.4.3.10]
K03346	1.30797E-06	3.44262E-07	7.01655E-06	4.00406E-06	replication initiation and membrane attachment protein
K03365	0	0	4.77318E-09	0	creatinine deaminase [EC:3.5.4.21]
K03366	6.19493E-06	4.1493E-06	2.74604E-05	6.76503E-05	meso-butanediol dehydrogenase / (S,S)-butanediol dehydrogenase / diacetyl reductase [EC:1.1.1.- 1.1.1.76 1.1.1.304]
K03367	0.000646845	0.000644533	0.000608774	0.000454756	D-alanine--poly(phosphoribitol) ligase subunit 1 [EC:6.1.1.13]
K03379	2.16193E-06	2.63907E-06	1.35989E-05	3.3953E-05	cyclohexanone monooxygenase [EC:1.14.13.22]
K03380	1.64415E-06	3.28931E-06	4.29992E-06	4.66909E-05	phenol 2-monooxygenase [EC:1.14.13.7]
K03381	1.68172E-06	3.55632E-06	1.57246E-05	3.10196E-05	catechol 1,2-dioxygenase [EC:1.13.11.1]
K03382	0.000215356	0.000214963	0.000200368	0.000148588	hydroxydechloroatrazine ethylaminohydrolase [EC:3.5.4.43]
K03383	1.01662E-06	9.29054E-08	6.74595E-07	2.00182E-06	cyanuric acid amidohydrolase [EC:3.5.2.15]
K03385	7.8409E-08	3.50969E-07	1.42441E-06	6.23013E-06	nitrite reductase (cytochrome c-552) [EC:1.7.2.2]
K03386	0.000872722	0.000863144	0.000813846	0.000692228	peroxiredoxin (alkyl hydroperoxide reductase subunit C) [EC:1.11.1.15]
K03387	0.000221133	0.000220369	0.000221533	0.00025791	alkyl hydroperoxide reductase subunit F [EC:1.6.4.-]
K03388	5.19025E-07	1.05838E-06	7.56508E-06	3.31444E-06	heterodisulfide reductase subunit A2 [EC:1.8.7.3 1.8.98.4 1.8.98.5 1.8.98.6]
K03389	6.54055E-09	0	5.49817E-07	0	heterodisulfide reductase subunit B2 [EC:1.8.7.3 1.8.98.4 1.8.98.5 1.8.98.6]
K03390	0	0	4.89888E-07	0	heterodisulfide reductase subunit C2 [EC:1.8.7.3 1.8.98.4 1.8.98.5 1.8.98.6]
K03392	3.59646E-07	1.27532E-06	3.06393E-06	2.65535E-06	aminocarboxymuconate-semialdehyde decarboxylase [EC:4.1.1.45]
K03394	0.000220617	0.000216714	0.000218177	0.000155387	precorrin-2/cobalt-factor-2 C20-methyltransferase [EC:2.1.1.130 2.1.1.151]
K03395	1.07575E-07	5.30836E-09	5.38846E-07	1.37245E-07	aminoglycoside 3-N-acetyltransferase I [EC:2.3.1.60]
K03396	1.93609E-07	9.46795E-08	4.04887E-07	1.87772E-06	S-(hydroxymethyl)glutathione synthase [EC:4.4.1.22]
K03397	3.61085E-10	4.61751E-10	1.06547E-08	0	indoleacetate---lysine synthetase [EC:6.3.2.20]
K03399	0.000213957	0.00021182	0.000186515	0.000114427	cobalt-precorrin-7 (C5)-methyltransferase [EC:2.1.1.289]
K03400	3.1635E-09	0	9.89572E-09	1.58716E-07	long-chain-fatty-acyl-CoA reductase [EC:1.2.1.50]

K03402	4.76444E-06	6.34738E-06	2.30229E-05	3.83307E-05	transcriptional regulator of arginine metabolism
K03403	1.02166E-06	2.65765E-08	6.88118E-07	2.73164E-07	magnesium chelatase subunit H [EC:6.6.1.1]
K03404	0.000219248	0.000216147	0.00022562	0.000150609	magnesium chelatase subunit D [EC:6.6.1.1]
K03405	0.000217823	0.000217363	0.00022092	0.000152359	magnesium chelatase subunit I [EC:6.6.1.1]
K03406	0.004614981	0.004576833	0.004551933	0.004716846	methyl-accepting chemotaxis protein
K03407	0.000447735	0.000442875	0.000451439	0.00059931	two-component system, chemotaxis family, sensor kinase CheA [EC:2.7.13.3]
K03408	0.000227819	0.000224255	0.000238806	0.000374487	purine-binding chemotaxis protein CheW
K03409	8.77679E-07	4.88567E-07	1.39905E-06	1.64799E-05	chemotaxis protein CheX
K03410	9.84846E-07	5.18415E-07	1.38033E-06	1.30023E-05	chemotaxis protein CheC
K03411	0.000217339	0.000216789	0.000209374	0.000218958	chemotaxis protein CheD [EC:3.5.1.44]
K03412	0.000233611	0.000231542	0.000264776	0.000394563	two-component system, chemotaxis family, protein-glutamate methylesterase/glutaminase [EC:3.1.1.61 3.5.1.44]
K03413	0.000667273	0.000659909	0.000656748	0.000829879	two-component system, chemotaxis family, chemotaxis protein CheY
K03414	0.0004345	0.000428705	0.000391814	0.00033047	chemotaxis protein CheZ
K03415	0.000217357	0.000219541	0.000208071	0.000260599	two-component system, chemotaxis family, chemotaxis protein CheV
K03416	1.07052E-06	9.43853E-07	7.2355E-06	8.07881E-06	methylmalonyl-CoA carboxyltransferase 5S subunit [EC:2.1.3.1]
K03417	0.000434547	0.000435416	0.000408776	0.000374682	methylisocitrate lyase [EC:4.1.3.30]
K03418	2.97893E-08	2.27774E-07	4.40059E-07	1.90612E-07	N,N-dimethylformamidase [EC:3.5.1.56]
K03420	2.92123E-06	6.59957E-06	1.05125E-05	2.45851E-06	proteasome regulatory subunit
K03424	0.000442546	0.000444494	0.000440506	0.000469564	TatD DNase family protein [EC:3.1.21.-]
K03425	1.68107E-07	5.96155E-07	3.48617E-07	1.64568E-05	sec-independent protein translocase protein TatE
K03426	9.81016E-06	1.4667E-05	4.83646E-05	0.000150451	NAD+ diphosphatase [EC:3.6.1.22]
K03427	0.00064945	0.000647995	0.000596122	0.000433185	type I restriction enzyme M protein [EC:2.1.1.72]
K03428	1.03907E-06	3.39455E-07	4.57975E-07	1.98147E-07	magnesium-protoporphyrin O-methyltransferase [EC:2.1.1.11]
K03429	1.48346E-06	3.74493E-07	6.37469E-06	2.58264E-07	processive 1,2-diacylglycerol beta-glucosyltransferase [EC:2.4.1.315]
K03430	1.06126E-06	1.52303E-06	1.44252E-06	4.86488E-06	2-aminoethylphosphonate-pyruvate transaminase [EC:2.6.1.37]
K03431	0.000226451	0.000226177	0.000235288	0.00023505	phosphoglucosamine mutase [EC:5.4.2.10]
K03432	5.4988E-07	5.44503E-07	6.04388E-06	1.95489E-06	proteasome alpha subunit [EC:3.4.25.1]
K03433	5.4988E-07	5.44503E-07	6.54683E-06	1.95489E-06	proteasome beta subunit [EC:3.4.25.1]
K03435	0.000218058	0.000218995	0.000220589	0.00022515	LacI family transcriptional regulator, fructose operon transcriptional repressor
K03436	5.88596E-06	5.33254E-06	3.43702E-05	2.77705E-05	DeoR family transcriptional regulator, fructose operon transcriptional repressor
K03437	0.000223618	0.000227176	0.000238221	0.000218182	RNA methyltransferase, TrmH family
K03438	0.000437775	0.00043603	0.000417961	0.000339434	16S rRNA (cytosine1402-N4)-methyltransferase [EC:2.1.1.199]
K03439	0.000223508	0.000223888	0.000228084	0.000225606	tRNA (guanine-N7-)-methyltransferase [EC:2.1.1.33]
K03442	0.000216177	0.000216657	0.000205736	0.000201676	small conductance mechanosensitive channel
K03444	1.96455E-07	8.48607E-07	1.80706E-07	8.14051E-07	MFS transporter, SP family, sugar porter, other
K03445	7.68956E-07	8.50377E-07	2.59389E-06	3.38939E-05	MFS transporter, DHA1 family, purine ribonucleoside efflux pump
K03446	0.001520262	0.001501534	0.00139772	0.001115142	MFS transporter, DHA2 family, multidrug resistance protein
K03449	0.000221092	0.00022084	0.000210253	0.000237021	MFS transporter, CP family, cyanate transporter
K03451	1.1188E-05	1.70758E-05	3.4541E-05	0.000231768	betaine/carnitine transporter, BCCT family
K03453	2.49598E-06	5.26914E-06	9.50127E-06	1.04352E-05	bile acid:Na+ symporter, BASS family
K03455	0.000655404	0.000654173	0.000619582	0.000594131	monovalent cation:H+ antiporter-2, CPA2 family
K03457	0.000221511	0.000223393	0.000250593	0.00024411	nucleobase:cation symporter-1, NCS1 family
K03458	0.000223757	0.000232729	0.000274331	0.00029819	nucleobase:cation symporter-2, NCS2 family
K03459	3.98104E-09	1.31768E-08	2.86691E-08	2.93982E-06	formate transporter
K03462	0.000215429	0.000212173	0.000188368	0.000142539	nicotinamide phosphoribosyltransferase [EC:2.4.2.12]
K03464	1.33696E-06	3.91001E-06	1.76301E-05	3.75932E-05	muconolactone D-isomerase [EC:5.3.3.4]
K03465	5.4331E-07	8.26149E-07	3.51312E-06	2.01871E-06	thymidylate synthase (FAD) [EC:2.1.1.148]
K03466	0.000443318	0.000441199	0.000437936	0.00035032	DNA segregation ATPase FtsK/SpoIIIE, S-DNA-T family
K03468	9.82847E-08	3.01184E-07	8.12613E-08	8.01925E-07	p-hydroxybenzoic acid efflux pump subunit AaeB
K03469	0.000226704	0.000227418	0.000243414	0.000305329	ribonuclease HI [EC:3.1.26.4]
K03470	0.000223701	0.000223789	0.000228272	0.000227338	ribonuclease HII [EC:3.1.26.4]
K03471	1.2358E-06	3.62158E-07	6.42832E-06	3.76557E-06	ribonuclease HIII [EC:3.1.26.4]
K03472	2.06817E-06	1.19774E-06	4.36258E-06	2.23074E-05	D-erythrose 4-phosphate dehydrogenase [EC:1.2.1.72]
K03473	1.73808E-06	1.535E-06	5.71629E-06	1.7823E-05	erythronate-4-phosphate dehydrogenase [EC:1.1.1.290]
K03474	0.000219614	0.000218804	0.000210754	0.000217666	pyridoxine 5-phosphate synthase [EC:2.6.99.2]
K03475	1.20008E-06	1.42239E-06	1.10687E-05	2.07093E-05	PTS system, ascorbate-specific IIC component
K03476	1.68699E-07	2.00207E-07	2.20845E-07	6.92544E-06	L-ascorbate 6-phosphate lactonase [EC:3.1.1.-]
K03477	6.87072E-07	6.79253E-07	6.38566E-06	1.21472E-05	DeoR family transcriptional regulator, ulaG and ulaABCDEF operon transcriptional repressor
K03478	1.86338E-06	3.72631E-06	1.92732E-05	3.65425E-05	chitin disaccharide deacetylase [EC:3.5.1.105]
K03480	1.07177E-06	4.80849E-07	3.32993E-06	2.38589E-07	transcriptional antiterminator
K03481	1.06762E-06	5.80069E-08	3.22649E-06	5.87575E-07	RpiR family transcriptional regulator, glv operon transcriptional regulator
K03482	9.05876E-08	3.83381E-08	2.93383E-07	1.66901E-06	GntR family transcriptional regulator, glv operon transcriptional regulator
K03483	4.82818E-07	4.73676E-07	3.90684E-06	2.7281E-06	mannitol operon transcriptional antiterminator
K03484	0.000215948	0.000212693	0.000192648	0.000141275	LacI family transcriptional regulator, sucrose operon repressor
K03485	1.86617E-07	3.53163E-07	6.81115E-07	1.79635E-05	LacI family transcriptional regulator, trehalose operon repressor
K03486	7.17117E-07	7.82174E-07	4.60888E-06	2.11489E-06	GntR family transcriptional regulator, trehalose operon transcriptional repressor
K03487	5.8129E-07	7.1819E-07	1.48817E-06	2.39317E-05	LacI family transcriptional regulator, asc operon repressor
K03488	1.49275E-06	1.15242E-06	1.04253E-05	4.62399E-06	beta-glucoside operon transcriptional antiterminator
K03489	1.38088E-07	3.20543E-08	3.26759E-07	1.95557E-07	GntR family transcriptional regulator, transcriptional regulator of bglA
K03490	9.82474E-08	3.01092E-07	4.01887E-07	1.43182E-06	AraC family transcriptional regulator, dual regulator of chb operon
K03491	1.08382E-06	1.11657E-06	2.0604E-05	4.65353E-06	lichenan operon transcriptional antiterminator
K03492	4.22881E-07	7.85959E-08	3.32265E-06	2.27273E-06	GntR family transcriptional regulator
K03493	6.22638E-08	0	6.96678E-08	3.99974E-07	transcriptional antiterminator
K03495	0.000221974	0.0002205	0.000219517	0.000221724	tRNA uridine 5-carboxymethylaminomethyl modification enzyme
K03496	0.00111152	0.001101393	0.00107892	0.000987914	chromosome partitioning protein
K03497	0.00109415	0.001085903	0.001029917	0.000800135	chromosome partitioning protein, ParB family
K03498	1.13641E-05	1.64007E-05	4.88488E-05	8.96057E-05	trk system potassium uptake protein
K03499	1.32637E-05	2.23945E-05	6.17711E-05	7.85714E-05	trk system potassium uptake protein
K03500	0.000655031	0.000651917	0.000622055	0.000499912	16S rRNA (cytosine967-C5)-methyltransferase [EC:2.1.1.176]
K03501	0.000223855	0.000223729	0.000232852	0.000225492	16S rRNA (guanine527-N7)-methyltransferase [EC:2.1.1.170]
K03502	7.03384E-06	3.34206E-06	1.37241E-05	4.13548E-05	DNA polymerase V
K03503	2.89816E-06	2.09963E-06	4.52975E-06	2.29824E-05	DNA polymerase V [EC:3.4.21.-]
K03516	9.46829E-08	3.15431E-07	9.47625E-08	7.9272E-07	flagellar protein FlhE
K03517	0.000219966	0.000220725	0.000212694	0.00017053	quinolinate synthase [EC:2.5.1.72]
K03518	0.000229059	0.000228501	0.000260517	0.000309682	aerobic carbon-monoxide dehydrogenase small subunit [EC:1.2.5.3]
K03519	0.000218443	0.000217312	0.000211657	0.000174538	aerobic carbon-monoxide dehydrogenase medium subunit [EC:1.2.5.3]
K03520	0.000221059	0.000221727	0.000230587	0.000219208	aerobic carbon-monoxide dehydrogenase large subunit [EC:1.2.5.3]

K03521	0.00022573	0.000228596	0.00024821	0.000262707	electron transfer flavoprotein beta subunit
K03522	0.00022599	0.000228804	0.000251569	0.000262936	electron transfer flavoprotein alpha subunit
K03523	5.03084E-06	8.37115E-06	2.96659E-05	4.27792E-05	biotin transport system substrate-specific component
K03524	0.000224967	0.000224425	0.000225171	0.00022607	BirA family transcriptional regulator, biotin operon repressor / biotin---[acetyl-CoA-carboxylase] ligase [EC:6.3.4.15]
K03525	0.000219588	0.00021946	0.000215341	0.000205913	type III pantothenate kinase [EC:2.7.1.33]
K03526	0.000222251	0.000222892	0.000224697	0.000222093	(E)-4-hydroxy-3-methylbut-2-enyl-diphosphate synthase [EC:1.17.7.1 1.17.7.3]
K03527	0.000224577	0.000224029	0.000227084	0.000222313	4-hydroxy-3-methylbut-2-en-1-yl diphosphate reductase [EC:1.17.7.4]
K03528	2.58491E-06	1.99578E-06	3.90943E-06	6.9124E-05	cell division protein ZipA
K03529	0.000220781	0.000218755	0.000219289	0.000218102	chromosome segregation protein
K03530	0.00086836	0.000863425	0.000802752	0.000584886	DNA-binding protein HU-beta
K03531	0.000223516	0.000223804	0.000228324	0.0002263	cell division protein FtsZ
K03532	1.18923E-07	1.67722E-06	1.80624E-06	2.32502E-05	trimethylamine-N-oxide reductase (cytochrome c), cytochrome c-type subunit TorC
K03533	4.61566E-09	7.31863E-08	4.01909E-07	1.30822E-05	TorA specific chaperone
K03534	1.36889E-06	2.89067E-06	7.54815E-06	1.19275E-05	L-rhamnose mutarotase [EC:5.1.3.32]
K03535	0.00043155	0.000423931	0.000376565	0.00023326	MFS transporter, ACS family, glucarate transporter
K03536	0.000223182	0.000223269	0.000227649	0.000225165	ribonuclease P protein component [EC:3.1.26.5]
K03543	0.000662429	0.000651378	0.000616188	0.000763466	membrane fusion protein, multidrug efflux system
K03544	0.000224212	0.000224164	0.000226534	0.000226871	ATP-dependent Clp protease ATP-binding subunit ClpX
K03545	0.000224621	0.000225199	0.000231445	0.000239333	trigger factor
K03546	6.81529E-06	8.24357E-06	2.99266E-05	5.77758E-05	DNA repair protein SbcC/Rad50
K03547	8.25112E-06	8.55007E-06	3.16089E-05	5.72107E-05	DNA repair protein SbcD/Mre11
K03548	8.48833E-07	9.42585E-07	3.08872E-06	6.90122E-05	putative permease
K03549	0.000436381	0.000430809	0.000407196	0.000326594	KUP system potassium uptake protein
K03550	0.000223559	0.000223833	0.000228519	0.000227136	holliday junction DNA helicase RuvA [EC:3.6.4.12]
K03551	0.000225076	0.000224058	0.000228312	0.000227588	holliday junction DNA helicase RuvB [EC:3.6.4.12]
K03553	0.000223512	0.000223877	0.000230533	0.000225483	recombination protein RecA
K03554	0.000217807	0.000219423	0.000218256	0.000260459	recombination associated protein RdgC
K03555	0.000222142	0.000220013	0.000220318	0.000221677	DNA mismatch repair protein MutS
K03556	0.000651827	0.00065511	0.000622118	0.000506322	LuxR family transcriptional regulator, maltose regulon positive regulatory protein
K03557	0.000217675	0.000217093	0.000207334	0.000217791	Fis family transcriptional regulator, factor for inversion stimulation protein
K03558	0.000219503	0.000217881	0.000208744	0.000212509	membrane protein required for colicin V production
K03559	0.000447506	0.000446698	0.000463491	0.000693139	biopolymer transport protein ExbD
K03560	0.000440423	0.000436189	0.000421108	0.000569742	biopolymer transport protein TolR
K03561	0.000442935	0.000447371	0.000447717	0.000634396	biopolymer transport protein ExbB
K03562	0.000438406	0.000433884	0.000416559	0.000422823	biopolymer transport protein TolQ
K03563	3.03643E-06	2.50504E-06	5.81259E-06	7.00086E-05	carbon storage regulator
K03564	0.000440572	0.000443735	0.000433698	0.000398034	peroxiredoxin Q/BCP [EC:1.11.1.15]
K03565	0.000222254	0.000223199	0.000226952	0.000228598	regulatory protein
K03566	0.003068251	0.003095901	0.003195997	0.003635959	LysR family transcriptional regulator, glycine cleavage system transcriptional activator
K03567	1.3147E-06	2.02601E-06	3.50899E-06	6.87415E-05	glycine cleavage system transcriptional repressor
K03568	0.000219671	0.000219748	0.000215592	0.000327036	TldD protein
K03569	0.000221286	0.000222401	0.000229199	0.000240888	rod shape-determining protein MreB and related proteins
K03570	0.000222197	0.000219907	0.000219811	0.000223196	rod shape-determining protein MreC
K03571	0.000219722	0.000217662	0.000211796	0.000219038	rod shape-determining protein MreD
K03572	0.000222488	0.000220466	0.000219462	0.000233578	DNA mismatch repair protein MutL
K03573	5.23065E-07	3.19102E-07	5.39642E-07	1.74156E-05	DNA mismatch repair protein MutH
K03574	0.000896593	0.000899033	0.00090651	0.00097275	8-oxo-dGTP diphosphatase [EC:3.6.1.55]
K03575	0.000223599	0.00022402	0.000229467	0.000227352	A/G-specific adenine glycosylase [EC:3.2.2.31]
K03576	0.000651457	0.000650349	0.000623612	0.000735221	LysR family transcriptional regulator, regulator for metE and metH
K03577	0.000646674	0.000646083	0.000598373	0.000505105	TetR/AcrR family transcriptional regulator, acrAB operon repressor
K03578	0.0002206	0.00022302	0.000218286	0.000268536	ATP-dependent helicase HrpA [EC:3.6.4.13]
K03579	5.63674E-06	8.15958E-06	1.95061E-05	8.72256E-05	ATP-dependent helicase HrpB [EC:3.6.4.13]
K03580	1.85092E-06	1.25654E-06	3.75479E-06	2.21382E-05	ATP-dependent helicase HepA [EC:3.6.4.-]
K03581	0.000223351	0.000221499	0.000224126	0.000235741	exodeoxyribonuclease V alpha subunit [EC:3.1.11.5]
K03582	5.50274E-06	6.97544E-06	2.59194E-05	0.000111736	exodeoxyribonuclease V beta subunit [EC:3.1.11.5]
K03583	4.44026E-06	5.59852E-06	2.4559E-05	0.000105816	exodeoxyribonuclease V gamma subunit [EC:3.1.11.5]
K03584	0.000223304	0.000223541	0.000228281	0.000225445	DNA repair protein RecO (recombination protein O)
K03585	0.00066885	0.00066979	0.000676357	0.001015634	membrane fusion protein, multidrug efflux system
K03586	0.000217642	0.00021692	0.000206951	0.000217464	cell division protein FtsL
K03587	0.000223638	0.000226197	0.00022635	0.000237279	cell division protein FtsI (penicillin-binding protein 3) [EC:3.4.16.4]
K03588	0.000442292	0.000443916	0.000432939	0.000350928	cell division protein FtsW
K03589	0.000222956	0.000222954	0.000224783	0.000225733	cell division protein FtsQ
K03590	0.000222769	0.00022004	0.000218654	0.000221434	cell division protein FtsA
K03591	9.82118E-08	3.15443E-07	2.90783E-07	3.89675E-06	cell division protein FtsN
K03592	0.000217295	0.000217213	0.000210993	0.000220574	PmbA protein
K03593	0.000441808	0.00044031	0.000434954	0.000429692	ATP-binding protein involved in chromosome partitioning
K03594	0.000225559	0.00022649	0.000245983	0.000391047	bacterioferritin [EC:1.16.3.1]
K03595	0.000224538	0.000224251	0.000229972	0.000237518	GTPase
K03596	0.000223751	0.000224606	0.000230668	0.000227611	GTP-binding protein LepA
K03597	0.000216187	0.000216243	0.000205912	0.000217282	sigma-E factor negative regulatory protein RseA
K03598	0.000213982	0.000212737	0.000190852	0.000131282	sigma-E factor negative regulatory protein RseB
K03599	0.000217877	0.000217087	0.000207579	0.000228739	stringent starvation protein A
K03600	0.000217642	0.00021692	0.00020717	0.000217494	stringent starvation protein B
K03601	0.000224945	0.000222969	0.000227803	0.00022564	exodeoxyribonuclease VII large subunit [EC:3.1.11.6]
K03602	0.000223399	0.000222963	0.000225921	0.000225454	exodeoxyribonuclease VII small subunit [EC:3.1.11.6]
K03603	1.11378E-07	3.91395E-07	5.64355E-07	1.74472E-05	GntR family transcriptional regulator, negative regulator for fad regulon and positive regulator of fabA
K03604	0.000214078	0.000212209	0.000190279	0.000137669	LacI family transcriptional regulator, purine nucleotide synthesis repressor
K03605	7.23564E-07	7.08668E-07	1.65762E-06	6.08357E-06	hydrogenase maturation protease [EC:3.4.23.-]
K03606	0.000227606	0.000227148	0.000221521	0.0001639	putative colanic acid biosynthesis UDP-glucose lipid carrier transferase
K03607	3.53313E-07	4.47684E-07	1.60518E-06	1.75843E-05	ProP effector
K03608	0.000218722	0.000217131	0.000206412	0.000216895	cell division topological specificity factor
K03609	0.000220416	0.000220656	0.000209116	0.000217305	septum site-determining protein MinD
K03610	0.000219848	0.000217296	0.000206973	0.000217071	septum site-determining protein MinC
K03611	0.000217915	0.000217174	0.000208617	0.000217902	disulfide bond formation protein DsbB
K03612	1.56377E-06	2.24535E-06	8.24459E-06	1.87207E-05	electron transport complex protein RnfG
K03613	1.65067E-06	2.86236E-06	8.15822E-06	2.04429E-05	electron transport complex protein RnfE
K03614	1.50099E-06	3.32058E-06	8.67868E-06	1.79824E-05	electron transport complex protein RnfD

K03615	1.88416E-06	2.62302E-06	8.67339E-06	1.95778E-05	electron transport complex protein RnfC
K03616	0.000219444	0.000218832	0.000214959	0.000218902	electron transport complex protein RnfB
K03617	1.61006E-06	2.22972E-06	9.0716E-06	1.86163E-05	electron transport complex protein RnfA
K03618	2.35009E-07	5.87455E-08	3.8783E-07	3.9945E-07	hydrogenase-1 operon protein HyaF
K03619	2.25278E-07	4.33851E-08	3.14463E-07	2.99926E-07	hydrogenase-1 operon protein HyaE
K03620	2.99138E-07	4.74438E-07	6.35222E-07	1.78895E-06	Ni/Fe-hydrogenase 1 B-type cytochrome subunit
K03621	0.000219652	0.000217164	0.000211151	0.000170015	glycerol-3-phosphate acyltransferase PlsX [EC:2.3.1.15]
K03623	1.5237E-07	5.80393E-07	4.9338E-07	2.72416E-06	ribonuclease inhibitor
K03624	0.000225637	0.000225126	0.000230011	0.000226835	transcription elongation factor GreA
K03625	0.000223492	0.000223729	0.000228113	0.000225456	N utilization substance protein B
K03627	0	2.70375E-09	2.27787E-08	0	putative transcription factor
K03628	0.000222597	0.000223586	0.000224245	0.000225177	transcription termination factor Rho
K03629	9.21582E-06	9.78712E-06	2.84059E-05	0.000111306	DNA replication and repair protein RecF
K03630	0.000225	0.000225928	0.000240051	0.000296525	DNA repair protein RadC
K03631	0.000223521	0.000223774	0.000228162	0.000226576	DNA repair protein RecN (Recombination protein N)
K03632	1.06111E-07	3.46343E-07	6.02716E-07	1.22986E-05	chromosome partition protein MukB
K03633	9.96404E-08	3.20272E-07	6.00864E-07	1.22967E-05	chromosome partition protein MukF
K03634	0.000218073	0.000217017	0.000207438	0.000217585	outer membrane lipoprotein carrier protein
K03635	0.000221473	0.000218119	0.000218916	0.00021001	molybdopterin synthase catalytic subunit [EC:2.8.1.12]
K03636	0.000221495	0.000218324	0.00021996	0.000210108	sulfur-carrier protein
K03637	0.000222598	0.000219454	0.000219557	0.000212542	cyclic pyranopterin monophosphate synthase [EC:4.6.1.17]
K03638	5.88963E-06	2.8641E-06	1.46152E-05	1.59073E-05	molybdopterin adenylyltransferase [EC:2.7.7.75]
K03639	0.000222565	0.000218798	0.000224251	0.000212408	GTP 3,8-cyclase [EC:4.1.99.22]
K03640	0.000222566	0.000223993	0.000239317	0.000294957	peptidoglycan-associated lipoprotein
K03641	0.000224044	0.000222994	0.000226802	0.000286857	TolB protein
K03642	0.000226535	0.000222086	0.000232448	0.000338702	rare lipoprotein A
K03643	0.000219352	0.000217113	0.000207751	0.000217636	LPS-assembly lipoprotein
K03644	0.000222533	0.000223149	0.000222173	0.000222671	lipoyl synthase [EC:2.8.1.8]
K03645	1.0124E-07	3.14658E-07	3.62733E-07	1.71559E-05	negative modulator of initiation of replication
K03646	0.000220283	0.000217634	0.000208804	0.000217998	colicin import membrane protein
K03647	2.38815E-06	3.89703E-06	6.8643E-06	1.0266E-05	protein involved in ribonucleotide reduction
K03648	0.000222156	0.000224388	0.000226413	0.000225231	uracil-DNA glycosylase [EC:3.2.2.27]
K03649	0.000217736	0.000220041	0.000204079	0.000201395	double-stranded uracil-DNA glycosylase [EC:3.2.2.28]
K03650	0.000223995	0.00022027	0.000224506	0.000224057	tRNA modification GTPase [EC:3.6.-.-]
K03651	0.000644425	0.000639985	0.00058308	0.000398869	3,5-cyclic-AMP phosphodiesterase [EC:3.1.4.53]
K03652	5.26509E-06	5.78031E-06	1.57479E-05	4.61284E-05	DNA-3-methyladenine glycosylase [EC:3.2.2.21]
K03653	0	0	1.92766E-07	1.85931E-08	N-glycosylase/DNA lyase [EC:3.2.2.- 4.2.99.18]
K03654	0.000225423	0.000227512	0.00023863	0.000238168	ATP-dependent DNA helicase RecQ [EC:3.6.4.12]
K03655	0.00022948	0.000231405	0.000261458	0.000254187	ATP-dependent DNA helicase RecG [EC:3.6.4.12]
K03656	0.000219142	0.00022059	0.000220961	0.000245429	ATP-dependent DNA helicase Rep [EC:3.6.4.12]
K03657	0.000879342	0.000882555	0.000864036	0.000750254	DNA helicase II / ATP-dependent DNA helicase PcrA [EC:3.6.4.12]
K03658	2.80709E-06	1.40208E-06	4.33891E-06	7.32515E-05	DNA helicase IV [EC:3.6.4.12]
K03660	5.60078E-07	2.96102E-07	7.52235E-07	6.62358E-08	N-glycosylase/DNA lyase [EC:3.2.2.- 4.2.99.18]
K03664	0.000223507	0.000224001	0.00022793	0.000225447	SsrA-binding protein
K03665	0.000440702	0.000437451	0.000416401	0.000371575	GTPase
K03666	0.000220866	0.000217626	0.000212131	0.000220868	host factor-I protein
K03667	0.000219934	0.000218884	0.000211437	0.0002192	ATP-dependent HslUV protease ATP-binding subunit HslU
K03668	1.02399E-07	7.4168E-07	1.72164E-06	1.72396E-05	heat shock protein HslJ
K03669	0.000217268	0.000213296	0.000191697	0.000170015	membrane glycosyltransferase [EC:2.4.1.-]
K03670	0.000645696	0.000636775	0.000563105	0.000397804	periplasmic glucans biosynthesis protein
K03671	0.000880013	0.000896594	0.000883927	0.000928488	thioredoxin 1
K03672	0.000432648	0.000433286	0.000402214	0.000348864	thioredoxin 2 [EC:1.8.1.8]
K03673	0.000432901	0.00043049	0.000395758	0.000389334	thiol:disulfide interchange protein DsbA
K03674	9.82677E-08	3.1472E-07	3.65666E-07	1.7136E-05	glutaredoxin 1
K03675	1.24827E-07	3.13655E-07	1.57493E-07	6.24554E-06	glutaredoxin 2
K03676	0.000219615	0.000217478	0.000208594	0.000225083	glutaredoxin 3
K03679	4.21341E-11	1.04797E-10	1.88576E-07	1.24811E-09	exosome complex component RRP4
K03680	0	0	0	5.70522E-09	translation initiation factor eIF-2B subunit delta
K03683	2.45335E-06	1.99578E-06	3.90943E-06	6.9124E-05	ribonuclease T [EC:3.1.13.-]
K03684	7.50575E-06	6.70953E-06	1.4884E-05	7.30276E-05	ribonuclease D [EC:3.1.13.5]
K03685	0.000224717	0.000226663	0.000240116	0.00026013	ribonuclease III [EC:3.1.26.3]
K03686	0.000232699	0.000241059	0.00026771	0.000328513	molecular chaperone DnaJ
K03687	0.000224409	0.00022652	0.00022899	0.000226984	molecular chaperone GrpE
K03688	0.00022876	0.000227346	0.000232665	0.000381403	ubiquinone biosynthesis protein
K03690	0.000217642	0.00021692	0.000206905	0.000217428	ubiquinone biosynthesis protein UbiJ
K03692	2.16535E-07	9.79857E-08	2.04148E-06	1.34111E-05	glucosylglycerol-phosphate synthase [EC:2.4.1.213]
K03693	1.30969E-06	2.60917E-06	1.10766E-05	3.12685E-05	penicillin-binding protein 1B
K03694	0.000450268	0.000458637	0.000483165	0.000504723	ATP-dependent Clp protease ATP-binding subunit ClpA
K03695	0.000662857	0.000668978	0.000664753	0.000628554	ATP-dependent Clp protease ATP-binding subunit ClpB
K03696	0.000665413	0.000671266	0.000667551	0.000664629	ATP-dependent Clp protease ATP-binding subunit ClpC
K03697	1.10482E-05	1.19972E-05	3.11889E-05	0.000158584	ATP-dependent Clp protease ATP-binding subunit ClpE
K03698	1.88693E-06	1.25858E-06	7.18098E-06	4.61862E-06	3-5 exoribonuclease [EC:3.1.-.-]
K03699	0.00044991	0.00045377	0.00047925	0.000519738	putative hemolysin
K03700	1.51483E-06	5.10882E-07	7.54091E-06	3.80114E-06	recombination protein U
K03701	0.000444022	0.000444833	0.00045627	0.000449893	excinuclease ABC subunit A
K03702	0.000225293	0.000228314	0.000229581	0.000228424	excinuclease ABC subunit B
K03703	0.000224494	0.00022583	0.000234927	0.000226692	excinuclease ABC subunit C
K03704	0.00109538	0.001082708	0.000996378	0.000862255	cold shock protein (beta-ribbon, CspA family)
K03705	0.000221424	0.000221234	0.000222754	0.000211013	heat-inducible transcriptional repressor
K03706	1.27853E-06	4.51734E-07	3.62468E-06	3.6402E-06	transcriptional pleiotropic repressor
K03707	3.81101E-06	2.26592E-06	1.41094E-05	6.32463E-06	thiaminase (transcriptional activator TenA) [EC:3.5.99.2]
K03708	1.64357E-06	4.88656E-07	4.4429E-06	3.64344E-06	transcriptional regulator of stress and heat shock response
K03709	3.14242E-06	6.05829E-06	1.60794E-05	1.16468E-05	DtxR family transcriptional regulator, Mn-dependent transcriptional regulator
K03710	0.000874144	0.000876262	0.000874407	0.000790982	GntR family transcriptional regulator
K03711	0.000656423	0.000661592	0.000657299	0.00058805	Fur family transcriptional regulator, ferric uptake regulator
K03712	1.81327E-07	1.01808E-07	2.15376E-07	9.05599E-07	MarR family transcriptional regulator, multiple antibiotic resistance protein MarR
K03713	1.2237E-06	3.06025E-07	3.75404E-06	3.65575E-06	MerR family transcriptional regulator, glutamine synthetase repressor

K03715	2.28045E-08	2.76961E-08	1.31461E-07	8.56528E-08	1,2-diacylglycerol 3-beta-galactosyltransferase [EC:2.4.1.46]
K03716	1.39568E-06	4.77747E-07	6.44896E-07	3.92199E-08	spore photoproduct lyase [EC:4.1.99.14]
K03717	0.000223277	0.000222058	0.000241082	0.000344661	LysR family transcriptional regulator, transcriptional activator of nhaA
K03718	8.90486E-07	2.91871E-06	7.25317E-06	1.83109E-05	Lrp/AsnC family transcriptional regulator, regulator for asnA, asnC and gidA
K03719	0.00259879	0.002589898	0.002424732	0.00197828	Lrp/AsnC family transcriptional regulator, leucine-responsive regulatory protein
K03720	1.23686E-06	3.23013E-07	8.36266E-07	1.10161E-05	TrpR family transcriptional regulator, trp operon repressor
K03721	0.001080777	0.001076862	0.001008851	0.000794331	transcriptional regulator of aroF, aroG, tyrA and aromatic amino acid transport
K03722	0.000223459	0.000226648	0.0002486	0.00033898	ATP-dependent DNA helicase DinG [EC:3.6.4.12]
K03723	0.000226346	0.000224846	0.000229839	0.000227814	transcription-repair coupling factor (superfamily II helicase) [EC:3.6.4.-]
K03724	6.98404E-06	1.00851E-05	2.96307E-05	0.000132358	ATP-dependent helicase Lhr and Lhr-like helicase [EC:3.6.4.-]
K03726	5.12601E-08	1.06288E-07	1.8085E-06	4.39204E-06	helicase [EC:3.6.4.-]
K03727	1.76696E-06	5.07387E-06	1.04801E-05	4.29383E-06	ATP-dependent RNA helicase HelY [EC:3.6.4.-]
K03731	0	0	7.03844E-08	4.71496E-07	trehalose 6-phosphate phosphorylase [EC:2.4.1.216]
K03732	0.000655614	0.000655165	0.000635753	0.000584089	ATP-dependent RNA helicase RhlB [EC:3.6.4.13]
K03733	0.000236413	0.000243149	0.000254152	0.000263676	integrase/recombinase XerC
K03734	0.000435141	0.000430537	0.000405829	0.00037317	FAD:protein FMN transferase [EC:2.7.1.180]
K03735	0.000217735	0.000213171	0.000191608	0.000123576	ethanolamine ammonia-lyase large subunit [EC:4.3.1.7]
K03736	0.000216414	0.000213142	0.000191289	0.000121682	ethanolamine ammonia-lyase small subunit [EC:4.3.1.7]
K03737	3.97213E-06	3.34974E-06	1.54347E-05	7.35662E-06	pyruvate-ferredoxin/flavodoxin oxidoreductase [EC:1.2.7.1 1.2.7.-]
K03738	2.66209E-07	2.44238E-07	5.69711E-07	3.65311E-07	aldehyde:ferredoxin oxidoreductase [EC:1.2.7.5]
K03739	2.22021E-06	6.70551E-07	4.20643E-06	3.74929E-06	membrane protein involved in D-alanine export
K03740	1.29019E-06	1.50496E-07	3.53398E-06	3.71172E-06	D-alanine transfer protein
K03741	0.000224535	0.00022773	0.000223378	0.000247696	arsenate reductase [EC:1.20.4.1]
K03742	0.000219897	0.000219804	0.000205581	0.00014226	nicotinamide-nucleotide amidase [EC:3.5.1.42]
K03743	0.000221433	0.0002232	0.00022463	0.000226385	nicotinamide-nucleotide amidase [EC:3.5.1.42]
K03744	0.000437537	0.000439991	0.000440804	0.000408149	LemA protein
K03745	5.08329E-06	5.13539E-06	2.02452E-05	0.000104175	SlyX protein
K03746	0.001087807	0.001099579	0.001117988	0.001169098	DNA-binding protein H-NS
K03747	1.28354E-06	1.62429E-06	2.86473E-06	6.88511E-05	Smg protein
K03748	7.81361E-07	1.2216E-06	8.07927E-06	1.49728E-05	SanA protein
K03749	0.000218372	0.000220804	0.000226865	0.000263875	DedD protein
K03750	0.000441181	0.000434763	0.000436727	0.000376894	molybdopterin molybdotransferase [EC:2.10.1.1]
K03752	0.000221354	0.000217652	0.000218592	0.000208041	molybdenum cofactor guanylyltransferase [EC:2.7.7.77]
K03753	0.000430636	0.000427247	0.000393592	0.000275345	molybdopterin-guanine dinucleotide biosynthesis adapter protein
K03755	9.22055E-09	3.36919E-08	6.6401E-08	4.02072E-07	AraC family transcriptional regulator, transcriptional activator of adiA
K03756	4.31888E-07	5.29744E-07	8.86749E-07	1.22534E-05	putrescine:ornithine antiporter
K03757	9.05341E-07	8.56703E-07	1.50264E-06	4.80011E-05	cadaverine:lysine antiporter
K03758	2.91668E-06	2.49827E-06	1.47263E-05	7.32725E-05	arginine:ornithine antiporter / lysine permease
K03759	9.87488E-07	5.95525E-07	1.48862E-06	4.83085E-05	arginine:agmatine antiporter
K03760	5.04204E-06	7.14184E-06	3.1043E-05	0.000157161	lipid A ethanolaminephosphotransferase [EC:2.7.8.43]
K03761	0.001519193	0.001501778	0.001381517	0.001231023	MFS transporter, MHS family, alpha-ketoglutarate permease
K03762	0.00153051	0.001516434	0.001425072	0.001324961	MFS transporter, MHS family, proline/betaine transporter
K03763	3.0234E-06	1.94317E-06	9.84492E-06	5.82153E-05	DNA polymerase III subunit alpha, Gram-positive type [EC:2.7.7.7]
K03764	1.11715E-07	3.32951E-07	3.84549E-07	1.71502E-05	MetJ family transcriptional regulator, methionine regulon repressor
K03765	9.80503E-08	3.13029E-07	2.43984E-07	2.70486E-06	transcriptional activator of cad operon
K03767	0.000439162	0.000440001	0.000434822	0.000391301	peptidyl-prolyl cis-trans isomerase A (cyclophilin A) [EC:5.2.1.8]
K03768	0.000443456	0.00044216	0.000449462	0.00040133	peptidyl-prolyl cis-trans isomerase B (cyclophilin B) [EC:5.2.1.8]
K03769	0.000225095	0.000228116	0.000265739	0.000361125	peptidyl-prolyl cis-trans isomerase C [EC:5.2.1.8]
K03770	0.000649675	0.000645829	0.000598506	0.000475883	peptidyl-prolyl cis-trans isomerase D [EC:5.2.1.8]
K03771	0.000435317	0.000435247	0.000414946	0.000390091	peptidyl-prolyl cis-trans isomerase SurA [EC:5.2.1.8]
K03772	0.000440808	0.000441778	0.000435943	0.000434443	FKBP-type peptidyl-prolyl cis-trans isomerase FkpA [EC:5.2.1.8]
K03773	7.20276E-06	1.12522E-05	1.78702E-05	0.000103983	FKBP-type peptidyl-prolyl cis-trans isomerase FklB [EC:5.2.1.8]
K03774	0.000216538	0.000215648	0.000204818	0.000166153	FKBP-type peptidyl-prolyl cis-trans isomerase SlpA [EC:5.2.1.8]
K03775	0.000219143	0.000221908	0.000227749	0.000261127	FKBP-type peptidyl-prolyl cis-trans isomerase SlyD [EC:5.2.1.8]
K03776	0.003464471	0.003473959	0.003445793	0.003164369	aerotaxis receptor
K03777	0.000218521	0.000217087	0.000208178	0.000257331	D-lactate dehydrogenase (quinone) [EC:1.1.5.12]
K03778	0.000229676	0.00022594	0.00023651	0.000235507	D-lactate dehydrogenase [EC:1.1.1.28]
K03779	1.97681E-08	1.6149E-07	1.07409E-07	4.27543E-07	L(+)-tartrate dehydratase alpha subunit [EC:4.2.1.32]
K03780	2.01344E-08	1.62578E-07	1.42566E-07	4.40087E-07	L(+)-tartrate dehydratase beta subunit [EC:4.2.1.32]
K03781	0.00022816	0.000232307	0.00024608	0.000342554	catalase [EC:1.11.1.6]
K03782	0.000216743	0.000213121	0.000193086	0.000157393	catalase-peroxidase [EC:1.11.1.21]
K03783	3.74146E-06	5.99452E-06	1.10129E-05	1.00354E-05	purine-nucleoside phosphorylase [EC:2.4.2.1]
K03784	3.01518E-06	1.57639E-06	9.30959E-06	2.93011E-05	purine-nucleoside phosphorylase [EC:2.4.2.1]
K03785	3.29154E-06	1.8048E-06	8.94343E-06	7.81471E-05	3-dehydroquinate dehydratase I [EC:4.2.1.10]
K03786	0.000437861	0.000437845	0.000422546	0.000371238	3-dehydroquinate dehydratase II [EC:4.2.1.10]
K03787	0.000218467	0.000218819	0.000211508	0.000220959	5-nucleotidase [EC:3.1.3.5]
K03788	2.83668E-08	1.95539E-08	2.19676E-07	2.72343E-06	acid phosphatase (class B) [EC:3.1.3.2]
K03789	0.00044602	0.000444539	0.00046029	0.000514606	[ribosomal protein S18]-alanine N-acetyltransferase [EC:2.3.1.266]
K03790	0.000229618	0.000238767	0.00025858	0.000405331	[ribosomal protein S5]-alanine N-acetyltransferase [EC:2.3.1.267]
K03791	0.000216879	0.000215094	0.000196845	0.000221309	putative chitinase
K03793	8.27321E-06	9.07615E-06	4.40436E-05	0.000147046	pteridine reductase [EC:1.5.1.33]
K03794	2.20873E-08	2.06813E-07	2.30544E-06	1.05773E-07	sirohydrochlorin ferrochelatase [EC:4.99.1.4]
K03795	0.000215135	0.000214875	0.000205292	0.00014909	sirohydrochlorin cobaltochelatase [EC:4.99.1.3]
K03796	7.12666E-08	3.35802E-08	7.92536E-07	1.56949E-05	Bax protein
K03797	0.000443875	0.000442974	0.000428987	0.000452199	carboxyl-terminal processing protease [EC:3.4.21.102]
K03798	0.000230627	0.000229469	0.000245641	0.000246829	cell division protease FtsH [EC:3.4.24.-]
K03799	0.000219293	0.000220293	0.000214387	0.000230743	heat shock protein HtpX [EC:3.4.24.-]
K03800	4.27985E-06	6.10682E-06	1.15992E-05	1.5138E-05	lipoate---protein ligase [EC:6.3.1.20]
K03801	0.000221167	0.000223045	0.000218843	0.000221706	lipoyl(octanoyl) transferase [EC:2.3.1.181]
K03802	4.00231E-06	8.18049E-06	4.0623E-05	7.15219E-05	cyanophycin synthetase [EC:6.3.2.29 6.3.2.30]
K03803	6.11054E-07	1.63872E-06	2.72273E-06	1.78637E-05	sigma-E factor negative regulatory protein RseC
K03804	9.96566E-08	3.20313E-07	6.00981E-07	1.23004E-05	chromosome partition protein MukE
K03805	3.53704E-06	7.2347E-06	2.96945E-05	0.000121845	thiol:disulfide interchange protein DsbG
K03806	0.000218156	0.000216838	0.000206752	0.000169249	N-acetyl-anhydromuramoyl-L-alanine amidase [EC:3.5.1.28]
K03807	1.31143E-06	1.33849E-06	2.98687E-06	5.72466E-05	AmpE protein
K03808	0.000428414	0.000425384	0.000375848	0.000257619	paraquat-inducible protein A
K03809	0.000647197	0.000641783	0.000582169	0.000445231	NAD(P)H dehydrogenase (quinone) [EC:1.6.5.2]

K03810	0.000214258	0.000212278	0.00018719	0.000126547	virulence factor
K03811	5.61668E-06	6.98945E-06	1.48369E-05	0.000115143	nicotinamide mononucleotide transporter
K03812	1.59752E-07	4.68554E-07	2.18662E-06	1.31472E-05	ribosome modulation factor
K03813	6.33065E-08	5.51488E-08	3.1734E-07	1.69436E-07	molybdenum transport protein [EC:2.4.2.-]
K03814	0.000223031	0.000223611	0.000211082	0.000210623	monofunctional glycosyltransferase [EC:2.4.1.129]
K03815	5.39658E-07	5.78095E-07	3.98355E-07	4.81188E-06	xanthosine phosphorylase [EC:2.4.2.-]
K03816	2.53427E-06	1.73995E-06	8.56789E-06	3.98885E-06	xanthine phosphoribosyltransferase [EC:2.4.2.22]
K03817	1.83026E-06	6.72237E-07	5.69468E-06	4.56742E-05	ribosomal-protein-serine acetyltransferase [EC:2.3.1.-]
K03818	0.000215129	0.000215548	0.00018737	0.000115707	putative colanic acid biosynthesis acetyltransferase WcaF [EC:2.3.1.-]
K03819	2.5532E-08	3.0469E-07	5.08645E-07	3.08105E-06	putative colanic acid biosynthesis acetyltransferase WcaB [EC:2.3.1.-]
K03820	0.000219265	0.000218034	0.000217693	0.000184939	apolipoprotein N-acyltransferase [EC:2.3.1.-]
K03821	0.000221952	0.000216512	0.000210488	0.00020808	polyhydroxyalkanoate synthase [EC:2.3.1.-]
K03822	0	1.34585E-07	3.18094E-08	1.7338E-06	putative long chain acyl-CoA synthase [EC:6.2.1.-]
K03823	0.000439403	0.000433456	0.000417396	0.000386447	phosphinothricin acetyltransferase [EC:2.3.1.183]
K03824	3.6459E-06	2.5507E-06	7.89066E-06	8.24863E-05	putative acetyltransferase [EC:2.3.1.-]
K03825	9.18585E-07	8.22388E-07	3.00362E-06	1.39684E-05	putative acetyltransferase [EC:2.3.1.-]
K03826	7.98915E-07	1.4467E-06	5.57893E-06	1.48454E-05	putative acetyltransferase [EC:2.3.1.-]
K03827	3.34448E-06	5.11128E-06	1.81266E-05	0.000117814	putative acetyltransferase [EC:2.3.1.-]
K03828	3.72757E-07	1.48993E-06	3.83368E-06	1.52625E-05	putative acetyltransferase [EC:2.3.1.-]
K03829	2.28635E-06	3.53895E-06	1.81434E-05	4.95935E-05	putative acetyltransferase [EC:2.3.1.-]
K03830	0.000214251	0.000212849	0.000187491	0.000120284	putative acetyltransferase [EC:2.3.1.-]
K03831	0.000218228	0.000216953	0.000211386	0.000198158	molybdopterin adenylyltransferase [EC:2.7.7.75]
K03832	0.000450252	0.000453401	0.000464774	0.000895106	periplasmic protein TonB
K03833	5.33217E-06	6.24437E-06	1.77126E-05	6.61704E-05	selenocysteine-specific elongation factor
K03834	9.82377E-08	3.41802E-07	4.66217E-07	1.93244E-05	tyrosine-specific transport protein
K03835	1.96568E-07	6.02365E-07	6.28619E-07	1.42422E-05	tryptophan-specific transport protein
K03836	2.84612E-08	1.86389E-08	5.45684E-08	1.7294E-06	low affinity tryptophan permease
K03837	2.768E-07	1.51462E-06	3.49049E-06	3.35736E-05	serine transporter
K03838	5.23477E-08	1.9566E-08	5.98293E-07	1.56209E-06	threonine transporter
K03839	8.08067E-07	1.92175E-06	4.93845E-06	3.19401E-05	flavodoxin I
K03840	9.82118E-08	3.14581E-07	4.20462E-07	1.60372E-05	flavodoxin II
K03841	0.000219268	0.000217816	0.000208031	0.0002198	fructose-1,6-bisphosphatase I [EC:3.1.3.11]
K03851	9.06584E-08	1.64581E-07	3.44451E-06	5.349E-07	taurine-pyruvate aminotransferase [EC:2.6.1.77]
K03852	1.59787E-06	3.13082E-06	1.40203E-05	3.47397E-05	sulfoacetaldehyde acetyltransferase [EC:2.3.3.15]
K03855	4.84333E-07	5.37425E-07	4.99577E-06	1.74854E-06	ferredoxin like protein
K03856	3.59518E-06	4.58029E-06	2.15303E-05	3.57948E-05	3-deoxy-7-phosphoheptulonate synthase [EC:2.5.1.54]
K03862	0.000215646	0.00021599	0.000206195	0.000162142	vanillate monooxygenase [EC:1.14.13.82]
K03863	0.000218727	0.000223307	0.000233472	0.000231937	vanillate monooxygenase ferredoxin subunit
K03867	1.03124E-06	4.78411E-07	2.44421E-07	1.85934E-08	UDP-glucose:tetrahydrobiopterin glucosyltransferase [EC:2.4.1.-]
K03885	0.000227915	0.000230427	0.000239594	0.000285757	NADH dehydrogenase [EC:1.6.99.3]
K03886	1.82528E-07	2.06813E-07	9.45222E-07	7.86262E-08	menaquinol-cytochrome c reductase iron-sulfur subunit [EC:1.10.2.-]
K03887	1.91697E-07	2.06813E-07	7.94772E-07	1.90174E-09	menaquinol-cytochrome c reductase cytochrome b subunit
K03888	1.55488E-07	2.06813E-07	7.17671E-07	1.90174E-09	menaquinol-cytochrome c reductase cytochrome b/c subunit
K03889	1.54035E-06	4.10651E-06	7.87336E-06	4.03576E-06	ubiquinol-cytochrome c reductase cytochrome c subunit
K03890	1.50897E-06	4.07287E-06	7.85843E-06	4.00786E-06	ubiquinol-cytochrome c reductase iron-sulfur subunit
K03891	1.55676E-06	4.08925E-06	7.93663E-06	4.15781E-06	ubiquinol-cytochrome c reductase cytochrome b subunit
K03892	0.000233488	0.000236095	0.000269896	0.000406556	ArsR family transcriptional regulator, arsenate/arsenite/antimonite-responsive transcriptional repressor
K03893	7.39738E-06	5.31199E-06	2.40725E-05	7.24026E-05	arsenical pump membrane protein
K03894	0.000213982	0.000211789	0.00018632	0.000113382	N2-citryl-N6-acetyl-N6-hydroxylysine synthase [EC:6.3.2.38]
K03895	0.000214729	0.000211946	0.000186915	0.000113458	aerobactin synthase [EC:6.3.2.39]
K03896	2.3572E-06	2.99997E-06	1.65556E-05	3.5027E-05	acetyl CoA:N6-hydroxylysine acetyl transferase [EC:2.3.1.102]
K03897	0.000216416	0.000212516	0.000191183	0.000118455	lysine N6-hydroxylase [EC:1.14.13.59]
K03918	7.24513E-09	3.05104E-07	2.45693E-07	1.77645E-06	L-lysine 6-transaminase [EC:2.6.1.36]
K03919	0.000216571	0.000216409	0.00020322	0.000150852	DNA oxidative demethylase [EC:1.14.11.33]
K03921	0	8.96466E-07	4.8948E-07	1.92508E-06	acyl-[acyl-carrier-protein] desaturase [EC:1.14.19.2 1.14.19.11 1.14.19.26]
K03922	0	1.34585E-07	1.54857E-08	2.82158E-08	acyl-[acyl-carrier-protein] desaturase [EC:1.14.19.2]
K03923	1.56415E-07	1.51237E-06	2.09356E-06	4.56935E-06	modulator of drug activity B
K03924	0.000656605	0.000653692	0.000619184	0.000653098	MoxR-like ATPase [EC:3.6.3.-]
K03925	0.000220679	0.000223159	0.000224116	0.000214221	MraZ protein
K03926	0.000217582	0.00021645	0.000203243	0.000211312	periplasmic divalent cation tolerance protein
K03928	6.27108E-06	9.71176E-06	4.07427E-05	0.000106427	carboxylesterase [EC:3.1.1.1]
K03929	6.85719E-06	5.89406E-06	3.44475E-05	7.99805E-05	para-nitrobenzyl esterase [EC:3.1.1.-]
K03930	1.70096E-06	8.47972E-08	9.40383E-06	3.18743E-06	putative tributyrin esterase [EC:3.1.1.-]
K03931	5.61857E-07	1.40281E-06	2.83683E-07	4.62864E-06	putative isomerase
K03932	5.5781E-07	1.81987E-06	1.01229E-05	2.27437E-05	polyhydroxybutyrate depolymerase
K03933	1.32215E-06	1.04299E-06	3.95422E-06	8.38507E-05	chitin-binding protein
K03969	3.21541E-06	8.40305E-06	2.7915E-05	6.74962E-05	phage shock protein A
K03970	2.30396E-07	3.78097E-07	1.78153E-06	1.74855E-05	phage shock protein B
K03971	9.8121E-08	3.00777E-07	7.42683E-08	7.53619E-07	phage shock protein D
K03972	2.97385E-06	2.96205E-06	8.94665E-06	2.39216E-05	phage shock protein E
K03973	7.36315E-07	2.48742E-06	5.05102E-06	2.39586E-05	phage shock protein C
K03974	0.000868535	0.000859891	0.000799748	0.000721498	psp operon transcriptional activator
K03975	0.000217996	0.000217996	0.000216154	0.000166397	membrane-associated protein
K03976	7.94484E-06	7.29415E-06	2.32668E-05	7.4532E-05	Cys-tRNA(Pro)/Cys-tRNA(Cys) deacylase [EC:3.1.1.-]
K03977	0.000228294	0.000229734	0.000243601	0.00022584	GTPase
K03978	0.000221914	0.000219575	0.000218748	0.000221647	GTP-binding protein
K03979	0.000223692	0.000223845	0.000230097	0.000225479	GTPase [EC:3.6.5.-]
K03980	0.000222321	0.000222396	0.0002253	0.0002238	putative peptidoglycan lipid II flippase
K03981	0.000218347	0.000218874	0.000214386	0.000234538	thiol:disulfide interchange protein DsbC [EC:5.3.4.1]
K04013	4.3616E-09	4.54878E-08	4.28723E-07	5.59478E-06	cytochrome c-type protein NrfB
K04014	1.21288E-06	3.01118E-06	1.5413E-05	4.31255E-05	protein NrfC
K04015	4.3616E-09	3.19097E-08	1.52185E-06	8.25861E-06	protein NrfD
K04016	3.73776E-06	1.95201E-06	5.36693E-06	7.1031E-05	cytochrome c-type biogenesis protein NrfE
K04017	4.34863E-09	1.82993E-08	2.70871E-07	1.12608E-06	formate-dependent nitrite reductase complex subunit NrfF
K04018	8.6843E-09	5.01445E-08	5.96693E-07	5.08961E-06	formate-dependent nitrite reductase complex subunit NrfG
K04019	1.25672E-07	4.16019E-07	6.12119E-07	1.85158E-06	ethanolamine utilization protein EutA
K04020	1.4183E-06	4.56335E-07	3.60291E-06	4.74441E-07	phosphotransacetylase

K04021	7.93278E-07	5.00614E-07	5.29206E-06	3.62768E-07	aldehyde dehydrogenase
K04022	1.16572E-06	1.56898E-06	1.05122E-06	5.22793E-06	alcohol dehydrogenase
K04023	4.02494E-07	7.40776E-07	1.22023E-06	1.84112E-06	ethanolamine transporter
K04024	3.818E-07	2.232E-07	7.1521E-07	3.55401E-07	ethanolamine utilization protein EutJ
K04025	2.83454E-08	1.83509E-08	4.18799E-08	2.97531E-07	ethanolamine utilization protein EutK
K04026	3.88172E-08	1.51317E-07	6.11442E-07	1.85502E-06	ethanolamine utilization protein EutL
K04027	8.7982E-07	6.22721E-07	2.97236E-06	9.05291E-06	ethanolamine utilization protein EutM
K04028	4.14569E-07	3.87437E-07	1.1064E-06	2.70692E-06	ethanolamine utilization protein EutN
K04029	4.54653E-07	4.89849E-07	1.13511E-06	2.03753E-06	ethanolamine utilization protein EutP
K04030	9.27257E-08	4.67935E-08	1.0393E-06	2.08279E-06	ethanolamine utilization protein EutQ
K04031	4.05911E-07	2.85003E-07	1.23333E-06	1.94639E-06	ethanolamine utilization protein EutS
K04032	3.87861E-08	1.83074E-08	3.95754E-07	3.1408E-07	ethanolamine utilization cobalamin adenosyltransferase [EC:2.5.1.17]
K04033	0.000214941	0.000213509	0.000192996	0.000172229	AraC family transcriptional regulator, ethanolamine operon transcriptional activator
K04034	3.07686E-07	7.71826E-07	1.24766E-06	1.51335E-07	anaerobic magnesium-protoporphyrin IX monomethyl ester cyclase [EC:1.21.98.3]
K04035	9.7076E-07	1.11305E-08	8.07648E-08	1.22569E-07	magnesium-protoporphyrin IX monomethyl ester (oxidative) cyclase [EC:1.14.13.81]
K04036	0	9.59746E-09	4.12951E-08	1.02483E-07	divinyl protochlorophyllide a 8-vinyl-reductase [EC:1.-.-.-]
K04037	1.11148E-06	1.06979E-07	8.62025E-08	1.46789E-07	light-independent protochlorophyllide reductase subunit L [EC:1.3.7.7]
K04038	9.7076E-07	1.11305E-08	8.07648E-08	1.22569E-07	light-independent protochlorophyllide reductase subunit N [EC:1.3.7.7]
K04039	9.7076E-07	1.11305E-08	8.07648E-08	1.22569E-07	light-independent protochlorophyllide reductase subunit B [EC:1.3.7.7]
K04040	9.7076E-07	1.11305E-08	9.12305E-08	1.30606E-07	chlorophyll/bacteriochlorophyll a synthase [EC:2.5.1.62 2.5.1.133]
K04041	1.73429E-06	1.13983E-06	5.90873E-06	2.3034E-06	fructose-1,6-bisphosphatase III [EC:3.1.3.11]
K04042	0.000227935	0.000227023	0.000246887	0.000233635	bifunctional UDP-N-acetylglucosamine pyrophosphorylase / Glucosamine-1-phosphate N-acetyltransferase [EC:2.7.7.[illegible]
K04043	0.000656918	0.000657788	0.000638704	0.000570127	molecular chaperone DnaK
K04044	0.000433463	0.000431704	0.000405445	0.000311308	molecular chaperone HscA
K04045	0.000216719	0.000214739	0.000201113	0.000170688	molecular chaperone HscC
K04046	0.000217513	0.000216045	0.000208013	0.000218527	hypothetical chaperone protein
K04047	0.000224348	0.000222078	0.000220022	0.000242073	starvation-inducible DNA-binding protein
K04049	0	0	5.76224E-08	1.77873E-07	type III secretion protein B
K04050	0	0	0	1.41313E-09	type III secretion protein E
K04051	0	0	5.76224E-08	1.77873E-07	type III secretion protein G
K04052	0	0	5.76224E-08	1.77873E-07	type III secretion protein H
K04053	0	1.83961E-08	1.29889E-07	1.77873E-07	type III secretion protein I
K04054	1.74228E-08	5.64392E-08	3.48405E-07	2.13083E-07	type III secretion protein K
K04055	0	0	0	2.82626E-09	type III secretion protein M
K04056	0	1.83961E-08	1.29889E-07	1.77873E-07	type III secretion protein O
K04057	0	0	5.76224E-08	1.77873E-07	type III secretion protein P
K04058	2.31084E-09	2.84977E-08	1.45244E-07	2.59933E-07	type III secretion protein W
K04059	0	1.83961E-08	1.29889E-07	1.77873E-07	type III secretion protein X
K04060	0	5.10398E-11	6.28553E-08	1.87871E-07	type III secretion protein Y
K04061	1.30339E-06	1.36488E-06	3.20554E-06	5.59304E-06	flagellar biosynthesis protein
K04062	0.000428942	0.000426729	0.000383872	0.00025656	osmotically inducible lipoprotein OsmB
K04063	0.000440475	0.000440364	0.000428502	0.000421224	osmotically inducible protein OsmC
K04064	2.68413E-07	5.04612E-07	9.97274E-07	9.07475E-07	osmotically inducible lipoprotein OsmE
K04065	2.26713E-06	3.60473E-06	4.53875E-06	6.3997E-05	hyperosmotically inducible periplasmic protein
K04066	0.00022378	0.000224416	0.000230986	0.000234828	primosomal protein N (replication factor Y) (superfamily II helicase) [EC:3.6.4.-]
K04067	9.82118E-08	3.14581E-07	1.61387E-07	1.40919E-05	primosomal replication protein N
K04068	3.7004E-06	2.59998E-06	1.97723E-05	2.67356E-05	anaerobic ribonucleoside-triphosphate reductase activating protein [EC:1.97.1.4]
K04069	0.000218137	0.000216276	0.000216673	0.000148258	pyruvate formate lyase activating enzyme [EC:1.97.1.4]
K04070	8.0684E-07	4.71409E-07	6.24443E-07	2.90994E-08	putative pyruvate formate lyase activating enzyme [EC:1.97.1.4]
K04072	6.30387E-06	6.86997E-06	2.76671E-05	7.55479E-05	acetaldehyde dehydrogenase / alcohol dehydrogenase [EC:1.2.1.10 1.1.1.1]
K04073	0.000213984	0.00021271	0.000188488	0.000119446	acetaldehyde dehydrogenase [EC:1.2.1.10]
K04074	3.30943E-06	3.89749E-06	8.76287E-06	9.26942E-06	cell division initiation protein
K04075	0.000224679	0.000224776	0.000231909	0.000225608	tRNA(Ile)-lysidine synthase [EC:6.3.4.19]
K04076	1.72343E-07	6.94739E-07	2.24145E-07	2.53939E-08	ATP-dependent Lon protease [EC:3.4.21.53]
K04077	0.000226439	0.000228146	0.000239194	0.000233172	chaperonin GroEL
K04078	0.000224926	0.000224	0.000226382	0.000228603	chaperonin GroES
K04079	0.000220734	0.000220253	0.000215222	0.000274133	molecular chaperone HtpG
K04080	5.90294E-06	1.00132E-06	3.09272E-06	2.19197E-05	molecular chaperone IbpA
K04081	9.81956E-08	3.00963E-07	1.14473E-07	1.14448E-06	molecular chaperone IbpB
K04082	0.000216565	0.000215641	0.00020558	0.0001667	molecular chaperone HscB
K04083	0.000221642	0.000218106	0.000216077	0.000221344	molecular chaperone Hsp33
K04084	0.000219849	0.000219679	0.000211465	0.000224846	thiol:disulfide interchange protein DsbD [EC:1.8.1.8]
K04085	0.000220048	0.000216962	0.000208182	0.00016883	tRNA 2-thiouridine synthesizing protein A [EC:2.8.1.-]
K04086	2.79944E-06	5.07554E-06	2.05133E-05	5.26277E-05	ATP-dependent Clp protease ATP-binding subunit ClpL
K04087	0.000218013	0.000217394	0.000207609	0.000218308	membrane protease subunit HflC [EC:3.4.-.-]
K04088	0.000431664	0.000429183	0.000394175	0.000334534	membrane protease subunit HflK [EC:3.4.-.-]
K04090	0.000216628	0.000216694	0.000209096	0.000208599	indolepyruvate ferredoxin oxidoreductase [EC:1.2.7.8]
K04091	0.000228507	0.000226273	0.000231161	0.000273361	alkanesulfonate monooxygenase [EC:1.14.14.5]
K04092	2.57177E-06	3.31882E-06	6.71578E-06	4.08162E-06	chorismate mutase [EC:5.4.99.5]
K04093	0.000214021	0.000212923	0.000189424	0.000115245	chorismate mutase [EC:5.4.99.5]
K04094	4.73284E-06	1.57268E-06	9.86828E-06	5.6097E-05	methylenetetrahydrofolate--tRNA-(uracil-5-)-methyltransferase [EC:2.1.1.74]
K04095	4.37179E-06	4.2473E-06	2.02938E-05	4.71495E-05	cell filamentation protein
K04096	0.00022443	0.000224085	0.000229725	0.000236773	DNA processing protein
K04097	0	0	0	2.31643E-06	prostaglandin-H2 D-isomerase / glutathione transferase [EC:5.3.99.2 2.5.1.18]
K04098	5.66679E-07	2.14047E-07	2.71597E-06	6.20644E-07	hydroxyquinol 1,2-dioxygenase [EC:1.13.11.37]
K04099	3.92374E-06	9.51038E-06	4.46598E-05	0.000115494	gallate dioxygenase [EC:1.13.11.57]
K04100	3.9098E-06	9.48837E-06	4.45003E-05	0.000114123	protocatechuate 4,5-dioxygenase, alpha chain [EC:1.13.11.8]
K04101	3.9324E-06	9.59324E-06	4.47031E-05	0.000116231	protocatechuate 4,5-dioxygenase, beta chain [EC:1.13.11.8]
K04102	5.12795E-07	9.28867E-07	2.91729E-06	6.9696E-06	4,5-dihydroxyphthalate decarboxylase [EC:4.1.1.55]
K04103	1.14684E-06	7.75314E-07	3.63472E-06	1.53491E-06	indolepyruvate decarboxylase [EC:4.1.1.74]
K04105	2.88757E-07	6.74822E-07	3.10756E-06	7.10594E-06	4-hydroxybenzoate-CoA ligase [EC:6.2.1.27 6.2.1.25]
K04107	0	3.00077E-08	5.70617E-08	1.20557E-08	4-hydroxybenzoyl-CoA reductase subunit gamma [EC:1.3.7.9]
K04108	1.26739E-08	2.62409E-08	2.40723E-07	1.95407E-07	4-hydroxybenzoyl-CoA reductase subunit alpha [EC:1.3.7.9]
K04109	0	9.59746E-09	0	4.01858E-09	4-hydroxybenzoyl-CoA reductase subunit beta [EC:1.3.7.9]
K04110	1.29971E-06	3.10832E-06	1.48508E-05	3.50467E-05	benzoate-CoA ligase [EC:6.2.1.25]
K04112	0	9.59746E-09	0	4.01858E-09	benzoyl-CoA reductase subunit C [EC:1.3.7.8]
K04113	0	9.59746E-09	0	4.01858E-09	benzoyl-CoA reductase subunit B [EC:1.3.7.8]

K04114	8.87965E-07	4.41002E-07	1.14898E-06	7.28653E-07	benzoyl-CoA reductase subunit A [EC:1.3.7.8]
K04115	0	1.36702E-08	3.09713E-08	5.14914E-09	benzoyl-CoA reductase subunit D [EC:1.3.7.8]
K04116	1.69241E-07	2.73762E-07	1.57668E-06	5.25087E-06	cyclohexanecarboxylate-CoA ligase [EC:6.2.1.-]
K04117	5.03897E-06	9.23629E-06	4.27968E-05	0.000106539	cyclohexanecarboxyl-CoA dehydrogenase [EC:1.3.99.-]
K04118	0.000217947	0.000219508	0.000225558	0.000202837	pimeloyl-CoA dehydrogenase [EC:1.3.1.62]
K04127	0.000213897	0.000211777	0.000186603	0.00011344	isopenicillin-N epimerase [EC:5.1.1.17]
K04128	2.56886E-09	4.14328E-10	1.97874E-08	1.41313E-09	hydroxymethyl cephem carbamoyltransferase [EC:2.1.3.7]
K04333	4.61487E-08	1.98408E-08	4.47148E-06	1.43293E-05	LuxR family transcriptional regulator, csgAB operon transcriptional regulatory protein
K04334	4.3065E-09	1.93443E-08	4.9669E-08	2.55197E-07	major curlin subunit
K04335	1.11988E-06	1.61106E-07	7.28125E-08	3.45188E-06	minor curlin subunit
K04336	4.33243E-09	1.94088E-08	3.81767E-08	2.55965E-07	curli production protein
K04337	1.72177E-07	9.74107E-08	1.98643E-07	7.43536E-06	curli production assembly/transport component CsgE
K04338	1.14124E-06	1.53233E-07	3.0117E-07	7.4368E-06	curli production assembly/transport component CsgF
K04343	2.07636E-06	4.03462E-06	1.61151E-05	6.60016E-05	streptomycin 6-kinase [EC:2.7.1.72]
K04477	5.50723E-07	1.57572E-06	2.50765E-06	1.74328E-05	putative hydrolase
K04478	1.03544E-06	0	2.78268E-06	3.03626E-08	monofunctional glycosyltransferase [EC:2.4.1.129]
K04479	6.95695E-07	8.33064E-07	1.22778E-06	5.03143E-07	DNA polymerase IV (archaeal DinB-like DNA polymerase) [EC:2.7.7.7]
K04483	8.65702E-07	2.76116E-06	3.64347E-07	0	DNA repair protein RadA
K04484	0	3.70315E-07	2.30513E-07	2.66243E-08	DNA repair protein RadB
K04485	0.00022353	0.000224501	0.000227099	0.000227327	DNA repair protein RadA/Sms
K04486	1.53504E-06	2.32123E-06	1.57942E-05	2.37353E-06	histidinol-phosphatase (PHP family) [EC:3.1.3.15]
K04487	0.000442729	0.000441609	0.000433435	0.000332757	cysteine desulfurase [EC:2.8.1.7]
K04488	0.00022	0.000218101	0.00022297	0.000173491	nitrogen fixation protein NifU and related proteins
K04496	9.72326E-12	2.20755E-09	3.0778E-07	2.69124E-08	C-terminal binding protein
K04516	3.50854E-07	1.54493E-06	3.18993E-06	3.14447E-06	chorismate mutase [EC:5.4.99.5]
K04517	0.000222645	0.000220926	0.000225507	0.000209829	prephenate dehydrogenase [EC:1.3.1.12]
K04518	0.000218466	0.000217343	0.000202322	0.000128085	prephenate dehydratase [EC:4.2.1.51]
K04561	0.000214959	0.000214071	0.00019597	0.000115341	nitric oxide reductase subunit B [EC:1.7.2.5]
K04562	0.000219667	0.000220132	0.000218804	0.000218961	flagellar biosynthesis protein FlhG
K04564	0.000226578	0.000229192	0.00024425	0.000372171	superoxide dismutase, Fe-Mn family [EC:1.15.1.1]
K04565	0.00021907	0.000218564	0.000210481	0.000255872	superoxide dismutase, Cu-Zn family [EC:1.15.1.1]
K04566	1.78253E-06	2.26422E-07	7.11061E-07	2.32815E-07	lysyl-tRNA synthetase, class I [EC:6.1.1.6]
K04567	0.000223596	0.000224987	0.000234884	0.000232509	lysyl-tRNA synthetase, class II [EC:6.1.1.6]
K04568	3.62362E-06	1.63392E-06	3.32569E-06	6.45803E-05	elongation factor P--(R)-beta-lysine ligase [EC:6.3.1.-]
K04618	5.10905E-09	1.34034E-08	3.06707E-07	2.66243E-08	galactose oxidase [EC:1.1.3.9]
K04651	4.77888E-07	6.73724E-07	1.4523E-06	6.92762E-06	hydrogenase nickel incorporation protein HypA/HybF
K04652	1.97933E-06	4.27466E-06	2.77812E-06	8.73303E-06	hydrogenase nickel incorporation protein HypB
K04653	1.09173E-06	9.37781E-07	2.18034E-06	8.74725E-06	hydrogenase expression/formation protein HypC
K04654	8.57929E-07	8.35538E-07	1.49643E-06	6.67047E-06	hydrogenase expression/formation protein HypD
K04655	9.47853E-07	9.1182E-07	1.5458E-06	6.68037E-06	hydrogenase expression/formation protein HypE
K04656	8.7062E-07	9.73119E-07	1.70898E-06	6.71392E-06	hydrogenase maturation protein HypF
K04691	0.000222316	0.000224565	0.000221795	0.000241238	serine protease DegS [EC:3.4.21.-]
K04708	3.66417E-08	1.14301E-08	6.46823E-08	1.96807E-06	3-dehydrosphinganine reductase [EC:1.1.1.102]
K04712	0	0	2.19096E-08	1.52625E-08	sphingolipid 4-desaturase/C4-monooxygenase [EC:1.14.19.17 1.14.18.5]
K04719	0.000216946	0.000215894	0.000209861	0.000151325	5,6-dimethylbenzimidazole synthase [EC:1.13.11.79]
K04720	0.000215055	0.000213207	0.000189079	0.000114522	threonine-phosphate decarboxylase [EC:4.1.1.81]
K04739	0	0	1.04657E-08	1.60743E-08	cAMP-dependent protein kinase regulator
K04744	0.000219465	0.000217355	0.000207998	0.000217664	LPS-assembly protein
K04747	0	8.0909E-08	2.68264E-08	0	nitric oxide reductase NorF protein
K04748	2.29305E-06	4.48186E-06	1.79602E-05	3.49867E-05	nitric oxide reductase NorQ protein
K04749	1.82825E-06	6.36914E-07	5.60939E-06	1.58041E-06	anti-sigma B factor antagonist
K04750	0.000431963	0.000430821	0.00039739	0.00033055	PhnB protein
K04751	0.000652949	0.000649328	0.000619208	0.000592133	nitrogen regulatory protein P-II 1
K04752	0.000437574	0.000433581	0.000414531	0.000438748	nitrogen regulatory protein P-II 2
K04753	2.53186E-07	3.90681E-07	5.02199E-07	4.96489E-06	suppressor of ftsI
K04754	0.000217876	0.000217534	0.000209686	0.000217757	phospholipid-binding lipoprotein MlaA
K04755	0.000219759	0.000219234	0.000218801	0.00019678	ferredoxin, 2Fe-2S
K04756	0.000214045	0.000212525	0.000187976	0.000115177	alkyl hydroperoxide reductase subunit D
K04757	1.18962E-06	7.80012E-07	6.3539E-06	4.99389E-06	serine/threonine-protein kinase RsbW [EC:2.7.11.1]
K04758	0.000222057	0.00021957	0.000227533	0.000215339	ferrous iron transport protein A
K04759	0.000222656	0.000220534	0.000229918	0.000265857	ferrous iron transport protein B
K04760	0.000216673	0.000216948	0.000207101	0.000219432	transcription elongation factor GreB
K04761	0.001103868	0.001107196	0.001134337	0.001252689	LysR family transcriptional regulator, hydrogen peroxide-inducible genes activator
K04762	0.000222804	0.000222255	0.000219694	0.000222065	ribosome-associated heat shock protein Hsp15
K04763	0.000456107	0.000462258	0.000483872	0.000430918	integrase/recombinase XerD
K04764	0.000220332	0.000217188	0.000211701	0.000217728	integration host factor subunit alpha
K04765	6.39091E-06	4.06409E-06	1.73864E-05	7.32634E-05	nucleoside triphosphate diphosphatase [EC:3.6.1.9]
K04766	3.05232E-07	2.06813E-07	6.28068E-07	1.2618E-08	acetoin utilization protein AcuA [EC:2.3.1.-]
K04767	4.62897E-06	4.71737E-06	2.23272E-05	0.000109289	acetoin utilization protein AcuB
K04768	3.5573E-06	4.03818E-06	5.0499E-06	5.51583E-06	acetoin utilization protein AcuC
K04769	8.84619E-07	7.36877E-07	1.33236E-06	3.47902E-08	AbrB family transcriptional regulator, stage V sporulation protein T
K04770	1.39226E-06	8.35733E-07	2.45162E-06	2.71571E-05	Lon-like ATP-dependent protease [EC:3.4.21.-]
K04771	0.00067134	0.000668743	0.000684417	0.000649588	serine protease Do [EC:3.4.21.107]
K04772	0.000223976	0.000225051	0.000222136	0.000277069	serine protease DegQ [EC:3.4.21.-]
K04773	0.000435379	0.000431119	0.000399305	0.000333401	protease IV [EC:3.4.21.-]
K04774	2.69666E-06	9.10132E-07	2.53583E-06	1.77771E-05	serine protease SohB [EC:3.4.21.-]
K04775	1.13071E-06	3.24577E-07	3.03647E-07	8.63271E-07	protease YdgD [EC:3.4.21.-]
K04780	0.001295059	0.001288882	0.001203271	0.000878239	nonribosomal peptide synthetase DhbF
K04781	7.49459E-08	5.23924E-08	2.87079E-08	2.88893E-06	salicylate synthetase [EC:5.4.4.2 4.2.99.21]
K04782	1.91744E-06	3.02641E-06	1.7096E-05	3.52081E-05	isochorismate pyruvate lyase [EC:4.2.99.21]
K04783	4.35729E-07	1.62662E-07	4.62151E-07	1.04743E-05	yersiniabactin salicyl-AMP ligase [EC:6.3.2.-]
K04784	0.000860889	0.000853318	0.00077809	0.000521265	yersiniabactin nonribosomal peptide synthetase
K04785	0.000213795	0.000211794	0.000186117	0.000116046	yersiniabactin synthetase, thiazolinyl reductase component
K04786	0.000431209	0.000428124	0.000396387	0.000280767	yersiniabactin nonribosomal peptide/polyketide synthase
K04787	1.45274E-06	1.10772E-06	6.60595E-06	4.65677E-05	mycobactin salicyl-AMP ligase [EC:6.3.2.-]
K04788	0.000435922	0.0004346	0.000424256	0.00036216	mycobactin phenyloxazoline synthetase
K04789	0.001077364	0.001073782	0.000997814	0.000721487	mycobactin peptide synthetase MbtE

K04790	9.7937E-08	4.04496E-07	3.24864E-06	2.09368E-06	mycobactin polyketide synthetase MbtC
K04791	0	1.23941E-07	4.68279E-07	8.42698E-08	mycobactin polyketide synthetase MbtD
K04792	0.000431901	0.00043069	0.000409626	0.000359207	mycobactin peptide synthetase MbtF
K04793	0	1.23941E-07	5.44821E-08	2.82158E-08	mycobactin lysine-N-oxygenase
K04794	2.57365E-07	5.59131E-07	1.19317E-06	5.7591E-08	peptidyl-tRNA hydrolase, PTH2 family [EC:3.1.1.29]
K04799	7.13521E-08	0	6.6378E-08	3.42565E-07	flap endonuclease-1 [EC:3.-.-.-]
K04800	2.48332E-08	2.18949E-07	2.62943E-07	7.49209E-08	replication factor C large subunit
K04801	1.44434E-10	1.35949E-07	1.65697E-07	3.55487E-08	replication factor C small subunit
K04835	1.08047E-07	5.25813E-09	9.73945E-08	5.77874E-08	methylaspartate ammonia-lyase [EC:4.3.1.2]
K04844	1.23701E-06	2.41296E-07	5.10458E-07	3.00212E-07	hypothetical glycosyl hydrolase [EC:3.2.1.-]
K04940	1.06698E-06	2.06923E-07	3.15707E-06	6.64126E-08	opine dehydrogenase [EC:1.5.1.28]
K05020	1.13956E-05	1.85324E-05	3.72438E-05	0.000224873	glycine betaine transporter
K05245	4.41869E-09	5.96525E-07	3.46107E-07	1.28677E-06	L-carnitine/gamma-butyrobetaine antiporter
K05275	0.000229961	0.000235305	0.000258876	0.000361939	pyridoxine 4-dehydrogenase [EC:1.1.1.65]
K05281	1.38766E-07	2.6325E-08	1.0713E-06	6.17955E-06	2-hydroxyisoflavone reductase [EC:1.3.1.45]
K05296	0.00021716	0.000215865	0.000205155	0.000162736	3(or 17)beta-hydroxysteroid dehydrogenase [EC:1.1.1.51]
K05297	0.000220816	0.000215647	0.000198292	0.000170428	rubredoxin---NAD+ reductase [EC:1.18.1.1]
K05299	6.26338E-07	4.41951E-07	1.08191E-06	5.61429E-06	formate dehydrogenase (NADP+) alpha subunit [EC:1.17.1.10]
K05303	2.40598E-07	2.08296E-08	2.13585E-07	1.17065E-06	O-methyltransferase [EC:2.1.1.-]
K05304	0	0	4.91986E-08	1.8372E-06	sialic acid synthase [EC:2.5.1.56 2.5.1.57 2.5.1.132]
K05305	1.79571E-07	7.03627E-07	3.09405E-07	1.53089E-09	fucokinase [EC:2.7.1.52]
K05306	1.62874E-06	4.121E-06	1.4997E-05	4.15785E-05	phosphonoacetaldehyde hydrolase [EC:3.11.1.1]
K05308	1.01402E-07	1.2139E-07	1.25823E-07	1.74175E-08	gluconate/galactonate dehydratase [EC:4.2.1.140]
K05311	1.1772E-06	2.76783E-07	3.6095E-06	8.05393E-07	central glycolytic genes regulator
K05337	2.36525E-06	1.79272E-06	9.73049E-06	8.40517E-06	ferredoxin
K05338	9.65439E-07	4.42717E-08	2.94877E-06	8.4053E-07	holin-like protein
K05339	1.41644E-06	5.71609E-07	3.3614E-06	3.88194E-06	holin-like protein LrgB
K05340	2.32137E-06	6.63572E-07	6.9501E-06	1.3074E-06	glucose uptake protein
K05341	1.41819E-06	8.3022E-07	1.14788E-05	2.46316E-06	amylosucrase [EC:2.4.1.4]
K05342	1.33319E-06	2.02848E-06	1.68098E-05	5.09661E-06	alpha,alpha-trehalose phosphorylase [EC:2.4.1.64]
K05343	0.000221352	0.000218603	0.000220789	0.000173549	maltose alpha-D-glucosyltransferase / alpha-amylase [EC:5.4.99.16 3.2.1.1]
K05346	1.11306E-06	1.313E-06	1.19772E-05	2.43359E-06	deoxyribonucleoside regulator
K05349	7.55641E-06	1.21072E-05	4.70787E-05	0.000199736	beta-glucosidase [EC:3.2.1.21]
K05350	2.03667E-06	2.02231E-06	2.24934E-05	2.84702E-05	beta-glucosidase [EC:3.2.1.21]
K05351	5.21228E-07	3.91551E-07	2.94331E-06	3.50312E-07	D-xylulose reductase [EC:1.1.1.9]
K05352	1.06485E-06	1.59585E-07	2.71465E-06	1.29072E-06	ribitol-5-phosphate 2-dehydrogenase (NADP+) [EC:1.1.1.405]
K05356	2.91863E-07	1.23151E-08	2.48894E-07	1.82987E-07	all-trans-nonaprenyl-diphosphate synthase [EC:2.5.1.84 2.5.1.85]
K05358	2.93723E-06	4.2333E-06	1.88279E-05	0.000104928	quinate dehydrogenase (quinone) [EC:1.1.5.8]
K05362	1.21679E-06	1.04875E-07	6.59342E-06	3.6383E-06	UDP-N-acetylmuramoyl-L-alanyl-D-glutamate-L-lysine ligase [EC:6.3.2.7]
K05363	9.19761E-08	6.30272E-08	3.0383E-06	3.57821E-06	serine/alanine adding enzyme [EC:2.3.2.10]
K05364	4.77669E-06	1.02922E-05	2.83372E-05	3.8797E-05	penicillin-binding protein A
K05365	6.64918E-06	6.59018E-06	1.05822E-05	7.32376E-05	penicillin-binding protein 1B [EC:2.4.1.129 3.4.16.4]
K05366	0.00023429	0.00023381	0.000276109	0.00031571	penicillin-binding protein 1A [EC:2.4.1.129 3.4.16.4]
K05367	4.02423E-06	6.12579E-06	2.27008E-05	9.22209E-05	penicillin-binding protein 1C [EC:2.4.1.129]
K05368	9.82118E-08	3.14581E-07	4.76032E-07	1.73048E-05	aquacobalamin reductase / NAD(P)H-flavin reductase [EC:1.16.1.3 1.5.1.41]
K05372	2.52337E-08	3.6378E-08	1.47554E-07	1.39703E-07	AraC family transcriptional regulator
K05373	7.34288E-08	1.0085E-07	8.29095E-07	6.21231E-06	MFS transporter, putative signal transducer
K05374	2.51819E-08	2.0027E-08	5.97724E-08	4.43625E-06	yersiniabactin synthetase, thioesterase component
K05375	0.0004291	0.000427317	0.000391275	0.000269361	MbtH protein
K05396	9.60238E-07	6.50466E-07	2.51095E-06	4.20704E-06	D-cysteine desulfhydrase [EC:4.4.1.15]
K05499	0.000430141	0.000425851	0.000382518	0.000314348	LacI family transcriptional regulator, repressor for deo operon, udp, cdd, tsx, nupC, and nupG
K05501	0.000215184	0.000215196	0.000203311	0.000165583	TetR/AcrR family transcriptional regulator
K05515	0.000222284	0.000223946	0.000223867	0.000236431	penicillin-binding protein 2 [EC:3.4.16.4]
K05516	0.000224933	0.000231121	0.000240014	0.000242393	curved DNA-binding protein
K05517	4.40677E-07	1.17333E-06	6.08483E-07	1.40379E-05	nucleoside-specific channel-forming protein
K05518	1.24809E-07	9.5208E-10	5.28596E-07	0	phosphoserine phosphatase RsbX [EC:3.1.3.3]
K05519	0	2.05861E-07	4.61104E-08	0	transcriptional activator of comK gene
K05520	0.000224988	0.000226522	0.000221453	0.00020808	protease I [EC:3.5.1.124]
K05521	2.1314E-06	3.70555E-06	1.76622E-05	7.14179E-05	ADP-ribosyl-[dinitrogen reductase] hydrolase [EC:3.2.2.24]
K05522	3.71305E-06	9.51268E-06	1.47161E-05	4.03433E-05	endonuclease VIII [EC:3.2.2.- 4.2.99.18]
K05523	1.17647E-06	1.80579E-06	6.63984E-06	4.52726E-05	D-lactate dehydratase / protein deglycase [EC:4.2.1.130 3.5.1.124]
K05524	0.000435444	0.00043423	0.000404988	0.000328623	ferredoxin
K05525	9.18352E-08	2.37239E-07	4.44014E-07	1.7507E-06	linalool 8-monooxygenase [EC:1.14.14.84]
K05526	7.50166E-07	4.32247E-07	1.72474E-06	2.08109E-05	succinylglutamate desuccinylase [EC:3.5.1.96]
K05527	0.000436713	0.000433757	0.000413251	0.000426371	BolA family transcriptional regulator, general stress-responsive regulator
K05535	0	0	3.05555E-08	0	alpha 1,2-mannosyltransferase [EC:2.4.1.-]
K05539	0.000220931	0.000218031	0.000212084	0.000218169	tRNA-dihydrouridine synthase A [EC:1.-.-.-]
K05540	0.000222598	0.000223977	0.000224999	0.000226032	tRNA-dihydrouridine synthase B [EC:1.-.-.-]
K05541	0.000217246	0.000216213	0.000205304	0.000167764	tRNA-dihydrouridine synthase C [EC:1.-.-.-]
K05548	0.000216706	0.000218503	0.000219358	0.000186197	MFS transporter, AAHS family, benzoate transport protein
K05549	4.02937E-07	1.02889E-06	2.91675E-06	2.53049E-06	benzoate/toluate 1,2-dioxygenase subunit alpha [EC:1.14.12.10 1.14.12.-]
K05550	1.5756E-07	6.21321E-07	1.42877E-06	1.32756E-06	benzoate/toluate 1,2-dioxygenase subunit beta [EC:1.14.12.10 1.14.12.-]
K05551	7.3094E-07	7.39586E-07	2.25247E-06	2.62125E-06	minimal PKS ketosynthase (KS/KS alpha) [EC:2.3.1.- 2.3.1.260 2.3.1.235]
K05552	1.53799E-07	6.2087E-08	3.9865E-07	6.99982E-08	minimal PKS chain-length factor (CLF/KS beta) [EC:2.3.1.- 2.3.1.260 2.3.1.235]
K05553	0	0	1.749E-07	1.78604E-09	minimal PKS acyl carrier protein
K05554	0	0	1.87751E-07	1.78604E-09	aromatase [EC:4.2.1.-]
K05555	1.76101E-06	3.32342E-06	2.0477E-05	3.59039E-05	cyclase [EC:4.-.-.-]
K05556	1.04701E-06	4.36745E-07	8.73378E-07	1.9905E-06	ketoreductase RED1 [EC:1.1.1.-]
K05558	0	1.06439E-08	1.13779E-07	2.07064E-06	pyridoxamine 5-phosphate oxidase family protein
K05559	7.10473E-06	1.23743E-05	3.11917E-05	9.66318E-05	multicomponent K+:H+ antiporter subunit A
K05560	4.11055E-06	4.79569E-06	2.31382E-05	9.05413E-05	multicomponent K+:H+ antiporter subunit C
K05561	4.12223E-06	8.555E-06	2.10423E-05	9.43736E-05	multicomponent K+:H+ antiporter subunit D
K05562	3.07405E-06	4.79185E-06	1.96922E-05	9.04742E-05	multicomponent K+:H+ antiporter subunit E
K05563	3.14997E-06	4.83224E-06	2.04394E-05	9.04978E-05	multicomponent K+:H+ antiporter subunit F
K05564	3.07405E-06	4.93347E-06	1.96931E-05	9.04778E-05	multicomponent K+:H+ antiporter subunit G
K05565	8.1876E-06	1.36412E-05	3.58864E-05	9.71709E-05	multicomponent Na+:H+ antiporter subunit A
K05566	5.28599E-06	6.83592E-06	1.11194E-05	4.68064E-06	multicomponent Na+:H+ antiporter subunit B

K05567	6.78293E-06	8.84982E-06	1.46201E-05	5.7969E-05	multicomponent Na+:H+ antiporter subunit C
K05568	9.20127E-06	1.10712E-05	1.84832E-05	6.30864E-05	multicomponent Na+:H+ antiporter subunit D
K05569	4.4304E-06	8.8835E-06	1.25708E-05	6.29484E-06	multicomponent Na+:H+ antiporter subunit E
K05570	5.18404E-06	9.39376E-06	1.2808E-05	5.98472E-05	multicomponent Na+:H+ antiporter subunit F
K05571	5.13632E-06	8.89339E-06	1.17266E-05	6.33922E-06	multicomponent Na+:H+ antiporter subunit G
K05572	4.08517E-08	0	6.14351E-08	0	NAD(P)H-quinone oxidoreductase subunit 1 [EC:1.6.5.3]
K05573	1.03107E-06	7.66506E-09	1.34075E-06	1.90174E-09	NAD(P)H-quinone oxidoreductase subunit 2 [EC:1.6.5.3]
K05574	0	2.05861E-07	1.72617E-06	0	NAD(P)H-quinone oxidoreductase subunit 3 [EC:1.6.5.3]
K05575	1.11008E-06	4.7711E-07	1.66128E-06	2.56326E-06	NAD(P)H-quinone oxidoreductase subunit 4 [EC:1.6.5.3]
K05576	2.44485E-08	2.06606E-07	4.97104E-07	8.26472E-07	NAD(P)H-quinone oxidoreductase subunit 4L [EC:1.6.5.3]
K05577	1.19046E-06	4.26502E-07	7.69373E-06	5.02856E-06	NAD(P)H-quinone oxidoreductase subunit 5 [EC:1.6.5.3]
K05578	2.997E-09	1.02324E-06	3.43374E-07	3.03455E-08	NAD(P)H-quinone oxidoreductase subunit 6 [EC:1.6.5.3]
K05579	0	1.02051E-08	6.3169E-08	1.83232E-08	NAD(P)H-quinone oxidoreductase subunit H [EC:1.6.5.3]
K05580	3.2196E-07	1.32452E-07	2.96159E-07	3.80348E-09	NAD(P)H-quinone oxidoreductase subunit I [EC:1.6.5.3]
K05581	0	0	4.30523E-08	1.73885E-08	NAD(P)H-quinone oxidoreductase subunit J [EC:1.6.5.3]
K05582	7.29798E-08	0	1.23191E-07	3.86349E-08	NAD(P)H-quinone oxidoreductase subunit K [EC:1.6.5.3]
K05586	1.06571E-07	4.03206E-08	6.40274E-07	1.12829E-07	bidirectional [NiFe] hydrogenase diaphorase subunit [EC:1.6.5.3]
K05587	7.76308E-07	5.07208E-07	1.56384E-06	2.12697E-07	bidirectional [NiFe] hydrogenase diaphorase subunit [EC:1.6.5.3]
K05588	9.16819E-09	5.50297E-08	6.17387E-07	6.78334E-09	bidirectional [NiFe] hydrogenase diaphorase subunit [EC:1.6.5.3]
K05589	0.000217676	0.00021691	0.000207295	0.000217472	cell division protein FtsB
K05590	0.000220993	0.000222227	0.00022599	0.000238619	ATP-dependent RNA helicase SrmB [EC:3.6.4.13]
K05591	0.000446103	0.000454169	0.000492901	0.000614394	ATP-independent RNA helicase DbpA [EC:3.6.4.13]
K05592	0.000453352	0.00046337	0.000514241	0.00074353	ATP-dependent RNA helicase DeaD [EC:3.6.4.13]
K05593	3.5707E-07	2.14306E-07	1.51846E-06	3.16169E-06	aminoglycoside 6-adenylyltransferase [EC:2.7.7.-]
K05594	1.01191E-07	3.01003E-07	8.55765E-08	8.03496E-07	ElaB protein
K05595	0.000433654	0.00043178	0.000399009	0.000323342	multiple antibiotic resistance protein
K05596	0.000442273	0.0004455	0.000446789	0.000564336	LysR family transcriptional regulator, chromosome initiation inhibitor
K05597	2.4928E-06	3.5864E-06	2.02931E-05	3.5139E-05	glutamin-(asparagin-)ase [EC:3.5.1.38]
K05599	5.28395E-08	7.1032E-08	2.69803E-07	4.68242E-07	anthranilate 1,2-dioxygenase (deaminating, decarboxylating) large subunit [EC:1.14.12.1]
K05600	1.14015E-08	2.78522E-08	8.78085E-07	4.9694E-08	anthranilate 1,2-dioxygenase (deaminating, decarboxylating) small subunit [EC:1.14.12.1]
K05601	1.93212E-06	2.40453E-06	4.96996E-06	1.05982E-05	hydroxylamine reductase [EC:1.7.99.1]
K05602	3.94497E-06	4.87643E-06	1.78708E-05	5.80684E-05	histidinol-phosphatase [EC:3.1.3.15]
K05603	3.36404E-06	5.00293E-06	2.55454E-05	8.69006E-05	formimidoylglutamate deiminase [EC:3.5.3.13]
K05606	2.76374E-06	3.14674E-06	1.14529E-05	3.17638E-06	methylmalonyl-CoA/ethylmalonyl-CoA epimerase [EC:5.1.99.1]
K05685	1.7257E-05	2.78721E-05	9.19772E-05	0.000338898	macrolide transport system ATP-binding/permease protein [EC:3.6.3.-]
K05708	3.93839E-07	7.23462E-07	4.12645E-06	9.29256E-06	3-phenylpropionate/trans-cinnamate dioxygenase subunit alpha [EC:1.14.12.19]
K05709	1.1349E-07	4.95038E-08	5.08801E-07	9.00662E-07	3-phenylpropionate/trans-cinnamate dioxygenase subunit beta [EC:1.14.12.19]
K05710	3.63744E-06	5.73392E-06	1.19719E-05	5.83702E-05	3-phenylpropionate/trans-cinnamate dioxygenase ferredoxin component
K05711	3.91717E-07	7.60913E-07	3.86939E-06	8.03676E-06	2,3-dihydroxy-2,3-dihydrophenylpropionate dehydrogenase [EC:1.3.1.87]
K05712	0.000434692	0.000437915	0.000415973	0.000377702	3-(3-hydroxy-phenyl)propionate hydroxylase [EC:1.14.13.127]
K05713	1.34167E-06	3.10997E-06	1.41579E-05	3.67878E-05	2,3-dihydroxyphenylpropionate 1,2-dioxygenase [EC:1.13.11.16]
K05714	1.11033E-06	2.24371E-07	7.15867E-07	7.13622E-06	2-hydroxy-6-oxonona-2,4-dienedioate hydrolase [EC:3.7.1.14]
K05715	0	0	3.13971E-08	0	2-phosphoglycerate kinase [EC:2.7.2.-]
K05739	6.4392E-07	4.73548E-07	5.23927E-07	7.60696E-09	uncharacterized protein
K05770	3.61381E-06	2.08447E-06	5.26034E-06	4.32489E-05	translocator protein
K05772	0.000214938	0.000213616	0.000195176	0.000139319	tungstate transport system substrate-binding protein
K05773	0.00021419	0.000211852	0.000186743	0.000118514	tungstate transport system permease protein
K05774	1.12119E-06	9.60723E-07	1.66817E-06	9.73072E-07	ribose 1,5-bisphosphokinase [EC:2.7.4.23]
K05775	9.82118E-08	3.83357E-07	5.53699E-07	1.44776E-05	maltose operon periplasmic protein
K05776	2.19471E-06	2.81049E-06	1.07621E-05	1.456E-05	molybdate transport system ATP-binding protein
K05777	9.20103E-08	8.41813E-08	9.04962E-07	1.55859E-05	putative thiamine transport system substrate-binding protein
K05778	3.28192E-08	5.8693E-08	5.39299E-07	1.55037E-05	putative thiamine transport system permease protein
K05779	1.89423E-06	1.007E-06	3.36434E-06	7.29453E-05	putative thiamine transport system ATP-binding protein
K05780	2.61228E-06	1.56925E-06	7.21382E-06	5.59371E-06	alpha-D-ribose 1-methylphosphonate 5-triphosphate synthase subunit PhnL [EC:2.7.8.37]
K05781	2.6135E-06	1.12815E-06	8.8358E-06	6.41742E-06	putative phosphonate transport system ATP-binding protein
K05782	4.71099E-06	8.38597E-06	3.62747E-05	0.000130406	benzoate membrane transport protein
K05783	3.2664E-06	5.30016E-06	2.1679E-05	8.02589E-05	dihydroxycyclohexadiene carboxylate dehydrogenase [EC:1.3.1.25 1.3.1.-]
K05784	1.57973E-07	6.04971E-07	1.89825E-06	2.31357E-06	benzoate/toluate 1,2-dioxygenase reductase component [EC:1.18.1.-]
K05785	3.57335E-07	4.27595E-07	8.41459E-07	1.50547E-05	transcriptional antiterminator RfaH
K05786	0.000221971	0.000218657	0.000201399	0.000196987	chloramphenicol-sensitive protein RarD
K05787	7.02204E-07	1.81656E-06	2.18289E-06	2.87112E-05	DNA-binding protein HU-alpha
K05788	0.000648818	0.000642561	0.000582049	0.000449915	integration host factor subunit beta
K05789	2.73966E-08	6.86278E-08	2.49531E-07	6.74961E-07	chain length determinant protein (polysaccharide antigen chain regulator)
K05790	9.82118E-08	3.01003E-07	7.49218E-08	8.11175E-07	lipopolysaccharide biosynthesis protein WzzE
K05791	3.54026E-06	1.94337E-07	2.82024E-06	4.31525E-06	tellurium resistance protein TerZ
K05792	1.67326E-06	3.38786E-08	1.83264E-06	6.46406E-06	tellurite resistance protein TerA
K05793	5.46078E-08	5.18697E-08	1.6483E-07	2.28872E-06	tellurite resistance protein TerB
K05794	6.48365E-06	9.36868E-06	1.80183E-05	7.01461E-05	tellurite resistance protein TerC
K05795	4.7806E-06	2.26141E-07	4.81239E-06	1.04128E-05	tellurium resistance protein TerD
K05796	9.35684E-08	1.25097E-07	8.04589E-07	6.16831E-06	electron transport protein HydN
K05797	1.59235E-07	1.77403E-07	5.77143E-07	2.47753E-07	4-cresol dehydrogenase (hydroxylating) flavoprotein subunit [EC:1.17.99.1]
K05798	0.000429063	0.000428182	0.000391231	0.000277137	LysR family transcriptional regulator, transcriptional activator for leuABCD operon
K05799	0.00045083	0.00046111	0.000536467	0.000702211	GntR family transcriptional regulator, transcriptional repressor for pyruvate dehydrogenase complex
K05800	0.00086596	0.000863853	0.000816841	0.000693123	Lrp/AsnC family transcriptional regulator
K05801	3.12367E-06	3.61567E-06	4.80136E-06	7.31734E-05	DnaJ like chaperone protein
K05802	0.000220954	0.000222577	0.000231526	0.000269004	potassium-dependent mechanosensitive channel
K05803	9.87527E-08	3.15286E-07	3.63936E-07	1.71946E-05	lipoprotein NlpI
K05804	7.03739E-07	2.41391E-06	3.84691E-07	5.17427E-06	AraC family transcriptional regulator, mar-sox-rob regulon activator
K05805	0.000216487	0.000215751	0.000203638	0.000164867	CreA protein
K05807	0.000219884	0.00021889	0.00021141	0.000217786	outer membrane protein assembly factor BamD
K05808	0.000220171	0.000220678	0.000226041	0.000230792	putative sigma-54 modulation protein
K05809	1.60122E-07	3.83357E-07	4.29265E-07	1.37769E-05	ribosome-associated inhibitor A
K05810	0.000224523	0.000223004	0.000226202	0.000224108	polyphenol oxidase [EC:1.10.3.-]
K05811	2.83682E-08	1.95572E-08	5.50684E-08	8.00866E-07	putative lipoprotein
K05812	2.00097E-06	4.21906E-06	1.64486E-05	5.93973E-05	DTW domain-containing protein
K05813	0.000217158	0.000219434	0.000220557	0.00018939	sn-glycerol 3-phosphate transport system substrate-binding protein
K05814	0.000217365	0.000219988	0.000220807	0.000197442	sn-glycerol 3-phosphate transport system permease protein

K05815	0.000218865	0.00022335	0.000227326	0.000190628	sn-glycerol 3-phosphate transport system permease protein
K05816	0.000665418	0.000683633	0.000716619	0.000749694	sn-glycerol 3-phosphate transport system ATP-binding protein [EC:3.6.3.20]
K05817	0.000216452	0.000214488	0.000200443	0.000136154	LysR family transcriptional regulator, hca operon transcriptional activator
K05818	9.05205E-08	5.33273E-08	2.73587E-06	1.30426E-06	IclR family transcriptional regulator, mhp operon transcriptional activator
K05819	0.000214641	0.000212473	0.000187344	0.000143141	MFS transporter, AAHS family, 3-hydroxyphenylpropionic acid transporter
K05820	3.95852E-06	1.71773E-06	7.59139E-06	5.49444E-05	MFS transporter, PPP family, 3-phenylpropionic acid transporter
K05822	1.66675E-06	7.11228E-07	8.75138E-06	4.79275E-06	tetrahydrodipicolinate N-acetyltransferase [EC:2.3.1.89]
K05823	0.000224306	0.000227651	0.000247493	0.000274553	N-acetyldiaminopimelate deacetylase [EC:3.5.1.47]
K05824	4.57601E-08	2.90903E-08	3.66293E-07	1.17285E-07	homoisocitrate dehydrogenase [EC:1.1.1.87]
K05825	0.000652567	0.000656401	0.000652816	0.00055712	2-aminoadipate transaminase [EC:2.6.1.-]
K05826	3.31529E-08	0	1.1263E-07	0	alpha-aminoadipate/glutamate carrier protein LysW
K05827	0	0	1.14366E-07	0	[lysine-biosynthesis-protein LysW]---L-2-aminoadipate ligase [EC:6.3.2.43]
K05828	0	0	3.33112E-07	0	LysW-gamma-L-alpha-aminoadipate/LysW-L-glutamate kinase [EC:2.7.2.-]
K05829	3.69596E-07	6.74352E-07	2.35981E-06	1.55778E-07	LysW-gamma-L-alpha-aminoadipyl-6-phosphate/LysW-L-glutamyl-5-phosphate reductase [EC:1.2.1.-]
K05830	2.52549E-06	3.12251E-06	2.85704E-06	1.28248E-06	LysW-gamma-L-lysine/LysW-L-ornithine aminotransferase [EC:2.6.1.-]
K05831	0	0	8.36842E-08	0	LysW-gamma-L-lysine/LysW-L-ornithine carboxypeptidase
K05832	0.00021905	0.000220002	0.000212402	0.000163973	putative ABC transport system permease protein
K05833	0.000220504	0.000223009	0.000215918	0.000168939	putative ABC transport system ATP-binding protein
K05834	0.000436422	0.000439122	0.000445074	0.000470161	homoserine/homoserine lactone efflux protein
K05835	5.37673E-07	1.87709E-06	6.05331E-06	3.53116E-05	threonine efflux protein
K05836	0.00021737	0.0002162	0.000208058	0.00021927	GntR family transcriptional regulator, histidine utilization repressor
K05837	0.000226636	0.00022754	0.000235857	0.000276446	rod shape determining protein RodA
K05838	0.000221097	0.000222439	0.000220646	0.000224368	putative thioredoxin
K05839	1.99404E-07	6.12611E-07	1.20168E-07	1.05146E-06	haemolysin expression modulating protein
K05841	6.74678E-07	4.84133E-07	1.55546E-06	4.16915E-05	sterol 3beta-glucosyltransferase [EC:2.4.1.173]
K05844	4.35171E-06	9.46406E-06	5.14515E-06	8.47215E-05	ribosomal protein S6--L-glutamate ligase [EC:6.3.2.-]
K05845	0.000224604	0.000224993	0.000219407	0.00013248	osmoprotectant transport system substrate-binding protein
K05846	0.000444416	0.000442104	0.00042583	0.000253009	osmoprotectant transport system permease protein
K05847	0.000900397	0.000905124	0.000992931	0.000908055	osmoprotectant transport system ATP-binding protein
K05851	9.82831E-08	7.51932E-07	1.47918E-06	1.39129E-05	adenylate cyclase, class 1 [EC:4.6.1.1]
K05873	1.52211E-07	1.02568E-07	5.36789E-07	7.50243E-06	adenylate cyclase, class 2 [EC:4.6.1.1]
K05874	0.003665268	0.003658371	0.003473661	0.003018443	methyl-accepting chemotaxis protein I, serine sensor receptor
K05875	0.002379494	0.00238506	0.002346997	0.002201943	methyl-accepting chemotaxis protein II, aspartate sensor receptor
K05876	0.000652165	0.000652602	0.000641753	0.000732304	methyl-accepting chemotaxis protein III, ribose and galactose sensor receptor
K05877	0.001507331	0.001507787	0.001428932	0.001109158	methyl-accepting chemotaxis protein IV, peptide sensor receptor
K05878	5.1385E-06	5.77126E-06	3.08566E-05	4.42204E-05	phosphoenolpyruvate---glycerone phosphotransferase subunit DhaK [EC:2.7.1.121]
K05879	4.25231E-06	5.29789E-06	2.98302E-05	4.27128E-05	phosphoenolpyruvate---glycerone phosphotransferase subunit DhaL [EC:2.7.1.121]
K05880	1.11873E-07	5.47828E-07	8.77662E-07	4.72789E-06	transcriptional activator for dhaKLM operon
K05881	1.90867E-06	8.04703E-07	1.02428E-05	6.09897E-06	phosphoenolpyruvate---glycerone phosphotransferase subunit DhaM [EC:2.7.1.121]
K05882	0.000228302	0.000230571	0.000252124	0.000333591	aryl-alcohol dehydrogenase (NADP+) [EC:1.1.1.91]
K05884	8.99101E-09	4.99842E-09	1.80761E-07	6.35528E-08	L-2-hydroxycarboxylate dehydrogenase (NAD+) [EC:1.1.1.337]
K05886	0.000215095	0.000213925	0.000189229	0.000164924	serine 3-dehydrogenase (NADP+) [EC:1.1.1.276]
K05887	7.54131E-09	2.28149E-08	4.05817E-08	8.47945E-07	quinate/shikimate dehydrogenase [EC:1.1.1.282]
K05889	6.12846E-08	3.39321E-09	3.40506E-07	1.0175E-08	polyvinyl alcohol dehydrogenase (cytochrome) [EC:1.1.2.6]
K05895	0.000217184	0.000215935	0.000208257	0.000151401	precorrin-6A/cobalt-precorrin-6A reductase [EC:1.3.1.54 1.3.1.106]
K05896	0.000220669	0.000218704	0.000218498	0.000215799	segregation and condensation protein A
K05898	5.72335E-07	1.02832E-06	4.4349E-06	1.5225E-05	3-oxosteroid 1-dehydrogenase [EC:1.3.99.4]
K05910	1.03036E-06	2.14876E-07	3.22645E-06	1.57749E-06	NADH peroxidase [EC:1.11.1.1]
K05913	1.60322E-08	3.23246E-08	1.06501E-07	1.54745E-08	2,4-dihydroxyacetophenone dioxygenase [EC:1.13.11.41]
K05916	0.000221426	0.00022039	0.000211239	0.000178828	nitric oxide dioxygenase [EC:1.14.12.17]
K05917	3.99601E-09	2.69171E-07	1.29017E-07	1.75467E-06	sterol 14-demethylase [EC:1.14.13.70]
K05919	6.75702E-07	7.24865E-07	3.52403E-06	5.5568E-08	superoxide reductase [EC:1.15.1.2]
K05921	1.01534E-05	1.89745E-05	6.25582E-05	0.000121267	5-oxopent-3-ene-1,2,5-tricarboxylate decarboxylase / 2-hydroxyhepta-2,4-diene-1,7-dioate isomerase [EC:4.1.1.68 5.3.
K05922	1.06355E-07	4.35106E-07	5.1559E-07	1.47202E-06	quinone-reactive Ni/Fe-hydrogenase large subunit [EC:1.12.5.1]
K05927	4.40738E-09	2.24306E-07	4.17067E-07	1.66162E-06	quinone-reactive Ni/Fe-hydrogenase small subunit [EC:1.12.5.1]
K05928	3.22817E-07	1.81251E-07	4.8791E-07	3.80348E-09	tocopherol O-methyltransferase [EC:2.1.1.95]
K05934	0.000216951	0.00021595	0.000206542	0.000150108	precorrin-3B C17-methyltransferase [EC:2.1.1.131]
K05936	0.000435043	0.000429232	0.000400263	0.000273699	precorrin-4/cobalt-precorrin-4 C11-methyltransferase [EC:2.1.1.133 2.1.1.271]
K05937	1.03461E-07	1.06045E-08	4.88673E-07	1.1555E-08	uncharacterized protein
K05939	0.000219764	0.000218653	0.000211323	0.000231359	acyl-[acyl-carrier-protein]-phospholipid O-acyltransferase / long-chain-fatty-acid--[acyl-carrier-protein] ligase [EC:2.3
K05942	8.67992E-08	1.09786E-07	1.00321E-07	9.95695E-09	citrate (Re)-synthase [EC:2.3.3.3]
K05946	4.97109E-06	3.11115E-06	7.7525E-06	1.68129E-05	N-acetylglucosaminyldiphosphoundecaprenol N-acetyl-beta-D-mannosaminyltransferase [EC:2.4.1.187]
K05951	0	1.41965E-08	5.42361E-08	7.05394E-09	NAD+---dinitrogen-reductase ADP-D-ribosyltransferase [EC:2.4.2.37]
K05952	1.54817E-07	7.43436E-07	1.49476E-06	2.63983E-05	uncharacterized protein
K05957	0	0	0	7.6937E-09	L-glutamine:scyllo-inosose aminotransferase [EC:2.6.1.50]
K05962	3.24676E-08	4.64135E-08	8.10649E-08	0	protein-histidine pros-kinase [EC:2.7.13.1]
K05964	1.99735E-07	3.07605E-07	3.59635E-07	7.69229E-06	holo-ACP synthase [EC:2.7.7.61]
K05966	2.11979E-06	5.10399E-07	8.25474E-07	7.80626E-06	triphosphoribosyl-dephospho-CoA synthase [EC:2.4.2.52]
K05967	1.75776E-07	2.23874E-07	6.95673E-07	2.66092E-08	uncharacterized protein
K05970	1.6684E-06	4.83276E-06	9.11786E-06	4.53833E-06	sialate O-acetylesterase [EC:3.1.1.53]
K05973	0.000433869	0.000428271	0.0003925	0.000313248	poly(3-hydroxybutyrate) depolymerase [EC:3.1.1.75]
K05977	0	0	1.17294E-09	1.85112E-08	2-hydroxybiphenyl-2-sulfinate desulfinase [EC:3.13.1.3]
K05978	0	0	3.48857E-09	7.72481E-08	glucosylglycerol 3-phosphatase [EC:3.1.3.69]
K05979	0.000213902	0.000211876	0.000186814	0.000116873	2-phosphosulfolactate phosphatase [EC:3.1.3.71]
K05982	1.3701E-06	1.23411E-06	2.81465E-06	4.11375E-05	deoxyribonuclease V [EC:3.1.21.7]
K05984	3.88862E-07	5.49128E-07	7.26046E-07	2.13316E-05	excinuclease Cho [EC:3.1.25.-]
K05985	2.12542E-06	6.71003E-07	7.75628E-06	3.72021E-06	ribonuclease M5 [EC:3.1.26.8]
K05986	1.06379E-06	1.21727E-06	1.75105E-06	5.14341E-05	nuclease S1 [EC:3.1.30.1]
K05988	2.16276E-07	1.32728E-08	3.40991E-07	1.64458E-07	dextranase [EC:3.2.1.11]
K05989	7.11693E-07	1.87963E-06	2.56275E-06	5.43147E-07	alpha-L-rhamnosidase [EC:3.2.1.40]
K05991	1.51535E-06	8.10371E-07	1.65042E-05	3.53999E-06	endoglycosylceramidase [EC:3.2.1.123]
K05994	4.42822E-07	1.80085E-07	1.73508E-06	1.38727E-05	bacterial leucyl aminopeptidase [EC:3.4.11.10]
K05995	5.57695E-07	2.01137E-06	4.50338E-06	7.80769E-06	dipeptidase E [EC:3.4.13.21]
K05996	0	0	1.00642E-06	9.52204E-08	carboxypeptidase T [EC:3.4.17.18]
K05997	2.87023E-07	7.74127E-07	3.94767E-07	6.77991E-06	Fe-S cluster assembly protein SufA
K05998	1.22674E-07	1.33928E-07	3.2835E-07	3.67522E-08	pseudomonalisin [EC:3.4.21.100]
K05999	1.30032E-07	8.73769E-08	4.01089E-07	3.06127E-07	xanthomonalisin [EC:3.4.21.101]

K06001	0.00021744	0.000221396	0.000220793	0.000183015	tryptophan synthase beta chain [EC:4.2.1.20]
K06006	1.72418E-07	6.15511E-07	2.29994E-07	1.50759E-05	periplasmic protein CpxP
K06011	0	0	0	2.28005E-10	botulinum neurotoxin [EC:3.4.24.69]
K06012	8.62278E-07	7.24816E-07	5.97037E-07	2.53664E-08	spore protease [EC:3.4.24.78]
K06013	0.000215328	0.000215012	0.000204009	0.000149059	STE24 endopeptidase [EC:3.4.24.84]
K06015	0.000215595	0.000213857	0.000191461	0.000122499	N-acyl-D-amino-acid deacylase [EC:3.5.1.81]
K06016	0.000227537	0.000228692	0.000262142	0.000263872	beta-ureidopropionase / N-carbamoyl-L-amino-acid hydrolase [EC:3.5.1.6 3.5.1.87]
K06019	1.67236E-07	2.31737E-07	1.26395E-06	3.73485E-07	pyrophosphatase PpaX [EC:3.6.1.1]
K06020	0.000224615	0.00022603	0.000224105	0.000224649	sulfate-transporting ATPase [EC:3.6.3.25]
K06023	0.000218707	0.000216793	0.000212766	0.000203517	HPr kinase/phosphorylase [EC:2.7.11.- 2.7.4.-]
K06024	0.000220872	0.000218768	0.000218747	0.000215842	segregation and condensation protein B
K06027	0	3.86545E-08	1.13318E-08	0	vesicle-fusing ATPase [EC:3.6.4.6]
K06033	5.79178E-08	4.2926E-08	8.953E-08	5.65278E-09	arylmalonate decarboxylase [EC:4.1.1.76]
K06034	1.69344E-08	4.17396E-08	4.74434E-07	2.64433E-08	sulfopyruvate decarboxylase subunit alpha [EC:4.1.1.79]
K06039	1.02214E-07	3.04294E-07	3.43928E-07	5.74895E-06	uncharacterized protein involved in oxidation of intracellular sulfur
K06041	0.000220064	0.000219579	0.000215521	0.000224671	arabinose-5-phosphate isomerase [EC:5.3.1.13]
K06042	0.000217505	0.000216306	0.000213274	0.000153281	precorrin-8X/cobalt-precorrin-8 methylmutase [EC:5.4.99.61 5.4.99.60]
K06044	0.000218223	0.000216321	0.000195516	0.000167762	(1->4)-alpha-D-glucan 1-alpha-D-glucosylmutase [EC:5.4.99.15]
K06045	2.5412E-06	3.66227E-07	8.44769E-07	2.07958E-07	squalene-hopene/tetraprenyl-beta-curcumene cyclase [EC:5.4.99.17 4.2.1.129]
K06046	3.1635E-09	0	1.91986E-08	1.58716E-07	long-chain-fatty-acid---luciferin-component ligase [EC:6.2.1.19]
K06048	0.000435263	0.000434475	0.000414982	0.000315106	glutamate---cysteine ligase / carboxylate-amine ligase [EC:6.3.2.2 6.3.-.-]
K06049	1.05222E-06	1.73179E-07	3.58578E-07	1.63925E-07	magnesium chelatase accessory protein
K06073	1.9308E-06	8.69429E-07	6.38E-06	1.39096E-05	vitamin B12 transport system permease protein
K06074	1.39398E-06	3.4449E-06	1.95767E-06	1.93842E-05	vitamin B12 transport system ATP-binding protein [EC:3.6.3.33]
K06075	0.000432997	0.000431978	0.000406459	0.000371939	MarR family transcriptional regulator, transcriptional regulator for hemolysin
K06076	7.35596E-06	3.89769E-06	1.2317E-05	9.46399E-05	long-chain fatty acid transport protein
K06077	0.000215407	0.000214476	0.000191201	0.000189669	outer membrane lipoprotein SlyB
K06078	1.09978E-06	1.4945E-06	1.76576E-06	6.3623E-05	murein lipoprotein
K06079	2.67576E-07	7.12053E-07	7.52657E-07	3.84822E-06	copper homeostasis protein (lipoprotein)
K06080	9.82254E-08	3.01037E-07	2.6219E-07	5.35113E-06	RcsF protein
K06113	3.00469E-06	2.06303E-06	6.99242E-06	6.05713E-07	arabinan endo-1,5-alpha-L-arabinosidase [EC:3.2.1.99]
K06118	4.32148E-07	5.26297E-07	1.87427E-06	7.23128E-07	UDP-sulfoquinovose synthase [EC:3.13.1.1]
K06120	7.96147E-08	2.19769E-07	6.8135E-07	9.18177E-07	glycerol dehydratase large subunit [EC:4.2.1.30]
K06121	5.89535E-08	1.35127E-09	2.01776E-07	9.04157E-07	glycerol dehydratase medium subunit [EC:4.2.1.30]
K06122	4.84954E-08	1.06472E-07	6.69191E-09	2.29125E-07	glycerol dehydratase small subunit [EC:4.2.1.30]
K06125	0	0	6.57495E-08	0	4-hydroxybenzoate polyprenyltransferase [EC:2.5.1.39]
K06131	0.000444384	0.000446008	0.000467618	0.000529589	cardiolipin synthase A/B [EC:2.7.8.-]
K06132	7.65985E-06	1.02689E-05	3.79689E-05	0.00017809	cardiolipin synthase C [EC:2.7.8.-]
K06133	0.000649448	0.000645713	0.000595905	0.000450141	4-phosphopantetheinyl transferase [EC:2.7.8.-]
K06134	0.000217918	0.00021668	0.000207145	0.00020042	3-demethoxyubiquinol 3-hydroxylase [EC:1.14.99.60]
K06135	3.37927E-08	1.0119E-07	4.48238E-08	1.3095E-07	pyrroloquinoline quinone biosynthesis protein A
K06136	1.29473E-06	8.70972E-07	1.84065E-06	8.09474E-07	pyrroloquinoline quinone biosynthesis protein B
K06137	2.56558E-06	1.10392E-06	2.36678E-06	9.65136E-07	pyrroloquinoline-quinone synthase [EC:1.3.3.11]
K06138	2.26876E-06	9.18692E-07	1.85835E-06	8.03426E-07	pyrroloquinoline quinone biosynthesis protein D
K06139	1.30211E-06	9.03806E-07	2.1641E-06	8.32105E-07	pyrroloquinoline quinone biosynthesis protein E
K06140	0.000216875	0.000216962	0.000210183	0.000214539	regulator of nucleoside diphosphate kinase
K06141	1.05122E-07	3.28879E-07	2.05502E-07	1.59409E-06	MFS transporter, TsgA protein
K06142	0.000216428	0.000219549	0.000213601	0.000166214	outer membrane protein
K06143	0.000216191	0.000216547	0.000207349	0.000204638	inner membrane protein
K06144	9.82021E-08	3.14557E-07	1.39031E-07	9.20888E-06	universal stress protein B
K06145	0.000220892	0.000223107	0.000241738	0.000269115	LacI family transcriptional regulator, gluconate utilization system Gnt-I transcriptional repressor
K06146	7.20112E-08	2.86837E-07	5.77323E-08	5.44517E-06	LacI family transcriptional regulator, gluconate utilization system Gnt-II transcriptional activator
K06147	0.001813543	0.001803349	0.00194109	0.002040143	ATP-binding cassette, subfamily B, bacterial
K06148	9.66393E-06	1.23683E-05	5.5662E-05	8.36388E-05	ATP-binding cassette, subfamily C, bacterial
K06149	0.000216515	0.000218787	0.000209911	0.000170488	universal stress protein A
K06151	7.58375E-07	9.46974E-07	1.68774E-06	2.23516E-05	gluconate 2-dehydrogenase alpha chain [EC:1.1.99.3]
K06152	8.48714E-07	7.8816E-07	1.4541E-06	2.22856E-05	gluconate 2-dehydrogenase gamma chain [EC:1.1.99.3]
K06153	0.000225311	0.000225349	0.000235794	0.000242311	undecaprenyl-diphosphatase [EC:3.6.1.27]
K06155	0.000215192	0.000212454	0.000192583	0.000115486	Gnt-I system high-affinity gluconate transporter
K06156	4.44281E-07	1.16995E-06	1.19659E-06	7.13342E-06	Gnt-I system low-affinity gluconate transporter
K06157	3.3262E-07	3.28706E-07	2.57566E-07	9.89032E-07	Gnt-II system L-idonate transporter
K06158	0.000912508	0.000919432	0.001050078	0.001212706	ATP-binding cassette, subfamily F, member 3
K06159	2.17129E-06	4.1043E-07	2.87794E-06	7.81445E-06	multidrug/microcin transport system ATP-binding/permease protein
K06160	0.000217653	0.000215622	0.000208358	0.000154029	putative pyoverdin transport system ATP-binding/permease protein
K06161	1.85357E-09	1.54877E-08	4.96297E-07	2.442E-09	putative syringomycin transport system ATP-binding/permease protein
K06162	2.65754E-06	2.0951E-06	4.37906E-06	6.44081E-05	alpha-D-ribose 1-methylphosphonate 5-triphosphate diphosphatase [EC:3.6.1.63]
K06163	1.16813E-06	5.46659E-07	1.76531E-06	1.91116E-06	alpha-D-ribose 1-methylphosphonate 5-phosphate C-P lyase [EC:4.7.1.1]
K06164	1.16895E-06	5.86962E-07	1.7294E-06	1.41536E-06	alpha-D-ribose 1-methylphosphonate 5-triphosphate synthase subunit PhnI [EC:2.7.8.37]
K06165	1.1681E-06	5.4657E-07	1.7294E-06	1.02092E-06	alpha-D-ribose 1-methylphosphonate 5-triphosphate synthase subunit PhnH [EC:2.7.8.37]
K06166	1.16895E-06	5.4657E-07	1.7294E-06	9.13465E-07	alpha-D-ribose 1-methylphosphonate 5-triphosphate synthase subunit PhnG [EC:2.7.8.37]
K06167	2.84116E-06	3.22999E-06	1.10657E-05	4.60976E-06	phosphoribosyl 1,2-cyclic phosphate phosphodiesterase [EC:3.1.4.55]
K06168	0.000225361	0.000225354	0.000234654	0.0002221	tRNA-2-methylthio-N6-dimethylallyladenosine synthase [EC:2.8.4.3]
K06169	1.397E-06	7.91219E-07	2.42334E-06	1.56153E-05	tRNA-(ms[2]io[6]A)-hydroxylase [EC:1.-.-.-]
K06173	0.000223699	0.000223925	0.000228601	0.000226352	tRNA pseudouridine38-40 synthase [EC:5.4.99.12]
K06174	2.07103E-06	4.40902E-06	1.49498E-05	6.41766E-06	ATP-binding cassette, sub-family E, member 1
K06175	3.99691E-06	5.49529E-06	2.16761E-05	0.000146887	tRNA pseudouridine65 synthase [EC:5.4.99.26]
K06176	2.45335E-06	2.00023E-06	4.136E-06	6.91227E-05	tRNA pseudouridine13 synthase [EC:5.4.99.27]
K06177	0.000225481	0.000226996	0.00023898	0.000349926	tRNA pseudouridine32 synthase / 23S rRNA pseudouridine746 synthase [EC:5.4.99.28 5.4.99.29]
K06178	0.000442698	0.000441204	0.000442028	0.000475264	23S rRNA pseudouridine2605 synthase [EC:5.4.99.22]
K06179	0.000223997	0.000220435	0.000227871	0.000281662	23S rRNA pseudouridine955/2504/2580 synthase [EC:5.4.99.24]
K06180	0.000452569	0.000452769	0.000490712	0.000573815	23S rRNA pseudouridine1911/1915/1917 synthase [EC:5.4.99.23]
K06181	0.000220411	0.000218045	0.000208948	0.000224784	23S rRNA pseudouridine2457 synthase [EC:5.4.99.20]
K06182	0.000219996	0.000221844	0.000226545	0.000306898	23S rRNA pseudouridine2604 synthase [EC:5.4.99.21]
K06183	0.000222972	0.000219597	0.000221065	0.000247866	16S rRNA pseudouridine516 synthase [EC:5.4.99.19]
K06186	0.00021761	0.000216916	0.000207148	0.000217477	outer membrane protein assembly factor BamE
K06187	0.000222885	0.000223885	0.000226135	0.000238719	recombination protein RecR
K06188	5.09589E-06	8.6797E-06	2.00651E-05	9.6554E-05	aquaporin Z

K06189	0.000439488	0.000441505	0.00041703	0.000352711	magnesium and cobalt transporter
K06190	0.000218284	0.000215455	0.000205104	0.00016419	intracellular septation protein
K06191	1.36464E-06	3.91033E-06	4.11413E-06	1.04997E-05	glutaredoxin-like protein NrdH
K06192	0.000428206	0.000424883	0.000375192	0.000248756	paraquat-inducible protein B
K06193	0.000219174	0.000217869	0.000209393	0.000225834	protein PhnA
K06194	0.000434598	0.000432844	0.000405016	0.000402709	lipoprotein NlpD
K06195	0.000218161	0.000217859	0.000207075	0.0002175	ApaG protein
K06196	1.05416E-05	1.64701E-05	5.94574E-05	0.000163237	cytochrome c-type biogenesis protein
K06197	1.75769E-07	1.02548E-06	1.84725E-07	2.54978E-06	cation transport regulator
K06198	1.28081E-06	9.79324E-08	4.02188E-06	3.70665E-06	competence protein CoiA
K06199	0.000225354	0.000224689	0.000238263	0.000225739	fluoride exporter
K06200	0.000221014	0.000219383	0.000207782	0.000183494	carbon starvation protein
K06201	2.53226E-06	2.62287E-06	1.3127E-05	7.22478E-05	copper homeostasis protein
K06202	0.000215422	0.000215608	0.000204508	0.000165574	CyaY protein
K06203	1.5729E-06	9.01795E-07	2.03226E-06	1.7814E-05	CysZ protein
K06204	0.000221975	0.000220369	0.000220919	0.000226408	DnaK suppressor protein
K06205	2.7056E-07	7.7038E-07	1.85696E-06	2.26338E-05	MioC protein
K06206	1.86823E-06	1.31162E-06	5.20586E-06	1.84141E-05	sugar fermentation stimulation protein A
K06207	0.000225102	0.000224659	0.000234955	0.00027915	GTP-binding protein
K06208	2.22212E-07	2.47848E-07	1.0956E-06	1.2947E-07	chorismate mutase [EC:5.4.99.5]
K06209	1.68403E-06	7.4592E-07	6.60287E-06	9.25852E-08	chorismate mutase [EC:5.4.99.5]
K06211	1.72584E-06	6.2966E-07	1.71518E-06	3.2661E-05	HTH-type transcriptional regulator, transcriptional repressor of NAD biosynthesis genes [EC:2.7.7.1 2.7.1.22]
K06212	1.77453E-06	6.71581E-07	6.45803E-06	2.20708E-05	formate transporter
K06213	9.3136E-06	1.26573E-05	3.2329E-05	0.000169828	magnesium transporter
K06214	1.66693E-06	2.43027E-07	7.16808E-07	7.58533E-06	curli production assembly/transport component CsgG
K06215	2.95554E-06	4.66512E-06	1.3612E-05	5.09547E-06	pyridoxal 5-phosphate synthase pdxS subunit [EC:4.3.3.6]
K06216	9.67297E-07	0	2.98273E-06	5.08911E-07	putative ribose uptake protein
K06217	0.000225027	0.000224701	0.000228402	0.000229181	phosphate starvation-inducible protein PhoH and related proteins
K06218	0.000214322	0.000212986	0.000191836	0.000120033	mRNA interferase RelE/StbE
K06219	1.3931E-06	7.60291E-07	2.23205E-06	1.4315E-05	S-adenosylmethionine-dependent methyltransferase
K06221	0.000225234	0.000225858	0.000238281	0.00020629	2,5-diketo-D-gluconate reductase A [EC:1.1.1.346]
K06222	0.00022502	0.000225253	0.000228221	0.000337497	2,5-diketo-D-gluconate reductase B [EC:1.1.1.346]
K06223	4.52719E-06	5.44465E-06	2.21892E-05	8.5638E-05	DNA adenine methylase [EC:2.1.1.72]
K06281	8.85487E-07	7.08514E-07	1.76352E-06	5.84364E-06	hydrogenase large subunit [EC:1.12.99.6]
K06282	8.87004E-07	7.06585E-07	1.72252E-06	5.78731E-06	hydrogenase small subunit [EC:1.12.99.6]
K06283	8.62278E-07	7.08441E-07	1.04798E-06	2.34647E-08	putative DeoR family transcriptional regulator, stage III sporulation protein D
K06284	1.82879E-06	1.46911E-06	2.65926E-06	1.39656E-07	transcriptional pleiotropic regulator of transition state genes
K06285	1.14351E-07	2.06813E-07	5.80021E-07	1.29419E-08	transcription attenuation protein (tryptophan RNA-binding attenuator protein)
K06286	1.30797E-06	3.67588E-07	6.78337E-06	3.63511E-06	septation ring formation regulator
K06287	0.00044061	0.000441538	0.000431692	0.000440207	septum formation protein
K06288	5.54378E-08	2.46859E-07	7.71644E-08	0	spore germination protein AA
K06289	0	5.12659E-10	1.20243E-08	0	spore germination protein AB
K06291	5.54378E-08	9.5208E-10	2.9902E-09	0	spore germination protein BA
K06294	5.54378E-08	2.06813E-07	4.47816E-07	0	spore germination protein D
K06295	9.17716E-07	1.1726E-06	1.22911E-06	7.43972E-08	spore germination protein KA
K06296	1.18375E-07	3.26902E-07	2.29609E-07	9.5087E-09	spore germination protein KB
K06297	1.73813E-07	8.61355E-08	5.44567E-07	9.5087E-09	spore germination protein KC
K06298	4.64342E-07	3.99145E-07	4.13772E-07	5.70522E-09	germination protein M
K06299	1.10876E-07	4.12674E-07	7.00719E-08	0	spore germination protein PA
K06300	5.60397E-08	2.07583E-07	3.40631E-08	0	spore germination protein PB
K06301	5.54378E-08	2.06813E-07	5.73987E-08	0	spore germination protein PC
K06302	5.54378E-08	2.06813E-07	5.73987E-08	0	spore germination protein PD
K06303	0	2.06813E-07	5.73987E-08	0	spore germination protein PE
K06304	5.54378E-08	4.12674E-07	1.03982E-07	0	spore germination protein PF
K06305	5.54378E-08	2.06813E-07	5.40647E-07	1.90174E-09	spore germination protein Q
K06306	1.1141E-06	4.48633E-07	2.04635E-06	9.88537E-08	spore germination protein
K06307	0	2.05861E-07	2.61291E-08	0	spore germination protein
K06308	0	2.08278E-07	1.41197E-07	0	spore germination protein
K06309	0	0	1.13318E-08	0	spore germination protein
K06310	2.20016E-07	8.15488E-07	6.50419E-07	1.2862E-08	spore germination protein
K06311	5.54378E-08	3.96065E-08	5.51399E-08	0	spore germination protein
K06312	0	9.5208E-10	2.93681E-08	0	spore germination protein
K06313	5.54378E-08	2.45467E-07	2.34729E-07	0	spore germination protein
K06314	1.10876E-07	4.13626E-07	8.95786E-07	0	prespore-specific regulator
K06315	0	0	3.64773E-08	0	transcriptional regulator of the spore photoproduct lyase operon
K06317	1.42237E-07	2.58456E-07	4.55414E-07	2.9957E-09	inhibitor of the pro-sigma K processing machinery
K06318	0	2.05861E-07	4.31831E-07	0	forespore regulator of the sigma-K checkpoint
K06320	1.31651E-06	3.40547E-07	9.13483E-08	2.03835E-08	spore maturation protein CgeB
K06322	4.22178E-07	3.06602E-09	2.52287E-07	0	spore maturation protein CgeD
K06324	1.85096E-07	2.93883E-07	9.1218E-07	3.44897E-06	spore coat protein A, manganese oxidase [EC:1.16.3.3]
K06325	0	5.12659E-10	2.54138E-08	0	spore coat protein B
K06327	5.54378E-08	2.05861E-07	7.91389E-08	0	spore coat protein D
K06328	5.54378E-08	2.06813E-07	5.405E-07	1.90174E-09	spore coat protein E
K06329	1.66314E-07	1.02051E-08	1.13523E-08	0	spore coat protein F
K06330	5.54378E-08	0	8.19601E-08	1.21049E-07	spore coat protein H
K06331	0	2.18849E-07	4.41555E-08	1.09396E-09	spore coat protein I
K06332	5.54378E-08	2.06813E-07	7.91447E-08	0	spore coat protein JA
K06333	6.04913E-07	4.80044E-07	7.55753E-07	2.53664E-08	spore coat protein JB
K06334	1.04525E-06	9.40719E-07	1.60064E-06	1.09529E-07	spore coat protein JC
K06335	0	0	2.78209E-08	0	spore coat protein M
K06336	0	8.78843E-10	6.4867E-08	0	spore coat-associated protein N
K06338	5.28731E-08	4.64451E-07	5.46814E-07	9.93107E-08	spore coat protein SA
K06341	0	0	7.21284E-09	0	spore coat protein W
K06342	0	0	1.06503E-07	1.90174E-09	spore coat protein X
K06343	5.54378E-08	0	1.1092E-08	0	spore coat protein Y
K06344	5.54378E-08	0	2.15062E-08	0	spore coat protein Z
K06345	0	2.05861E-07	5.20828E-08	0	spore cortex protein

K06346	2.83445E-06	4.73016E-06	1.33881E-05	7.7255E-06	spoIIIJ-associated protein
K06347	9.92093E-07	9.5208E-10	3.15849E-06	3.03626E-08	kinase-associated protein B
K06348	0	0	4.9827E-07	1.90174E-09	sporulation inhibitor KapD
K06349	1.46319E-07	2.06813E-07	6.09645E-07	1.90174E-09	KinB signaling pathway activation protein
K06350	0.000432678	0.00042715	0.000387253	0.000264816	antagonist of KipI
K06351	0.000435036	0.000431511	0.000402913	0.000280984	inhibitor of KinA
K06361	0	0	8.97061E-09	0	response regulator aspartate phosphatase C [EC:3.1.-.-]
K06370	7.54806E-07	4.75814E-07	1.12118E-06	1.79115E-08	morphogenetic protein associated with SpoVID
K06371	0	2.063E-07	5.98928E-08	0	developmental checkpoint coupling sporulation initiation to replication initiation
K06372	0	0	4.48531E-09	0	antagonist of SinR
K06373	0.000216134	0.00021648	0.000207018	0.000149756	spore maturation protein A
K06374	0.000216134	0.00021648	0.000207018	0.000149756	spore maturation protein B
K06375	1.46319E-07	2.06813E-07	5.7146E-07	1.90174E-09	stage 0 sporulation protein B (sporulation initiation phosphotransferase) [EC:2.7.-.-]
K06376	0	9.5208E-10	4.38215E-07	0	stage 0 sporulation regulatory protein
K06377	9.08815E-08	2.34409E-07	8.34824E-07	6.84801E-06	sporulation-control protein
K06378	8.67243E-07	7.60122E-07	1.19083E-06	9.74115E-07	stage II sporulation protein AA (anti-sigma F factor antagonist)
K06379	8.62278E-07	7.38666E-07	7.7472E-07	2.53664E-08	stage II sporulation protein AB (anti-sigma F factor) [EC:2.7.11.1]
K06380	0	2.06813E-07	6.88771E-08	0	stage II sporulation protein B
K06381	1.95663E-06	2.19135E-06	4.27022E-06	1.93963E-06	stage II sporulation protein D
K06382	8.62278E-07	7.24816E-07	1.14066E-06	2.53664E-08	stage II sporulation protein E [EC:3.1.3.16]
K06383	6.04913E-07	4.40522E-07	1.13206E-06	2.40945E-08	stage II sporulation protein GA (sporulation sigma-E factor processing peptidase) [EC:3.4.23.-]
K06384	4.64197E-07	3.90993E-07	4.29934E-07	6.79918E-09	stage II sporulation protein M
K06385	8.62278E-07	7.6347E-07	1.26152E-06	2.53664E-08	stage II sporulation protein P
K06386	5.54378E-08	2.23188E-07	1.052E-06	2.91472E-07	stage II sporulation protein Q
K06387	8.39937E-07	7.08441E-07	1.1355E-06	1.68086E-08	stage II sporulation protein R
K06390	6.1085E-07	5.07234E-07	1.12151E-06	2.7902E-08	stage III sporulation protein AA
K06391	5.54378E-08	2.62529E-07	4.16112E-07	2.22452E-09	stage III sporulation protein AB
K06392	8.62278E-07	7.08441E-07	6.82037E-07	2.34647E-08	stage III sporulation protein AC
K06393	6.04913E-07	4.40522E-07	6.63019E-07	2.21928E-08	stage III sporulation protein AD
K06394	6.04913E-07	5.92366E-07	4.87917E-07	2.34647E-08	stage III sporulation protein AE
K06395	5.54378E-08	3.01775E-07	8.27947E-08	1.09396E-09	stage III sporulation protein AF
K06396	7.21563E-07	6.20841E-07	8.5214E-07	7.29987E-09	stage III sporulation protein AG
K06397	7.43903E-07	7.22594E-07	1.14066E-06	1.77594E-08	stage III sporulation protein AH
K06398	8.62278E-07	7.24816E-07	1.50661E-06	2.53664E-08	stage IV sporulation protein A
K06399	8.62278E-07	7.24816E-07	1.89005E-06	3.97704E-08	stage IV sporulation protein B [EC:3.4.21.116]
K06400	0.000219204	0.000214875	0.000199842	0.000124247	site-specific DNA recombinase
K06401	7.59174E-08	6.4259E-07	1.02192E-06	7.51115E-08	stage IV sporulation protein FA
K06402	3.23006E-07	3.68426E-07	2.08398E-06	5.32999E-06	stage IV sporulation protein FB [EC:3.4.24.-]
K06403	1.42237E-07	3.20672E-07	7.65142E-08	2.40243E-09	stage V sporulation protein AA
K06404	1.42237E-07	3.20672E-07	7.65142E-08	2.40243E-09	stage V sporulation protein AB
K06405	9.80653E-07	9.46399E-07	8.2736E-07	3.29733E-08	stage V sporulation protein AC
K06406	8.62278E-07	9.44178E-07	8.13471E-07	2.53664E-08	stage V sporulation protein AD
K06407	1.18424E-06	1.07758E-06	1.45708E-06	2.91699E-08	stage V sporulation protein AE
K06408	3.77398E-07	7.84889E-07	1.31187E-06	9.20161E-09	stage V sporulation protein AF
K06409	1.56943E-06	1.28024E-06	5.84691E-06	3.74943E-06	stage V sporulation protein B
K06410	8.62302E-07	6.01655E-07	1.17687E-06	2.30006E-08	dipicolinate synthase subunit A
K06411	8.62278E-07	6.06707E-07	1.04428E-06	2.10988E-08	dipicolinate synthase subunit B
K06412	2.10028E-06	7.2608E-07	4.64255E-06	1.25889E-07	stage V sporulation protein G
K06413	3.27015E-06	1.16034E-06	5.43592E-06	1.42995E-06	stage V sporulation protein K
K06415	2.66335E-06	4.14127E-06	1.66366E-05	5.20775E-05	stage V sporulation protein R
K06416	1.09747E-07	2.9711E-07	5.95568E-07	2.14924E-08	stage V sporulation protein S
K06417	5.54378E-08	2.06813E-07	4.34821E-07	0	stage VI sporulation protein D
K06418	1.10876E-07	4.54624E-07	9.81109E-07	7.60696E-09	small acid-soluble spore protein A (major alpha-type SASP)
K06419	1.66314E-07	4.16922E-07	1.12409E-06	1.14104E-08	small acid-soluble spore protein B (major beta-type SASP)
K06420	0	2.07252E-07	6.60387E-07	3.80348E-09	small acid-soluble spore protein C (minor alpha/beta-type SASP)
K06421	1.66314E-07	4.30423E-07	1.28594E-06	1.44061E-08	small acid-soluble spore protein D (minor alpha/beta-type SASP)
K06422	5.54378E-08	2.06813E-07	4.54273E-07	0	small acid-soluble spore protein E (minor gamma-type SASP)
K06423	5.54378E-08	3.96065E-08	4.35143E-07	0	small acid-soluble spore protein F (minor alpha/beta-type SASP)
K06425	4.32836E-07	3.49983E-07	3.66284E-07	3.80348E-09	small acid-soluble spore protein H (minor)
K06426	5.54378E-08	2.45467E-07	5.37919E-07	1.90174E-09	small acid-soluble spore protein I (minor)
K06428	0	2.06813E-07	6.01262E-08	0	small acid-soluble spore protein K (minor)
K06429	5.54378E-08	2.06374E-07	1.87022E-07	0	small acid-soluble spore protein L (minor)
K06431	0	2.06813E-07	6.01262E-08	0	small acid-soluble spore protein N (minor)
K06432	5.54378E-08	2.06813E-07	5.0659E-08	0	small acid-soluble spore protein O (minor)
K06433	5.54378E-08	2.06813E-07	6.88771E-08	0	small acid-soluble spore protein P (minor)
K06434	5.54378E-08	2.58456E-07	8.10865E-08	0	small acid-soluble spore protein (thioredoxin-like protein)
K06436	5.54378E-08	2.58456E-07	6.26913E-07	4.89744E-09	spore coat assemly protein
K06438	8.62278E-07	7.08441E-07	1.04798E-06	2.34647E-08	similar to stage IV sporulation protein
K06439	1.10876E-07	1.29883E-08	4.64434E-08	0	similar to spore coat protein
K06440	5.54378E-08	2.04102E-08	6.73271E-08	0	similar to spore coat protein
K06441	0	4.59904E-09	2.18626E-07	6.10772E-09	ferredoxin hydrogenase gamma subunit [EC:1.12.7.2]
K06442	5.7839E-06	8.2993E-06	3.16739E-05	4.32025E-05	23S rRNA (cytidine1920-2-O)/16S rRNA (cytidine1409-2-O)-methyltransferase [EC:2.1.1.226 2.1.1.227]
K06443	4.85824E-07	1.3156E-06	9.61204E-07	1.83803E-06	lycopene beta-cyclase [EC:5.5.1.19]
K06445	0.000215402	0.000214221	0.00019074	0.000202897	acyl-CoA dehydrogenase [EC:1.3.99.-]
K06446	0.001093954	0.001110099	0.00112676	0.001096791	acyl-CoA dehydrogenase [EC:1.3.99.-]
K06447	2.72077E-06	1.88625E-06	8.48017E-06	3.3439E-05	succinylglutamic semialdehyde dehydrogenase [EC:1.2.1.71]
K06518	0.000219996	0.000220205	0.000208586	0.00018068	holin-like protein
K06580	0	3.98157E-08	3.48857E-08	0	ammonium transporter Rh
K06595	5.06385E-07	1.90565E-06	2.61553E-06	1.00748E-05	heam-based aerotactic trancducer
K06596	0.000436271	0.000432535	0.000400281	0.000375642	chemosensory pili system protein ChpA (sensor histidine kinase/response regulator)
K06597	2.35513E-06	1.67766E-06	3.46474E-06	5.15854E-05	chemosensory pili system protein ChpB (putative protein-glutamate methylesterase)
K06598	1.21649E-06	1.67277E-06	2.85327E-06	5.1609E-05	chemosensory pili system protein ChpC
K06599	0.000214349	0.000212034	0.000188396	0.000132513	AraC family transcriptional regulator, chemosensory pili system protein ChpD
K06600	0	5.92629E-09	1.35076E-06	1.16015E-07	chemosensory pili system protein ChpE
K06601	2.57807E-06	2.18022E-07	4.63021E-07	1.68757E-07	flagellar protein FlbT
K06602	2.57324E-06	2.18022E-07	4.81948E-07	1.68757E-07	flagellar protein FlaF
K06603	8.84001E-07	9.06606E-07	2.59116E-06	1.65582E-05	flagellar protein FlaG

K06604	0	1.35781E-08	6.41787E-08	8.40539E-06	flagellar rod protein FlaI
K06605	0.000428712	0.000425377	0.000384095	0.000242534	myo-inositol catabolism protein IolH
K06606	0.000214371	0.000213301	0.000191696	0.000127565	2-keto-myo-inositol isomerase [EC:5.3.99.11]
K06607	4.69489E-06	9.43694E-06	2.0695E-05	8.62095E-05	myo-inositol catabolism protein IolS [EC:1.1.1.-]
K06608	1.28329E-07	3.96627E-08	7.86235E-08	6.1183E-09	DeoR family transcriptional regulator, myo-inositol catabolism operon repressor
K06609	3.70399E-06	4.74196E-06	1.82206E-05	6.24129E-06	MFS transporter, SP family, major inositol transporter
K06610	1.83391E-07	9.29855E-08	3.03476E-07	1.3182E-07	MFS transporter, SP family, inositol transporter
K06714	0.001081557	0.001074239	0.000997369	0.000735784	arginine utilization regulatory protein
K06718	1.11784E-07	1.11434E-06	8.20485E-07	1.01666E-05	L-2,4-diaminobutyric acid acetyltransferase [EC:2.3.1.178]
K06720	9.8772E-07	4.26254E-06	1.34852E-06	1.01244E-05	L-ectoine synthase [EC:4.2.1.108]
K06726	2.01141E-06	1.03096E-06	1.17222E-05	1.25672E-05	D-ribose pyranase [EC:5.4.99.62]
K06857	0.000437756	0.000442714	0.000407516	0.000269486	tungstate transport system ATP-binding protein [EC:3.6.3.55]
K06858	2.95658E-07	5.76148E-07	1.80746E-06	1.77541E-05	vitamin B12 transport system substrate-binding protein
K06859	1.44369E-08	1.59555E-07	2.86572E-06	1.4121E-07	glucose-6-phosphate isomerase, archaeal [EC:5.3.1.9]
K06860	0	5.96007E-07	2.61966E-06	1.83563E-06	putative heme uptake system protein
K06861	0.000224448	0.000224184	0.000240378	0.000268223	lipopolysaccharide export system ATP-binding protein [EC:3.6.3.-]
K06862	9.16819E-09	0	0	0	energy-converting hydrogenase B subunit Q
K06864	2.57246E-06	4.97326E-07	3.93528E-06	3.42099E-06	pyridinium-3,5-biscarboxylic acid mononucleotide sulfurtransferase [EC:4.4.1.37]
K06865	2.98321E-07	6.64376E-07	2.98826E-06	6.93882E-06	ATPase
K06866	1.96647E-07	6.83099E-07	7.21587E-07	2.64997E-05	autonomous glycyl radical cofactor
K06867	0.000866129	0.000867994	0.000810415	0.000713508	uncharacterized protein
K06870	0	0	1.54857E-08	0	uncharacterized protein
K06871	4.78367E-06	4.41523E-06	1.92979E-05	3.5108E-05	uncharacterized protein
K06872	0.000220658	0.000219095	0.000215111	0.000249327	uncharacterized protein
K06873	0	0	0	2.67905E-09	uncharacterized protein
K06876	2.06542E-06	4.24323E-07	4.02005E-06	2.20709E-05	deoxyribodipyrimidine photolyase-related protein
K06877	4.12435E-06	6.37288E-06	2.00072E-05	6.51059E-05	DEAD/DEAH box helicase domain-containing protein
K06878	4.4112E-06	4.65348E-06	2.97827E-05	5.1945E-05	tRNA-binding protein
K06879	0.000217667	0.00021717	0.000206655	0.000217331	7-cyano-7-deazaguanine reductase [EC:1.7.1.13]
K06880	1.3071E-06	1.69989E-07	1.27799E-06	1.32346E-07	erythromycin esterase [EC:3.1.1.-]
K06881	3.93814E-06	6.14262E-06	2.73951E-05	4.61956E-05	bifunctional oligoribonuclease and PAP phosphatase NrnA [EC:3.1.3.7 3.1.13.3]
K06882	0	0	9.54637E-09	0	uncharacterized protein
K06883	2.24422E-07	3.61851E-07	6.47369E-07	8.2949E-08	uncharacterized protein
K06884	3.28553E-06	1.3174E-06	5.09867E-06	6.75717E-05	uncharacterized protein
K06885	2.28671E-06	2.45354E-06	1.05427E-05	1.63377E-05	uncharacterized protein
K06886	0.00022041	0.000221061	0.00022156	0.000214425	hemoglobin
K06887	1.30184E-06	3.00956E-06	1.38827E-05	3.71002E-05	uncharacterized protein
K06888	1.44331E-06	1.48954E-06	7.07336E-06	2.04318E-06	uncharacterized protein
K06889	0.000443889	0.000449757	0.00045085	0.00045727	uncharacterized protein
K06890	3.41501E-06	1.04996E-06	9.71783E-06	8.49541E-06	uncharacterized protein
K06891	0.000220917	0.000222337	0.000214505	0.000221567	ATP-dependent Clp protease adaptor protein ClpS
K06893	4.0295E-07	1.17689E-06	4.61178E-06	1.29806E-06	uncharacterized protein
K06894	0.0002175	0.00021789	0.000207949	0.000207549	uncharacterized protein
K06895	0.00043589	0.000435524	0.000406166	0.000386517	L-lysine exporter family protein LysE/ArgO
K06896	1.90766E-07	2.35382E-07	3.61399E-06	3.61656E-06	maltose 6-phosphate phosphatase [EC:3.1.3.90]
K06897	1.81247E-06	1.37519E-06	5.55156E-06	2.13274E-05	7,8-dihydropterin-6-yl-methyl-4-(beta-D-ribofuranosyl)aminobenzene 5-phosphate synthase [EC:2.5.1.105]
K06898	2.57544E-06	4.81005E-07	3.70511E-06	4.14818E-07	pyridinium-3,5-biscarboxylic acid mononucleotide synthase [EC:2.5.1.143]
K06899	2.59079E-07	7.78309E-07	1.63074E-06	2.23E-05	nucleoid-associated protein
K06900	7.70881E-07	1.01299E-06	3.40064E-06	8.86072E-06	uncharacterized protein
K06901	0.000437926	0.00043462	0.000412822	0.000396322	putative MFS transporter, AGZA family, xanthine/uracil permease
K06902	2.42844E-06	4.95331E-06	1.2508E-05	4.36828E-06	MFS transporter, UMF1 family
K06903	0.000217512	0.000217969	0.000208691	0.000200024	uncharacterized protein
K06904	0.000216604	0.000212908	0.000193075	0.000129194	uncharacterized protein
K06905	3.81773E-06	5.06413E-06	2.18748E-05	8.82689E-05	uncharacterized protein
K06906	2.9046E-06	5.09446E-06	2.09736E-05	8.64184E-05	uncharacterized protein
K06907	0.00021808	0.000218323	0.000209193	0.000203871	uncharacterized protein
K06908	3.57595E-06	5.34497E-06	2.14815E-05	8.59507E-05	uncharacterized protein
K06909	2.44807E-06	5.18607E-06	2.2793E-05	6.18399E-05	phage terminase large subunit
K06910	0.000432936	0.000430126	0.000400574	0.000332346	uncharacterized protein
K06911	0.000874321	0.000877685	0.000857499	0.000887859	uncharacterized protein
K06912	5.02516E-08	3.78688E-09	8.91245E-08	1.1693E-07	alpha-ketoglutarate-dependent 2,4-dichlorophenoxyacetate dioxygenase [EC:1.14.11.-]
K06915	0.000220781	0.000219132	0.000214605	0.00022637	uncharacterized protein
K06916	0.000220601	0.000220509	0.000209834	0.000228853	cell division protein ZapE
K06917	3.16721E-06	8.18951E-06	2.5762E-05	6.00276E-05	tRNA 2-selenouridine synthase [EC:2.9.1.-]
K06918	1.40266E-06	4.69501E-07	7.16414E-07	1.72711E-05	uncharacterized protein
K06919	0.000434711	0.000434208	0.000413195	0.000373113	putative DNA primase/helicase
K06920	0.000221729	0.000218483	0.000212749	0.000222935	7-cyano-7-deazaguanine synthase [EC:6.3.4.20]
K06921	9.54461E-07	1.31456E-06	3.62531E-06	1.5021E-07	uncharacterized protein
K06922	4.33011E-09	1.92286E-08	5.44714E-08	3.97516E-07	uncharacterized protein
K06923	0.000218321	0.000215946	0.000209313	0.00015074	uncharacterized protein
K06924	8.54206E-10	0	9.28178E-08	8.02722E-06	uncharacterized protein
K06925	0.000223884	0.000223893	0.00022958	0.000235838	tRNA threonylcarbamoyladenosine biosynthesis protein TsaE
K06926	1.31797E-06	1.79564E-06	1.44677E-05	5.95121E-06	uncharacterized protein
K06927	1.19151E-08	2.88628E-08	1.19081E-07	4.8658E-07	diphthine-ammonia ligase [EC:6.3.1.14]
K06928	3.2196E-07	1.72153E-07	2.65079E-07	5.21904E-09	nucleoside-triphosphatase [EC:3.6.1.15]
K06929	0.000218318	0.000219904	0.000205466	0.000161423	uncharacterized protein
K06930	0	1.99937E-08	0	0	uncharacterized protein
K06933	0	4.21761E-08	1.23678E-07	3.43565E-06	uncharacterized protein
K06934	5.9505E-07	4.98706E-07	3.82986E-06	7.93501E-06	uncharacterized protein
K06937	4.59224E-07	2.03232E-07	4.93521E-07	4.40083E-08	7,8-dihydro-6-hydroxymethylpterin dimethyltransferase [EC:2.1.1.-]
K06938	0.000435967	0.000432065	0.000411477	0.000335343	uncharacterized protein
K06939	0	3.86545E-08	0	0	uncharacterized protein
K06940	0.000220631	0.00022363	0.00023664	0.00028408	uncharacterized protein
K06941	0.000223688	0.000224366	0.000229817	0.000225052	23S rRNA (adenine2503-C2)-methyltransferase [EC:2.1.1.192]
K06942	0.000226813	0.000230745	0.000245421	0.00031085	ribosome-binding ATPase
K06943	0	6.10918E-09	0	0	nucleolar GTP-binding protein
K06944	0	3.27506E-09	2.20741E-07	6.78334E-09	uncharacterized protein

K06945	3.64955E-06	7.27152E-06	3.126E-05	0.000140805	uncharacterized protein
K06946	1.01506E-06	4.26163E-07	2.96607E-06	7.63672E-06	uncharacterized protein
K06947	0	0	0	3.57207E-09	polynucleotide 5-hydroxyl-kinase GRC3/NOL9 [EC:2.7.1.-]
K06948	1.31114E-06	3.44439E-07	8.97957E-06	3.69679E-06	30S ribosome assembly GTPase
K06949	0.000224741	0.000227734	0.00023055	0.000246385	ribosome biogenesis GTPase / thiamine phosphate phosphatase [EC:3.6.1.- 3.1.3.100]
K06950	0.000219215	0.000219136	0.00022093	0.00016806	uncharacterized protein
K06951	9.16819E-09	0	1.55894E-07	0	uncharacterized protein
K06952	1.98221E-06	3.46229E-07	3.17871E-06	8.58384E-07	uncharacterized protein
K06953	3.74043E-06	4.83002E-06	1.81905E-05	8.63377E-05	uncharacterized protein
K06954	3.58989E-06	4.17447E-06	2.07059E-05	0.000104854	uncharacterized protein
K06955	0.000218426	0.000218819	0.00022109	0.000185342	uncharacterized protein
K06956	3.32553E-06	4.92484E-06	6.95375E-06	4.79014E-05	uncharacterized protein
K06957	9.3669E-07	8.66213E-07	1.92455E-06	7.09553E-05	tRNA(Met) cytidine acetyltransferase [EC:2.3.1.193]
K06958	0.000221702	0.000222602	0.000227194	0.000225024	RNase adapter protein RapZ
K06959	0.000220889	0.000220081	0.000219586	0.0002282	protein Tex
K06960	2.95697E-06	4.72775E-06	1.3402E-05	7.70861E-06	uncharacterized protein
K06962	1.91512E-06	7.4592E-07	7.17351E-06	3.42634E-06	uncharacterized protein
K06963	0	0	1.63185E-07	4.15537E-09	tRNA acetyltransferase TAN1
K06966	0.000440701	0.000442922	0.00043928	0.000440346	pyrimidine/purine-5-nucleotide nucleosidase [EC:3.2.2.10 3.2.2.-]
K06967	2.21222E-06	8.63008E-07	7.91549E-06	1.63334E-05	tRNA (adenine22-N1)-methyltransferase [EC:2.1.1.217]
K06968	1.33825E-06	1.58949E-06	3.24689E-06	6.92172E-05	23S rRNA (cytidine2498-2-O)-methyltransferase [EC:2.1.1.186]
K06969	0.000221724	0.00022103	0.000222902	0.00023867	23S rRNA (cytosine1962-C5)-methyltransferase [EC:2.1.1.191]
K06970	6.19134E-07	1.94783E-06	1.69746E-06	1.71826E-05	23S rRNA (adenine1618-N6)-methyltransferase [EC:2.1.1.181]
K06971	2.77542E-08	2.58147E-07	3.39895E-07	4.00415E-06	uncharacterized protein
K06972	1.58022E-06	3.23743E-07	1.21586E-06	4.22761E-07	presequence protease [EC:3.4.24.-]
K06973	1.92465E-06	2.22684E-06	5.20036E-06	1.01093E-06	uncharacterized protein
K06974	1.83364E-08	8.85435E-08	2.18657E-07	3.158E-06	archaemetzincin [EC:3.4.-.-]
K06975	8.92273E-06	1.57228E-05	2.55274E-05	8.61358E-05	uncharacterized protein
K06976	1.66073E-06	3.64377E-06	7.65327E-06	2.15604E-06	uncharacterized protein
K06977	1.10221E-06	1.25392E-06	1.73453E-06	5.15913E-05	uncharacterized protein
K06978	5.74313E-06	1.09071E-05	5.1324E-05	0.000181304	uncharacterized protein
K06979	8.18937E-08	3.06834E-09	5.20515E-07	8.37808E-07	macrolide phosphotransferase
K06980	0.000220895	0.000221217	0.000217819	0.000221682	tRNA-modifying protein YgfZ
K06981	0	0	5.30825E-08	8.47917E-09	isopentenyl phosphate kinase [EC:2.7.4.26]
K06983	1.06114E-06	6.43555E-07	1.69565E-06	5.14205E-05	uncharacterized protein
K06984	9.87446E-07	4.21631E-08	6.3322E-08	1.33207E-07	beta-ribofuranosylaminobenzene 5-phosphate synthase [EC:2.4.2.54]
K06985	6.00726E-06	8.20435E-06	3.4585E-05	8.86362E-05	aspartyl protease family protein
K06986	0.000213818	0.00021243	0.000187798	0.000114971	uncharacterized protein
K06987	1.29862E-06	4.22118E-06	2.39179E-06	1.96628E-05	uncharacterized protein
K06988	3.6978E-06	4.00054E-06	5.23134E-06	4.92891E-05	8-hydroxy-5-deazaflavin:NADPH oxidoreductase [EC:1.5.1.40]
K06989	0.000215519	0.000215673	0.000200938	0.00014869	aspartate dehydrogenase [EC:1.4.1.21]
K06990	9.16819E-09	7.29593E-08	3.02035E-06	1.26922E-06	MEMO1 family protein
K06991	0.000216212	0.000215088	0.000203447	0.000154737	uncharacterized protein
K06992	0.000214633	0.000213092	0.000188699	0.00016738	uncharacterized protein
K06993	7.13117E-07	1.43911E-07	1.28822E-06	2.56067E-06	ribonuclease H-related protein
K06994	4.06892E-06	1.42401E-05	1.83087E-05	2.27951E-05	putative drug exporter of the RND superfamily
K06995	0.000429378	0.000424356	0.000378167	0.000238455	uncharacterized protein
K06996	0.00022075	0.000224404	0.000225324	0.000351506	uncharacterized protein
K06997	0.000653583	0.000651101	0.000616045	0.000538094	PLP dependent protein
K06998	0.00022172	0.000222942	0.000223022	0.00029413	trans-2,3-dihydro-3-hydroxyanthranilate isomerase [EC:5.3.3.17]
K06999	8.92925E-06	1.30423E-05	3.23785E-05	0.000134052	phospholipase/carboxylesterase
K07000	0.00021679	0.00021596	0.000205771	0.0001796	uncharacterized protein
K07001	0.001088637	0.001085111	0.001029746	0.000808323	NTE family protein
K07002	0.000219817	0.000218623	0.000228734	0.000193781	uncharacterized protein
K07003	3.45902E-06	3.46936E-06	1.4591E-05	2.22852E-05	uncharacterized protein
K07004	0.000219201	0.000222812	0.000217697	0.000286626	uncharacterized protein
K07005	0.000216178	0.000216651	0.000203599	0.000121004	uncharacterized protein
K07006	9.70298E-06	1.14386E-05	5.01052E-05	0.000119996	uncharacterized protein
K07007	0.000441669	0.000438769	0.000439618	0.000465206	uncharacterized protein
K07008	9.13545E-09	1.56499E-07	3.95172E-07	9.60897E-06	gamma-glutamyl hercynylcysteine S-oxide hydrolase [EC:3.5.1.118]
K07009	1.91802E-06	1.1965E-06	1.48932E-05	5.63997E-06	uncharacterized protein
K07010	6.10997E-06	6.30081E-06	2.94138E-05	0.000105826	putative glutamine amidotransferase
K07011	0.000222095	0.000223155	0.000219001	0.000244036	uncharacterized protein
K07012	4.28249E-06	5.5267E-06	2.969E-05	5.51318E-05	CRISPR-associated endonuclease/helicase Cas3 [EC:3.1.-.- 3.6.4.-]
K07013	0	1.66614E-09	0	0	uncharacterized protein
K07014	8.62907E-07	8.03424E-07	2.26203E-06	7.2011E-05	uncharacterized protein
K07015	2.22058E-06	8.52426E-07	7.66181E-06	5.93831E-06	uncharacterized protein
K07016	9.17166E-07	1.48454E-07	5.61121E-06	7.38698E-07	CRISPR-associated protein Csm1
K07017	5.59041E-06	4.35119E-06	1.00175E-05	0.000244862	uncharacterized protein
K07018	0.000219687	0.000217424	0.000210837	0.000209026	uncharacterized protein
K07019	0.000217685	0.000216573	0.000207967	0.000218849	uncharacterized protein
K07020	3.33084E-07	8.0704E-07	2.46078E-06	1.52622E-05	uncharacterized protein
K07022	0	0	1.34027E-07	0	uncharacterized protein
K07023	3.05311E-06	1.64434E-06	9.71929E-06	1.8207E-05	putative hydrolases of HD superfamily
K07024	5.89134E-08	0	1.47828E-07	0	sucrose-6-phosphatase [EC:3.1.3.24]
K07025	0.000439029	0.000436753	0.000403801	0.000308747	putative hydrolase of the HAD superfamily
K07026	4.33445E-09	5.10621E-08	5.78324E-07	3.51215E-06	mannosyl-3-phosphoglycerate phosphatase [EC:3.1.3.70]
K07027	5.87917E-06	4.68984E-06	2.00102E-05	1.43748E-05	glycosyltransferase 2 family protein
K07028	1.69444E-06	9.41774E-07	2.36129E-06	3.48394E-06	uncharacterized protein
K07029	4.03671E-06	5.96179E-06	1.68509E-05	5.64235E-05	diacylglycerol kinase (ATP) [EC:2.7.1.107]
K07030	4.13765E-06	4.77009E-06	1.59814E-05	5.87761E-06	uncharacterized protein
K07031	1.28503E-06	4.2963E-07	1.32489E-06	1.93727E-06	D-glycero-alpha-D-manno-heptose-7-phosphate kinase [EC:2.7.1.168]
K07032	4.55653E-06	7.19279E-06	1.97451E-05	0.000118449	uncharacterized protein
K07033	6.64899E-07	1.115E-06	9.39778E-07	3.28908E-08	uncharacterized protein
K07034	1.79042E-07	1.18787E-06	5.70921E-07	1.3904E-05	uncharacterized protein
K07035	3.23416E-06	4.07617E-07	9.83568E-06	1.12883E-06	uncharacterized protein
K07037	6.41902E-07	1.05695E-06	2.9525E-06	7.21064E-07	uncharacterized protein

K07038	4.73848E-06	3.0331E-06	1.57874E-05	0.000107495	inner membrane protein
K07039	5.36784E-06	7.00469E-06	3.11595E-05	8.55287E-05	uncharacterized protein
K07040	0.000221266	0.000222071	0.000225328	0.000222206	uncharacterized protein
K07041	1.73944E-07	3.78347E-07	1.01367E-06	3.45632E-06	uncharacterized protein
K07042	0.000223493	0.00022317	0.000227592	0.000225437	probable rRNA maturation factor
K07043	0.000652243	0.000649906	0.000609731	0.000541265	uncharacterized protein
K07044	0.000427712	0.000423993	0.000378179	0.000231744	uncharacterized protein
K07045	4.10578E-06	7.72901E-06	3.36957E-05	7.44036E-05	uncharacterized protein
K07046	7.68129E-07	1.21093E-06	4.75833E-06	7.49876E-06	L-fuconolactonase [EC:3.1.1.-]
K07048	6.87125E-07	6.0602E-07	9.69768E-06	6.1238E-06	phosphotriesterase-related protein
K07050	0.000215152	0.0002151	0.000202623	0.000149438	misacylated tRNA(Ala) deacylase [EC:3.1.1.-]
K07051	1.37621E-07	2.179E-07	2.36131E-07	4.79562E-08	uncharacterized protein
K07052	0.000233298	0.000230889	0.00025281	0.000240503	uncharacterized protein
K07053	0.000218968	0.000219934	0.000218175	0.000170397	3,5-nucleoside bisphosphate phosphatase [EC:3.1.3.97]
K07054	0.00021968	0.000220378	0.000211634	0.000160467	uncharacterized protein
K07056	0.00043887	0.000440209	0.000434318	0.00037384	16S rRNA (cytidine1402-2-O)-methyltransferase [EC:2.1.1.198]
K07057	0	0	1.05504E-07	0	N4-bis(aminopropyl)spermidine synthase [EC:2.5.1.128]
K07058	0.000442777	0.000442509	0.000438763	0.000435044	membrane protein
K07059	1.54502E-06	2.68506E-06	6.56183E-06	4.59644E-05	uncharacterized protein
K07061	4.22178E-07	8.03749E-08	3.5813E-07	4.67412E-07	CRISPR-associated protein Cmr1
K07062	0.000217393	0.000218777	0.000216389	0.000182963	toxin FitB [EC:3.1.-.-]
K07063	7.22121E-07	1.83486E-06	9.1996E-06	2.09543E-05	uncharacterized protein
K07064	1.04687E-06	1.70786E-06	7.87737E-06	1.82139E-05	uncharacterized protein
K07065	0	0	1.09989E-07	4.01858E-09	uncharacterized protein
K07066	0	0	2.09314E-08	0	uncharacterized protein
K07067	6.313E-07	8.76124E-07	7.19881E-06	7.07649E-06	diadenylate cyclase [EC:2.7.7.85]
K07068	2.19644E-06	4.80956E-06	2.01474E-05	6.26731E-05	uncharacterized protein
K07069	2.32031E-07	1.85968E-08	4.37854E-07	5.08728E-07	uncharacterized protein
K07070	1.3922E-06	7.60186E-07	2.1782E-06	1.76785E-05	uncharacterized protein
K07071	5.25734E-06	1.0183E-05	2.38047E-05	4.01101E-05	uncharacterized protein
K07072	9.69047E-07	1.53301E-09	8.07261E-09	2.3117E-08	(4-(4-[2-(gamma-L-glutamylamino)ethyl]phenoxymethyl)furan-2-yl)methanamine synthase [EC:2.5.1.131]
K07074	0.000216577	0.000215331	0.000203379	0.000189943	uncharacterized protein
K07075	4.07479E-06	5.90655E-06	2.31819E-05	3.88672E-05	uncharacterized protein
K07076	2.83725E-06	4.53192E-06	1.68724E-05	3.79247E-05	uncharacterized protein
K07077	2.90577E-07	1.62242E-06	2.21214E-06	3.92903E-06	uncharacterized protein
K07078	1.99134E-06	1.55125E-06	3.0945E-06	4.36386E-06	uncharacterized protein
K07079	1.72533E-06	1.55705E-06	7.34135E-06	7.42944E-06	uncharacterized protein
K07080	5.15969E-06	7.84646E-06	3.44161E-05	8.53072E-05	uncharacterized protein
K07082	0.000222361	0.000223528	0.000225418	0.000227368	UPF0755 protein
K07084	1.30004E-06	6.59231E-07	5.46806E-06	1.6979E-05	putative amino acid transporter
K07085	4.3439E-06	1.07588E-05	3.18987E-05	5.99752E-05	putative transport protein
K07086	1.19505E-07	3.14701E-07	1.61657E-06	4.43283E-06	uncharacterized protein
K07088	0.000227131	0.000229835	0.000267547	0.000293596	uncharacterized protein
K07089	3.22483E-06	2.71097E-06	1.58004E-05	1.42316E-05	uncharacterized protein
K07090	0.001530435	0.001529553	0.001461821	0.001309608	uncharacterized protein
K07091	0.000219836	0.000219089	0.000210862	0.000217806	lipopolysaccharide export system permease protein
K07092	0	6.87762E-08	4.01186E-08	1.32604E-07	uncharacterized protein
K07093	5.33318E-06	9.32628E-06	2.52318E-05	5.07679E-05	uncharacterized protein
K07094	1.09343E-06	2.09368E-07	3.43105E-06	3.79696E-08	putative glycerol-1-phosphate prenyltransferase [EC:2.5.1.-]
K07095	4.41459E-06	6.75325E-06	1.81119E-05	1.99746E-05	uncharacterized protein
K07096	3.81906E-07	1.36308E-07	6.70611E-07	1.00451E-07	uncharacterized protein
K07097	8.79054E-08	9.5208E-10	4.60034E-07	1.01746E-08	uncharacterized protein
K07098	1.00292E-05	1.21995E-05	4.38575E-05	9.74603E-05	uncharacterized protein
K07099	8.17444E-07	3.49115E-07	8.31681E-07	7.37784E-08	uncharacterized protein
K07100	0.000215897	0.000212621	0.000188548	0.000115403	putative phosphoribosyl transferase
K07101	0.000215946	0.000215318	0.000214114	0.000170448	uncharacterized protein
K07102	0.00022035	0.000218441	0.000209556	0.000257128	N-acetylmuramate 1-kinase [EC:2.7.1.221]
K07104	3.30963E-06	4.58058E-07	8.79264E-06	7.79826E-06	catechol 2,3-dioxygenase [EC:1.13.11.2]
K07105	9.85851E-07	8.33694E-07	1.43718E-06	1.50417E-06	uncharacterized protein
K07106	2.40225E-06	2.19433E-06	1.34829E-05	2.11214E-05	N-acetylmuramic acid 6-phosphate etherase [EC:4.2.1.126]
K07107	0.000876459	0.000877491	0.000863488	0.000842634	acyl-CoA thioester hydrolase [EC:3.1.2.-]
K07108	0	0	2.22133E-08	0	uncharacterized protein
K07109	1.69529E-07	6.05037E-07	9.22743E-07	1.26064E-05	uncharacterized protein
K07110	3.09545E-06	4.27734E-06	1.94869E-05	9.03613E-05	XRE family transcriptional regulator, fatty acid utilization regulator
K07112	0.00065516	0.00065359	0.000609068	0.00052735	uncharacterized protein
K07113	1.65118E-06	2.17922E-06	4.48566E-06	1.9751E-05	UPF0716 protein FxsA
K07114	0.000227601	0.000228352	0.000244574	0.0003087	Ca-activated chloride channel homolog
K07115	0.00021935	0.000217168	0.000206931	0.000217608	23S rRNA (adenine2030-N6)-methyltransferase [EC:2.1.1.266]
K07116	0.000215473	0.000215234	0.000204445	0.000153698	acyl-homoserine-lactone acylase [EC:3.5.1.97]
K07117	0.000216704	0.000216246	0.00020445	0.000199562	uncharacterized protein
K07118	0.000217514	0.000217778	0.000209348	0.000225831	uncharacterized protein
K07119	0.000220279	0.000220286	0.000218591	0.000203894	uncharacterized protein
K07120	3.63058E-06	3.80273E-06	2.07394E-05	4.72074E-05	uncharacterized protein
K07121	1.40087E-06	2.01616E-06	3.57662E-06	6.91483E-05	uncharacterized protein
K07122	0.000216447	0.000216865	0.000206158	0.000217022	phospholipid transport system transporter-binding protein
K07123	9.91579E-07	3.2924E-07	3.88258E-07	1.38785E-07	uncharacterized protein
K07124	0.001961321	0.001948087	0.001873717	0.001527152	uncharacterized protein
K07126	0.000658909	0.000647615	0.000621583	0.000500589	uncharacterized protein
K07127	0.000218656	0.000215882	0.000205481	0.000156644	5-hydroxyisourate hydrolase [EC:3.5.2.17]
K07128	1.07056E-06	1.39377E-07	9.69603E-07	6.07279E-08	uncharacterized protein
K07129	0	0	6.50889E-10	1.86974E-07	uncharacterized protein
K07130	0.000216516	0.00021544	0.000207967	0.000155573	arylformamidase [EC:3.5.1.9]
K07131	3.40167E-06	3.93293E-06	1.8049E-05	0.000106198	uncharacterized protein
K07132	0.000213947	0.000211903	0.000187095	0.000116416	ATP-dependent target DNA activator [EC:3.6.1.3]
K07133	5.9031E-06	5.81924E-06	4.21704E-05	5.27231E-06	uncharacterized protein
K07136	1.51964E-06	3.97943E-07	9.67563E-07	1.70878E-05	uncharacterized protein
K07137	3.82889E-06	6.62795E-06	2.87881E-05	5.68593E-05	uncharacterized protein

K07138	1.31073E-06	1.15232E-06	8.74951E-06	2.43437E-08	uncharacterized protein
K07139	2.66629E-06	1.97212E-06	1.01769E-05	1.76061E-05	uncharacterized protein
K07140	3.01036E-06	4.55318E-06	1.99808E-05	5.08426E-05	uncharacterized protein
K07141	0.000220212	0.000217138	0.000207547	0.00019201	molybdenum cofactor cytidylyltransferase [EC:2.7.7.76]
K07144	0	0	5.81429E-09	4.37121E-08	5-(aminomethyl)-3-furanmethanol phosphate kinase [EC:2.7.4.31]
K07145	1.21484E-06	5.05627E-09	3.2411E-06	6.58316E-08	heme oxygenase (staphylobilin-producing) [EC:1.14.99.48]
K07146	0.000220602	0.000223863	0.000229117	0.000251422	UPF0176 protein
K07147	0.000219264	0.000216478	0.000205045	0.000154312	methionine sulfoxide reductase catalytic subunit [EC:1.8.-.-]
K07148	3.11885E-06	5.5664E-06	1.22864E-05	6.58845E-05	uncharacterized protein
K07149	1.48227E-06	1.94742E-06	3.47245E-06	5.50085E-05	uncharacterized protein
K07150	8.35408E-07	1.11456E-06	2.01782E-06	1.48385E-06	uncharacterized protein
K07151	1.71257E-09	0	2.7048E-08	0	dolichyl-diphosphooligosaccharide---protein glycosyltransferase [EC:2.4.99.18]
K07152	0.001292672	0.001283181	0.001160306	0.000860507	protein SCO1/2
K07153	1.31472E-06	1.98742E-06	3.21381E-06	6.91247E-05	high frequency lysogenization protein
K07154	0.000649151	0.000649957	0.000623545	0.000518709	serine/threonine-protein kinase HipA [EC:2.7.11.1]
K07155	1.70841E-09	0	3.47019E-08	2.0899E-07	quercetin 2,3-dioxygenase [EC:1.13.11.24]
K07156	0.000218894	0.000218715	0.000203138	0.000189494	copper resistance protein C
K07157	0.00021803	0.000217171	0.000207869	0.000224121	uncharacterized protein
K07160	0.000435507	0.000433463	0.000410639	0.000300942	UPF0271 protein
K07161	0.000215551	0.000215474	0.000201673	0.000152372	uncharacterized protein
K07164	1.88789E-06	6.09814E-06	1.23525E-05	4.01934E-06	uncharacterized protein
K07165	0.000463293	0.000497423	0.000695962	0.001289293	transmembrane sensor
K07166	2.81321E-06	1.09366E-06	6.43015E-06	3.15112E-06	ACT domain-containing protein
K07167	1.39557E-06	7.51037E-07	4.07384E-06	6.79119E-05	putative transcriptional regulator
K07168	0.000216381	0.000215888	0.000204127	0.000165176	CBS domain-containing membrane protein
K07169	1.35765E-06	3.28964E-06	1.66727E-05	3.51975E-05	FHA domain-containing protein
K07171	4.15224E-06	3.43355E-06	9.54694E-06	1.00652E-05	mRNA interferase MazF [EC:3.1.-.-]
K07172	0.000428566	0.000424121	0.000372949	0.000228923	antitoxin MazE
K07173	4.76797E-06	1.9475E-06	2.63403E-05	1.80622E-05	S-ribosylhomocysteine lyase [EC:4.4.1.21]
K07175	0.000218429	0.000221129	0.000220636	0.000223001	PhoH-like ATPase
K07176	0	0	2.09314E-08	0	putative serine/threonine protein kinase
K07177	1.88699E-06	4.40248E-06	1.07833E-05	7.61929E-06	Lon-like protease
K07178	5.16705E-07	1.05077E-06	2.37499E-06	2.02843E-05	RIO kinase 1 [EC:2.7.11.1]
K07180	2.67146E-06	4.15159E-06	1.70079E-05	5.20748E-05	serine protein kinase
K07181	7.92877E-08	8.12463E-08	8.28525E-07	2.94615E-05	c-di-GMP phosphodiesterase [EC:3.1.4.52]
K07182	4.73684E-06	7.58849E-06	3.0866E-05	0.000101894	CBS domain-containing protein
K07183	0.000216621	0.000215076	0.000204489	0.000157161	two-component system, response regulator / RNA-binding antiterminator
K07184	2.33101E-07	7.50533E-07	1.37614E-06	1.76291E-05	SH3 domain protein
K07186	2.53557E-07	7.51755E-07	1.32021E-06	1.72377E-05	membrane protein
K07190	4.46815E-09	2.4421E-08	2.442E-08	3.42565E-07	phosphorylase kinase alpha/beta subunit
K07192	9.60292E-07	1.20233E-06	3.0983E-06	7.65215E-06	flotillin
K07212	0	4.42564E-08	1.04855E-07	8.26767E-06	GGDEF domain
K07213	0.000224558	0.000225279	0.000225836	0.000198252	copper chaperone
K07214	2.39253E-06	4.61012E-06	2.06564E-05	4.44993E-05	enterochelin esterase and related enzymes
K07215	2.4722E-06	1.11211E-06	2.89722E-06	6.12259E-05	heme oxygenase (biliverdin-IX-beta and delta-forming) [EC:1.14.99.58]
K07216	3.28391E-06	4.1119E-06	1.68158E-05	9.74162E-05	hemerythrin
K07217	3.65226E-06	4.86184E-06	4.66454E-06	6.90751E-05	Mn-containing catalase
K07218	0.000214111	0.000212504	0.000187443	0.000115557	nitrous oxidase accessory protein
K07219	1.25449E-06	5.48281E-07	2.29471E-06	9.70739E-07	putative molybdopterin biosynthesis protein
K07220	0.000219777	0.00022095	0.000209714	0.000216777	uncharacterized protein
K07221	5.38792E-06	4.02187E-06	7.78329E-06	0.000182323	phosphate-selective porin OprO and OprP
K07222	7.92743E-06	1.85287E-05	5.85388E-05	0.000180657	putative flavoprotein involved in K+ transport
K07223	2.36388E-06	3.38256E-06	1.27415E-05	9.37001E-05	putative iron-dependent peroxidase
K07224	1.85211E-06	7.85473E-07	1.60745E-06	4.98527E-06	iron uptake system component EfeO
K07225	0.000215217	0.000215976	0.000201786	0.000151976	putative hemin transport protein
K07226	2.64363E-06	3.82379E-06	1.56961E-05	4.76774E-05	heme iron utilization protein
K07227	3.55264E-07	3.01944E-08	5.59206E-07	1.18414E-05	heme iron utilization protein
K07228	8.75552E-07	2.77785E-06	7.29978E-07	1.82636E-06	TrkA domain protein
K07229	1.09011E-06	1.24576E-06	3.61713E-06	4.47659E-05	ferric-chelate reductase (NADPH) [EC:1.16.1.9]
K07230	1.1424E-06	1.94243E-07	4.82246E-07	3.52342E-07	periplasmic iron binding protein
K07231	1.22684E-06	3.44048E-06	1.76422E-05	4.37892E-05	putative iron-regulated protein
K07232	0.000216735	0.000215487	0.000203014	0.000149047	glutathione-specific gamma-glutamylcyclotransferase [EC:4.3.2.7]
K07233	0.000219251	0.000217002	0.000203029	0.000235018	copper resistance protein B
K07234	0.000217558	0.00021622	0.000205276	0.000172287	uncharacterized protein involved in response to NO
K07235	1.39264E-06	7.6074E-07	2.22142E-06	1.76899E-05	tRNA 2-thiouridine synthesizing protein D [EC:2.8.1.-]
K07236	8.74637E-07	7.56354E-07	1.90286E-06	1.76725E-05	tRNA 2-thiouridine synthesizing protein C
K07237	2.53557E-07	7.51755E-07	1.53474E-06	1.76371E-05	tRNA 2-thiouridine synthesizing protein B
K07238	7.46074E-06	1.03984E-05	3.21099E-05	9.45113E-05	zinc transporter, ZIP family
K07239	3.70842E-06	2.20871E-06	6.47654E-06	3.99316E-05	heavy-metal exporter, HME family
K07240	0.000866616	0.000865103	0.000827422	0.00062046	chromate transporter
K07241	0.000215037	0.000214897	0.000186896	0.000115591	high-affinity nickel-transport protein
K07242	4.28144E-09	0	0	0	putative multicomponent Na+:H+ antiporter subunit B
K07243	4.89338E-06	2.51326E-06	1.01922E-05	4.81543E-05	high-affinity iron transporter
K07245	0.00021818	0.00021905	0.000206305	0.000195893	copper resistance protein D
K07246	0.000862331	0.000859043	0.000787566	0.000561979	tartrate dehydrogenase/decarboxylase / D-malate dehydrogenase [EC:1.1.1.93 4.1.1.73 1.1.1.83]
K07248	6.0991E-06	1.09906E-05	5.34547E-05	0.000116672	lactaldehyde dehydrogenase / glycolaldehyde dehydrogenase [EC:1.2.1.22 1.2.1.21]
K07250	0.000434011	0.000438784	0.000427726	0.000358075	4-aminobutyrate aminotransferase / (S)-3-amino-2-methylpropionate transaminase [EC:2.6.1.19 2.6.1.22]
K07251	1.07786E-07	3.01003E-07	3.0033E-07	7.02266E-06	thiamine kinase [EC:2.7.1.89]
K07255	2.48908E-09	9.79603E-08	2.442E-08	3.28568E-09	taurine dehydrogenase small subunit [EC:1.4.2.-]
K07256	1.22275E-06	3.09592E-06	1.38915E-05	3.46427E-05	taurine dehydrogenase large subunit [EC:1.4.2.-]
K07257	1.14918E-07	8.04164E-08	8.66644E-07	3.97462E-06	spore coat polysaccharide biosynthesis protein SpsF
K07258	0.000226979	0.00022368	0.000222114	0.000282539	serine-type D-Ala-D-Ala carboxypeptidase (penicillin-binding protein 5/6) [EC:3.4.16.4]
K07259	0.000218381	0.000220932	0.000219243	0.000166495	serine-type D-Ala-D-Ala carboxypeptidase/endopeptidase (penicillin-binding protein 4) [EC:3.4.16.4 3.4.21.-]
K07260	4.05524E-06	5.42538E-06	1.48406E-05	6.55418E-05	zinc D-Ala-D-Ala carboxypeptidase [EC:3.4.17.14]
K07261	1.43812E-06	4.75544E-07	6.37938E-07	4.1559E-06	penicillin-insensitive murein DD-endopeptidase [EC:3.4.24.-]
K07262	0.000218265	0.000216499	0.000206624	0.000155888	serine-type D-Ala-D-Ala endopeptidase (penicillin-binding protein 7) [EC:3.4.21.-]
K07263	0.000443684	0.000441522	0.000437245	0.000406547	zinc protease [EC:3.4.24.-]

K07264	0.000214008	0.00021208	0.000186329	0.000114666	4-amino-4-deoxy-L-arabinose transferase [EC:2.4.2.43]
K07265	1.41032E-06	1.04306E-07	9.19126E-07	2.4914E-06	capsular polysaccharide export protein
K07266	1.39697E-06	8.52685E-08	1.10262E-06	9.42134E-07	capsular polysaccharide export protein
K07267	0.000220313	0.000213279	0.000188822	0.000178956	porin
K07268	0	0	2.22866E-08	1.14104E-08	opacity associated protein
K07269	9.81826E-08	3.14508E-07	3.30362E-07	1.22803E-05	uncharacterized protein
K07270	1.88154E-06	4.1482E-07	1.27E-06	7.16853E-06	glycosyl transferase, family 25
K07271	1.13867E-06	2.06884E-06	4.85034E-06	2.82195E-06	lipopolysaccharide cholinephosphotransferase [EC:2.7.8.-]
K07272	5.68298E-07	1.75199E-07	1.13597E-06	2.51767E-05	rhamnosyltransferase [EC:2.4.1.-]
K07273	4.15832E-06	3.50006E-06	1.76092E-05	5.60742E-06	lysozyme
K07274	2.63325E-06	1.15057E-06	4.40562E-06	8.55465E-05	MipA family protein
K07275	0.000646753	0.000645704	0.000597624	0.000528787	outer membrane protein
K07276	2.5349E-06	1.5037E-06	2.00629E-06	5.161E-05	uncharacterized protein
K07277	0.000219817	0.000218922	0.00021106	0.000217952	outer membrane protein insertion porin family
K07278	0.000219139	0.000217185	0.000207309	0.000217362	translocation and assembly module TamA
K07279	4.86396E-09	1.95811E-08	3.50274E-08	2.08786E-07	autotransporter family porin
K07280	0	0	8.72143E-09	0	outer membrane protein
K07281	6.64752E-08	7.94201E-08	6.4771E-08	2.34862E-07	1L-myo-inositol 1-phosphate cytidylyltransferase [EC:2.7.7.74]
K07282	3.83707E-06	3.13319E-06	2.28037E-05	5.23396E-06	gamma-polyglutamate biosynthesis protein CapA
K07283	1.17455E-06	1.81196E-06	2.20664E-06	6.42066E-05	putative salt-induced outer membrane protein
K07284	3.43804E-06	1.91775E-06	2.39416E-05	1.64373E-05	sortase A [EC:3.4.22.70]
K07285	1.24767E-06	1.66639E-06	2.21929E-06	6.54237E-05	outer membrane lipoprotein
K07286	2.5633E-07	7.62928E-07	1.47506E-06	1.7667E-05	uncharacterized lipoprotein
K07287	0.000216463	0.000216891	0.000206582	0.000217251	outer membrane protein assembly factor BamC
K07289	1.78038E-06	1.07381E-06	2.28659E-06	1.80542E-05	AsmA protein
K07290	0.000216656	0.000216523	0.000205048	0.000201808	AsmA family protein
K07291	6.64752E-08	7.94201E-08	6.4771E-08	2.34862E-07	CDP-L-myo-inositol myo-inositolphosphotransferase [EC:2.7.8.34]
K07300	2.72665E-06	9.06608E-07	2.42573E-06	5.97653E-06	Ca2+:H+ antiporter
K07301	3.76146E-06	4.7752E-06	1.76363E-05	9.29334E-05	cation:H+ antiporter
K07302	0.000651145	0.000647156	0.000609025	0.000466353	isoquinoline 1-oxidoreductase subunit alpha [EC:1.3.99.16]
K07303	0.000434809	0.000434445	0.000419739	0.000335907	isoquinoline 1-oxidoreductase subunit beta [EC:1.3.99.16]
K07304	0.000231084	0.000227481	0.000253415	0.000341911	peptide-methionine (S)-S-oxide reductase [EC:1.8.4.11]
K07305	0.000227386	0.000227732	0.000246098	0.000326661	peptide-methionine (R)-S-oxide reductase [EC:1.8.4.12]
K07306	0.000215572	0.000212617	0.00019563	0.000126492	anaerobic dimethyl sulfoxide reductase subunit A [EC:1.8.5.3]
K07307	6.18049E-07	9.95087E-07	7.04623E-06	5.82409E-06	anaerobic dimethyl sulfoxide reductase subunit B (DMSO reductase iron- sulfur subunit)
K07308	5.61979E-07	3.02608E-07	5.72355E-06	2.36892E-06	anaerobic dimethyl sulfoxide reductase subunit C (DMSO reductase anchor subunit)
K07309	0.000215683	0.000215231	0.00020737	0.000150742	Tat-targeted selenate reductase subunit YnfE [EC:1.97.1.9]
K07310	3.75433E-08	6.46425E-08	2.56547E-07	1.98801E-06	Tat-targeted selenate reductase subunit YnfF [EC:1.97.1.9]
K07311	5.66429E-08	3.8882E-08	1.18624E-07	1.4436E-06	Tat-targeted selenate reductase subunit YnfG
K07312	5.66655E-08	3.89385E-08	1.1427E-07	1.49995E-06	Tat-targeted selenate reductase subunit YnfH
K07313	4.36098E-06	2.43642E-06	5.6565E-06	9.97524E-06	serine/threonine protein phosphatase 1 [EC:3.1.3.16]
K07314	4.46207E-09	1.85815E-08	3.21332E-08	2.01591E-07	serine/threonine protein phosphatase 2 [EC:3.1.3.16]
K07315	9.30142E-06	1.15694E-05	4.33182E-05	0.000151612	phosphoserine phosphatase RsbU/P [EC:3.1.3.3]
K07316	4.13407E-06	3.6535E-06	1.6989E-05	2.9415E-05	adenine-specific DNA-methyltransferase [EC:2.1.1.72]
K07317	4.88006E-07	1.95825E-06	2.00773E-06	1.46084E-06	adenine-specific DNA-methyltransferase [EC:2.1.1.72]
K07318	6.83102E-07	6.27502E-07	1.27671E-06	2.15358E-06	adenine-specific DNA-methyltransferase [EC:2.1.1.72]
K07319	8.34779E-07	9.67947E-07	9.44141E-07	4.82304E-06	adenine-specific DNA-methyltransferase [EC:2.1.1.72]
K07320	0.000218202	0.000218043	0.000209557	0.000218389	ribosomal protein L3 glutamine methyltransferase [EC:2.1.1.298]
K07321	8.04494E-07	4.82203E-07	2.15522E-06	5.19854E-05	CO dehydrogenase maturation factor
K07322	0.000215737	0.000214857	0.00019767	0.000117878	regulator of cell morphogenesis and NO signaling
K07323	0.000217553	0.000217025	0.000206765	0.000221045	phospholipid transport system substrate-binding protein
K07326	0.000855765	0.000848203	0.00074619	0.000485677	hemolysin activation/secretion protein
K07332	9.96235E-07	1.49097E-06	9.13524E-06	2.16804E-05	archaeal flagellar protein FlaI
K07333	0	0	0	1.57691E-07	archaeal flagellar protein FlaJ
K07334	4.2916E-06	7.58366E-06	2.97E-05	9.3824E-05	toxin HigB-1
K07335	0.000218797	0.000221233	0.000220633	0.000162408	basic membrane protein A and related proteins
K07336	4.37358E-06	6.58488E-06	2.89677E-05	0.000114661	PKHD-type hydroxylase [EC:1.14.11.-]
K07337	9.82118E-08	1.11633E-06	7.32903E-07	2.16197E-05	penicillin-binding protein activator
K07338	1.22684E-06	3.44048E-06	1.73719E-05	4.37457E-05	uncharacterized protein
K07339	2.3812E-08	3.02993E-08	5.52815E-08	6.49149E-07	mRNA interferase HicA [EC:3.1.-.-]
K07340	3.82771E-06	1.95843E-06	3.94498E-06	4.85886E-05	inner membrane protein
K07341	1.98054E-06	1.76402E-06	1.0107E-05	1.04728E-05	death on curing protein
K07343	0.000214658	0.00021293	0.000189484	0.000131627	DNA transformation protein and related proteins
K07344	3.22896E-06	3.99233E-06	1.96863E-05	5.26507E-05	type IV secretion system protein TrbL
K07345	2.97138E-06	6.47663E-06	2.1694E-05	9.82186E-05	major type 1 subunit fimbrin (pilin)
K07346	3.18376E-06	4.87343E-06	8.24724E-06	0.000137933	fimbrial chaperone protein
K07347	5.25001E-06	9.12509E-06	2.80528E-05	0.000186984	outer membrane usher protein
K07348	3.66092E-08	5.28823E-08	4.62341E-08	1.4309E-06	minor fimbrial subunit
K07349	9.12681E-08	2.75856E-07	9.52794E-08	1.00761E-06	minor fimbrial subunit
K07350	8.7882E-08	2.54882E-07	1.15052E-07	1.45215E-06	minor fimbrial subunit
K07351	4.21536E-08	7.82364E-08	4.46143E-08	1.25623E-06	fimbrial protein
K07352	3.78009E-09	1.38267E-08	2.7222E-08	2.0836E-07	type 1 fimbrial protein
K07353	9.94385E-09	3.3997E-08	3.00626E-08	1.17004E-07	fimbrial chaperone protein
K07354	4.03249E-09	1.44545E-08	3.60168E-08	2.41636E-07	outer membrane usher protein
K07355	3.77685E-09	1.26689E-08	2.71987E-08	1.73323E-07	fimbrial-like protein
K07356	3.96807E-09	1.31445E-08	2.85757E-08	2.04725E-07	fimbrial protein
K07357	1.94444E-07	5.84406E-07	1.64343E-07	1.42476E-06	type 1 fimbriae regulatory protein FimB
K07358	1.19594E-07	2.92571E-07	1.94403E-07	1.29826E-06	type 1 fimbriae regulatory protein FimE
K07386	8.53897E-06	1.02821E-05	2.3691E-05	0.000225481	putative endopeptidase [EC:3.4.24.-]
K07387	2.22834E-06	4.94839E-06	2.24195E-05	5.7333E-05	putative metalloprotease [EC:3.4.24.-]
K07389	0.00021379	0.000212182	0.000186314	0.000119514	cytolysin-activating lysine-acyltransferase [EC:2.3.1.-]
K07390	0.000434199	0.000430203	0.000395961	0.000382395	monothiol glutaredoxin
K07391	0.000652345	0.000651373	0.000597627	0.000480741	magnesium chelatase family protein
K07392	0.00021411	0.000213359	0.000189298	0.000120137	AAA family ATPase
K07393	2.28805E-06	4.79942E-06	3.53563E-06	2.07937E-05	glutathionyl-hydroquinone reductase [EC:1.8.5.7]
K07394	0.000215097	0.000212979	0.00018835	0.000131557	SM-20-related protein
K07395	0.000218487	0.000215586	0.00020564	0.000202357	putative proteasome-type protease

K07396	0.000642799	0.000639152	0.000576248	0.000392513	putative protein-disulfide isomerase
K07397	0.000433643	0.000434408	0.000412296	0.000362103	putative redox protein
K07399	0.000216847	0.00021924	0.000211779	0.000155402	cytochrome c biogenesis protein
K07400	2.45336E-06	2.03446E-06	3.88857E-06	6.91319E-05	Fe/S biogenesis protein NfuA
K07401	1.25837E-06	8.34904E-07	1.68651E-06	1.2509E-05	selenoprotein W-related protein
K07402	0.000437782	0.000432877	0.000416009	0.00034146	xanthine dehydrogenase accessory factor
K07403	0.000215137	0.000213086	0.000191247	0.000121274	membrane-bound serine protease (ClpP class)
K07404	0.000217647	0.000217895	0.000208414	0.000153707	6-phosphogluconolactonase [EC:3.1.1.31]
K07405	3.42888E-07	9.20719E-07	2.586E-06	2.18841E-08	alpha-amylase [EC:3.2.1.1]
K07406	1.63223E-07	3.21691E-07	9.81049E-07	1.6876E-06	alpha-galactosidase [EC:3.2.1.22]
K07407	2.75134E-06	4.53736E-06	2.15109E-05	2.13669E-05	alpha-galactosidase [EC:3.2.1.22]
K07442	2.67819E-06	7.45116E-06	1.29191E-05	1.29422E-05	tRNA (adenine57-N1/adenine58-N1)-methyltransferase catalytic subunit [EC:2.1.1.219 2.1.1.220]
K07443	2.08028E-06	3.84713E-06	6.22274E-06	7.32824E-05	methylated-DNA-protein-cysteine methyltransferase related protein
K07444	0.000219523	0.000218422	0.000213488	0.00018349	putative N6-adenine-specific DNA methylase [EC:2.1.1.-]
K07445	1.39589E-08	2.96113E-08	1.42119E-07	4.58448E-07	putative DNA methylase
K07446	1.26152E-07	7.55746E-08	2.61598E-07	1.20864E-08	tRNA (guanine10-N2)-dimethyltransferase [EC:2.1.1.213]
K07447	0.000223507	0.000223733	0.000228049	0.000225459	putative holliday junction resolvase [EC:3.1.-.-]
K07448	0.000220077	0.00021702	0.00021271	0.000182154	restriction system protein
K07450	2.07027E-07	2.60248E-07	5.85622E-06	8.21985E-09	putative resolvase
K07451	0.000218555	0.00021657	0.000213542	0.000184738	5-methylcytosine-specific restriction enzyme A [EC:3.1.21.-]
K07452	3.9009E-06	4.93467E-06	6.47261E-06	2.73766E-05	5-methylcytosine-specific restriction enzyme B [EC:3.1.21.-]
K07453	2.40676E-07	5.86058E-07	2.74349E-06	6.92149E-06	putative restriction endonuclease
K07454	2.25999E-06	4.19968E-06	1.6422E-05	6.87369E-05	putative restriction endonuclease
K07455	1.3106E-06	2.1631E-06	9.40682E-06	2.40196E-05	recombination protein RecT
K07456	2.56663E-06	2.39214E-06	1.14107E-05	4.52287E-06	DNA mismatch repair protein MutS2
K07457	1.08967E-06	1.83275E-08	2.8866E-06	1.45993E-07	endonuclease III related protein
K07458	0.00021606	0.000213008	0.000191189	0.000123161	DNA mismatch endonuclease, patch repair protein [EC:3.1.-.-]
K07459	2.19628E-06	4.01826E-06	1.31268E-05	6.97962E-05	putative ATP-dependent endonuclease of the OLD family
K07460	0.000222194	0.000223398	0.00022107	0.00022163	putative endonuclease
K07461	6.31471E-06	2.36627E-06	1.27363E-05	7.53776E-05	putative endonuclease
K07462	0.000222087	0.000219693	0.000219478	0.000222111	single-stranded-DNA-specific exonuclease [EC:3.1.-.-]
K07464	1.19398E-06	1.77569E-06	4.74028E-06	7.71784E-06	CRISPR-associated exonuclease Cas4 [EC:3.1.12.1]
K07465	8.14197E-08	1.05864E-06	3.00427E-06	2.00411E-06	putative RecB family exonuclease
K07466	2.64796E-10	1.51786E-09	6.97714E-09	0	replication factor A1
K07467	9.41256E-07	1.5856E-06	7.05863E-06	3.0957E-05	phage replication initiation protein
K07469	1.03617E-06	4.55453E-07	1.34517E-06	5.80795E-06	aldehyde oxidoreductase [EC:1.2.99.7]
K07470	9.8215E-08	3.01011E-07	7.49452E-08	7.88634E-07	DNA gyrase inhibitor
K07471	4.35714E-09	1.83205E-08	3.13776E-08	1.93772E-07	probable transcriptional regulator
K07472	0	3.74292E-09	1.40599E-07	0	TBP-interacting protein
K07473	4.765E-06	3.2171E-06	2.26464E-05	3.25812E-05	DNA-damage-inducible protein J
K07474	3.35514E-06	2.38094E-06	1.55519E-05	3.00408E-05	phage terminase small subunit
K07476	1.05101E-06	2.06813E-07	3.36174E-06	3.03626E-08	toprim domain protein
K07478	0.000226534	0.000228309	0.00023251	0.000237579	putative ATPase
K07479	1.40382E-07	3.14627E-07	3.67542E-07	1.75008E-05	putative DNA topoisomerase
K07480	1.79799E-06	5.6752E-07	1.68885E-06	1.97831E-05	insertion element IS1 protein InsB
K07481	9.1189E-06	6.31641E-06	2.71008E-05	7.00736E-05	transposase, IS5 family
K07482	8.27177E-06	1.41389E-05	3.66491E-05	9.3872E-05	transposase, IS30 family
K07483	0.000264248	0.000259779	0.00032464	0.000525323	transposase
K07484	0.000441528	0.000436007	0.00046665	0.000532367	transposase
K07485	7.37951E-06	2.02035E-05	8.93661E-06	2.8699E-05	transposase
K07486	0.001076207	0.001063249	0.000955945	0.000772532	transposase
K07487	0.004924768	0.004875169	0.004302869	0.002608202	transposase
K07488	0.00042819	0.000423573	0.000372398	0.000226517	transposase
K07489	1.58003E-11	3.92987E-11	1.13785E-10	1.23756E-05	transposase
K07490	9.82118E-08	3.01003E-07	7.49218E-08	7.63931E-07	ferrous iron transport protein C
K07491	0.000225316	0.000218244	0.000236592	0.000300963	putative transposase
K07492	0.001737081	0.001720378	0.001536848	0.000953114	putative transposase
K07493	3.53431E-05	8.29326E-05	5.49788E-05	6.61916E-05	putative transposase
K07494	1.30533E-05	4.88777E-06	1.80285E-05	8.86044E-05	putative transposase
K07495	3.34089E-07	5.04126E-07	4.67947E-06	4.87811E-05	putative transposase
K07496	2.17949E-05	3.76152E-06	2.56136E-05	1.70594E-05	putative transposase
K07497	0.001396591	0.001365418	0.00142817	0.001751371	putative transposase
K07498	3.21555E-06	2.70482E-06	4.8884E-06	1.08522E-05	putative transposase
K07499	5.77848E-08	4.99842E-09	6.57216E-08	0	putative transposase
K07501	2.15162E-06	9.53943E-07	1.79295E-06	4.1051E-07	3-5 exonuclease
K07502	1.24799E-06	1.35041E-06	2.36453E-06	5.151E-05	uncharacterized protein
K07503	1.79149E-06	4.68577E-06	1.23114E-05	1.09756E-05	endonuclease [EC:3.1.-.-]
K07504	4.5968E-07	5.77167E-07	1.80867E-06	2.83228E-06	predicted type IV restriction endonuclease
K07505	1.99703E-07	1.92775E-07	2.95269E-06	5.41738E-06	regulatory protein RepA
K07506	0.000663194	0.000663143	0.000678854	0.000875221	AraC family transcriptional regulator
K07507	0.000225664	0.000222447	0.000230492	0.000341352	putative Mg2+ transporter-C (MgtC) family protein
K07508	1.53321E-07	1.99117E-07	2.4609E-07	9.53062E-08	acetyl-CoA acyltransferase 2 [EC:2.3.1.16]
K07516	0.000444062	0.000450125	0.000479123	0.000502054	3-hydroxyacyl-CoA dehydrogenase [EC:1.1.1.35]
K07518	0.00021536	0.000215524	0.000202491	0.000149881	hydroxybutyrate-dimer hydrolase [EC:3.1.1.22]
K07533	0.000218469	0.000218016	0.000218884	0.000165233	foldase protein PrsA [EC:5.2.1.8]
K07534	8.43985E-09	2.44891E-08	3.07213E-08	1.0175E-08	cyclohex-1-ene-1-carboxyl-CoA hydratase [EC:4.2.1.-]
K07535	1.53089E-06	3.84172E-06	1.87931E-05	4.12084E-05	2-hydroxycyclohexanecarboxyl-CoA dehydrogenase [EC:1.1.1.-]
K07536	7.86561E-07	1.12309E-06	2.04739E-06	4.65659E-05	2-ketocyclohexanecarboxyl-CoA hydrolase [EC:3.1.2.-]
K07537	2.25003E-08	0	2.37223E-08	2.05886E-08	cyclohexa-1,5-dienecarbonyl-CoA hydratase [EC:4.2.1.100]
K07538	0	1.22815E-08	0	2.14235E-08	6-hydroxycyclohex-1-ene-1-carbonyl-CoA dehydrogenase [EC:1.1.1.368]
K07539	0	3.74292E-09	6.81884E-08	8.03716E-09	6-oxocyclohex-1-ene-carbonyl-CoA hydrolase [EC:3.7.1.21]
K07540	0	2.83244E-08	2.00271E-07	1.35866E-08	benzylsuccinate synthase [EC:4.1.99.11]
K07543	8.96062E-09	0	0	4.01858E-09	benzylsuccinate CoA-transferase BbsE subunit [EC:2.8.3.15]
K07544	1.2908E-08	1.43284E-08	3.8791E-08	3.10721E-06	benzylsuccinate CoA-transferase BbsF subunit [EC:2.8.3.15]
K07545	8.99101E-09	1.34433E-08	6.97714E-09	7.4656E-08	(R)-benzylsuccinyl-CoA dehydrogenase [EC:1.3.8.3]
K07546	1.16005E-06	4.04624E-08	1.5445E-06	4.50804E-07	E-phenylitaconyl-CoA hydratase [EC:4.2.1.-]
K07548	0	0	6.34747E-08	4.01858E-09	2-[hydroxy(phenyl)methyl]-succinyl-CoA dehydrogenase BbsD subunit [EC:1.1.1.35]

K07549	0	0	0	4.01858E-09	benzoylsuccinyl-CoA thiolase BbsA subunit [EC:2.3.1.-]
K07550	5.39461E-08	2.83838E-08	4.37066E-08	1.46591E-08	benzoylsuccinyl-CoA thiolase BbsB subunit [EC:2.3.1.-]
K07552	0.000663698	0.000661601	0.000671156	0.000781032	MFS transporter, DHA1 family, multidrug resistance protein
K07557	1.44434E-10	1.847E-10	0	0	archaeosine synthase [EC:2.6.1.97]
K07559	1.12329E-06	2.0419E-06	9.73924E-06	4.372E-05	putative RNA 2-phosphotransferase [EC:2.7.1.-]
K07560	0.000221859	0.000223078	0.000225933	0.000221647	D-aminoacyl-tRNA deacylase [EC:3.1.1.96]
K07566	0.000437926	0.000438126	0.0004193	0.000358769	L-threonylcarbamoyladenylate synthase [EC:2.7.7.87]
K07568	0.000222036	0.00022034	0.000218081	0.000221445	S-adenosylmethionine:tRNA ribosyltransferase-isomerase [EC:2.4.99.17]
K07570	1.95797E-06	7.86555E-07	4.81399E-06	3.84926E-06	general stress protein 13
K07571	2.20712E-06	7.1803E-07	4.66291E-06	8.19245E-07	S1 RNA binding domain protein
K07574	0.000219715	0.000217704	0.000214663	0.000220128	RNA-binding protein
K07576	1.9835E-06	3.26757E-06	4.39162E-06	2.3988E-05	metallo-beta-lactamase family protein
K07577	3.93994E-06	6.09825E-06	1.97964E-05	8.92612E-05	putative mRNA 3-end processing factor
K07579	9.09551E-07	2.75788E-06	8.50609E-07	1.88616E-06	putative methylase
K07584	2.09262E-06	8.4589E-07	7.65482E-06	3.70057E-06	uncharacterized protein
K07586	1.31497E-06	3.27418E-07	4.19292E-06	5.53857E-06	uncharacterized protein
K07588	3.56339E-06	6.80048E-06	3.08404E-05	4.79175E-05	LAO/AO transport system kinase [EC:2.7.-.-]
K07589	1.18688E-07	8.14961E-07	1.17046E-06	6.10945E-06	D-erythro-7,8-dihydroneopterin triphosphate epimerase [EC:5.1.99.7]
K07590	1.31405E-06	3.7792E-07	3.65158E-06	3.41661E-08	large subunit ribosomal protein L7A
K07591	4.41122E-10	1.09716E-09	3.1767E-09	1.3067E-08	threonine dehydratase operon activator protein
K07592	1.49374E-06	3.25914E-06	1.42824E-05	3.5736E-05	LysR family transcriptional regulator, tdc operon transcriptional activator
K07636	0.000701599	0.000711716	0.000825403	0.001109208	two-component system, OmpR family, phosphate regulon sensor histidine kinase PhoR [EC:2.7.13.3]
K07637	7.11702E-06	8.92652E-06	3.49373E-05	0.00020292	two-component system, OmpR family, sensor histidine kinase PhoQ [EC:2.7.13.3]
K07638	0.000658968	0.000653875	0.000633574	0.000718263	two-component system, OmpR family, osmolarity sensor histidine kinase EnvZ [EC:2.7.13.3]
K07639	6.31465E-06	6.60468E-06	1.51359E-05	0.000123196	two-component system, OmpR family, sensor histidine kinase RstB [EC:2.7.13.3]
K07640	0.000219924	0.000224145	0.000224667	0.000281313	two-component system, OmpR family, sensor histidine kinase CpxA [EC:2.7.13.3]
K07641	0.000216495	0.000215688	0.000206464	0.000203795	two-component system, OmpR family, sensor histidine kinase CreC [EC:2.7.13.3]
K07642	0.000232561	0.000238739	0.000269158	0.000648783	two-component system, OmpR family, sensor histidine kinase BaeS [EC:2.7.13.3]
K07643	1.13415E-07	3.73139E-07	1.18899E-06	1.32027E-06	two-component system, OmpR family, sensor histidine kinase BasS [EC:2.7.13.3]
K07644	0.000659043	0.000668562	0.000672099	0.000703983	two-component system, OmpR family, heavy metal sensor histidine kinase CusS [EC:2.7.13.3]
K07645	0.000657138	0.000654102	0.000646546	0.000647641	two-component system, OmpR family, sensor histidine kinase QseC [EC:2.7.13.3]
K07646	0.000661796	0.000665661	0.000689539	0.000750097	two-component system, OmpR family, sensor histidine kinase KdpD [EC:2.7.13.3]
K07647	0.000232016	0.000225022	0.000234352	0.000452582	two-component system, OmpR family, sensor histidine kinase TorS [EC:2.7.13.3]
K07648	6.81308E-06	1.04996E-05	3.80448E-05	0.000146502	two-component system, OmpR family, aerobic respiration control sensor histidine kinase ArcB [EC:2.7.13.3]
K07649	0.001524891	0.001514202	0.001451847	0.001385654	two-component system, OmpR family, sensor histidine kinase TctE [EC:2.7.13.3]
K07650	1.0332E-07	1.30357E-07	3.13285E-06	4.21235E-06	two-component system, OmpR family, sensor histidine kinase CssS [EC:2.7.13.3]
K07651	7.67415E-06	7.66473E-06	3.08335E-05	9.63243E-05	two-component system, OmpR family, sensor histidine kinase ResE [EC:2.7.13.3]
K07652	7.87728E-06	8.45463E-06	4.08868E-05	4.18972E-05	two-component system, OmpR family, sensor histidine kinase VicK [EC:2.7.13.3]
K07653	0.00022026	0.000224176	0.000254538	0.000279939	two-component system, OmpR family, sensor histidine kinase MprB [EC:2.7.13.3]
K07654	0.000219254	0.000218408	0.00020735	0.000144286	two-component system, OmpR family, sensor histidine kinase MtrB [EC:2.7.13.3]
K07655	3.36927E-07	1.10102E-06	3.86392E-06	3.46189E-06	two-component system, OmpR family, sensor histidine kinase PrrB [EC:2.7.13.3]
K07656	0.000213811	0.000211908	0.000186734	0.00011331	two-component system, OmpR family, sensor histidine kinase TrcS [EC:2.7.13.3]
K07657	0.001117246	0.001121391	0.001151878	0.001367512	two-component system, OmpR family, phosphate regulon response regulator PhoB
K07658	0.00067734	0.000686085	0.000720498	0.000802038	two-component system, OmpR family, alkaline phosphatase synthesis response regulator PhoP
K07659	0.000880384	0.000879796	0.000873075	0.000984487	two-component system, OmpR family, phosphate regulon response regulator OmpR
K07660	0.000656322	0.000655639	0.000642259	0.000679532	two-component system, OmpR family, response regulator PhoP
K07661	6.13565E-06	3.05039E-06	1.25127E-05	0.00011245	two-component system, OmpR family, response regulator RstA
K07662	0.000447024	0.000444573	0.000469623	0.000593612	two-component system, OmpR family, response regulator CpxR
K07663	0.000652756	0.000647178	0.000615945	0.000556712	two-component system, OmpR family, catabolic regulation response regulator CreB
K07664	0.000650396	0.000647697	0.000618877	0.000552657	two-component system, OmpR family, response regulator BaeR
K07665	0.001314126	0.00131061	0.001273463	0.001458465	two-component system, OmpR family, copper resistance phosphate regulon response regulator CusR
K07666	0.00131584	0.001318737	0.001331407	0.001467096	two-component system, OmpR family, response regulator QseB
K07667	0.000897451	0.000900056	0.000974839	0.001163104	two-component system, OmpR family, KDP operon response regulator KdpE
K07668	1.63465E-05	1.15922E-05	3.69616E-05	0.00015458	two-component system, OmpR family, response regulator VicR
K07669	0.000869097	0.000869963	0.000844546	0.000700698	two-component system, OmpR family, response regulator MprA
K07670	1.42742E-05	2.44881E-05	7.06562E-05	0.000315877	two-component system, OmpR family, response regulator MtrA
K07671	0.000215376	0.000216603	0.000202485	0.000154891	two-component system, OmpR family, response regulator PrrA
K07672	2.64829E-06	3.62621E-06	6.37394E-06	3.06903E-07	two-component system, OmpR family, response regulator TrcR
K07673	0.000218076	0.000216906	0.000214767	0.000239759	two-component system, NarL family, nitrate/nitrite sensor histidine kinase NarX [EC:2.7.13.3]
K07674	1.10695E-06	4.11865E-07	1.40799E-06	1.37633E-05	two-component system, NarL family, nitrate/nitrite sensor histidine kinase NarQ [EC:2.7.13.3]
K07675	0.000224382	0.000223848	0.000237022	0.000278193	two-component system, NarL family, sensor histidine kinase UhpB [EC:2.7.13.3]
K07676	1.00901E-07	3.11966E-07	7.5109E-08	8.01035E-07	two-component system, NarL family, sensor histidine kinase RcsD [EC:2.7.13.3]
K07677	0.00024096	0.000245065	0.000304887	0.000698184	two-component system, NarL family, capsular synthesis sensor histidine kinase RcsC [EC:2.7.13.3]
K07678	0.000244196	0.000239493	0.000290731	0.000635772	two-component system, NarL family, sensor histidine kinase BarA [EC:2.7.13.3]
K07679	0.000465752	0.000471014	0.000554898	0.001077489	two-component system, NarL family, sensor histidine kinase EvgS [EC:2.7.13.3]
K07680	5.08008E-07	8.06306E-07	5.9473E-06	5.40953E-06	two-component system, NarL family, sensor histidine kinase ComP [EC:2.7.13.3]
K07681	9.68623E-07	9.5208E-10	3.2572E-06	3.22644E-08	two-component system, NarL family, vancomycin resistance sensor histidine kinase VraS [EC:2.7.13.3]
K07682	1.98094E-07	1.78523E-07	6.86878E-07	6.13672E-06	two-component system, NarL family, sensor histidine kinase DevS [EC:2.7.13.3]
K07683	1.97897E-06	6.92097E-08	3.42211E-06	1.82759E-06	two-component system, NarL family, sensor histidine kinase NreB [EC:2.7.13.3]
K07684	0.000449224	0.000448596	0.000478253	0.000729765	two-component system, NarL family, nitrate/nitrite response regulator NarL
K07685	1.25871E-06	8.00017E-07	3.75832E-06	2.47125E-05	two-component system, NarL family, nitrate/nitrite response regulator NarP
K07686	1.64566E-06	3.69873E-06	1.51587E-05	6.5793E-05	two-component system, NarL family, uhpT operon response regulator UhpA
K07687	0.000439303	0.000440396	0.000442515	0.000627438	two-component system, NarL family, captular synthesis response regulator RcsB
K07688	0.000213914	0.000212282	0.000186891	0.000120429	two-component system, NarL family, response regulator, fimbrial Z protein, FimZ
K07689	0.000223405	0.00023096	0.000238501	0.000317175	two-component system, NarL family, invasion response regulator UvrY
K07690	7.6689E-07	1.19214E-06	4.2701E-06	1.53918E-05	two-component system, NarL family, response regulator EvgA
K07691	5.54378E-08	2.05861E-07	3.38649E-06	8.89213E-08	two-component system, NarL family, competent response regulator ComA
K07692	2.76988E-06	4.01331E-06	1.51102E-05	6.56194E-05	two-component system, NarL family, response regulator DegU
K07693	0.000229791	0.000240479	0.000260428	0.00037989	two-component system, NarL family, response regulator DesR
K07694	9.36655E-07	0	2.86403E-06	3.03626E-08	two-component system, NarL family, vancomycin resistance associated response regulator VraR
K07695	9.86528E-07	1.6175E-06	2.94666E-06	4.7117E-05	two-component system, NarL family, response regulator DevR
K07696	8.27625E-06	1.31208E-05	3.69231E-05	0.000148152	two-component system, NarL family, response regulator NreC
K07697	2.7614E-06	4.67172E-06	1.76382E-05	3.55763E-05	two-component system, sporulation sensor kinase B [EC:2.7.13.3]
K07698	1.71257E-09	2.44515E-07	3.30558E-08	0	two-component system, sporulation sensor kinase C [EC:2.7.13.3]
K07699	3.65064E-06	1.88941E-06	4.76243E-06	1.33041E-06	two-component system, response regulator, stage 0 sporulation protein A
K07700	4.41332E-07	5.27435E-07	7.73362E-07	7.96673E-06	two-component system, CitB family, cit operon sensor histidine kinase CitA [EC:2.7.13.3]
K07701	5.34324E-07	5.45686E-07	1.15956E-06	3.36627E-06	two-component system, CitB family, sensor histidine kinase DcuS [EC:2.7.13.3]

K07702	2.60856E-07	6.64756E-07	3.27502E-07	7.86414E-06	two-component system, CitB family, response regulator CitB
K07703	1.07554E-07	3.11816E-07	1.0729E-07	9.62983E-07	two-component system, CitB family, response regulator DcuR
K07704	3.36722E-06	3.14551E-06	9.84038E-06	9.8126E-05	two-component system, LytTR family, sensor histidine kinase LytS [EC:2.7.13.3]
K07705	2.46782E-06	4.44505E-06	1.44593E-05	9.45965E-06	two-component system, LytTR family, response regulator LytT
K07706	1.97493E-06	8.59748E-07	4.28339E-06	5.52219E-06	two-component system, LytTR family, sensor histidine kinase AgrC [EC:2.7.13.3]
K07707	1.48544E-06	9.69844E-07	3.66405E-06	5.34495E-06	two-component system, LytTR family, response regulator AgrA
K07708	0.000439581	0.000435519	0.00041472	0.00038528	two-component system, NtrC family, nitrogen regulation sensor histidine kinase GlnL [EC:2.7.13.3]
K07709	3.39917E-06	5.14336E-06	2.16379E-05	9.73737E-05	two-component system, NtrC family, sensor histidine kinase HydH [EC:2.7.13.3]
K07710	2.45699E-06	1.31021E-06	3.66724E-06	5.44269E-05	two-component system, NtrC family, sensor histidine kinase AtoS [EC:2.7.13.3]
K07711	2.16138E-06	2.84213E-06	8.66984E-06	2.2652E-05	two-component system, NtrC family, sensor histidine kinase GlrK [EC:2.7.13.3]
K07712	0.00174821	0.001743641	0.001701028	0.0016561	two-component system, NtrC family, nitrogen regulation response regulator GlnG
K07713	0.001314138	0.001313568	0.001294265	0.001228921	two-component system, NtrC family, response regulator HydG
K07714	0.001526069	0.001519473	0.001461988	0.001292957	two-component system, NtrC family, response regulator AtoC
K07715	0.001306597	0.001302245	0.001243574	0.001181963	two-component system, NtrC family, response regulator GlrR
K07716	0.000245812	0.000236676	0.000282812	0.000524467	two-component system, cell cycle sensor histidine kinase PleC [EC:2.7.13.3]
K07717	6.66213E-07	2.92045E-07	6.81145E-07	1.87639E-06	two-component system, sensor histidine kinase YcbA [EC:2.7.13.3]
K07718	8.24465E-06	7.81546E-06	2.81877E-05	0.000148281	two-component system, sensor histidine kinase YesM [EC:2.7.13.3]
K07719	6.76508E-07	2.78047E-07	7.18657E-07	2.72281E-08	two-component system, response regulator YcbB
K07720	0.000455082	0.000465324	0.000511699	0.000585075	two-component system, response regulator YesN
K07722	1.57503E-06	3.07706E-06	1.55922E-05	3.55148E-05	CopG family transcriptional regulator, nickel-responsive regulator
K07723	1.09172E-06	2.58456E-07	3.36544E-06	1.90634E-07	CopG family transcriptional regulator / antitoxin EndoAI
K07724	1.88152E-07	3.09883E-07	8.172E-07	1.58656E-06	Ner family transcriptional regulator
K07726	0.000223551	0.000216161	0.000210494	0.000187729	putative transcriptional regulator
K07727	1.04447E-05	6.69015E-06	2.78054E-05	0.000100622	putative transcriptional regulator
K07728	0	0	0	1.60743E-08	putative transcriptional regulator
K07729	0.000229024	0.000228896	0.000231514	0.000216447	putative transcriptional regulator
K07733	5.19479E-06	3.94778E-06	1.84814E-05	9.19225E-05	prophage regulatory protein
K07734	0.000219212	0.000216425	0.00020785	0.000214095	transcriptional regulator
K07735	0.000220211	0.000219963	0.000219016	0.000219658	putative transcriptional regulator
K07736	4.67584E-06	4.90057E-06	1.17803E-05	4.28657E-06	CarD family transcriptional regulator
K07738	0.000223251	0.000222251	0.000222442	0.000225458	transcriptional repressor NrdR
K07739	6.83365E-09	5.25626E-08	2.60206E-07	2.44344E-06	elongator complex protein 3 [EC:2.3.1.48]
K07740	2.53545E-07	7.51726E-07	1.46819E-06	1.7663E-05	regulator of sigma D
K07741	1.62028E-06	1.57833E-06	7.57558E-06	1.75476E-05	anti-repressor protein
K07742	4.24766E-06	1.54317E-06	1.16588E-05	5.94313E-06	uncharacterized protein
K07743	0	0	2.94399E-08	0	transcriptional regulator
K07744	0	0	9.0419E-08	0	transcriptional regulator
K07746	4.89245E-06	8.4208E-06	3.39848E-05	0.000118799	antitoxin ParD1/3/4
K07749	0.001112066	0.001133225	0.001276147	0.001354948	formyl-CoA transferase [EC:2.8.3.16]
K07751	2.69808E-07	8.50876E-07	1.38292E-06	2.44803E-05	PepB aminopeptidase [EC:3.4.11.23]
K07755	3.96633E-06	3.68247E-06	6.48685E-06	4.6306E-06	arsenite methyltransferase [EC:2.1.1.137]
K07757	1.58552E-06	4.15012E-07	9.58119E-06	2.63915E-06	sugar-phosphatase [EC:3.1.3.23]
K07768	0.000221544	0.00021873	0.000205954	0.000130095	two-component system, OmpR family, sensor histidine kinase SenX3 [EC:2.7.13.3]
K07769	0.000215685	0.00021266	0.000190115	0.000124209	two-component system, OmpR family, sensor histidine kinase NblS [EC:2.7.13.3]
K07770	1.45628E-06	2.55507E-06	6.44181E-06	5.56378E-05	two-component system, OmpR family, response regulator CssR
K07771	0.00043829	0.000437984	0.000441717	0.000544194	two-component system, OmpR family, response regulator BasR
K07772	2.13467E-06	4.32725E-06	1.80552E-05	6.9197E-05	two-component system, OmpR family, torCAD operon response regulator TorR
K07773	1.74542E-06	3.50883E-06	1.44881E-05	5.21486E-05	two-component system, OmpR family, aerobic respiration control protein ArcA
K07774	0.001302636	0.001302142	0.001250657	0.001172849	two-component system, OmpR family, response regulator TctD
K07775	0.000227238	0.000226198	0.000231254	0.000339814	two-component system, OmpR family, response regulator ResD
K07776	0.0002265	0.000225617	0.000230234	0.000315658	two-component system, OmpR family, response regulator RegX3
K07777	3.20152E-06	5.75361E-06	2.65744E-05	8.72958E-05	two-component system, NarL family, sensor histidine kinase DegS [EC:2.7.13.3]
K07778	8.73377E-06	1.48326E-05	2.80875E-05	0.000214424	two-component system, NarL family, sensor histidine kinase DesK [EC:2.7.13.3]
K07781	1.67914E-07	5.82097E-07	1.04413E-07	7.51667E-07	LuxR family transcriptional regulator, capsular biosynthesis positive transcription factor
K07782	0.000214621	0.000213043	0.000191513	0.000124191	LuxR family transcriptional regulator, quorum-sensing system regulator SdiA
K07783	2.02137E-07	6.12073E-07	8.6217E-06	2.37417E-05	MFS transporter, OPA family, sugar phosphate sensor protein UhpC
K07784	5.30825E-08	1.95136E-08	1.95595E-06	8.12646E-06	MFS transporter, OPA family, hexose phosphate transport protein UhpT
K07785	0	1.77569E-10	8.65169E-08	7.25206E-08	MFS transporter, NRE family, putaive nickel resistance protein
K07786	4.47606E-07	7.60566E-08	3.83276E-07	7.45511E-07	MFS transporter, DHA2 family, multidrug resistance protein
K07787	0.000438768	0.000437108	0.000435377	0.000475261	Cu(I)/Ag(I) efflux system membrane protein CusA/SilA
K07788	0.000656766	0.000653805	0.000611778	0.000603938	multidrug efflux pump
K07789	0.000663342	0.000663267	0.000657106	0.000804544	multidrug efflux pump
K07790	3.28663E-09	3.47372E-08	1.69602E-07	1.82053E-07	putative membrane protein PagO
K07791	2.21618E-06	2.40415E-06	1.90225E-05	2.61371E-05	anaerobic C4-dicarboxylate transporter DcuA
K07792	9.07165E-07	2.42845E-06	9.79709E-06	2.04739E-05	anaerobic C4-dicarboxylate transporter DcuB
K07793	0.000222501	0.000225589	0.000247848	0.000243039	putative tricarboxylic transport membrane protein
K07794	3.05666E-06	6.87543E-06	2.11992E-05	5.42091E-05	putative tricarboxylic transport membrane protein
K07795	3.72926E-05	9.05132E-05	0.000437684	0.001028432	putative tricarboxylic transport membrane protein
K07796	1.01372E-06	5.56339E-07	1.84197E-06	5.40102E-05	outer membrane protein, Cu(I)/Ag(I) efflux system
K07797	4.47726E-09	1.84447E-08	3.12025E-08	5.42463E-07	multidrug resistance protein K
K07798	0.000219501	0.000219014	0.00021867	0.000240017	membrane fusion protein, Cu(I)/Ag(I) efflux system
K07799	0.000227657	0.000226769	0.000245166	0.000405732	membrane fusion protein, multidrug efflux system
K07800	9.81336E-07	1.25557E-07	2.77165E-06	4.98441E-08	AgrD protein
K07803	2.83425E-08	1.83437E-08	3.14447E-08	6.41543E-07	zinc resistance-associated protein
K07804	1.1815E-08	4.20114E-08	9.20622E-08	4.80387E-07	putatice virulence related protein PagC
K07805	2.83595E-12	7.05362E-12	2.04229E-11	8.40071E-11	putative membrane protein PagD
K07806	0.000216588	0.00021304	0.00019075	0.000128546	UDP-4-amino-4-deoxy-L-arabinose-oxoglutarate aminotransferase [EC:2.6.1.87]
K07807	1.1468E-06	9.04706E-08	7.47589E-07	2.35214E-06	uncharacterized protein
K07810	1.58727E-06	1.39818E-06	6.59741E-06	2.13955E-05	Cu(I)/Ag(I) efflux system periplasmic protein CusF
K07811	1.41031E-07	5.20095E-07	1.52854E-06	2.34653E-05	trimethylamine-N-oxide reductase (cytochrome c) [EC:1.7.2.3]
K07812	2.71056E-07	1.01376E-06	1.83682E-06	1.94875E-05	trimethylamine-N-oxide reductase (cytochrome c) [EC:1.7.2.3]
K07813	1.64902E-06	6.73607E-07	3.55845E-06	5.40239E-07	accessory gene regulator B
K07814	0.000463951	0.000471289	0.000579822	0.000887245	putative two-component system response regulator
K07816	3.92829E-06	4.02551E-06	8.60783E-06	8.21515E-06	putative GTP pyrophosphokinase [EC:2.7.6.5]
K07821	7.41713E-08	4.1914E-07	4.32524E-07	1.20616E-05	trimethylamine-N-oxide reductase (cytochrome c), cytochrome c-type subunit TorY
K07823	0.000218165	0.000223395	0.000208715	0.000175712	3-oxoadipyl-CoA thiolase [EC:2.3.1.174]
K07862	0.000216966	0.000216501	0.000205777	0.000178677	serine/threonine transporter

K07979	0.000221777	0.000223621	0.00024121	0.000262131	GntR family transcriptional regulator
K08068	0.000214157	0.000212865	0.000188684	0.000118294	UDP-N-acetylglucosamine 2-epimerase (hydrolysing) [EC:3.2.1.183]
K08070	1.29685E-06	9.26564E-07	3.36827E-06	7.0115E-06	2-alkenal reductase [EC:1.3.1.74]
K08077	0	0	1.54111E-08	0	UDP-sugar diphosphatase [EC:3.6.1.45]
K08080	0	0	1.04142E-08	0	CMP-N-acetylneuraminate monooxygenase [EC:1.14.18.2]
K08081	3.26013E-06	2.72999E-06	4.28542E-06	8.30486E-05	tropinone reductase I [EC:1.1.1.206]
K08082	5.75788E-06	8.17414E-06	4.05202E-05	0.000136317	two-component system, LytTR family, sensor histidine kinase AlgZ [EC:2.7.13.3]
K08083	1.13545E-05	1.37545E-05	8.0637E-05	0.000221072	two-component system, LytTR family, response regulator AlgR
K08084	0.001079695	0.001076555	0.001002832	0.000829244	type IV fimbrial biogenesis protein FimT
K08085	2.67847E-07	2.54695E-07	1.23587E-06	2.30655E-05	type IV fimbrial biogenesis protein FimU
K08086	0.000431422	0.000428466	0.000394225	0.000332456	pilus assembly protein FimV
K08087	1.52903E-07	6.16073E-07	4.37982E-08	2.67127E-08	fimbrial protein FimW
K08088	2.83595E-12	1.15681E-09	6.99757E-09	8.65624E-08	fimbrial protein FimY
K08092	1.17418E-07	1.94948E-07	1.07637E-07	3.42428E-07	3-dehydro-L-gulonate 2-dehydrogenase [EC:1.1.1.130]
K08093	2.05455E-06	2.84186E-06	7.08828E-06	6.32208E-07	3-hexulose-6-phosphate synthase [EC:4.1.2.43]
K08094	1.14938E-06	1.46095E-07	6.82818E-06	3.92962E-07	6-phospho-3-hexuloisomerase [EC:5.3.1.27]
K08095	0	1.51923E-06	1.87628E-07	6.71403E-07	cutinase [EC:3.1.1.74]
K08097	9.16819E-09	2.12879E-08	1.7984E-07	8.49675E-08	phosphosulfolactate synthase [EC:4.4.1.19]
K08100	1.96649E-07	6.66288E-07	1.82538E-06	2.6128E-06	bilirubin oxidase [EC:1.3.3.5]
K08137	1.84776E-06	6.79931E-07	5.51344E-06	2.41604E-05	MFS transporter, SP family, galactose:H+ symporter
K08138	2.71763E-06	5.99123E-06	1.05273E-05	2.26471E-05	MFS transporter, SP family, xylose:H+ symportor
K08139	2.86714E-06	4.34536E-06	6.04285E-06	6.40256E-05	MFS transporter, SP family, sugar:H+ symporter
K08151	1.534E-06	2.32305E-06	7.28E-06	3.05551E-05	MFS transporter, DHA1 family, tetracycline resistance protein
K08152	8.48152E-08	1.46474E-09	3.48957E-07	5.24977E-07	MFS transporter, DHA1 family, multidrug resistance protein B
K08153	1.41093E-06	3.53299E-07	4.10964E-06	4.55731E-06	MFS transporter, DHA1 family, multidrug resistance protein
K08154	3.04117E-07	4.95927E-07	1.7546E-06	2.51483E-05	MFS transporter, DHA1 family, 2-module integral membrane pump EmrD
K08155	0	0	0	4.27891E-09	MFS transporter, DHA1 family, solute carrier family 18 (vesicular amine transporter), member 1/2
K08156	4.04942E-06	1.50736E-06	8.68765E-06	4.77008E-05	MFS transporter, DHA1 family, arabinose polymer utilization protein
K08159	3.14401E-06	3.99988E-06	1.52114E-05	4.43586E-05	MFS transporter, DHA1 family, L-arabinose/isopropyl-beta-D-thiogalactopyranoside export protein
K08160	3.95614E-07	5.01526E-07	9.67563E-07	1.36199E-05	MFS transporter, DHA1 family, multidrug/chloramphenicol efflux transport protein
K08161	0.000214679	0.00021278	0.000188447	0.000118764	MFS transporter, DHA1 family, multidrug resistance protein
K08162	1.63166E-07	4.27544E-07	3.96329E-07	4.28097E-06	MFS transporter, DHA1 family, multidrug resistance protein
K08163	2.83798E-08	1.95861E-08	3.08772E-07	6.02125E-06	MFS transporter, DHA1 family, multidrug resistance protein
K08164	5.14752E-08	3.8671E-08	6.75972E-08	4.02153E-09	MFS transporter, DHA1 family, putative efflux transporter
K08166	0.00043565	0.000436327	0.000426648	0.000430696	MFS transporter, DHA2 family, methylenomycin A resistance protein
K08167	4.59569E-06	9.26936E-06	4.16632E-05	0.000172304	MFS transporter, DHA2 family, multidrug resistance protein
K08168	1.01541E-06	3.04368E-08	3.58222E-06	6.94094E-08	MFS transporter, DHA2 family, metal-tetracycline-proton antiporter
K08169	0.000436994	0.000435294	0.000427752	0.000388185	MFS transporter, DHA2 family, multidrug resistance protein
K08170	2.21306E-06	6.36511E-07	5.75164E-06	7.26682E-07	MFS transporter, DHA2 family, multidrug resistance protein
K08172	0.000437771	0.000433622	0.000403982	0.000361686	MFS transporter, MHS family, shikimate and dehydroshikimate transport protein
K08173	0.000438273	0.000436466	0.000412803	0.000432511	MFS transporter, MHS family, metabolite:H+ symporter
K08174	0	0	2.00826E-08	0	MFS transporter, FHS family, glucose/mannose:H+ symporter
K08176	0	0	2.58094E-09	5.4955E-10	MFS transporter, PHS family, inorganic phosphate transporter
K08177	0.000219585	0.000213195	0.000192509	0.000119027	MFS transporter, OFA family, oxalate/formate antiporter
K08178	0.000216375	0.000215558	0.000200239	0.000150853	MFS transporter, SHS family, lactate transporter
K08191	0.000645443	0.000637556	0.000563494	0.000397855	MFS transporter, ACS family, hexuronate transporter
K08194	0.000217939	0.000212215	0.000187562	0.000118279	MFS transporter, ACS family, D-galactonate transporter
K08195	0.000860099	0.000855699	0.000775824	0.000531677	MFS transporter, AAHS family, 4-hydroxybenzoate transporter
K08196	4.96461E-09	1.53736E-07	8.07851E-08	1.46865E-08	MFS transporter, AAHS family, cis,cis-muconate transporter
K08217	1.08131E-06	8.63497E-07	1.70153E-06	8.44575E-07	MFS transporter, DHA3 family, macrolide efflux protein
K08218	0.000221741	0.000220085	0.000219209	0.000234879	MFS transporter, PAT family, beta-lactamase induction signal transducer AmpG
K08219	2.03485E-07	5.47705E-07	3.35269E-07	3.87245E-06	MFS transporter, UMF2 family, putative MFS family transporter protein
K08221	1.65275E-06	6.20053E-07	6.29033E-06	2.08662E-06	MFS transporter, ACDE family, multidrug resistance protein
K08222	5.54378E-08	2.06813E-07	1.64992E-07	0	MFS transporter, YQGE family, putative transporter
K08223	0.000216618	0.000216675	0.000207008	0.000205229	MFS transporter, FSR family, fosmidomycin resistance protein
K08224	0.000430306	0.000429486	0.000380776	0.000302568	MFS transporter, YNFM family, putative membrane transport protein
K08225	1.33137E-06	3.52943E-06	9.2413E-07	1.14616E-05	MFS transporter, ENTS family, enterobactin (siderophore) exporter
K08226	1.94152E-06	3.68568E-08	1.98741E-07	2.62999E-07	MFS transporter, BCD family, chlorophyll transporter
K08227	0.000215265	0.000215281	0.000203404	0.00014901	MFS transporter, LPLT family, lysophospholipid transporter
K08234	3.2264E-06	4.63296E-06	2.16297E-05	0.000109414	glyoxylase I family protein
K08246	0	0	2.09314E-08	0	cycloeucalenol cycloisomerase [EC:5.5.1.9]
K08252	1.19048E-06	3.60574E-06	2.41475E-06	5.83374E-06	receptor protein-tyrosine kinase [EC:2.7.10.1]
K08253	1.12766E-07	1.51735E-07	6.15441E-08	2.21494E-07	non-specific protein-tyrosine kinase [EC:2.7.10.2]
K08255	1.61622E-06	1.65204E-06	6.24094E-06	1.49587E-05	CoA-disulfide reductase [EC:1.8.1.14]
K08256	1.09512E-06	4.13134E-06	4.40964E-06	4.00296E-06	phosphatidyl-myo-inositol alpha-mannosyltransferase [EC:2.4.1.345]
K08258	9.89199E-07	0	2.67168E-06	1.1315E-07	staphopain A [EC:3.4.22.48]
K08259	1.53616E-07	1.69465E-08	6.17137E-08	2.68656E-08	lysostaphin [EC:3.4.24.75]
K08260	0	0	8.75084E-09	2.48769E-09	adenosylcobinamide hydrolase [EC:3.5.1.90]
K08261	7.30757E-07	9.89212E-07	3.75973E-06	6.83866E-06	D-sorbitol dehydrogenase (acceptor) [EC:1.1.99.21]
K08264	1.13237E-07	2.08668E-07	5.74041E-07	1.3511E-08	heterodisulfide reductase subunit D [EC:1.8.98.1]
K08276	2.67399E-07	4.03189E-07	1.34333E-06	5.4562E-06	ecotin
K08277	4.34863E-09	1.82993E-08	3.13163E-08	1.95705E-07	transcriptional activator CaiF
K08279	1.76352E-07	1.58314E-07	2.26134E-07	1.21305E-06	carnitine operon protein CaiE
K08280	1.70497E-07	2.62734E-07	3.00759E-08	6.11862E-07	lipopolysaccharide O-acetyltransferase [EC:2.3.1.-]
K08281	0.000218183	0.000217191	0.000201853	0.000176725	nicotinamidase/pyrazinamidase [EC:3.5.1.19 3.5.1.-]
K08282	6.0606E-06	9.87861E-06	4.29754E-05	9.90061E-05	non-specific serine/threonine protein kinase [EC:2.7.11.1]
K08289	3.34816E-06	6.88859E-06	2.45599E-05	0.000103202	phosphoribosylglycinamide formyltransferase 2 [EC:2.1.2.2]
K08295	0.000219593	0.000221845	0.000231988	0.000215881	2-aminobenzoate-CoA ligase [EC:6.2.1.32]
K08296	0.000220537	0.000218834	0.000202297	0.000191901	phosphohistidine phosphatase [EC:3.1.3.-]
K08297	1.04591E-07	4.91207E-08	1.00357E-07	1.52798E-06	crotonobetainyl-CoA dehydrogenase [EC:1.3.8.13]
K08298	4.85869E-07	1.19022E-06	5.54281E-06	1.52022E-05	L-carnitine CoA-transferase [EC:2.8.3.21]
K08299	4.22183E-06	6.39951E-06	1.67504E-05	0.000111735	crotonobetainyl-CoA hydratase [EC:4.2.1.149]
K08300	0.000436059	0.000435729	0.000409516	0.000402328	ribonuclease E [EC:3.1.26.12]
K08301	0.000223529	0.000228291	0.000231613	0.000237784	ribonuclease G [EC:3.1.26.-]
K08302	2.95417E-07	5.33978E-07	6.47328E-07	4.15777E-06	tagatose 1,6-diphosphate aldolase GatY/KbaY [EC:4.1.2.40]
K08303	0.000222876	0.000223642	0.000246268	0.000224853	putative protease [EC:3.4.-.-]
K08304	0.000217911	0.000215958	0.000205443	0.000214682	membrane-bound lytic murein transglycosylase A [EC:4.2.2.-]

K08305	0.000440685	0.000434502	0.000413948	0.000427661	membrane-bound lytic murein transglycosylase B [EC:4.2.2.-]
K08306	0.000215172	0.000215074	0.000200585	0.000164674	membrane-bound lytic murein transglycosylase C [EC:4.2.2.-]
K08307	0.000871574	0.000871654	0.000814036	0.000792795	membrane-bound lytic murein transglycosylase D [EC:4.2.2.-]
K08308	2.00736E-07	6.01188E-07	1.49809E-07	2.64427E-06	membrane-bound lytic murein transglycosylase E [EC:4.2.2.-]
K08309	0.000872884	0.000866687	0.000833597	0.000815062	soluble lytic murein transglycosylase [EC:4.2.2.-]
K08310	0.000216623	0.000218693	0.000207444	0.000151679	dihydroneopterin triphosphate diphosphatase [EC:3.6.1.67]
K08311	0.000220058	0.000217564	0.000209512	0.000224266	putative (di)nucleoside polyphosphate hydrolase [EC:3.6.1.-]
K08312	1.46795E-06	2.38067E-06	5.21777E-06	7.09194E-05	ADP-ribose diphosphatase [EC:3.6.1.-]
K08313	2.10482E-07	4.30069E-07	3.10553E-06	5.23078E-06	fructose-6-phosphate aldolase 1 [EC:4.1.2.-]
K08314	1.40499E-07	4.21021E-07	3.08858E-06	3.30757E-06	fructose-6-phosphate aldolase 2 [EC:4.1.2.-]
K08315	1.27849E-07	2.41588E-07	3.07406E-07	2.13822E-06	hydrogenase 3 maturation protease [EC:3.4.23.51]
K08316	0.000223647	0.000223506	0.00022823	0.00022551	16S rRNA (guanine966-N2)-methyltransferase [EC:2.1.1.171]
K08317	9.39832E-07	3.26268E-08	2.92212E-06	1.55895E-06	uncharacterized oxidoreductase [EC:1.1.-.-]
K08318	1.33498E-08	2.26612E-08	1.21419E-07	3.35767E-07	4-hydroxybutyrate dehydrogenase / sulfolactaldehyde 3-reductase [EC:1.1.1.61 1.1.1.373]
K08319	4.77668E-06	3.70516E-06	1.53014E-05	4.75197E-05	L-threonate 2-dehydrogenase [EC:1.1.1.411]
K08320	3.74133E-07	4.93358E-07	1.1296E-06	1.3993E-05	(d)CTP diphosphatase [EC:3.6.1.65]
K08321	8.35533E-08	4.30823E-08	2.64982E-07	7.54133E-07	3-hydroxy-5-phosphonooxypentane-2,4-dione thiolase [EC:2.3.1.245]
K08322	1.03223E-06	6.01587E-07	6.42922E-06	1.71035E-06	L-gulonate 5-dehydrogenase [EC:1.1.1.380]
K08323	0.000214301	0.000212199	0.000187997	0.000118199	mannonate dehydratase [EC:4.2.1.8]
K08324	1.8319E-06	1.28284E-06	3.53167E-06	7.00325E-05	succinate-semialdehyde dehydrogenase [EC:1.2.1.16 1.2.1.24]
K08325	2.31525E-06	2.87478E-06	8.20145E-06	2.1711E-05	NADP-dependent alcohol dehydrogenase [EC:1.1.-.-]
K08326	7.11767E-08	1.74814E-07	2.90375E-07	1.98836E-06	aminopeptidase [EC:3.4.11.-]
K08344	2.2656E-06	3.76173E-06	1.53138E-05	4.72528E-05	suppressor for copper-sensitivity B
K08348	3.96591E-06	6.41223E-06	1.39258E-05	6.03137E-05	formate dehydrogenase-N, alpha subunit [EC:1.17.5.3]
K08349	3.11023E-06	4.41914E-06	1.47803E-05	3.64489E-05	formate dehydrogenase-N, beta subunit
K08350	3.77082E-07	6.88101E-07	1.53197E-07	1.52807E-06	formate dehydrogenase-N, gamma subunit
K08351	2.09482E-06	4.36579E-06	2.29217E-05	5.99785E-05	biotin/methionine sulfoxide reductase [EC:1.-.-.-]
K08352	0.000213803	0.000211912	0.000188086	0.000117796	thiosulfate reductase / polysulfide reductase chain A [EC:1.8.5.5]
K08353	4.34539E-09	1.82913E-08	6.22643E-08	2.70603E-06	thiosulfate reductase electron transport protein
K08354	4.34539E-09	1.82913E-08	3.12929E-08	2.16851E-07	thiosulfate reductase cytochrome b subunit
K08355	5.37662E-09	1.85452E-08	1.04231E-07	1.0175E-08	arsenite oxidase small subunit [EC:1.20.2.1 1.20.9.1]
K08356	5.37662E-09	1.85452E-08	8.67885E-08	1.0175E-08	arsenite oxidase large subunit [EC:1.20.2.1 1.20.9.1]
K08357	0.000215103	0.000215068	0.000202012	0.000156853	tetrathionate reductase subunit A
K08358	1.36294E-06	3.26563E-06	1.43989E-05	4.31483E-05	tetrathionate reductase subunit B
K08359	1.31706E-06	3.23738E-06	1.41505E-05	4.0665E-05	tetrathionate reductase subunit C
K08363	1.80598E-06	2.41414E-06	1.07147E-05	4.22956E-05	mercuric ion transport protein
K08364	3.33257E-06	2.7765E-06	1.37047E-05	4.22129E-05	periplasmic mercuric ion binding protein
K08365	0.000219948	0.000217979	0.000209702	0.000227561	MerR family transcriptional regulator, mercuric resistance operon regulatory protein
K08368	0.000215583	0.000212343	0.000194357	0.000115201	MFS transporter, putative metabolite transport protein
K08369	0.000226599	0.000225793	0.00022432	0.000245355	MFS transporter, putative metabolite:H+ symporter
K08372	0.000224701	0.000224951	0.00023307	0.000232549	putative serine protease PepD [EC:3.4.21.-]
K08384	3.67959E-06	2.12473E-06	3.53957E-06	2.23089E-06	stage V sporulation protein D (sporulation-specific penicillin-binding protein)
K08475	2.0999E-07	2.32941E-07	8.55184E-07	1.99337E-05	two-component system, NtrC family, phosphoglycerate transport system sensor histidine kinase PgtB [EC:2.7.13.3]
K08476	2.85394E-06	6.02695E-06	3.17788E-05	7.88159E-05	two-component system, NtrC family, phosphoglycerate transport system response regulator PgtA
K08477	7.85964E-11	4.40377E-09	8.44119E-09	2.79138E-08	outer membrane protease E [EC:3.4.21.-]
K08478	1.2276E-06	2.93054E-06	1.38576E-05	4.14253E-05	phosphoglycerate transport regulatory protein PgtC
K08479	1.41087E-06	1.29759E-06	2.83844E-06	5.44727E-06	two-component system, OmpR family, clock-associated histidine kinase SasA [EC:2.7.13.3]
K08481	1.47934E-07	1.00871E-07	2.50669E-07	1.92394E-07	circadian clock protein KaiB
K08482	4.45762E-06	3.15534E-06	4.7893E-06	1.88138E-05	circadian clock protein KaiC
K08483	0.001082099	0.001075394	0.001014281	0.000876901	phosphotransferase system, enzyme I, PtsI [EC:2.7.3.9]
K08484	0.000216402	0.000213104	0.00019184	0.000132043	phosphotransferase system, enzyme I, PtsP [EC:2.7.3.9]
K08485	3.00521E-07	3.61321E-07	4.11086E-07	1.71954E-05	phosphocarrier protein NPr
K08566	3.33038E-09	1.67914E-08	2.15315E-08	6.393E-08	plasminogen activator [EC:3.4.23.48]
K08589	0	0	0	1.79879E-08	gingipain R [EC:3.4.22.37]
K08591	0.000219822	0.000217014	0.000220322	0.000169625	glycerol-3-phosphate acyltransferase PlsY [EC:2.3.1.15]
K08598	2.51588E-09	9.57646E-09	1.58692E-06	1.61653E-07	YopJ protease family
K08599	0	0	9.17879E-09	3.30288E-08	YopT peptidase [EC:3.4.22.-]
K08600	1.23332E-06	7.70131E-07	1.06772E-05	2.47384E-06	sortase B [EC:3.4.22.70]
K08602	5.54184E-06	1.54235E-06	1.17512E-05	6.26943E-05	oligoendopeptidase F [EC:3.4.24.-]
K08604	6.59838E-07	2.39315E-06	2.82144E-06	6.0211E-05	vibriolysin [EC:3.4.24.25]
K08605	0	0	0	4.44141E-07	coccolysin [EC:3.4.24.30]
K08640	3.04764E-07	5.45527E-07	2.09962E-07	3.68999E-08	zinc D-Ala-D-Ala carboxypeptidase [EC:3.4.17.14]
K08641	1.57162E-06	3.16953E-06	3.75188E-06	5.26581E-05	zinc D-Ala-D-Ala dipeptidase [EC:3.4.13.22]
K08642	5.53664E-10	8.20565E-09	3.49273E-07	8.75978E-06	LasA protease [EC:3.4.24.-]
K08643	1.69248E-07	7.97654E-08	2.68273E-07	2.73328E-06	zinc metalloprotease ZmpB [EC:3.4.24.-]
K08646	9.96501E-08	3.14198E-08	5.73435E-07	4.47479E-06	peptidyl-Lys metalloendopeptidase [EC:3.4.24.20]
K08651	1.22933E-06	3.04693E-06	2.09805E-06	1.19954E-05	thermitase [EC:3.4.21.66]
K08652	3.58239E-08	0	6.20919E-08	8.99721E-07	C5a peptidase [EC:3.4.21.110]
K08659	1.35459E-06	2.82441E-07	8.65795E-06	1.12232E-05	dipeptidase [EC:3.4.-.-]
K08675	0	1.18379E-10	1.03204E-09	0	ATP-dependent Lon protease [EC:3.4.21.53]
K08676	1.5587E-06	2.9372E-06	1.12053E-05	4.94931E-06	tricorn protease [EC:3.4.21.-]
K08677	1.31243E-07	2.72043E-07	3.87435E-07	7.86071E-08	kumamolisin
K08678	1.19886E-06	3.00008E-06	5.27894E-06	1.36063E-05	UDP-glucuronate decarboxylase [EC:4.1.1.35]
K08679	4.50342E-06	4.35459E-06	5.19556E-06	6.59708E-05	UDP-glucuronate 4-epimerase [EC:5.1.3.6]
K08680	4.11565E-06	4.73236E-06	2.20203E-05	9.83153E-05	2-succinyl-6-hydroxy-2,4-cyclohexadiene-1-carboxylate synthase [EC:4.2.99.20]
K08681	2.9001E-06	4.11539E-06	1.26708E-05	4.73589E-06	5-phosphate synthase pdxT subunit [EC:4.3.3.6]
K08682	2.7689E-07	7.56445E-07	1.26566E-06	9.42631E-06	acyl carrier protein phosphodiesterase [EC:3.1.4.14]
K08685	1.24208E-08	2.67444E-09	2.93506E-08	1.68705E-08	quinohemoprotein amine dehydrogenase [EC:1.4.9.1]
K08687	1.48149E-08	3.14964E-09	6.45236E-10	1.58498E-08	N-carbamoylsarcosine amidase [EC:3.5.1.59]
K08688	9.33273E-07	6.41845E-08	8.66507E-07	8.67377E-08	creatinase [EC:3.5.3.3]
K08689	2.60732E-07	6.01634E-07	3.29702E-06	6.96236E-06	biphenyl 2,3-dioxygenase subunit alpha [EC:1.14.12.18]
K08690	3.04097E-07	6.29844E-07	3.21454E-06	7.73576E-06	cis-2,3-dihydrobiphenyl-2,3-diol dehydrogenase [EC:1.3.1.56]
K08691	1.97793E-06	2.57744E-07	4.51922E-07	3.05436E-07	malyl-CoA/(S)-citramalyl-CoA lyase [EC:4.1.3.24 4.1.3.25]
K08692	1.93397E-06	1.90329E-06	4.51378E-06	5.53977E-05	malate-CoA ligase subunit alpha [EC:6.2.1.9]
K08693	2.97788E-07	8.10034E-08	9.35456E-07	5.92625E-06	2,3-cyclic-nucleotide 2-phosphodiesterase / 3-nucleotidase / 5-nucleotidase [EC:3.1.4.16 3.1.3.6 3.1.3.5]
K08696	2.45193E-08	1.35127E-09	2.86255E-07	1.85757E-06	carbon dioxide concentrating mechanism protein CcmK
K08697	2.06612E-08	0	7.50671E-08	3.78957E-11	carbon dioxide concentrating mechanism protein CcmL

K08698	1.25345E-06	3.1244E-06	1.38122E-05	3.47122E-05	carbon dioxide concentrating mechanism protein CcmM
K08700	4.05785E-07	3.08105E-07	7.71556E-07	4.55207E-07	carbon dioxide concentrating mechanism protein CcmO
K08710	4.92514E-09	9.19807E-10	2.73276E-09	4.98775E-09	N-isopropylammelide isopropylaminohydrolase [EC:3.5.4.42]
K08713	8.74059E-08	2.06813E-07	5.78358E-07	1.90174E-09	potassium channel LctB
K08714	8.31334E-08	5.85871E-08	1.55272E-06	3.7413E-06	voltage-gated sodium channel
K08715	3.35827E-08	8.48734E-08	2.36619E-07	7.4393E-07	inward rectifier potassium channel
K08717	2.07793E-06	3.72346E-06	6.54227E-06	1.93826E-06	urea transporter
K08720	0.000857083	0.0008517	0.000767204	0.00050574	outer membrane protein OmpU
K08721	3.68708E-06	7.38466E-06	3.47807E-05	0.000133099	outer membrane protein, multidrug efflux system
K08722	5.60969E-07	5.22406E-07	9.44993E-07	1.38436E-05	5-deoxynucleotidase [EC:3.1.3.89]
K08723	1.09432E-07	1.38878E-07	5.69566E-07	1.7037E-05	5-nucleotidase [EC:3.1.3.5]
K08724	8.01395E-07	4.72982E-07	4.63885E-06	2.83391E-06	penicillin-binding protein 2B
K08728	1.28086E-07	3.45462E-08	5.5177E-07	3.18695E-08	nucleoside deoxyribosyltransferase [EC:2.4.2.6]
K08738	0.000224262	0.000220421	0.000225937	0.000236979	cytochrome c
K08744	0.000222446	0.000222197	0.000217128	0.000155096	cardiolipin synthase (CMP-forming) [EC:2.7.8.41]
K08776	3.38233E-07	2.14261E-07	1.02616E-06	2.7042E-06	puromycin-sensitive aminopeptidase [EC:3.4.11.-]
K08777	8.28047E-08	7.92523E-07	2.25646E-07	8.98935E-08	neutral peptidase B [EC:3.4.24.-]
K08884	1.1422E-06	2.78671E-06	6.64601E-06	7.05153E-06	serine/threonine protein kinase, bacterial [EC:2.7.11.1]
K08902	0	0	0	1.52139E-08	photosystem II Psb27 protein
K08906	0	0	1.5247E-07	0	cytochrome c6
K08926	9.7076E-07	1.11305E-08	8.07648E-08	1.22569E-07	light-harvesting complex 1 alpha chain
K08927	9.7076E-07	1.11305E-08	8.07648E-08	1.22569E-07	light-harvesting complex 1 beta chain
K08928	9.7076E-07	1.11305E-08	8.07648E-08	1.22569E-07	photosynthetic reaction center L subunit
K08929	9.7076E-07	1.11305E-08	8.07648E-08	1.37783E-07	photosynthetic reaction center M subunit
K08930	0	4.02294E-08	3.72114E-08	1.78604E-08	light-harvesting protein B-800-850 alpha chain
K08939	0	4.11492E-08	3.72114E-08	1.78604E-08	light-harvesting protein B-800-850 beta chain
K08952	0	0	1.71141E-06	4.69895E-08	chlorosome envelope protein I
K08961	0	4.95613E-07	2.14281E-07	2.67225E-08	chondroitin-sulfate-ABC endolyase/exolyase [EC:4.2.2.20 4.2.2.21]
K08963	3.27838E-06	2.11755E-06	4.96795E-06	5.49045E-05	methylthioribose-1-phosphate isomerase [EC:5.3.1.23]
K08964	1.37473E-06	1.74018E-06	2.70566E-06	5.47994E-05	methylthioribulose-1-phosphate dehydratase [EC:4.2.1.109]
K08965	6.44218E-08	2.07733E-07	6.01081E-07	7.25985E-09	2,3-diketo-5-methylthiopentyl-1-phosphate enolase [EC:5.3.2.5]
K08966	6.44218E-08	2.06898E-07	7.80725E-07	1.18999E-08	2-hydroxy-3-keto-5-methylthiopentenyl-1-phosphate phosphatase [EC:3.1.3.87]
K08967	2.57513E-06	5.11307E-06	1.73558E-05	8.92494E-05	1,2-dihydroxy-3-keto-5-methylthiopentene dioxygenase [EC:1.13.11.53 1.13.11.54]
K08968	3.35274E-06	3.61062E-06	1.15102E-05	7.29034E-05	L-methionine (R)-S-oxide reductase [EC:1.8.4.14]
K08969	3.81746E-06	6.7496E-07	8.17077E-06	1.63769E-06	aminotransferase [EC:2.6.1.-]
K08970	0.000217053	0.000212355	0.000187029	0.000114543	nickel/cobalt exporter
K08972	0.000215739	0.000216627	0.00020676	0.000153059	putative membrane protein
K08973	0.000219216	0.000218227	0.000207496	0.0002035	putative membrane protein
K08974	1.6634E-06	1.68608E-06	9.10926E-06	1.26422E-05	putative membrane protein
K08976	1.13575E-06	9.24422E-07	3.59154E-06	6.13188E-08	putative membrane protein
K08977	3.97169E-09	0	0	0	bisanhydrobacterioruberin hydratase [EC:4.2.1.161]
K08978	2.50124E-06	9.30489E-07	1.70332E-06	1.61259E-05	bacterial/archaeal transporter family protein
K08981	4.0732E-06	8.39255E-06	1.0618E-05	6.63393E-05	putative membrane protein
K08982	3.9001E-07	3.51734E-07	1.8511E-06	1.57388E-06	putative membrane protein
K08983	0.000215015	0.000215224	0.000204136	0.000151199	putative membrane protein
K08984	1.21187E-06	1.16512E-06	1.90978E-06	4.39278E-05	putative membrane protein
K08985	1.58324E-07	4.50752E-07	1.04686E-06	8.53488E-06	putative lipoprotein
K08986	4.30722E-07	3.23822E-07	5.29321E-07	3.443E-06	putative membrane protein
K08987	0.000216712	0.000216713	0.000205277	0.000207529	putative membrane protein
K08988	1.39353E-06	5.87657E-07	1.00553E-06	3.44249E-06	putative membrane protein
K08989	0.000216002	0.000212484	0.000187926	0.000114009	putative membrane protein
K08990	1.39366E-06	4.52215E-07	5.68096E-07	1.71917E-05	putative membrane protein
K08992	0.000216366	0.000215839	0.000204956	0.000217061	lipopolysaccharide assembly protein A
K08993	2.53557E-07	3.18122E-07	1.61076E-06	1.38708E-05	putative membrane protein
K08994	0.000220504	0.000219655	0.000216715	0.000239844	ion channel-forming bestrophin family protein
K08995	1.85715E-06	2.20741E-06	1.09008E-05	1.82428E-05	putative membrane protein
K08996	2.42681E-06	2.45249E-07	7.87841E-06	2.25775E-06	putative membrane protein
K08997	0.000221027	0.000221086	0.000210359	0.000222052	uncharacterized protein
K08998	0.000222131	0.00022163	0.000220617	0.000171999	uncharacterized protein
K08999	1.99088E-06	5.63946E-06	1.04131E-05	3.88038E-06	uncharacterized protein
K09000	4.22178E-07	4.17205E-08	3.23244E-07	4.70283E-07	CRISPR-associated protein Cmr4
K09001	0.000219178	0.000217863	0.000207894	0.00021842	anhydro-N-acetylmuramic acid kinase [EC:2.7.1.170]
K09002	8.29062E-07	1.24033E-07	5.52094E-06	1.23757E-07	CRISPR-associated protein Csm3
K09003	3.96848E-08	1.14307E-07	4.75446E-07	2.53843E-08	uncharacterized protein
K09004	0.000215461	0.000211872	0.000186576	0.000114275	uncharacterized protein
K09005	0.000432339	0.000430287	0.000398271	0.000331665	uncharacterized protein
K09007	0.000216516	0.000216098	0.00020511	0.000151681	GTP cyclohydrolase IB [EC:3.5.4.16]
K09009	1.55067E-06	4.09786E-06	9.46983E-06	4.38106E-06	uncharacterized protein
K09010	0	0	4.18629E-08	0	uncharacterized protein
K09011	0.000214105	0.000212563	0.00018704	0.000113417	(R)-citramalate synthase [EC:2.3.1.182]
K09012	0	1.17924E-09	6.97714E-09	1.04807E-08	DeoR family transcriptional regulator, suf operon transcriptional repressor
K09013	1.26964E-05	1.84733E-05	6.16748E-05	0.000112962	Fe-S cluster assembly ATP-binding protein
K09014	6.4531E-06	8.0158E-06	2.23833E-05	6.17632E-05	Fe-S cluster assembly protein SufB
K09015	6.37244E-06	8.60521E-06	2.23337E-05	6.0965E-05	Fe-S cluster assembly protein SufD
K09016	4.61303E-06	7.28834E-06	2.62921E-05	4.11104E-05	putative pyrimidine permease RutG
K09017	0.00021845	0.000219337	0.00021447	0.000167656	TetR/AcrR family transcriptional regulator
K09018	1.87568E-06	5.21789E-07	8.07123E-07	2.43492E-06	pyrimidine oxygenase [EC:1.14.99.46]
K09019	0.000217976	0.000218401	0.000200977	0.000195207	3-hydroxypropanoate dehydrogenase [EC:1.1.1.-]
K09020	1.71602E-06	2.65982E-07	1.37714E-06	7.30477E-07	ureidoacrylate peracid hydrolase [EC:3.5.1.110]
K09021	1.61861E-06	1.12937E-07	8.96374E-07	7.15837E-07	aminoacrylate peracid reductase
K09022	0.000442252	0.00044297	0.000455454	0.000512619	2-iminobutanoate/2-iminopropanoate deaminase [EC:3.5.99.10]
K09023	1.83033E-06	9.55791E-08	8.38015E-07	2.48362E-06	aminoacrylate hydrolase [EC:3.5.1.-]
K09024	3.8843E-06	1.08628E-06	2.69485E-06	3.90551E-06	flavin reductase [EC:1.5.1.-]
K09065	1.06533E-06	1.25518E-06	1.7537E-06	5.20706E-05	N-acetylornithine carbamoyltransferase [EC:2.1.3.9]
K09116	9.35864E-11	7.12066E-08	4.37354E-09	3.90279E-09	uncharacterized protein
K09117	0.000219507	0.000219431	0.000212678	0.000219153	uncharacterized protein
K09118	9.77179E-07	3.88625E-06	4.04554E-06	4.21451E-06	uncharacterized protein

K09120	0	0	0	8.03716E-09	uncharacterized protein
K09121	2.61262E-06	5.2058E-07	3.94295E-06	3.68658E-07	pyridinium-3,5-bisthiocarboxylic acid mononucleotide nickel chelatase [EC:4.99.1.12]
K09122	0.000214	0.00021186	0.00018687	0.000113394	uncharacterized protein
K09123	1.68638E-07	2.34871E-07	3.81036E-07	1.33881E-07	cis-L-3-hydroxyproline dehydratase [EC:4.2.1.171]
K09124	1.09976E-07	7.87222E-08	3.71364E-07	2.34039E-06	uncharacterized protein
K09125	6.61888E-06	9.43724E-06	3.64607E-05	0.000153858	uncharacterized protein
K09126	1.78726E-08	0	0	0	uncharacterized protein
K09127	4.22178E-07	3.06602E-09	4.22756E-07	4.79791E-07	CRISPR-associated protein Cmr3
K09128	3.30248E-07	2.15522E-07	4.95781E-07	8.24228E-08	uncharacterized protein
K09129	0.000213805	0.000211837	0.000186287	0.000113202	uncharacterized protein
K09131	2.02905E-06	8.40283E-07	2.74776E-06	1.41286E-05	uncharacterized protein
K09133	0	0	4.37253E-07	0	uncharacterized protein
K09136	0.000216756	0.000216225	0.000208859	0.000167952	ribosomal protein S12 methylthiotransferase accessory factor
K09137	4.8197E-08	9.39306E-09	3.06569E-07	4.89869E-08	uncharacterized protein
K09138	2.04795E-08	2.89397E-08	5.12541E-08	6.53358E-08	uncharacterized protein
K09140	0	0	0	2.67905E-09	pre-rRNA-processing protein TSR3
K09141	1.18375E-07	7.19057E-08	2.98684E-06	1.1741E-06	uncharacterized protein
K09143	0	9.82517E-09	4.80677E-08	4.60393E-08	uncharacterized protein
K09144	1.57793E-06	1.43984E-06	7.39081E-06	1.65486E-05	uncharacterized protein
K09145	9.68623E-07	9.5208E-10	3.32674E-06	3.40504E-08	uncharacterized protein
K09146	0	5.71015E-07	1.39596E-06	1.75726E-06	uncharacterized protein
K09150	4.46815E-09	2.4421E-08	0	0	uncharacterized protein
K09153	1.0077E-06	2.8111E-06	1.4192E-05	3.1642E-05	uncharacterized protein
K09154	9.87446E-07	4.21631E-08	6.3322E-08	1.33207E-07	uncharacterized protein
K09155	6.86214E-07	4.11904E-07	3.88154E-06	2.7925E-06	uncharacterized protein
K09157	1.24473E-06	1.09366E-06	6.4863E-06	3.15112E-06	uncharacterized protein
K09158	0.000217642	0.000217682	0.000207381	0.000217496	uncharacterized protein
K09159	0.000219353	0.000217124	0.000207563	0.00021764	antitoxin CptB
K09160	3.09335E-06	9.59671E-07	2.8219E-06	1.73545E-05	uncharacterized protein
K09161	1.70208E-07	3.79868E-07	5.97292E-07	1.25366E-05	uncharacterized protein
K09162	0	1.01891E-08	6.37674E-08	5.35811E-09	chlorite dismutase [EC:1.13.11.49]
K09163	2.23408E-08	1.28698E-07	4.78151E-07	8.55783E-09	uncharacterized protein
K09164	1.18348E-06	1.44267E-06	3.1358E-06	6.09094E-05	uncharacterized protein
K09165	0.000216451	0.000213546	0.000189888	0.000147637	uncharacterized protein
K09166	1.57345E-07	7.75857E-07	8.82253E-07	1.97213E-06	uncharacterized protein
K09167	4.11026E-06	8.76399E-06	1.0941E-05	6.0771E-05	uncharacterized protein
K09181	0.000221271	0.000223184	0.000223661	0.000305314	acetyltransferase
K09190	7.19116E-07	1.39886E-06	6.53864E-07	8.88491E-09	uncharacterized protein
K09251	0.000643513	0.000641162	0.000575356	0.000374114	putrescine aminotransferase [EC:2.6.1.82]
K09384	4.61737E-06	6.40559E-06	2.34453E-05	3.62148E-05	uncharacterized protein
K09386	0.00021529	0.000215141	0.000201293	0.000150195	uncharacterized protein
K09388	1.14351E-07	2.06813E-07	5.47809E-07	0	uncharacterized protein
K09456	0.000219314	0.000217811	0.000207496	0.000206149	putative acyl-CoA dehydrogenase
K09457	3.57371E-06	2.3346E-06	8.97039E-06	1.37987E-06	7-cyano-7-deazaguanine reductase [EC:1.7.1.13]
K09458	0.00109318	0.001086146	0.001030867	0.000966285	3-oxoacyl-[acyl-carrier-protein] synthase II [EC:2.3.1.179]
K09459	3.28594E-08	9.08649E-08	8.96677E-07	8.43221E-08	phosphonopyruvate decarboxylase [EC:4.1.1.82]
K09461	0.001508778	0.001494878	0.001344228	0.000958884	anthraniloyl-CoA monooxygenase [EC:1.14.13.40]
K09469	0	1.02051E-08	3.69959E-09	0	2-aminoethylphosphonate-pyruvate transaminase
K09470	2.54531E-07	9.03392E-07	8.58854E-07	3.80665E-06	gamma-glutamylputrescine synthase [EC:6.3.1.11]
K09471	0.000647944	0.000642014	0.000589851	0.000463785	gamma-glutamylputrescine oxidase [EC:1.4.3.-]
K09472	0.000870342	0.000868937	0.000817796	0.000739837	4-(gamma-glutamylamino)butanal dehydrogenase [EC:1.2.1.99]
K09473	1.26728E-07	6.87119E-07	3.28096E-07	5.37776E-06	gamma-glutamyl-gamma-aminobutyrate hydrolase [EC:3.5.1.94]
K09474	4.67855E-06	1.44427E-06	2.45972E-06	5.23042E-05	acid phosphatase (class A) [EC:3.1.3.2]
K09475	4.8869E-07	1.49343E-06	4.07802E-07	4.01654E-06	outer membrane pore protein C
K09476	4.91854E-07	1.50458E-06	4.83879E-07	6.57337E-06	outer membrane pore protein F
K09477	2.49656E-07	3.02543E-07	7.24027E-08	7.50643E-07	citrate:succinate antiporter
K09478	2.70408E-08	0	0	0	short/branched chain acyl-CoA dehydrogenase [EC:1.3.99.12]
K09482	1.62054E-11	4.35369E-07	1.33993E-06	5.07141E-08	glutamyl-tRNA(Gln) amidotransferase subunit D [EC:6.3.5.7]
K09483	2.69061E-07	1.69159E-07	7.62831E-07	2.24453E-05	3-dehydroshikimate dehydratase [EC:4.2.1.118]
K09516	1.17062E-06	9.88341E-07	3.92127E-06	5.17277E-05	all-trans-retinol 13,14-reductase [EC:1.3.99.23]
K09568	8.99101E-09	1.39302E-08	1.56986E-08	0	FK506-binding protein 1 [EC:5.2.1.8]
K09607	3.25519E-07	5.09074E-07	3.32565E-06	1.0772E-05	immune inhibitor A [EC:3.4.24.-]
K09612	1.08859E-07	4.20193E-07	2.65859E-07	8.21891E-07	alkaline phosphatase isozyme conversion protein [EC:3.4.11.-]
K09667	2.39918E-06	5.3622E-06	2.58432E-05	6.26004E-05	protein O-GlcNAc transferase [EC:2.4.1.255]
K09680	1.61531E-06	5.17093E-07	3.45663E-06	5.20316E-08	type II pantothenate kinase [EC:2.7.1.33]
K09681	7.48041E-06	4.51715E-06	2.62356E-05	4.77528E-05	LysR family transcriptional regulator, transcription activator of glutamate synthase operon
K09682	9.08815E-08	2.07765E-07	5.65699E-07	1.90174E-09	MarR family transcriptional regulator, protease production regulatory protein HPr
K09683	0	5.9505E-09	5.49288E-08	0	hut operon positive regulatory protein
K09684	0.000215589	0.000216084	0.000198736	0.000138845	purine catabolism regulatory protein
K09685	1.42635E-06	3.58425E-07	3.99737E-06	3.67041E-06	purine operon repressor
K09688	1.75222E-06	1.13078E-07	8.86282E-07	1.47395E-06	capsular polysaccharide transport system permease protein
K09689	8.82315E-06	3.39333E-06	2.57471E-05	1.36181E-05	capsular polysaccharide transport system ATP-binding protein [EC:3.6.3.38]
K09690	5.30252E-06	7.36469E-06	3.19614E-05	0.00012102	lipopolysaccharide transport system permease protein
K09691	1.92245E-05	2.62183E-05	7.88529E-05	0.000230449	lipopolysaccharide transport system ATP-binding protein
K09692	3.18096E-06	3.69198E-06	1.25184E-05	4.96481E-06	teichoic acid transport system permease protein
K09693	6.33282E-06	8.65732E-06	3.88228E-05	8.4029E-05	teichoic acid transport system ATP-binding protein [EC:3.6.3.40]
K09694	0.000214955	0.000216171	0.000191746	0.000124468	lipooligosaccharide transport system permease protein
K09695	0.000216772	0.000218775	0.000198173	0.000143491	lipooligosaccharide transport system ATP-binding protein
K09696	1.339E-06	1.33651E-06	1.95091E-06	5.63329E-05	sodium transport system permease protein
K09697	0.000216742	0.000214452	0.000191714	0.000170173	sodium transport system ATP-binding protein [EC:3.6.3.7]
K09698	2.72951E-06	2.31465E-06	1.55624E-05	1.35617E-05	nondiscriminating glutamyl-tRNA synthetase [EC:6.1.1.24]
K09699	2.62854E-06	3.49042E-06	6.12206E-06	7.23928E-06	2-oxoisovalerate dehydrogenase E2 component (dihydrolipoyl transacylase) [EC:2.3.1.168]
K09700	3.42985E-08	5.6191E-08	2.07021E-07	2.35528E-07	uncharacterized protein
K09701	3.58989E-06	4.0812E-06	2.06371E-05	0.000104807	uncharacterized protein
K09702	4.09671E-07	7.13067E-07	3.78866E-06	5.94425E-06	uncharacterized protein
K09703	9.39625E-08	7.81683E-07	1.21237E-06	5.15003E-06	uncharacterized protein
K09704	7.73991E-07	4.7537E-07	6.79494E-06	3.25512E-06	uncharacterized protein

K09705	0.000217339	0.000212129	0.000191181	0.000115793	uncharacterized protein
K09706	4.8488E-07	4.00966E-07	3.70329E-07	1.72587E-08	uncharacterized protein
K09707	2.49738E-07	1.65696E-07	4.83112E-06	4.80216E-06	uncharacterized protein
K09709	2.80071E-06	3.88177E-06	2.15676E-05	3.60841E-05	3-methylfumaryl-CoA hydratase [EC:4.2.1.153]
K09710	0.000223492	0.000223735	0.000227454	0.000225369	ribosome-associated protein
K09712	0.000216965	0.000215449	0.000201351	0.000187199	uncharacterized protein
K09717	0	0	1.99347E-09	0	uncharacterized protein
K09726	4.63495E-08	5.48429E-08	1.64045E-07	5.13643E-09	uncharacterized protein
K09729	0	3.86545E-08	1.74416E-07	1.01746E-08	uncharacterized protein
K09732	0	0	3.56514E-08	2.1982E-09	uncharacterized protein
K09733	0	0	4.19781E-08	2.46069E-08	(5-formylfuran-3-yl)methyl phosphate synthase [EC:4.2.3.153]
K09740	2.9469E-08	3.9023E-08	2.62674E-07	5.0875E-09	uncharacterized protein
K09744	0	0	2.09314E-08	8.03716E-09	uncharacterized protein
K09747	0.000221429	0.00021971	0.000218018	0.000223295	uncharacterized protein
K09748	0.000223244	0.000223487	0.000226422	0.00022348	ribosome maturation factor RimP
K09749	5.12866E-07	9.16038E-07	1.21224E-06	1.94898E-05	uncharacterized protein
K09758	1.5854E-06	3.76543E-06	1.44179E-05	3.53198E-05	aspartate 4-decarboxylase [EC:4.1.1.12]
K09759	3.7552E-07	2.87941E-07	3.37978E-07	8.25549E-08	nondiscriminating aspartyl-tRNA synthetase [EC:6.1.1.23]
K09760	0.000433314	0.000434981	0.00040892	0.000337579	DNA recombination protein RmuC
K09761	0.00022335	0.000224162	0.000229079	0.000226347	16S rRNA (uracil1498-N3)-methyltransferase [EC:2.1.1.193]
K09762	3.76289E-06	5.05705E-06	1.7267E-05	7.76382E-06	uncharacterized protein
K09763	1.04068E-06	3.70675E-07	3.75076E-06	1.86139E-06	uncharacterized protein
K09764	2.74384E-07	2.82893E-07	1.37686E-06	1.90343E-06	uncharacterized protein
K09765	3.48807E-06	2.48958E-06	8.78078E-06	5.99121E-05	epoxyqueuosine reductase [EC:1.17.99.6]
K09766	1.18767E-07	3.00823E-07	4.47718E-07	1.10329E-08	uncharacterized protein
K09767	0.00021868	0.000220527	0.000209553	0.00022195	cyclic-di-GMP-binding protein
K09768	5.95207E-06	2.48806E-06	8.40837E-06	6.96024E-05	uncharacterized protein
K09769	3.38796E-06	8.13719E-07	4.93244E-06	9.03431E-07	uncharacterized protein
K09770	2.43577E-07	3.01775E-07	5.82796E-07	2.9957E-09	uncharacterized protein
K09771	0.000217926	0.000215198	0.000193883	0.000172503	small multidrug resistance family-3 protein
K09772	3.66549E-06	4.92043E-06	1.7635E-05	7.77989E-06	cell division inhibitor SepF
K09773	0.000222297	0.000217969	0.000220124	0.000214512	[pyruvate, water dikinase]-phosphate phosphotransferase / [pyruvate, water dikinase] kinase [EC:2.7.4.28 2.7.11.33]
K09774	0.000219374	0.00021712	0.000207884	0.000217626	lipopolysaccharide export system protein LptA
K09775	1.47791E-06	1.76449E-06	1.01688E-05	1.47624E-05	uncharacterized protein
K09776	5.54378E-08	8.29262E-08	1.95885E-07	1.73471E-08	uncharacterized protein
K09777	9.21192E-07	4.55187E-07	1.26736E-06	2.23341E-08	uncharacterized protein
K09778	1.82183E-06	3.77763E-07	5.98786E-07	1.72749E-07	uncharacterized protein
K09779	1.16703E-06	1.34755E-06	1.98565E-06	6.48226E-07	uncharacterized protein
K09780	3.42612E-06	2.58573E-06	1.72614E-05	7.29222E-05	uncharacterized protein
K09781	5.79235E-06	8.87462E-06	2.30155E-05	0.000107521	uncharacterized protein
K09785	0	0	3.13971E-08	0	uncharacterized protein
K09786	2.66328E-06	4.14111E-06	1.66314E-05	5.20756E-05	uncharacterized protein
K09787	2.21222E-06	8.4589E-07	7.81515E-06	3.71916E-06	uncharacterized protein
K09788	0.000218465	0.000216862	0.000205937	0.000216388	2-methylaconitate isomerase [EC:5.3.3.-]
K09789	1.71441E-07	7.76117E-07	2.30236E-06	2.71345E-08	pimeloyl-[acyl-carrier protein] methyl ester esterase [EC:3.1.1.85]
K09790	4.34311E-06	6.45887E-06	2.64268E-05	0.000153336	uncharacterized protein
K09791	0.000218841	0.000218117	0.000215587	0.000219595	uncharacterized protein
K09792	0.000216989	0.000216669	0.00020494	0.000164816	uncharacterized protein
K09793	3.28013E-06	5.17989E-06	7.17197E-06	5.3594E-05	uncharacterized protein
K09794	1.56735E-06	3.46295E-07	3.53114E-06	1.33014E-05	uncharacterized protein
K09795	1.81691E-06	1.67499E-07	3.54317E-07	2.1326E-07	uncharacterized protein
K09796	0.00022206	0.000224664	0.000229652	0.000269717	periplasmic copper chaperone A
K09797	1.3948E-06	5.38303E-07	3.06289E-06	1.24035E-05	uncharacterized protein
K09798	2.03313E-06	8.6132E-07	7.9546E-07	6.19782E-06	uncharacterized protein
K09799	1.39583E-06	1.51434E-07	1.26312E-06	1.81968E-06	uncharacterized protein
K09800	0.000219369	0.000217221	0.000207402	0.000217293	translocation and assembly module TamB
K09801	0.000217663	0.000216946	0.000206988	0.000217549	uncharacterized protein
K09802	9.81891E-08	3.14525E-07	5.20228E-07	1.18622E-05	uncharacterized protein
K09803	5.13636E-06	4.90972E-07	3.47746E-06	1.80021E-06	uncharacterized protein
K09804	3.31529E-08	0	1.02995E-07	8.03716E-09	uncharacterized protein
K09805	2.79042E-10	7.77503E-10	3.41377E-08	9.94597E-07	uncharacterized protein
K09806	0.000216979	0.000214512	0.000190674	0.000182776	uncharacterized protein
K09807	4.02528E-06	3.55984E-06	8.20159E-06	0.000129151	uncharacterized protein
K09808	0.000220679	0.000222593	0.000216146	0.000235842	lipoprotein-releasing system permease protein
K09809	8.39433E-06	8.72442E-06	3.83702E-05	4.96108E-05	CDP-glycerol glycerophosphotransferase [EC:2.7.8.12]
K09810	0.000681719	0.000688268	0.000748642	0.000908454	lipoprotein-releasing system ATP-binding protein [EC:3.6.3.-]
K09811	5.28303E-06	5.62195E-06	2.37423E-05	7.72639E-05	cell division transport system permease protein
K09812	2.4781E-05	3.35654E-05	0.000106455	0.000298836	cell division transport system ATP-binding protein
K09813	9.57571E-07	0	2.74951E-06	3.03626E-08	hemin transport system permease protein
K09814	1.04031E-06	3.68287E-07	3.04779E-06	7.62493E-08	hemin transport system ATP-binding protein [EC:3.6.3.-]
K09815	4.96482E-06	3.72536E-06	2.21813E-05	2.46447E-05	zinc transport system substrate-binding protein
K09816	3.75233E-06	3.11935E-06	1.66909E-05	1.92704E-05	zinc transport system permease protein
K09817	0.000234098	0.000235997	0.000249251	0.000297812	zinc transport system ATP-binding protein [EC:3.6.3.-]
K09818	4.96461E-08	7.8616E-10	3.34419E-07	7.56495E-08	manganese/iron transport system substrate-binding protein
K09819	1.93015E-06	3.51808E-06	7.93541E-06	5.74102E-06	manganese/iron transport system permease protein
K09820	0.000215359	0.000213633	0.000201049	0.000119614	manganese/iron transport system ATP-binding protein
K09822	1.14123E-06	1.9549E-07	5.7782E-06	4.13293E-06	uncharacterized protein
K09823	5.57367E-06	5.2759E-06	1.85425E-05	0.00010389	Fur family transcriptional regulator, zinc uptake regulator
K09824	3.06018E-07	4.24451E-07	9.07438E-07	9.65041E-06	uncharacterized protein
K09825	2.08105E-06	1.5804E-06	6.90449E-06	3.44122E-06	Fur family transcriptional regulator, peroxide stress response regulator
K09826	2.18886E-06	3.1016E-06	7.2674E-07	1.86512E-07	Fur family transcriptional regulator, iron response regulator
K09829	0	0	1.04657E-08	5.4955E-10	C-8 sterol isomerase [EC:5.-.-.-]
K09833	0	0	0	8.03716E-09	homogentisate phytyltransferase / homogentisate geranylgeranyltransferase [EC:2.5.1.115 2.5.1.116]
K09835	1.44513E-06	3.15151E-07	4.00124E-06	2.80827E-05	prolycopene isomerase [EC:5.2.1.13]
K09836	2.1894E-07	4.94744E-08	3.67296E-07	8.61221E-08	beta-carotene ketolase (CrtW type)
K09844	9.7076E-07	1.11305E-08	8.07648E-08	1.30606E-07	carotenoid 1,2-hydratase [EC:4.2.1.131]
K09845	1.19347E-06	1.52844E-08	4.0963E-07	2.36533E-07	1-hydroxycarotenoid 3,4-desaturase [EC:1.3.99.27]

K09846	9.7076E-07	1.11305E-08	8.07648E-08	1.40781E-07	demethylspheroidene O-methyltransferase [EC:2.1.1.210]
K09847	0	0	0	1.52139E-08	spheroidene monooxygenase [EC:1.14.15.9]
K09857	0.000214755	0.000213764	0.000191357	0.000136921	uncharacterized protein
K09858	0.000222554	0.000226176	0.000241517	0.000287564	SEC-C motif domain protein
K09859	0	7.56793E-07	4.34608E-07	1.66747E-05	uncharacterized protein
K09860	0	1.35781E-08	2.8856E-07	1.67323E-05	uncharacterized protein
K09861	0.000218619	0.000221231	0.000216553	0.000173383	uncharacterized protein
K09862	0.000218107	0.000215808	0.000205919	0.000166172	uncharacterized protein
K09879	1.44432E-07	1.63598E-07	1.11397E-06	1.70522E-07	isorenieratene synthase
K09880	1.31033E-06	1.51879E-06	2.13181E-06	5.60272E-05	enolase-phosphatase E1 [EC:3.1.3.77]
K09882	2.93819E-06	3.19471E-06	1.48783E-05	3.53975E-05	cobaltochelatase CobS [EC:6.6.1.2]
K09883	3.20754E-06	3.18432E-06	1.45509E-05	3.48938E-05	cobaltochelatase CobT [EC:6.6.1.2]
K09888	0.000220006	0.000219067	0.000211801	0.000218385	cell division protein ZapA
K09889	0.000217642	0.00021692	0.000206843	0.000217437	ribosome-associated protein
K09890	2.94055E-07	7.5176E-07	1.32023E-06	1.12694E-05	alternative ribosome-rescue factor
K09891	9.81972E-08	3.14545E-07	3.46135E-07	1.71586E-05	uncharacterized protein
K09892	1.35555E-06	1.99109E-06	3.28346E-06	6.73712E-05	cell division protein ZapB
K09893	1.06155E-07	3.14581E-07	3.74461E-07	1.71706E-05	regulator of ribonuclease activity B
K09894	9.82118E-08	3.14581E-07	3.58969E-07	1.71496E-05	uncharacterized protein
K09895	2.45335E-06	1.99578E-06	3.90943E-06	6.9124E-05	uncharacterized protein
K09896	1.00987E-07	3.25759E-07	3.49366E-07	1.22928E-05	uncharacterized protein
K09897	9.8231E-08	3.14629E-07	3.73115E-07	1.67103E-05	uncharacterized protein
K09898	2.5355E-07	7.51807E-07	1.5033E-06	1.75936E-05	uncharacterized protein
K09899	9.82118E-08	3.14581E-07	3.48557E-07	1.71496E-05	uncharacterized protein
K09900	9.82118E-08	3.13431E-07	3.66745E-07	1.47717E-05	uncharacterized protein
K09901	9.82183E-08	3.14597E-07	5.35773E-07	1.87429E-05	uncharacterized protein
K09902	2.45338E-06	1.99587E-06	3.88876E-06	6.8612E-05	uncharacterized protein
K09903	0.000223576	0.000223765	0.000228843	0.00022547	uridylate kinase [EC:2.7.4.22]
K09904	9.82118E-08	3.14581E-07	3.48557E-07	1.71183E-05	uncharacterized protein
K09906	1.23687E-06	3.23029E-07	1.01729E-06	1.30573E-05	elongation factor P hydroxylase [EC:1.14.-.-]
K09907	9.82345E-08	3.14637E-07	3.4872E-07	1.37409E-05	uncharacterized protein
K09908	2.53568E-07	7.51783E-07	1.49296E-06	1.62591E-05	uncharacterized protein
K09909	1.21298E-06	3.80986E-07	1.35172E-06	2.70976E-05	uncharacterized protein
K09910	9.8215E-08	3.14589E-07	3.80326E-07	1.85342E-05	uncharacterized protein
K09911	9.82539E-08	3.14686E-07	1.61691E-07	9.22183E-06	uncharacterized protein
K09912	1.47398E-06	2.10725E-06	3.16634E-06	6.74178E-05	uncharacterized protein
K09913	0.000217566	0.000215705	0.000205633	0.0001643	purine/pyrimidine-nucleoside phosphorylase [EC:2.4.2.1 2.4.2.2]
K09914	0.000215563	0.000215191	0.000202582	0.000161349	putative lipoprotein
K09915	3.05074E-06	3.90454E-06	1.94983E-05	0.000104157	uncharacterized protein
K09916	1.3922E-06	7.60186E-07	2.1782E-06	1.76785E-05	uncharacterized protein
K09917	9.82118E-08	3.14581E-07	1.61387E-07	8.3814E-06	uncharacterized protein
K09918	2.3308E-07	7.51763E-07	1.33022E-06	1.76107E-05	uncharacterized protein
K09919	0.000219335	0.000216847	0.000207309	0.000206836	uncharacterized protein
K09920	1.31471E-06	1.98738E-06	3.17962E-06	6.90991E-05	uncharacterized protein
K09921	0.000216846	0.000216959	0.000206498	0.000217563	uncharacterized protein
K09922	4.04048E-06	8.01914E-06	3.26717E-05	9.91789E-05	uncharacterized protein
K09923	3.12626E-07	7.70062E-07	1.67775E-06	1.68229E-05	uncharacterized protein
K09924	0.000430089	0.000426533	0.000375874	0.000281299	uncharacterized protein
K09925	2.15203E-06	3.63342E-06	1.56479E-05	0.000102597	uncharacterized protein
K09926	0.000216579	0.000215309	0.000204877	0.000164742	uncharacterized protein
K09927	2.71738E-06	4.58474E-06	1.85222E-05	9.52682E-05	uncharacterized protein
K09928	1.8735E-07	8.262E-07	3.8197E-06	1.82072E-05	uncharacterized protein
K09929	1.83548E-06	1.02681E-06	4.02256E-06	0.000109901	uncharacterized protein
K09930	0.000216053	0.000213079	0.000192302	0.000231223	uncharacterized protein
K09931	1.92489E-06	2.16389E-07	7.33503E-07	3.57806E-07	uncharacterized protein
K09932	1.7533E-06	8.09898E-07	2.44368E-06	4.36694E-06	uncharacterized protein
K09933	0.000216405	0.000218288	0.000206799	0.000209964	MtfA peptidase
K09934	0.000213821	0.000211821	0.000186445	0.000113447	uncharacterized protein
K09935	1.5388E-06	3.25275E-06	1.6044E-05	5.96512E-05	uncharacterized protein
K09936	8.71818E-06	5.85102E-06	2.21788E-05	5.37806E-05	bacterial/archaeal transporter family-2 protein
K09937	0.000214966	0.000213534	0.000190066	0.000178826	uncharacterized protein
K09938	1.22443E-06	1.68704E-06	2.99727E-06	6.82366E-05	uncharacterized protein
K09939	0.000219091	0.00022032	0.000218803	0.000292747	uncharacterized protein
K09940	3.35556E-06	6.719E-06	6.88986E-06	5.83463E-05	uncharacterized protein
K09941	0.000216181	0.000216079	0.000204679	0.000213359	uncharacterized protein
K09942	3.19681E-08	9.5208E-10	9.11047E-08	1.90174E-09	uncharacterized protein
K09943	1.61787E-06	3.43978E-06	1.46225E-05	3.47325E-05	uncharacterized protein
K09944	0	0	9.54637E-09	0	uncharacterized protein
K09945	0.000215402	0.000212163	0.000188388	0.000114267	uncharacterized protein
K09946	2.34201E-06	2.44518E-06	5.05933E-06	1.55031E-05	uncharacterized protein
K09947	1.23131E-06	3.46491E-06	1.73562E-05	4.37323E-05	uncharacterized protein
K09948	1.49259E-06	1.18458E-06	1.81027E-06	3.51439E-06	uncharacterized protein
K09949	1.831E-06	4.16489E-07	7.43111E-07	1.93321E-07	uncharacterized protein
K09950	1.85271E-06	1.92146E-06	8.05718E-06	0.000118249	uncharacterized protein
K09951	1.9863E-06	1.82182E-06	1.6446E-05	1.01903E-05	CRISPR-associated protein Cas2
K09952	1.89754E-07	1.99881E-07	2.11451E-06	1.7354E-06	CRISPR-associated endonuclease Csn1 [EC:3.1.-.-]
K09953	9.03858E-10	2.24809E-09	6.50906E-09	2.81874E-08	lipid A 3-O-deacylase
K09954	0.000215516	0.000216593	0.00020399	0.000164747	uncharacterized protein
K09955	1.98127E-06	3.94632E-06	2.83694E-06	4.86335E-07	uncharacterized protein
K09956	1.02842E-06	5.38062E-07	1.67525E-06	3.11081E-06	uncharacterized protein
K09957	1.65559E-07	6.73719E-07	2.28996E-06	1.84917E-06	uncharacterized protein
K09958	3.68709E-06	4.05215E-06	1.73808E-05	7.30802E-05	uncharacterized protein
K09959	3.7091E-06	7.624E-06	1.76207E-05	5.17854E-05	uncharacterized protein
K09960	3.44346E-07	7.2037E-07	3.06165E-06	9.14196E-06	uncharacterized protein
K09961	3.73294E-07	8.16546E-07	3.1388E-06	9.3013E-06	uncharacterized protein
K09962	0.000214816	0.000213301	0.000191953	0.000142706	uncharacterized protein
K09963	1.24812E-06	2.06817E-07	8.74314E-06	2.56972E-06	uncharacterized protein

K09964	9.47623E-08	1.43944E-07	1.2398E-06	1.04164E-05	uncharacterized protein
K09965	0.000216375	0.000215255	0.000201609	0.000180705	uncharacterized protein
K09966	1.19834E-06	1.71881E-07	1.28747E-06	3.69471E-06	uncharacterized protein
K09967	9.26433E-07	2.18062E-06	3.30048E-06	4.07769E-06	uncharacterized protein
K09968	1.18968E-07	6.66587E-08	4.04068E-08	2.42539E-06	uncharacterized protein
K09969	2.53991E-06	3.86326E-06	1.759E-05	4.80688E-05	general L-amino acid transport system substrate-binding protein
K09970	2.39817E-06	4.46068E-06	2.33119E-05	5.63128E-05	general L-amino acid transport system permease protein
K09971	4.56547E-06	7.02342E-06	3.46199E-05	8.77541E-05	general L-amino acid transport system permease protein
K09972	0.000871075	0.000866173	0.0008128	0.00063192	general L-amino acid transport system ATP-binding protein [EC:3.6.3.-]
K09973	3.14161E-06	6.43528E-06	1.91295E-05	0.000100203	uncharacterized protein
K09974	7.5583E-07	1.57236E-06	2.21149E-06	4.26128E-05	uncharacterized protein
K09975	0.000216341	0.00021654	0.00020604	0.000204151	uncharacterized protein
K09976	1.36341E-06	3.44262E-07	6.79212E-06	3.63511E-06	uncharacterized protein
K09977	3.13433E-07	5.35366E-07	1.64629E-06	9.13729E-06	uncharacterized protein
K09978	5.03346E-07	7.84054E-07	1.52894E-06	5.92318E-06	uncharacterized protein
K09979	9.82782E-08	3.01168E-07	5.88789E-07	8.13788E-07	uncharacterized protein
K09980	1.52188E-06	3.08007E-06	1.5081E-05	3.97934E-05	uncharacterized protein
K09981	2.36424E-06	3.54259E-06	1.63362E-05	9.10866E-05	uncharacterized protein
K09982	2.75715E-07	3.56619E-07	4.24525E-07	3.84781E-06	uncharacterized protein
K09983	0.000216762	0.000215226	0.000202195	0.000155353	uncharacterized protein
K09984	0.00021535	0.000214796	0.000201521	0.000151738	uncharacterized protein
K09985	1.70111E-06	1.99396E-07	6.21153E-07	1.76865E-07	uncharacterized protein
K09986	3.00305E-06	6.45002E-07	2.48207E-06	5.54923E-06	uncharacterized protein
K09987	1.70111E-06	1.99396E-07	6.1998E-07	1.76865E-07	uncharacterized protein
K09988	3.89632E-07	7.73657E-07	6.93808E-07	5.21337E-07	D-lyxose ketol-isomerase [EC:5.3.1.15]
K09989	0.000219509	0.000215813	0.000206234	0.000155536	uncharacterized protein
K09990	0.000216408	0.000218498	0.000214628	0.000184269	uncharacterized protein
K09991	1.70197E-06	1.99396E-07	6.03711E-07	1.84472E-07	uncharacterized protein
K09992	1.10892E-06	4.67108E-07	8.19965E-06	2.1727E-06	uncharacterized protein
K09994	1.12505E-08	4.65192E-08	1.64068E-07	2.63786E-07	aminoalkylphosphonate N-acetyltransferase [EC:2.3.1.-]
K09996	1.9666E-07	6.21104E-07	5.03002E-07	6.08773E-06	arginine transport system substrate-binding protein
K09997	2.95046E-07	6.83134E-07	5.60405E-07	1.32044E-05	arginine transport system substrate-binding protein
K09998	1.52171E-07	3.6836E-07	5.99809E-07	6.48638E-06	arginine transport system permease protein
K09999	1.24004E-06	7.431E-07	1.72725E-06	8.0351E-06	arginine transport system permease protein
K10000	0.000218292	0.000212974	0.000188804	0.000127912	arginine transport system ATP-binding protein [EC:3.6.3.-]
K10001	0.001293588	0.001287272	0.00120611	0.000867917	glutamate/aspartate transport system substrate-binding protein
K10002	0.000433641	0.00043075	0.000395448	0.000290792	glutamate/aspartate transport system permease protein
K10003	0.000218358	0.000215336	0.000205011	0.000150367	glutamate/aspartate transport system permease protein
K10004	0.001080564	0.001078778	0.000997913	0.000714392	glutamate/aspartate transport system ATP-binding protein [EC:3.6.3.-]
K10005	2.28863E-06	6.56371E-06	1.76526E-05	3.85878E-05	glutamate transport system substrate-binding protein
K10006	2.40503E-06	6.55614E-06	1.77587E-05	3.86063E-05	glutamate transport system permease protein
K10007	4.6389E-06	6.93644E-06	2.14446E-05	3.99189E-05	glutamate transport system permease protein
K10008	0.000438128	0.000441094	0.000413589	0.000313733	glutamate transport system ATP-binding protein [EC:3.6.3.-]
K10009	8.44732E-06	5.7985E-06	2.96009E-05	6.29757E-05	cystine transport system permease protein
K10010	0.000446141	0.00044285	0.000440032	0.000416947	cystine transport system ATP-binding protein [EC:3.6.3.-]
K10011	0.000214067	0.000212218	0.000186809	0.000114808	UDP-4-amino-4-deoxy-L-arabinose formyltransferase / UDP-glucuronic acid dehydrogenase (UDP-4-keto-hexauronic
K10012	0.000214789	0.000213882	0.000192306	0.000129679	undecaprenyl-phosphate 4-deoxy-4-formamido-L-arabinose transferase [EC:2.4.2.53]
K10013	6.20537E-07	1.62154E-06	1.63697E-06	6.95264E-06	lysine/arginine/ornithine transport system substrate-binding protein
K10014	5.15188E-07	8.18083E-07	8.99364E-07	5.17803E-06	histidine transport system substrate-binding protein
K10015	3.29242E-07	6.20295E-07	9.66191E-07	9.44326E-06	histidine transport system permease protein
K10016	3.34988E-07	7.2176E-07	2.3752E-06	1.31446E-05	histidine transport system permease protein
K10017	5.38564E-06	7.21232E-06	2.84124E-05	6.07009E-05	histidine transport system ATP-binding protein [EC:3.6.3.21]
K10018	1.97785E-06	6.37443E-07	1.68698E-06	3.62596E-06	octopine/nopaline transport system substrate-binding protein
K10019	1.52928E-06	1.44207E-06	4.78192E-06	1.71581E-05	octopine/nopaline transport system permease protein
K10020	1.23887E-07	3.72289E-07	3.89238E-07	3.27925E-06	octopine/nopaline transport system permease protein
K10021	5.03247E-06	4.47092E-06	1.01101E-05	1.34099E-05	octopine/nopaline transport system ATP-binding protein [EC:3.6.3.-]
K10022	9.44766E-07	5.81268E-07	3.09951E-06	2.56604E-05	arginine/ornithine transport system substrate-binding protein
K10023	1.36617E-06	1.09862E-06	4.09571E-06	1.24284E-05	arginine/ornithine transport system permease protein
K10024	3.03639E-07	7.16761E-07	1.98412E-06	1.2741E-05	arginine/ornithine transport system permease protein
K10025	5.65913E-06	2.37044E-06	1.19343E-05	2.20583E-05	arginine/ornithine transport system ATP-binding protein [EC:3.6.3.-]
K10026	0.000221524	0.000219543	0.000216134	0.000218246	7-carboxy-7-deazaguanine synthase [EC:4.3.99.3]
K10027	5.69494E-06	5.58109E-06	8.01385E-06	9.08158E-06	phytoene desaturase [EC:1.3.99.26 1.3.99.28 1.3.99.29 1.3.99.31]
K10036	2.36136E-07	6.9136E-07	7.709E-07	1.05654E-06	glutamine transport system substrate-binding protein
K10037	3.84517E-07	7.01121E-07	9.65454E-07	1.74031E-06	glutamine transport system permease protein
K10038	0.000219696	0.000216186	0.000217957	0.000140672	glutamine transport system ATP-binding protein [EC:3.6.3.-]
K10039	1.20712E-06	1.74007E-07	4.02226E-06	3.38409E-06	putative glutamine transport system substrate-binding protein
K10040	0.000217678	0.000216808	0.000193196	0.00013179	putative glutamine transport system permease protein
K10041	0.000654297	0.000655543	0.000622027	0.000478431	putative glutamine transport system ATP-binding protein [EC:3.6.3.-]
K10085	3.94687E-07	6.58299E-08	8.66094E-08	0	ER degradation enhancer, mannosidase alpha-like 2
K10094	1.20338E-06	2.92876E-06	1.37518E-05	3.46189E-05	nickel transport protein
K10107	3.70519E-06	1.61052E-07	9.73096E-07	1.49221E-06	capsular polysaccharide transport system permease protein
K10108	1.02791E-07	7.76799E-07	9.06267E-07	1.5419E-05	maltose/maltodextrin transport system substrate-binding protein
K10109	1.64377E-06	3.46745E-06	7.30144E-06	1.16872E-05	maltose/maltodextrin transport system permease protein
K10110	1.06261E-06	3.60644E-06	2.47201E-06	1.15009E-05	maltose/maltodextrin transport system permease protein
K10111	0.000229848	0.000241237	0.000293937	0.000397441	multiple sugar transport system ATP-binding protein [EC:3.6.3.-]
K10112	0.00134354	0.001356699	0.001437259	0.001513915	multiple sugar transport system ATP-binding protein
K10117	2.38268E-06	2.38744E-06	1.74727E-05	1.29189E-05	raffinose/stachyose/melibiose transport system substrate-binding protein
K10118	3.81818E-06	2.76472E-06	1.96814E-05	1.4282E-05	raffinose/stachyose/melibiose transport system permease protein
K10119	4.42484E-06	5.95271E-06	2.58286E-05	1.49431E-05	raffinose/stachyose/melibiose transport system permease protein
K10120	1.18631E-08	0	5.26942E-09	9.63027E-07	fructooligosaccharide transport system substrate-binding protein
K10121	1.18631E-08	1.85547E-09	5.50653E-08	9.6645E-07	fructooligosaccharide transport system permease protein
K10122	1.18631E-08	0	5.26942E-09	9.62824E-07	fructooligosaccharide transport system permease protein
K10123	0	1.35781E-08	6.41787E-08	8.40539E-06	putative ferrous iron transport protein C
K10124	3.08626E-08	2.882E-08	6.60549E-08	7.85524E-07	carbohydrate-specific outer membrane porin
K10125	0.000884195	0.000873116	0.000846351	0.000911819	two-component system, NtrC family, C4-dicarboxylate transport sensor histidine kinase DctB [EC:2.7.13.3]
K10126	0.001961122	0.001951479	0.001863553	0.001758133	two-component system, NtrC family, C4-dicarboxylate transport response regulator DctD
K10156	0	0	3.99847E-08	1.78604E-09	epi-isozizaene synthase [EC:4.2.3.37]

K10187	0	0	3.34326E-07	1.20468E-07	germacradienol/geosmin synthase [EC:4.2.3.22 4.2.3.75 4.1.99.16]
K10188	1.16256E-07	3.47877E-07	3.80883E-07	1.90877E-06	lactose/L-arabinose transport system substrate-binding protein
K10189	5.51409E-07	7.98149E-07	8.91443E-07	4.09787E-06	lactose/L-arabinose transport system permease protein
K10190	2.28297E-07	7.71825E-07	1.40214E-06	2.08904E-06	lactose/L-arabinose transport system permease protein
K10191	2.12297E-06	5.65875E-06	2.67145E-05	5.43241E-05	lactose/L-arabinose transport system ATP-binding protein
K10192	2.603E-07	2.04356E-07	1.62212E-07	3.45448E-06	oligogalacturonide transport system substrate-binding protein
K10193	4.48203E-07	4.79096E-07	3.24417E-06	3.53519E-06	oligogalacturonide transport system permease protein
K10194	4.05566E-07	2.78792E-07	6.05025E-07	3.46206E-06	oligogalacturonide transport system permease protein
K10195	1.53285E-06	1.45708E-06	9.97346E-06	1.90279E-05	oligogalacturonide transport system ATP-binding protein
K10199	1.28406E-06	3.86192E-06	2.22368E-05	3.49096E-05	glucose/arabinose transport system ATP-binding protein
K10200	8.51732E-07	5.82314E-07	6.4397E-06	8.50591E-08	N-acetylglucosamine transport system substrate-binding protein
K10201	1.02948E-06	7.17815E-07	6.82624E-06	1.52158E-06	N-acetylglucosamine transport system permease protein
K10202	8.47593E-07	5.75075E-07	6.61599E-06	1.56318E-07	N-acetylglucosamine transport system permease protein
K10206	4.62784E-06	6.74422E-06	2.48563E-05	7.88676E-06	LL-diaminopimelate aminotransferase [EC:2.6.1.83]
K10208	9.72628E-07	0	3.0326E-06	3.03626E-08	4,4-diapophytoene synthase [EC:2.5.1.96]
K10209	9.73485E-07	0	2.66666E-06	3.04953E-08	4,4-diapophytoene desaturase [EC:1.3.8.2]
K10210	2.05072E-06	4.96687E-09	3.61574E-06	5.74247E-08	diapolycopene oxygenase [EC:1.14.99.44]
K10211	1.00411E-06	1.02646E-08	2.88376E-06	1.31407E-07	4,4-diaponeurosporenoate glycosyltransferase [EC:2.4.1.-]
K10212	1.01564E-06	2.66094E-09	2.80437E-06	3.80833E-08	glycosyl-4,4-diaponeurosporenoate acyltransferase [EC:2.3.1.-]
K10213	3.3531E-07	1.99084E-07	8.85237E-07	2.66579E-07	ribosylpyrimidine nucleosidase [EC:3.2.2.8]
K10215	7.31541E-07	1.89473E-06	8.00174E-06	1.79343E-05	monooxygenase [EC:1.14.13.-]
K10216	1.52692E-07	1.15256E-06	3.76697E-06	5.11658E-06	2-hydroxymuconate-semialdehyde hydrolase [EC:3.7.1.9]
K10217	0.000650702	0.000649035	0.000606052	0.000499377	aminomuconate-semialdehyde/2-hydroxymuconate-6-semialdehyde dehydrogenase [EC:1.2.1.32 1.2.1.85]
K10218	0.000436501	0.000441802	0.000446618	0.000423579	4-hydroxy-4-methyl-2-oxoglutarate aldolase [EC:4.1.3.17]
K10219	1.50106E-06	3.64263E-06	1.70913E-05	4.40852E-05	2-hydroxy-4-carboxymuconate semialdehyde hemiacetal dehydrogenase [EC:1.1.1.312]
K10220	1.77059E-06	3.79464E-06	1.76919E-05	6.28702E-05	4-oxalmesaconate hydratase [EC:4.2.1.83]
K10221	5.36813E-06	1.21888E-05	5.85641E-05	0.000159972	2-pyrone-4,6-dicarboxylate lactonase [EC:3.1.1.57]
K10222	9.86965E-10	1.27658E-07	3.11685E-07	8.79284E-08	2,6-dioxo-6-phenylhexa-3-enoate hydrolase [EC:3.7.1.8]
K10227	1.65109E-06	3.71885E-06	1.66678E-05	3.8013E-05	sorbitol/mannitol transport system substrate-binding protein
K10228	1.56287E-06	3.70405E-06	1.68961E-05	3.92634E-05	sorbitol/mannitol transport system permease protein
K10229	1.85623E-06	3.84105E-06	2.11005E-05	4.3814E-05	sorbitol/mannitol transport system permease protein
K10231	1.28595E-06	3.05118E-08	2.63125E-07	1.06898E-07	kojibiose phosphorylase [EC:2.4.1.230]
K10232	3.79793E-08	4.63447E-08	7.39419E-07	4.19229E-06	alpha-glucoside transport system substrate-binding protein
K10233	1.60391E-07	7.47775E-08	3.60169E-06	6.33017E-06	alpha-glucoside transport system permease protein
K10234	1.33534E-06	4.43767E-07	9.51574E-06	4.79996E-06	alpha-glucoside transport system permease protein
K10235	3.09954E-06	6.31956E-06	2.52819E-05	5.00839E-05	alpha-glucoside transport system ATP-binding protein
K10236	1.09009E-08	1.12961E-07	1.15776E-06	4.28979E-07	trehalose/maltose transport system substrate-binding protein
K10237	4.82486E-07	1.04093E-06	7.6968E-06	2.49484E-06	trehalose/maltose transport system permease protein
K10238	1.56631E-07	6.76999E-07	2.81589E-06	2.38305E-06	trehalose/maltose transport system permease protein
K10240	5.81456E-07	4.8405E-07	6.5569E-06	6.47585E-06	cellobiose transport system substrate-binding protein
K10241	1.69045E-06	1.48249E-06	9.20655E-06	5.37888E-05	cellobiose transport system permease protein
K10242	6.48702E-07	8.64242E-07	8.52104E-06	3.1398E-06	cellobiose transport system permease protein
K10251	1.34805E-10	1.72387E-10	0	2.11979E-09	17beta-estradiol 17-dehydrogenase / very-long-chain 3-oxoacyl-CoA reductase [EC:1.1.1.62 1.1.1.330]
K10253	7.74325E-07	5.67982E-07	2.57191E-06	5.74515E-05	DOPA 4,5-dioxygenase [EC:1.14.99.-]
K10254	5.10733E-06	3.90129E-06	1.52199E-05	3.27631E-05	oleate hydratase [EC:4.2.1.53]
K10255	1.99069E-07	1.42295E-07	1.17543E-06	1.03973E-07	acyl-lipid omega-6 desaturase (Delta-12 desaturase) [EC:1.14.19.23 1.14.19.45]
K10297	2.997E-09	6.31053E-09	8.73379E-08	9.59474E-08	F-box protein 11
K10353	2.45886E-07	3.66551E-08	8.61492E-07	1.13843E-06	deoxyadenosine kinase [EC:2.7.1.76]
K10439	0.000863038	0.000862893	0.000807172	0.000559413	ribose transport system substrate-binding protein
K10440	0.000868929	0.000873074	0.000855068	0.00068638	ribose transport system permease protein
K10441	0.001313129	0.001315623	0.001302945	0.001094292	ribose transport system ATP-binding protein [EC:3.6.3.17]
K10530	1.33064E-07	1.53339E-07	4.30124E-07	7.88506E-07	L-lactate oxidase [EC:1.1.3.2]
K10531	2.38527E-06	3.14492E-06	1.71998E-05	3.70149E-05	L-ornithine N5-monooxygenase [EC:1.14.13.195 1.14.13.196]
K10533	4.55979E-08	1.47E-07	1.11933E-07	6.49834E-06	limonene-1,2-epoxide hydrolase [EC:3.3.2.8]
K10535	0	1.19447E-07	1.388E-07	1.0175E-08	hydroxylamine dehydrogenase [EC:1.7.2.6]
K10536	4.38002E-06	8.9913E-06	1.67455E-05	0.000130625	agmatine deiminase [EC:3.5.3.12]
K10537	0.000214053	0.000211954	0.000186614	0.000124299	L-arabinose transport system substrate-binding protein
K10538	0.000214166	0.000211893	0.000187015	0.000125433	L-arabinose transport system permease protein
K10539	0.00043106	0.000428347	0.000389543	0.000285856	L-arabinose transport system ATP-binding protein [EC:3.6.3.17]
K10540	5.73901E-07	1.0163E-06	8.73801E-07	1.19581E-05	methyl-galactoside transport system substrate-binding protein
K10541	0.000214513	0.000213078	0.00019254	0.000132101	methyl-galactoside transport system permease protein
K10542	0.000431336	0.000427701	0.000383707	0.000267308	methyl-galactoside transport system ATP-binding protein [EC:3.6.3.17]
K10543	0.000214129	0.000212904	0.000188037	0.000118824	D-xylose transport system substrate-binding protein
K10544	0.000431174	0.000430151	0.000393188	0.000281715	D-xylose transport system permease protein
K10545	0.000646918	0.000644095	0.000589851	0.00043475	D-xylose transport system ATP-binding protein [EC:3.6.3.17]
K10546	1.57728E-06	5.93949E-07	3.6554E-06	2.78167E-06	putative multiple sugar transport system substrate-binding protein
K10547	0.000431477	0.00043059	0.000400995	0.000292303	putative multiple sugar transport system permease protein
K10548	0.000647467	0.000643667	0.00058732	0.000404779	putative multiple sugar transport system ATP-binding protein [EC:3.6.3.17]
K10549	1.25424E-08	4.3161E-08	9.40436E-08	2.22223E-07	D-allose transport system substrate-binding protein
K10550	5.13272E-09	1.97008E-08	7.07478E-08	2.22564E-07	D-allose transport system permease protein
K10551	0.000429595	0.000426147	0.00037713	0.000235967	D-allose transport system ATP-binding protein [EC:3.6.3.17]
K10552	1.68069E-07	3.332E-07	4.34328E-07	4.13958E-07	fructose transport system substrate-binding protein
K10553	0.000215632	0.000215342	0.000201926	0.000152057	fructose transport system permease protein
K10554	2.11868E-06	5.05118E-06	1.93305E-05	4.31145E-05	fructose transport system ATP-binding protein
K10555	1.30991E-07	4.48985E-08	4.73595E-08	7.0395E-07	AI-2 transport system substrate-binding protein
K10556	1.01529E-07	2.16697E-08	1.80566E-07	7.76496E-07	AI-2 transport system permease protein
K10557	1.35889E-07	1.30377E-07	4.68803E-08	7.24462E-07	AI-2 transport system permease protein
K10558	0.000215767	0.000213287	0.000197873	0.000130944	AI-2 transport system ATP-binding protein
K10559	2.66806E-07	2.05302E-07	5.04369E-07	2.18504E-07	rhamnose transport system substrate-binding protein
K10560	5.58647E-07	4.32895E-07	1.41714E-06	2.62575E-07	rhamnose transport system permease protein
K10561	1.63853E-06	3.49716E-06	1.44687E-05	3.4903E-05	rhamnose transport system permease protein
K10562	0.000646164	0.000640615	0.000586016	0.00038113	rhamnose transport system ATP-binding protein [EC:3.6.3.17]
K10563	0.000224302	0.000226388	0.000235144	0.0002298	formamidopyrimidine-DNA glycosylase [EC:3.2.2.23 4.2.99.18]
K10564	9.91739E-07	1.35498E-07	1.00948E-07	3.57629E-08	chemotaxis protein MotC
K10565	1.37006E-08	1.21701E-07	7.80425E-08	0	chemotaxis protein MotD
K10616	0	3.27506E-09	2.59514E-08	0	p-cymene methyl-monooxygenase [EC:1.14.15.25]
K10617	3.09401E-08	3.40185E-08	2.2775E-07	6.21397E-08	p-cumic alcohol dehydrogenase

K10619	1.23012E-07	3.94306E-07	1.84424E-07	4.86869E-07	p-cumate 2,3-dioxygenase subunit alpha [EC:1.14.12.25]
K10620	8.59948E-09	1.10451E-09	2.82408E-09	4.47196E-08	2,3-dihydroxy-2,3-dihydro-p-cumate dehydrogenase [EC:1.3.1.58]
K10621	0.000213791	0.000211831	0.000186637	0.000113221	2,3-dihydroxy-p-cumate/2,3-dihydroxybenzoate 3,4-dioxygenase [EC:1.13.11.- 1.13.11.14]
K10622	0.000213774	0.000211779	0.000186309	0.000113202	HCOMODA/2-hydroxy-3-carboxy-muconic semialdehyde decarboxylase [EC:4.1.1.-]
K10623	0	0	1.37217E-07	4.9107E-08	HOMODA hydrolase [EC:3.7.1.-]
K10670	1.94864E-07	1.45103E-10	7.12903E-07	2.71955E-08	glycine/sarcosine/betaine reductase complex component A [EC:1.21.4.2 1.21.4.3 1.21.4.4]
K10671	9.74318E-08	7.25515E-11	2.67391E-07	3.90651E-08	glycine reductase complex component B subunit alpha and beta [EC:1.21.4.2]
K10672	1.94864E-07	3.87996E-08	6.23843E-07	6.53964E-08	glycine reductase complex component B subunit gamma [EC:1.21.4.2]
K10673	1.10035E-07	2.48152E-08	4.0486E-07	2.55764E-07	streptomycin 3\-kinase [EC:2.7.1.87]
K10674	1.25699E-07	9.93411E-07	7.88321E-07	1.01411E-06	ectoine hydroxylase [EC:1.14.11.55]
K10676	1.35117E-06	1.34594E-06	6.25765E-06	3.94024E-05	2,4-dichlorophenol 6-monooxygenase [EC:1.14.13.20]
K10677	1.97006E-08	4.87385E-08	2.93563E-07	8.06477E-08	inulin fructotransferase (DFA-I-forming) [EC:4.2.2.17]
K10678	1.77653E-06	3.11065E-07	1.44616E-06	1.51838E-05	nitroreductase [EC:1.-.-.-]
K10679	1.54436E-06	1.91269E-06	5.61797E-06	1.84514E-05	nitroreductase / dihydropteridine reductase [EC:1.-.-.- 1.5.1.34]
K10680	0.001518531	0.001511526	0.001393348	0.00122086	N-ethylmaleimide reductase [EC:1.-.-.-]
K10681	4.25282E-06	4.3336E-06	2.038E-05	8.96812E-05	two-component system, OmpR family, sensor histidine kinase SaeS [EC:2.7.13.3]
K10682	1.33172E-06	4.62382E-07	7.03686E-06	4.14668E-06	two-component system, OmpR family, response regulator SaeR
K10697	0.000215192	0.000212942	0.000189236	0.000153872	two-component system, OmpR family, response regulator RpaA
K10702	1.44434E-10	1.58932E-07	3.64171E-08	1.90174E-09	2-hydroxy-6-oxohepta-2,4-dienoate hydroxylase [EC:3.7.1.-]
K10703	0	0	0	3.80348E-09	very-long-chain (3R)-3-hydroxyacyl-CoA dehydratase [EC:4.2.1.134]
K10708	3.92974E-08	3.49427E-08	1.07804E-07	2.79742E-07	fructoselysine 6-phosphate deglycase [EC:3.5.-.-]
K10709	3.3179E-09	1.24661E-07	5.16301E-07	2.00489E-06	fructoselysine 3-epimerase [EC:5.1.3.41]
K10710	2.9762E-08	7.74798E-08	6.49424E-07	2.13757E-06	fructoselysine 6-kinase [EC:2.7.1.218]
K10711	6.43927E-09	3.47486E-08	1.54709E-07	5.22555E-07	GntR family transcriptional regulator, frlABCD operon transcriptional regulator
K10713	1.98172E-06	8.39533E-08	1.63785E-07	3.05112E-07	5,6,7,8-tetrahydromethanopterin hydro-lyase [EC:4.2.1.147]
K10714	9.87446E-07	4.21631E-08	6.3322E-08	1.56028E-07	methylene-tetrahydromethanopterin dehydrogenase [EC:1.5.1.-]
K10715	0.000233012	0.000225785	0.000241498	0.000402258	two-component system, sensor histidine kinase RpfC [EC:2.7.13.3]
K10716	5.80382E-06	6.54395E-06	2.81694E-05	0.000106391	voltage-gated potassium channel
K10725	6.48218E-12	1.61226E-11	3.77272E-08	2.03218E-06	archaeal cell division control protein 6
K10726	0	0	9.26651E-08	0	replicative DNA helicase Mcm [EC:3.6.4.-]
K10742	8.14889E-07	2.10111E-06	5.5247E-06	1.35808E-05	DNA replication ATP-dependent helicase Dna2 [EC:3.6.4.12]
K10747	4.33501E-06	5.88649E-06	2.06255E-05	0.000102595	DNA ligase 1 [EC:6.5.1.1 6.5.1.6 6.5.1.7]
K10748	9.85002E-08	3.0287E-07	2.72755E-07	6.94719E-06	DNA replication terminus site-binding protein
K10762	1.30348E-08	5.91824E-08	6.04703E-08	1.05203E-06	putative replication protein
K10763	0.000218033	0.000216945	0.000207081	0.000211073	DnaA-homolog protein
K10764	0.000446042	0.000450801	0.000447892	0.000527877	O-succinylhomoserine sulfhydrylase [EC:2.5.1.-]
K10773	0.000225693	0.000224561	0.000229085	0.000226799	endonuclease III [EC:4.2.99.18]
K10774	0	0	2.22133E-08	1.90174E-09	tyrosine ammonia-lyase [EC:4.3.1.23]
K10775	5.15016E-08	2.45629E-08	1.27914E-08	2.82626E-09	phenylalanine ammonia-lyase [EC:4.3.1.24]
K10778	0.000864342	0.000858685	0.000781293	0.000566462	AraC family transcriptional regulator, regulatory protein of adaptative response / methylated-DNA-[protein]-cysteine n
K10780	1.42402E-06	4.8137E-07	1.88683E-06	5.23426E-06	enoyl-[acyl-carrier protein] reductase III [EC:1.3.1.104]
K10793	0	1.02051E-08	2.63691E-08	2.87364E-08	D-proline reductase (dithiol) PrdA [EC:1.21.4.1]
K10794	0	1.02051E-08	2.63691E-08	2.87364E-08	D-proline reductase (dithiol) PrdB [EC:1.21.4.1]
K10795	0	0	1.04657E-08	1.29419E-08	D-proline reductase (dithiol)-stabilizing protein PrdD
K10796	0	1.02051E-08	1.07415E-08	1.29419E-08	D-proline reductase (dithiol)-stabilizing protein PrdE
K10797	0.000215748	0.000213201	0.000196192	0.000116683	2-enoate reductase [EC:1.3.1.31]
K10798	0	0	3.65944E-07	0	poly [ADP-ribose] polymerase [EC:2.4.2.30]
K10800	9.9505E-08	2.17092E-07	1.04657E-08	5.71442E-09	single-strand selective monofunctional uracil DNA glycosylase [EC:3.2.2.-]
K10804	0.000218859	0.000218553	0.00021541	0.000235214	acyl-CoA thioesterase I [EC:3.1.2.- 3.1.1.5]
K10805	5.32798E-06	6.9142E-06	1.32589E-05	8.09508E-05	acyl-CoA thioesterase II [EC:3.1.2.-]
K10806	0.000218997	0.000216851	0.000206707	0.000166979	acyl-CoA thioesterase YciA [EC:3.1.2.-]
K10810	1.26436E-06	3.4947E-07	3.85331E-06	3.51304E-08	thiazole tautomerase (transcriptional regulator TenI) [EC:5.3.99.10]
K10811	8.56287E-10	7.49763E-09	6.3447E-08	8.03716E-09	thiamine pyridinylase [EC:2.5.1.2]
K10814	0	1.60106E-09	1.39919E-08	7.19162E-07	hydrogen cyanide synthase HcnA [EC:1.4.99.5]
K10815	4.10304E-09	1.16889E-08	2.09412E-07	7.29783E-07	hydrogen cyanide synthase HcnB [EC:1.4.99.5]
K10816	4.96461E-09	1.94634E-09	6.53625E-09	7.19162E-07	hydrogen cyanide synthase HcnC [EC:1.4.99.5]
K10817	2.43251E-09	5.16367E-08	4.6856E-06	3.28065E-07	6-deoxyerythronolide-B synthase [EC:2.3.1.94]
K10819	4.71827E-06	6.79569E-06	2.05668E-05	0.00020127	histidine kinase [EC:2.7.13.3]
K10823	0.001104353	0.001099084	0.00107215	0.000883003	oligopeptide transport system ATP-binding protein
K10824	5.37405E-06	4.98485E-06	2.79345E-05	4.73472E-05	nickel transport system ATP-binding protein [EC:3.6.3.24]
K10830	1.0121E-07	7.94201E-08	4.30013E-07	4.71976E-06	manganese/zinc transport system ATP-binding protein [EC:3.6.3.35]
K10831	0.00022239	0.000219623	0.000224224	0.000187412	taurine transport system ATP-binding protein [EC:3.6.3.36]
K10834	3.1294E-10	4.00184E-10	0	0	heme-transporting ATPase [EC:3.6.3.41]
K10843	6.6106E-07	1.48739E-06	9.13765E-06	3.92523E-06	DNA excision repair protein ERCC-3 [EC:3.6.4.12]
K10844	1.85754E-06	3.91667E-06	1.79137E-05	3.56782E-05	DNA excision repair protein ERCC-2 [EC:3.6.4.12]
K10850	9.53671E-07	0	2.95761E-06	3.03626E-08	MFS transporter, NNP family, putative nitrate transporter
K10851	9.23842E-07	0	2.87573E-06	3.03626E-08	nitrogen regulatory protein A
K10854	2.08165E-08	1.62021E-08	1.80777E-07	9.4323E-08	acetone carboxylase, alpha subunit [EC:6.4.1.6]
K10855	2.52999E-08	1.62021E-08	2.26157E-07	9.4323E-08	acetone carboxylase, beta subunit [EC:6.4.1.6]
K10856	2.08165E-08	1.62021E-08	1.75893E-07	9.4323E-08	acetone carboxylase, gamma subunit [EC:6.4.1.6]
K10857	1.44047E-06	4.24106E-07	3.04903E-06	9.95457E-07	exodeoxyribonuclease X [EC:3.1.11.-]
K10896	0	6.90546E-11	3.19992E-08	7.60696E-09	fanconi anemia group M protein
K10906	1.74609E-07	4.8273E-07	1.77023E-07	1.00186E-06	exodeoxyribonuclease VIII [EC:3.1.11.-]
K10907	0.000221233	0.000218927	0.00020216	0.000134017	aminotransferase [EC:2.6.1.-]
K10908	4.80212E-08	3.51765E-08	1.04138E-07	2.05517E-06	DNA-directed RNA polymerase, mitochondrial [EC:2.7.7.6]
K10909	4.99284E-06	3.33452E-06	1.18347E-05	0.00013708	two-component system, autoinducer 2 sensor kinase/phosphatase LuxQ [EC:2.7.13.3 3.1.3.-]
K10910	0	1.35781E-08	6.41787E-08	8.30855E-06	autoinducer 2-binding periplasmic protein LuxP
K10911	0	1.35781E-08	6.41787E-08	9.17535E-06	two-component system, phosphorelay protein LuxU
K10912	0.000650338	0.000648709	0.000617091	0.0005026	two-component system, repressor protein LuxO
K10913	0	1.35781E-08	2.51348E-07	1.17954E-05	TetR/AcrR family transcriptional regulator, hemagglutinin/protease regulatory protein
K10914	0.000226918	0.000235491	0.000287337	0.000378677	CRP/FNR family transcriptional regulator, cyclic AMP receptor protein
K10915	8.45118E-08	5.49232E-08	2.60116E-07	8.028E-06	CAI-1 autoinducer synthase [EC:2.3.-.-]
K10916	0.000427784	0.000423787	0.000372804	0.000239421	two-component system, CAI-1 autoinducer sensor kinase/phosphatase CqsS [EC:2.7.13.3 3.1.3.-]
K10917	7.18032E-09	1.13298E-07	3.9072E-07	8.8111E-06	PadR family transcriptional regulator, regulatory protein AphA
K10918	0.000431323	0.00043086	0.000398916	0.000385932	LysR family transcriptional regulator, transcriptional activator AphB
K10921	9.16819E-09	1.35781E-08	6.78657E-08	1.11709E-05	cholera toxin transcriptional activator
K10922	0	1.35781E-08	6.41787E-08	8.40941E-06	transmembrane regulatory protein ToxS

K10924	8.76412E-08	1.02668E-07	1.19187E-06	2.69131E-05	MSHA pilin protein MshA
K10925	0	1.35781E-08	2.1159E-07	1.37574E-05	MSHA pilin protein MshB
K10926	1.09807E-07	9.71194E-08	5.44029E-07	1.6092E-05	MSHA pilin protein MshC
K10927	1.24156E-06	1.25863E-07	1.39919E-06	1.40817E-05	MSHA pilin protein MshD
K10928	6.15807E-11	1.53164E-10	4.43468E-10	1.82415E-09	cholera enterotoxin subunit A [EC:2.4.2.36]
K10929	8.75094E-11	2.17655E-10	6.30191E-10	2.59222E-09	cholera enterotoxin subunit B
K10930	4.53752E-11	1.12858E-10	7.30391E-09	2.93283E-08	toxin co-regulated pilin
K10931	4.53752E-11	1.12858E-10	7.30391E-09	2.93283E-08	toxin co-regulated pilus biosynthesis protein B
K10932	5.18574E-11	1.2898E-10	7.35059E-09	2.95203E-08	toxin co-regulated pilus biosynthesis outer membrane protein C
K10934	4.53752E-11	1.12858E-10	7.30391E-09	2.93283E-08	toxin co-regulated pilus biosynthesis protein E
K10936	0	0	6.41787E-08	1.97017E-07	accessory colonization factor AcfA
K10938	6.8199E-07	7.30791E-08	6.92407E-07	2.87375E-06	accessory colonization factor AcfC
K10939	3.42533E-09	2.63059E-08	6.33492E-08	2.2434E-07	accessory colonization factor AcfD
K10940	0	0	3.97019E-08	1.61637E-06	outer membrane protein OmpT
K10941	0.000879818	0.000871447	0.000855679	0.000911673	sigma-54 dependent transcriptional regulator, flagellar regulatory protein
K10942	3.95602E-06	7.88516E-06	3.53812E-05	0.000107728	two-component system, sensor histidine kinase FlrB [EC:2.7.13.3]
K10943	0.001732714	0.00172554	0.001624506	0.001308318	two-component system, response regulator FlrC
K10944	0	1.30091E-07	1.14934E-07	3.0525E-08	methane/ammonia monooxygenase subunit A [EC:1.14.18.3 1.14.99.39]
K10945	0	1.30091E-07	1.14934E-07	3.0525E-08	methane/ammonia monooxygenase subunit B
K10946	0	2.19548E-07	1.84705E-07	5.0875E-08	methane/ammonia monooxygenase subunit C
K10947	6.68092E-06	6.44946E-06	2.24004E-05	5.87003E-05	PadR family transcriptional regulator, regulatory protein PadR
K10948	4.73985E-08	2.45629E-08	1.39414E-07	8.49634E-06	hemolysin
K10953	2.00947E-10	4.99799E-10	1.48073E-07	9.11661E-06	RTX toxin RtxA
K10954	0.000214946	0.00021381	0.000196426	0.000151154	zona occludens toxin
K10960	2.33875E-06	4.24459E-06	1.64311E-05	3.50742E-05	geranylgeranyl diphosphate/geranylgeranyl-bacteriochlorophyllide a reductase [EC:1.3.1.83 1.3.1.111]
K10961	0	1.35781E-08	0	6.10772E-09	toxin co-regulated pilus biosynthesis protein I
K10962	4.53752E-11	1.12858E-10	7.30391E-09	2.93283E-08	toxin co-regulated pilus biosynthesis protein Q
K10965	4.53752E-11	1.12858E-10	7.30391E-09	2.93283E-08	toxin co-regulated pilus biosynthesis protein T
K10972	3.25332E-06	7.11229E-06	2.97159E-05	9.88299E-05	LysR family transcriptional regulator, transcriptional activator of the allD operon
K10973	3.95472E-08	1.60126E-07	5.01105E-08	1.97757E-07	IclR family transcriptional regulator, negative regulator of allantoin and glyoxylate utilization operons
K10974	4.27671E-07	7.57238E-07	2.11316E-06	6.634E-06	cytosine permease
K10975	5.72241E-08	1.84686E-08	5.35195E-08	8.47185E-07	allantoin permease
K10977	5.94906E-09	2.00476E-09	4.49382E-08	1.97977E-08	methanogen homocitrate synthase [EC:2.3.3.14 2.3.3.-]
K10978	1.11483E-06	7.0554E-07	2.70766E-06	5.61718E-05	methanogen homoisocitrate dehydrogenase [EC:1.1.1.87 1.1.1.-]
K10979	5.22206E-06	7.42456E-06	2.70543E-05	0.000109554	DNA end-binding protein Ku
K10984	1.40851E-07	6.54061E-07	1.00763E-07	3.0326E-07	PTS system, galactosamine-specific IIB component [EC:2.7.1.-]
K10985	7.43804E-08	3.60675E-07	8.28293E-08	2.86616E-07	PTS system, galactosamine-specific IIC component
K10986	7.7117E-09	1.0886E-07	2.15326E-07	3.55151E-06	PTS system, galactosamine-specific IID component
K11003	0.00021426	0.00021198	0.000190872	0.000123209	hemolysin D
K11004	0.000226343	0.000227061	0.000241886	0.000233057	ATP-binding cassette, subfamily B, bacterial HlyB/CyaB
K11005	0.000213783	0.000211779	0.000187207	0.000114364	hemolysin A
K11006	1.62054E-09	4.03064E-09	1.16702E-08	4.80041E-08	shiga toxin subunit A
K11007	1.6173E-09	4.02258E-09	1.16469E-08	4.7908E-08	shiga toxin subunit B
K11008	8.10272E-11	2.01532E-10	5.8351E-10	2.4002E-09	cytotoxic necrotizing factor 1
K11009	0	9.82517E-09	0	0	murine toxin
K11013	9.72326E-11	2.41838E-10	7.00212E-10	2.88024E-09	cytolethal distending toxin subunit A
K11014	9.72326E-11	2.41838E-10	1.88614E-08	2.88024E-09	cytolethal distending toxin subunit B
K11015	9.72326E-11	2.41838E-10	7.00212E-10	2.88024E-09	cytolethal distending toxin subunit C
K11016	8.34841E-08	3.11521E-07	5.46028E-08	6.75045E-08	hemolysin
K11017	6.98644E-08	2.8644E-07	8.49092E-08	1.53763E-08	hemolysin activation/secretion protein
K11018	5.01138E-08	4.92657E-08	6.44433E-08	8.00558E-06	thermolabile hemolysin
K11019	0	0	2.2947E-08	6.74424E-08	thermostable direct hemolysin
K11020	0	0	1.23678E-07	6.75205E-07	exotoxin A [EC:2.4.2.-]
K11021	3.03437E-07	7.87466E-07	2.76727E-06	6.9975E-06	insecticidal toxin complex protein TccC
K11022	1.15913E-06	1.1201E-08	7.1985E-07	8.54442E-08	structural toxin protein (hemagglutinin/hemolysin) RtxA
K11023	3.24109E-11	8.06128E-11	2.33404E-10	3.78634E-09	pertussis toxin subunit 1 [EC:2.4.2.-]
K11029	1.1988E-08	1.5312E-10	2.46363E-07	1.32216E-08	anthrax edema toxin adenylate cyclase [EC:4.6.1.1]
K11031	4.08856E-07	3.22279E-07	3.01569E-07	4.53781E-07	thiol-activated cytolysin
K11032	1.24386E-08	0	1.06454E-08	0	hemolysin II
K11033	0	0	4.48531E-09	0	non-hemolytic enterotoxin A
K11034	0	0	1.79412E-08	0	non-hemolytic enterotoxin B/C
K11035	0	0	2.24265E-08	0	hemolysin BL binding component
K11037	0	0	8.97061E-09	0	hemolysin BL lytic component L1
K11038	6.21931E-08	0	8.37401E-09	0	leukocidin/hemolysin toxin family protein
K11039	1.24386E-08	0	1.6748E-09	0	delta-hemolysin
K11040	1.56792E-06	0	5.28809E-06	3.92928E-08	staphylococcal enterotoxin
K11041	1.12328E-07	9.29982E-08	5.56012E-07	9.80601E-06	exfoliative toxin A/B
K11042	9.9509E-08	0	1.33984E-08	0	superantigen-like protein
K11045	2.52515E-06	1.30907E-06	2.77214E-05	1.74167E-07	cAMP factor
K11050	3.24544E-06	3.55811E-06	1.54845E-05	5.76679E-05	multidrug/hemolysin transport system ATP-binding protein
K11051	6.87721E-07	3.51819E-07	8.08708E-07	4.35092E-06	multidrug/hemolysin transport system permease protein
K11060	0	1.85547E-09	3.42385E-09	0	probable enterotoxin B
K11063	6.32701E-09	1.3045E-08	1.70959E-09	2.2701E-08	toxin A/B
K11065	0.000217173	0.00021779	0.000206459	0.000129245	thiol peroxidase, atypical 2-Cys peroxiredoxin [EC:1.11.1.15]
K11066	3.71569E-06	3.23639E-06	7.26319E-06	5.92582E-05	N-acetylmuramoyl-L-alanine amidase [EC:3.5.1.28]
K11068	7.63437E-06	1.08252E-05	3.33936E-05	0.000104275	hemolysin III
K11069	7.45058E-06	4.10164E-06	1.84818E-05	9.43233E-05	spermidine/putrescine transport system substrate-binding protein
K11070	8.51708E-06	9.37577E-06	3.17264E-05	0.000103522	spermidine/putrescine transport system permease protein
K11071	7.23394E-06	5.70534E-06	2.00448E-05	7.25524E-05	spermidine/putrescine transport system permease protein
K11072	0.000690071	0.000699441	0.000785224	0.000886856	spermidine/putrescine transport system ATP-binding protein [EC:3.6.3.31]
K11073	3.78027E-06	3.08621E-06	1.41519E-05	6.48552E-05	putrescine transport system substrate-binding protein
K11074	4.45272E-06	2.91728E-06	9.05229E-06	5.98098E-05	putrescine transport system permease protein
K11075	4.40074E-06	3.73864E-06	9.7062E-06	6.00369E-05	putrescine transport system permease protein
K11076	1.34062E-05	1.54239E-05	6.28625E-05	0.000200568	putrescine transport system ATP-binding protein
K11077	5.99401E-09	1.1455E-07	1.08453E-07	0	mannopine transport system substrate-binding protein
K11078	4.4018E-09	1.19236E-07	1.12899E-07	0	mannopine transport system permease protein
K11079	5.22186E-07	2.22656E-07	2.85734E-07	1.21244E-07	mannopine transport system permease protein

K11080	4.01555E-07	4.72464E-07	1.35876E-06	1.55661E-05	mannopine transport system ATP-binding protein
K11081	1.27377E-07	3.76435E-07	2.11013E-07	2.14801E-06	2-aminoethylphosphonate transport system substrate-binding protein
K11082	1.5445E-07	3.57137E-07	1.70556E-07	2.14791E-06	2-aminoethylphosphonate transport system permease protein
K11083	1.25991E-07	3.57137E-07	1.85021E-07	2.1497E-06	2-aminoethylphosphonate transport system permease protein
K11084	0.000214676	0.000213849	0.000190733	0.000116761	2-aminoethylphosphonate transport system ATP-binding protein
K11085	0.000474982	0.000478516	0.000550458	0.000727951	ATP-binding cassette, subfamily B, bacterial MsbA [EC:3.6.3.-]
K11089	1.3363E-06	2.33925E-07	2.03483E-06	2.06561E-05	60 kDa SS-A/Ro ribonucleoprotein
K11102	0.000222674	0.000222982	0.000236902	0.000341085	proton glutamate symport protein
K11103	0.000869338	0.000864195	0.000812447	0.000697048	aerobic C4-dicarboxylate transport protein
K11104	5.7666E-07	3.26018E-07	3.26506E-06	1.55132E-06	melibiose permease
K11105	1.54562E-06	2.18531E-06	1.25593E-05	1.75344E-05	cell volume regulation protein A
K11106	6.17094E-07	9.80997E-07	3.16341E-06	1.71915E-05	L-tartrate/succinate antiporter
K11107	1.47486E-06	4.07226E-07	1.14703E-06	1.76968E-05	ferredoxin
K11131	4.44556E-08	0	1.17963E-07	2.41686E-08	H/ACA ribonucleoprotein complex subunit 4 [EC:5.4.99.-]
K11139	4.74107E-09	1.50671E-08	3.41424E-08	2.01885E-07	hemolysin E
K11144	1.30797E-06	3.45441E-07	6.82705E-06	3.65927E-06	primosomal protein DnaI
K11145	2.21222E-06	8.22564E-07	7.57975E-06	3.69801E-06	ribonuclease III family protein [EC:3.1.26.-]
K11159	0.000215232	0.000212395	0.000187452	0.000137112	carotenoid cleavage dioxygenase
K11173	1.09738E-07	1.73881E-07	3.54483E-07	4.22395E-07	hydroxyacid-oxoacid transhydrogenase [EC:1.1.99.24]
K11175	0.000223593	0.000224418	0.000229459	0.000235702	phosphoribosylglycinamide formyltransferase 1 [EC:2.1.2.2]
K11176	0	1.66614E-09	2.58094E-09	6.76627E-07	IMP cyclohydrolase [EC:3.5.4.10]
K11177	1.13775E-05	7.94428E-06	3.85335E-05	0.000105237	xanthine dehydrogenase YagR molybdenum-binding subunit [EC:1.17.1.4]
K11178	9.09059E-06	6.11403E-06	2.9532E-05	8.84249E-05	xanthine dehydrogenase YagS FAD-binding subunit [EC:1.17.1.4]
K11179	1.39224E-06	7.60283E-07	2.38915E-06	1.77482E-05	tRNA 2-thiouridine synthesizing protein E [EC:2.8.1.-]
K11180	0	0	6.27943E-08	0	dissimilatory sulfite reductase alpha subunit [EC:1.8.99.5]
K11181	0	0	2.09314E-08	0	dissimilatory sulfite reductase beta subunit [EC:1.8.99.5]
K11183	0.000433399	0.000430272	0.000405807	0.000374613	phosphocarrier protein FPr
K11184	9.36418E-07	7.59391E-07	1.71512E-06	8.57071E-07	catabolite repression HPr-like protein
K11189	0.000439386	0.000435025	0.000446036	0.000367809	phosphocarrier protein
K11191	1.47203E-06	4.03706E-07	8.41608E-06	9.7832E-06	PTS system, N-acetylmuramic acid-specific IIB component [EC:2.7.1.192]
K11192	1.47203E-06	4.03706E-07	8.41608E-06	9.7832E-06	PTS system, N-acetylmuramic acid-specific IIC component
K11194	0	0	0	1.09397E-07	PTS system, fructose-specific IIA component [EC:2.7.1.202]
K11195	0	0	0	1.31268E-07	PTS system, fructose-specific IIB component [EC:2.7.1.202]
K11196	0	1.02051E-08	2.75737E-10	1.26502E-07	PTS system, fructose-specific IIC component
K11198	9.92722E-07	1.46593E-07	1.17023E-07	2.49461E-06	PTS system, 2-O-A-mannosyl-D-glycerate-specific IIA component [EC:2.7.1.195]
K11199	9.92722E-07	1.46593E-07	1.17023E-07	2.49461E-06	PTS system, 2-O-A-mannosyl-D-glycerate-specific IIB component [EC:2.7.1.195]
K11200	9.92722E-07	1.46593E-07	1.17023E-07	2.49461E-06	PTS system, 2-O-A-mannosyl-D-glycerate-specific IIC component
K11201	1.80776E-07	2.19543E-07	1.48345E-06	7.36434E-06	PTS system, fructose-specific IIA-like component [EC:2.7.1.-]
K11202	8.35338E-07	7.486E-07	7.51178E-06	1.6222E-05	PTS system, fructose-specific IIB-like component [EC:2.7.1.-]
K11203	4.10126E-06	1.00798E-06	1.93378E-05	2.2171E-05	PTS system, fructose-specific IIC-like component
K11206	0.000221343	0.000222874	0.000221575	0.000199983	deaminated glutathione amidase [EC:3.5.1.128]
K11208	1.31264E-07	3.65181E-07	6.41643E-07	2.65872E-06	GST-like protein
K11209	0.000229106	0.000228226	0.000257551	0.000354151	GSH-dependent disulfide-bond oxidoreductase [EC:1.8.4.-]
K11210	9.00751E-07	1.09386E-09	2.69983E-06	3.35364E-08	metallothiol transferase [EC:2.5.1.-]
K11211	1.11807E-06	1.29595E-06	2.16412E-06	6.82486E-05	3-deoxy-D-manno-octulosonic acid kinase [EC:2.7.1.166]
K11212	3.56108E-07	4.25778E-07	8.89771E-07	3.83445E-06	LPPG:FO 2-phospho-L-lactate transferase [EC:2.7.8.28]
K11216	1.53606E-07	3.17269E-07	1.77839E-07	7.21522E-07	autoinducer-2 kinase [EC:2.7.1.189]
K11249	7.21303E-08	1.5965E-07	1.58586E-06	4.32403E-06	cysteine/O-acetylserine efflux protein
K11250	0.000218856	0.000220386	0.000226389	0.000255669	leucine efflux protein
K11258	1.09847E-06	8.71926E-07	1.97394E-06	6.85371E-05	acetolactate synthase II small subunit [EC:2.2.1.6]
K11261	3.33977E-07	3.15744E-07	2.22117E-07	3.51808E-06	formylmethanofuran dehydrogenase subunit E [EC:1.2.7.12]
K11263	0.000449176	0.000459009	0.00049767	0.000510656	acetyl-CoA/propionyl-CoA carboxylase, biotin carboxylase, biotin carboxyl carrier protein [EC:6.4.1.2 6.4.1.3 6.3.4.14
K11264	8.81078E-07	2.80854E-06	4.09893E-07	3.9681E-07	methylmalonyl-CoA decarboxylase [EC:4.1.1.41]
K11311	1.01104E-09	1.5945E-08	2.42501E-07	4.82261E-08	anthranilate 1,2-dioxygenase reductase component [EC:1.18.1.-]
K11312	1.34732E-06	9.11506E-07	1.77123E-06	1.59024E-05	cupin 2 domain-containing protein
K11325	0	0	9.0419E-08	0	L-cysteine/cystine lyase
K11326	1.2933E-06	9.32167E-07	2.46274E-06	2.70786E-07	cation efflux system protein involved in nickel and cobalt tolerance
K11328	9.98889E-07	6.79991E-08	1.34655E-07	6.53775E-08	two-component system, OmpR family, Ni(II)-sensor and/or redox sensor kinase NrsS [EC:2.7.13.3]
K11329	0.000214044	0.000211885	0.000189128	0.000114631	two-component system, OmpR family, response regulator RpaB
K11330	1.34865E-08	2.23685E-08	1.5325E-07	8.74403E-08	two-component system, OmpR family, Ni(II)-responsive and/or redox-responsive regulator NrsR
K11332	4.48335E-09	3.89849E-07	1.33553E-06	5.57305E-08	two-component system, OmpR family, response regulator NblR
K11333	9.7076E-07	2.17504E-07	9.50482E-08	1.29352E-07	3,8-divinyl chlorophyllide a/chlorophyllide a reductase subunit X [EC:1.3.7.14 1.3.7.15]
K11334	9.7076E-07	1.11305E-08	8.07648E-08	1.22569E-07	3,8-divinyl chlorophyllide a/chlorophyllide a reductase subunit Y [EC:1.3.7.14 1.3.7.15]
K11335	9.7076E-07	1.11305E-08	8.07648E-08	1.22569E-07	3,8-divinyl chlorophyllide a/chlorophyllide a reductase subunit Z [EC:1.3.7.14 1.3.7.15]
K11336	9.7076E-07	1.11305E-08	8.07648E-08	1.22569E-07	3-vinyl bacteriochlorophyllide hydratase [EC:4.2.1.165]
K11337	9.7076E-07	1.11305E-08	1.02978E-07	1.22569E-07	bacteriochlorophyllide a dehydrogenase [EC:1.1.1.396]
K11354	1.16473E-06	3.93389E-07	3.8044E-06	4.72201E-07	two-component system, chemotaxis family, sensor kinase Cph1 [EC:2.7.13.3]
K11355	3.5169E-08	2.53065E-07	5.35412E-07	4.42901E-08	two-component system, chemotaxis family, response regulator Rcp1
K11356	1.2521E-05	9.72252E-06	4.4018E-05	0.000240891	two-component system, sensor histidine kinase and response regulator [EC:2.7.13.3]
K11357	0.000230642	0.000228096	0.000227554	0.000360819	two-component system, cell cycle sensor histidine kinase DivJ [EC:2.7.13.3]
K11358	1.14521E-06	1.10459E-06	1.49033E-06	2.48553E-06	aspartate aminotransferase [EC:2.6.1.1]
K11381	3.58106E-06	4.3128E-06	6.74747E-06	5.73372E-05	2-oxoisovalerate dehydrogenase E1 component [EC:1.2.4.4]
K11382	8.11233E-07	5.96871E-10	2.67506E-06	1.25715E-07	MFS transporter, OPA family, phosphoglycerate transporter protein
K11383	1.11321E-06	3.5688E-06	2.56527E-06	1.27344E-06	two-component system, NtrC family, sensor histidine kinase KinB [EC:2.7.13.3]
K11384	1.02548E-05	1.45682E-05	6.63969E-05	0.000185448	two-component system, NtrC family, response regulator AlgB
K11385	0	4.41244E-07	1.95316E-06	4.61337E-06	arabinosyltransferase A [EC:2.4.2.-]
K11386	0	4.91404E-07	3.35002E-06	5.2406E-06	arabinosyltransferase B [EC:2.4.2.-]
K11387	1.7545E-06	8.9135E-07	9.17894E-06	5.70552E-06	arabinosyltransferase C [EC:2.4.2.-]
K11391	1.11929E-06	3.50971E-06	2.10132E-06	1.72914E-05	23S rRNA (guanine1835-N2)-methyltransferase [EC:2.1.1.174]
K11392	8.07012E-07	1.66755E-06	6.20294E-06	1.99467E-05	16S rRNA (cytosine1407-C5)-methyltransferase [EC:2.1.1.178]
K11410	2.74029E-06	7.05945E-06	1.59692E-05	3.56168E-05	short/branched chain acyl-CoA dehydrogenase [EC:1.3.99.12]
K11418	0	0	5.73309E-08	4.50627E-06	histone deacetylase 11 [EC:3.5.1.98]
K11434	0	0	1.15321E-07	4.15743E-08	type I protein arginine methyltransferase [EC:2.1.1.319]
K11440	4.4784E-07	4.20351E-07	4.64649E-07	3.92079E-07	choline dehydrogenase [EC:1.1.1.1]
K11441	1.73675E-07	5.40937E-08	9.8178E-07	4.00902E-07	dehydrogluconokinase [EC:2.7.1.13]
K11442	9.36655E-07	4.33197E-08	2.79533E-06	3.03626E-08	putative uridylyltransferase [EC:2.7.7.-]
K11443	7.07716E-06	7.61662E-06	2.98575E-05	0.000113818	two-component system, cell cycle response regulator DivK

K11444	0.000652991	0.000652968	0.000644072	0.000659659	two-component system, chemotaxis family, response regulator WspR [EC:2.7.7.65]
K11472	0.000216716	0.000215736	0.000205076	0.000150559	glycolate oxidase FAD binding subunit
K11473	0.000217671	0.000218693	0.000205628	0.000148619	glycolate oxidase iron-sulfur subunit
K11474	3.72758E-07	1.25815E-06	1.96981E-06	7.80153E-06	GntR family transcriptional regulator, glc operon transcriptional activator
K11475	0.000228397	0.000231533	0.000252339	0.000304792	GntR family transcriptional regulator, vanillate catabolism transcriptional regulator
K11476	1.43582E-06	4.78013E-07	3.53613E-06	3.38412E-07	GntR family transcriptional regulator, gluconate operon transcriptional repressor
K11477	0.000216909	0.000218889	0.000218436	0.000184781	glc operon protein GlcG
K11520	1.27683E-06	2.64872E-06	2.61856E-06	7.58215E-06	two-component system, OmpR family, manganese sensing sensor histidine kinase [EC:2.7.13.3]
K11521	3.39613E-06	5.19796E-06	1.93717E-05	0.000144552	two-component system, OmpR family, manganese sensing response regulator
K11525	0.000214678	0.00021319	0.000194232	0.000181375	methyl-accepting chemotaxis protein PixJ
K11526	0	1.17924E-09	6.97714E-09	8.67846E-08	two-component system, chemotaxis family, sensor histidine kinase and response regulator PixL
K11527	1.40354E-05	2.22962E-05	6.2362E-05	0.00024853	two-component system, sensor histidine kinase and response regulator [EC:2.7.13.3]
K11528	9.51396E-08	8.3163E-08	4.47456E-07	2.86559E-06	UDP-N-acetylglucosamine pyrophosphorylase [EC:2.7.7.23]
K11529	0.000430512	0.000427545	0.000391249	0.00026285	glycerate 2-kinase [EC:2.7.1.165]
K11530	9.79007E-08	3.13043E-07	8.23183E-08	7.08724E-07	(4S)-4-hydroxy-5-phosphonooxypentane-2,3-dione isomerase [EC:5.3.1.32]
K11531	6.13386E-08	2.35813E-08	5.48749E-08	1.26351E-06	lsr operon transcriptional repressor
K11532	2.53256E-06	3.26341E-06	2.39583E-06	7.60269E-06	fructose-1,6-bisphosphatase II / sedoheptulose-1,7-bisphosphatase [EC:3.1.3.11 3.1.3.37]
K11533	1.20197E-07	6.40517E-07	1.83182E-06	3.59228E-06	fatty acid synthase, bacteria type [EC:2.3.1.-]
K11534	3.91437E-07	9.1463E-07	6.07657E-07	9.02475E-06	DeoR family transcriptional regulator, deoxyribose operon repressor
K11535	1.27629E-06	7.30489E-07	3.78125E-06	7.54459E-06	nucleoside transport protein
K11537	1.23958E-06	2.34498E-06	6.01319E-06	5.10171E-05	MFS transporter, NHS family, xanthosine permease
K11600	0	0	2.62525E-08	0	exosome complex component RRP41
K11601	1.80894E-07	6.39211E-07	7.4238E-07	4.16376E-06	manganese transport system substrate-binding protein
K11602	8.69292E-07	1.26664E-06	7.07272E-06	7.47616E-06	manganese transport system permease protein
K11603	6.97075E-07	9.63279E-07	2.29457E-06	7.1035E-06	manganese transport system ATP-binding protein
K11604	6.64119E-07	9.61092E-07	8.75934E-07	4.32882E-06	manganese/iron transport system substrate-binding protein
K11605	2.74735E-07	9.26053E-07	7.27871E-07	4.37345E-06	manganese/iron transport system permease protein
K11606	4.19401E-07	9.61323E-07	1.07037E-06	4.38422E-06	manganese/iron transport system permease protein
K11607	1.4635E-06	1.71067E-06	9.37394E-06	6.41184E-06	manganese/iron transport system ATP-binding protein
K11608	9.42487E-08	4.7547E-07	3.50815E-07	1.9898E-06	beta-ketoacyl-[acyl-carrier-protein] synthase III [EC:2.3.1.180]
K11609	1.65311E-07	4.6297E-07	1.64676E-06	8.16385E-06	beta-ketoacyl ACP synthase [EC:2.3.1.-]
K11610	2.12934E-06	4.29802E-06	8.32517E-06	4.51571E-05	beta-ketoacyl ACP reductase [EC:1.1.1.100]
K11611	0	2.76207E-07	3.81909E-07	1.81217E-06	enoyl ACP reductase [EC:1.3.1.9]
K11614	6.69196E-07	9.04556E-07	1.10522E-06	3.20883E-06	two-component system, CitB family, sensor histidine kinase MalK [EC:2.7.13.3]
K11615	1.60276E-06	3.21403E-06	1.8009E-06	9.01337E-06	two-component system, CitB family, response regulator MalR
K11616	1.55429E-06	8.75084E-07	1.38396E-06	2.6161E-06	malate:Na+ symporter
K11617	1.24239E-06	1.20918E-06	5.76E-06	9.8002E-06	two-component system, NarL family, sensor histidine kinase LiaS [EC:2.7.13.3]
K11618	7.82401E-06	1.07541E-05	3.45005E-05	9.49972E-05	two-component system, NarL family, response regulator LiaR
K11619	5.54378E-08	2.06813E-07	5.60616E-07	0	lia operon protein LiaI
K11621	0	0	5.15201E-08	0	lia operon protein LiaG
K11622	1.53302E-07	2.06813E-07	8.45663E-07	6.66011E-07	lia operon protein LiaF
K11623	0	3.27506E-09	1.19305E-06	1.83217E-08	two-component system, NarL family, sensor histidine kinase YdfH [EC:2.7.13.3]
K11624	5.18383E-07	9.61075E-07	1.31303E-06	2.2521E-06	two-component system, NarL family, response regulator YdfI
K11625	7.81318E-07	5.71315E-07	6.81898E-06	2.01283E-06	membrane protein YdfJ
K11626	1.66997E-06	1.67798E-06	5.46909E-06	9.2152E-06	putative sodium/glutamine symporter
K11628	3.87908E-09	1.65264E-07	8.00458E-07	1.27856E-07	mycocerosic acid synthase [EC:2.3.1.111]
K11629	1.78368E-06	6.68594E-07	6.98092E-06	7.61805E-07	two-component system, OmpR family, bacitracin resistance sensor histidine kinase BceS [EC:2.7.13.3]
K11630	1.86873E-06	5.0993E-07	6.92926E-06	3.16271E-06	two-component system, OmpR family, bacitracin resistance response regulator BceR
K11631	3.78727E-06	7.87497E-07	1.54067E-05	1.82239E-06	bacitracin transport system ATP-binding protein
K11632	1.90666E-06	4.39422E-10	5.96158E-06	6.07252E-08	bacitracin transport system permease protein
K11633	5.89134E-08	1.02051E-08	1.83488E-07	2.28005E-10	two-component system, OmpR family, sensor histidine kinase YxdK [EC:2.7.13.3]
K11634	1.3369E-07	1.03898E-08	3.25325E-07	7.61882E-07	two-component system, OmpR family, response regulator YxdJ
K11635	3.47967E-06	1.50331E-06	9.02653E-06	1.44429E-05	putative ABC transport system ATP-binding protein
K11636	2.06745E-07	2.22152E-09	2.08144E-07	5.03372E-07	putative ABC transport system permease protein
K11637	3.69744E-07	1.65185E-07	9.77499E-07	9.5886E-06	two-component system, CitB family, sensor histidine kinase CitS [EC:2.7.13.3]
K11638	9.00786E-08	9.5208E-10	7.22714E-07	5.52795E-06	two-component system, CitB family, response regulator CitT
K11639	1.37862E-07	8.50664E-11	1.64594E-07	1.4404E-08	Mg2+/citrate complex secondary transporter
K11640	0	0	2.33356E-08	0	two-component system, LytTR family, sensor histidine kinase NatK [EC:2.7.13.3]
K11641	0	0	2.33356E-08	0	two-component system, LytTR family, response regulator NatR
K11645	1.48302E-06	1.74377E-06	4.48458E-06	7.77745E-06	fructose-bisphosphate aldolase, class I [EC:4.1.2.13]
K11646	2.4135E-07	5.88797E-07	3.03008E-06	6.94811E-06	3-dehydroquinate synthase II [EC:1.4.1.24]
K11685	5.38575E-08	4.25328E-08	4.89195E-08	7.90843E-07	DNA-binding protein StpA
K11686	1.20362E-10	2.06967E-07	6.01262E-08	0	chromosome-anchoring protein RacA
K11688	4.27921E-06	4.80579E-06	2.25597E-05	4.85136E-05	C4-dicarboxylate-binding protein DctP
K11689	1.47471E-06	2.90758E-06	1.3062E-05	4.02393E-05	C4-dicarboxylate transporter, DctQ subunit
K11690	1.03338E-05	1.12021E-05	6.26453E-05	0.000141323	C4-dicarboxylate transporter, DctM subunit
K11691	2.93736E-07	6.59106E-09	1.36467E-07	4.96073E-07	two-component system, CitB family, sensor histidine kinase DctS [EC:2.7.13.3]
K11692	1.60627E-06	3.04657E-06	1.1354E-06	4.21405E-07	two-component system, CitB family, response regulator DctR
K11693	9.68623E-07	0	3.06486E-06	3.41661E-08	peptidoglycan pentaglycine glycine transferase (the first glycine) [EC:2.3.2.16]
K11694	2.05135E-06	3.97012E-08	5.65141E-06	1.20531E-07	peptidoglycan pentaglycine glycine transferase (the second and third glycine) [EC:2.3.2.17]
K11695	1.86489E-06	1.63753E-08	8.25664E-06	7.22069E-08	peptidoglycan pentaglycine glycine transferase (the fourth and fifth glycine) [EC:2.3.2.18]
K11704	9.26681E-08	7.94201E-08	2.27733E-07	3.38887E-06	iron/zinc/manganese/copper transport system substrate-binding protein
K11705	9.19359E-08	8.03722E-08	2.94387E-07	2.90389E-06	iron/zinc/manganese/copper transport system permease protein
K11706	1.1827E-07	9.34701E-07	2.84028E-07	2.51511E-06	iron/zinc/manganese/copper transport system ATP-binding protein
K11707	1.01114E-07	6.44783E-07	1.51041E-06	6.11984E-07	manganese/zinc/iron transport system substrate-binding protein
K11708	1.0336E-07	3.20389E-07	1.35984E-06	3.84668E-06	manganese/zinc/iron transport system permease protein
K11709	1.71947E-06	3.90839E-06	7.4815E-06	7.32515E-06	manganese/zinc/iron transport system permease protein
K11710	1.67801E-06	2.07474E-06	1.82169E-05	1.0858E-05	manganese/zinc/iron transport system ATP- binding protein
K11711	0.000220634	0.000226783	0.000249993	0.000278004	two-component system, LuxR family, sensor histidine kinase DctS [EC:2.7.13.3]
K11712	0.000220628	0.000223145	0.000243114	0.000293601	two-component system, LuxR family, response regulator DctR
K11717	1.0291E-05	1.37587E-05	4.54421E-05	0.000139214	cysteine desulfurase / selenocysteine lyase [EC:2.8.1.7 4.4.1.16]
K11719	0.000219318	0.000217083	0.000207825	0.000217599	lipopolysaccharide export system protein LptC
K11720	0.000219809	0.000219137	0.000210768	0.000217816	lipopolysaccharide export system permease protein
K11731	0.000218393	0.000221337	0.000224683	0.000196899	citronellyl-CoA dehydrogenase [EC:1.3.99.-]
K11732	3.99355E-07	9.13391E-07	1.59934E-06	9.01787E-07	phenylalanine-specific permease
K11733	0.000657692	0.000664375	0.000690077	0.000566383	lysine-specific permease
K11734	0.000227314	0.000231154	0.000291812	0.000258092	aromatic amino acid transport protein AroP

K11735	0.000227126	0.000234935	0.000300388	0.000371008	GABA permease
K11736	0.000214619	0.000213298	0.000192751	0.000116312	proline-specific permease ProY
K11737	1.51474E-05	1.95655E-05	0.000118831	0.000230438	D-serine/D-alanine/glycine transporter
K11738	0.000227289	0.000233342	0.000293455	0.000323342	L-asparagine permease
K11739	4.35147E-09	1.94507E-08	8.41281E-08	2.07961E-07	bacteriophage N4 adsorption protein A
K11740	0.000215427	0.000215027	0.000201987	0.000172424	bacteriophage N4 adsorption protein B
K11741	1.0982E-05	1.34859E-05	4.76075E-05	0.000195845	quaternary ammonium compound-resistance protein SugE
K11742	7.00389E-07	6.96134E-07	1.68891E-06	4.44623E-05	spermidine export protein MdtI
K11743	7.00389E-07	6.96134E-07	1.93528E-06	4.45103E-05	spermidine export protein MdtJ
K11744	2.33713E-07	5.68027E-07	3.23358E-07	6.37477E-06	AI-2 transport protein TqsA
K11745	0.000217178	0.000219349	0.00021825	0.000231652	glutathione-regulated potassium-efflux system ancillary protein KefC
K11746	0.000214334	0.000213612	0.000189589	0.000119265	glutathione-regulated potassium-efflux system ancillary protein KefF
K11747	0.00022226	0.000221003	0.000222979	0.000308714	glutathione-regulated potassium-efflux system protein KefB
K11748	0.000214364	0.000213755	0.000187301	0.000130453	glutathione-regulated potassium-efflux system ancillary protein KefG
K11749	0.000442741	0.000442009	0.000448208	0.000427428	regulator of sigma E protease [EC:3.4.24.-]
K11750	9.82053E-08	3.28143E-07	3.32081E-07	1.4235E-05	esterase FrsA [EC:3.1.-.-]
K11751	2.95688E-06	4.49836E-06	2.36173E-05	5.89511E-05	5-nucleotidase / UDP-sugar diphosphatase [EC:3.1.3.5 3.6.1.45]
K11752	0.000224623	0.00022591	0.000227576	0.000226805	diaminohydroxyphosphoribosylaminopyrimidine deaminase / 5-amino-6-(5-phosphoribosylamino)uracil reductase [EC
K11753	0.000223772	0.00022388	0.000229807	0.00022574	riboflavin kinase / FMN adenylyltransferase [EC:2.7.1.26 2.7.7.2]
K11754	0.000224919	0.000226875	0.00023151	0.000229437	dihydrofolate synthase / folylpolyglutamate synthase [EC:6.3.2.12 6.3.2.17]
K11755	9.73011E-06	1.0505E-05	4.07439E-05	0.000105617	phosphoribosyl-ATP pyrophosphohydrolase / phosphoribosyl-AMP cyclohydrolase [EC:3.6.1.31 3.5.4.19]
K11777	9.16819E-09	0	2.09314E-08	0	HAD superfamily phosphatase
K11779	2.16888E-07	1.47151E-07	1.08141E-06	3.88687E-06	FO synthase [EC:2.5.1.77]
K11780	7.94338E-09	1.85931E-08	5.20289E-07	3.66114E-06	FO synthase subunit 1 [EC:2.5.1.77]
K11781	2.16888E-07	1.42535E-07	1.399E-06	2.00211E-06	FO synthase subunit 2 [EC:2.5.1.77]
K11782	4.23211E-08	9.5208E-10	8.31349E-07	1.70981E-08	chorismate dehydratase [EC:4.2.1.151]
K11783	6.23025E-09	9.5208E-10	1.93949E-07	3.36064E-08	futalosine hydrolase [EC:3.2.2.26]
K11784	6.81371E-08	9.5208E-10	1.75765E-06	9.00643E-08	cyclic dehypoxanthinyl futalosine synthase [EC:1.21.98.1]
K11785	4.23211E-08	9.5208E-10	7.93688E-07	1.70981E-08	1,4-dihydroxy-6-naphthoate synthase [EC:1.14.-.-]
K11811	6.40497E-06	5.20815E-06	1.96826E-05	9.55144E-05	arsenical resistance protein ArsH
K11814	2.47616E-07	5.31567E-09	4.61011E-08	8.5633E-09	multidrug resistance protein EbrA
K11815	1.15412E-07	2.4421E-08	1.48921E-07	1.20694E-07	multidrug resistance protein EbrB
K11816	3.97169E-09	4.03924E-08	1.43119E-07	5.88382E-08	indole-3-pyruvate monooxygenase [EC:1.14.13.168]
K11889	0	0	9.38355E-09	0	type VI secretion system protein ImpN [EC:2.7.11.1]
K11890	0.0002155	0.000215999	0.000204289	0.000165477	type VI secretion system protein ImpM
K11891	0.000215819	0.000216347	0.000205766	0.00018276	type VI secretion system protein ImpL
K11892	0.000431365	0.000433786	0.000412105	0.000337188	type VI secretion system protein ImpK
K11893	0.00021591	0.000216336	0.000205447	0.000182587	type VI secretion system protein ImpJ
K11894	2.50534E-07	1.48368E-07	9.5431E-07	1.40908E-05	type VI secretion system protein ImpI
K11895	0.000215916	0.000216307	0.000205317	0.000182387	type VI secretion system protein ImpH
K11896	0.000215929	0.000216361	0.0002056	0.000182745	type VI secretion system protein ImpG
K11897	0.000215636	0.000216186	0.000204424	0.000171748	type VI secretion system protein ImpF
K11898	1.38235E-06	3.28521E-06	1.66228E-05	3.95428E-05	type VI secretion system protein ImpE
K11899	0.000213766	0.000211788	0.00018625	0.000122308	type VI secretion system protein ImpD
K11900	0.000215769	0.000216308	0.000205302	0.000185224	type VI secretion system protein ImpC
K11901	0.000215759	0.000216277	0.000205184	0.000182468	type VI secretion system protein ImpB
K11902	0.000215639	0.000216228	0.000205059	0.000171797	type VI secretion system protein ImpA
K11903	0.000215988	0.000216582	0.000206094	0.000201098	type VI secretion system secreted protein Hcp
K11904	0.001720012	0.001713181	0.001594701	0.001263121	type VI secretion system secreted protein VgrG
K11905	2.16543E-07	9.64829E-08	8.16834E-07	1.05906E-05	type VI secretion system protein
K11906	0.000215581	0.000216197	0.000204819	0.000180952	type VI secretion system protein VasD
K11907	0.000439374	0.000444025	0.000438179	0.000362522	type VI secretion system protein VasG
K11908	1.97927E-06	8.1434E-07	6.27863E-06	2.02478E-05	sigma-54 dependent transcriptional regulator
K11909	1.17177E-08	5.86175E-08	1.94854E-07	9.56482E-06	type VI secretion system protein VasI
K11910	2.71971E-07	7.36067E-07	8.15467E-07	1.05341E-05	type VI secretion system protein VasJ
K11911	2.99327E-08	3.69187E-08	2.65968E-07	1.03432E-05	type VI secretion system protein VasL
K11912	5.52286E-07	1.38696E-06	4.79647E-06	6.05429E-06	serine/threonine-protein kinase PpkA [EC:2.7.11.1]
K11913	1.26128E-07	6.6682E-08	6.67678E-07	2.58057E-08	type VI secretion system protein
K11914	5.10152E-06	8.72852E-06	3.52443E-05	0.0001205	sigma-54 dependent transcriptional regulator
K11915	2.26022E-07	1.30187E-07	1.23992E-06	4.43738E-06	serine/threonine protein phosphatase Stp1 [EC:3.1.3.16]
K11916	1.64513E-07	6.58516E-08	5.16162E-07	1.69867E-07	serine/threonine-protein kinase Stk1 [EC:2.7.11.-]
K11917	0.000217788	0.000215332	0.000208183	0.000155116	sigma-54 dependent transcriptional regulator
K11918	0	2.29077E-09	9.26445E-08	3.34137E-09	type VI secretion system protein
K11919	0	1.11153E-09	3.73814E-08	3.34137E-09	type VI secretion system lysozyme-related protein
K11920	8.24529E-09	3.12662E-08	5.93778E-08	3.73788E-07	AraC family transcriptional regulator
K11921	0.000229786	0.000242915	0.000334168	0.000682169	LysR family transcriptional regulator, cyn operon transcriptional activator
K11922	1.33465E-08	2.82302E-08	1.79545E-07	2.45679E-06	GntR family transcriptional regulator, mannosyl-D-glycerate transport/metabolism system repressor
K11923	4.10304E-09	5.46146E-07	6.22979E-07	8.8303E-08	MerR family transcriptional regulator, copper efflux regulator
K11924	1.95231E-06	7.90182E-07	1.67219E-06	5.2198E-05	DtxR family transcriptional regulator, manganese transport regulator
K11925	1.95274E-07	6.12655E-07	3.92894E-07	1.2245E-05	SgrR family transcriptional regulator
K11926	9.82474E-08	3.01092E-07	3.26527E-07	1.22825E-05	sigma factor-binding protein Crl
K11927	0.000661894	0.000666325	0.000675758	0.000743596	ATP-dependent RNA helicase RhlE [EC:3.6.4.13]
K11928	6.09048E-06	1.15267E-05	2.50359E-05	5.44809E-05	sodium/proline symporter
K11929	5.41098E-07	1.70343E-06	4.56992E-07	4.9253E-06	outer membrane pore protein E
K11930	8.38839E-09	7.13809E-08	6.68494E-07	9.8968E-06	periplasmic protein TorT
K11931	0.000215809	0.000213981	0.000190322	0.000217083	poly-beta-1,6-N-acetyl-D-glucosamine N-deacetylase [EC:3.5.1.-]
K11932	2.09108E-07	5.97366E-07	1.31253E-07	1.48471E-06	universal stress protein G
K11933	2.33096E-07	3.32888E-07	1.24649E-06	1.14877E-05	NADH oxidoreductase Hcr [EC:1.-.-.-]
K11934	9.90612E-08	3.02941E-07	8.67661E-08	8.18461E-07	outer membrane protein X
K11935	8.60799E-07	7.42946E-07	1.66487E-06	5.22207E-05	biofilm PGA synthesis protein PgaA
K11936	0.000215644	0.000214198	0.000190835	0.000194515	poly-beta-1,6-N-acetyl-D-glucosamine synthase [EC:2.4.1.-]
K11937	9.71551E-07	7.82808E-07	1.78293E-06	6.33439E-05	biofilm PGA synthesis protein PgaD
K11938	1.57065E-07	4.47373E-07	2.88899E-06	1.87425E-06	HMP-PP phosphatase [EC:3.6.1.-]
K11939	0.000219439	0.000219811	0.000214	0.000219384	inner membrane transporter RhtA
K11940	1.64728E-06	5.00496E-07	6.5725E-07	9.71461E-07	heat shock protein HspQ
K11941	1.71195E-07	3.30679E-07	1.08214E-06	1.64004E-06	glucans biosynthesis protein C [EC:2.1.-.-]
K11942	0.000214452	0.000214427	0.000188184	0.000113506	isobutyryl-CoA mutase [EC:5.4.99.13]

K11943	0	2.12879E-08	2.61643E-08	3.14668E-08	PAH dioxygenase large subunit [EC:1.13.11.-]
K11944	0	2.12879E-08	0	2.42598E-08	PAH dioxygenase small subunit [EC:1.13.11.-]
K11945	0	2.12879E-08	2.32571E-09	1.74764E-08	extradiol dioxygenase [EC:1.13.11.-]
K11946	0	1.06439E-08	0	1.29542E-08	hydratase-aldolase [EC:4.1.2.-]
K11947	0	1.06439E-08	0	1.29542E-08	aldehyde dehydrogenase [EC:1.2.1.-]
K11948	0	1.06439E-08	2.79086E-08	2.64068E-08	1-hydroxy-2-naphthoate dioxygenase [EC:1.13.11.38]
K11949	2.40676E-07	5.96396E-07	2.77222E-06	6.95259E-06	4-(2-carboxyphenyl)-2-oxobut-3-enoate aldolase [EC:4.1.2.34]
K11950	1.51034E-08	1.09169E-07	2.47838E-07	1.11247E-07	bicarbonate transport system substrate-binding protein
K11951	1.23051E-07	1.21581E-07	1.06347E-07	4.40825E-08	bicarbonate transport system permease protein
K11952	1.55611E-06	3.03935E-06	1.58671E-05	3.80024E-05	bicarbonate transport system ATP-binding protein [EC:3.6.3.-]
K11953	0.000214147	0.000212006	0.000187493	0.000115282	bicarbonate transport system ATP-binding protein [EC:3.6.3.-]
K11954	6.98097E-10	8.92718E-10	1.04657E-08	0	neutral amino acid transport system substrate-binding protein
K11955	4.28073E-08	0	1.12795E-06	1.39348E-07	neutral amino acid transport system permease protein
K11956	4.82798E-09	1.38505E-08	3.60273E-07	7.14415E-09	neutral amino acid transport system permease protein
K11957	1.52555E-07	1.55179E-07	5.99376E-07	2.38233E-07	neutral amino acid transport system ATP-binding protein
K11958	1.0199E-06	1.36785E-07	7.60588E-07	7.93439E-08	neutral amino acid transport system ATP-binding protein
K11959	0.000225901	0.000218068	0.000214731	0.000217287	urea transport system substrate-binding protein
K11960	0.000221688	0.000216022	0.000207882	0.00015113	urea transport system permease protein
K11961	0.000221986	0.00021608	0.000208043	0.000152961	urea transport system permease protein
K11962	0.000436916	0.000428373	0.000399848	0.000266861	urea transport system ATP-binding protein
K11963	0.000657781	0.000647281	0.000608762	0.000440526	urea transport system ATP-binding protein
K11987	0	7.0625E-08	7.29361E-08	7.14415E-09	prostaglandin-endoperoxide synthase 2 [EC:1.14.99.1]
K11991	0.000446035	0.000451278	0.000480622	0.000489006	tRNA(adenine34) deaminase [EC:3.5.4.33]
K12048	0	0	9.54637E-09	0	ComB10 competence protein
K12049	0	0	9.54637E-09	0	ComB9 competence protein
K12050	0	0	9.54637E-09	0	ComB8 competence protein
K12053	0	2.3326E-08	9.54637E-09	0	ComB4 competence protein
K12055	1.20357E-06	2.59103E-06	1.29723E-05	3.00703E-05	chromosome partitioning related protein ParA
K12056	4.37324E-07	6.86727E-07	3.51547E-06	7.62546E-06	conjugal transfer mating pair stabilization protein TraG
K12057	3.23396E-07	6.57153E-07	3.08369E-06	7.62331E-06	conjugal transfer pilus assembly protein TraF
K12058	3.47876E-07	6.57286E-07	3.20306E-06	8.26659E-06	conjugal transfer mating pair stabilization protein TraN
K12059	3.15026E-07	6.55153E-07	3.00638E-06	7.1082E-06	conjugal transfer pilus assembly protein TrbC
K12060	3.23333E-07	6.56819E-07	3.07149E-06	7.57046E-06	conjugal transfer pilus assembly protein TraU
K12061	3.20893E-07	6.56698E-07	3.08134E-06	7.6004E-06	conjugal transfer pilus assembly protein TraW
K12062	4.77164E-07	6.43354E-07	3.03428E-06	7.63425E-06	conjugal transfer pilin signal peptidase TrbI
K12063	4.95902E-07	1.07304E-06	3.78464E-06	7.66091E-06	conjugal transfer ATP-binding protein TraC
K12064	3.58518E-07	6.56956E-07	3.08367E-06	7.57088E-06	conjugal transfer pilus assembly protein TraV
K12065	3.46363E-07	6.66904E-07	3.2711E-06	7.56957E-06	conjugal transfer pilus assembly protein TraB
K12066	3.42368E-07	6.60795E-07	2.86466E-06	7.61844E-06	conjugal transfer pilus assembly protein TraK
K12067	3.23637E-07	6.61029E-07	3.0831E-06	7.62526E-06	conjugal transfer pilus assembly protein TraE
K12068	3.21924E-07	6.60884E-07	3.03088E-06	7.61407E-06	conjugal transfer pilus assembly protein TraL
K12069	2.72793E-08	1.55304E-09	5.36081E-08	4.59897E-07	conjugal transfer pilus assembly protein TraA
K12070	3.1824E-07	6.92289E-07	3.584E-06	9.47097E-06	conjugal transfer pilus assembly protein TraI
K12071	1.46129E-06	3.16634E-06	1.5801E-05	3.72133E-05	conjugal transfer pilus assembly protein TraD
K12072	3.23566E-07	6.57577E-07	3.08491E-06	7.58443E-06	conjugative transfer pilus assembly protein TraH
K12073	0	0	1.04657E-08	0	1,4-dihydroxy-2-naphthoyl-CoA hydrolase [EC:3.1.2.28]
K12111	1.07515E-06	9.73475E-07	6.34846E-06	1.3135E-05	evolved beta-galactosidase subunit alpha [EC:3.2.1.23]
K12112	5.06704E-08	2.19624E-07	2.91729E-07	3.83126E-06	evolved beta-galactosidase subunit beta
K12113	6.20103E-07	4.81373E-07	5.77208E-06	6.83767E-06	LacI family transcriptional regulator, ebg operon repressor
K12132	2.2854E-05	3.9421E-05	0.000126944	0.000224648	eukaryotic-like serine/threonine-protein kinase [EC:2.7.11.1]
K12136	3.28686E-08	5.3078E-08	2.65737E-07	2.62639E-06	hydrogenase-4 component A [EC:1.-.-.-]
K12137	5.00209E-07	1.09884E-06	1.36841E-06	2.43159E-06	hydrogenase-4 component B [EC:1.-.-.-]
K12138	3.23418E-08	3.75686E-08	2.49761E-07	2.30592E-06	hydrogenase-4 component C [EC:1.-.-.-]
K12139	1.50897E-07	1.73469E-07	5.19935E-07	3.80514E-07	hydrogenase-4 component D [EC:1.-.-.-]
K12140	3.3874E-08	9.8301E-08	7.87872E-07	2.46067E-07	hydrogenase-4 component E [EC:1.-.-.-]
K12141	1.90458E-07	1.56736E-07	1.05363E-06	2.80296E-07	hydrogenase-4 component F [EC:1.-.-.-]
K12142	3.22279E-08	3.74308E-08	2.49808E-07	2.30611E-06	hydrogenase-4 component G [EC:1.-.-.-]
K12143	3.93242E-09	1.9204E-08	1.95262E-07	1.5984E-06	hydrogenase-4 component H
K12144	3.22182E-08	3.74066E-08	2.49738E-07	2.30582E-06	hydrogenase-4 component I [EC:1.-.-.-]
K12145	2.78404E-08	1.7885E-08	6.65772E-08	1.3421E-06	hydrogenase-4 component J [EC:1.-.-.-]
K12146	1.58641E-06	5.08551E-07	2.60468E-06	3.81971E-06	hydrogenase-4 transcriptional activator
K12147	9.81956E-08	3.00963E-07	7.48051E-08	7.53275E-07	acidic protein MsyB
K12148	9.82118E-08	3.01003E-07	8.55765E-08	8.2193E-07	biofilm regulator BssS
K12149	1.08003E-07	3.42333E-07	2.88444E-07	4.10811E-06	DNA-damage-inducible protein I
K12151	3.52297E-07	1.32398E-06	2.02912E-07	9.88684E-07	multiple stress resistance protein BhsA
K12152	0.000216473	0.000216922	0.000205815	0.000206699	phosphatase NudJ [EC:3.6.1.-]
K12203	5.46777E-07	5.02926E-08	2.08272E-07	3.89823E-07	defect in organelle trafficking protein DotB
K12204	5.46768E-07	5.02684E-08	2.08202E-07	4.74163E-08	defect in organelle trafficking protein DotC
K12205	3.4434E-09	2.83931E-08	1.04379E-08	4.62821E-08	defect in organelle trafficking protein DotD
K12206	5.46905E-07	6.12549E-08	8.86238E-08	5.04593E-08	intracellular multiplication protein IcmB
K12209	4.39202E-07	4.9526E-08	1.76782E-08	4.1138E-08	intracellular multiplication protein IcmE
K12211	6.37981E-10	2.73655E-09	4.59437E-09	3.8929E-08	intracellular multiplication protein IcmG
K12212	0	5.52437E-10	4.81619E-09	0	intracellular multiplication protein IcmJ
K12213	4.39221E-07	4.95744E-08	1.78183E-08	4.70198E-08	intracellular multiplication protein IcmK
K12214	4.39207E-07	4.95384E-08	1.7714E-08	4.6591E-08	intracellular multiplication protein IcmL
K12217	1.17888E-07	5.62215E-08	2.05756E-07	7.32003E-08	intracellular multiplication protein IcmO
K12218	8.70551E-09	4.93203E-08	1.38981E-07	5.58584E-08	intracellular multiplication protein IcmP
K12222	8.50456E-09	4.82681E-08	6.91393E-09	4.55198E-08	intracellular multiplication protein IcmT
K12226	0	0	1.05933E-08	6.91384E-07	proline/betaine transport protein TphA
K12227	5.70411E-09	3.70268E-08	5.92236E-09	3.87604E-08	TraL protein
K12228	8.67634E-09	4.86953E-08	8.15097E-09	5.41803E-08	TrbB protein
K12234	2.53064E-07	2.84156E-07	1.12493E-06	3.83544E-06	coenzyme F420-0:L-glutamate ligase / coenzyme F420-1:gamma-L-glutamate ligase [EC:6.3.2.31 6.3.2.34]
K12235	0	0	1.04657E-08	0	serine racemase [EC:5.1.1.18]
K12237	2.12366E-07	8.5601E-07	1.00426E-07	1.02497E-05	nonribosomal peptide synthetase VibF
K12238	9.8901E-08	1.67371E-07	3.41087E-07	1.03053E-05	pyochelin biosynthesis protein PchD
K12239	0.000215323	0.000214795	0.000204545	0.000157933	dihydroaeruginoic acid synthetase
K12240	0.000650329	0.000645697	0.000619307	0.000503849	pyochelin synthetase

K12241	2.31094E-09	2.9552E-09	5.32955E-08	0	pyochelin biosynthetic protein PchG
K12242	7.97456E-08	2.53236E-07	2.19728E-06	2.95403E-07	pyochelin biosynthetic protein PchC
K12243	9.85356E-07	2.45499E-06	1.11373E-05	2.77104E-05	AraC family transcriptional regulator, transcriptional activator of the genes for pyochelin and ferripyochelin receptors
K12246	2.40381E-10	5.97878E-10	1.73108E-09	7.1206E-09	poly-alpha-2,8 sialosyl sialyltransferase [EC:2.4.-.-]
K12251	2.16277E-06	3.73853E-06	6.30446E-06	6.46955E-05	N-carbamoylputrescine amidase [EC:3.5.1.53]
K12252	1.04319E-06	1.12928E-06	2.90578E-06	2.87137E-06	arginine:pyruvate transaminase [EC:2.6.1.84]
K12253	1.35274E-07	3.25518E-09	4.71678E-07	1.25163E-07	5-guanidino-2-oxopentanoate decarboxylase [EC:4.1.1.75]
K12254	4.73563E-06	5.53897E-06	1.2813E-05	8.16531E-05	4-guanidinobutyraldehyde dehydrogenase / NAD-dependent aldehyde dehydrogenase [EC:1.2.1.54 1.2.1.-]
K12255	2.17481E-06	5.79073E-06	1.24668E-05	2.8096E-05	guanidinobutyrase [EC:3.5.3.7]
K12256	0.000431517	0.00043062	0.000395132	0.000287997	putrescine---pyruvate transaminase [EC:2.6.1.113]
K12257	7.627E-06	5.53956E-06	1.31624E-05	8.06021E-05	SecD/SecF fusion protein
K12262	0.000225172	0.000221722	0.000220074	0.000296083	cytochrome b561
K12263	4.91573E-07	5.55186E-07	1.8527E-06	1.26523E-05	cytochrome c551
K12264	2.22188E-07	1.07811E-06	4.32474E-06	7.2692E-06	anaerobic nitric oxide reductase flavorubredoxin
K12265	1.12741E-06	4.71319E-08	2.58354E-06	5.66348E-06	nitric oxide reductase FlRd-NAD(+) reductase [EC:1.18.1.-]
K12266	0.001088931	0.001091969	0.00106782	0.001035085	anaerobic nitric oxide reductase transcription regulator
K12267	9.40427E-06	5.47922E-06	3.56592E-05	2.81458E-05	peptide methionine sulfoxide reductase msrA/msrB [EC:1.8.4.11 1.8.4.12]
K12268	9.96486E-07	4.33197E-08	3.00537E-06	2.84065E-06	accessory secretory protein Asp1
K12269	9.96486E-07	4.33197E-08	3.01667E-06	2.16402E-06	accessory secretory protein Asp2
K12270	9.96486E-07	4.33197E-08	2.91111E-06	1.99936E-06	accessory secretory protein Asp3
K12276	1.75681E-06	2.51568E-06	1.17061E-05	7.37014E-05	MSHA biogenesis protein MshE
K12277	0	1.35781E-08	6.41787E-08	8.40539E-06	MSHA biogenesis protein MshF
K12278	1.31878E-07	8.66275E-08	8.87368E-07	1.41783E-05	MSHA biogenesis protein MshG
K12279	8.76347E-08	5.85289E-08	5.59288E-07	1.40038E-05	MSHA biogenesis protein MshI
K12280	8.76347E-08	5.85289E-08	5.38357E-07	1.44951E-05	MSHA biogenesis protein MshJ
K12281	4.08517E-08	2.37672E-08	2.88817E-07	1.19162E-05	MSHA biogenesis protein MshK
K12282	1.56399E-06	3.05056E-06	1.43567E-05	5.09661E-05	MSHA biogenesis protein MshL
K12283	4.47742E-07	7.49519E-08	3.27364E-06	2.5742E-05	MSHA biogenesis protein MshM
K12284	8.31665E-08	3.41079E-08	5.35422E-07	1.39818E-05	MSHA biogenesis protein MshN
K12285	2.2906E-07	1.27637E-07	9.00843E-07	2.96706E-05	MSHA biogenesis protein MshO
K12286	4.6783E-08	8.19422E-08	4.75337E-07	1.39718E-05	MSHA biogenesis protein MshP
K12287	7.77874E-08	8.19422E-08	7.37032E-07	1.5367E-05	MSHA biogenesis protein MshQ
K12288	9.82442E-08	3.01083E-07	7.51553E-08	8.00724E-07	pilus assembly protein HofM
K12289	9.82118E-08	3.01003E-07	7.49218E-08	7.99159E-07	pilus assembly protein HofN
K12290	2.83409E-08	1.94894E-08	5.48719E-08	7.44952E-07	pilus assembly protein HofO
K12291	7.14627E-08	2.8868E-07	5.88123E-08	2.81867E-07	pilus assembly protein HofP
K12292	3.21011E-07	1.26241E-07	9.15676E-07	2.70174E-06	ATP-binding cassette, subfamily C, bacterial, competence factor transporting protein [EC:3.4.22.-]
K12293	6.24792E-08	7.94201E-08	3.23679E-08	2.29404E-06	competence factor transport accessory protein ComB
K12294	7.35515E-08	8.03479E-08	1.1417E-07	2.37014E-06	two-component system, LytTR family, sensor histidine kinase ComD [EC:2.7.13.3]
K12295	7.19697E-08	7.94201E-08	1.73734E-07	3.07487E-06	two-component system, LytTR family, response regulator ComE
K12296	1.48128E-07	7.94201E-08	2.98065E-07	3.65333E-06	competence protein ComX
K12297	3.15803E-06	3.74991E-06	7.91196E-06	7.68593E-05	23S rRNA (guanine2445-N2)-methyltransferase / 23S rRNA (guanine2069-N7)-methyltransferase [EC:2.1.1.173 2.1.1
K12299	1.16709E-06	6.25888E-08	1.62774E-07	4.76623E-07	MFS transporter, ACS family, probable galactarate transporter
K12308	2.53169E-06	5.40298E-06	1.22257E-05	1.21238E-05	beta-galactosidase [EC:3.2.1.23]
K12339	0.000436662	0.000438435	0.000403768	0.000293414	cysteine synthase B [EC:2.5.1.47]
K12340	0.000436022	0.000432206	0.000403645	0.000338413	outer membrane protein
K12341	1.94465E-11	4.83677E-11	2.72098E-09	1.98918E-09	adhesin YadA
K12342	0	1.14976E-09	3.61918E-08	2.00306E-08	adhesin YadB/C
K12343	1.70403E-07	7.20002E-07	6.1098E-07	1.71157E-08	3-oxo-5-alpha-steroid 4-dehydrogenase 1 [EC:1.3.1.22]
K12349	1.95307E-07	7.06504E-08	3.43981E-06	1.76832E-06	neutral ceramidase [EC:3.5.1.23]
K12368	0.000218925	0.000219467	0.000220639	0.000195573	dipeptide transport system substrate-binding protein
K12369	1.79427E-06	1.60976E-06	5.49194E-06	7.71979E-06	dipeptide transport system permease protein
K12370	0.000215272	0.000216034	0.000193698	0.00012127	dipeptide transport system permease protein
K12371	0.000221306	0.000220134	0.000203488	0.000131901	dipeptide transport system ATP-binding protein
K12372	0.000221913	0.000221032	0.000229467	0.000172458	dipeptide transport system ATP-binding protein
K12373	6.2037E-06	1.19323E-05	3.28333E-05	8.56115E-05	hexosaminidase [EC:3.2.1.52]
K12405	1.79303E-06	4.01383E-06	1.66698E-05	3.71174E-05	3-hydroxyacyl-CoA dehydrogenase / 3a,7a,12a-trihydroxy-5b-cholest-24-enoyl-CoA hydratase / enoyl-CoA hydratase
K12410	0.000225644	0.00023091	0.0002448	0.000266081	NAD-dependent deacetylase [EC:3.5.1.-]
K12415	5.93157E-08	7.94201E-08	2.39081E-08	2.10684E-07	competence-stimulating peptide
K12420	0.000217844	0.000221992	0.000219824	0.000196953	ketoreductase [EC:1.1.1.-]
K12421	8.59255E-07	3.97345E-07	2.0324E-06	2.92144E-06	fatty acid CoA ligase FadD9
K12422	0	0	9.54637E-09	0	long chain fatty acid CoA ligase FadD10 [EC:6.2.1.-]
K12423	0.000215117	0.000215061	0.000203998	0.000148138	fatty acid CoA ligase FadD21
K12424	0	0	1.801E-07	1.70245E-08	4-hydroxybenzoate adenylyltransferase [EC:6.2.1.50]
K12425	0	2.12879E-08	0	2.10716E-08	fatty acid CoA ligase FadD23
K12426	0.000214815	0.000212118	0.000188202	0.000113409	long chain fatty acid CoA FadD26
K12427	2.05517E-08	2.92734E-07	8.91647E-07	1.46204E-07	long-chain fatty acid adenylyltransferase FadD28 [EC:6.2.1.49]
K12428	0.000215135	0.000215762	0.00020642	0.000151755	fatty acid CoA ligase FadD32
K12429	2.82853E-06	3.37769E-06	6.99329E-06	6.008E-06	fatty acid CoA ligase FadD36
K12430	5.20045E-08	3.5741E-07	2.84658E-06	3.77412E-07	4-hydroxyphenylalkanoate synthase [EC:2.3.1.261]
K12431	1.71257E-09	1.61707E-07	2.1089E-07	5.73246E-08	polyketide synthase 2
K12432	2.50696E-09	4.0188E-07	7.51131E-07	1.46725E-07	mycolipanoate synthase [EC:2.3.1.252]
K12433	0.000215506	0.000213776	0.000197657	0.000128175	polyketide synthase 5
K12434	6.76152E-08	2.3893E-07	4.15374E-06	4.89642E-07	polyketide synthase 7
K12435	0	0	7.9877E-08	6.53964E-08	polyketide synthase 8
K12436	0.000218498	0.000220181	0.000232152	0.000190971	polyketide synthase 12
K12437	0.000429807	0.000427555	0.000394024	0.000260517	polyketide synthase 13
K12440	0.000214777	0.000212139	0.00018859	0.000115175	phthiocerol/phenolphthiocerol synthesis type-I polyketide synthase A
K12441	0.000428678	0.000424214	0.000376769	0.000227305	phthiocerol/phenolphthiocerol synthesis type-I polyketide synthase B
K12442	5.63695E-07	1.55352E-06	7.4087E-06	1.42335E-05	phthiocerol/phenolphthiocerol synthesis type-I polyketide synthase C
K12443	0.000428584	0.000426835	0.000388995	0.000249886	phthiocerol/phenolphthiocerol synthesis type-I polyketide synthase D
K12444	0.000215189	0.000215422	0.000205527	0.000148711	phthiocerol/phenolphthiocerol synthesis type-I polyketide synthase E
K12445	1.05176E-06	2.74051E-06	1.17868E-05	2.96373E-05	trans enoyl reductase [EC:1.3.1.-]
K12448	6.00084E-07	3.59893E-07	7.13505E-07	1.89193E-07	UDP-arabinose 4-epimerase [EC:5.1.3.5]
K12449	0	8.44232E-09	1.20444E-08	0	UDP-apiose/xylose synthase
K12452	7.72478E-07	1.88516E-06	5.53951E-06	1.80111E-05	CDP-4-dehydro-6-deoxyglucose reductase, E1 [EC:1.17.1.1]
K12454	2.19272E-07	4.18138E-08	1.40535E-06	1.33548E-07	CDP-paratose 2-epimerase [EC:5.1.3.10]

K12455	2.83595E-12	7.05362E-12	2.04229E-11	8.40071E-11	CDP-abequose synthase [EC:1.1.1.341]
K12466	1.99694E-07	4.51882E-07	5.84124E-07	1.83909E-05	(+)-trans-carveol dehydrogenase [EC:1.1.1.275]
K12472	3.73374E-07	0	1.48348E-07	2.43457E-06	epidermal growth factor receptor substrate 15
K12500	1.38712E-07	3.01011E-07	1.04853E-07	1.29523E-06	thioesterase III [EC:3.1.2.-]
K12503	1.53734E-06	4.12187E-06	9.95156E-06	4.03168E-06	short-chain Z-isoprenyl diphosphate synthase [EC:2.5.1.68]
K12506	8.14533E-06	9.68052E-06	3.79392E-05	0.000114156	2-C-methyl-D-erythritol 4-phosphate cytidylyltransferase / 2-C-methyl-D-erythritol 2,4-cyclodiphosphate synthase [EC
K12507	4.75645E-07	5.39135E-07	1.61069E-06	2.72291E-06	acyl-CoA synthetase [EC:6.2.1.-]
K12508	0.000217636	0.000218689	0.00020498	0.000166143	feruloyl-CoA synthase [EC:6.2.1.34]
K12510	0.000434083	0.000433779	0.000407244	0.000346725	tight adherence protein B
K12511	0.000433413	0.000432489	0.000396807	0.000340791	tight adherence protein C
K12512	0	1.35781E-08	1.57351E-08	6.41739E-08	tight adherence protein D
K12513	0	3.20455E-08	1.57351E-08	6.5143E-08	tight adherence protein E
K12514	0	1.35781E-08	1.57351E-08	6.56716E-08	tight adherence protein F
K12515	0	1.35781E-08	1.57351E-08	6.5143E-08	tight adherence protein G
K12516	4.86083E-08	4.51524E-08	1.43546E-07	4.05409E-06	putative surface-exposed virulence protein
K12517	1.11631E-08	4.76517E-08	1.35233E-07	6.88271E-07	major pilin subunit PapA
K12518	3.01223E-07	1.2258E-06	4.3653E-07	3.21202E-06	outer membrane usher protein PapC
K12519	2.94813E-07	1.27461E-06	4.86727E-07	2.64863E-06	chaperone protein PapD
K12520	3.53279E-10	8.36202E-09	2.5441E-09	7.51692E-08	minor pilin subunit PapE
K12521	5.26677E-10	8.7933E-09	3.79282E-09	8.03057E-08	minor pilin subunit PapF
K12522	3.2735E-10	5.02248E-09	2.35738E-09	1.29569E-08	adhesin PapG
K12523	3.53279E-10	8.36202E-09	2.5441E-09	7.51692E-08	minor pilin subunit PapK
K12524	2.72957E-06	3.85149E-06	8.25045E-06	0.000127384	bifunctional aspartokinase / homoserine dehydrogenase 1 [EC:2.7.2.4 1.1.1.3]
K12525	1.08149E-06	8.35867E-07	3.58305E-06	5.83087E-05	bifunctional aspartokinase / homoserine dehydrogenase 2 [EC:2.7.2.4 1.1.1.3]
K12526	1.06414E-06	1.2356E-06	1.80864E-06	5.25622E-05	bifunctional diaminopimelate decarboxylase / aspartate kinase [EC:4.1.1.20 2.7.2.4]
K12527	2.30069E-06	3.56502E-06	1.30378E-05	6.54423E-06	putative selenate reductase [EC:1.97.1.9]
K12528	1.3201E-06	5.31465E-07	4.29967E-07	6.15498E-06	putative selenate reductase molybdopterin-binding subunit
K12529	4.2356E-09	1.80182E-08	1.54181E-07	8.67561E-07	putative selenate reductase FAD-binding subunit
K12530	1.54164E-06	5.48928E-07	3.05946E-06	1.80906E-05	ATP-binding cassette, subfamily B, bacterial RtxB
K12531	1.44902E-06	1.46592E-06	1.31272E-05	2.89454E-05	ATP-binding cassette, subfamily B, bacterial RtxE
K12532	3.27494E-07	8.65947E-08	1.49985E-06	8.5596E-06	membrane fusion protein, RTX toxin transport system
K12533	0	8.50664E-11	2.06475E-08	1.27255E-08	ATP-binding cassette, subfamily C, bacterial RsaD
K12536	2.87621E-06	6.37867E-06	2.43755E-05	5.25818E-05	ATP-binding cassette, subfamily C, bacterial exporter for protease/lipase
K12537	3.60809E-07	8.19418E-07	1.38832E-06	2.11793E-07	membrane fusion protein, protease secretion system
K12538	1.85236E-07	4.01066E-07	1.19914E-06	1.7953E-07	outer membrane protein, protease secretion system
K12539	3.44397E-07	4.22879E-07	1.18268E-06	7.23864E-08	ATP-binding cassette, subfamily C, bacterial PrsD
K12540	1.07036E-08	2.20893E-08	5.30008E-08	0	membrane fusion protein
K12541	0.00065086	0.00064914	0.000621708	0.000554749	ATP-binding cassette, subfamily C, bacterial LapB
K12542	0.000429968	0.000427162	0.000389518	0.000299213	membrane fusion protein, adhesin transport system
K12543	0.000218315	0.000215204	0.000211434	0.000170528	outer membrane protein, adhesin transport system
K12545	6.77593E-08	2.79469E-07	2.17133E-07	7.31366E-08	heme acquisition protein HasA
K12549	8.40061E-07	1.88368E-06	8.86836E-06	2.58325E-05	surface adhesion protein
K12551	9.36655E-07	5.12659E-10	2.9173E-06	6.37555E-08	monofunctional glycosyltransferase [EC:2.4.1.129]
K12552	9.36655E-07	5.49742E-08	6.01813E-06	1.5358E-07	penicillin-binding protein 1 [EC:3.4.-.-]
K12553	1.00506E-06	3.08607E-09	2.97745E-06	5.72692E-07	penicillin-binding protein 3 [EC:3.4.-.-]
K12554	1.70131E-07	1.83961E-09	2.84946E-07	3.7925E-06	alanine adding enzyme [EC:2.3.2.-]
K12555	0.000216749	0.00021434	0.000194351	0.000124592	penicillin-binding protein 2A [EC:2.4.1.129 3.4.16.4]
K12556	9.83032E-08	1.15874E-07	3.80145E-07	3.58053E-06	penicillin-binding protein 2X
K12567	8.71626E-07	1.40647E-06	6.04755E-06	2.32289E-05	titin [EC:2.7.11.1]
K12570	0	0	5.76604E-08	0	streptomycin 6-kinase [EC:2.7.1.72]
K12573	0.000221713	0.000220034	0.000218966	0.000225457	ribonuclease R [EC:3.1.-.-]
K12574	7.9738E-06	5.99408E-06	2.90078E-05	1.27397E-05	ribonuclease J [EC:3.1.-.-]
K12582	9.82312E-08	3.01051E-07	7.50619E-08	8.0034E-07	dTDP-N-acetylfucosamine:lipid II N-acetylfucosaminyltransferase [EC:2.4.1.325]
K12583	4.56972E-06	7.97141E-06	1.09296E-05	4.63015E-06	phosphatidylinositol alpha 1,6-mannosyltransferase [EC:2.4.1.-]
K12589	3.07516E-08	2.0227E-08	4.94411E-08	1.86879E-06	exosome complex component RRP42
K12600	3.0014E-08	1.44536E-08	2.50828E-07	4.86548E-08	superkiller protein 3
K12645	0	0	1.26272E-07	9.8232E-09	epi-isozizaene 5-monooxygenase / beta-farnesene synthase [EC:1.14.13.106 4.2.3.47]
K12658	7.74525E-07	5.50738E-07	1.16491E-06	2.95961E-06	4-hydroxyproline epimerase [EC:5.1.1.8]
K12660	2.34761E-06	5.32293E-06	2.51493E-05	6.30343E-05	2-dehydro-3-deoxy-L-rhamnonate aldolase [EC:4.1.2.53]
K12661	1.72465E-06	3.00284E-06	1.40931E-05	3.49134E-05	L-rhamnonate dehydratase [EC:4.2.1.90]
K12673	0	2.05861E-07	3.23295E-08	0	N2-(2-carboxyethyl)arginine synthase [EC:2.5.1.66]
K12674	0	0	1.96563E-08	0	(carboxyethyl)arginine beta-lactam-synthase [EC:6.3.3.4]
K12675	0	0	1.96563E-08	0	clavaminate synthase [EC:1.14.11.21]
K12676	0	0	1.96563E-08	0	proclavaminate amidinohydrolase [EC:3.5.3.22]
K12677	0	1.06439E-08	1.87668E-07	9.23179E-08	clavulanate-9-aldehyde reducatase
K12678	2.32751E-06	1.62762E-06	7.47518E-06	0.000140712	autotransporter family porin
K12679	3.2292E-10	5.01146E-09	2.32548E-09	1.28257E-08	outer membrane protein IcsA
K12684	3.57641E-09	4.41246E-08	1.00801E-07	1.61205E-07	serine protease autotransporter [EC:3.4.21.-]
K12685	5.75283E-06	4.17145E-06	7.79426E-06	9.15407E-05	subtilase-type serine protease [EC:3.4.21.-]
K12686	0.000215513	0.000213548	0.00018885	0.000169951	outer membrane lipase/esterase
K12687	1.08467E-08	3.78788E-08	1.17577E-07	2.13584E-07	antigen 43
K12688	1.31562E-07	0	0	1.15974E-07	autotransporter serine protease [EC:3.4.21.-]
K12690	4.05323E-08	8.86741E-11	8.97817E-09	2.3877E-08	adhesin Hsf
K12700	1.17328E-06	5.61471E-07	5.00411E-06	5.00928E-06	non-specific ribonucleoside hydrolase [EC:3.2.-.-]
K12701	0.000216666	0.000211851	0.000186955	0.000123588	novobiocin biosynthesis protein NovH
K12702	0	0	8.31174E-08	0	novobiocin biosynthesis protein NovI
K12703	0	1.83961E-08	7.22664E-08	0	novobiocin biosynthesis protein NovJ
K12709	0	0	1.42236E-08	0	8-demethylnovobiocic acid synthase [EC:6.3.1.15]
K12710	6.2228E-08	1.55896E-07	2.27351E-07	7.19698E-08	C-methyltransferase [EC:2.1.1.-]
K12711	0	0	2.20352E-07	0	L-demethylnoviosyl transferase [EC:2.4.1.302]
K12712	0	0	1.76979E-09	0	demethyldecarbamoylnovobiocin O-methyltransferase [EC:2.1.1.285]
K12713	9.41916E-09	0	0	0	decarbamoylnovobiocin carbamoyltransferase [EC:2.1.3.12]
K12719	0.000213766	0.000211794	0.000189029	0.000113472	L-proline---[L-prolyl-carrier protein] ligase [EC:6.2.1.53]
K12720	0	0	0	8.03716E-09	peptidyl carrier protein
K12721	0	0	3.63225E-08	8.03716E-09	L-prolyl-[peptidyl-carrier protein] dehydrogenase [EC:1.3.8.14]
K12724	0	1.06439E-08	1.30075E-07	0	clorobiocin/coumermycin A biosynthesis protein CloN7/CouN7
K12733	0	4.42717E-08	1.39346E-07	2.035E-09	peptidyl-prolyl cis-trans isomerase-like 1 [EC:5.2.1.8]

K12743	5.46395E-08	2.26444E-08	2.09045E-07	2.06455E-06	N-(5-amino-5-carboxypentanoyl)-L-cysteinyl-D-valine synthase [EC:6.3.2.26]
K12784	1.0177E-09	2.53124E-09	7.32889E-09	3.01465E-08	translocated intimin receptor
K12785	1.02094E-09	2.5393E-09	7.35223E-09	3.02426E-08	LEE-encoded effector EspG
K12786	1.01122E-09	2.51512E-09	7.28221E-09	2.99545E-08	LEE-encoded effector EspF
K12787	9.98255E-10	2.48287E-09	7.18885E-09	2.95705E-08	LEE-encoded effector Map
K12788	9.98255E-10	2.48287E-09	7.18885E-09	2.95705E-08	LEE-encoded effector EspH
K12789	1.2381E-09	3.07941E-09	8.91604E-09	3.66751E-08	Tir-cytoskeleton coupling protein
K12790	1.00798E-09	2.50706E-09	7.25887E-09	3.12717E-08	intimin
K12909	2.56886E-09	0	0	0	carboxyvinyl-carboxyphosphonate phosphorylmutase [EC:2.7.8.23]
K12940	0.000221391	0.000221746	0.000237125	0.000226776	aminobenzoyl-glutamate utilization protein A
K12941	4.67103E-06	4.22345E-06	1.99559E-05	3.90012E-05	aminobenzoyl-glutamate utilization protein B
K12942	9.51325E-07	3.21807E-06	3.37919E-06	2.97403E-05	aminobenzoyl-glutamate transport protein
K12943	1.62974E-07	3.9951E-07	1.39197E-06	3.61343E-06	lipoprotein YgeR
K12944	1.69113E-07	3.44272E-07	5.16687E-07	5.37395E-06	nucleoside triphosphatase [EC:3.6.1.-]
K12945	5.15317E-07	1.52599E-06	2.87923E-06	2.16723E-06	GDP-mannose pyrophosphatase NudK [EC:3.6.1.-]
K12949	1.40052E-06	3.38968E-06	7.69444E-06	3.26477E-07	cation-transporting P-type ATPase A/B [EC:3.6.3.-]
K12950	3.4013E-07	3.92531E-07	1.87722E-06	2.88165E-07	manganese/zinc-transporting P-type ATPase C [EC:3.6.3.-]
K12951	4.92514E-09	1.24861E-07	1.39185E-07	3.98554E-07	cobalt/nickel-transporting P-type ATPase D [EC:3.6.3.-]
K12952	2.8045E-06	3.33082E-06	6.90693E-06	1.48126E-05	cation-transporting P-type ATPase E [EC:3.6.3.-]
K12953	1.10492E-06	7.93318E-07	1.87866E-06	8.23901E-06	cation-transporting P-type ATPase F [EC:3.6.3.-]
K12954	5.03179E-06	7.81127E-06	2.11037E-05	4.68299E-05	cation-transporting P-type ATPase G [EC:3.6.3.-]
K12955	2.90859E-08	2.37549E-07	6.44649E-07	1.88975E-06	cation-transporting P-type ATPase I [EC:3.6.3.-]
K12956	2.71046E-07	2.72025E-07	3.05397E-06	2.05653E-07	copper-transporting P-type ATPase V [EC:3.6.3.54]
K12957	0.000215661	0.000215008	0.000202434	0.000137706	uncharacterized zinc-type alcohol dehydrogenase-like protein [EC:1.-.-.-]
K12960	0.000437341	0.000433328	0.000398117	0.000394866	5-methylthioadenosine/S-adenosylhomocysteine deaminase [EC:3.5.4.31 3.5.4.28]
K12961	4.56577E-07	8.70977E-07	1.9942E-06	1.78971E-05	DnaA initiator-associating protein
K12962	2.68231E-07	3.69594E-07	2.04984E-06	1.47353E-06	undecaprenyl phosphate-alpha-L-ara4N flippase subunit ArnE
K12963	2.33087E-07	3.0133E-07	2.28631E-07	1.50458E-06	undecaprenyl phosphate-alpha-L-ara4N flippase subunit ArnF
K12972	0.000438862	0.000439422	0.000434448	0.000381732	glyoxylate/hydroxypyruvatereductase [EC:1.1.1.79 1.1.1.81]
K12973	1.06186E-07	3.39065E-07	3.90865E-07	8.96302E-07	lipid IVA palmitoyltransferase [EC:2.3.1.251]
K12974	3.72721E-07	7.34884E-07	5.81845E-07	2.56722E-06	KDO2-lipid IV(A) palmitoleoyltransferase [EC:2.3.1.242]
K12975	1.18483E-07	3.01456E-07	1.00381E-06	8.5125E-07	KDO II ethanolaminephosphotransferase [EC:2.7.8.42]
K12976	0.000215218	0.000215623	0.00020134	0.000153299	lipid A 3-O-deacylase [EC:3.1.-.-]
K12978	3.70658E-07	1.64915E-08	1.37968E-07	4.85503E-08	lipid A 4-phosphatase [EC:3.1.3.-]
K12979	0.000215194	0.000213077	0.000188365	0.000167092	beta-hydroxylase [EC:1.14.11.-]
K12980	0	0	1.52483E-08	1.43658E-06	lipid A oxidase
K12981	1.09151E-06	1.75564E-07	8.63521E-07	2.69428E-07	KDO transferase III [EC:2.4.99.-]
K12982	1.06561E-06	1.28249E-06	2.50011E-06	6.81595E-05	heptosyltransferase I [EC:2.4.-.-]
K12983	7.24788E-08	1.11706E-07	9.5389E-08	5.14007E-08	UDP-glucose:(glucosyl)LPS beta-1,3-glucosyltransferase [EC:2.4.1.-]
K12984	0.000215289	0.000213417	0.000189283	0.00018023	(heptosyl)LPS beta-1,4-glucosyltransferase [EC:2.4.1.-]
K12985	1.56392E-09	8.09808E-09	1.12624E-08	5.01924E-08	(galactosyl)LPS 1,2-glucosyltransferase [EC:2.4.1.-]
K12986	1.17435E-09	2.92087E-09	8.45701E-09	3.47869E-08	1,5-rhamnosyltransferase [EC:2.4.1.-]
K12987	3.14299E-07	2.91016E-07	1.07542E-07	4.69534E-08	alpha-1,6-rhamnosyltransferase [EC:2.4.1.-]
K12988	9.26475E-07	1.39669E-06	1.21171E-06	1.95372E-06	alpha-1,3-rhamnosyltransferase [EC:2.4.1.-]
K12989	3.69189E-07	8.05982E-07	9.81802E-07	1.33507E-06	mannosyltransferase [EC:2.4.1.-]
K12990	0.000214956	0.000212321	0.000187139	0.000132149	rhamnosyltransferase [EC:2.4.1.-]
K12991	1.34966E-07	5.95173E-10	3.54793E-08	1.47162E-06	rhamnosyltransferase [EC:2.4.1.-]
K12992	1.16384E-06	3.92837E-07	3.54272E-06	1.59642E-06	rhamnosyltransferase [EC:2.4.1.-]
K12993	1.21164E-06	1.70304E-07	5.58968E-07	1.12441E-06	alpha-1,2-rhamnosyltransferase [EC:2.4.1.-]
K12994	4.18724E-06	3.88489E-06	1.7081E-05	3.50872E-05	mannosyl-N-acetyl-alpha-D-glucosaminyl-diphospho-ditrans,octacis-undecaprenol 3-alpha-mannosyltransferase / alpha
K12995	1.10866E-06	3.18058E-06	1.54223E-05	2.89029E-05	N-acetyl-alpha-D-glucosaminyl-diphospho-ditrans,octacis-undecaprenol 3-alpha-mannosyltransferase / rhamnosyltrans
K12996	1.47198E-06	1.3661E-06	1.67851E-06	7.91839E-06	rhamnosyltransferase [EC:2.4.1.-]
K12997	7.79918E-07	8.00695E-07	8.69345E-06	4.08718E-05	rhamnosyltransferase [EC:2.4.1.-]
K12998	4.48656E-07	9.56985E-07	4.1434E-07	2.55106E-06	glucosyltransferase [EC:2.4.1.-]
K12999	4.38282E-07	3.8469E-07	3.59684E-06	3.90355E-06	glucosyltransferase [EC:2.4.1.-]
K13001	2.84863E-07	5.78042E-07	2.08573E-06	3.07055E-08	mannosyltransferase [EC:2.4.1.-]
K13002	1.07662E-06	3.47917E-06	4.46969E-06	1.72017E-07	glycosyltransferase [EC:2.4.1.-]
K13004	1.05019E-06	1.70034E-07	7.15135E-07	1.55258E-06	galacturonosyltransferase [EC:2.4.1.-]
K13005	2.83595E-12	4.48425E-08	4.83509E-08	2.59623E-09	abequosyltransferase [EC:2.4.1.60]
K13006	2.74729E-07	6.0419E-07	2.76027E-06	6.92577E-06	UDP-perosamine 4-acetyltransferase [EC:2.3.1.-]
K13007	2.66207E-06	2.1473E-06	3.25455E-06	5.28697E-05	Fuc2NAc and GlcNAc transferase [EC:2.4.1.-]
K13009	1.03911E-07	4.32255E-07	2.811E-07	1.19389E-05	O-antigen polymerase [EC:2.4.1.-]
K13010	0.00043817	0.00043651	0.000405152	0.000287217	perosamine synthetase [EC:2.6.1.102]
K13012	0.000221179	0.000221377	0.000202363	0.000129812	O-antigen biosynthesis protein WbqP
K13013	0.000218744	0.000216751	0.000194655	0.000176839	O-antigen biosynthesis protein WbqV
K13014	0.000214031	0.000212167	0.000186509	0.000114663	undecaprenyl phosphate-alpha-L-ara4FN deformylase [EC:3.5.1.-]
K13015	3.01324E-06	7.10946E-06	7.01016E-06	2.35954E-05	UDP-N-acetyl-D-glucosamine dehydrogenase [EC:1.1.1.136]
K13016	2.69639E-07	9.20045E-08	1.93437E-06	8.15495E-07	UDP-N-acetyl-2-amino-2-deoxyglucuronate dehydrogenase [EC:1.1.1.335]
K13017	0.000433398	0.000429744	0.000388718	0.000242117	UDP-2-acetamido-2-deoxy-ribo-hexuluronate aminotransferase [EC:2.6.1.98]
K13018	3.9333E-06	4.19552E-06	1.61599E-05	2.03113E-05	UDP-2-acetamido-3-amino-2,3-dideoxy-glucuronate N-acetyltransferase [EC:2.3.1.201]
K13019	1.06742E-06	3.02896E-07	7.28036E-06	1.35005E-06	UDP-GlcNAc3NAcA epimerase [EC:5.1.3.23]
K13020	2.87667E-07	1.33479E-07	4.95134E-07	2.73815E-06	UDP-N-acetyl-2-amino-2-deoxyglucuronate dehydrogenase [EC:1.1.1.335]
K13021	0.000444368	0.000442978	0.000453721	0.000424979	MFS transporter, ACS family, tartrate transporter
K13028	2.64576E-08	5.02282E-08	1.20474E-06	1.76541E-06	aldoxime dehydratase [EC:4.99.1.5]
K13038	0.000223721	0.000224658	0.000228797	0.000226973	phosphopantothenoylcysteine decarboxylase / phosphopantothenate---cysteine ligase [EC:4.1.1.36 6.3.2.5]
K13039	1.11027E-07	1.64379E-07	7.0735E-08	0	sulfopyruvate decarboxylase subunit beta [EC:4.1.1.79]
K13040	0.000216911	0.000216765	0.000210029	0.000179066	two-component system, LuxR family, sensor histidine kinase TtrS [EC:2.7.13.3]
K13041	0.00021683	0.000216341	0.00020925	0.000166373	two-component system, LuxR family, response regulator TtrR
K13043	9.04537E-07	1.86108E-06	3.98226E-06	5.14298E-05	N-succinyl-L-ornithine transcarbamylase [EC:2.1.3.11]
K13048	1.8688E-06	3.55477E-06	1.44284E-05	3.5018E-05	carboxypeptidase Ss1 [EC:3.4.17.-]
K13049	0.000214304	0.000212466	0.000193074	0.000118284	carboxypeptidase PM20D1 [EC:3.4.17.-]
K13051	0.000219696	0.000218653	0.000216849	0.000317301	beta-aspartyl-peptidase (threonine type) [EC:3.4.19.5]
K13052	1.39477E-06	4.03957E-07	6.79976E-06	3.64195E-06	cell division protein DivIC
K13053	1.53378E-06	1.95279E-06	3.27196E-06	6.05421E-05	cell division inhibitor SulA
K13057	9.33908E-09	7.60125E-08	2.15261E-07	1.73552E-08	trehalose synthase [EC:2.4.1.245]
K13058	5.17567E-07	3.83253E-09	3.29735E-07	6.15173E-09	mannosylfructose-phosphate synthase [EC:2.4.1.246]
K13059	4.30889E-07	7.80851E-07	2.71548E-06	3.69824E-07	N-acetylhexosamine 1-kinase [EC:2.7.1.162]

K13060	4.71081E-07	3.29661E-07	6.25113E-07	1.04077E-05	acyl homoserine lactone synthase [EC:2.3.1.184]
K13061	0.000428061	0.000424219	0.000375778	0.000233422	acyl homoserine lactone synthase [EC:2.3.1.184]
K13062	0	1.35781E-08	5.76224E-08	5.18537E-06	acyl homoserine lactone synthase [EC:2.3.1.184]
K13063	3.2857E-09	3.21513E-07	2.75183E-07	1.9747E-06	2-amino-4-deoxychorismate synthase [EC:2.6.1.86]
K13069	0.001324056	0.001325143	0.001369506	0.00170506	diguanylate cyclase [EC:2.7.7.65]
K13074	2.88181E-08	1.66281E-07	2.01082E-06	4.2979E-07	biflaviolin synthase [EC:1.14.21.7]
K13075	0.000216428	0.000213379	0.000190417	0.000175452	N-acyl homoserine lactone hydrolase [EC:3.1.1.81]
K13085	2.55236E-11	6.34826E-11	1.83806E-10	2.71497E-06	phosphatidylinositol-4,5-bisphosphate 4-phosphatase [EC:3.1.3.78]
K13086	0	0	3.21097E-08	0	mannosylfructose-6-phosphate phosphatase [EC:3.1.3.79]
K13237	0	0	1.04657E-08	0	peroxisomal 2,4-dienoyl-CoA reductase [EC:1.3.1.34]
K13243	4.94804E-06	6.1209E-06	2.88736E-05	0.000126601	c-di-GMP-specific phosphodiesterase [EC:3.1.4.52]
K13244	1.37878E-07	7.74166E-08	1.61148E-06	1.84175E-06	c-di-GMP-specific phosphodiesterase [EC:3.1.4.52]
K13245	3.89194E-07	7.57351E-08	3.91993E-07	2.02277E-07	c-di-GMP-specific phosphodiesterase [EC:3.1.4.52]
K13246	4.76436E-06	6.41978E-06	3.26731E-05	8.15886E-05	c-di-GMP phosphodiesterase [EC:3.1.4.52]
K13252	9.34164E-09	0	6.33822E-08	1.98419E-06	putrescine carbamoyltransferase [EC:2.1.3.6]
K13255	1.23362E-06	1.24985E-07	3.67207E-06	1.19221E-06	ferric iron reductase protein FhuF
K13256	3.05428E-07	3.972E-07	6.43543E-07	3.13246E-06	protein PsiE
K13274	4.92328E-07	4.44539E-07	3.03081E-06	1.68805E-05	cell wall-associated protease [EC:3.4.21.-]
K13275	1.79246E-06	1.36214E-06	5.6382E-06	2.84863E-05	major intracellular serine protease [EC:3.4.21.-]
K13276	0.00021403	0.000211775	0.000186112	0.000113241	bacillopeptidase F [EC:3.4.21.-]
K13277	1.19873E-07	8.22348E-08	4.73531E-07	6.78614E-07	minor extracellular protease Epr [EC:3.4.21.-]
K13280	6.83057E-08	1.09157E-07	1.3485E-06	8.78271E-06	signal peptidase I [EC:3.4.21.89]
K13281	3.70634E-07	1.00533E-07	3.89703E-07	5.00685E-08	UV DNA damage endonuclease [EC:3.-.-.-]
K13282	5.60984E-07	5.04684E-07	1.88651E-06	4.98542E-06	cyanophycinase [EC:3.4.15.6]
K13283	1.63137E-06	1.53843E-06	3.08721E-06	1.77532E-05	ferrous-iron efflux pump FieF
K13284	3.84879E-11	9.57277E-11	2.77167E-10	1.1401E-09	invasin A
K13285	7.02756E-10	9.48731E-09	7.04429E-08	1.1962E-08	invasin B
K13286	2.55236E-11	7.56112E-09	6.0247E-08	7.56064E-10	invasin C
K13287	3.36668E-10	8.93647E-09	6.97868E-08	1.1386E-08	invasin D
K13288	0.000219202	0.000221031	0.00021645	0.000222103	oligoribonuclease [EC:3.1.-.-]
K13292	0.000224851	0.000223662	0.00022919	0.000226849	phosphatidylglycerol:prolipoprotein diacylglycerol transferase [EC:2.-.-.-]
K13300	2.01757E-07	4.12674E-07	7.33614E-07	1.90174E-09	cytochrome c550
K13301	9.82085E-08	3.00995E-07	7.48985E-08	7.99668E-07	secretion monitor
K13307	0	0	1.9931E-08	0	dTDP-3-amino-3,6-dideoxy-alpha-D-glucopyranose N,N-dimethyltransferase [EC:2.1.1.235]
K13308	1.07078E-06	5.70525E-08	1.65332E-07	8.73206E-09	dTDP-4-amino-4,6-dideoxy-D-glucose transaminase [EC:2.6.1.33]
K13310	9.00074E-09	3.7973E-09	1.84298E-07	4.75405E-06	dTDP-3-amino-3,4,6-trideoxy-alpha-D-glucose transaminase [EC:2.6.1.106]
K13311	0	0	3.80922E-08	0	dTDP-3-amino-3,4,6-trideoxy-alpha-D-glucopyranose N,N-dimethyltransferase [EC:2.1.1.234]
K13313	0	0	0	1.78604E-09	dTDP-4-dehydro-6-deoxy-alpha-D-gulose 4-ketoreductase [EC:1.1.1.364]
K13315	4.50345E-06	7.15875E-06	2.28762E-05	7.93286E-05	NDP-hexose C3-ketoreductase / dTDP-4-oxo-2-deoxy-alpha-D-pentos-2-ene 2,3-reductase [EC:1.1.1.-]
K13316	0	0	5.62647E-08	2.28209E-08	NDP-hexose 5-epimerase [EC:5.1.3.-]
K13317	0	0	2.84057E-08	1.07162E-08	NDP-4-keto-2,6-dideoxyhexose 3-C-methyltransferase [EC:2.1.1.-]
K13318	0	0	2.60478E-08	0	dTDP-4-keto-6-deoxy-L-hexose 4-reductase [EC:1.1.1.-]
K13322	0	0	1.05031E-07	0	dTDP-4-keto-6-deoxyhexose 4-ketoreductase
K13326	0	0	1.76979E-09	0	dTDP-3-amino-2,3,6-trideoxy-4-keto-D-glucose/dTDP-3-amino-3,4,6-trideoxy-alpha-D-glucopyranose N,N-dimethylt
K13327	4.1639E-07	1.3264E-06	1.8495E-06	4.46875E-06	dTDP-3,4-didehydro-2,6-dideoxy-alpha-D-glucose 3-reductase [EC:1.1.1.384]
K13328	5.3218E-08	7.30716E-09	6.37263E-07	3.60442E-06	dTDP-4-dehydro-2,6-dideoxy-D-glucose 3-dehydratase [EC:4.2.1.164]
K13329	0	0	2.06475E-09	0	dTDP-4-dehydro-2,3,6-trideoxy-D-glucose 4-aminotransferase [EC:2.6.1.110]
K13330	0	0	3.63225E-08	0	dTDP-4-amino-2,3,4,6-tetradeoxy-D-glucose N,N-dimethyltransferase [EC:2.1.1.324]
K13356	0	3.74292E-09	1.3749E-07	6.22457E-08	alcohol-forming fatty acyl-CoA reductase [EC:1.2.1.84]
K13372	5.39461E-08	2.83838E-08	4.09739E-08	5.65278E-09	aralkylamine dehydrogenase heavy chain [EC:1.4.9.2]
K13378	2.26984E-06	3.32451E-06	5.81124E-06	5.42227E-06	NADH-quinone oxidoreductase subunit C/D [EC:1.6.5.3]
K13380	1.31376E-06	3.75773E-07	1.19572E-06	6.91352E-07	NADH-quinone oxidoreductase subunit B/C/D [EC:1.6.5.3]
K13381	2.57583E-09	1.389E-08	2.40151E-08	1.50981E-07	bifunctional chitinase/lysozyme [EC:3.2.1.14 3.2.1.17]
K13408	3.10726E-06	1.70195E-06	4.0106E-06	2.98857E-05	membrane fusion protein
K13409	8.57567E-06	1.1154E-05	2.34001E-05	8.57174E-05	ATP-binding cassette, subfamily B, bacterial RaxB
K13419	3.04112E-07	5.34113E-07	1.08227E-06	9.36215E-07	serine/threonine-protein kinase PknK [EC:2.7.11.1]
K13421	0	4.42717E-08	1.32186E-07	2.035E-09	uridine monophosphate synthetase [EC:2.4.2.10 4.1.1.23]
K13439	0.000213766	0.000211774	0.000186978	0.000113255	cysteine protease avirulence protein AvrRpt2 [EC:3.4.22.-]
K13444	1.87289E-08	2.08486E-08	2.48041E-07	1.79912E-08	formylglycine-generating enzyme [EC:1.8.3.7]
K13450	9.72326E-12	7.52182E-09	7.00212E-11	2.88024E-10	phosphothreonine lyase [EC:4.2.3.-]
K13461	0	0	1.18611E-08	6.27574E-09	effector protein HopM1
K13472	0.000213785	0.000211777	0.000186144	0.000113264	sulfotransferase
K13479	3.41492E-07	1.50511E-07	5.57205E-07	1.22047E-06	xanthine dehydrogenase FAD-binding subunit [EC:1.17.1.4]
K13480	9.93607E-09	2.78837E-08	2.13455E-07	9.03955E-07	xanthine dehydrogenase iron-sulfur-binding subunit
K13481	0.00021867	0.000215875	0.000205412	0.000160254	xanthine dehydrogenase small subunit [EC:1.17.1.4]
K13482	0.000216822	0.000215689	0.000206216	0.000152012	xanthine dehydrogenase large subunit [EC:1.17.1.4]
K13483	0.000226122	0.000221807	0.000231317	0.000245255	xanthine dehydrogenase YagT iron-sulfur-binding subunit
K13485	0.000215178	0.000212527	0.000189225	0.000113902	2-oxo-4-hydroxy-4-carboxy-5-ureidoimidazoline decarboxylase [EC:4.1.1.97]
K13486	0.000214411	0.000211911	0.000187003	0.000113307	chemotaxis protein methyltransferase WspC
K13487	0.000214143	0.000211969	0.000187259	0.000115911	methyl-accepting chemotaxis protein WspA
K13488	1.07082E-06	1.17222E-07	7.91576E-07	1.0474E-07	chemotaxis-related protein WspB
K13489	2.09448E-07	1.15859E-07	7.9077E-07	1.03945E-07	chemotaxis-related protein WspD
K13490	5.50197E-07	2.62582E-07	4.52503E-06	4.24356E-06	two-component system, chemotaxis family, sensor histidine kinase and response regulator WspE
K13491	6.49393E-06	4.23453E-06	2.40711E-05	5.80412E-05	two-component system, chemotaxis family, response regulator WspF [EC:3.1.1.61]
K13497	7.84918E-07	1.62356E-06	4.06551E-06	1.73492E-05	anthranilate synthase/phosphoribosyltransferase [EC:4.1.3.27 2.4.2.18]
K13498	0.000215573	0.000213185	0.000188946	0.000137223	indole-3-glycerol phosphate synthase / phosphoribosylanthranilate isomerase [EC:4.1.1.48 5.3.1.24]
K13500	0	0	2.63012E-09	3.23077E-09	chondroitin synthase [EC:2.4.1.175 2.4.1.226]
K13503	2.24496E-06	2.9013E-06	7.45532E-07	7.931E-09	anthranilate synthase [EC:4.1.3.27]
K13520	2.26876E-11	5.6429E-11	1.63383E-10	6.72057E-10	outer membrane protease [EC:3.4.23.-]
K13522	0.000216714	0.000215074	0.000204361	0.000176163	bifunctional NMN adenylyltransferase/nudix hydrolase [EC:2.7.7.1 3.6.1.-]
K13525	0.000436705	0.000437555	0.000412251	0.00027353	transitional endoplasmic reticulum ATPase
K13527	1.02444E-06	4.23706E-06	3.53981E-06	2.06677E-06	proteasome-associated ATPase
K13529	0.000218704	0.000218664	0.000217031	0.000251482	AraC family transcriptional regulator, regulatory protein of adaptative response / DNA-3-methyladenine glycosylase II
K13530	5.26063E-07	2.27773E-07	5.37449E-07	1.03793E-06	AraC family transcriptional regulator, regulatory protein of adaptative response / methylphosphotriester-DNA alkyltrar
K13531	3.248E-07	9.79437E-08	2.28129E-06	2.62694E-06	methylated-DNA-[protein]-cysteine S-methyltransferase [EC:2.1.1.63]
K13532	8.18431E-07	6.99453E-07	1.99348E-06	5.25488E-05	two-component system, sporulation sensor kinase D [EC:2.7.13.3]
K13533	1.22599E-06	7.78718E-07	1.6475E-06	2.28061E-07	two-component system, sporulation sensor kinase E [EC:2.7.13.3]

K13540	2.99765E-06	3.74096E-06	1.95042E-05	3.68133E-05	precorrin-2 C20-methyltransferase / precorrin-3B C17-methyltransferase [EC:2.1.1.130 2.1.1.131]
K13541	0.000217539	0.000216464	0.000213307	0.000153355	cobalt-precorrin 5A hydrolase / precorrin-3B C17-methyltransferase [EC:3.7.1.12 2.1.1.131]
K13542	7.79896E-06	6.71024E-06	2.27387E-05	2.18304E-05	uroporphyrinogen III methyltransferase / synthase [EC:2.1.1.107 4.2.1.75]
K13543	0.000215158	0.000214943	0.000202666	0.0001515	uroporphyrinogen III methyltransferase / synthase [EC:2.1.1.107 4.2.1.75]
K13547	0	3.74882E-09	9.30556E-09	2.99939E-08	L-glutamine:2-deoxy-scyllo-inosose/3-amino-2,3-dideoxy-scyllo-inosose aminotransferase [EC:2.6.1.100 2.6.1.101]
K13566	3.84687E-06	4.60734E-06	1.31631E-05	5.73861E-05	omega-amidase [EC:3.5.1.3]
K13570	9.91973E-07	3.50199E-06	3.43315E-06	2.06561E-06	prokaryotic ubiquitin-like protein Pup
K13571	9.91973E-07	3.89345E-06	4.00172E-06	2.24544E-06	proteasome accessory factor A [EC:6.3.1.19]
K13572	1.50811E-06	4.04772E-06	9.2704E-06	3.96073E-06	proteasome accessory factor B
K13573	2.6054E-06	5.37239E-06	1.59727E-05	4.96557E-05	proteasome accessory factor C
K13574	1.66494E-06	5.01547E-07	1.08523E-06	9.03369E-07	uncharacterized oxidoreductase [EC:1.1.1.-]
K13580	0.000215144	0.000214763	0.000200873	0.000147932	magnesium chelatase subunit ChlD-like protein
K13581	0.000430543	0.000424635	0.00037785	0.000237548	modification methylase [EC:2.1.1.72]
K13582	1.68944E-06	2.36527E-07	6.11035E-07	1.61651E-07	localization factor PodJL
K13583	2.12679E-06	2.59616E-07	9.84912E-07	3.43308E-07	GcrA cell cycle regulator
K13584	8.98148E-06	5.36635E-06	2.27568E-05	9.5724E-05	two-component system, cell cycle response regulator CtrA
K13585	7.29798E-08	1.64915E-08	7.60122E-08	2.09243E-08	holdfast attachment protein HfaA
K13586	4.45718E-07	6.59659E-08	3.04049E-07	1.21375E-07	holdfast attachment protein HfaB
K13587	3.51189E-05	1.75102E-05	7.39622E-05	0.000385464	two-component system, cell cycle sensor histidine kinase and response regulator CckA [EC:2.7.13.3]
K13588	1.70111E-06	1.99396E-07	6.21153E-07	1.76865E-07	histidine phosphotransferase ChpT
K13589	1.73846E-06	2.44041E-07	1.13777E-06	1.84472E-07	two-component system, cell cycle response regulator CpdR
K13590	0.002424875	0.002425321	0.002478767	0.003171517	diguanylate cyclase [EC:2.7.7.65]
K13591	3.84726E-07	4.94744E-08	2.47129E-07	1.10626E-07	two-component system, cell cycle response regulator PopA
K13592	1.70111E-06	1.99396E-07	6.03711E-07	1.61651E-07	regulator of CtrA degradation
K13593	1.69314E-06	8.26942E-08	3.44791E-07	1.51476E-07	cyclic-di-GMP phosphodiesterase, flagellum assembly factor TipF
K13598	0.000219578	0.000216382	0.000205813	0.0001496	two-component system, NtrC family, nitrogen regulation sensor histidine kinase NtrY [EC:2.7.13.3]
K13599	0.000873913	0.000873554	0.000855382	0.000752311	two-component system, NtrC family, nitrogen regulation response regulator NtrX
K13601	0	0	0	5.28572E-10	bacteriochlorophyllide d C-8(2)-methyltransferase [EC:2.1.1.332]
K13602	3.97169E-09	0	0	0	bacteriochlorophyllide d C-12(1)-methyltransferase [EC:2.1.1.331]
K13607	0	0	1.22398E-07	0	cinnamoyl-CoA:phenyllactate CoA-transferase [EC:2.8.3.17]
K13609	1.00749E-06	1.69742E-06	6.44801E-06	1.4894E-05	delta1-piperideine-2-carboxylate reductase [EC:1.5.1.21]
K13611	0.000864215	0.000862938	0.00083397	0.000641774	polyketide synthase PksJ
K13612	0.000430085	0.000427854	0.000396529	0.000266448	polyketide synthase PksL
K13613	0.0006419	0.000636422	0.000565644	0.000348009	polyketide synthase PksM
K13614	0.001076084	0.001070228	0.000987694	0.00068679	polyketide synthase PksN
K13615	6.87506E-08	2.77373E-08	1.17845E-06	1.81015E-07	polyketide synthase PksR
K13620	3.84751E-09	1.49276E-08	4.53393E-08	2.09877E-07	putative colanic acid polymerase
K13622	1.60071E-08	1.26454E-07	1.52106E-07	3.52055E-07	S-adenosylmethionine-diacylglycerol 3-amino-3-carboxypropyl transferase
K13623	1.98585E-08	1.263E-07	1.83313E-07	3.86246E-08	S-adenosylmethionine-diacylgycerolhomoserine-N-methlytransferase
K13626	5.92256E-07	4.57085E-07	1.38289E-06	1.99487E-07	flagellar assembly factor FliW
K13628	0.000223417	0.000222452	0.000221653	0.00022244	iron-sulfur cluster assembly protein
K13629	2.85177E-08	3.43588E-08	2.42402E-07	1.86966E-06	D-serine transporter
K13630	2.83182E-08	1.9433E-08	5.47085E-08	7.34584E-07	multiple antibiotic resistance protein MarB
K13631	1.22207E-07	3.02201E-07	9.4055E-08	1.22058E-06	AraC family transcriptional regulator, mar-sox-rob regulon activator
K13632	8.4873E-08	2.47278E-07	9.65201E-08	7.67616E-07	AraC family transcriptional regulator, mar-sox-rob regulon activator
K13633	0.000220936	0.000222076	0.000230106	0.000286441	AraC family transcriptional regulator, transcriptional activator FtrA
K13634	0.000435273	0.000433092	0.000416892	0.000382061	LysR family transcriptional regulator, cys regulon transcriptional activator
K13635	0.000432293	0.000427999	0.000395363	0.00027781	LysR family transcriptional regulator, cys regulon transcriptional activator
K13636	0.000431946	0.000428692	0.000400051	0.000291642	LysR family transcriptional regulator, D-serine deaminase activator
K13637	3.07961E-06	6.82287E-06	3.59897E-05	7.76007E-05	GntR family transcriptional regulator, uxu operon transcriptional repressor
K13638	1.98811E-06	2.20631E-06	1.25336E-05	4.55043E-05	MerR family transcriptional regulator, Zn(II)-responsive regulator of zntA
K13639	0.0002194	0.000222001	0.000226082	0.000300269	MerR family transcriptional regulator, redox-sensitive transcriptional activator SoxR
K13640	1.61681E-06	4.22091E-06	9.85569E-06	7.16294E-06	MerR family transcriptional regulator, heat shock protein HspR
K13641	1.36743E-06	1.876E-06	7.68848E-06	8.81321E-06	IclR family transcriptional regulator, acetate operon repressor
K13642	1.60453E-06	7.2056E-08	3.46322E-08	2.77276E-08	CRP/FNR family transcriptional regulator, transcriptional activator FtrB
K13643	0.000217914	0.000217629	0.000210501	0.000166458	Rrf2 family transcriptional regulator, iron-sulfur cluster assembly transcription factor
K13650	8.85374E-08	2.61975E-07	7.23677E-08	6.98636E-07	MqsR-controlled colanic acid and biofilm protein A
K13651	4.88523E-07	6.46114E-08	1.49234E-07	1.41012E-07	motility quorum-sensing regulator / GCU-specific mRNA interferase toxin
K13652	0.000433568	0.000434835	0.000422615	0.000364442	AraC family transcriptional regulator
K13653	3.17524E-06	2.08904E-06	6.84662E-06	8.69941E-05	AraC family transcriptional regulator
K13654	6.23099E-08	1.59357E-07	5.24237E-07	4.61376E-07	GntR family transcriptional regulator, colanic acid and biofilm gene transcriptional regulator
K13655	5.09195E-07	9.51619E-08	2.29239E-07	1.94807E-07	HTH-type transcriptional regulator / antitoxin MqsA
K13656	5.13066E-07	9.22981E-07	1.10853E-06	1.83328E-05	undecaprenyl-phosphate glucose phosphotransferase [EC:2.7.8.31]
K13657	3.54646E-08	1.17078E-08	2.1872E-07	1.77913E-07	alpha-1,3-mannosyltransferase [EC:2.4.1.252]
K13658	5.6332E-08	4.43712E-08	2.53003E-07	3.76005E-06	beta-1,4-mannosyltransferase [EC:2.4.1.251]
K13659	2.997E-09	0	1.61098E-07	0	2-beta-glucuronyltransferase [EC:2.4.1.264]
K13660	2.997E-09	8.142E-08	3.49021E-07	8.65957E-10	beta-1,4-glucosyltransferase [EC:2.4.1.-]
K13661	2.997E-09	0	1.36678E-07	0	GumC protein
K13662	2.997E-09	7.46852E-09	1.61098E-07	0	putative polymerase
K13663	2.997E-09	5.11004E-09	1.36678E-07	1.11362E-09	acyltransferase [EC:2.3.1.-]
K13664	2.997E-09	0	1.36678E-07	0	acyltransferase [EC:2.3.1.-]
K13665	6.11553E-09	5.63486E-09	2.28723E-07	3.98457E-07	pyruvyltransferase
K13668	6.72577E-07	2.32254E-06	4.35259E-06	1.12352E-05	phosphatidyl-myo-inositol dimannoside synthase [EC:2.4.1.346]
K13669	0	3.61181E-07	4.17082E-07	9.0757E-08	alpha-1,2-mannosyltransferase [EC:2.4.1.-]
K13670	1.04139E-07	2.57231E-07	3.02343E-08	3.50908E-08	putative glycosyltransferase [EC:2.4.-.-]
K13671	6.95591E-07	1.82558E-06	1.01965E-05	5.68933E-06	alpha-1,2-mannosyltransferase [EC:2.4.1.-]
K13677	1.33139E-06	1.57806E-07	7.46174E-07	3.31441E-06	1,2-diacylglycerol-3-alpha-glucose alpha-1,2-glucosyltransferase [EC:2.4.1.208]
K13678	1.9725E-07	1.25256E-07	3.12688E-06	4.0591E-07	1,2-diacylglycerol-3-alpha-glucose alpha-1,2-galactosyltransferase [EC:2.4.1.-]
K13683	7.7433E-07	2.64458E-06	6.28684E-06	4.81105E-07	putative colanic acid biosynthesis glycosyltransferase [EC:2.4.-.-]
K13684	1.2939E-08	1.51775E-08	4.60628E-08	2.18212E-07	putative colanic acid biosynthesis glycosyltransferase [EC:2.4.-.-]
K13686	0	5.96007E-07	2.43706E-06	1.76234E-06	galactan 5-O-arabinofuranosyltransferase [EC:2.4.2.46]
K13687	4.81352E-07	1.74252E-06	6.73755E-06	1.56028E-05	arabinofuranosyltransferase [EC:2.4.2.-]
K13688	1.3945E-06	2.83974E-06	1.30639E-05	3.19598E-05	cyclic beta-1,2-glucan synthetase [EC:2.4.1.-]
K13693	0.000213779	0.000212562	0.000189538	0.000118331	glucosyl-3-phosphoglycerate synthase [EC:2.4.1.266]
K13694	5.97707E-07	1.88637E-06	3.64261E-06	1.33807E-05	murein DD-endopeptidase / murein LD-carboxypeptidase [EC:3.4.-.- 3.4.17.13]
K13695	2.32658E-07	5.68792E-07	1.45321E-06	1.63242E-05	probable lipoprotein NlpC
K13714	3.17077E-06	1.83275E-08	9.01492E-06	2.22646E-06	bifunctional autolysin [EC:3.5.1.28 3.2.1.96]

K13715	1.24386E-07	0	1.6748E-08	0	staphopain B [EC:3.4.22.-]
K13722	5.567E-08	2.49253E-08	6.82544E-08	3.70294E-06	tricorn protease interacting factor F2/3 [EC:3.4.11.-]
K13727	1.26852E-07	5.14513E-08	2.60868E-07	1.49431E-06	phenolic acid decarboxylase [EC:4.1.1.-]
K13730	1.16952E-06	2.00512E-07	1.25522E-06	8.41441E-07	internalin A
K13731	1.55884E-07	1.83275E-08	3.26318E-08	4.96043E-08	internalin B
K13732	4.58579E-08	0	6.31488E-09	3.38314E-07	fibronectin-binding protein A
K13733	4.61227E-08	3.38617E-10	5.02441E-09	0	fibronectin-binding protein B
K13734	1.58175E-09	0	5.74377E-08	3.34946E-07	fibronectin-binding protein 1
K13735	6.67081E-07	1.61177E-06	5.83439E-06	1.79968E-05	adhesin/invasin
K13739	2.83595E-12	7.05362E-12	2.04229E-11	8.40071E-11	secreted effector protein SopD
K13740	5.6719E-12	1.41072E-11	4.08457E-11	1.68014E-10	secreted effector protein SptP
K13741	5.6719E-12	7.51174E-09	5.67202E-08	1.68014E-10	guanine nucleotide exchange factor SopE
K13742	2.26876E-11	5.6429E-11	1.63383E-10	6.72057E-10	protein IpgB1
K13743	1.8928E-09	4.70779E-09	1.36308E-08	5.60687E-08	protein IpgB2
K13745	1.36268E-06	7.13214E-07	4.15562E-06	2.46121E-05	L-2,4-diaminobutyrate decarboxylase [EC:4.1.1.86]
K13746	2.97877E-09	4.50344E-07	1.16794E-06	8.87552E-06	carboxynorspermidine synthase [EC:1.5.1.43]
K13747	1.32453E-06	2.02884E-06	4.44546E-06	9.92438E-06	carboxynorspermidine decarboxylase [EC:4.1.1.96]
K13766	0.000654876	0.000658894	0.000656014	0.000642932	methylglutaconyl-CoA hydratase [EC:4.2.1.18]
K13767	3.12105E-06	4.05904E-06	1.79452E-05	4.04193E-05	enoyl-CoA hydratase [EC:4.2.1.17]
K13770	0.000214066	0.000212139	0.000186925	0.000116236	TetR/AcrR family transcriptional regulator, fatty acid metabolism regulator protein
K13771	0.000217669	0.00021856	0.000205196	0.000159503	Rrf2 family transcriptional regulator, nitric oxide-sensitive transcriptional repressor
K13772	6.52851E-08	1.54857E-07	1.43609E-07	1.07162E-08	Rrf2 family transcriptional regulator, iron-responsive regulator
K13774	0.000215882	0.000213856	0.000192051	0.000138922	citronellol/citronellal dehydrogenase
K13775	2.91158E-06	2.10608E-06	6.34022E-06	6.08786E-05	citronellol/citronellal dehydrogenase
K13776	4.72686E-07	1.64967E-07	1.31816E-06	3.08358E-06	citronellyl-CoA synthetase [EC:6.2.1.-]
K13777	0.000224181	0.000228923	0.000251432	0.000288101	geranyl-CoA carboxylase alpha subunit [EC:6.4.1.5]
K13778	6.29471E-07	1.06263E-06	4.60905E-06	8.32353E-06	geranyl-CoA carboxylase beta subunit [EC:6.4.1.5]
K13779	1.53205E-06	3.27168E-06	1.97295E-05	3.52668E-05	isohexenylglutaconyl-CoA hydratase [EC:4.2.1.57]
K13786	4.38195E-07	1.23218E-07	4.08459E-07	6.40133E-08	cob(II)yrinic acid a,c-diamide reductase [EC:1.16.8.1]
K13787	6.04355E-06	1.1834E-05	1.4856E-05	7.34441E-06	geranylgeranyl diphosphate synthase, type I [EC:2.5.1.1 2.5.1.10 2.5.1.29]
K13788	4.6951E-06	8.44807E-06	2.33081E-05	5.32234E-05	phosphate acetyltransferase [EC:2.3.1.8]
K13789	0.000224832	0.000226079	0.000224312	0.000224224	geranylgeranyl diphosphate synthase, type II [EC:2.5.1.1 2.5.1.10 2.5.1.29]
K13790	2.26876E-11	7.55406E-09	5.68427E-08	6.72057E-10	virulence protein IcsB
K13791	1.62054E-10	4.03064E-10	1.16702E-09	4.80041E-09	invasion plasmid antigen
K13792	2.49564E-10	6.20719E-10	1.79721E-09	8.80575E-09	secreted effector OspG
K13793	3.24109E-11	8.06128E-11	2.33404E-10	9.60081E-10	secreted effector OspE
K13794	0.00021601	0.000218125	0.000203203	0.000151888	LysR family transcriptional regulator, regulatory protein for tcuABC
K13795	1.47546E-06	3.0122E-06	1.41715E-05	3.54501E-05	citrate/tricarballylate utilization protein
K13796	2.47406E-06	3.04936E-06	1.51709E-05	3.60692E-05	tricarballylate dehydrogenase
K13797	4.49993E-06	1.91642E-06	9.39368E-06	1.76743E-05	DNA-directed RNA polymerase subunit beta-beta [EC:2.7.7.6]
K13799	4.76991E-07	1.17738E-06	4.8741E-06	2.83943E-06	pantoate ligase / CMP/dCMP kinase [EC:6.3.2.1 2.7.4.25]
K13810	2.50377E-06	3.2481E-06	2.65446E-06	3.94135E-07	transaldolase / glucose-6-phosphate isomerase [EC:2.2.1.2 5.3.1.9]
K13812	1.39161E-08	2.67795E-08	6.10637E-08	9.10962E-08	bifunctional enzyme Fae/Hps [EC:4.2.1.147 4.1.2.43]
K13815	1.62605E-06	1.33506E-06	2.7659E-06	7.11584E-05	two-component system, response regulator RpfG
K13816	1.11203E-06	1.50421E-06	1.89561E-06	5.16385E-05	DSF synthase
K13818	3.68254E-07	1.99715E-07	2.73982E-07	9.3448E-06	molybdopterin-guanine dinucleotide biosynthesis protein [EC:2.7.7.77]
K13819	8.05224E-07	1.47838E-06	4.5828E-06	1.20825E-07	NifU-like protein
K13820	8.74059E-08	8.58513E-07	9.69107E-07	4.60838E-08	flagellar biosynthetic protein FliR/FlhB
K13821	0.000438001	0.000443683	0.00043208	0.000442104	RHH-type transcriptional regulator, proline utilization regulon repressor / proline dehydrogenase / delta 1-pyrroline-5-[illegible]
K13829	0.000220801	0.000221692	0.000217467	0.000173858	shikimate kinase / 3-dehydroquinate synthase [EC:2.7.1.71 4.2.3.4]
K13831	0.000214174	0.000212006	0.000186819	0.000113239	3-hexulose-6-phosphate synthase / 6-phospho-3-hexuloisomerase [EC:4.1.2.43 5.3.1.27]
K13832	4.23211E-08	3.86545E-08	0	0	3-dehydroquinate dehydratase / shikimate dehydrogenase [EC:4.2.1.10 1.1.1.25]
K13853	1.7094E-06	5.58106E-07	3.86301E-06	4.9388E-07	3-deoxy-7-phosphoheptulonate synthase / chorismate mutase [EC:2.5.1.54 5.4.99.5]
K13873	1.75578E-07	1.062E-07	1.4172E-07	8.58516E-09	L-arabinose 1-dehydrogenase [EC:1.1.1.376]
K13874	0.000429932	0.000425915	0.000384825	0.00025778	L-arabinonolactonase [EC:3.1.1.15]
K13875	5.55181E-06	3.50132E-06	1.91447E-05	3.70143E-05	L-arabonate dehydrase [EC:4.2.1.25]
K13876	2.33522E-06	3.08778E-06	1.70723E-05	4.23093E-05	2-keto-3-deoxy-L-arabinonate dehydratase [EC:4.2.1.43]
K13877	0.000216457	0.000212619	0.000191739	0.000120675	2,5-dioxopentanoate dehydrogenase [EC:1.2.1.26]
K13888	0.00022066	0.000221574	0.000227642	0.000343723	membrane fusion protein, macrolide-specific efflux system
K13889	0.000214579	0.00021247	0.000190259	0.0001173	glutathione transport system substrate-binding protein
K13890	0.000214373	0.000212785	0.000190943	0.000115019	glutathione transport system permease protein
K13891	0.000214062	0.000212309	0.000189378	0.00011485	glutathione transport system permease protein
K13892	0.000872481	0.000868904	0.000819111	0.000609236	glutathione transport system ATP-binding protein
K13893	0.00022037	0.000216792	0.000207029	0.000163846	microcin C transport system substrate-binding protein
K13894	0.000218143	0.000216244	0.00020587	0.000161864	microcin C transport system permease protein
K13895	0.000219271	0.000216193	0.000206709	0.000160662	microcin C transport system permease protein
K13896	0.000465477	0.000469346	0.000552045	0.000574273	microcin C transport system ATP-binding protein
K13918	0.000215296	0.000215002	0.000201486	0.00014969	glucarate dehydratase-related protein
K13919	7.96147E-08	2.30413E-07	5.09515E-07	9.2496E-07	propanediol dehydratase medium subunit [EC:4.2.1.28]
K13920	7.96147E-08	3.43711E-07	5.22221E-07	9.11788E-07	propanediol dehydratase small subunit [EC:4.2.1.28]
K13921	1.34776E-06	6.1138E-07	4.81618E-06	4.87973E-06	1-propanol dehydrogenase
K13922	1.21442E-06	5.5102E-07	6.63311E-06	4.73892E-07	propionaldehyde dehydrogenase [EC:1.2.1.87]
K13923	5.57553E-08	1.64148E-09	4.13098E-07	1.38784E-07	phosphate propanoyltransferase [EC:2.3.1.222]
K13924	0.001114539	0.001090865	0.001086668	0.001040421	two-component system, chemotaxis family, CheB/CheR fusion protein [EC:2.1.1.80 3.1.1.61]
K13925	4.34982E-08	0	9.85777E-09	1.59961E-07	plasmin and fibronectin-binding protein A
K13926	5.33327E-06	8.23123E-06	3.49815E-05	0.0001044	ribosome-dependent ATPase
K13927	3.99468E-07	1.64227E-08	4.44666E-07	3.58549E-06	holo-ACP synthase / triphosphoribosyl-dephospho-CoA synthase [EC:2.7.7.61 2.4.2.52]
K13928	1.30805E-06	3.06089E-06	1.18439E-05	2.84274E-05	LysR family transcriptional regulator, malonate utilization transcriptional regulator
K13929	3.3203E-06	3.44501E-06	1.3298E-05	7.98026E-05	malonate decarboxylase alpha subunit [EC:2.3.1.187]
K13930	3.44066E-06	3.44002E-06	1.33366E-05	8.39342E-05	triphosphoribosyl-dephospho-CoA synthase [EC:2.4.2.52]
K13931	3.31633E-06	3.44501E-06	1.32646E-05	7.98026E-05	malonate decarboxylase delta subunit
K13932	3.39145E-06	3.56183E-06	1.34057E-05	8.03932E-05	malonate decarboxylase beta subunit [EC:4.1.1.87]
K13933	3.31633E-06	3.44001E-06	1.32813E-05	7.97721E-05	malonate decarboxylase gamma subunit [EC:4.1.1.87]
K13934	3.32532E-06	3.43116E-06	1.32958E-05	7.98065E-05	phosphoribosyl-dephospho-CoA transferase [EC:2.7.7.66]
K13935	4.59732E-07	5.76052E-07	9.63793E-07	6.93061E-07	malonate decarboxylase epsilon subunit [EC:2.3.1.39]
K13936	5.08572E-08	1.67308E-09	2.34389E-08	5.1093E-07	malonate transporter
K13938	3.92548E-07	7.42989E-07	1.55713E-06	8.97219E-06	dihydromonapterin reductase / dihydrofolate reductase [EC:1.5.1.50 1.5.1.3]

K13940	2.08251E-06	6.22132E-06	2.47302E-06	3.77993E-06	dihydroneopterin aldolase / 2-amino-4-hydroxy-6-hydroxymethyldihydropteridine diphosphokinase [EC:4.1.2.25 2.7.6
K13941	0	4.59904E-09	0	0	2-amino-4-hydroxy-6-hydroxymethyldihydropteridine diphosphokinase / dihydropteroate synthase [EC:2.7.6.3 2.5.1.1
K13950	0.000219922	0.000224273	0.000213905	0.000160792	para-aminobenzoate synthetase [EC:2.6.1.85]
K13953	0.000443803	0.000445274	0.000456167	0.000442265	alcohol dehydrogenase, propanol-preferring [EC:1.1.1.1]
K13954	0.0002212	0.000218733	0.000210908	0.000180491	alcohol dehydrogenase [EC:1.1.1.1]
K13955	1.10063E-06	9.18456E-09	7.01108E-07	8.1233E-09	zinc-binding alcohol dehydrogenase/oxidoreductase
K13963	1.59923E-07	4.15521E-08	4.53912E-06	6.39852E-08	serpin B
K13967	2.83818E-08	5.88338E-08	9.64433E-08	2.86136E-07	N-acetylmannosamine-6-phosphate 2-epimerase / N-acetylmannosamine kinase [EC:5.1.3.9 2.7.1.60]
K13979	0.000434847	0.000429799	0.000402127	0.000330346	uncharacterized zinc-type alcohol dehydrogenase-like protein [EC:1.-.-.-]
K13985	0.000215048	0.00021218	0.000186378	0.000117238	N-acyl-phosphatidylethanolamine-hydrolysing phospholipase D [EC:3.1.4.54]
K13990	4.48394E-07	5.81263E-07	1.07562E-06	1.91542E-06	glutamate formiminotransferase / formiminotetrahydrofolate cyclodeaminase [EC:2.1.2.5 4.3.1.4]
K13991	9.7076E-07	1.11305E-08	8.07648E-08	1.22569E-07	photosynthetic reaction center H subunit
K13992	9.69047E-07	1.53301E-09	1.68437E-07	1.07355E-07	photosynthetic reaction center cytochrome c subunit
K13993	8.82023E-06	1.50683E-05	3.9543E-05	6.23837E-05	HSP20 family protein
K13995	2.0097E-06	4.47277E-06	1.96204E-05	4.1881E-05	maleamate amidohydrolase [EC:3.5.1.107]
K14028	3.99131E-06	7.14212E-08	3.68498E-07	2.82006E-07	methanol dehydrogenase (cytochrome c) subunit 1 [EC:1.1.2.7]
K14029	9.73019E-07	6.53143E-09	2.25832E-09	4.59379E-08	methanol dehydrogenase (cytochrome c) subunit 2 [EC:1.1.2.7]
K14048	7.96777E-06	9.898E-06	3.40462E-05	7.04932E-05	urease subunit gamma/beta [EC:3.5.1.5]
K14051	0.000654687	0.000651441	0.000654915	0.000561084	c-di-GMP phosphodiesterase Gmr [EC:3.1.4.52]
K14052	1.68076E-06	9.99342E-07	1.6764E-06	8.26635E-06	putrescine importer
K14053	4.21582E-09	1.91187E-08	3.7337E-08	2.37601E-07	outer membrane protein G
K14054	9.82211E-08	3.05469E-07	1.54889E-06	1.11399E-06	protein MpaA
K14055	1.18662E-07	7.59462E-07	2.54844E-06	1.91586E-05	universal stress protein E
K14056	4.38949E-07	1.19038E-06	1.89638E-06	1.12817E-05	HTH-type transcriptional regulator, repressor for puuD
K14057	1.98902E-07	3.17704E-07	4.78537E-07	8.31307E-06	LysR family transcriptional regulator, regulator of abg operon
K14058	0.000219093	0.000218451	0.000211383	0.000217576	tRNA 2-thiocytidine biosynthesis protein TtcA
K14059	2.97635E-06	3.41365E-06	1.2907E-05	4.49781E-05	integrase
K14060	0.000430386	0.000424287	0.000376918	0.000250464	putative DNA-invertase from lambdoid prophage Rac
K14061	1.71159E-07	6.26661E-07	1.23894E-07	7.25663E-07	universal stress protein F
K14062	4.88932E-07	1.49304E-06	7.9286E-07	1.02781E-05	outer membrane protein N
K14063	3.40533E-07	9.09004E-07	3.28596E-06	7.82136E-06	AraC family transcriptional regulator, positive regulator of tynA and feaB
K14064	9.82085E-08	3.00995E-07	1.39077E-07	9.54641E-07	universal stress protein C
K14065	4.36848E-09	1.83487E-08	3.14592E-08	1.94108E-07	universal stress protein D
K14067	0.000216463	0.000215895	0.000187603	0.000119982	malate-CoA ligase subunit beta [EC:6.2.1.9]
K14070	1.95804E-07	0	1.3765E-08	0	methanophenazine hydrogenase [EC:1.12.98.3]
K14080	0	0	8.90604E-08	0	[methyl-Co(III) methanol-specific corrinoid protein]:coenzyme M methyltransferase [EC:2.1.1.246]
K14081	0	0	0	6.78334E-09	methanol corrinoid protein
K14083	0	3.33228E-09	3.93637E-07	9.03326E-08	trimethylamine---corrinoid protein Co-methyltransferase [EC:2.1.1.250]
K14084	4.83296E-07	5.30276E-07	2.89471E-08	1.4263E-08	trimethylamine corrinoid protein
K14086	2.04795E-08	5.99912E-07	2.02787E-07	5.692E-08	ech hydrogenase subunit A
K14087	0	6.19804E-08	1.03238E-08	0	ech hydrogenase subunit B
K14088	1.19151E-08	8.11046E-07	1.90911E-07	4.83369E-08	ech hydrogenase subunit C
K14089	0	3.86545E-08	0	0	ech hydrogenase subunit D
K14090	0	6.19804E-08	8.51666E-08	0	ech hydrogenase subunit E
K14091	0	3.86545E-08	0	0	ech hydrogenase subunit F
K14105	0	6.19804E-08	6.97714E-09	5.4955E-10	energy-converting hydrogenase A subunit N
K14106	0	0	6.20514E-08	0	energy-converting hydrogenase A subunit O
K14120	0	4.33197E-08	7.6371E-09	0	energy-converting hydrogenase B subunit K
K14126	3.63932E-08	5.67111E-08	1.30949E-07	8.7269E-08	F420-non-reducing hydrogenase large subunit [EC:1.12.99.- 1.8.98.5]
K14127	0	0	6.90285E-08	0	F420-non-reducing hydrogenase iron-sulfur subunit [EC:1.12.99.- 1.8.98.5 1.8.98.6]
K14128	3.23946E-08	2.6458E-08	9.64887E-08	1.12741E-08	F420-non-reducing hydrogenase small subunit [EC:1.12.99.- 1.8.98.5]
K14136	4.94055E-07	1.76991E-06	1.05377E-05	1.64558E-05	decaprenyl-phosphate phosphoribosyltransferase [EC:2.4.2.45]
K14153	0.000215608	0.000215988	0.000207755	0.00016481	hydroxymethylpyrimidine kinase / phosphomethylpyrimidine kinase / thiamine-phosphate diphosphorylase [EC:2.7.1.4
K14155	0.000432492	0.000430142	0.000389837	0.000242848	cystathione beta-lyase [EC:4.4.1.8]
K14157	0	0	5.23286E-09	0	alpha-aminoadipic semialdehyde synthase [EC:1.5.1.8 1.5.1.9]
K14159	1.51549E-06	1.91356E-08	1.10704E-06	5.86797E-08	ribonuclease HI / DNA polymerase III subunit epsilon [EC:3.1.26.4 2.7.7.7]
K14160	0.000218611	0.000216919	0.000208063	0.000201602	protein ImuA
K14161	0.000219707	0.000217925	0.0002162	0.000208032	protein ImuB
K14162	0.00043459	0.000434049	0.000406938	0.000321758	error-prone DNA polymerase [EC:2.7.7.7]
K14164	2.64885E-07	4.99842E-09	6.75198E-07	2.8686E-07	glycyl-tRNA synthetase [EC:6.1.1.14]
K14165	1.20338E-06	2.99112E-06	1.37837E-05	3.47448E-05	atypical dual specificity phosphatase [EC:3.1.3.16 3.1.3.48]
K14166	1.02431E-06	1.63151E-07	3.65795E-06	3.04086E-06	copper transport protein
K14170	0.00021895	0.000217744	0.000211779	0.000220054	chorismate mutase / prephenate dehydratase [EC:5.4.99.5 4.2.1.51]
K14187	9.82118E-08	6.32432E-07	3.69488E-07	1.73158E-05	chorismate mutase / prephenate dehydrogenase [EC:5.4.99.5 1.3.1.12]
K14188	2.3742E-06	3.07663E-06	1.83521E-05	3.83535E-05	D-alanine--poly(phosphoribitol) ligase subunit 2 [EC:6.1.1.13]
K14189	0.000216792	0.000215711	0.000196394	0.000153504	uncharacterized oxidoreductase [EC:1.-.-.-]
K14192	1.36825E-07	0	1.97133E-08	3.59746E-07	clumping factor B
K14193	4.97545E-08	0	6.69921E-09	0	iron-regulated surface determinant protein A
K14194	2.78444E-06	1.30352E-08	8.41613E-06	2.3761E-07	serine-aspartate repeat-containing protein C/D/E
K14195	1.0547E-06	0	3.32446E-06	4.62565E-08	surface protein G
K14196	1.24386E-08	0	1.6748E-09	0	immunoglobulin G-binding protein A
K14197	1.24386E-08	0	1.6748E-09	0	immunoglobulin G-binding protein Sbi
K14198	6.83365E-09	0	2.85396E-08	1.64664E-07	staphylokinase
K14199	2.48773E-08	0	3.3496E-09	0	staphylococcal complement inhibitor
K14200	3.73159E-08	0	5.02441E-09	0	fibrinogen-binding protein
K14201	4.71662E-08	1.83961E-09	1.87894E-08	1.41319E-08	clumping factor A
K14203	1.24386E-08	0	1.6748E-09	0	FPRL1 inhibitory protein
K14204	2.48773E-08	0	3.3496E-09	0	protein Map
K14205	0.000217837	0.000212742	0.000193923	0.000188007	phosphatidylglycerol lysyltransferase [EC:2.3.2.3]
K14215	8.65702E-07	3.03614E-06	1.49456E-06	1.60421E-07	trans,polycis-decaprenyl diphosphate synthase [EC:2.5.1.86]
K14250	5.24217E-08	4.92932E-08	4.68189E-07	2.2342E-09	cyclase
K14251	0	0	1.05134E-07	1.78604E-09	C-methyltransferase [EC:2.1.1.-]
K14252	2.7416E-07	4.95598E-07	2.16103E-06	1.05147E-05	6-methylpretetramide 4-monooxygenase / 4-hydroxy-6-methylpretetramide 12a-monooxygenase [EC:1.14.13.232 1.14
K14253	0	0	2.67528E-07	1.32216E-08	6-methylpretetramide 4-monooxygenase [EC:1.14.13.232]
K14254	8.99101E-09	1.6291E-07	1.50585E-06	1.82301E-06	aminotransferase
K14256	6.43051E-09	2.83436E-08	2.70919E-07	3.90597E-08	anhydrotetracycline 6-monooxygenase / 5a,11a-dehydrotetracycline 5-monooxygenase [EC:1.14.13.38 1.14.13.234]
K14257	1.54063E-09	1.32166E-08	6.35471E-08	1.16092E-08	tetracycline 7-halogenase / FADH2 O2-dependent halogenase [EC:1.14.19.49 1.14.19.-]

K14259	5.44156E-07	5.38257E-07	6.21848E-06	1.92544E-06	2-dehydro-3-deoxy-D-arabinonate dehydratase [EC:4.2.1.141]
K14260	0.000220663	0.00022409	0.000211912	0.000226456	alanine-synthesizing transaminase [EC:2.6.1.66 2.6.1.2]
K14261	5.10548E-06	3.70597E-06	1.25735E-05	1.52241E-06	alanine-synthesizing transaminase [EC:2.6.1.-]
K14266	1.05673E-06	4.95631E-07	2.9771E-06	1.37619E-05	tryptophan 7-halogenase [EC:1.14.19.9]
K14267	0.000221609	0.000224608	0.000227048	0.000201749	N-succinyldiaminopimelate aminotransferase [EC:2.6.1.17]
K14268	2.2251E-07	5.8876E-07	1.90554E-06	5.40741E-06	5-aminovalerate/4-aminobutyrate aminotransferase [EC:2.6.1.48 2.6.1.19]
K14273	1.61901E-06	3.93162E-07	1.70852E-06	4.02601E-05	D-xylose 1-dehydrogenase (NADP+) [EC:1.1.1.179]
K14274	0.000219171	0.000220444	0.000220143	0.000207214	xylonolactonase [EC:3.1.1.-]
K14275	9.16819E-09	0	2.86391E-08	0	D-xylonate dehydratase [EC:4.2.1.82]
K14287	0.000221833	0.000221006	0.000211428	0.000210317	methionine transaminase [EC:2.6.1.88]
K14292	0	0	1.04657E-08	1.0175E-08	trimethylguanosine synthase [EC:2.1.1.-]
K14331	0	8.50664E-11	0	0	fatty aldehyde decarbonylase [EC:4.1.99.5]
K14333	2.70705E-07	2.36986E-07	2.48933E-07	4.85118E-07	2,3-dihydroxybenzoate decarboxylase [EC:4.1.1.46]
K14335	2.77691E-06	1.6134E-06	1.09389E-05	3.89527E-06	alpha-1,6-mannosyltransferase [EC:2.4.1.-]
K14337	2.12264E-06	3.83203E-06	1.45764E-05	1.90799E-06	alpha-1,6-mannosyltransferase [EC:2.4.1.-]
K14338	2.89546E-07	5.60825E-07	6.5765E-07	3.92208E-06	cytochrome P450 / NADPH-cytochrome P450 reductase [EC:1.14.14.1 1.6.2.4]
K14339	9.56972E-07	3.70305E-06	4.92349E-06	1.90288E-06	alpha-1,6-mannosyltransferase [EC:2.4.1.-]
K14340	2.12172E-08	5.06983E-08	6.21789E-08	8.98251E-08	mannosyltransferase [EC:2.4.1.-]
K14347	0.00021739	0.000217594	0.000214335	0.00021024	solute carrier family 10 (sodium/bile acid cotransporter), member 7
K14348	3.88895E-06	4.12211E-06	1.85603E-05	8.71638E-05	GntR family transcriptional regulator, L-lactate dehydrogenase operon regulator
K14358	0	0	0	3.48277E-08	secernin
K14367	0	0	6.16974E-08	0	erythronolide mycarosyltransferase [EC:2.4.1.328]
K14368	0	0	7.78864E-08	0	3-alpha-mycarosylerythronolide B desosaminyl transferase [EC:2.4.1.278]
K14371	1.15547E-09	4.0907E-08	2.56776E-06	1.26733E-07	type I polyketide synthase AVES
K14379	2.48711E-07	4.63728E-07	2.76368E-07	3.8705E-06	tartrate-resistant acid phosphatase type 5 [EC:3.1.3.2]
K14392	1.81755E-06	3.42258E-06	1.66869E-05	4.14208E-05	sodium/pantothenate symporter
K14393	0.000223379	0.000224626	0.000224987	0.000209174	cation/acetate symporter
K14414	0.000433203	0.000431465	0.000407465	0.000327962	transcriptional regulatory protein RtcR
K14415	0.000220165	0.000223078	0.000237388	0.000284615	tRNA-splicing ligase RtcB (3-phosphate/5-hydroxy nucleic acid ligase) [EC:6.5.1.8]
K14424	0	0	0	8.03716E-09	methylsterol monooxygenase 2
K14441	0.000223089	0.000220151	0.000223136	0.000204493	ribosomal protein S12 methylthiotransferase [EC:2.8.4.4]
K14445	2.62177E-06	8.87339E-06	6.96048E-06	4.41524E-05	solute carrier family 13 (sodium-dependent dicarboxylate transporter), member 2/3/5
K14446	9.78736E-07	2.88342E-08	4.71977E-07	1.85732E-07	crotonyl-CoA carboxylase/reductase [EC:1.3.1.85]
K14447	1.07157E-06	2.04156E-07	1.30408E-06	4.41136E-07	ethylmalonyl-CoA mutase [EC:5.4.99.63]
K14448	7.88071E-06	1.30171E-05	6.46517E-05	0.000144256	(2S)-methylsuccinyl-CoA dehydrogenase [EC:1.3.8.12]
K14449	0.000215012	0.000212544	0.000189338	0.000120288	2-methylfumaryl-CoA hydratase [EC:4.2.1.148]
K14451	8.68231E-08	2.18358E-07	1.50501E-07	3.61382E-08	(3S)-malyl-CoA thioesterase [EC:3.1.2.30]
K14465	0	1.83961E-09	1.81612E-08	1.60743E-08	succinate semialdehyde reductase (NADPH) [EC:1.1.1.-]
K14466	0.000214744	0.000211782	0.000186406	0.000113266	4-hydroxybutyrate---CoA ligase (AMP-forming) [EC:6.2.1.40]
K14467	3.97169E-09	4.99842E-09	1.10312E-08	2.84475E-08	4-hydroxybutyrate---CoA ligase (AMP-forming) [EC:6.2.1.40]
K14468	0	2.55502E-09	1.44647E-08	0	malonyl-CoA reductase / 3-hydroxypropionate dehydrogenase (NADP+) [EC:1.2.1.75 1.1.1.298]
K14469	0	1.18232E-08	9.88709E-08	0	acrylyl-CoA reductase (NADPH) / 3-hydroxypropionyl-CoA dehydratase / 3-hydroxypropionyl-CoA synthetase [EC:1
K14470	1.06342E-06	2.69942E-06	1.17414E-05	3.57507E-05	2-methylfumaryl-CoA isomerase [EC:5.4.1.3]
K14471	7.2975E-08	1.33685E-07	1.13306E-07	6.5007E-09	succinyl-CoA:(S)-malate CoA-transferase subunit A [EC:2.8.3.22]
K14472	1.10399E-06	2.00279E-07	1.49534E-06	3.65754E-07	succinyl-CoA:(S)-malate CoA-transferase subunit B [EC:2.8.3.22]
K14475	2.2222E-07	4.43094E-07	1.69977E-06	4.80007E-07	inhibitor of cysteine peptidase
K14519	0.000215223	0.000212487	0.00019035	0.000117193	NADP-dependent aldehyde dehydrogenase [EC:1.2.1.4]
K14520	1.31648E-06	1.05424E-06	4.19198E-06	1.41679E-05	4-hydroxyacetophenone monooxygenase [EC:1.14.13.84]
K14534	2.24697E-06	8.50957E-07	2.05977E-06	3.85187E-07	4-hydroxybutyryl-CoA dehydratase / vinylacetyl-CoA-Delta-isomerase [EC:4.2.1.120 5.3.3.3]
K14540	3.55406E-06	3.88965E-06	2.4958E-05	5.61675E-05	ribosome biogenesis GTPase A
K14578	0.000214141	0.00021354	0.000190452	0.000122496	naphthalene 1,2-dioxygenase ferredoxin component
K14579	2.44759E-07	5.99713E-07	2.74431E-06	6.9361E-06	naphthalene 1,2-dioxygenase subunit alpha [EC:1.14.12.12 1.14.12.23 1.14.12.24]
K14580	8.66604E-11	1.39614E-08	0	1.88426E-09	naphthalene 1,2-dioxygenase subunit beta [EC:1.14.12.12 1.14.12.23 1.14.12.24]
K14581	0.000213837	0.000211857	0.000187454	0.000113636	naphthalene 1,2-dioxygenase ferredoxin reductase component [EC:1.18.1.7]
K14582	2.40907E-07	6.10542E-07	2.77171E-06	6.9449E-06	cis-1,2-dihydro-1,2-dihydroxynaphthalene/dibenzothiophene dihydrodiol dehydrogenase [EC:1.3.1.29 1.3.1.60]
K14583	2.44759E-07	5.99713E-07	2.85952E-06	7.11315E-06	1,2-dihydroxynaphthalene dioxygenase [EC:1.13.11.56]
K14584	9.16898E-07	1.08249E-06	5.09207E-06	4.91211E-05	2-hydroxychromene-2-carboxylate isomerase [EC:5.99.1.4]
K14585	4.85434E-07	1.20675E-06	5.63981E-06	1.38878E-05	trans-o-hydroxybenzylidenepyruvate hydratase-aldolase [EC:4.1.2.45]
K14587	1.50413E-09	3.74111E-09	1.08319E-08	4.45558E-08	protein sgcE [EC:5.1.3.-]
K14588	0.000217163	0.000216787	0.000190296	0.0001395	blue copper oxidase
K14591	7.60473E-08	2.00841E-07	2.0524E-07	7.78819E-07	protein AroM
K14596	2.06044E-07	4.34037E-07	1.65219E-07	4.01659E-08	zeaxanthin glucosyltransferase [EC:2.4.1.276]
K14597	0	3.33228E-09	4.93729E-07	2.70295E-09	chlorobactene glucosyltransferase
K14598	5.89134E-08	0	0	0	chlorobactene lauroyltransferase
K14599	2.27728E-09	1.2742E-07	5.27665E-08	2.32901E-07	dibenzofuran dioxygenase subunit alpha [EC:1.14.12.-]
K14600	5.39461E-08	2.83838E-08	4.7951E-08	5.65278E-09	dibenzofuran dioxygenase subunit beta [EC:1.14.12.-]
K14605	0	0	2.06475E-09	0	lycopene cyclase CruA [EC:5.5.1.19]
K14623	1.11624E-06	1.65219E-06	2.09089E-06	6.30702E-07	DNA-damage-inducible protein D
K14626	0	0	4.55139E-08	0	actinorhodin biosynthesis protein ActVIA
K14627	0	1.13298E-07	7.3146E-08	0	dehydratase [EC:4.2.1.-]
K14628	0	0	2.55829E-08	0	enoyl reductase
K14630	0	0	1.95625E-07	0	two-component flavin-dependent monooxygenase [EC:1.14.14.-]
K14631	5.67369E-09	9.23083E-09	3.46614E-07	8.74978E-08	flavin reductase ActVB [EC:1.5.1.-]
K14633	1.23434E-06	2.92133E-08	4.39054E-07	7.96899E-08	ketoreductase RED2 [EC:1.1.1.-]
K14645	0.000648166	0.000647333	0.000590347	0.000530355	serine protease [EC:3.4.21.-]
K14647	1.24731E-06	1.28078E-07	2.76705E-06	7.36446E-06	minor extracellular serine protease Vpr [EC:3.4.21.-]
K14652	0.000439624	0.000436853	0.000420084	0.000363253	3,4-dihydroxy 2-butanone 4-phosphate synthase / GTP cyclohydrolase II [EC:4.1.99.12 3.5.4.25]
K14654	1.05886E-06	3.64206E-07	1.02512E-06	1.64055E-07	2,5-diamino-6-(ribosylamino)-4(3H)-pyrimidinone 5-phosphate reductase [EC:1.1.1.302]
K14656	1.83036E-08	3.57748E-08	0	3.44244E-07	FAD synthetase [EC:2.7.7.2]
K14657	1.07952E-06	9.08409E-08	1.35826E-06	1.20615E-06	LysR family transcriptional regulator, nod-box dependent transcriptional activator
K14658	1.92665E-08	0	8.48987E-08	1.56275E-08	nodulation protein A [EC:2.3.1.-]
K14659	1.8932E-07	3.90527E-07	4.82115E-07	5.40932E-08	chitooligosaccharide deacetylase [EC:3.5.1.-]
K14660	0.000214503	0.000213791	0.000190626	0.000121385	nodulation protein E [EC:2.3.1.-]
K14661	0	0	8.78058E-08	0	nodulation protein F [EC:2.3.1.-]
K14665	2.39245E-06	2.52442E-07	7.67172E-06	4.99118E-06	amidohydrolase [EC:3.5.1.-]
K14666	1.11317E-08	0	6.86187E-08	0	N-acetylglucosaminyltransferase [EC:2.4.1.-]
K14667	2.88275E-07	5.8937E-07	2.50376E-06	4.18911E-07	minimal PKS ketosynthase (KS/KS alpha) [EC:2.3.1.-]

K14668	0	0	2.14861E-07	1.87534E-08	minimal PKS chain-length factor (CLF/KS beta) [EC:2.3.1.-]
K14669	0	0	8.41597E-08	8.93018E-09	minimal PKS acyl carrier protein
K14670	0	0	1.26247E-07	1.69673E-08	aromatase
K14671	0	0	1.61258E-07	2.58975E-08	cyclase
K14672	2.1195E-08	2.42423E-07	5.20326E-07	1.96274E-06	putative polyketide hydroxylase
K14673	0	0	1.24752E-07	1.69673E-08	putative monooxygenase
K14680	0	0	1.1497E-08	1.8761E-07	RNA ligase [EC:6.5.1.3]
K14681	1.5077E-06	5.54809E-07	3.43154E-06	1.78731E-05	argininosuccinate lyase / amino-acid N-acetyltransferase [EC:4.3.2.1 2.3.1.1]
K14682	0.000216938	0.000216018	0.00020561	0.000162609	amino-acid N-acetyltransferase [EC:2.3.1.1]
K14683	3.50005E-08	5.08595E-07	9.42731E-07	1.3194E-05	solute carrier family 34 (sodium-dependent phosphate cotransporter)
K14696	1.7807E-07	1.31839E-08	1.52269E-08	4.72462E-08	solute carrier family 30 (zinc transporter), member 9
K14698	0.000428055	0.000423973	0.000378572	0.000231618	ATP-binding cassette, subfamily B, bacterial IrtA [EC:3.6.3.-]
K14699	3.43882E-07	2.22111E-07	5.87547E-07	3.35144E-06	ATP-binding cassette, subfamily B, bacterial IrtB [EC:3.6.3.-]
K14727	0.000216685	0.000215732	0.000205237	0.000186084	3-oxoadipate enol-lactonase / 4-carboxymuconolactone decarboxylase [EC:3.1.1.24 4.1.1.44]
K14728	4.98787E-09	5.10611E-08	1.42095E-08	5.66705E-08	phthiodiolone/phenolphthiodiolone dimycocerosates ketoreductase [EC:1.2.-.-]
K14730	3.63011E-08	1.17741E-07	1.17622E-07	7.99731E-06	(-)-trans-carveol dehydrogenase [EC:1.1.1.243]
K14731	0.000221345	0.000221487	0.000233437	0.000231194	epsilon-lactone hydrolase [EC:3.1.1.83]
K14733	1.13378E-07	3.14698E-07	7.93039E-07	2.60942E-06	limonene 1,2-monooxygenase [EC:1.14.13.107]
K14742	0.000223062	0.000223344	0.000222026	0.000224822	tRNA threonylcarbamoyladenosine biosynthesis protein TsaB
K14743	5.30627E-07	1.84885E-06	4.77661E-06	4.23147E-05	membrane-anchored mycosin MYCP [EC:3.4.21.-]
K14744	5.36122E-08	1.13617E-07	1.87297E-07	2.70299E-06	prophage endopeptidase [EC:3.4.-.-]
K14748	3.87824E-07	7.2368E-07	3.67853E-06	8.63493E-06	ethylbenzene dioxygenase subunit alpha [EC:1.14.12.-]
K14749	6.84615E-08	6.58241E-08	1.88226E-07	2.29146E-07	ethylbenzene dioxygenase subunit beta [EC:1.14.12.-]
K14750	1.30111E-08	1.67379E-08	2.08464E-07	1.00465E-08	ethylbenzene dioxygenase ferredoxin component
K14751	2.4472E-07	5.85814E-07	2.85887E-06	6.9283E-06	2,3-dihydroxyethylbenzene 1,2-dioxygenase [EC:1.13.11.-]
K14761	0.000218654	0.000217755	0.000204786	0.000188141	ribosome-associated protein
K14762	9.81988E-08	3.14549E-07	1.39007E-07	9.97393E-06	ribosome-associated protein
K14940	6.33816E-08	3.82716E-08	6.47032E-08	3.4766E-07	gamma-F420-2:alpha-L-glutamate ligase [EC:6.3.2.32]
K14941	2.4272E-07	1.5236E-07	7.85031E-07	3.79776E-06	2-phospho-L-lactate guanylyltransferase [EC:2.7.7.68]
K14949	0	6.06651E-07	2.87837E-06	1.79993E-06	serine/threonine-protein kinase PknG [EC:2.7.11.1]
K14952	6.49351E-08	1.47781E-07	9.88915E-08	3.5835E-06	UDP-MurNAc hydroxylase
K14953	0	2.79815E-07	3.61784E-08	1.58298E-06	ipoprotein LpqH
K14954	0	2.7954E-07	1.4021E-07	9.4331E-08	lipoprotein LprG
K14955	0	0	0	1.52139E-08	lipoprotein LprA
K14956	0	1.23941E-07	1.54857E-08	1.08019E-08	6 kDa early secretory antigenic target
K14974	1.54339E-06	3.41828E-06	1.6136E-05	4.0286E-05	6-hydroxynicotinate 3-monooxygenase [EC:1.14.13.114]
K14977	1.13976E-06	8.61477E-07	1.61674E-06	9.69218E-07	(S)-ureidoglycine aminohydrolase [EC:3.5.3.26]
K14978	9.01E-06	6.73311E-06	3.27081E-05	9.13684E-05	two-component system, LuxR family, secretion system sensor histidine kinase SsrA
K14979	3.56429E-06	4.14206E-06	1.66788E-05	8.69976E-05	two-component system, LuxR family, secretion system response regulator SsrB
K14980	3.51591E-06	1.1693E-06	6.48547E-06	1.12663E-05	two-component system, OmpR family, sensor histidine kinase ChvG [EC:2.7.13.3]
K14981	2.72009E-06	2.02949E-06	3.53094E-06	1.46876E-05	two-component system, OmpR family, response regulator ChvI
K14982	5.14535E-06	9.4363E-06	2.6046E-05	8.31794E-05	two-component system, OmpR family, sensor histidine kinase CiaH [EC:2.7.13.3]
K14983	9.85654E-07	1.45987E-06	5.80635E-06	9.44052E-06	two-component system, OmpR family, response regulator CiaR
K14986	0.000225547	0.000229972	0.000261499	0.00034241	two-component system, LuxR family, sensor kinase FixL [EC:2.7.13.3]
K14987	0.000651382	0.000649649	0.000613407	0.000480157	two-component system, LuxR family, response regulator FixJ
K14988	6.32701E-09	0	1.09761E-08	2.80633E-08	two-component system, NarL family, secretion system sensor histidine kinase SalK
K14989	2.01132E-07	6.64661E-08	4.45122E-08	8.10598E-08	two-component system, NarL family, secretion system response regulator SalR
K14998	0.000220506	0.000219265	0.000223279	0.000243515	surfeit locus 1 family protein
K15011	0.000219415	0.000217247	0.000207894	0.000204479	two-component system, sensor histidine kinase RegB [EC:2.7.13.3]
K15012	0.000222167	0.000222784	0.000236739	0.000279124	two-component system, response regulator RegA
K15016	0.000220356	0.000220207	0.000217731	0.000241885	enoyl-CoA hydratase / 3-hydroxyacyl-CoA dehydrogenase [EC:4.2.1.17 1.1.1.35]
K15018	0	6.35361E-07	2.05366E-07	7.63809E-08	3-hydroxypropionyl-coenzyme A synthetase [EC:6.2.1.36]
K15019	2.98802E-07	1.00913E-06	5.85746E-07	3.17819E-06	3-hydroxypropionyl-coenzyme A dehydratase [EC:4.2.1.116]
K15020	2.7633E-07	6.0076E-07	2.844E-06	8.64573E-06	acryloyl-coenzyme A reductase [EC:1.3.1.84]
K15022	1.30293E-06	2.47E-07	2.01387E-06	1.95083E-06	formate dehydrogenase (NADP+) beta subunit [EC:1.17.1.10]
K15023	0	1.66614E-09	0	0	5-methyltetrahydrofolate corrinoid/iron sulfur protein methyltransferase [EC:2.1.1.258]
K15024	4.18473E-07	1.78746E-07	1.33895E-06	3.42131E-06	putative phosphotransacetylase [EC:2.3.1.8]
K15034	1.2855E-05	2.24852E-05	5.42309E-05	0.000218721	ribosome-associated protein
K15036	1.19581E-06	1.87639E-06	9.07598E-06	2.0546E-07	acetyl-CoA/propionyl-CoA carboxylase [EC:6.4.1.2 6.4.1.3 2.1.3.15]
K15038	6.77083E-08	1.11794E-06	1.3351E-06	6.21531E-07	succinyl-CoA reductase [EC:1.2.1.76]
K15039	3.44213E-08	0	3.56514E-08	0	3-hydroxypropionate dehydrogenase (NADP+) [EC:1.1.1.298]
K15045	8.99101E-09	1.24417E-08	8.10434E-09	1.05615E-06	radical S-adenosyl methionine domain-containing protein 2
K15051	8.06399E-07	3.39246E-07	4.37257E-06	6.61811E-06	DNA-entry nuclease
K15052	1.19706E-06	7.52126E-08	8.82338E-07	1.20376E-07	propionyl-CoA carboxylase [EC:6.4.1.3 2.1.3.15]
K15054	1.40594E-06	3.9848E-06	1.80435E-05	3.73926E-05	(S)-mandelate dehydrogenase [EC:1.1.99.31]
K15058	7.2217E-11	2.69919E-10	1.54806E-09	0	2-aminophenol/2-amino-5-chlorophenol 1,6-dioxygenase subunit alpha
K15059	7.2217E-11	2.69919E-10	1.54806E-09	0	2-aminophenol/2-amino-5-chlorophenol 1,6-dioxygenase subunit beta [EC:1.13.11.74 1.13.11.76]
K15060	2.76631E-07	7.97371E-07	2.90626E-06	7.09265E-06	5,5-dehydrodivanillate O-demethylase
K15064	1.59354E-08	1.18379E-10	1.8058E-07	1.90566E-06	syringate O-demethylase [EC:2.1.1.-]
K15065	4.48335E-09	0	0	7.60696E-09	3-O-methylgallate 3,4-dioxygenase [EC:1.13.11.-]
K15066	1.59354E-08	2.95948E-10	3.66307E-07	1.97435E-06	vanillate/3-O-methylgallate O-demethylase [EC:2.1.1.341]
K15067	8.01056E-08	7.29406E-07	5.16007E-07	4.0199E-06	2-aminomuconate deaminase [EC:3.5.99.5]
K15125	0.001287504	0.001277338	0.001141094	0.000833085	filamentous hemagglutinin
K15226	2.94567E-08	0	0	1.35667E-08	arogenate dehydrogenase (NADP+) [EC:1.3.1.78]
K15228	8.41872E-08	5.33472E-08	6.91013E-07	4.49004E-06	methylamine dehydrogenase light chain [EC:1.4.9.1]
K15229	8.41872E-08	5.33472E-08	6.63584E-07	2.80646E-06	methylamine dehydrogenase heavy chain [EC:1.4.9.1]
K15230	4.39927E-09	1.84253E-08	8.20524E-08	1.91714E-06	ATP-citrate lyase alpha-subunit [EC:2.3.3.8]
K15231	0	0	5.03714E-08	0	ATP-citrate lyase beta-subunit [EC:2.3.3.8]
K15234	0	0	2.09314E-08	0	citryl-CoA lyase [EC:4.1.3.34]
K15241	4.81447E-11	6.15667E-11	0	2.11979E-09	tetrachloro-p-hydroquinone reductive dehalogenase [EC:2.5.1.-]
K15242	0	0	1.16882E-07	0	2,6-dichloro-p-hydroquinone 1,2-dioxygenase [EC:1.13.11.-]
K15253	4.96461E-09	0	9.30286E-09	0	chlorocatechol 1,2-dioxygenase [EC:1.13.11.-]
K15256	5.01642E-06	4.83105E-06	2.18613E-05	0.000107088	tRNA (cmo5U34)-methyltransferase [EC:2.1.1.-]
K15257	1.4922E-06	7.77857E-07	2.8496E-06	1.73907E-05	tRNA (mo5U34)-methyltransferase [EC:2.1.1.-]
K15268	0.000217043	0.000216144	0.000205288	0.000166965	O-acetylserine/cysteine efflux transporter
K15269	0.000215224	0.000215658	0.000201863	0.000162676	probable blue pigment (indigoidine) exporter
K15270	0.000215281	0.00021564	0.000204062	0.000148363	S-adenosylmethionine uptake transporter

K15303	0	0	2.00271E-07	9.56805E-09	aflatoxin B1 aldehyde reductase
K15311	3.25157E-08	6.65937E-09	2.75413E-07	1.14762E-08	polyketide biosynthesis 3-hydroxy-3-methylglutaryl-CoA synthase-like enzyme PksG
K15312	3.24916E-08	6.62858E-09	1.71518E-07	3.88463E-08	polyketide biosynthesis enoyl-CoA hydratase PksH
K15313	9.74027E-08	1.97934E-08	2.836E-07	0	polyketide biosynthesis enoyl-CoA hydratase PksI
K15314	2.59564E-07	6.8364E-07	6.08318E-06	9.38981E-06	enediyne polyketide synthase
K15315	9.69047E-07	1.53301E-09	6.80078E-08	2.22578E-09	enediyne core biosynthesis thioesterase
K15320	0.000429137	0.000425322	0.000383456	0.000244124	6-methylsalicylic acid synthase [EC:2.3.1.165]
K15327	3.24676E-08	6.5978E-09	2.44154E-09	0	polyketide biosynthesis malonyl-CoA-[acyl-carrier-protein] transacylase
K15328	6.49592E-08	1.44056E-08	3.47648E-07	7.51615E-08	bacillaene synthase trans-acting acyltransferase
K15329	3.36827E-08	2.03659E-08	1.22542E-06	1.48404E-06	trans-AT polyketide synthase, acyltransferase and oxidoreductase domains
K15337	3.24916E-08	6.62858E-09	2.08077E-07	0	polyketide biosynthesis acyl carrier protein
K15342	6.47358E-06	5.16883E-06	4.15471E-05	4.65048E-05	CRISP-associated protein Cas1
K15344	2.83595E-12	7.50469E-09	1.35558E-08	6.1544E-09	secreted effector protein SseB
K15345	2.83595E-12	7.50469E-09	1.01719E-08	5.62583E-09	secreted effector protein SseC
K15346	2.83595E-12	7.50469E-09	1.01719E-08	5.62583E-09	secreted effector protein SseD
K15347	2.83595E-12	7.05362E-12	2.04229E-11	3.3813E-09	secreted effector protein SifA
K15349	2.83595E-12	7.05362E-12	2.04229E-11	4.28001E-08	secreted effector protein SseJ
K15350	2.83595E-12	7.05362E-12	2.04229E-11	5.62583E-09	secreted effector protein SseF
K15351	2.83595E-12	7.05362E-12	2.04229E-11	3.86835E-10	secreted effector protein SseG
K15352	3.74605E-07	6.02335E-08	2.69647E-07	4.860082E-07	secreted effector protein PipB2
K15353	2.50134E-08	4.50424E-10	1.17699E-08	5.36445E-09	E3 ubiquitin-protein ligase SspH2
K15354	2.83595E-12	7.05362E-12	2.04229E-11	8.40071E-11	secreted effector protein SseI
K15355	3.25398E-08	1.86483E-08	4.39813E-07	2.86593E-08	malonyl CoA-acyl carrier protein transacylase
K15357	1.51638E-06	3.46747E-06	1.62501E-05	3.48124E-05	N-formylmaleamate deformylase [EC:3.5.1.106]
K15358	3.94347E-08	7.09374E-08	2.04787E-07	0	enamidase [EC:3.5.2.18]
K15359	0	2.03446E-10	8.00918E-09	0	6-hydroxy-3-succinoylpyridine 3-monooxygenase [EC:1.14.13.163]
K15366	0	6.29491E-10	0	0	salmonella plasmid virulence protein B
K15367	2.83595E-12	7.05362E-12	2.04229E-11	5.09726E-09	secretion system chaperone SseA
K15368	1.00109E-09	9.98756E-09	1.73608E-08	5.4748E-08	secretion system chaperone SscA
K15371	3.75868E-06	2.89822E-06	1.05261E-05	7.77943E-05	glutamate dehydrogenase [EC:1.4.1.2]
K15372	0.000431818	0.000431157	0.000394685	0.000351253	taurine---2-oxoglutarate transaminase [EC:2.6.1.55]
K15373	1.39401E-06	2.46029E-06	2.59492E-06	5.20393E-05	sulfoacetaldehyde reductase [EC:1.1.1.313]
K15383	5.48969E-07	8.40489E-07	7.9244E-07	6.79196E-07	MtN3 and saliva related transmembrane protein
K15395	1.01171E-06	4.1507E-07	2.07902E-06	5.72409E-07	hybrid polyketide synthase / nonribosomal peptide synthetase FtdB
K15396	0.000217455	0.000213984	0.000190817	0.000183602	tRNA (cytidine32/uridine32-2-O)-methyltransferase [EC:2.1.1.200]
K15408	2.09517E-06	3.05276E-06	1.01459E-05	1.68318E-05	cytochrome c oxidase subunit I+III [EC:1.9.3.1]
K15429	0	0	1.04657E-08	1.29419E-08	tRNA (guanine37-N1)-methyltransferase [EC:2.1.1.228]
K15431	0	6.46505E-10	0	5.28572E-10	phloroglucinol synthase [EC:2.3.1.253]
K15460	1.37033E-06	2.72504E-06	4.28028E-06	2.03909E-05	tRNA1Val (adenine37-N6)-methyltransferase [EC:2.1.1.223]
K15461	0.000217138	0.000216358	0.000206845	0.000166323	tRNA 5-methylaminomethyl-2-thiouridine biosynthesis bifunctional protein [EC:2.1.1.61 1.5.-.-]
K15466	0	0	1.27285E-08	0	amide synthase
K15468	1.10752E-06	3.82454E-07	3.43956E-06	1.22675E-06	cytochrome P450 PksS
K15469	0	0	1.57245E-07	0	rhizoxin biosynthesis acyltransferase
K15471	9.16819E-09	0	0	0	O-methyltransferase [EC:2.1.1.-]
K15495	2.9944E-08	6.32401E-08	3.22571E-08	9.45845E-08	molybdate/tungstate transport system substrate-binding protein
K15496	2.59143E-07	1.48579E-07	5.9404E-07	3.85821E-06	molybdate/tungstate transport system permease protein
K15497	7.20841E-06	7.71428E-06	3.72986E-05	7.62798E-05	molybdate/tungstate transport system ATP-binding protein [EC:3.6.3.- 3.6.3.55]
K15502	0	1.99937E-08	0	0	serine/threonine-protein phosphatase 6 regulatory ankyrin repeat subunit A
K15509	7.70673E-07	7.26042E-07	1.17249E-06	1.96636E-06	sulfopropanediol 3-dehydrogenase [EC:1.1.1.308]
K15510	3.43163E-07	4.52017E-07	1.71907E-06	2.47944E-05	coenzyme F420-dependent glucose-6-phosphate dehydrogenase [EC:1.1.98.2]
K15511	1.22425E-06	3.02567E-06	1.39992E-05	3.4763E-05	benzoyl-CoA 2,3-epoxidase subunit A [EC:1.14.13.208]
K15512	1.25885E-06	3.06069E-06	1.42872E-05	3.47746E-05	benzoyl-CoA 2,3-epoxidase subunit B [EC:1.14.13.208]
K15513	1.28637E-06	3.15165E-06	1.42916E-05	3.48047E-05	benzoyl-CoA-dihydrodiol lyase [EC:4.1.2.44]
K15514	8.94497E-07	2.89082E-06	1.34113E-06	2.20174E-06	3,4-dehydroadipyl-CoA semialdehyde dehydrogenase [EC:1.2.1.77]
K15515	1.02405E-06	4.60577E-07	1.58485E-06	1.63835E-05	sulfoacetaldehyde dehydrogenase [EC:1.2.1.81]
K15518	1.13567E-06	1.09043E-06	4.20309E-06	3.46549E-07	deoxyguanosine kinase [EC:2.7.1.113]
K15519	1.15595E-06	2.51524E-07	3.53775E-06	3.55972E-07	deoxyadenosine/deoxycytidine kinase [EC:2.7.1.76 2.7.1.74]
K15520	1.99839E-06	7.18535E-06	4.27318E-06	3.95414E-06	mycothiol synthase [EC:2.3.1.189]
K15521	2.95259E-06	5.32174E-06	7.60413E-06	3.94256E-06	D-inositol-3-phosphate glycosyltransferase [EC:2.4.1.250]
K15524	4.29429E-08	7.81508E-08	2.88766E-06	2.42551E-06	mannosylglycerate hydrolase [EC:3.2.1.170]
K15525	3.92148E-07	1.68587E-06	4.18142E-06	4.61473E-06	N-acetyl-1-D-myo-inositol-2-amino-2-deoxy-alpha-D-glucopyranoside deacetylase [EC:3.5.1.103]
K15526	1.12139E-06	3.84233E-06	3.55799E-06	2.17353E-06	L-cysteine:1D-myo-inositol 2-amino-2-deoxy-alpha-D-glucopyranoside ligase [EC:6.3.1.13]
K15527	0	0	5.26447E-07	0	cysteate synthase [EC:2.5.1.76]
K15531	8.67992E-08	1.51271E-06	1.68602E-07	0	oligosaccharide reducing-end xylanase [EC:3.2.1.156]
K15532	0.000215416	0.000215153	0.000188952	0.000115421	unsaturated rhamnogalacturonyl hydrolase [EC:3.2.1.172]
K15533	8.73243E-07	6.08661E-07	5.90763E-06	1.22556E-07	1,3-beta-galactosyl-N-acetylhexosamine phosphorylase [EC:2.4.1.211]
K15534	0	0	7.15978E-09	0	beta-D-galactosyl-(1->4)-L-rhamnose phosphorylase [EC:2.4.1.247]
K15536	1.0135E-07	3.18848E-07	4.0579E-07	1.4857E-05	soluble cytochrome b562
K15538	0	0	0	2.46092E-07	glycoprotein endo-alpha-1,2-mannosidase [EC:3.2.1.130]
K15539	0.000217744	0.000216957	0.000207933	0.000217016	cytoskeleton protein RodZ
K15540	3.663E-06	7.70833E-06	2.24658E-05	0.000129824	chaperone protein EcpD
K15545	1.29547E-07	3.81027E-07	1.08996E-06	1.64959E-05	transcriptional regulator of PTS gene
K15546	2.28145E-06	3.06171E-06	1.41006E-05	3.48948E-05	XRE family transcriptional regulator, aerobic/anaerobic benzoate catabolism transcriptional regulator
K15547	1.59618E-07	2.57759E-07	1.29717E-07	7.20952E-07	multidrug resistance protein MdtO
K15548	5.67002E-06	1.74273E-06	5.21834E-06	5.82624E-05	p-hydroxybenzoic acid efflux pump subunit AaeA
K15549	1.26227E-06	4.46302E-07	6.22014E-07	6.95423E-06	membrane fusion protein, multidrug efflux system
K15550	0.000216347	0.000215341	0.000203009	0.000200786	outer membrane protein, multidrug efflux system
K15551	1.41727E-06	8.11729E-07	3.78554E-06	5.30271E-06	taurine transport system substrate-binding protein
K15552	2.4596E-06	3.9096E-06	5.0219E-06	5.40467E-06	taurine transport system permease protein
K15553	0.000443333	0.000440245	0.000449831	0.000395231	sulfonate transport system substrate-binding protein
K15554	0.000221847	0.000222918	0.00022582	0.000201574	sulfonate transport system permease protein
K15555	0.000893218	0.000897105	0.000936604	0.000957613	sulfonate transport system ATP-binding protein [EC:3.6.3.-]
K15576	0.000216499	0.000215193	0.000206334	0.000156804	nitrate/nitrite transport system substrate-binding protein
K15577	0.000216491	0.000215237	0.00020524	0.000155539	nitrate/nitrite transport system permease protein
K15578	0.000446803	0.000447112	0.00047649	0.000428695	nitrate/nitrite transport system ATP-binding protein [EC:3.6.3.-]
K15579	0.000213931	0.000211967	0.000186756	0.000114997	nitrate/nitrite transport system ATP-binding protein
K15580	0.000223701	0.000222698	0.000233442	0.000196256	oligopeptide transport system substrate-binding protein

K15581	6.26014E-06	7.32393E-06	3.72909E-05	5.93919E-05	oligopeptide transport system permease protein
K15582	0.00022127	0.00022207	0.000215264	0.000137262	oligopeptide transport system permease protein
K15583	0.000668938	0.000662213	0.000671097	0.000529166	oligopeptide transport system ATP-binding protein
K15584	1.78787E-06	1.12231E-06	3.85316E-06	3.05559E-06	nickel transport system substrate-binding protein
K15585	1.34648E-06	2.58896E-07	4.2636E-06	1.90719E-06	nickel transport system permease protein
K15586	1.64646E-06	9.25251E-07	8.32203E-06	5.35804E-06	nickel transport system permease protein
K15587	4.90122E-06	1.2153E-06	1.47191E-05	1.32094E-05	nickel transport system ATP-binding protein [EC:3.6.3.24]
K15598	2.95591E-07	4.31871E-07	3.15974E-07	1.43392E-06	putative hydroxymethylpyrimidine transport system substrate-binding protein
K15599	2.08383E-06	3.91765E-06	1.70305E-05	6.69866E-05	putative hydroxymethylpyrimidine transport system permease protein
K15600	6.4192E-06	4.89281E-06	2.21112E-05	5.04629E-05	putative hydroxymethylpyrimidine transport system ATP-binding protein
K15629	0.000214378	0.000212854	0.000188426	0.000113603	fatty-acid peroxygenase [EC:1.11.2.4]
K15632	0	2.78321E-09	3.91048E-08	0	23S rRNA (adenine-C8)-methyltransferase [EC:2.1.1.224]
K15633	4.53932E-06	3.86942E-06	1.26731E-05	1.81304E-05	2,3-bisphosphoglycerate-independent phosphoglycerate mutase [EC:5.4.2.12]
K15634	0.000217512	0.000219667	0.000211794	0.000153026	probable phosphoglycerate mutase [EC:5.4.2.12]
K15635	7.35288E-07	7.3461E-07	2.27614E-06	3.42493E-08	2,3-bisphosphoglycerate-independent phosphoglycerate mutase [EC:5.4.2.12]
K15640	9.45706E-07	1.99349E-07	4.70112E-06	2.34055E-06	uncharacterized phosphatase
K15641	5.41862E-09	1.52891E-07	1.25477E-06	3.67347E-07	myxalamid-type polyketide synthase MxaF
K15642	0.000213773	0.000211951	0.000187208	0.000113628	myxalamid-type polyketide synthase MxaD
K15643	0.000213827	0.000211999	0.000187195	0.000113705	myxalamid-type polyketide synthase MxaB
K15644	0.000213768	0.000211827	0.000188444	0.000113419	coronafacic acid polyketide synthase Cfa6
K15645	8.18459E-10	6.21519E-09	2.14638E-06	1.02871E-07	coronafacic acid polyketide synthase Cfa7
K15646	1.1314E-09	1.44682E-09	9.18063E-08	0	coronamic acid synthetase CmaA, adenylation and thiolation didomain protein [EC:6.2.1.46]
K15650	2.35909E-09	2.95733E-08	3.40238E-07	8.58671E-09	non-haem Fe2+, alpha-ketoglutarate-dependent halogenase
K15652	3.11512E-09	2.75942E-09	6.28585E-08	1.50769E-08	3-dehydroshikimate dehydratase [EC:4.2.1.118]
K15653	0.000430179	0.000428132	0.000391578	0.000277169	nonribosomal peptide synthetase MxcG
K15654	0.000863214	0.000858716	0.000804946	0.000596209	surfactin family lipopeptide synthetase A
K15655	0.000433637	0.000432455	0.000422773	0.000333951	surfactin family lipopeptide synthetase B
K15656	0.000430183	0.000427465	0.000395234	0.000270241	surfactin family lipopeptide synthetase C
K15657	0	0	1.27285E-08	0	external thioesterase TEII
K15658	0.00064525	0.000641786	0.000592157	0.000414429	arthrofactin-type cyclic lipopeptide synthetase A
K15659	0.000863468	0.000857437	0.00080317	0.000582985	arthrofactin-type cyclic lipopeptide synthetase B
K15660	0.001076318	0.001069196	0.00099166	0.000701441	arthrofactin-type cyclic lipopeptide synthetase C
K15661	0.000643662	0.000638109	0.000571723	0.000377425	iturin family lipopeptide synthetase A
K15662	0.001076516	0.001070814	0.000995831	0.000717507	iturin family lipopeptide synthetase B
K15663	0.000859046	0.000855249	0.00078273	0.000546382	iturin family lipopeptide synthetase C
K15664	0.000215396	0.000213793	0.000200296	0.000138876	fengycin family lipopeptide synthetase A
K15665	0.000432229	0.000431635	0.000413067	0.000321522	fengycin family lipopeptide synthetase B
K15666	0.000432324	0.000428997	0.000401727	0.000299474	fengycin family lipopeptide synthetase C
K15667	0.000862617	0.000857997	0.000804615	0.000603605	fengycin family lipopeptide synthetase D
K15668	0.000214768	0.000213937	0.000197166	0.000133514	fengycin family lipopeptide synthetase E
K15669	6.08189E-08	4.78843E-08	5.11131E-07	2.8605E-06	D-glycero-alpha-D-manno-heptose 1-phosphate guanylyltransferase [EC:2.7.7.71]
K15670	1.94986E-09	1.52329E-07	2.84557E-06	2.17153E-07	rifamycin polyketide synthase modules 1, 2 and 3
K15671	1.44253E-08	1.44649E-07	2.39334E-06	2.05467E-07	rifamycin polyketide synthase modules 4, 5 and 6
K15672	0	1.06439E-08	1.74121E-06	5.15203E-08	rifamycin polyketide synthase modules 7 and 8
K15673	1.35106E-08	1.33839E-07	1.93496E-06	1.59514E-07	rifamycin polyketide synthase modules 9 and 10
K15674	0.000213766	0.000211789	0.000186419	0.000113232	rhizoxin biosynthesis, polyketide synthase / nonribosomal peptide synthetase RhiA
K15675	0.000215081	0.000214201	0.000198735	0.000140305	rhizoxin biosynthesis, polyketide synthase / nonribosomal peptide synthetase RhiB
K15676	3.64735E-08	4.75056E-08	1.63466E-06	2.92867E-07	rhizoxin biosynthesis, polyketide synthase RhiC
K15677	3.82976E-07	6.68532E-07	3.685E-06	6.98447E-06	rhizoxin biosynthesis, polyketide synthase RhiD
K15678	6.96195E-08	1.5124E-07	1.76909E-06	2.78494E-07	rhizoxin biosynthesis, polyketide synthase RhiE
K15679	6.94993E-08	4.65261E-08	1.09034E-06	9.20254E-08	rhizoxin biosynthesis, polyketide synthase RhiF
K15681	0	0	1.04657E-08	1.60743E-08	aminotransferase MxcL
K15721	0.00021391	0.000212201	0.000186749	0.000118045	pesticin/yersiniabactin receptor
K15722	2.83474E-08	1.95055E-08	5.49186E-08	7.26295E-07	cell division activator
K15723	9.82118E-08	3.14581E-07	3.2627E-07	1.71382E-05	SecY interacting protein Syd
K15724	0.000219984	0.000217424	0.000213956	0.000219676	iron-sulfur cluster insertion protein
K15725	0.000437864	0.000437465	0.000434457	0.000484603	outer membrane protein, cobalt-zinc-cadmium efflux system
K15726	0.000663937	0.000658222	0.000659569	0.000704254	cobalt-zinc-cadmium resistance protein CzcA
K15727	0.001086541	0.001079101	0.001016174	0.000895535	membrane fusion protein, cobalt-zinc-cadmium efflux system
K15729	0	0	0	8.03716E-09	microsomal prostaglandin-E synthase 1 [EC:5.3.99.3]
K15731	1.74417E-07	1.02308E-07	2.60317E-07	6.54076E-06	carboxy-terminal domain RNA polymerase II polypeptide A small phosphatase [EC:3.1.3.16]
K15733	1.01817E-06	3.63154E-06	1.98402E-06	1.9841E-06	dye decolorizing peroxidase [EC:1.11.1.19]
K15734	8.65702E-07	3.10056E-06	1.71064E-06	2.07108E-08	all-trans-retinol dehydrogenase (NAD+) [EC:1.1.1.105]
K15735	9.16735E-07	2.80503E-06	6.17764E-07	6.62063E-07	GntR family transcriptional regulator, carbon starvation induced regulator
K15736	1.77639E-06	3.91048E-06	1.86939E-05	4.11955E-05	L-2-hydroxyglutarate oxidase [EC:1.1.3.-]
K15737	2.83214E-08	1.99683E-08	5.0338E-08	1.32967E-06	protein CsiD
K15738	0.000901158	0.000909548	0.000979471	0.001028847	ABC transport system ATP-binding/permease protein
K15739	0	1.63753E-08	8.64189E-07	6.6024E-08	D-alanine---(R)-lactate ligase [EC:6.1.2.1]
K15746	4.36923E-07	1.24765E-06	7.54363E-07	1.27528E-07	beta-carotene 3-hydroxylase [EC:1.14.15.24]
K15750	7.94338E-09	1.77569E-10	1.47507E-07	8.14E-10	biphenyl 2,3-dioxygenase subunit beta [EC:1.14.12.18]
K15751	0	1.17924E-09	1.08825E-08	1.13059E-06	carbazole 1,9a-dioxygenase [EC:1.14.12.22]
K15752	0	1.38505E-08	0	0	carbazole 1,9a-dioxygenase ferredoxin component
K15756	4.04415E-10	1.6964E-09	3.09336E-08	3.28568E-09	2-hydroxy-6-oxo-6-(2-aminophenyl)hexa-2,4-dienoate hydrolase [EC:3.7.1.13]
K15760	5.53664E-10	1.45586E-08	2.79086E-08	5.90766E-09	toluene monooxygenase system protein A [EC:1.14.13.236 1.14.13.-]
K15761	5.53664E-10	1.45586E-08	2.09314E-08	5.35811E-09	toluene monooxygenase system protein B [EC:1.14.13.236 1.14.13.-]
K15762	2.20854E-09	1.45586E-08	2.09314E-08	3.86697E-08	toluene monooxygenase system ferredoxin subunit
K15763	5.53664E-10	1.45586E-08	2.79086E-08	5.35811E-09	toluene monooxygenase system protein D [EC:1.14.13.236 1.14.13.-]
K15764	5.53664E-10	1.45586E-08	2.79086E-08	5.90766E-09	toluene monooxygenase system protein E [EC:1.14.13.236 1.14.13.-]
K15765	5.41724E-07	6.00526E-07	3.02005E-06	6.95069E-06	toluene monooxygenase electron transfer component [EC:1.18.1.3]
K15770	2.53167E-06	4.36748E-06	8.74174E-06	1.15969E-05	arabinogalactan oligomer / maltooligosaccharide transport system substrate-binding protein
K15771	2.79001E-06	5.49993E-06	1.19754E-05	1.52493E-05	arabinogalactan oligomer / maltooligosaccharide transport system permease protein
K15772	3.3994E-06	4.7265E-06	1.21598E-05	1.21013E-05	arabinogalactan oligomer / maltooligosaccharide transport system permease protein
K15773	0.000430249	0.000428971	0.000394797	0.000297076	HTH-type transcriptional regulator / antitoxin HipB
K15777	0.000219522	0.00021706	0.000209423	0.000212305	4,5-DOPA dioxygenase extradiol [EC:1.13.11.-]
K15778	0.000435932	0.000430642	0.000398907	0.000406592	phosphomannomutase / phosphoglucomutase [EC:5.4.2.8 5.4.2.2]
K15780	1.04582E-08	0	1.6125E-08	4.71826E-07	bifunctional protein TilS/HprT [EC:6.3.4.19 2.4.2.8]
K15781	0	9.71283E-07	1.90754E-06	3.97111E-06	putative phosphoserine phosphatase / 1-acylglycerol-3-phosphate O-acyltransferase [EC:3.1.3.3 2.3.1.51]

K15782	0.000213813	0.000212653	0.000189188	0.000115089	Lrp/AsnC family transcriptional regulator, regulator of ectoine-degradation genes
K15783	1.37044E-07	1.29645E-07	1.1815E-06	2.95335E-07	ectoine hydrolase [EC:3.5.4.44]
K15784	2.02031E-08	1.88809E-07	9.52429E-07	2.3897E-07	N2-acetyl-L-2,4-diaminobutanoate deacetylase [EC:3.5.1.125]
K15785	1.95583E-06	2.01906E-06	1.31363E-05	2.38765E-05	L-2,4-diaminobutyrate transaminase [EC:2.6.1.76]
K15786	0.000217426	0.000220556	0.000213847	0.000180618	aspartate-semialdehyde dehydrogenase [EC:1.2.1.-]
K15790	5.61956E-08	1.22733E-07	2.58815E-07	1.2313E-07	nitrogen fixation protein NifQ
K15792	0.000215997	0.000215346	0.00020607	0.000148671	murE/murF fusion protein [EC:6.3.2.13 6.3.2.10]
K15827	4.06566E-08	7.45111E-08	2.13566E-07	2.4276E-06	formate hydrogenlyase subunit 2
K15828	2.83668E-08	3.05583E-08	2.08001E-07	2.12136E-06	formate hydrogenlyase subunit 3
K15829	3.22117E-08	3.73905E-08	2.49691E-07	2.30563E-06	formate hydrogenlyase subunit 4
K15830	3.22279E-08	3.74308E-08	2.49808E-07	2.30611E-06	formate hydrogenlyase subunit 5
K15831	2.83474E-08	2.45039E-08	5.49186E-08	7.5193E-07	formate hydrogenlyase subunit 6
K15832	3.6849E-08	3.80501E-08	2.49551E-07	2.30506E-06	formate hydrogenlyase subunit 7
K15833	2.83474E-08	1.95055E-08	5.49186E-08	7.45288E-07	formate hydrogenlyase regulatory protein HycA
K15834	2.8396E-08	2.46249E-08	1.89271E-07	2.11204E-06	formate hydrogenlyase maturation protein HycH
K15835	1.15097E-06	1.52598E-06	1.95412E-06	6.244E-05	RpiR family transcriptional regulator, murPQ operon repressor
K15836	0.000650681	0.000646037	0.000608471	0.000486123	formate hydrogenlyase transcriptional activator
K15843	1.44434E-10	2.05911E-09	0	0	outer membrane protein HopC/AlpA
K15850	1.21049E-06	8.16683E-07	6.69733E-06	9.47779E-06	two-component system, autoinducer 1 sensor kinase/phosphatase LuxN [EC:2.7.13.3 3.1.3.-]
K15852	7.16412E-08	1.26513E-07	9.14992E-07	1.4101E-05	LuxR family transcriptional regulator, transcriptional activator of the bioluminescence operon
K15853	3.1635E-09	0	9.89572E-09	1.58716E-07	acyl transferase [EC:2.3.1.-]
K15854	1.14023E-07	6.99898E-08	2.57101E-07	9.5052E-06	alkanal monooxygenase beta chain [EC:1.14.14.3]
K15855	2.44485E-08	1.06439E-08	7.40571E-07	1.53089E-09	exo-1,4-beta-D-glucosaminidase [EC:3.2.1.165]
K15856	2.16001E-06	4.49914E-07	1.48763E-06	2.11355E-06	GDP-4-dehydro-6-deoxy-D-mannose reductase [EC:1.1.1.281]
K15861	2.31653E-06	9.06349E-07	5.79166E-07	9.51609E-06	CRP/FNR family transcriptional regulator, nitrogen fixation regulation protein
K15862	0.000217279	0.000217777	0.000207716	0.000172165	cytochrome c oxidase cbb3-type subunit I/II [EC:1.9.3.1]
K15864	4.46815E-09	9.43088E-08	1.44324E-07	5.30455E-08	nitrite reductase (NO-forming) / hydroxylamine reductase [EC:1.7.2.1 1.7.99.1]
K15865	8.75145E-07	2.3326E-08	8.41072E-06	6.88707E-08	threonylcarbamoyladenosine tRNA methylthiotransferase CDKAL1 [EC:2.8.4.5]
K15866	0.001313252	0.001323429	0.00132698	0.001236142	2-(1,2-epoxy-1,2-dihydrophenyl)acetyl-CoA isomerase [EC:5.3.3.18]
K15868	1.52845E-06	3.36615E-06	1.46098E-05	3.58854E-05	bile acid-coenzyme A ligase [EC:6.2.1.7]
K15876	7.40182E-08	3.18986E-07	4.37132E-07	4.41843E-08	cytochrome c nitrite reductase small subunit
K15878	3.97169E-09	9.82517E-09	3.13971E-08	2.28209E-08	rieske iron-sulfur protein
K15879	0	0	3.13971E-08	1.70245E-08	cytochrome b-561
K15884	0	0	2.0226E-08	0	bifunctional aromatase (cyclase/dehydratase) [EC:4.2.1.-]
K15885	0	0	1.44647E-08	0	C7-C12 aromatase (ARO/CYC) [EC:4.2.1.-]
K15888	1.38485E-06	3.59617E-06	3.88546E-06	1.93634E-06	tritrans,polycis-undecaprenyl-diphosphate synthase [geranylgeranyl-diphosphate specific] [EC:2.5.1.89]
K15894	3.47108E-06	2.19097E-06	5.7377E-06	6.31227E-05	UDP-N-acetylglucosamine 4,6-dehydratase [EC:4.2.1.115]
K15895	0.000215575	0.000212101	0.00018867	0.000116145	UDP-4-amino-4,6-dideoxy-L-N-acetyl-beta-L-altrosamine transaminase [EC:2.6.1.92]
K15896	0	0	0	6.58671E-08	UDP-4-amino-4,6-dideoxy-N-acetyl-beta-L-altrosamine N-acetyltransferase [EC:2.3.1.202]
K15897	0	1.85547E-09	0	0	UDP-2,4-diacetamido-2,4,6-trideoxy-beta-L-altropyranose hydrolase [EC:3.6.1.57]
K15898	1.37103E-06	2.71398E-07	2.30456E-06	6.49959E-06	pseudaminic acid synthase [EC:2.5.1.97]
K15899	1.24319E-06	7.65648E-08	1.33958E-06	7.40515E-07	pseudaminic acid cytidylyltransferase [EC:2.7.7.81]
K15904	2.23408E-08	0	5.16189E-09	8.55783E-09	bifunctional N6-L-threonylcarbamoyladenine synthase / protein kinase Bud32 [EC:2.3.1.234 2.7.11.1]
K15907	9.76649E-10	1.53917E-10	1.69804E-07	3.17969E-09	pentalenene oxygenase [EC:1.14.13.133]
K15910	3.82092E-07	1.43701E-06	1.9254E-06	2.68018E-06	UDP-N-acetylbacillosamine transaminase [EC:2.6.1.34]
K15912	0.000216964	0.000215648	0.000193776	0.000175018	UDP-N-acetyl-D-glucosamine 4,6-dehydratase [EC:4.2.1.135]
K15913	2.08492E-07	4.98406E-08	2.45536E-07	5.34911E-07	UDP-N-acetylbacillosamine N-acetyltransferase [EC:2.3.1.203]
K15914	3.23946E-08	2.6458E-08	1.40901E-07	2.55719E-08	N,N-diacetylbacillosaminyl-diphospho-undecaprenol alpha-1,3-N-acetylgalactosaminyltransferase [EC:2.4.1.290]
K15915	1.99104E-06	2.16428E-06	2.50922E-06	5.70753E-06	undecaprenyl phosphate N,N-diacetylbacillosamine 1-phosphate transferase [EC:2.7.8.36]
K15916	0	0	9.56518E-08	0	glucose/mannose-6-phosphate isomerase [EC:5.3.1.9 5.3.1.8]
K15918	1.37792E-06	1.26878E-06	1.98647E-06	5.47528E-05	D-glycerate 3-kinase [EC:2.7.1.31]
K15921	5.31886E-07	1.29482E-06	2.6162E-06	5.40464E-08	arabinoxylan arabinofuranohydrolase [EC:3.2.1.55]
K15922	7.75312E-08	8.10752E-08	2.21348E-07	2.46801E-07	sulfoquinovosidase [EC:3.2.1.199]
K15923	2.57651E-06	4.7898E-06	1.26388E-05	3.15402E-06	alpha-L-fucosidase 2 [EC:3.2.1.51]
K15924	2.997E-09	1.48438E-08	6.31506E-07	2.64433E-08	glucuronoarabinoxylan endo-1,4-beta-xylanase [EC:3.2.1.136]
K15926	0	0	9.84151E-08	1.78604E-09	cyclase
K15927	2.76562E-08	8.81714E-08	6.69171E-07	3.59965E-08	oxygenase
K15928	8.87738E-07	1.36182E-06	6.63977E-06	3.41942E-05	bifunctional hydroxylase/dehydrase
K15930	0.000213765	0.000211784	0.000186513	0.000113231	bifunctional oxygenase/reductase
K15935	0	0	9.75427E-08	0	L-rhodinosyltransferase
K15941	0	0	1.81612E-08	0	deoxynogalonate / 12-deoxyaklanonic acid monooxygenase [EC:1.13.12.22 1.13.12.-]
K15942	0	0	1.81612E-08	0	O-methyltransferase / aklanonic acid methyltransferase [EC:2.1.1.- 2.1.1.288]
K15943	0	0	1.81612E-08	0	nogalonic acid methyl ester cyclase / aklanonic acid methyl ester cyclase [EC:5.5.1.26 5.5.1.23]
K15944	0	1.1704E-07	3.0454E-07	1.60463E-08	nogalaviketone/aklaviketone reductase [EC:1.1.1.- 1.1.1.362]
K15950	0	0	4.35362E-08	0	aklavinone 12-hydroxylase [EC:1.14.13.180]
K15953	8.10272E-11	3.94445E-09	3.27726E-07	7.75831E-09	10-carbomethoxy-13-deoxycarminomycin esterase
K15955	0	0	2.22133E-08	0	13-deoxydaunorubicin hydroxylase [EC:1.14.13.181]
K15971	0	0	5.76604E-08	1.0991E-09	O-methyltransferase [EC:2.1.1.-]
K15972	6.6526E-08	2.01332E-07	7.2455E-07	6.53187E-08	tetracenomycin A2 monooxygenase-dioxygenase [EC:1.14.13.200]
K15973	1.32633E-06	2.54805E-07	3.87185E-06	4.25744E-06	MarR family transcriptional regulator, 2-MHQ and catechol-resistance regulon repressor
K15974	2.76313E-07	5.10942E-07	4.19558E-07	9.51173E-07	MarR family transcriptional regulator, negative regulator of the multidrug operon emrRAB
K15975	5.4126E-06	2.8293E-06	1.91947E-05	3.22646E-05	glyoxalase family protein
K15976	6.05228E-08	6.53588E-07	4.41353E-07	5.21723E-08	putative NAD(P)H nitroreductase [EC:1.-.-.-]
K15977	0.000655853	0.000654919	0.000631595	0.000808225	putative oxidoreductase
K15980	0	0	1.04657E-08	0	acyl-CoA dehydrogenase family member 9 [EC:1.3.99.-]
K15981	1.67605E-07	1.92755E-06	2.41759E-06	8.48556E-06	cholest-4-en-3-one 26-monooxygenase [EC:1.14.15.29]
K15982	5.84207E-08	8.23915E-07	7.77617E-07	7.7347E-06	3-ketosteroid 9alpha-monooxygenase subunit A [EC:1.14.15.30]
K15983	0.000214733	0.000213178	0.000189095	0.000168266	3-ketosteroid 9alpha-monooxygenase subunit B [EC:1.14.15.30]
K15984	1.43271E-06	8.41179E-07	2.22455E-06	1.78278E-05	16S rRNA (guanine1516-N2)-methyltransferase [EC:2.1.1.242]
K15986	3.32317E-06	1.19018E-06	1.11602E-05	1.75146E-05	manganese-dependent inorganic pyrophosphatase [EC:3.6.1.1]
K15987	5.64515E-06	6.95297E-06	3.22739E-05	8.70566E-05	K(+)-stimulated pyrophosphate-energized sodium pump [EC:3.6.1.1]
K15988	0	0	4.95913E-07	3.57207E-09	tylactone synthase
K15996	0	0	2.56357E-08	0	macrocin O-methyltransferase [EC:2.1.1.101]
K16000	0	0	1.05326E-07	0	narbonolide/10-deoxymethynolide synthase [EC:2.3.1.240 2.3.1.239]
K16001	1.71257E-09	0	8.80323E-07	1.0991E-08	narbonolide/10-deoxymethynolide synthase [EC:2.3.1.240 2.3.1.239]
K16007	2.64796E-10	3.38617E-10	8.34103E-07	1.20901E-08	8,8a-deoxyoleandolide synthase
K16011	7.3067E-06	1.06122E-05	2.82157E-05	8.65881E-05	mannose-1-phosphate guanylyltransferase / mannose-6-phosphate isomerase [EC:2.7.7.13 5.3.1.8]

K16012	2.03524E-05	2.16772E-05	7.2935E-05	0.000354436	ATP-binding cassette, subfamily C, bacterial CydC
K16013	0.00023433	0.000239283	0.000292786	0.000401946	ATP-binding cassette, subfamily C, bacterial CydD
K16014	0.00022393	0.000227134	0.000237759	0.000295869	ATP-binding cassette, subfamily C, bacterial CydCD
K16015	0	0	1.27285E-08	0	oxidoreductase [EC:1.1.1.-]
K16016	9.39085E-07	2.82683E-06	7.06771E-07	1.49715E-07	3-amino-5-hydroxybenzoate synthase [EC:4.2.1.144 2.6.1.-]
K16017	8.74126E-09	3.33228E-09	1.62135E-07	0	AHBA synthesis associated protein
K16018	0	1.31002E-09	2.5457E-08	4.27891E-09	kanosamine 6-kinase [EC:2.7.1.179]
K16019	0	0	6.97714E-09	0	3,4-Dideoxy-4-amino-D-arabino-heptulosonate 7-phosphate synthase
K16020	0	4.62635E-07	2.6827E-07	1.6745E-07	5-deoxy-5-amino-3-dehydroquinate synthase
K16021	0	0	1.48499E-08	0	5-deoxy-5-amino-3-dehydroquinate dehydratase
K16022	6.0969E-08	6.27614E-08	6.00062E-07	3.92806E-07	flavoprotein hydroxylase
K16024	0	0	4.70247E-08	9.15344E-09	methoxymalonate biosynthesis protein
K16025	0	0	4.70247E-08	0	methoxymalonate biosynthesis acyl carrier protein
K16026	1.07844E-09	1.14677E-07	2.58504E-07	1.28283E-08	methoxymalonate biosynthesis protein
K16027	0	2.99905E-08	1.26987E-07	3.05637E-10	methoxymalonate biosynthesis protein
K16028	2.59239E-08	1.14273E-06	1.04948E-06	1.58114E-07	O-methyltransferase
K16029	3.24007E-09	7.95682E-09	1.19847E-07	6.46706E-10	ansamitocin polyketide synthase A
K16030	0	0	2.02568E-07	3.57207E-09	ansamitocin polyketide synthase B
K16032	0	0	4.98163E-07	0	ansamitocin polyketide synthase D
K16033	0	0	1.27285E-08	0	FADH2-dependent halogenase
K16035	8.80359E-10	3.07834E-11	0	0	7-O-carbamoyltransferase [EC:2.1.3.-]
K16037	2.91332E-07	2.92941E-07	2.46066E-06	3.59197E-06	4,5-epoxidase
K16039	0	0	7.03889E-08	8.03716E-09	N-glycosyltransferase [EC:2.4.1.-]
K16041	1.4196E-09	3.53084E-09	1.02231E-08	4.20516E-08	non-LEE-encoded effector NleA
K16042	1.86038E-09	4.62717E-09	1.33974E-08	5.51087E-08	non-LEE-encoded effector NleH
K16043	0	0	7.16653E-08	3.78957E-11	scyllo-inositol 2-dehydrogenase (NAD+) [EC:1.1.1.370]
K16044	0.000217408	0.000216798	0.000190416	0.000180444	scyllo-inositol 2-dehydrogenase (NADP+) [EC:1.1.1.371]
K16045	0	1.50609E-07	4.56913E-08	3.03552E-08	3beta-hydroxy-Delta5-steroid dehydrogenase / steroid Delta-isomerase [EC:1.1.1.145 5.3.3.1]
K16046	3.0053E-08	2.84703E-07	3.40362E-07	2.99034E-06	cholest-4-en-3-one 26-monooxygenase [EC:1.14.15.28]
K16047	1.27557E-07	7.58302E-07	3.05081E-06	1.02474E-05	3-hydroxy-9,10-secoandrosta-1,3,5(10)-triene-9,17-dione monooxygenase [EC:1.14.14.12]
K16048	0.000217598	0.000220003	0.000222855	0.000222036	3-hydroxy-9,10-secoandrosta-1,3,5(10)-triene-9,17-dione monooxygenase reductase component [EC:1.5.1.-]
K16049	2.2379E-08	5.51143E-07	4.31598E-07	6.11888E-06	3,4-dihydroxy-9,10-secoandrosta-1,3,5(10)-triene-9,17-dione 4,5-dioxygenase [EC:1.13.11.25]
K16050	8.3193E-08	1.17604E-06	2.10382E-06	8.41779E-06	4,5:9,10-diseco-3-hydroxy-5,9,17-trioxoandrosta-1(10),2-diene-4-oate hydrolase [EC:3.7.1.17]
K16051	4.4905E-08	6.13092E-07	1.99491E-06	9.02331E-06	3-oxo-5alpha-steroid 4-dehydrogenase [EC:1.3.99.5]
K16052	1.91958E-06	7.55842E-07	4.83716E-06	5.47643E-06	MscS family membrane protein
K16053	3.20644E-06	7.35022E-06	2.15891E-05	0.000113105	miniconductance mechanosensitive channel
K16055	0.000215268	0.000215464	0.00019256	0.000180961	trehalose 6-phosphate synthase/phosphatase [EC:2.4.1.15 3.1.3.12]
K16066	0.000432222	0.000428883	0.00038829	0.000299042	3-hydroxy acid dehydrogenase / malonic semialdehyde reductase [EC:1.1.1.381 1.1.1.-]
K16074	1.82564E-06	4.202E-06	1.57462E-05	5.12426E-05	zinc transporter
K16076	4.11405E-07	1.27521E-06	5.39044E-07	5.41251E-06	outer membrane porin protein LC
K16077	1.81066E-07	6.63383E-07	3.40177E-07	1.19129E-05	sucrose porin
K16078	7.73441E-12	1.92371E-11	5.56987E-11	3.40121E-08	attachment invasion locus protein
K16079	1.70077E-05	2.48176E-06	4.80196E-06	1.28295E-05	outer membrane immunogenic protein
K16080	0.000215831	0.000211871	0.000187966	0.000113319	high affinity Mn2+ porin
K16081	2.04795E-08	3.19547E-09	1.27031E-06	1.84069E-07	alginate production protein
K16087	9.8484E-06	1.03153E-05	3.95327E-05	0.000330217	hemoglobin/transferrin/lactoferrin receptor protein
K16088	2.54086E-05	5.30463E-05	0.000246168	0.000838222	outer-membrane receptor for ferric coprogen and ferric-rhodotorulic acid
K16089	9.89905E-06	2.02669E-05	5.98431E-05	0.000358327	outer membrane receptor for ferrienterochelin and colicins
K16090	0.000222323	0.000227409	0.000259158	0.000334646	catecholate siderophore receptor
K16091	3.46469E-06	7.54301E-06	3.01398E-05	0.000129995	Fe(3+) dicitrate transport protein
K16092	0.000221783	0.000222011	0.000229997	0.000357723	vitamin B12 transporter
K16093	0.000217859	0.000218967	0.000226779	0.000199864	bacitracin synthase 1
K16094	0.000428858	0.000424861	0.000381986	0.000241058	bacitracin synthase 2
K16095	0.000430766	0.000431151	0.000408258	0.000322767	bacitracin synthase 3
K16097	1.02505E-08	3.33985E-08	1.45667E-07	7.65444E-09	gramicidin S synthase 2
K16098	3.24676E-08	1.41805E-08	3.40484E-07	5.82597E-08	nonribosomal peptide synthetase protein VioF
K16099	1.03738E-06	1.39212E-06	9.95938E-06	2.26194E-05	nonribosomal peptide synthetase protein VioA
K16100	0.000213768	0.000211776	0.000186482	0.000113229	nonribosomal peptide synthetase protein VioI
K16101	1.22135E-06	1.59154E-07	4.00967E-07	2.31955E-07	nonribosomal peptide synthetase protein VioG
K16102	1.20362E-10	1.24224E-07	1.12879E-07	1.87152E-06	nonribosomal peptide synthetase protein VioO
K16104	0	0	4.85093E-08	0	2,3-diaminopropionyl alpha,beta-desaturase
K16118	0.000643493	0.000639275	0.000579093	0.000392813	pristinamycin I synthase 3 and 4
K16119	0.000214916	0.000214171	0.000197396	0.000140045	lichenysin synthetase A
K16120	1.80164E-06	3.57594E-07	1.45085E-06	2.89283E-06	lichenysin synthetase B
K16121	1.19435E-07	2.5992E-07	1.11774E-06	9.62077E-07	lichenysin synthetase C
K16122	1.34517E-06	1.23026E-06	8.76447E-06	1.46852E-05	tyrocidine synthetase I
K16123	0.000643905	0.000639308	0.000583864	0.000386306	tyrocidine synthetase II
K16124	0.000860568	0.00085566	0.000786086	0.000545205	tyrocidine synthetase III
K16125	0.000646481	0.000642758	0.000598171	0.000435072	syringomycin synthetase protein SyrE
K16126	0.000642303	0.000636047	0.000562912	0.000390473	Syringomycin synthetase protein SyrB1
K16127	6.47035E-09	2.71593E-08	2.88533E-08	4.53395E-10	microcystin synthetase protein McyG
K16128	0.000215046	0.000215158	0.000201719	0.000148565	microcystin synthetase protein McyD
K16129	0.000214381	0.000213314	0.000193069	0.000127589	microcystin synthetase protein McyE
K16130	0.000647337	0.000642585	0.000600593	0.000430449	microcystin synthetase protein McyA
K16131	3.57203E-07	7.92345E-07	3.70898E-06	9.55235E-06	microcystin synthetase protein McyB
K16135	0.002180083	0.002186726	0.002159783	0.002615781	LysR family transcriptional regulator, transcriptional activator for dmlA
K16136	1.40385E-07	3.2274E-07	4.73276E-07	2.91695E-06	LacI family transcriptional regulator, maltose regulon regulatory protein
K16137	0.000664728	0.000667777	0.000671639	0.000762764	TetR/AcrR family transcriptional regulator, transcriptional repressor for nem operon
K16138	4.45782E-09	3.67925E-08	1.03329E-07	5.63796E-07	TetR/AcrR family transcriptional regulator, repressor for uid operon
K16139	6.07138E-08	2.8115E-08	2.04088E-07	7.38928E-07	glucuronide carrier protein
K16140	4.85664E-09	2.37711E-08	4.70899E-08	2.30932E-07	putative glucuronide porin
K16146	0.000215435	0.00021357	0.000194099	0.00011547	maltokinase [EC:2.7.1.175]
K16147	0.00021695	0.000216762	0.000201595	0.000117539	starch synthase (maltosyl-transferring) [EC:2.4.99.16]
K16148	1.8417E-06	6.24372E-06	1.12562E-05	5.80079E-06	alpha-maltose-1-phosphate synthase [EC:2.4.1.342]
K16149	6.52654E-08	1.89754E-07	4.6012E-06	1.75349E-06	1,4-alpha-glucan branching enzyme [EC:2.4.1.18]
K16150	3.39006E-07	3.15624E-07	1.74302E-06	1.8132E-06	glycogen synthase [EC:2.4.1.11]
K16152	6.77593E-08	2.79469E-07	2.18608E-07	6.13606E-08	heme acquisition protein HasR

K16153	4.86765E-07	1.54981E-06	3.15496E-06	4.10202E-08	glycogen phosphorylase/synthase [EC:2.4.1.1 2.4.1.11]
K16157	3.97169E-09	5.32197E-08	0	0	methane monooxygenase component A alpha chain [EC:1.14.13.25]
K16158	0	3.19318E-08	0	0	methane monooxygenase component A beta chain [EC:1.14.13.25]
K16159	0	1.06439E-08	0	0	methane monooxygenase component A gamma chain [EC:1.14.13.25]
K16160	0	1.06439E-08	0	0	methane monooxygenase regulatory protein B
K16161	6.98097E-10	1.15367E-08	0	0	methane monooxygenase component C [EC:1.14.13.25]
K16163	6.13418E-07	3.6226E-07	6.95027E-06	9.73367E-06	maleylpyruvate isomerase [EC:5.2.1.4]
K16164	0.000217702	0.000221976	0.000210946	0.000153652	acylpyruvate hydrolase [EC:3.7.1.5]
K16165	0.000654033	0.000653976	0.000619044	0.00050146	fumarylpyruvate hydrolase [EC:3.7.1.20]
K16167	1.80415E-07	7.52606E-07	1.02682E-06	2.13867E-06	alkylresorcinol/alkylpyrone synthase
K16168	6.58953E-08	7.28111E-07	4.27503E-07	1.08408E-07	methyltransferase
K16169	0.000217621	0.000215918	0.000209649	0.000153669	xanthine permease
K16170	5.54378E-08	0	6.88952E-08	0	uric acid permease
K16171	0.000216655	0.000216674	0.000206979	0.000213221	fumarylacetoacetate (FAA) hydrolase [EC:3.7.1.2]
K16173	6.38602E-07	1.48381E-06	1.3931E-06	1.12046E-05	glutaryl-CoA dehydrogenase (non-decarboxylating) [EC:1.3.99.32]
K16176	0	0	2.09314E-08	0	methylamine---corrinoid protein Co-methyltransferase [EC:2.1.1.248]
K16177	0	1.63753E-08	0	0	monomethylamine corrinoid protein
K16179	1.79571E-07	4.60463E-07	3.80385E-07	1.57939E-08	dimethylamine corrinoid protein
K16188	5.54378E-08	2.06813E-07	7.10924E-07	1.90174E-09	tetraprenyl-beta-curcumene synthase [EC:4.2.3.130]
K16190	9.16819E-09	1.66614E-09	0	0	glucuronokinase [EC:2.7.1.43]
K16191	4.05742E-08	3.47665E-07	2.98639E-07	8.53578E-07	peptidoglycan-binding protein ArfA
K16192	0	0	0	5.4955E-10	uncharacterized membrane protein ArfB
K16193	0	3.1768E-07	0	0	uncharacterized membrane protein ArfC
K16199	3.34743E-07	3.64817E-07	7.09336E-07	5.74633E-07	dipeptide transport system substrate-binding protein
K16200	2.13745E-07	4.35369E-08	5.9913E-07	1.96818E-08	dipeptide transport system permease protein
K16201	4.28318E-07	2.84822E-07	9.01457E-07	1.97434E-06	dipeptide transport system permease protein
K16202	4.22104E-06	3.63262E-06	2.90491E-06	7.21539E-06	dipeptide transport system ATP-binding protein
K16203	0.00021389	0.00021225	0.000189336	0.000115589	D-amino peptidase [EC:3.4.11.-]
K16209	7.99038E-07	3.34136E-07	1.1812E-06	8.98041E-07	lactose/raffinose/galactose permease
K16210	4.80566E-08	5.27569E-08	1.72018E-07	1.84252E-06	oligogalacturonide transporter
K16211	1.84488E-06	1.96951E-06	5.87958E-06	5.64908E-05	maltose/moltooligosaccharide transporter
K16212	1.10178E-07	1.28702E-06	3.12797E-07	1.40237E-06	4-O-beta-D-mannosyl-D-glucose phosphorylase [EC:2.4.1.281]
K16213	1.10178E-07	9.14132E-07	3.0794E-07	1.40491E-06	cellobiose epimerase [EC:5.1.3.11]
K16214	0.000213842	0.000211874	0.0001865	0.000115256	UDP-N-acetylglucosamine kinase [EC:2.7.1.176]
K16215	0	1.49953E-08	6.74258E-07	3.96649E-08	2-ketoarginine methyltransferase [EC:2.1.1.243]
K16216	0.000215248	0.000214819	0.000201134	0.000148087	benzil reductase ((S)-benzoin forming) [EC:1.1.1.320]
K16217	0	0	1.4747E-07	0	geranyl diphosphate 2-C-methyltransferase [EC:2.1.1.255]
K16218	0	0	1.4747E-07	0	2-methylisoborneol synthase [EC:4.2.3.118]
K16227	0	1.06439E-08	1.6858E-08	4.78108E-08	glycopeptidolipid biosynthesis protein
K16228	6.46031E-06	1.34982E-05	4.90451E-05	0.000113368	glycopeptidolipid biosynthesis protein
K16229	0.000858742	0.000854041	0.00077845	0.000538074	glycopeptidolipid biosynthesis protein
K16232	0	0	1.44647E-08	1.78604E-09	germicidin synthase
K16233	0	0	3.05555E-08	0	alpha-pyrone synthase
K16234	0.0002228	0.000229793	0.000260883	0.000258221	histidine transporter
K16235	0.000222476	0.000227865	0.000237374	0.000212305	S-methylmethionine transporter
K16236	3.56538E-08	3.85016E-08	1.89049E-07	1.58269E-07	histidine permease
K16237	4.48977E-06	8.3171E-06	3.94072E-05	0.000126183	aromatic amino acid permease
K16238	1.40714E-06	7.44403E-07	4.51716E-06	7.45084E-06	ethanolamine permease
K16239	0	9.5208E-10	2.9902E-09	0	4-hydroxybenzoate decarboxylase subunit C [EC:4.1.1.61]
K16242	6.91621E-08	2.18755E-07	4.44509E-07	7.57852E-08	phenol hydroxylase P3 protein [EC:1.14.13.-]
K16243	6.92269E-08	2.18916E-07	4.44976E-07	7.77054E-08	Phenol hydroxylase P1 protein
K16244	6.91621E-08	2.18755E-07	4.44509E-07	7.57852E-08	phenol hydroxylase P2 protein
K16245	6.91621E-08	2.18755E-07	4.44509E-07	7.57852E-08	phenol hydroxylase P4 protein
K16246	5.15137E-07	5.41152E-07	1.34352E-06	3.28328E-05	phenol hydroxylase P5 protein
K16247	0	0	1.10674E-07	0	LuxR family transcriptional regulator, glucitol operon activator
K16248	2.23408E-08	7.20795E-08	1.63305E-08	8.61467E-09	probable glucitol transport protein GutA
K16249	6.91621E-08	7.33898E-08	3.58878E-07	7.52357E-08	phenol hydroxylase P0 protein
K16254	1.01412E-06	2.17151E-08	8.48348E-08	1.76378E-07	mxaJ protein
K16255	9.76134E-07	6.53143E-09	2.25832E-09	6.87587E-08	cytochrome c-L
K16256	9.82587E-07	2.28944E-08	9.88028E-08	2.20476E-07	mxaA protein
K16257	9.82587E-07	2.28944E-08	9.88028E-08	2.20476E-07	mxaC protein
K16258	9.82587E-07	2.28944E-08	9.88028E-08	2.20476E-07	mxaK protein
K16259	9.82587E-07	2.28944E-08	9.88028E-08	2.20476E-07	mxaL protein
K16260	1.9472E-06	8.06445E-09	5.1626E-08	1.6897E-07	mxaD protein
K16263	4.65053E-08	2.1196E-07	1.38054E-06	1.37036E-05	amino acid efflux transporter
K16264	0.000443607	0.000440197	0.000438018	0.000426882	cobalt-zinc-cadmium efflux system protein
K16267	0.000215484	0.000215418	0.000205995	0.000155122	zinc and cadmium transporter
K16268	0	0	1.86811E-08	0	benzene/toluene/chlorobenzene dioxygenase subunit beta [EC:1.14.12.3 1.14.12.11 1.14.12.-]
K16269	2.40748E-07	5.85844E-07	2.74431E-06	6.92618E-06	cis-1,2-dihydrobenzene-1,2-diol dehydrogenase [EC:1.3.1.19 1.3.1.-]
K16291	2.64962E-06	1.45875E-06	2.68187E-06	1.94913E-05	L,D-transpeptidase ErfK/SrfK
K16293	0	0	4.25666E-07	4.64351E-06	polysulfide reductase chain B
K16294	0	0	4.26354E-07	3.70061E-06	polysulfide reductase chain C
K16299	6.16636E-07	3.27649E-07	1.6886E-06	2.84599E-07	ATP-binding cassette, subfamily C, bacterial EexD
K16300	4.41805E-07	8.39646E-08	3.6277E-07	3.85729E-06	membrane fusion protein, epimerase transport system
K16301	1.72493E-06	1.12455E-06	2.64615E-06	5.17216E-06	deferrochelatase/peroxidase EfeB [EC:1.11.1.-]
K16303	4.82798E-09	2.32054E-07	7.26771E-08	3.73947E-07	p-cumate 2,3-dioxygenase subunit beta [EC:1.14.12.25]
K16304	0	1.13298E-07	2.70567E-07	1.88602E-07	p-cumate 2,3-dioxygenase ferredoxin component
K16305	0	0	1.03238E-08	0	fructose-bisphosphate aldolase / 6-deoxy-5-ketofructose 1-phosphate synthase [EC:4.1.2.13 2.2.1.11]
K16306	2.39825E-08	2.19949E-09	6.73903E-07	9.15755E-08	fructose-bisphosphate aldolase / 2-amino-3,7-dideoxy-D-threo-hept-6-ulosonate synthase [EC:4.1.2.13 2.2.1.10]
K16317	1.34865E-08	6.53688E-07	7.46444E-08	5.26668E-06	tRNA (pseudouridine54-N1)-methyltransferase [EC:2.1.1.257]
K16318	5.54378E-08	0	0	2.2483E-08	tRNA (guanine6-N2)-methyltransferase [EC:2.1.1.256]
K16319	0.000213995	0.000212114	0.00018873	0.000114515	anthranilate 1,2-dioxygenase large subunit [EC:1.14.12.1]
K16320	9.86693E-08	1.42703E-07	1.02926E-06	1.81973E-07	anthranilate 1,2-dioxygenase small subunit [EC:1.14.12.1]
K16321	1.34739E-07	1.82741E-08	6.52869E-07	5.70377E-07	high-affinity gluconate transporter
K16322	2.53074E-06	1.05597E-06	2.3021E-06	5.57839E-05	low-affinity inorganic phosphate transporter
K16323	1.02627E-06	1.83275E-08	2.93805E-06	2.40046E-07	purine nucleoside transport protein
K16324	1.66784E-08	2.73396E-07	8.59373E-08	4.59563E-07	putative pseudouridine transporter

K16325	8.46296E-09	3.60159E-08	6.09453E-08	3.91206E-07	nucleoside permease
K16326	4.40495E-09	1.84394E-08	8.7346E-08	6.81297E-07	CRP/FNR family transcriptional regulator, putaive post-exponential-phase nitrogen-starvation regulator
K16327	8.56287E-10	2.24445E-08	4.38748E-08	2.58645E-08	putative LysE/RhtB family amino acid efflux pump
K16328	8.58449E-07	3.82773E-07	1.56694E-06	4.7023E-06	pseudouridine kinase [EC:2.7.1.83]
K16329	5.27988E-07	6.14931E-07	2.01154E-06	5.78419E-06	pseudouridylate synthase [EC:4.2.1.70]
K16331	0	2.05861E-07	6.35936E-08	0	sulfate permease
K16345	1.93428E-06	5.10573E-06	1.69779E-05	5.15837E-05	xanthine permease XanP
K16346	9.02553E-08	3.3834E-07	3.81063E-07	1.42158E-06	xanthine permease XanQ
K16347	3.19097E-07	1.28205E-07	2.28732E-06	4.8108E-07	entericidin A
K16348	1.24625E-07	4.46458E-07	2.90655E-07	8.39473E-07	entericidin B
K16363	0.000215725	0.000214395	0.000192304	0.000113566	UDP-3-O-[3-hydroxymyristoyl] N-acetylglucosamine deacetylase / 3-hydroxyacyl-[acyl-carrier-protein] dehydratase [
K16370	6.82291E-07	1.60646E-06	1.00651E-05	1.56847E-06	6-phosphofructokinase 2 [EC:2.7.1.11]
K16371	2.6955E-06	4.0949E-06	1.62867E-05	9.04419E-05	D-tagatose-1,6-bisphosphate aldolase subunit GatZ/KbaZ
K16372	1.20362E-10	1.19629E-07	1.45746E-06	3.00191E-08	niddamycin polyketide synthase 1
K16373	1.92579E-10	1.03243E-09	1.21735E-06	2.87435E-08	niddamycin polyketide synthase 3
K16374	0	2.12879E-08	6.03647E-07	5.94027E-08	niddamycin polyketide synthase 4/5
K16375	4.81422E-09	1.17214E-08	2.37937E-06	8.12126E-08	polyketide synthase FkbB
K16376	6.74025E-10	8.61934E-10	9.03535E-07	1.51917E-07	polyketide synthase FkbC
K16377	4.84026E-09	2.45254E-07	3.25092E-06	2.78186E-07	polyketide synthase FkbA
K16378	0.000645446	0.000638161	0.000574302	0.000380841	pipecolate-incorporating enzyme
K16379	0	3.74292E-09	3.02087E-07	0	cytochrome P450 C-9 hydroxylase
K16380	1.52803E-08	0	1.37875E-07	0	cytochrome P450 RapN
K16381	2.997E-09	0	1.71827E-07	0	31-O-methyltransferase
K16382	4.81447E-11	1.24081E-09	1.93681E-06	4.733E-08	polyene macrolide polyketide synthase, loading module
K16383	0	1.15481E-07	2.55E-06	1.37004E-07	polyene macrolide polyketide synthase, A-type KR domains
K16384	5.76292E-08	3.5277E-08	3.19739E-06	5.39193E-07	polyene macrolide polyketide synthase
K16385	5.46969E-08	9.57219E-08	3.64159E-06	3.4949E-07	polyene macrolide polyketide synthase, KS-AT-KR-ACP domains
K16386	3.63026E-08	4.7461E-08	3.02971E-06	2.06411E-07	polyene macrolide polyketide synthase
K16387	9.62893E-11	1.23133E-10	2.52351E-06	1.10604E-07	polyene macrolide polyketide synthase
K16388	0	0	2.22133E-08	0	polyene glycosyltransferase
K16389	1.88959E-08	1.55873E-07	9.44971E-07	1.06767E-07	cytochrome P450 monooxygenase
K16390	4.92514E-09	9.19807E-10	1.12536E-07	1.00293E-08	cytochrome P450 monooxygenase
K16391	2.43919E-07	6.99734E-07	4.53961E-06	7.11073E-06	pimaricinolide synthase, loading module
K16392	0	0	2.67936E-07	0	candicidin polyketide synthase FscA
K16393	1.20362E-10	1.53917E-10	8.80192E-07	7.00676E-09	candicidin polyketide synthase FscB
K16394	5.731E-08	2.88764E-08	1.47985E-07	7.33919E-08	epothilone polyketide synthase A
K16395	2.05277E-08	7.5592E-09	5.49811E-08	0	epothilone synthetase B
K16396	3.69509E-08	6.5978E-09	2.44032E-07	1.78604E-08	epothilone polyketide synthase C
K16397	5.07426E-09	3.17437E-07	1.37222E-06	4.11133E-07	epothilone polyketide synthase D
K16398	0	2.18337E-09	2.86483E-07	1.07788E-07	epothilone polyketide synthase E
K16400	1.71257E-09	0	0	0	cytochrome P450 enzyme
K16401	0	4.63652E-10	5.75925E-07	4.12162E-09	soraphen polyketide synthase A
K16402	7.29411E-07	2.02335E-06	1.00937E-05	2.08114E-05	soraphen polyketide synthase B
K16405	0	2.18337E-09	2.06627E-07	3.46615E-08	stigmatellin polyketide synthase StiA
K16416	0.000858174	0.000850495	0.000761296	0.000489095	myxalamid-type nonribosomal peptide synthetase MxaA
K16417	0.000642668	0.00063884	0.000577629	0.000375103	myxalamid-type polyketide synthase MxaC
K16420	9.96063E-07	8.08584E-08	3.37753E-06	2.8665E-07	rapamycin polyketide synthase A/B/C
K16421	0	0	1.97056E-08	0	4-hydroxymandelate synthase [EC:1.13.11.46]
K16422	3.02946E-06	4.80899E-06	1.87995E-05	3.80855E-05	4-hydroxymandelate oxidase [EC:1.1.3.46]
K16423	8.99101E-09	2.14715E-08	4.82839E-07	1.72743E-07	(S)-3,5-dihydroxyphenylglycine transaminase [EC:2.6.1.103]
K16424	0	3.74292E-09	7.69392E-08	0	3,5-dihydroxyphenylacetyl-CoA synthase [EC:2.3.1.246]
K16426	9.54425E-09	1.427E-08	1.17621E-07	2.02618E-07	dehydration protein DpgD
K16427	0	1.35681E-08	1.11725E-07	0	(3,5-dihydroxyphenyl)acetyl-CoA 1,2-dioxygenase [EC:1.13.11.80]
K16428	1.01403E-06	1.6945E-07	2.3386E-06	4.76762E-06	nonribosomal peptide synthetase CepA
K16429	0.000218333	0.000218021	0.000205014	0.00015722	nonribosomal peptide synthetase CepB
K16430	2.26267E-06	1.96696E-06	9.66793E-06	2.4359E-05	nonribosomal peptide synthetase CepC
K16431	6.93842E-07	1.92851E-06	6.19537E-06	1.4248E-05	FAD-dependent halogenase [EC:1.14.19.-]
K16432	1.08549E-06	8.53724E-08	1.45177E-06	1.96547E-06	nonribosomal peptide synthetase CepK
K16433	0	0	5.97251E-08	0	hydroxylation protein CepL
K16434	0	0	1.4951E-09	0	thioesterase CepJ
K16435	1.69364E-07	2.76293E-07	2.00787E-07	1.78604E-09	dTDP-4-dehydro-6-deoxy-alpha-D-glucopyranose 2,3-dehydratase [EC:4.2.1.159]
K16436	0.000215422	0.000213735	0.0001906	0.000122296	dTDP-3-amino-2,3,6-trideoxy-4-keto-D-glucose/dTDP-3-amino-3,4,6-trideoxy-alpha-D-glucose/dTDP-2,6-dideoxy-D
K16437	3.24676E-08	6.5978E-09	1.2697E-07	1.0991E-09	methylation protein EvaC
K16438	0	3.5636E-08	1.49678E-06	0	5-epimerase [EC:5.1.3.-]
K16443	0	0	2.22133E-08	1.4263E-08	desvancosaminyl-vancomycin vancosaminetransferase [EC:2.4.1.322]
K16444	7.82133E-07	7.07443E-07	2.43292E-06	3.0672E-05	vancomycin aglycone glucosyltransferase [EC:2.4.1.310]
K16445	0	1.23941E-07	8.63341E-08	0	oxidation protein CepE
K16447	0	1.37918E-07	1.65578E-07	6.78334E-09	oxidation protein CepG
K16509	1.63653E-06	6.91023E-07	7.75467E-06	6.73864E-06	regulatory protein spx
K16511	1.56898E-06	6.68552E-07	4.03601E-06	3.6238E-06	adapter protein MecA 1/2
K16514	3.22338E-06	3.73723E-06	1.66019E-05	8.78816E-05	4-oxalomesaconate tautomerase [EC:5.3.2.8]
K16515	1.1394E-06	9.07032E-08	1.23956E-07	1.29457E-07	4-oxalomesaconate hydratase [EC:4.2.1.83]
K16516	0.000428983	0.000426709	0.000386813	0.000265727	LysR family transcriptional regulator, regulator for genes of the gallate degradation pathway
K16517	2.98701E-07	7.15182E-08	1.47791E-06	2.04751E-07	porin-like protein GalP
K16552	0	4.03924E-08	1.38355E-07	1.27188E-09	polysaccharide biosynthesis/export protein ExoF
K16553	0	4.03924E-08	2.22254E-08	0	succinoglycan exporter
K16554	6.02672E-06	7.08058E-06	1.16294E-05	2.28447E-05	polysaccharide biosynthesis transport protein
K16555	1.71819E-06	1.43891E-06	1.44331E-06	5.10785E-06	succinoglycan biosynthesis protein ExoO [EC:2.4.-.-]
K16556	1.6279E-07	9.51131E-08	1.23597E-07	5.96711E-07	succinoglycan biosynthesis protein ExoM [EC:2.4.-.-]
K16557	9.69047E-07	1.75495E-07	2.99456E-07	6.33792E-08	succinoglycan biosynthesis protein ExoA [EC:2.4.-.-]
K16558	9.69047E-07	8.23177E-08	1.25855E-07	2.76147E-08	succinoglycan biosynthesis protein ExoL [EC:2.-.-.-]
K16559	9.69047E-07	8.23177E-08	1.06763E-07	2.22578E-09	endo-1,3-1,4-beta-glycanase ExoK [EC:3.2.1.-]
K16560	0	8.07847E-08	2.22254E-08	0	succinoglycan biosynthesis protein ExoH
K16561	7.9431E-09	0	7.32701E-08	2.28209E-08	succinoglycan biosynthesis protein ExoI
K16562	4.65959E-08	8.07847E-08	9.69067E-08	1.34593E-07	succinoglycan biosynthesis protein ExoW [EC:2.4.-.-]
K16563	1.00992E-06	4.19818E-08	6.20924E-08	5.15783E-07	succinoglycan biosynthesis protein ExoV
K16564	9.69047E-07	1.2271E-07	1.67024E-07	5.2571E-07	succinoglycan biosynthesis protein ExoU [EC:2.4.-.-]

K16565	0	4.03924E-08	8.96711E-08	0	exopolysaccharide production repressor protein
K16566	5.98451E-06	6.22753E-06	8.85947E-06	1.46641E-05	exopolysaccharide production protein ExoY
K16567	1.53799E-07	4.03924E-08	1.45819E-07	5.77648E-07	exopolysaccharide production protein ExoQ
K16568	1.9822E-06	4.57636E-07	1.05871E-06	6.36535E-06	exopolysaccharide production protein ExoZ
K16593	2.94252E-06	2.11811E-07	9.62484E-07	1.12761E-06	pimeloyl-[acyl-carrier protein] synthase [EC:1.14.14.46]
K16623	0	0	0	2.28005E-10	botulinum neurotoxin type non-toxic component
K16628	7.34313E-07	8.78774E-07	3.54122E-06	7.0019E-06	collagen, type VII, alpha
K16636	0	0	0	2.19557E-08	exoenzyme T
K16637	0	7.49763E-09	3.06336E-08	4.0058E-08	adenylate cyclase ExoY
K16638	0	1.87146E-10	0	9.9982E-09	exoenzyme U
K16644	9.34164E-09	0	0	0	actin-assembly inducing protein
K16645	0	1.34585E-07	2.19264E-07	1.76095E-06	heparin binding hemagglutinin HbhA
K16647	0	5.96007E-07	2.75127E-06	1.773E-06	arabinofuranan 3-O-arabinosyltransferase [EC:2.4.2.47]
K16648	1.62839E-07	8.85916E-07	2.49261E-06	1.8962E-06	arabinofuranan 3-O-arabinosyltransferase [EC:2.4.2.-]
K16649	4.10241E-08	6.00804E-07	2.85521E-06	1.84078E-06	rhamnopyranosyl-N-acetylglucosaminyl-diphospho-decaprenol beta-1,3/1,4-galactofuranosyltransferase [EC:2.4.1.287
K16650	6.17305E-08	6.27481E-07	4.33299E-06	5.51476E-06	galactofuranosylgalactofuranosylrhamnosyl-N-acetylglucosaminyl-diphospho-decaprenol beta-1,5/1,6-galactofuranosy
K16651	7.11995E-08	1.55493E-08	1.9381E-06	1.24499E-06	L-threonine kinase [EC:2.7.1.177]
K16652	2.6214E-07	1.41617E-06	7.99267E-06	2.04934E-05	decaprenylphospho-beta-D-erythro-pentofuranosid-2-ulose 2-reductase [EC:1.1.1.333]
K16653	8.95987E-07	2.12285E-06	1.15286E-05	4.32406E-05	decaprenylphospho-beta-D-ribofuranose 2-oxidase [EC:1.1.98.3]
K16654	0	0	4.48531E-09	0	spore-specific protein
K16692	0.000218191	0.000218208	0.000192174	0.000153304	tyrosine-protein kinase Etk/Wzc [EC:2.7.10.-]
K16693	1.00523E-07	3.10432E-07	1.01667E-07	1.48178E-06	enterobacterial common antigen flippase
K16694	0	0	2.2947E-08	6.6861E-08	teichuronic acid exporter
K16695	3.61118E-08	4.837E-08	1.12203E-07	2.15662E-07	lipopolysaccharide exporter
K16696	0	0	0	3.57207E-09	exopolysaccharide (amylovoran) exporter
K16697	0	0	3.75448E-07	1.10396E-08	teichuronic acid biosynthesis glycosyltransferase TuaC [EC:2.4.-.-]
K16698	2.66374E-06	2.68199E-06	2.85545E-06	7.53458E-06	teichuronic acid biosynthesis glycosyltransferase TuaG [EC:2.4.-.-]
K16699	9.85028E-09	1.83961E-09	2.22133E-08	1.4263E-08	teichuronic acid biosynthesis glycosyltransferase TuaH [EC:2.4.-.-]
K16700	1.29644E-11	3.22451E-11	1.37253E-07	1.18876E-08	amylovoran biosynthesis glycosyltransferase AmsB [EC:2.4.-.-]
K16701	2.73102E-07	3.66643E-07	1.74964E-07	7.7958E-08	amylovoran biosynthesis glycosyltransferase AmsD [EC:2.4.-.-]
K16702	1.2352E-06	5.24042E-08	3.00779E-06	1.06155E-06	amylovoran biosynthesis glycosyltransferase AmsE [EC:2.4.-.-]
K16703	2.96465E-08	5.50641E-08	2.08237E-07	1.14204E-06	colanic acid/amylovoran biosynthesis glycosyltransferase [EC:2.4.-.-]
K16704	9.82118E-08	3.01003E-07	2.24087E-07	1.47854E-06	dTDP-4-amino-4,6-dideoxy-D-galactose acyltransferase [EC:2.3.1.210]
K16705	0	0	4.72501E-07	2.0207E-08	teichuronic acid biosynthesis protein TuaE
K16707	1.01117E-07	1.8789E-07	3.21641E-07	1.58199E-08	UDP-galactose-lipid carrier transferase
K16708	0	0	0	3.57207E-09	amylovoran biosynthesis protein AmsC
K16709	5.54602E-09	2.2346E-08	1.58791E-09	2.96989E-08	amylovoran biosynthesis protein AmsF
K16710	8.69648E-09	1.13526E-06	8.71198E-08	1.00355E-06	colanic acid/amylovoran biosynthesis protein
K16711	4.20889E-09	1.91015E-08	4.79417E-08	2.82026E-07	colanic acid biosynthesis protein WcaM
K16712	0.000213765	0.000211774	0.000186082	0.000113202	EPS I polysaccharide export inner membrane protein EpsE
K16713	0.000213765	0.000211774	0.000186082	0.000113202	EPS I polysaccharide export inner membrane protein EpsF
K16783	2.62194E-06	7.29608E-06	2.32874E-05	3.67321E-05	biotin transport system permease protein
K16784	5.85858E-06	1.31198E-05	5.02765E-05	9.90662E-05	biotin transport system ATP-binding protein [EC:3.6.3.-]
K16785	7.09602E-06	4.5542E-06	3.75375E-05	1.96924E-05	energy-coupling factor transport system permease protein
K16786	0.000463402	0.000469888	0.000566665	0.00070718	energy-coupling factor transport system ATP-binding protein [EC:3.6.3.-]
K16787	0.000683173	0.000688706	0.000780933	0.000883786	energy-coupling factor transport system ATP-binding protein [EC:3.6.3.-]
K16788	2.29865E-07	1.08784E-07	2.6672E-07	1.55175E-06	niacin transporter
K16789	9.85586E-07	4.09826E-07	4.33497E-06	3.34288E-06	thiamine transporter
K16792	4.74018E-08	1.18379E-07	3.11016E-06	1.1301E-08	methanogen homoaconitase large subunit [EC:4.2.1.114]
K16793	4.96461E-09	1.92035E-07	2.77189E-06	1.24769E-08	methanogen homoaconitase small subunit [EC:4.2.1.114]
K16838	5.00199E-08	3.27506E-09	8.88096E-08	0	urate oxidase / 2-oxo-4-hydroxy-4-carboxy-5-ureidoimidazoline decarboxylase [EC:1.7.3.3 4.1.1.97]
K16839	1.2189E-06	2.44285E-07	1.26788E-06	3.65145E-06	FAD-dependent urate hydroxylase [EC:1.14.13.113]
K16840	9.35017E-07	1.36555E-07	1.1134E-06	7.20494E-06	2-oxo-4-hydroxy-4-carboxy-5-ureidoimidazoline decarboxylase [EC:4.1.1.97]
K16841	3.2214E-06	8.89384E-07	5.41592E-06	7.66468E-06	allantoin racemase [EC:5.1.99.3]
K16842	0.000216217	0.000215542	0.00020466	0.000153892	allantoinase [EC:3.5.2.5]
K16843	4.3344E-06	2.9292E-06	1.20488E-06	1.67197E-07	(S)-sulfolactate dehydrogenase [EC:1.1.1.310]
K16844	1.24161E-06	1.57775E-06	6.77765E-06	1.41796E-05	(2R)-3-sulfolactate dehydrogenase (NADP+) [EC:1.1.1.338]
K16845	3.08619E-07	5.70378E-08	2.66514E-07	5.66102E-08	(2R)-sulfolactate sulfo-lyase subunit alpha [EC:4.4.1.24]
K16846	3.08619E-07	7.3413E-08	2.8322E-07	5.58182E-08	(2R)-sulfolactate sulfo-lyase subunit beta [EC:4.4.1.24]
K16849	7.29827E-08	3.28738E-08	1.1401E-07	2.93411E-08	altronate dehydratase small subunit [EC:4.2.1.7]
K16850	8.05839E-08	3.73556E-07	5.14587E-07	2.10297E-07	altronate dehydratase large subunit [EC:4.2.1.7]
K16856	5.904E-06	8.24388E-06	3.7852E-05	7.31964E-05	ureidoglycolate lyase [EC:4.3.2.3]
K16868	8.45176E-07	6.20796E-07	1.08741E-06	3.46786E-06	tellurite methyltransferase [EC:2.1.1.265]
K16869	1.1259E-06	2.06813E-07	3.56269E-06	9.0834E-07	octanoyl-[GcvH]:protein N-octanoyltransferase [EC:2.3.1.204]
K16870	0.000215064	0.000213793	0.000190261	0.000115954	N-acetylglucosaminyl-diphospho-decaprenol L-rhamnosyltransferase [EC:2.4.1.289]
K16871	0.000215339	0.000213491	0.0001936	0.000123399	4-aminobutyrate---pyruvate transaminase [EC:2.6.1.96]
K16872	0.000215519	0.000212289	0.00018883	0.000117306	beta-ketodecanoyl-[acyl-carrier-protein] synthase [EC:2.3.1.207]
K16873	1.36357E-06	1.21825E-06	4.16838E-06	8.75974E-06	5-(hydroxymethyl)furfural/furfural oxidase [EC:1.1.3.47 1.1.3.-]
K16874	1.04494E-06	1.83838E-07	3.64894E-08	1.02629E-08	2,5-furandicarboxylate decarboxylase 1
K16875	3.83048E-08	1.20619E-07	2.44587E-07	1.91396E-06	2,5-furandicarboxylate decarboxylase 2
K16876	3.62204E-06	3.51643E-06	1.522E-05	6.60402E-05	2-furoate---CoA ligase [EC:6.2.1.31]
K16877	3.14554E-06	2.05117E-06	9.73629E-06	2.15487E-05	2-furoyl-CoA dehydrogenase large subunit [EC:1.3.99.8]
K16878	1.02692E-06	7.07593E-08	6.21993E-07	4.66778E-09	2-furoyl-CoA dehydrogenase FAD binding subunit [EC:1.3.99.8]
K16879	1.12231E-06	1.36257E-07	7.86969E-07	7.9976E-08	2-furoyl-CoA dehydrogenase 2Fe-2S iron sulfur subunit [EC:1.3.99.8]
K16880	1.05453E-06	1.40957E-07	6.92787E-07	3.08745E-07	2-oxoglutaroyl-CoA hydrolase
K16881	1.17815E-06	7.10483E-07	9.14481E-07	2.10647E-06	mannose-1-phosphate guanylyltransferase / phosphomannomutase [EC:2.7.7.13 5.4.2.8]
K16883	1.42608E-10	3.54696E-10	1.02698E-09	4.22436E-09	heat-stable enterotoxin STa/STI
K16885	0	1.66614E-09	1.1135E-07	0	quinone-modifying oxidoreductase, subunit QmoA
K16886	8.56287E-10	0	4.18629E-08	0	quinone-modifying oxidoreductase, subunit QmoB
K16887	0	0	2.09314E-08	0	quinone-modifying oxidoreductase, subunit QmoC
K16898	0.000220256	0.000215157	0.00020118	0.000133691	ATP-dependent helicase/nuclease subunit A [EC:3.1.-.- 3.6.4.12]
K16899	0.000217605	0.000213406	0.000197836	0.000117499	ATP-dependent helicase/nuclease subunit B [EC:3.1.-.- 3.6.4.12]
K16901	9.62893E-11	1.09004E-08	9.96735E-09	5.29948E-10	anthranilate 3-monooxygenase (FAD) / 4-hydroxyphenylacetate 3-monooxygenase [EC:1.14.14.8 1.14.14.9]
K16905	0	6.23613E-08	3.31593E-08	1.56236E-08	fluoroquinolone transport system permease protein
K16906	0	6.23613E-08	3.1968E-08	1.28284E-08	fluoroquinolone transport system permease protein
K16907	4.43126E-07	1.73011E-07	3.03745E-07	1.06434E-07	fluoroquinolone transport system ATP-binding protein [EC:3.6.3.-]
K16915	0	9.99684E-09	3.23896E-07	1.14104E-08	nickel transport protein

K16916	7.32176E-10	0	0	3.5645E-07	putative peptide transport system permease protein
K16917	7.32176E-10	0	0	1.92819E-07	putative peptide transport system ATP-binding protein
K16918	0	9.5208E-10	4.4087E-07	0	acetoin utilization transport system permease protein
K16920	2.31612E-06	9.30466E-07	7.86539E-06	7.20433E-05	acetoin utilization transport system ATP-binding protein
K16922	1.91221E-07	1.81363E-07	1.45022E-06	7.19686E-06	putative peptide zinc metalloprotease protein
K16923	3.59785E-07	4.86839E-07	1.87897E-06	1.00938E-06	energy-coupling factor transport system substrate-specific component
K16924	5.42389E-07	1.86635E-07	5.9918E-07	3.51481E-06	energy-coupling factor transport system substrate-specific component
K16925	1.83412E-06	7.57501E-07	1.12554E-05	4.85122E-06	energy-coupling factor transport system substrate-specific component
K16926	1.2723E-06	7.92617E-07	8.4497E-06	3.32299E-06	energy-coupling factor transport system substrate-specific component
K16927	8.3779E-07	7.23925E-07	6.93807E-06	4.60646E-07	energy-coupling factor transport system substrate-specific component
K16936	5.01007E-09	8.31816E-07	4.6355E-08	7.74022E-09	thiosulfate dehydrogenase [quinone] small subunit [EC:1.8.5.2]
K16937	1.07289E-06	8.45165E-07	3.9017E-06	1.03651E-07	thiosulfate dehydrogenase [quinone] large subunit [EC:1.8.5.2]
K16950	9.31813E-12	7.82303E-08	4.52033E-07	8.08074E-08	anaerobic sulfite reductase subunit A
K16951	9.31813E-12	6.80412E-08	2.61822E-07	8.08074E-08	anaerobic sulfite reductase subunit B
K16955	0	0	0	4.01858E-09	methylated-thiol--corrinoid protein
K16956	1.04582E-08	0	0	0	L-cystine transport system substrate-binding protein
K16957	3.16126E-07	6.20987E-09	2.90545E-07	2.66244E-06	L-cystine transport system substrate-binding protein
K16958	3.28524E-07	4.43559E-07	4.11936E-06	2.57107E-06	L-cystine transport system permease protein
K16959	0.000214151	0.000212469	0.000189802	0.000117147	L-cystine transport system permease protein
K16960	1.06303E-05	1.25698E-05	4.94503E-05	0.000106558	L-cystine transport system ATP-binding protein [EC:3.6.3.-]
K16961	1.61768E-07	3.604E-07	1.04146E-06	1.36595E-05	putative amino-acid transport system substrate-binding protein
K16962	1.51687E-06	2.18805E-07	1.64643E-06	1.47592E-05	putative amino-acid transport system permease protein
K16963	7.70168E-06	7.67087E-06	2.47299E-05	0.000113624	putative amino-acid transport system ATP-binding protein [EC:3.6.3.-]
K16964	0	1.01891E-08	1.18064E-08	4.27891E-09	dimethylsulfide dehydrogenase subunit alpha [EC:1.8.2.4]
K16968	1.79931E-09	1.08286E-08	0	2.22568E-08	methanesulfonate monooxygenase subunit alpha [EC:1.14.13.111]
K16969	1.79931E-09	1.847E-10	0	1.07162E-08	methanesulfonate monooxygenase subunit beta [EC:1.14.13.111]
K17050	7.2217E-11	1.36137E-08	1.89573E-07	2.26496E-08	complex iron-sulfur molybdoenzyme family reductase subunit alpha
K17051	1.24605E-08	1.01891E-08	8.72215E-08	1.07999E-06	complex iron-sulfur molybdoenzyme family reductase subunit beta
K17052	0	0	2.09314E-08	0	complex iron-sulfur molybdoenzyme family reductase subunit gamma
K17060	1.4096E-06	2.99777E-06	1.46255E-05	3.47082E-05	two-component system, sensor histidine kinase AauS [EC:2.7.13.3]
K17061	2.135E-06	3.72268E-06	1.73256E-05	4.52273E-05	two-component system, response regulator AauR
K17062	2.40012E-07	3.22421E-07	2.54055E-07	7.79191E-07	arginine/lysine/histidine/glutamine transport system substrate-binding and permease protein
K17063	3.70049E-07	3.44116E-07	3.46824E-06	4.42383E-07	arginine/lysine/histidine/glutamine transport system ATP-binding protein [EC:3.6.3.-]
K17064	1.18432E-07	4.89584E-08	5.25898E-07	5.23719E-07	D-2-hydroxyacid dehydrogenase (NADP+) [EC:1.1.1.272]
K17067	1.18375E-07	1.29554E-07	7.45099E-08	1.71319E-06	formaldehyde dismutase / methanol dehydrogenase [EC:1.2.98.1 1.1.99.37]
K17073	1.46653E-06	1.41133E-06	1.29681E-05	6.0626E-06	putative lysine transport system substrate-binding protein
K17074	1.60271E-06	1.72277E-06	1.87669E-05	7.31062E-06	putative lysine transport system permease protein
K17076	1.0272E-05	9.99871E-06	4.48055E-05	7.35692E-05	putative lysine transport system ATP-binding protein [EC:3.6.3.-]
K17103	0.000220625	0.000220176	0.000214524	0.000257298	CDP-diacylglycerol---serine O-phosphatidyltransferase [EC:2.7.8.8]
K17104	4.61151E-08	2.99444E-09	4.54168E-07	1.90174E-09	phosphoglycerol geranylgeranyltransferase [EC:2.5.1.41]
K17105	0	2.55502E-09	5.23286E-09	0	geranylgeranylglycerol-phosphate geranylgeranyltransferase [EC:2.5.1.42]
K17108	0	0	6.97714E-09	0	non-lysosomal glucosylceramidase [EC:3.2.1.45]
K17195	1.70762E-07	3.37776E-07	1.59055E-07	1.44808E-06	D-allulose-6-phosphate 3-epimerase [EC:5.1.3.-]
K17202	7.12738E-08	3.98847E-07	1.78857E-07	8.18932E-07	erythritol transport system substrate-binding protein
K17203	3.43638E-07	9.18327E-07	1.34829E-06	3.02067E-06	erythritol transport system permease protein
K17204	0.000646397	0.000641432	0.000584917	0.000390166	erythritol transport system ATP-binding protein
K17205	1.2714E-06	3.28995E-06	1.37536E-05	3.46222E-05	putative xylitol transport system substrate-binding protein
K17206	0.000215807	0.000216671	0.000205898	0.000165033	putative xylitol transport system permease protein
K17207	0.000215799	0.00021628	0.000203378	0.000165436	putative xylitol transport system ATP-binding protein
K17208	3.95568E-08	4.81524E-09	6.405E-08	4.20457E-08	inositol transport system substrate-binding protein
K17209	1.77736E-07	1.04737E-07	3.07069E-07	1.17636E-06	inositol transport system permease protein
K17210	4.64484E-07	7.38406E-07	3.66122E-06	7.37935E-06	inositol transport system ATP-binding protein
K17213	0.000429345	0.00042577	0.000378032	0.000240621	inositol transport system substrate-binding protein
K17214	0.00042941	0.000428494	0.000395735	0.0002774	inositol transport system permease protein
K17215	0.000646062	0.000642032	0.000584691	0.000420873	inositol transport system ATP-binding protein
K17216	2.62264E-06	9.23939E-07	5.53373E-06	4.07889E-06	cystathionine beta-synthase (O-acetyl-L-serine) [EC:2.5.1.134]
K17217	4.70223E-06	6.06735E-06	9.41299E-06	6.52362E-05	cystathionine gamma-lyase / homocysteine desulfhydrase [EC:4.4.1.1 4.4.1.2]
K17218	0.000218619	0.000216783	0.000204352	0.000172678	sulfide:quinone oxidoreductase [EC:1.8.5.4]
K17222	0.000214153	0.00021185	0.000186886	0.000113471	sulfur-oxidizing protein SoxA
K17223	0.00021415	0.00021185	0.000186872	0.000113466	sulfur-oxidizing protein SoxX
K17224	0.000214257	0.000211934	0.000187037	0.000113481	sulfur-oxidizing protein SoxB
K17225	4.89419E-06	3.21304E-06	1.5109E-05	3.51042E-05	sulfane dehydrogenase subunit SoxC
K17226	0.000215231	0.000212027	0.000187903	0.000113891	sulfur-oxidizing protein SoxY
K17227	0.000214497	0.00021188	0.000186919	0.000113535	sulfur-oxidizing protein SoxZ
K17228	2.57767E-06	4.74538E-07	3.43145E-06	4.0018E-06	dimethylsulfone monooxygenase [EC:1.14.14.35]
K17229	0.000213863	0.000211849	0.000186891	0.000113502	sulfide dehydrogenase [flavocytochrome c] flavoprotein chain [EC:1.8.2.3]
K17230	0	0	1.89411E-07	8.09436E-07	cytochrome subunit of sulfide dehydrogenase
K17234	8.67992E-08	9.77457E-08	1.25096E-08	3.42313E-09	arabinosaccharide transport system substrate-binding protein
K17235	1.77238E-07	1.10818E-07	1.416E-07	1.34611E-07	arabinosaccharide transport system permease protein
K17236	1.42237E-07	1.17996E-07	2.85089E-07	5.22978E-08	arabinosaccharide transport system permease protein
K17237	0	4.03924E-08	3.0315E-08	0	inositol-phosphate transport system substrate-binding protein
K17238	0	4.03924E-08	6.17121E-08	3.42313E-09	inositol-phosphate transport system permease protein
K17239	0	1.55737E-07	9.18826E-08	2.13946E-09	inositol-phosphate transport system permease protein
K17240	4.53892E-06	9.96156E-06	4.91962E-05	0.000109171	inositol-phosphate transport system ATP-binding protein
K17241	2.997E-09	4.31756E-08	4.33566E-07	8.42491E-08	alpha-1,4-digalacturonate transport system substrate-binding protein
K17242	3.09848E-07	4.68076E-07	8.53613E-07	2.51003E-07	alpha-1,4-digalacturonate transport system permease protein
K17243	5.99219E-08	1.47278E-07	8.98137E-07	5.8161E-07	alpha-1,4-digalacturonate transport system permease protein
K17244	5.62158E-08	4.83877E-08	3.01441E-07	5.89606E-08	putative chitobiose transport system substrate-binding protein
K17245	3.10793E-07	1.60768E-07	7.99891E-07	1.59403E-06	putative chitobiose transport system permease protein
K17246	2.46835E-07	6.45424E-07	1.25981E-06	1.80519E-07	putative chitobiose transport system permease protein
K17247	0.00021642	0.000216428	0.000204629	0.000154115	methionine sulfoxide reductase heme-binding subunit
K17248	5.69249E-08	1.35323E-07	3.27498E-07	1.90281E-06	N-acetylgalactosamine-N,N-diacetylbacillosaminyl-diphospho-undecaprenol 4-alpha-N-acetylgalactosaminyltransferas
K17249	9.42407E-08	4.678E-08	1.78913E-07	6.66171E-08	GalNAc-alpha-(1->4)-GalNAc-alpha-(1->3)-diNAcBac-PP-undecaprenol alpha-1,4-N-acetyl-D-galactosaminyltransfe
K17250	0	0	0	1.95517E-08	GalNAc5-diNAcBac-PP-undecaprenol beta-1,3-glucosyltransferase [EC:2.4.1.293]
K17251	0	0	9.54637E-09	0	undecaprenyl-diphosphooligosaccharide---protein glycotransferase [EC:2.4.99.19]
K17266	0	0	0	8.03716E-09	major vault protein
K17285	8.75386E-08	8.63677E-08	1.4106E-07	5.04533E-08	selenium-binding protein 1

K17312	2.97877E-09	1.06439E-08	5.33176E-08	4.93776E-08	trehalose transport system permease protein
K17314	6.24378E-08	1.32401E-07	3.68385E-07	2.75173E-08	trehalose transport system ATP-binding protein
K17315	1.72301E-06	2.89592E-06	1.93059E-05	3.44836E-05	glucose/mannose transport system substrate-binding protein
K17316	1.81555E-06	3.34133E-06	1.60758E-05	2.23646E-05	glucose/mannose transport system permease protein
K17317	1.60467E-06	2.81113E-06	1.657E-05	2.2994E-05	glucose/mannose transport system permease protein
K17318	3.21693E-06	3.69649E-06	8.59436E-06	5.70987E-06	putative aldouronate transport system substrate-binding protein
K17319	2.52309E-06	1.51078E-06	1.07737E-05	7.05438E-06	putative aldouronate transport system permease protein
K17320	3.5014E-06	2.31935E-06	1.76185E-05	1.41266E-05	putative aldouronate transport system permease protein
K17321	0.000215048	0.000214815	0.000203819	0.000148583	glycerol transport system substrate-binding protein
K17322	1.38766E-06	3.08888E-06	1.84844E-05	3.53523E-05	glycerol transport system permease protein
K17323	1.31444E-06	3.07961E-06	1.74439E-05	3.54132E-05	glycerol transport system permease protein
K17324	0.000217549	0.000216912	0.000216047	0.000216787	glycerol transport system ATP-binding protein
K17325	3.15489E-06	6.17861E-06	3.53435E-05	7.7034E-05	glycerol transport system ATP-binding protein
K17326	9.62893E-11	1.23133E-10	4.35031E-08	5.94249E-09	xylobiose transport system substrate-binding protein
K17327	9.85028E-09	1.83961E-09	3.74727E-08	1.78604E-09	xylobiose transport system permease protein
K17328	0	0	3.74727E-08	1.78604E-09	xylobiose transport system permease protein
K17329	1.16971E-07	2.24856E-07	5.57667E-07	1.54331E-07	N,N-diacetylchitobiose transport system substrate-binding protein
K17330	1.03912E-07	1.9965E-07	8.333E-07	3.30079E-07	N,N-diacetylchitobiose transport system permease protein
K17331	1.08875E-07	2.01021E-07	1.50566E-06	4.53312E-07	N,N-diacetylchitobiose transport system permease protein
K17363	1.40381E-07	1.48691E-07	4.70046E-07	7.29084E-08	urocanate reductase [EC:1.3.99.33]
K17364	9.69047E-07	1.53301E-09	8.07261E-09	3.57629E-08	dihydromethanopterin reductase [EC:1.5.1.47]
K17398	0	7.15054E-07	5.64293E-07	0	DNA (cytosine-5)-methyltransferase 3A [EC:2.1.1.37]
K17462	7.38321E-07	4.02109E-07	1.43324E-06	1.71909E-07	putative AdoMet-dependent methyltransferase [EC:2.1.1.-]
K17463	9.68393E-08	2.84036E-07	2.39118E-07	9.50504E-06	2-dehydro-3-deoxy-phosphogluconate aldolase [EC:4.1.2.14]
K17464	7.40724E-10	1.84234E-09	5.33426E-09	8.66863E-07	PTS system, D-glucosaminate-specific IIA component [EC:2.7.1.203]
K17465	7.40724E-10	1.04167E-07	5.33426E-09	8.67993E-07	PTS system, D-glucosaminate-specific IIB component [EC:2.7.1.203]
K17466	7.50447E-10	1.20566E-07	5.40428E-09	9.08167E-07	PTS system, D-glucosaminate-specific IIC component
K17467	3.15361E-09	1.35E-07	1.4172E-07	3.60906E-06	PTS system, D-glucosaminate-specific IID component
K17468	6.04058E-10	1.50242E-09	1.46497E-08	3.81394E-07	D-glucosaminate-6-phosphate ammonia-lyase [EC:4.3.1.29]
K17472	1.11977E-06	4.55101E-07	3.56276E-06	6.69794E-08	Rrf2 family transcriptional regulator, cysteine metabolism repressor
K17473	2.30567E-08	1.85894E-07	1.22299E-07	2.21524E-06	sigma-54 dependent transcriptional regulator, dga operon transcriptional activator
K17474	2.44266E-08	1.75935E-08	7.99436E-08	5.12614E-07	pulcherriminic acid synthase [EC:1.14.15.13]
K17476	1.8498E-06	2.8271E-07	2.30208E-06	1.72223E-07	pentalenolactone synthase [EC:1.14.19.8]
K17483	0	0	1.86278E-08	5.4955E-10	mycocyclosin synthase [EC:1.14.21.9]
K17484	0	0	1.42038E-07	2.23002E-08	cyclo(L-tyrosyl-L-tyrosyl) synthase [EC:2.3.2.21]
K17485	1.24386E-08	0	1.6748E-09	5.05007E-07	cyclo(L-leucyl-L-leucyl) synthase [EC:2.3.2.22]
K17486	0	0	1.16286E-08	0	dimethylsulfoniopropionate demethylase [EC:2.1.1.269]
K17488	4.92514E-09	9.19807E-10	9.0419E-08	0	GTP cyclohydrolase IV [EC:3.5.4.39]
K17489	2.99206E-06	6.47975E-06	1.98131E-05	2.40853E-06	methylmalonyl-CoA carboxyltransferase 12S subunit [EC:2.1.3.1]
K17490	5.38173E-07	2.6348E-07	5.93353E-06	3.16896E-06	methylmalonyl-CoA carboxyltransferase 1.3S subunit [EC:2.1.3.1]
K17550	0	1.02051E-08	7.19544E-10	0	protein phosphatase 1 regulatory subunit 7
K17616	1.18886E-07	8.05403E-08	2.54574E-07	5.13917E-06	CTD small phosphatase-like protein 2 [EC:3.1.3.-]
K17623	0.000213765	0.00021218	0.000186339	0.000121214	pseudouridine 5-phosphatase [EC:3.1.3.96]
K17624	7.72054E-07	6.66855E-07	6.93299E-06	1.26059E-06	endo-alpha-N-acetylgalactosaminidase [EC:3.2.1.97]
K17625	2.0979E-08	0	2.09818E-07	6.889E-09	dTDP-4-keto-6-deoxyhexose reductase [EC:1.1.1.-]
K17640	4.69703E-08	1.80414E-08	2.35998E-07	1.91846E-06	LacI family transcriptional regulator, xylobiose transport system transcriptional regulator
K17641	4.37607E-08	2.05701E-08	3.35995E-07	1.93572E-06	beta-xylosidase
K17662	1.70111E-06	1.99396E-07	6.03711E-07	1.51476E-07	cytochrome b pre-mRNA-processing protein 3
K17675	3.95592E-06	1.97816E-06	9.44905E-06	6.41752E-07	ATP-dependent RNA helicase SUPV3L1/SUV3 [EC:3.6.4.13]
K17680	1.10632E-06	1.96771E-06	6.35108E-06	2.15438E-05	twinkle protein [EC:3.6.4.12]
K17686	0.000673312	0.000679423	0.000675138	0.000658888	Cu+-exporting ATPase [EC:3.6.3.54]
K17713	0.000216577	0.000214034	0.0001936	0.000183196	outer membrane protein assembly factor BamB
K17716	2.09444E-06	2.02149E-06	3.58486E-06	7.01857E-06	UDP-glucose 4-epimerase [EC:5.1.3.2]
K17717	0.000213768	0.000211826	0.000186105	0.000113236	phospholipase D [EC:3.1.4.4]
K17722	4.34174E-06	6.91037E-06	2.54902E-05	6.01715E-05	dihydropyrimidine dehydrogenase (NAD+) subunit PreT [EC:1.3.1.1]
K17723	1.34191E-06	3.23467E-06	1.71818E-05	3.50748E-05	dihydropyrimidine dehydrogenase (NAD+) subunit PreA [EC:1.3.1.1]
K17733	0.000215076	0.000214188	0.000189694	0.000138947	peptidoglycan LD-endopeptidase CwlK [EC:3.4.-.-]
K17734	1.06374E-06	2.4206E-06	8.22288E-06	1.76531E-05	serine protease AprX [EC:3.4.21.-]
K17735	1.0409E-06	6.9481E-07	4.42894E-06	3.77918E-06	carnitine 3-dehydrogenase [EC:1.1.1.108]
K17736	0.000214623	0.00021293	0.000190328	0.000125243	AraC family transcriptional regulator, carnitine catabolism transcriptional activator
K17737	0.000433063	0.000431388	0.000406801	0.000396017	LysR family transcriptional regulator, carnitine catabolism transcriptional activator
K17743	4.66814E-08	9.29511E-09	1.92867E-07	1.39408E-06	D-xylose reductase [EC:1.1.1.307]
K17744	0	0	2.58094E-09	0	L-galactose dehydrogenase [EC:1.1.1.316]
K17745	6.98963E-09	2.90033E-08	2.03885E-06	3.21288E-06	sepiapterin reductase [EC:1.1.1.325]
K17750	6.79724E-08	0	2.06475E-09	1.70245E-08	3-benzylmalate dehydrogenase [EC:1.1.1.-]
K17752	1.87784E-06	3.57578E-06	1.81603E-05	4.06175E-05	serine/threonine-protein kinase RsbT [EC:2.7.11.1]
K17754	3.48867E-08	6.29452E-08	2.63099E-07	1.82844E-06	cyclopentanol dehydrogenase [EC:1.1.1.163]
K17755	8.75552E-07	2.78113E-06	4.99625E-07	3.73844E-06	choline oxidase [EC:1.1.3.17]
K17758	0.000440754	0.000441498	0.000433613	0.000377507	ADP-dependent NAD(P)H-hydrate dehydratase [EC:4.2.1.136]
K17759	0.000438469	0.000436032	0.000412107	0.000335588	NAD(P)H-hydrate epimerase [EC:5.1.99.6]
K17760	4.34758E-06	4.85562E-06	2.10148E-05	4.93658E-05	quinohemoprotein ethanol dehydrogenase [EC:1.1.9.1]
K17762	1.57863E-06	2.94907E-06	1.48938E-05	3.9125E-05	rsbT antagonist protein RsbS
K17763	2.37042E-06	2.95334E-06	1.9726E-05	4.03711E-05	rsbT co-antagonist protein RsbR
K17810	5.30284E-07	3.36008E-07	8.1188E-07	1.2307E-07	D-aspartate ligase [EC:6.3.1.12]
K17816	0	0	2.58094E-09	0	8-oxo-dGTP diphosphatase / 2-hydroxy-dATP diphosphatase [EC:3.6.1.55 3.6.1.56]
K17818	0	0	6.38998E-08	0	D-arabinitol dehydrogenase (NADP+) [EC:1.1.1.287]
K17828	3.48067E-06	5.02715E-06	3.00822E-05	3.84619E-05	dihydroorotate dehydrogenase (NAD+) catalytic subunit [EC:1.3.1.14]
K17829	9.78038E-07	1.96914E-07	8.5621E-07	2.2114E-07	crotonyl-CoA reductase [EC:1.3.1.86]
K17830	2.27728E-09	1.0572E-07	6.22735E-07	2.29449E-06	digeranylgeranylglycerophospholipid reductase [EC:1.3.1.101 1.3.7.11]
K17831	3.24676E-08	1.6423E-08	0	1.06924E-06	L-lysine 6-oxidase [EC:1.4.3.20]
K17835	6.01808E-10	2.95296E-09	3.1186E-07	2.66243E-08	3-amino-4-hydroxybenzoic acid synthase [EC:4.1.99.20]
K17836	6.45771E-06	6.17408E-06	3.4497E-05	9.78232E-05	beta-lactamase class A [EC:3.5.2.6]
K17837	2.22746E-06	1.81306E-06	5.09909E-06	5.50456E-05	metallo-beta-lactamase class B [EC:3.5.2.6]
K17838	0.000216627	0.000218545	0.000217262	0.000193968	beta-lactamase class D [EC:3.5.2.6]
K17840	0	2.51215E-07	1.09024E-07	5.86355E-08	aminoglycoside 2-N-acetyltransferase I [EC:2.3.1.59]
K17850	0.000220125	0.000220842	0.000227907	0.000303394	LysR family transcriptional regulator, regulator of gene expression of beta-lactamase
K17865	5.09968E-06	3.60747E-06	1.70821E-05	3.73782E-05	3-hydroxybutyryl-CoA dehydratase [EC:4.2.1.55]

K17869	1.3168E-07	2.85327E-08	5.57025E-07	6.81515E-07	NADH oxidase (H2O-forming) [EC:1.6.3.4]
K17870	1.75534E-07	3.77245E-07	4.80841E-07	6.18012E-06	NADH oxidase (H2O2-forming) [EC:1.6.3.3]
K17876	5.71052E-08	5.22004E-07	2.66022E-06	5.32669E-07	pentalenic acid synthase [EC:1.14.15.11]
K17880	2.33566E-08	1.31002E-09	1.13925E-07	0	hygromycin-B 7-O-kinase [EC:2.7.1.119]
K17881	1.40919E-09	5.70211E-09	1.07638E-09	5.09649E-09	aminoglycoside 2-adenylyltransferase [EC:2.7.7.46]
K17882	0	4.39422E-10	3.11714E-07	0	kanamycin nucleotidyltransferase [EC:2.7.7.-]
K17883	0.00021634	0.000216323	0.000197745	0.00011766	mycothione reductase [EC:1.8.1.15]
K17884	1.18766E-06	3.20802E-06	7.60105E-07	9.66622E-08	archaetidylinositol phosphate synthase [EC:2.7.8.39]
K17892	0	0	5.03714E-08	0	ferredoxin-thioredoxin reductase catalytic chain [EC:1.8.7.2]
K17893	9.28626E-08	1.64915E-08	2.92567E-07	1.32379E-07	ubiquinol oxidase [EC:1.10.3.11]
K17898	5.29756E-07	2.75967E-07	6.20656E-06	1.00206E-07	D-ornithine 4,5-aminomutase subunit beta [EC:5.4.3.5]
K17899	5.05029E-07	2.61814E-07	5.94649E-06	6.84249E-08	D-ornithine 4,5-aminomutase subunit alpha [EC:5.4.3.5]
K17910	0	4.39422E-10	1.30952E-07	0	aminoglycoside 2-phosphotransferase [EC:2.7.1.190]
K17938	5.42922E-08	1.36256E-07	2.63952E-06	1.35718E-06	peptide/bleomycin uptake transporter
K17940	0	5.0142E-07	9.38241E-07	1.9177E-08	2-heptyl-3-hydroxy-4(1H)-quinolone synthase [EC:1.14.13.182]
K17947	5.35674E-07	3.97153E-07	2.55195E-06	1.14136E-05	dTDP-L-rhamnose 4-epimerase [EC:5.1.3.25]
K17948	4.93611E-09	1.97605E-08	4.60126E-08	3.19385E-06	N-acetylneuraminate epimerase [EC:5.1.3.24]
K17950	5.00738E-07	2.50708E-07	1.29127E-06	5.19966E-07	L-cysteate sulfo-lyase [EC:4.4.1.25]
K17989	0.000215065	0.000214832	0.000200022	0.000147865	L-serine/L-threonine ammonia-lyase [EC:4.3.1.17 4.3.1.19]
K17992	0	3.86545E-08	6.3391E-07	3.43263E-09	NADP-reducing hydrogenase subunit HndB [EC:1.12.1.3]
K17993	3.2515E-08	2.66119E-08	1.71364E-08	0	sulfhydrogenase subunit alpha [EC:1.12.1.3 1.12.1.5]
K17994	0	0	0	7.88243E-09	sulfhydrogenase subunit delta [EC:1.12.1.3 1.12.1.5]
K17995	0	1.63753E-08	1.73009E-08	0	sulfhydrogenase subunit gamma (sulfur reductase) [EC:1.12.98.4]
K17996	0	0	3.12552E-08	0	sulfhydrogenase subunit beta (sulfur reductase) [EC:1.12.98.4]
K17997	2.23408E-08	9.28572E-08	1.03007E-07	1.25974E-08	iron-hydrogenase subunit alpha [EC:1.12.1.4]
K17998	0	6.11549E-09	1.88098E-07	1.09397E-07	iron-hydrogenase subunit beta [EC:1.12.1.4]
K17999	0	0	3.09713E-08	6.78334E-09	iron-hydrogenase subunit gamma [EC:1.12.1.4]
K18000	0.000213802	0.000211802	0.000186163	0.000113202	anthranilate-CoA ligase [EC:6.2.1.32]
K18004	0	7.49763E-09	6.3447E-08	0	HQNO biosynthesis monooxygenase PqsL
K18005	1.88988E-06	1.65114E-06	6.91082E-06	2.19344E-06	[NiFe] hydrogenase diaphorase moiety large subunit [EC:1.12.1.2]
K18006	6.74544E-09	1.36166E-07	1.95138E-07	2.10912E-07	[NiFe] hydrogenase diaphorase moiety small subunit [EC:1.12.1.2]
K18007	1.59136E-08	5.67111E-08	1.30664E-07	9.83415E-08	NAD-reducing hydrogenase small subunit [EC:1.12.1.2]
K18008	0	0	2.58094E-09	5.35811E-09	[NiFe] hydrogenase small subunit [EC:1.12.2.1]
K18009	0.000223229	0.000222913	0.000246124	0.000217659	meso-butanediol dehydrogenase / (S,S)-butanediol dehydrogenase / diacetyl reductase [EC:1.1.1.- 1.1.1.76 1.1.1.304]
K18011	0	4.19867E-08	5.58662E-07	7.81044E-08	beta-lysine 5,6-aminomutase beta subunit [EC:5.4.3.3]
K18012	0	4.19867E-08	6.20605E-07	7.81044E-08	L-erythro-3,5-diaminohexanoate dehydrogenase [EC:1.4.1.11]
K18013	5.61967E-06	3.58282E-06	2.05664E-05	3.73999E-05	3-keto-5-aminohexanoate cleavage enzyme [EC:2.3.1.247]
K18014	0	1.87137E-07	6.19318E-07	8.90276E-08	3-aminobutyryl-CoA ammonia-lyase [EC:4.3.1.14]
K18015	4.48462E-09	1.86376E-08	3.22956E-08	1.97549E-07	deubiquitinase [EC:3.4.22.-]
K18020	0	6.60734E-09	3.29285E-08	0	glyceraldehyde dehydrogenase large subunit [EC:1.2.99.8]
K18021	0	0	1.20974E-07	0	glyceraldehyde dehydrogenase medium subunit [EC:1.2.99.8]
K18022	1.69344E-08	1.5625E-08	1.23676E-07	1.04456E-08	glyceraldehyde dehydrogenase small subunit [EC:1.2.99.8]
K18023	1.81107E-08	3.26502E-08	8.17685E-08	4.98703E-08	membrane-bound hydrogenase subunit mbhJ [EC:1.12.7.2]
K18028	1.60096E-06	3.41921E-06	1.61967E-05	3.491E-05	2,5-dihydroxypyridine 5,6-dioxygenase [EC:1.13.11.9]
K18029	3.67348E-06	6.80747E-06	3.10306E-05	7.03433E-05	nicotinate dehydrogenase subunit A [EC:1.17.2.1]
K18030	0.000225643	0.000236253	0.000293638	0.000418761	nicotinate dehydrogenase subunit B [EC:1.17.2.1]
K18058	0	0	1.70352E-07	0	L-asparagine oxygenase [EC:1.14.11.39]
K18067	4.26172E-07	6.34109E-07	3.19444E-06	8.85488E-06	phthalate 4,5-cis-dihydrodiol dehydrogenase [EC:1.3.1.64]
K18068	5.80066E-07	1.38402E-06	5.80698E-06	1.41173E-05	phthalate 4,5-dioxygenase [EC:1.14.12.7]
K18069	1.47187E-06	3.64565E-06	1.66258E-05	4.17876E-05	phthalate 4,5-dioxygenase reductase component [EC:1.18.1.-]
K18070	0.00043259	0.00042992	0.000401037	0.000295861	MFS transporter, ACS family, phthalate transporter
K18071	5.29591E-10	6.77234E-10	2.32571E-08	0	2-methyl-3-hydroxypyridine 5-carboxylic acid dioxygenase [EC:1.14.13.242]
K18072	7.70484E-07	6.58442E-07	2.37476E-06	5.17683E-05	two-component system, OmpR family, sensor kinase ParS [EC:2.7.13.3]
K18073	2.89716E-06	1.14503E-06	4.14833E-06	7.07193E-05	two-component system, OmpR family, response regulator ParR
K18074	4.96461E-09	1.10044E-08	1.96419E-07	5.70522E-08	terephthalate 1,2-dioxygenase oxygenase component alpha subunit [EC:1.14.12.15]
K18075	0	1.17924E-09	1.87291E-07	5.70522E-08	terephthalate 1,2-dioxygenase oxygenase component beta subunit [EC:1.14.12.15]
K18076	4.96461E-09	1.17924E-09	1.92175E-07	6.27183E-08	1,2-dihydroxy-3,5-cyclohexadiene-1,4-dicarboxylate dehydrogenase [EC:1.3.1.53]
K18077	0	1.17924E-09	1.47579E-07	0	terephthalate 1,2-dioxygenase reductase component [EC:1.18.1.-]
K18087	7.2217E-11	2.69919E-10	1.48158E-07	1.87788E-07	biphenyl 2,3-dioxygenase ferredoxin component
K18088	1.39492E-07	4.86895E-09	1.34329E-07	1.73222E-08	biphenyl 2,3-dioxygenase ferredoxin reductase component [EC:1.18.1.3]
K18089	7.2217E-11	2.69919E-10	1.47507E-07	8.14E-10	benzene/toluene/chlorobenzene dioxygenase ferredoxin component
K18090	7.2217E-11	2.69919E-10	1.27351E-07	8.14E-10	benzene/toluene/chlorobenzene dioxygenase ferredoxin reductase component [EC:1.18.1.3 1.18.1.-]
K18091	6.50049E-08	1.23941E-07	5.8088E-07	4.67299E-07	pentalenolactone D synthase [EC:1.14.13.170 1.14.13.171]
K18092	2.40723E-11	1.18691E-07	1.05905E-07	7.79192E-09	2-hydroxy-6-oxo-octa-2,4-dienoate hydrolase [EC:3.7.1.-]
K18093	1.39482E-06	2.12969E-06	1.23876E-05	1.99663E-06	imipenem/basic amino acid-specific outer membrane pore [EC:3.4.21.-]
K18094	0.000215117	0.000214435	0.000197821	0.000148417	membrane fusion protein, multidrug efflux system
K18095	0.000224956	0.000227621	0.000255494	0.000391971	multidrug efflux pump
K18096	1.53241E-06	9.1334E-08	6.01982E-07	1.87602E-08	acyl-homoserine lactone synthase [EC:2.3.1.228 2.3.1.229 2.3.1.-]
K18098	1.32476E-06	1.20572E-07	8.52532E-07	1.02437E-07	LuxR family transcriptional regulator, quorum-sensing system regulator BjaR1
K18099	0	0	3.48857E-09	4.68835E-09	LuxR family transcriptional regulator, quorum-sensing system regulator RhlR
K18100	4.40524E-09	3.78498E-08	1.38083E-06	2.36276E-07	rhamnosyltransferase subunit A [EC:2.4.1.-]
K18101	1.16457E-06	9.83818E-08	4.76862E-07	4.14877E-08	rhamnosyltransferase subunit B [EC:2.4.1.-]
K18104	9.18571E-06	3.18366E-06	2.2305E-05	1.44988E-05	ATP-binding cassette, subfamily B, bacterial AbcA/BmrA [EC:3.6.3.44]
K18105	0	2.35848E-09	0	0	RNA 3-terminal phosphate cyclase (GTP) [EC:6.5.1.5]
K18111	0	0	3.48857E-09	0	(+)-beta-caryophyllene/(+)-caryolan-1-ol synthase [EC:4.2.3.89 4.2.1.138]
K18115	8.27652E-08	4.52516E-08	2.05582E-07	1.95517E-08	sporulenol synthase [EC:4.2.1.137]
K18118	4.66254E-06	1.01137E-05	2.85691E-05	9.03747E-05	succinyl-CoA:acetate CoA-transferase [EC:2.8.3.18]
K18119	9.16819E-09	1.06439E-08	7.37412E-09	4.01858E-09	succinate-semialdehyde dehydrogenase [EC:1.2.1.76]
K18120	4.11108E-07	4.56481E-07	1.26599E-06	5.44553E-07	4-hydroxybutyrate dehydrogenase [EC:1.1.1.61]
K18121	0	0	9.25404E-08	1.90174E-09	glyoxylate/succinic semialdehyde reductase [EC:1.1.1.79 1.1.1.-]
K18122	3.602E-06	4.88001E-06	2.60507E-05	3.63573E-05	4-hydroxybutyrate CoA-transferase [EC:2.8.3.-]
K18123	3.97288E-08	0	1.21886E-07	5.68436E-11	4-hydroxy-2-oxoglutarate aldolase [EC:4.1.3.16]
K18126	0	1.31002E-09	0	0	2-dehydro-3-deoxygluconokinase / 2-dehydro-3-deoxygalactonokinase [EC:2.7.1.178]
K18128	3.51552E-07	5.86902E-07	2.92755E-06	7.30822E-06	D-glyceraldehyde dehydrogenase (NADP+) [EC:1.2.1.89]
K18129	1.07845E-08	5.04002E-08	2.02754E-07	8.58516E-09	TetR/AcrR family transcriptional regulator, mexXY operon repressor
K18132	1.58684E-08	3.33228E-09	2.07658E-08	2.15111E-07	major outer membrane protein P.IA
K18133	4.04966E-08	0	2.59277E-08	2.31619E-07	major outer membrane protein P.IB

K18135	3.31375E-07	4.35816E-07	1.38932E-06	3.07114E-07	TetR/AcrR family transcriptional regulator, repressor of the mexAB-oprM multidrug resistance operon
K18136	3.97169E-09	2.37832E-08	5.22288E-07	1.19179E-05	TetR/AcrR family transcriptional regulator, multidrug resistance operon repressor
K18137	0	0	3.27286E-08	7.07501E-07	TetR/AcrR family transcriptional regulator, repressor of the adeIJK operon
K18138	0.000887362	0.000885512	0.000881163	0.001112402	multidrug efflux pump
K18139	0.002161196	0.0021534	0.002015959	0.001812481	outer membrane protein, multidrug efflux system
K18140	6.07239E-08	3.69523E-08	1.03135E-06	8.27993E-07	TetR/AcrR family transcriptional regulator, acrEF/envCD operon repressor
K18141	4.59676E-07	8.44751E-07	1.5688E-06	2.03371E-05	membrane fusion protein, multidrug efflux system
K18142	0.000215825	0.000214593	0.000195323	0.000208706	multidrug efflux pump
K18143	2.91535E-06	4.02306E-06	2.13092E-05	0.000121482	two-component system, OmpR family, sensor histidine kinase AdeS [EC:2.7.13.3]
K18144	6.30422E-06	9.2437E-06	4.32726E-05	0.000166755	two-component system, OmpR family, response regulator AdeR
K18145	2.91381E-06	3.47396E-06	1.75931E-05	7.71826E-05	membrane fusion protein, multidrug efflux system
K18146	0.000443713	0.000445514	0.000461729	0.000646623	multidrug efflux pump
K18147	0	0	6.76143E-08	7.29871E-08	outer membrane protein, multidrug efflux system
K18148	2.48638E-06	5.90586E-06	2.76055E-05	6.96013E-05	release factor H-coupled RctB family protein
K18149	1.04582E-08	0	0	6.45801E-07	penicillin-binding protein
K18151	4.23211E-08	1.17924E-09	1.70726E-07	8.65957E-10	ureidoglycolate amidohydrolase [EC:3.5.1.116]
K18164	1.72459E-06	2.05994E-07	6.29243E-07	1.82223E-07	NADH dehydrogenase [ubiquinone] 1 alpha subcomplex assembly factor 7
K18195	2.997E-09	0	1.85729E-07	3.98855E-08	rhamnogalacturonan endolyase [EC:4.2.2.23]
K18197	1.51466E-06	2.9288E-06	6.75488E-06	3.26133E-05	rhamnogalacturonan endolyase [EC:4.2.2.23]
K18198	8.37561E-08	3.95743E-08	3.90578E-07	1.91579E-06	rhamnogalacturonan exolyase [EC:4.2.2.24]
K18199	0.000434601	0.000431611	0.00041052	0.000318347	cyclohexyl-isocyanide hydratase [EC:4.2.1.103]
K18200	2.84943E-08	0	8.93407E-08	0	peptidylamidoglycolate lyase [EC:4.3.2.5]
K18205	0	1.02051E-08	2.06778E-07	2.57496E-08	non-reducing end beta-L-arabinofuranosidase [EC:3.2.1.185]
K18206	2.997E-09	0	1.87514E-07	2.57496E-08	beta-L-arabinobiosidase [EC:3.2.1.187]
K18209	2.02134E-07	2.0867E-07	5.85823E-07	1.60876E-06	fumarate reductase (CoM/CoB) subunit A [EC:1.3.4.1]
K18210	0	0	2.36541E-08	8.10841E-09	fumarate reductase (CoM/CoB) subunit B [EC:1.3.4.1]
K18214	2.80762E-07	2.51587E-07	1.08235E-07	1.90174E-09	MFS transporter, DHA3 family, tetracycline resistance protein
K18215	8.14197E-08	5.76207E-07	1.80693E-07	1.88785E-06	MFS transporter, DHA3 family, tetracycline resistance protein
K18216	5.05029E-07	2.74802E-07	5.59299E-06	3.5877E-06	ATP-binding cassette, subfamily B, tetracycline resistant protein
K18217	1.30238E-07	1.60381E-08	2.42732E-07	4.51224E-06	ATP-binding cassette, subfamily B, tetracycline resistant protein
K18218	8.58891E-07	7.36397E-07	2.41363E-06	2.41963E-05	tetracycline resistance efflux pump
K18219	1.32071E-06	5.70367E-07	2.49016E-06	5.59853E-05	sigma-54 dependent transcriptional regulator, tetracycline resistant transcriptional regulator
K18220	1.07216E-06	1.06919E-06	6.02072E-06	5.88322E-07	ribosomal protection tetracycline resistance protein
K18221	1.62054E-11	4.68671E-08	5.408E-07	5.57682E-09	tetracycline 11a-monooxygenase, tetracycline resistance protein [EC:1.14.13.231]
K18223	1.25346E-08	4.25757E-08	2.62337E-08	2.61647E-07	propane 2-monooxygenase large subunit [EC:1.14.13.227]
K18224	1.25346E-08	1.06439E-08	1.92566E-08	2.26819E-07	propane 2-monooxygenase small subunit [EC:1.14.13.227]
K18225	6.64806E-08	4.96717E-08	1.01105E-07	1.03282E-06	propane monooxygenase reductase component [EC:1.18.1.-]
K18226	1.25346E-08	0	1.92566E-08	2.26819E-07	propane monooxygenase coupling protein
K18227	3.97169E-09	0	0	0	p-cumate 2,3-dioxygenase ferredoxin reductase component [EC:1.18.1.3]
K18230	1.78113E-05	2.2999E-05	6.36818E-05	0.000105538	macrolide transport system ATP-binding/permease protein
K18231	0.000224849	0.000229122	0.000234374	0.000195354	macrolide transport system ATP-binding/permease protein
K18232	1.23271E-06	3.88984E-06	2.16232E-06	1.9947E-06	oleandomycin transport system ATP-binding protein
K18233	4.39915E-08	3.55344E-07	6.9565E-07	1.0912E-07	oleandomycin transport system permease protein
K18234	0.00022222	0.000221342	0.000214257	0.000161325	virginiamycin A acetyltransferase [EC:2.3.1.-]
K18235	0.000427679	0.000423843	0.000372628	0.00022725	virginiamycin B lyase [EC:4.2.99.-]
K18236	6.48218E-12	1.61226E-11	1.77901E-07	2.49166E-09	lincosamide nucleotidyltransferase B/F
K18237	0	3.27506E-08	0	0	ribose 1,5-bisphosphate isomerase [EC:5.3.1.29]
K18239	5.66271E-09	4.43044E-09	4.31447E-08	1.69583E-09	chorismatase [EC:3.3.2.13]
K18240	3.30955E-07	7.77914E-07	7.38097E-07	1.57584E-07	chorismate lyase / 3-hydroxybenzoate synthase [EC:4.1.3.40 4.1.3.45]
K18242	0.00021388	0.000211913	0.00018821	0.000114303	salicylate 5-hydroxylase large subunit [EC:1.14.13.172]
K18243	0.000213784	0.000211864	0.000187568	0.000113708	salicylate 5-hydroxylase small subunit [EC:1.14.13.172]
K18244	0.00044805	0.000458868	0.000503914	0.000600529	acyl-CoA dehydrogenase [EC:1.3.99.-]
K18248	2.20956E-08	4.17565E-08	1.78628E-07	1.91342E-06	anthranilate 1,2-dioxygenase ferredoxin component
K18249	2.27481E-07	1.07562E-07	1.95058E-06	2.8285E-07	anthranilate 1,2-dioxygenase ferredoxin reductase component [EC:1.18.1.-]
K18251	0	1.06439E-08	2.61643E-08	3.87201E-08	phthalate 3,4-dioxygenase subunit alpha [EC:1.14.12.-]
K18252	0	1.06439E-08	1.74429E-08	3.54228E-08	phthalate 3,4-dioxygenase subunit beta [EC:1.14.12.-]
K18253	0	2.12879E-08	1.74429E-08	2.19987E-08	phthalate 3,4-dioxygenase ferredoxin component
K18254	0	1.06439E-08	4.90336E-08	1.23417E-08	phthalate 3,4-dioxygenase ferredoxin reductase component [EC:1.18.1.3]
K18255	0	1.06439E-08	5.8993E-09	4.89881E-08	phthalate 3,4-cis-dihydrodiol dehydrogenase [EC:1.3.1.-]
K18256	0	1.06439E-08	1.74429E-08	2.51072E-08	3,4-dihydroxyphthalate decarboxylase [EC:4.1.1.69]
K18257	0	1.06439E-08	0	1.06931E-08	cis-3,4-dihydrophenanthrene-3,4-diol dehydrogenase [EC:1.3.1.49]
K18258	0	3.53772E-09	4.41886E-08	1.78604E-08	thiomorpholine-carboxylate dehydrogenase [EC:1.5.1.25]
K18275	3.51456E-10	4.92483E-08	1.72429E-07	1.92912E-08	2-formylbenzoate dehydrogenase [EC:1.2.1.78]
K18277	1.93267E-07	7.65097E-07	3.21393E-07	3.97045E-07	trimethylamine monooxygenase [EC:1.14.13.148]
K18282	3.99576E-09	7.2529E-07	7.25288E-09	0	cyanide dihydratase [EC:3.5.5.-]
K18284	3.11287E-08	2.4421E-08	3.1605E-07	1.32399E-08	adenosylhomocysteine/aminodeoxyfutalosine nucleosidase [EC:3.2.2.9 3.2.2.30]
K18285	8.46423E-08	9.5208E-10	9.17034E-07	3.9919E-08	aminodeoxyfutalosine synthase [EC:2.5.1.120]
K18286	4.18038E-07	3.79199E-07	1.03857E-06	3.51421E-06	aminodeoxyfutalosine deaminase [EC:3.5.4.40]
K18287	6.74025E-10	8.61934E-10	1.51634E-07	0	2-amino-4,5-dihydroxy-6-oxo-7-(phosphooxy)heptanoate synthase [EC:4.1.2.56]
K18288	2.3016E-06	1.84113E-06	1.04709E-05	1.80045E-06	itaconate CoA-transferase [EC:2.8.3.-]
K18289	4.42868E-06	9.79952E-06	3.03834E-05	7.28573E-05	itaconate CoA-transferase [EC:2.8.3.- 2.8.3.22]
K18290	1.32395E-06	3.35776E-06	1.52489E-06	2.07876E-06	itaconyl-CoA hydratase [EC:4.2.1.56]
K18291	5.49936E-07	7.57125E-07	6.63978E-06	1.61137E-07	itaconyl-CoA hydratase / mesaconyl-C4 CoA hydratase [EC:4.2.1.56 4.2.1.-]
K18292	1.22499E-06	6.21896E-07	2.19491E-06	2.52922E-07	(S)-citramalyl-CoA lyase [EC:4.1.3.25]
K18293	4.14044E-09	1.41037E-08	2.59514E-08	0	p-cymene methyl-monooxygenase electron transfer component [EC:1.18.1.3]
K18294	1.60476E-06	3.19886E-06	1.50404E-05	6.07114E-05	TetR/AcrR family transcriptional regulator, mexCD-oprJ operon repressor
K18295	0.00021559	0.000215038	0.000201077	0.000163212	membrane fusion protein, multidrug efflux system
K18296	0.000220705	0.000221601	0.000224589	0.000288227	multidrug efflux pump
K18297	0.001076745	0.001070951	0.000989442	0.000721309	LysR family transcriptional regulator, mexEF-oprN operon transcriptional activator
K18298	2.20674E-06	1.56364E-06	3.11749E-06	3.01852E-05	membrane fusion protein, multidrug efflux system
K18299	0.000225675	0.000225997	0.00023837	0.000313138	multidrug efflux pump
K18300	0.00043508	0.000431858	0.00040728	0.00043428	outer membrane protein, multidrug efflux system
K18301	1.74357E-06	2.03077E-06	4.23848E-06	5.85229E-05	TetR/AcrR family transcriptional regulator, mexJK operon transcriptional repressor
K18302	3.78361E-06	4.6593E-06	1.89354E-05	0.000137896	membrane fusion protein, multidrug efflux system
K18303	0.000220004	0.000219134	0.000217689	0.000230626	multidrug efflux pump
K18304	8.37964E-08	7.49347E-08	9.58375E-07	8.00558E-07	LuxR family transcriptional regulator, quorum-sensing system regulator LasR
K18305	3.48368E-07	3.76685E-07	9.80969E-07	2.52579E-05	transmembrane protein

K18306	5.97841E-07	1.68285E-06	4.67701E-07	8.31317E-07	membrane fusion protein, multidrug efflux system
K18307	3.65226E-06	6.91138E-06	2.03278E-05	5.08133E-05	multidrug efflux pump
K18308	1.33787E-06	1.16575E-06	3.33952E-06	5.49096E-05	outer membrane protein, multidrug efflux system
K18310	0	0	1.47932E-08	0	beta-citrylglutamate/N-acetylaspartylglutamate synthase [EC:6.3.1.17 6.3.2.41]
K18313	7.2045E-07	1.42677E-06	5.83059E-06	1.40178E-05	succinyl-CoA---D-citramalate CoA-transferase [EC:2.8.3.20]
K18314	0	0	1.48079E-07	0	(R)-citramalyl-CoA lyase [EC:4.1.3.46]
K18320	2.32016E-06	2.09376E-06	5.05879E-06	5.74072E-06	transposase, IS6 family
K18321	5.46737E-07	3.96405E-07	1.21913E-06	3.41398E-05	membrane fusion protein, multidrug efflux system
K18322	0.00021931	0.000219415	0.000220125	0.000225216	multidrug efflux pump
K18323	5.29367E-07	3.27281E-07	3.84039E-06	3.56092E-05	outer membrane protein, multidrug efflux system
K18324	0.000436538	0.000439015	0.000427801	0.000483135	multidrug efflux pump
K18325	2.0538E-07	6.83354E-07	1.39166E-07	7.73937E-07	AraC family of transcriptional regulator, multidrug resistance transcriptional activator
K18326	0.000218852	0.000217255	0.00020687	0.00020945	MFS transporter, DHA2 family, multidrug resistance protein
K18330	9.74027E-08	4.1154E-07	6.49244E-07	1.38297E-07	NADP-reducing hydrogenase subunit HndA [EC:1.12.1.3]
K18331	1.65834E-06	8.62109E-07	1.58851E-06	3.55959E-07	NADP-reducing hydrogenase subunit HndC [EC:1.12.1.3]
K18332	4.19363E-07	7.06287E-07	1.97509E-06	6.35133E-07	NADP-reducing hydrogenase subunit HndD [EC:1.12.1.3]
K18333	4.11929E-06	7.1393E-06	3.14424E-05	0.00011401	L-fucose dehydrogenase
K18334	6.92121E-07	1.10517E-06	4.18651E-06	9.32506E-06	L-fuconate dehydratase [EC:4.2.1.68]
K18335	0.000220078	0.000223283	0.000234149	0.000240857	2-keto-3-deoxy-L-fuconate dehydrogenase [EC:1.1.1.-]
K18336	3.56013E-06	5.10451E-06	2.39231E-05	3.80575E-05	2,4-diketo-3-deoxy-L-fuconate hydrolase [EC:3.7.1.-]
K18337	5.54657E-06	1.01125E-06	6.66486E-06	5.46324E-06	L-rhamnose 1-dehydrogenase [EC:1.1.1.378 1.1.1.377 1.1.1.173]
K18344	1.49643E-07	4.93902E-07	2.04993E-06	1.05983E-05	two-component system, OmpR family, response regulator VanR
K18345	1.055E-06	1.25043E-07	1.04226E-06	2.31528E-07	two-component system, OmpR family, sensor histidine kinase VanS [EC:2.7.13.3]
K18346	3.97405E-07	1.4978E-07	1.60552E-06	4.02116E-07	vancomycin resistance protein VanW
K18347	1.6529E-07	1.07794E-07	3.42872E-07	2.24294E-07	D-specific alpha-keto acid dehydrogenase [EC:1.1.1.-]
K18348	3.44301E-07	1.55778E-07	3.92127E-07	1.50362E-08	serine/alanine racemase [EC:5.1.1.18 5.1.1.1]
K18349	5.57023E-06	9.71703E-06	2.6298E-05	4.92443E-05	two-component system, OmpR family, response regulator VanR
K18350	1.28591E-06	5.3507E-07	3.50427E-06	3.02341E-06	two-component system, OmpR family, sensor histidine kinase VanS [EC:2.7.13.3]
K18351	2.09452E-06	4.72973E-06	2.09366E-05	5.06465E-05	two-component system, OmpR family, sensor histidine kinase VanS [EC:2.7.13.3]
K18352	5.29607E-07	8.73335E-07	1.86898E-06	5.48639E-07	two-component system, OmpR family, response regulator VanR
K18353	0	0	1.8852E-07	1.45748E-08	vancomycin resistance protein VanJ
K18355	0	0	0	4.01858E-09	phenylglyoxylate dehydrogenase alpha subunit [EC:1.2.1.58]
K18356	0	0	0	4.01858E-09	phenylglyoxylate dehydrogenase beta subunit [EC:1.2.1.58]
K18357	0	0	0	4.01858E-09	phenylglyoxylate dehydrogenase gamma subunit [EC:1.2.1.58]
K18358	0	0	0	4.01858E-09	phenylglyoxylate dehydrogenase delta subunit [EC:1.2.1.58]
K18359	0	0	2.22133E-08	4.01858E-09	phenylglyoxylate dehydrogenase epsilon subunit [EC:1.2.1.58]
K18360	0	0	0	4.01858E-09	phenylacetyl-CoA:acceptor oxidoreductase accessory protein
K18361	0.000213765	0.000211774	0.000186317	0.000113229	phenylacetyl-CoA:acceptor oxidoreductase [EC:1.17.5.1 3.1.2.25]
K18362	0	0	0	9.733E-09	phenylacetyl-CoA:acceptor oxidoreductase 27-kDa subunit
K18363	0	0	0	9.733E-09	phenylacetyl-CoA:acceptor oxidoreductase 26-kDa subunit
K18364	1.01809E-07	6.14809E-07	2.06745E-06	8.58968E-07	2-oxopent-4-enoate/cis-2-oxohex-4-enoate hydratase [EC:4.2.1.80 4.2.1.132]
K18365	1.18173E-07	6.54835E-07	1.38758E-06	2.41841E-06	4-hydroxy-2-oxovalerate/4-hydroxy-2-oxohexanoate aldolase [EC:4.1.3.39 4.1.3.43]
K18366	0.000213923	0.000212634	0.00018791	0.000119309	acetaldehyde/propanal dehydrogenase [EC:1.2.1.10 1.2.1.87]
K18367	2.21436E-07	4.05048E-08	1.84219E-06	1.49458E-06	CoA-dependent NAD(P)H sulfur oxidoreductase [EC:1.8.1.18]
K18369	5.43594E-06	5.61144E-06	1.43641E-05	2.19009E-05	alcohol dehydrogenase [EC:1.1.1.-]
K18370	0	0	1.44647E-08	2.85261E-08	alcohol dehydrogenase [EC:1.1.1.-]
K18371	1.13646E-07	1.99512E-08	4.15574E-07	2.72423E-06	acetone monooxygenase (methyl acetate-forming) [EC:1.14.13.226]
K18372	6.26307E-07	2.25434E-07	4.82827E-07	1.41506E-05	methyl acetate hydrolase [EC:3.1.1.-]
K18373	0.000213777	0.000211805	0.000188393	0.000113321	type III secretion protein HrpB1
K18374	0.000213774	0.000211798	0.000186535	0.000113229	type III secretion inner rod protein HrpB2
K18376	0.000427531	0.000423552	0.000372354	0.000226411	type III secretion translocon protein HrpF
K18377	0.000213802	0.000211843	0.000186209	0.000113211	lysozyme-related protein Hpa2
K18379	0.000213766	0.000211783	0.000186142	0.000113202	type III secretion regulatory protein HpaA
K18380	0.000213774	0.000211798	0.00018876	0.000113361	type III secretion control protein HpaB
K18381	0.000213773	0.000211797	0.000186535	0.000113229	type III secretion control protein HpaP
K18382	2.80672E-07	4.00107E-07	1.01609E-06	9.36836E-07	NAD+-dependent secondary alcohol dehydrogenase Adh1 [EC:1.1.1.-]
K18383	0.000216626	0.000218415	0.000218441	0.00019004	trans-feruloyl-CoA hydratase / vanillin synthase [EC:4.2.1.101 4.1.2.41]
K18399	0	0	3.2626E-08	0	valacyclovir hydrolase [EC:3.1.-.-]
K18425	9.73738E-07	2.71225E-09	1.07308E-07	5.29605E-08	3-hydroxy-D-aspartate aldolase [EC:4.1.3.41]
K18427	0	0	0	8.47917E-10	4-hydroxyphenylacetate decarboxylase large subunit [EC:4.1.1.83]
K18429	6.9966E-08	1.50022E-07	6.1521E-07	2.14059E-06	GDP/UDP-N,N-diacetylbacillosamine 2-epimerase (hydrolysing) [EC:3.2.1.184]
K18430	4.28018E-07	8.01172E-07	4.26576E-06	1.41005E-05	N,N-diacetyllegionaminate synthase [EC:2.5.1.101]
K18431	1.8391E-07	2.31957E-07	7.7492E-07	3.64398E-06	CMP-N,N-diacetyllegionaminic acid synthase [EC:2.7.7.82]
K18433	0	0	5.86472E-09	0	type IV secretion system T-DNA border endonuclease VirD1
K18434	0	0	5.86472E-09	0	type IV secretion system T-DNA border endonuclease VirD2
K18444	9.086E-08	2.28616E-08	4.11122E-07	1.67514E-06	two-component system, glycerol uptake and utilization response regulator
K18445	2.9516E-08	5.16394E-08	4.39869E-07	2.23002E-08	diadenosine hexaphosphate hydrolase (ATP-forming) [EC:3.6.1.61]
K18446	0.000214056	0.000212677	0.000190028	0.00013126	triphosphatase [EC:3.6.1.25]
K18454	0	0	6.34367E-08	2.40116E-07	chitin disaccharide deacetylase [EC:3.5.1.105]
K18455	1.08238E-06	4.43288E-06	5.19589E-06	3.46245E-06	mycothiol S-conjugate amidase [EC:3.5.1.115]
K18456	0.000218281	0.00021599	0.000202373	0.000155748	8-oxoguanine deaminase [EC:3.5.4.32]
K18457	1.66031E-07	9.32555E-08	1.3623E-06	1.96336E-06	L-proline amide hydrolase [EC:3.5.1.101]
K18459	2.0403E-06	5.92863E-06	1.25588E-05	2.79303E-05	guanidinopropionase [EC:3.5.3.17]
K18471	5.69844E-07	9.28805E-07	1.70353E-06	4.17278E-07	methylglyoxal reductase [EC:1.1.1.-]
K18472	2.4166E-06	5.92953E-06	9.9657E-06	3.85292E-06	acetyl-CoA/propionyl-CoA carboxylase carboxyl transferase subunit [EC:6.4.1.2 6.4.1.3 2.1.3.15]
K18473	2.04795E-08	0	1.11571E-06	3.88528E-06	acetoacetyl-[acyl-carrier protein] synthase [EC:2.3.1.180]
K18474	1.11147E-07	7.94201E-08	2.13924E-07	2.81908E-06	trans-2-decenoyl-[acyl-carrier protein] isomerase [EC:5.3.3.14]
K18475	5.91541E-07	3.68295E-07	4.76705E-07	2.39714E-06	lysine-N-methylase [EC:2.1.1.-]
K18476	8.97675E-07	7.77397E-07	7.17951E-06	4.68237E-06	TetR/AcrR family transcriptional regulator, tetracycline repressor protein
K18478	4.3316E-07	4.27013E-07	5.35932E-07	2.18211E-06	sulfofructose kinase [EC:2.7.1.184]
K18479	3.00107E-07	4.86978E-07	9.65904E-07	2.13126E-06	sulfoquinovose isomerase [EC:5.3.1.31]
K18480	0.00021785	0.000217384	0.000209023	0.000209696	cholesterol transport system auxiliary component
K18481	0	1.48025E-06	8.73215E-07	1.53879E-05	Mce-associated membrane protein
K18500	0	0	2.09314E-08	0	heterodisulfide reductase cytochrome b-like subunit
K18501	0	0	2.09314E-08	0	heterodisulfide reductase iron-sulfur subunit
K18530	3.16722E-07	4.4175E-07	1.14159E-06	7.04844E-07	putative aminopeptidase FrvX [EC:3.4.11.-]
K18531	3.88705E-09	1.2943E-08	3.49694E-08	2.17297E-07	putative frv operon regulatory protein

K18532	0	9.27737E-10	2.86391E-08	0	adenylate kinase [EC:2.7.4.3]
K18534	1.31581E-08	1.21247E-08	2.57424E-08	4.15646E-09	MPBQ/MSBQ methyltransferase [EC:2.1.1.295]
K18540	1.11617E-06	2.37113E-06	1.10902E-05	3.10996E-05	(R)-amidase [EC:3.5.1.100]
K18544	0	0	1.99347E-09	0	streptogrisin A [EC:3.4.21.80]
K18545	0	0	2.82857E-07	1.16092E-08	streptogrisin B [EC:3.4.21.81]
K18546	2.02208E-10	3.19986E-07	3.11232E-06	1.73013E-07	streptogrisin C [EC:3.4.21.-]
K18547	0	0	4.25637E-07	8.93018E-09	streptogrisin D [EC:3.4.21.-]
K18548	0	0	3.48857E-09	0	glutamyl endopeptidase II [EC:3.4.21.82]
K18552	4.97916E-07	1.73139E-07	1.75857E-06	1.46904E-05	MFS transporter, DHA1 family, florfenicol/chloramphenicol resistance protein
K18553	2.36751E-06	3.67329E-06	3.63596E-06	5.58637E-06	MFS transporter, DHA1 family, chloramphenicol resistance protein
K18554	3.07564E-07	2.8528E-07	3.53182E-07	1.56441E-08	chloramphenicol 3-O phosphotransferase [EC:2.7.1.-]
K18555	9.90368E-07	7.54699E-07	1.39617E-06	5.15047E-05	fluoroquinolone resistance protein
K18556	4.17028E-08	0	2.86743E-08	0	NADH-dependent fumarate reductase subunit A [EC:1.3.1.6]
K18559	0	0	5.73412E-08	0	NADH-dependent fumarate reductase subunit D
K18560	0	0	2.2895E-07	0	NADH-dependent fumarate reductase subunit E
K18567	2.07763E-06	3.86481E-08	3.05713E-06	6.44386E-08	MFS transporter, DHA1 family, purine base/nucleoside efflux pump
K18572	1.23156E-06	1.74348E-06	6.65453E-06	8.88596E-06	putative pantetheine hydrolase [EC:3.5.1.-]
K18574	0	0	2.00271E-07	5.54947E-09	Xaa-Xaa-Pro tripeptidyl-peptidase [EC:3.4.14.12]
K18579	0	0	5.3103E-08	0	galactan endo-1,6-beta-galactosidase [EC:3.2.1.164]
K18581	1.20841E-06	3.65514E-06	1.62339E-06	1.87284E-06	unsaturated chondroitin disaccharide hydrolase [EC:3.2.1.180]
K18586	1.00952E-07	1.76707E-08	2.64316E-07	7.93223E-08	ubiquinone biosynthesis protein COQ4
K18587	5.1365E-07	1.02236E-07	4.25009E-07	1.2584E-07	ubiquinone biosynthesis protein COQ9
K18588	0.000216994	0.000215579	0.000204644	0.000157918	coenzyme Q-binding protein COQ10
K18589	9.75567E-10	3.5762E-09	1.72241E-08	1.18161E-07	dihydrofolate reductase (trimethoprim resistance protein) [EC:1.5.1.3]
K18590	1.22611E-08	6.79163E-10	1.96643E-09	3.08685E-08	dihydrofolate reductase (trimethoprim resistance protein) [EC:1.5.1.3]
K18593	3.17865E-07	7.8919E-09	9.30422E-08	3.4733E-08	4-hydroxybutyryl-CoA synthetase (ADP-forming) [EC:6.2.1.-]
K18594	1.2908E-08	8.19722E-09	1.41102E-07	4.55064E-08	3-hydroxypropionyl-CoA synthetase (ADP-forming) [EC:6.2.1.-]
K18601	5.39461E-08	2.83838E-08	4.62067E-08	5.65278E-09	aldehyde dehydrogenase [EC:1.2.1.-]
K18603	9.207E-07	2.77426E-06	5.2996E-07	1.33653E-06	acetyl-CoA/propionyl-CoA carboxylase [EC:6.4.1.2 6.4.1.3]
K18604	1.52453E-07	1.11198E-06	3.37794E-06	1.45334E-07	acetyl-CoA/propionyl-CoA carboxylase [EC:6.4.1.2 6.4.1.3 2.1.3.15]
K18605	0	1.02051E-08	6.17968E-08	0	biotin carboxyl carrier protein
K18607	0	3.8155E-08	2.90714E-08	0	pyridoxine 4-oxidase [EC:1.1.3.12]
K18608	0	3.8155E-08	2.90714E-08	0	pyridoxamine---pyruvate transaminase [EC:2.6.1.30]
K18609	0	0	5.63276E-08	0	pyridoxal 4-dehydrogenase [EC:1.1.1.107]
K18610	0	0	1.1949E-07	0	4-pyridoxolactonase [EC:3.1.1.27]
K18611	1.29627E-08	5.03106E-08	1.12966E-07	0	4-pyridoxate dehydrogenase [EC:1.1.99.42]
K18612	0	0	2.90714E-08	0	5-formyl-3-hydroxy-2-methylpyridine 4-carboxylate dehydrogenase [EC:1.2.1.-]
K18613	0	1.83961E-08	9.55235E-08	0	3-hydroxy-2-methylpyridine-4,5-dicarboxylate 4-decarboxylase [EC:4.1.1.51]
K18614	0	0	2.32571E-08	0	2-(acetamidomethylene)succinate hydrolase [EC:3.5.1.29]
K18640	1.32825E-06	1.53685E-06	7.37009E-06	1.84247E-05	plasmid segregation protein ParM
K18649	1.19151E-08	2.76372E-08	4.96057E-08	1.52139E-08	inositol-phosphate phosphatase / L-galactose 1-phosphate phosphatase / histidinol-phosphatase [EC:3.1.3.25 3.1.3.93 3
K18650	0.000213911	0.000211851	0.000186274	0.000113302	exo-poly-alpha-galacturonosidase [EC:3.2.1.82]
K18652	1.02543E-06	1.16878E-08	2.02554E-07	1.06146E-08	glucose-6-phosphate 3-dehydrogenase [EC:1.1.1.361]
K18653	8.0348E-10	1.64742E-08	4.19041E-07	1.2802E-06	3-dehydro-glucose-6-phosphate---glutamate transaminase [EC:2.6.1.104]
K18654	4.51972E-08	1.65204E-08	2.23737E-07	1.28713E-08	kanosamine-6-phosphate phosphatase [EC:3.1.3.92]
K18657	9.82507E-08	3.14678E-07	3.2655E-07	1.71394E-05	cell division protein ZapC
K18660	4.40499E-09	1.08979E-08	5.29889E-07	7.795E-08	malonyl-CoA/methylmalonyl-CoA synthetase [EC:6.2.1.-]
K18661	6.98581E-06	1.34161E-05	6.33887E-05	0.000150475	malonyl-CoA/methylmalonyl-CoA synthetase [EC:6.2.1.-]
K18662	5.62941E-08	1.34585E-07	4.66068E-07	2.5556E-07	malonyl-CoA/methylmalonyl-CoA synthetase [EC:6.2.1.-]
K18672	2.67515E-06	1.76827E-06	1.03421E-05	3.74615E-06	diadenylate cyclase [EC:2.7.7.85]
K18673	1.03806E-07	1.34277E-08	1.29858E-07	9.3685E-07	beta-glucoside kinase [EC:2.7.1.85]
K18675	2.1828E-07	5.68978E-08	8.21395E-08	1.00112E-05	N,N-diacetylchitobiose phosphorylase [EC:2.4.1.280]
K18676	2.35424E-07	2.58132E-07	1.26577E-06	1.60169E-05	glucosamine kinase [EC:2.7.1.8]
K18677	0	0	1.22739E-08	1.50769E-08	galacturonokinase [EC:2.7.1.44]
K18678	3.19681E-08	0	2.00121E-08	1.90174E-09	phytol kinase [EC:2.7.1.182]
K18682	3.01481E-06	2.6321E-06	1.6978E-05	4.49907E-06	ribonucrease Y [EC:3.1.-.-]
K18686	0	0	1.81612E-08	0	cyclooctat-9-en-7-ol synthase [EC:4.2.3.146]
K18687	0.000434151	0.000440721	0.00042276	0.000363476	HIP---CoA ligase [EC:6.2.1.41]
K18688	0.000218181	0.000219285	0.000221889	0.000205218	3-oxocholest-4-en-26-oate---CoA ligase [EC:6.2.1.42]
K18691	3.93345E-06	2.84676E-06	8.20013E-06	7.00644E-05	membrane-bound lytic murein transglycosylase F [EC:4.2.2.-]
K18692	6.34324E-06	5.84505E-06	2.90572E-05	0.000115305	ATP-dependent RNA helicase CshB [EC:3.6.4.13]
K18697	1.6534E-06	1.66916E-06	2.25793E-06	5.54495E-05	phosphatidylglycerophosphatase C [EC:3.1.3.27]
K18698	6.62107E-08	3.20097E-08	2.22012E-08	4.62638E-07	beta-lactamase class A TEM [EC:3.5.2.6]
K18699	1.0351E-06	2.98807E-08	2.20487E-08	4.41674E-07	beta-lactamase class A SHV [EC:3.5.2.6]
K18700	7.16735E-07	3.79306E-07	2.61427E-07	1.97084E-06	fluoroacetyl-CoA thioesterase [EC:3.1.2.29]
K18701	1.83252E-06	6.31744E-06	2.89827E-06	6.27308E-06	arsenate-mycothiol transferase [EC:2.8.4.2]
K18702	0.000455002	0.000472552	0.000596306	0.000737266	CoA:oxalate CoA-transferase [EC:2.8.3.19]
K18704	1.1315E-06	7.36131E-07	5.71013E-06	4.41302E-07	CDP-ribitol ribitolphosphotransferase / teichoic acid ribitol-phosphate polymerase [EC:2.7.8.14 2.7.8.47]
K18707	4.33301E-06	3.41071E-06	1.47419E-05	7.54661E-07	threonylcarbamoyladenosine tRNA methylthiotransferase MtaB [EC:2.8.4.5]
K18765	9.83398E-08	3.14899E-07	3.27192E-07	1.37619E-05	RNase E specificity factor CsrD
K18766	1.66464E-06	0	5.39795E-06	2.07284E-07	beta-lactamase class A BlaZ [EC:3.5.2.6]
K18767	2.1963E-09	1.59894E-08	4.17923E-08	1.5069E-07	beta-lactamase class A CTX-M [EC:3.5.2.6]
K18768	2.87727E-08	3.08507E-08	5.0202E-08	1.92787E-06	beta-lactamase class A KPC [EC:3.5.2.6]
K18770	4.96165E-07	2.03943E-07	1.29262E-06	3.20196E-07	penicillin-binding protein 4 [EC:2.4.1.129 3.4.16.4]
K18775	9.85028E-09	2.27904E-09	6.18983E-08	2.63212E-08	levanbiose-producing levanase [EC:3.2.1.64]
K18778	0.000215287	0.000215239	0.000203553	0.0001621	cell division protein ZapD
K18779	0	0	2.58094E-09	1.60743E-08	7-cyano-7-deazaguanine tRNA-ribosyltransferase [EC:2.4.2.48]
K18780	1.77219E-09	6.54579E-09	9.00838E-09	2.33298E-08	metallo-beta-lactamase class B NDM [EC:3.5.2.6]
K18781	0	5.91896E-11	5.1602E-10	8.71284E-09	metallo-beta-lactamase class B VIM [EC:3.5.2.6]
K18782	2.79894E-09	3.76094E-07	9.80678E-10	9.62377E-10	metallo-beta-lactamase class B IMP [EC:3.5.2.6]
K18783	1.15709E-06	1.40376E-08	2.02178E-08	4.05338E-08	nigerose phosphorylase [EC:2.4.1.279]
K18785	3.52522E-07	8.83427E-07	2.85336E-06	1.41124E-06	beta-1,4-mannooligosaccharide/beta-1,4-mannosyl-N-acetylglucosamine phosphorylase [EC:2.4.1.319 2.4.1.320]
K18786	5.0699E-08	2.37366E-08	1.67677E-07	0	cellobionic acid phosphorylase [EC:2.4.1.321]
K18788	1.29644E-10	3.22451E-10	9.33616E-10	3.84032E-09	UDP-Gal:alpha-D-GlcNAc-diphosphoundecaprenol beta-1,3-galactosyltransferase [EC:2.4.1.303]
K18790	1.64255E-09	7.3731E-09	2.24087E-09	6.07486E-08	beta-lactamase class D OXA-1 [EC:3.5.2.6]
K18791	1.20339E-06	2.92879E-06	1.38794E-05	3.47622E-05	beta-lactamase class D OXA-2 [EC:3.5.2.6]
K18792	7.0238E-09	1.37042E-10	3.29568E-08	1.12882E-07	beta-lactamase class D OXA-10 [EC:3.5.2.6]

K18793	0	0	1.52269E-08	2.98446E-09	beta-lactamase class D OXA-23 [EC:3.5.2.6]
K18794	0	0	1.52269E-08	2.98446E-09	beta-lactamase class D OXA-51 [EC:3.5.2.6]
K18795	3.88931E-11	1.36748E-08	1.60152E-08	1.41556E-07	beta-lactamase class A CARB-1 [EC:3.5.2.6]
K18796	6.59563E-08	2.81019E-08	2.0369E-08	3.49076E-07	beta-lactamase class A LEN [EC:3.5.2.6]
K18798	0	0	0	1.90174E-09	peroxisome-assembly ATPase [EC:3.6.4.7]
K18800	0.000218774	0.000217777	0.000207242	0.000235861	2-polyprenylphenol 6-hydroxylase [EC:1.14.13.240]
K18802	0	0	0	1.12276E-06	glutamine amidotransferase
K18814	0	0	3.43536E-07	0	putative inorganic carbon (hco3(-)) transporter
K18815	6.48218E-12	1.61226E-11	4.66808E-11	1.04498E-08	aminoglycoside 6-N-acetyltransferase I [EC:2.3.1.82]
K18816	5.08395E-07	7.2308E-07	1.34337E-06	2.44527E-05	aminoglycoside 6-N-acetyltransferase I [EC:2.3.1.82]
K18817	1.32436E-08	2.57961E-10	1.01632E-07	3.07226E-09	hygromycin-B 4-O-kinase [EC:2.7.1.163]
K18820	5.0699E-08	7.18797E-07	3.53732E-06	4.68835E-09	oligosaccharide 4-alpha-D-glucosyltransferase [EC:2.4.1.161]
K18821	6.5896E-08	0	2.32571E-09	0	lipoyl amidotransferase [EC:2.3.1.200]
K18824	2.27634E-08	4.17262E-07	1.99493E-07	2.33389E-06	dihydropteroate synthase type 2 [EC:2.5.1.15]
K18827	2.09006E-07	4.96061E-07	7.69711E-07	3.67936E-06	O-antigen chain-terminating methyltransferase [EC:2.1.1.- 2.1.1.294 2.7.1.181]
K18828	2.13968E-06	3.78759E-06	1.64022E-05	3.53889E-05	tRNA(fMet)-specific endonuclease VapC [EC:3.1.-.-]
K18829	1.5769E-06	2.51513E-06	9.33705E-06	2.12335E-05	antitoxin VapB
K18830	4.82435E-08	7.94201E-08	6.40344E-08	1.04662E-06	HTH-type transcriptional regulator / antitoxin PezA
K18831	0.000215141	0.000213677	0.000193598	0.00013836	HTH-type transcriptional regulator / antitoxin HigA
K18833	1.2829E-08	1.6168E-08	3.00599E-07	1.18302E-07	MFS transporter, DHA3 family, multidrug efflux protein
K18837	6.21139E-08	2.63032E-07	1.11719E-07	8.34398E-07	cytoskeleton-binding toxin CbtA and related proteins
K18838	3.83345E-08	1.65642E-07	7.27602E-07	4.28021E-06	cytoskeleton bundling-enhancing protein CbeA and related proteins
K18839	4.29272E-09	1.931E-08	4.15683E-08	2.37633E-07	toxin GhoT
K18840	4.20643E-09	1.79456E-08	3.02922E-08	1.89308E-07	antitoxin GhoS
K18841	4.94855E-07	1.91216E-07	3.01158E-07	3.57847E-07	mRNA interferase ChpB [EC:3.1.-.-]
K18842	4.75343E-07	2.08908E-07	5.06383E-07	4.56517E-07	antitoxin ChpS
K18843	3.69489E-07	5.2236E-07	6.34824E-07	7.63212E-07	antitoxin HicB
K18844	7.34135E-07	4.78802E-07	1.55663E-06	5.14249E-05	spectinomycin phosphotransferase
K18845	1.48374E-09	5.82833E-09	1.09719E-09	1.2525E-08	16S rRNA (guanine(1405)-N(7))-methyltransferase [EC:2.1.1.179]
K18850	0.000217678	0.000216924	0.000206919	0.000209626	50S ribosomal protein L16 3-hydroxylase [EC:1.14.11.47]
K18851	0	2.2762E-06	6.75506E-06	1.78648E-05	diacylglycerol O-acyltransferase / trehalose O-mycolyltransferase [EC:2.3.1.20 2.3.1.122]
K18853	2.1563E-08	4.06301E-08	6.17806E-08	9.02095E-07	dihydromethanopterin reductase (acceptor) [EC:1.5.99.15]
K18855	2.00208E-06	3.14872E-06	3.25512E-06	1.06916E-06	UDP-N-acetylglucosamine 3-dehydrogenase [EC:1.1.1.374]
K18856	3.2196E-07	1.82359E-07	3.34331E-06	7.94462E-08	D-alanine---D-serine ligase [EC:6.3.2.35]
K18862	1.82543E-08	7.11274E-08	1.31457E-07	1.3093E-06	small toxic polypeptide LdrA/B/C/D
K18866	3.2196E-07	1.48827E-07	3.93329E-07	6.45943E-09	zinc D-Ala-D-Ala dipeptidase/carboxypeptidase [EC:3.4.13.22 3.4.17.14]
K18874	0	0	6.67491E-07	3.96649E-08	type III effector protein AvrRps4
K18879	2.997E-08	0	1.62027E-06	9.25515E-08	type III effector protein XopD
K18887	2.98277E-06	3.64181E-06	6.03756E-06	6.78795E-06	ATP-binding cassette, subfamily B, multidrug efflux pump
K18888	2.43218E-06	8.96854E-07	2.96709E-06	9.13345E-06	ATP-binding cassette, subfamily B, multidrug efflux pump
K18889	1.34352E-05	1.49784E-05	5.12828E-05	0.000121053	ATP-binding cassette, subfamily B, multidrug efflux pump
K18890	1.06962E-05	1.3936E-05	6.23883E-05	0.000123411	ATP-binding cassette, subfamily B, multidrug efflux pump
K18891	1.81241E-06	5.36581E-07	1.60638E-06	5.09657E-06	ATP-binding cassette, subfamily B, multidrug efflux pump
K18892	3.03108E-07	2.55768E-07	1.88158E-06	1.82981E-06	ATP-binding cassette, subfamily B, multidrug efflux pump
K18893	0.00044572	0.000439464	0.000435187	0.000448661	ATP-binding cassette, subfamily B, multidrug efflux pump
K18894	2.97877E-09	1.17924E-09	5.76367E-08	1.78604E-09	ATP-binding cassette, subfamily B, multidrug efflux pump
K18895	5.35879E-07	3.18675E-07	8.68874E-06	7.74273E-06	ATP-binding cassette, subfamily B, salmochelin/enterobactin exporter
K18896	1.52803E-08	0	2.06475E-09	3.19802E-08	glycine/sarcosine N-methyltransferase [EC:2.1.1.156]
K18897	0	5.37919E-09	7.46549E-08	2.85178E-08	sarcosine/dimethylglycine N-methyltransferase [EC:2.1.1.157]
K18898	1.35921E-07	2.15996E-08	5.73406E-08	1.9588E-07	membrane fusion protein, multidrug efflux system
K18899	2.12152E-06	3.76537E-06	1.84208E-05	7.59787E-05	multidrug efflux pump
K18900	0.00303941	0.003032833	0.00289525	0.003110941	LysR family transcriptional regulator, regulator for bpeEF and oprC
K18901	0.0004348	0.000432538	0.000406543	0.000310401	membrane fusion protein, multidrug efflux system
K18902	0.000228947	0.000231943	0.000266028	0.000361902	multidrug efflux pump
K18903	0.000648361	0.000649515	0.000625207	0.000511173	outer membrane protein, multidrug efflux system
K18904	1.51453E-06	6.41143E-07	5.43475E-07	2.08507E-06	outer membrane protein, multidrug efflux system
K18905	3.97169E-09	4.03924E-08	1.40753E-08	5.0875E-09	TetR/AcrR family transcriptional regulator, repressor of the ameABC operon
K18906	9.36655E-07	1.33291E-08	2.77428E-06	3.7146E-08	MarR family transcriptional regulator, multiple gene regulator MgrA
K18907	2.96118E-06	7.22581E-06	3.21202E-05	7.78628E-05	GntR family transcriptional regulator, regulator for abcA and norABC
K18908	5.17996E-07	3.1874E-07	5.29607E-07	4.96875E-08	multidrug efflux pump
K18909	2.30846E-07	0	1.84228E-08	1.07162E-08	MarR family transcriptional regulator, repressor for mepA
K18910	1.24458E-06	3.19218E-06	1.45382E-05	3.50076E-05	D-psicose/D-tagatose/L-ribulose 3-epimerase [EC:5.1.3.30 5.1.3.31]
K18911	0.000218182	0.000216991	0.00020621	0.000210857	L-histidine Nalpha-methyltransferase [EC:2.1.1.44]
K18912	0.000434542	0.000429219	0.000394914	0.000322295	gamma-glutamyl hercynylcysteine S-oxide synthase [EC:1.14.99.50]
K18913	0	1.37918E-07	2.20596E-07	1.26868E-07	hercynylcysteine S-oxide lyase [EC:4.4.1.36]
K18916	1.64986E-06	4.17075E-06	1.69727E-05	4.25044E-05	phosphonate dehydrogenase [EC:1.20.1.1]
K18917	2.27155E-07	1.44991E-06	3.24626E-06	2.06083E-06	mycoredoxin [EC:1.20.4.3]
K18918	1.4893E-06	3.87966E-07	4.00988E-06	2.87457E-07	RHH-type transcriptional regulator, rel operon repressor / antitoxin RelB
K18919	2.23357E-07	8.94268E-07	1.60724E-07	5.30057E-07	protein HokC/D
K18920	1.01984E-07	3.11536E-07	9.26047E-08	5.53278E-07	protein HokA
K18921	2.67795E-08	1.70439E-08	1.69936E-08	2.1465E-07	protein HokB
K18922	3.40859E-08	4.94618E-08	7.28051E-08	5.08301E-07	protein HokE
K18923	2.94389E-07	3.39326E-07	8.29957E-07	5.85596E-06	antitoxin StbD
K18924	1.12561E-06	7.48955E-07	3.58468E-06	1.32066E-06	paired small multidrug resistance pump
K18925	1.27413E-06	7.3953E-07	3.9317E-06	1.11587E-06	paired small multidrug resistance pump
K18926	5.46585E-06	1.84712E-06	2.11137E-05	1.48145E-05	MFS transporter, DHA2 family, lincomycin resistance protein
K18927	0.00021592	0.000213675	0.000197029	0.000146958	LysR family transcriptional regulator, L-lactate utilization regulator
K18928	0.000216873	0.000217347	0.000197785	0.000120291	L-lactate dehydrogenase complex protein LldE
K18929	0.000216902	0.000217428	0.000198163	0.0001205	L-lactate dehydrogenase complex protein LldF
K18930	9.39434E-07	1.06303E-06	1.14296E-05	1.39097E-05	D-lactate dehydrogenase
K18931	3.52139E-07	2.34518E-07	4.67264E-07	7.53347E-07	AMP phosphorylase [EC:2.4.2.57]
K18933	0	1.17924E-09	2.50694E-08	3.74061E-07	tyrosine decarboxylase / aspartate 1-decarboxylase [EC:4.1.1.25 4.1.1.11]
K18934	3.14459E-06	3.22954E-08	9.74653E-06	1.13145E-06	MFS transporter, DHA2 family, multidrug resistance protein
K18935	9.36655E-07	0	2.75275E-06	3.03626E-08	MFS transporter, DHA2 family, multidrug resistance protein
K18936	1.54004E-06	8.0832E-08	4.52924E-06	7.31068E-07	MFS transporter, DHA2 family, multidrug resistance protein
K18937	0	1.13298E-07	1.54857E-08	0	TetR/AcrR family transcriptional regulator, repressor for lfrA
K18938	0	0	8.78778E-08	0	TetR/AcrR family transcriptional regulator, repressor for qacA

K18939	0.000215234	0.000212729	0.000189779	0.000172638	TetR/AcrR family transcriptional regulator, lmrAB and yxaGH operons repressor
K18940	2.17176E-06	9.94158E-07	4.93231E-06	1.66024E-06	two-component system, OmpR family, sensor histidine kinase ArlS [EC:2.7.13.3]
K18941	2.82291E-06	1.89707E-06	7.4709E-06	9.76108E-06	two-component system, OmpR family, response regulator ArlR
K18954	4.26862E-07	1.77548E-06	3.22159E-06	1.75025E-06	AraC family transcriptional regulator, transcriptional activator of pobA
K18955	3.28269E-06	9.88848E-06	2.54475E-05	1.97842E-05	WhiB family transcriptional regulator, redox-sensing transcriptional regulator
K18956	0	0	0	2.14324E-08	WhiB family transcriptional regulator, redox-sensing transcriptional regulator
K18957	0	2.69171E-07	3.09713E-08	2.96042E-08	WhiB family transcriptional regulator, redox-sensing transcriptional regulator
K18958	1.1642E-07	7.6013E-07	5.05132E-07	1.93954E-06	WhiB family transcriptional regulator, redox-sensing transcriptional regulator
K18967	0.000454816	0.000462863	0.000556718	0.00093422	diguanylate cyclase [EC:2.7.7.65]
K18968	0.001084573	0.001077286	0.001005513	0.001028539	diguanylate cyclase [EC:2.7.7.65]
K18970	0	0	0	1.45357E-09	beta-lactamase class A GES [EC:3.5.2.6]
K18971	0	0	1.52269E-08	1.53089E-09	beta-lactamase class D OXA-24 [EC:3.5.2.6]
K18973	2.04795E-08	0	1.8409E-08	1.22471E-08	beta-lactamase class D OXA-50 [EC:3.5.2.6]
K18974	3.22204E-07	4.53532E-07	5.76354E-07	2.22152E-06	dihydropteroate synthase type 1 [EC:2.5.1.15]
K18975	1.32188E-08	3.70113E-07	9.41211E-09	1.60312E-07	small multidrug resistance pump
K18976	2.59287E-11	6.44902E-11	1.86723E-10	1.37007E-07	beta-lactamase class D OXA-48 [EC:3.5.2.6]
K18978	3.15342E-08	5.30933E-07	4.82216E-07	2.01257E-07	glyceraldehyde-3-phosphate dehydrogenase [NAD(P)+] [EC:1.2.1.90]
K18979	0.000220447	0.000218777	0.000213819	0.000220564	epoxyqueuosine reductase [EC:1.17.99.6]
K18981	0.000429486	0.000425005	0.000380268	0.0002272	uronate dehydrogenase [EC:1.1.1.203]
K18982	3.22033E-06	7.22614E-06	3.71338E-05	9.45511E-05	D-galactarolactone isomerase [EC:5.4.1.4]
K18983	1.16488E-06	1.62704E-06	7.84007E-06	2.27237E-05	D-galactarolactone cycloisomerase [EC:5.5.1.27]
K18984	2.83595E-12	7.50469E-09	2.04229E-11	5.323E-09	secretion system chaperone SsaE
K18986	4.51331E-07	1.48827E-07	1.13803E-06	3.10859E-07	two-component system, OmpR family, sensor kinase Ihk [EC:2.7.13.3]
K18987	6.45921E-08	1.10697E-07	5.74867E-07	1.67406E-06	two-component system, OmpR family, response regulator Irr
K18988	1.37461E-07	3.78422E-07	1.93446E-07	2.48656E-06	serine-type D-Ala-D-Ala carboxypeptidase/endopeptidase [EC:3.4.16.4 3.4.21.-]
K18989	7.73096E-07	1.26955E-06	3.56007E-06	6.85408E-05	multidrug efflux pump
K18990	1.1988E-08	2.699E-07	1.24735E-06	8.85373E-06	membrane fusion protein, multidrug efflux system
K18991	1.79047E-06	8.6984E-07	4.27645E-06	1.59419E-05	AraC family transcriptional regulator, activator of mtrCDE
K18992	0	0	1.31335E-07	7.97668E-07	TetR/AcrR family transcriptional regulator, cmeABC operon repressor
K18996	1.35195E-06	3.40033E-07	3.81248E-06	1.3564E-07	replication initiation protein RepC
K18997	2.75504E-06	3.44797E-06	1.50201E-05	3.57894E-05	chaperone modulatory protein CbpM
K19000	3.42104E-07	4.60104E-07	2.03869E-06	3.50592E-05	Rho-binding antiterminator
K19002	2.75301E-06	1.45932E-06	1.44059E-05	1.45385E-05	1,2-diacylglycerol 3-alpha-glucosyltransferase [EC:2.4.1.337]
K19003	2.46011E-08	8.42237E-08	3.4108E-07	1.01177E-06	1,2-diacylglycerol 3-beta-glucosyltransferase [EC:2.4.1.336]
K19005	1.65943E-06	3.42136E-07	9.4213E-06	4.14967E-06	lipoteichoic acid synthase [EC:2.7.8.20]
K19033	1.06414E-06	1.99492E-06	1.89046E-06	5.14472E-05	30S ribosomal protein S31
K19046	3.68524E-07	2.62743E-07	5.93453E-06	1.32082E-06	CRISPR system Cascade subunit CasB
K19047	6.30392E-10	9.05126E-09	1.467E-08	1.00688E-07	TetR/AcrR family transcriptional regulator, repressor of tetCD
K19048	8.48543E-07	4.51575E-08	1.12531E-07	7.19136E-07	toxic protein SymE
K19049	8.63685E-09	8.02338E-07	1.73669E-07	7.2323E-08	chondroitin AC lyase [EC:4.2.2.5]
K19050	0	0	1.0714E-07	1.53121E-08	heparin lyase [EC:4.2.2.7]
K19051	0	0	4.01019E-08	0	heparin/heparan-sulfate lyase [EC:4.2.2.7 4.2.2.8]
K19052	0	0	2.07304E-07	3.06242E-08	heparan-sulfate lyase [EC:4.2.2.8]
K19055	0.000216327	0.000215918	0.000205362	0.000165847	Ala-tRNA(Pro) deacylase [EC:3.1.1.-]
K19056	7.03765E-08	2.90254E-07	3.38213E-08	8.99895E-08	AraC family transcriptional regulator, transposon Tn10 TetD protein
K19057	1.07451E-07	9.39627E-08	5.70379E-07	5.21229E-06	MerR family transcriptional regulator, mercuric resistance operon regulatory protein
K19058	1.41741E-07	2.77917E-07	9.00377E-07	7.2303E-06	mercuric ion transport protein
K19059	1.05867E-07	9.40333E-08	5.66754E-07	5.08895E-06	mercuric ion transport protein
K19062	5.73598E-07	3.96728E-07	1.18557E-06	2.91756E-05	rifampin ADP-ribosylating transferase
K19064	1.15208E-07	2.12674E-07	3.64681E-07	1.0175E-08	lysine 6-dehydrogenase [EC:1.4.1.18]
K19065	0	0	4.42447E-09	0	3-hydroxybenzoate 4-monooxygenase [EC:1.14.13.23]
K19066	6.53306E-07	1.78429E-06	8.12016E-06	2.35875E-06	cyclohex-1-ene-1-carbonyl-CoA dehydrogenase [EC:1.3.8.10]
K19067	0.000213769	0.000212117	0.000189021	0.000114928	cyclohexane-1-carbonyl-CoA dehydrogenase [EC:1.3.8.11]
K19068	1.12999E-07	1.32481E-06	7.51337E-07	1.12896E-06	UDP-2-acetamido-2,6-beta-L-arabino-hexul-4-ose reductase [EC:1.1.1.367]
K19072	0	0	0	3.48277E-08	oxalate oxidoreductase subunit delta [EC:1.2.7.10]
K19073	9.69047E-07	1.53301E-09	9.67853E-10	9.53907E-10	divinyl chlorophyllide a 8-vinyl-reductase [EC:1.3.1.75]
K19075	1.45261E-07	2.63798E-07	3.11642E-07	2.13628E-08	CRISPR-associated protein Cst2
K19076	4.22178E-07	4.17205E-08	3.23244E-07	4.67412E-07	CRISPR-associated protein Cmr2
K19077	9.36655E-07	0	2.92763E-06	3.03626E-08	two-component system, OmpR family, sensor histidine kinase GraS [EC:2.7.13.3]
K19078	1.14848E-06	1.0043E-07	6.51999E-06	1.23736E-06	two-component system, OmpR family, response regulator protein GraR
K19079	1.96557E-06	1.29355E-08	6.92675E-06	2.75432E-07	cationic antimicrobial peptide transport system ATP-binding protein
K19080	1.85122E-06	0	5.49902E-06	6.07252E-08	cationic antimicrobial peptide transport system permease protein
K19081	1.42163E-06	3.38033E-07	3.3739E-06	2.43169E-06	two-component system, OmpR family, sensor histidine kinase BraS/BceS [EC:2.7.13.3]
K19082	1.55087E-06	4.33353E-07	6.52705E-06	3.65264E-06	two-component system, OmpR family, response regulator protein BraR/BceR
K19083	1.0418E-06	1.37448E-07	5.94877E-06	2.92795E-07	bacitracin transport system ATP-binding protein
K19084	9.14568E-07	0	2.93377E-06	5.06347E-08	bacitracin transport system permease protein
K19088	1.45261E-07	2.63798E-07	5.07466E-08	1.71561E-08	CRISPR-associated protein Cst1
K19090	1.45633E-07	2.63798E-07	3.11642E-07	2.0314E-08	CRISPR-associated protein Cas5t
K19091	1.48424E-07	3.64846E-07	3.17703E-06	5.24139E-08	CRISPR-associated endoribonuclease Cas6 [EC:3.1.-.-]
K19092	2.72186E-06	5.82876E-06	2.57833E-05	9.14594E-05	toxin ParE1/3/4
K19095	3.52406E-08	1.79579E-08	5.82875E-07	3.14091E-06	beta-lactamase class C CMY-1 [EC:3.5.2.6]
K19096	1.39527E-07	2.0227E-08	4.05458E-08	6.11166E-07	beta-lactamase class C CMY-2 [EC:3.5.2.6]
K19100	4.19799E-09	1.79247E-08	3.02315E-08	1.89058E-07	beta-lactamase class C DHA [EC:3.5.2.6]
K19101	2.77301E-09	1.14452E-08	1.90114E-07	3.11701E-06	beta-lactamase class C FOX [EC:3.5.2.6]
K19113	2.88092E-07	4.07044E-07	7.10282E-07	1.51601E-05	acetyltransferase [EC:2.3.1.-]
K19114	0	7.73089E-08	5.57015E-08	4.13303E-09	CRISPR-associated protein Csh1
K19115	0	1.43775E-07	3.77858E-07	2.94188E-08	CRISPR-associated protein Csh2
K19116	0	7.73089E-08	5.57015E-08	3.92576E-09	CRISPR-associated protein Cas5h
K19117	7.72295E-07	1.41264E-06	4.07872E-06	7.21093E-06	CRISPR-associated protein Csd1
K19118	8.12792E-07	1.28381E-06	4.10912E-06	7.20966E-06	CRISPR-associated protein Csd2
K19119	7.72295E-07	1.28381E-06	4.52272E-06	7.23611E-06	CRISPR-associated protein Cas5d
K19123	4.7874E-07	3.26573E-07	6.19666E-06	1.34926E-06	CRISPR system Cascade subunit CasA
K19124	4.35649E-07	2.62979E-07	6.09116E-06	1.5106E-06	CRISPR system Cascade subunit CasC
K19125	3.38208E-07	2.62883E-07	6.08056E-06	1.51199E-06	CRISPR system Cascade subunit CasD
K19126	3.38221E-07	2.62915E-07	6.09705E-06	1.32286E-06	CRISPR system Cascade subunit CasE
K19127	1.41753E-06	2.97524E-06	1.4217E-05	3.61669E-05	CRISPR-associated protein Csy1
K19128	2.3534E-06	2.9826E-06	1.47969E-05	4.23604E-05	CRISPR-associated protein Csy2

K19129	2.35322E-06	2.98214E-06	1.47956E-05	4.16648E-05	CRISPR-associated protein Csy3
K19130	1.8194E-06	2.97831E-06	1.44751E-05	4.21427E-05	CRISPR-associated endonuclease Csy4 [EC:3.1.-.-]
K19131	0	0	2.22347E-07	1.14104E-08	CRISPR-associated protein Csb1
K19132	0	0	2.24928E-07	1.14104E-08	CRISPR-associated protein Csb2
K19133	0	0	1.96254E-07	1.14104E-08	CRISPR-associated protein Csb3
K19134	0	6.64661E-08	6.97714E-09	1.9107E-08	CRISPR-associated protein Csx10
K19135	0	0	1.39543E-08	0	CRISPR-associated protein Csx14
K19136	0	0	2.58094E-09	0	CRISPR-associated protein Csx17
K19137	4.12205E-08	0	5.87581E-08	6.92501E-07	CRISPR-associated protein Csn2
K19138	8.1673E-07	1.24033E-07	5.5004E-06	9.72073E-08	CRISPR-associated protein Csm2
K19139	8.1673E-07	1.24033E-07	5.47947E-06	9.97867E-08	CRISPR-associated protein Csm4
K19140	8.25898E-07	1.24033E-07	5.5004E-06	9.97867E-08	CRISPR-associated protein Csm5
K19141	4.22178E-07	3.06602E-09	3.02312E-07	4.70283E-07	CRISPR-associated protein Cmr5
K19142	4.22178E-07	4.17205E-08	3.02312E-07	4.70283E-07	CRISPR-associated protein Cmr6
K19147	2.21933E-06	1.25014E-06	3.10389E-06	1.6445E-05	5-methylcytosine-specific restriction enzyme subunit McrC
K19148	2.78238E-08	1.82033E-08	5.11481E-08	7.19939E-07	small toxic protein TisB
K19155	0.000215069	0.000214856	0.000197811	0.000141217	toxin YhaV [EC:3.1.-.-]
K19156	0.000215147	0.000215221	0.000198198	0.000141398	antitoxin PrlF
K19157	4.08924E-06	2.69703E-06	1.63854E-05	3.37036E-05	mRNA interferase YafQ [EC:3.1.-.-]
K19158	1.97779E-06	4.06995E-06	6.12707E-06	4.27287E-06	toxin YoeB [EC:3.1.-.-]
K19159	1.5547E-06	3.68128E-06	4.23628E-06	4.81298E-06	antitoxin YefM
K19160	2.72728E-09	1.14804E-08	3.49382E-08	1.78608E-06	mRNA interferase YafO [EC:3.1.-.-]
K19161	2.60412E-09	9.75205E-09	1.87533E-08	1.69942E-07	antitoxin YafN
K19162	9.82118E-08	3.01003E-07	7.49218E-08	7.99764E-07	hha toxicity modulator TomB
K19163	8.0001E-07	2.44813E-06	8.61286E-06	2.20766E-05	toxin CcdB
K19164	9.223E-07	2.40428E-06	8.7489E-06	2.20664E-05	antitoxin CcdA
K19165	6.90768E-08	1.40122E-07	2.93971E-07	5.70172E-07	antitoxin Phd
K19166	0.000214629	0.000213592	0.000192447	0.000128665	mRNA interferase HigB [EC:3.1.-.-]
K19167	1.37438E-07	0	1.92745E-07	5.27299E-07	protein AbiQ
K19168	1.0341E-06	1.75413E-06	2.16118E-06	3.43778E-05	toxin CptA
K19169	5.14428E-08	2.23323E-07	3.97649E-07	2.70255E-06	DNA sulfur modification protein DndB
K19170	5.27746E-08	2.69272E-07	6.94265E-07	3.69468E-06	DNA sulfur modification protein DndC
K19171	3.83944E-07	7.20344E-07	8.10891E-07	3.69062E-06	DNA sulfur modification protein DndD
K19172	3.21124E-08	2.06191E-07	2.75265E-07	2.59501E-06	DNA sulfur modification protein DndE
K19173	3.38064E-08	3.23282E-10	3.26816E-08	2.59499E-06	DNA phosphorothioation-dependent restriction protein DptF
K19174	3.38097E-08	3.31343E-10	3.2705E-08	2.59509E-06	DNA phosphorothioation-dependent restriction protein DptG
K19175	3.47211E-08	4.02343E-09	1.62283E-07	2.595E-06	DNA phosphorothioation-dependent restriction protein DptH
K19176	6.74259E-08	1.18379E-10	7.30188E-08	0	fatty acid amide hydrolase 2 [EC:3.5.1.99]
K19180	1.66453E-07	3.51316E-07	6.26145E-07	4.45113E-07	dTDP-6-deoxy-L-talose 4-dehydrogenase (NAD+) [EC:1.1.1.339]
K19181	2.42767E-06	3.21516E-06	1.89257E-05	8.0217E-05	1,5-anhydro-D-fructose reductase (1,5-anhydro-D-mannitol-forming) [EC:1.1.1.292]
K19185	4.92514E-09	9.19807E-10	3.81855E-08	3.48277E-08	6-hydroxypseudooxynicotine dehydrogenase subunit alpha [EC:1.5.99.14]
K19186	0	0	4.51626E-08	5.71279E-08	6-hydroxypseudooxynicotine dehydrogenase subunit beta [EC:1.5.99.14]
K19187	1.78878E-08	3.28342E-08	3.81855E-08	3.48277E-08	6-hydroxypseudooxynicotine dehydrogenase subunit gamma [EC:1.5.99.14]
K19188	4.92514E-09	9.19807E-10	3.81855E-08	3.48277E-08	2,6-dihydroxypseudooxynicotine hydrolase [EC:3.7.1.19]
K19189	0.000213772	0.000211924	0.000186321	0.000113313	2,6-dihydroxypyridine 3-monooxygenase [EC:1.14.13.10]
K19190	1.0872E-06	3.09705E-07	6.69078E-07	2.26338E-06	nicotine blue oxidoreductase [EC:1.1.1.328]
K19191	5.51075E-08	3.29362E-07	2.57122E-06	2.70532E-07	4-methylaminobutanoate oxidase (formaldehyde-forming) [EC:1.5.3.19]
K19199	0	0	0	8.03716E-09	histone-lysine N-methyltransferase SETD3 [EC:2.1.1.43]
K19200	2.64796E-09	1.5781E-07	1.71694E-06	1.06664E-07	isopenicillin-N N-acyltransferase like protein
K19203	5.17814E-08	4.44225E-08	3.19746E-06	4.01765E-07	pimaricinolide synthase PimS1
K19204	4.82604E-07	1.3471E-06	8.9017E-06	1.42476E-05	pimaricinolide synthase PimS2
K19205	0	0	1.35879E-06	2.38367E-08	pimaricinolide synthase PimS3
K19207	6.98097E-10	1.92889E-08	2.2021E-06	5.68097E-08	candicidin polyketide synthase FscD
K19208	5.64015E-08	4.0271E-08	1.96328E-06	7.11369E-08	candicidin polyketide synthase FscE
K19209	7.2217E-11	9.1623E-09	9.20861E-08	2.11979E-09	beta-lactamase class D OXA-42 [EC:3.5.2.6]
K19212	0	0	0	4.82131E-10	beta-lactamase class D OXA-63 [EC:3.5.2.6]
K19213	4.09239E-08	8.79714E-08	2.06421E-07	3.07413E-06	beta-lactamase class D OXA-12 [EC:3.5.2.6]
K19214	0	0	0	2.18468E-09	beta-lactamase class C ACC [EC:3.5.2.6]
K19215	8.86775E-09	2.04839E-08	8.92277E-08	6.20865E-07	beta-lactamase class C ACT/MIR [EC:3.5.2.6]
K19216	0	0	1.13561E-08	2.035E-09	metallo-beta-lactamase class B IND [EC:3.5.2.6]
K19217	3.99114E-09	1.60147E-08	9.07969E-08	2.68114E-07	beta-lactamase class A CARB-17 [EC:3.5.2.6]
K19220	1.19793E-06	2.71971E-07	4.15485E-06	5.8564E-06	peptidoglycan DL-endopeptidase CwlS [EC:3.4.-.-]
K19221	0.000217281	0.000216349	0.000213763	0.000165106	cob(I)alamin adenosyltransferase [EC:2.5.1.17]
K19222	3.83133E-06	9.70583E-06	2.10764E-05	0.000105278	1,4-dihydroxy-2-naphthoyl-CoA hydrolase [EC:3.1.2.28]
K19223	1.47299E-07	6.5465E-07	9.1779E-07	3.74076E-08	peptidoglycan DL-endopeptidase LytF [EC:3.4.-.-]
K19224	3.31397E-06	2.80636E-06	7.07421E-06	2.75018E-06	peptidoglycan DL-endopeptidase LytE [EC:3.4.-.-]
K19225	3.91283E-06	3.84778E-06	1.67346E-05	1.28022E-05	rhomboid protease GluP [EC:3.4.21.105]
K19226	3.31248E-07	9.41241E-07	4.72771E-06	1.89498E-05	cationic peptide transport system substrate-binding protein
K19227	1.44073E-07	4.91988E-07	6.98793E-07	1.75886E-05	cationic peptide transport system permease protein
K19228	3.15573E-07	3.71695E-07	2.5189E-06	1.85525E-05	cationic peptide transport system permease protein
K19229	2.89887E-07	4.18975E-07	1.15122E-06	1.84029E-05	cationic peptide transport system ATP-binding protein
K19230	4.25622E-06	5.12547E-06	2.26133E-05	7.42865E-05	cationic peptide transport system ATP-binding protein
K19231	0.000218374	0.000213613	0.000188384	0.000150499	fibronectin-binding autotransporter adhesin
K19232	4.46088E-08	2.99068E-08	1.18906E-07	3.74105E-06	hyaluronate-binding autotransporter adhesin
K19234	1.30958E-07	3.37784E-07	6.56195E-07	1.20966E-05	L,D-transpeptidase YnhG
K19235	2.20453E-07	6.0224E-07	7.25635E-07	1.27227E-05	L,D-transpeptidase YbiS
K19236	2.23067E-06	7.18044E-07	1.22551E-06	2.16776E-05	L,D-transpeptidase YcfS
K19237	0	0	0	2.16238E-08	gingipain K [EC:3.4.22.47]
K19238	2.83571E-08	1.83799E-08	3.73568E-08	2.9545E-07	signal transduction protein PmrD
K19239	2.37916E-06	3.99428E-06	1.60418E-05	8.71423E-05	predicted membrane protein
K19242	2.36585E-07	5.34171E-08	4.70966E-07	6.24027E-07	LysR family transcriptional regulator, repressor for citA
K19243	0.000213765	0.000211774	0.000186155	0.000113275	NAD+ dependent glucose-6-phosphate dehydrogenase [EC:1.1.1.388]
K19244	2.3808E-06	1.06007E-06	4.42079E-06	3.89586E-06	alanine dehydrogenase [EC:1.4.1.1]
K19246	0	2.99905E-08	5.1386E-08	2.41115E-08	arachidonate 15-lipoxygenase [EC:1.13.11.33]
K19265	5.75561E-06	6.21965E-06	2.26671E-05	8.86739E-05	L-glyceraldehyde 3-phosphate reductase [EC:1.1.1.-]
K19266	5.26396E-07	1.14413E-06	3.09141E-06	5.76564E-06	lactaldehyde dehydrogenase [EC:1.2.1.22]
K19267	0.000215008	0.000213033	0.000192633	0.000126262	NAD(P)H dehydrogenase (quinone) [EC:1.6.5.2]

K19268	9.80901E-08	1.79489E-09	2.60935E-07	5.93495E-08	methylaspartate mutase epsilon subunit [EC:5.4.99.1]
K19270	0.000227011	0.000226865	0.000256081	0.000317726	sugar-phosphatase [EC:3.1.3.23]
K19271	1.91294E-06	2.8002E-06	8.55108E-06	4.0757E-06	chloramphenicol O-acetyltransferase type A [EC:2.3.1.28]
K19272	1.3267E-07	7.19012E-09	3.91203E-07	7.28357E-07	aminoglycoside 3-phosphotransferase I [EC:2.7.1.95]
K19273	4.12703E-08	0	7.11179E-09	6.51746E-08	streptothricin acetyltransferase [EC:2.3.-.-]
K19274	2.26876E-11	5.6429E-11	1.63383E-10	6.72057E-10	aminoglycoside 3-phosphotransferase VI [EC:2.7.1.95]
K19275	1.90508E-09	8.02606E-09	9.96534E-09	4.46177E-08	aminoglycoside 3-N-acetyltransferase II [EC:2.3.1.81]
K19276	1.03715E-10	2.57961E-10	7.46893E-10	3.07226E-09	aminoglycoside 3-N-acetyltransferase IV [EC:2.3.1.81]
K19277	2.59287E-11	1.21425E-09	1.86723E-10	2.07986E-08	aminoglycoside 3-N-acetyltransferase VI [EC:2.3.1.81]
K19278	3.20407E-09	1.46333E-08	5.90098E-08	1.82309E-06	aminoglycoside 6-N-acetyltransferase Ib [EC:2.3.1.82]
K19279	0	0	0	5.11592E-10	aminoglycoside 9-adenylyltransferase [EC:2.7.7.-]
K19280	1.60209E-06	7.80339E-07	3.46967E-06	1.05984E-05	succinyl-CoA:mesaconate CoA transferase [EC:2.8.3.-]
K19285	3.49403E-06	1.38249E-06	8.12535E-06	1.92599E-05	FMN reductase (NADPH) [EC:1.5.1.38]
K19286	2.85871E-07	7.58422E-07	2.82039E-06	1.62535E-07	FMN reductase [NAD(P)H] [EC:1.5.1.39]
K19290	2.04795E-08	8.39802E-08	1.29895E-06	1.99283E-07	mannuronan synthase [EC:2.4.1.33]
K19291	2.04795E-08	4.35878E-08	1.27031E-06	1.84069E-07	mannuronan synthase [EC:2.4.1.33]
K19292	2.04795E-08	3.60378E-09	1.1331E-06	1.34962E-07	alginate biosynthesis protein AlgK
K19293	2.04795E-08	4.35878E-08	1.29895E-06	1.84069E-07	alginate biosynthesis protein AlgX
K19294	5.49711E-06	6.35204E-06	2.52771E-05	3.61628E-05	alginate O-acetyltransferase complex protein AlgI
K19295	4.51283E-06	5.9432E-06	3.12157E-05	6.95929E-05	alginate O-acetyltransferase complex protein AlgJ
K19296	2.04795E-08	3.19547E-09	1.27031E-06	1.84069E-07	alginate O-acetyltransferase complex protein AlgF
K19298	3.24109E-11	8.06128E-11	2.33404E-10	2.37321E-09	subtilase cytotoxin, subunit B
K19300	6.37492E-07	4.20864E-07	1.34864E-06	3.16344E-05	aminoglycoside 3-phosphotransferase II [EC:2.7.1.95]
K19301	3.20407E-09	1.34541E-08	5.20326E-08	1.82309E-06	aminoglycoside 6-N-acetyltransferase II [EC:2.3.1.82]
K19302	0.000231397	0.000228289	0.00026102	0.000418891	undecaprenyl-diphosphatase [EC:3.6.1.27]
K19303	1.07809E-06	1.76528E-06	3.66133E-06	6.18255E-06	murein DD-endopeptidase [EC:3.4.-.-]
K19304	0.00022457	0.000225465	0.000238681	0.000329859	murein DD-endopeptidase [EC:3.4.24.-]
K19309	9.81589E-07	4.77214E-07	2.17144E-06	2.96227E-06	bacitracin transport system ATP-binding protein
K19310	7.5852E-07	5.64957E-07	7.52947E-07	1.01341E-07	bacitracin transport system permease protein
K19311	9.69903E-07	2.1935E-07	4.70895E-07	3.53919E-06	lipase [EC:3.1.1.-]
K19312	2.93446E-06	5.63976E-06	1.42145E-05	6.8167E-07	acetyl-CoA/propionyl-CoA carboxylase carboxyl transferase subunit [EC:6.4.1.2 6.4.1.3 2.1.3.15]
K19316	2.87825E-08	2.05762E-08	6.85801E-08	1.97273E-06	beta-lactamase class A IMI [EC:3.5.2.6]
K19318	0	0	1.52269E-08	2.98446E-09	beta-lactamase class D OXA-213 [EC:3.5.2.6]
K19319	0	0	1.52269E-08	2.98446E-09	beta-lactamase class D OXA-134 [EC:3.5.2.6]
K19333	5.78573E-07	6.42239E-07	9.97962E-07	5.76179E-06	IclR family transcriptional regulator, KDG regulon repressor
K19334	3.0976E-07	2.34713E-07	2.80466E-07	6.66872E-06	biofilm protein TabA
K19335	8.74498E-07	1.33226E-07	3.15392E-06	3.59673E-06	TetR/AcrR family transcriptional regulator, repressor for divergent bdcA
K19336	4.88425E-07	3.8872E-07	1.24157E-06	1.97443E-05	cyclic-di-GMP-binding biofilm dispersal mediator protein
K19337	0.000430482	0.000428742	0.00039777	0.000278001	RpiR family transcriptional regulator, carbohydrate utilization regulator
K19338	0.000876728	0.000886493	0.000926581	0.001088389	LysR family transcriptional regulator, nitrogen assimilation regulatory protein
K19339	0.000215358	0.000212814	0.000187394	0.000114526	NosR/NirI family transcriptional regulator, nitrous oxide reductase regulator
K19340	0.000219917	0.000218559	0.000199762	0.000127008	Cu-processing system ATP-binding protein
K19341	0.000214067	0.000212504	0.000187355	0.000115554	Cu-processing system permease protein
K19342	0.000214099	0.00021271	0.000187274	0.000115601	copper chaperone NosL
K19344	4.46815E-09	9.43088E-08	9.61336E-08	3.32335E-08	cytochrome c55X
K19345	5.84142E-08	1.22744E-07	1.21622E-07	3.88863E-08	protein NirF
K19349	1.39508E-07	1.00879E-09	3.23126E-06	3.72778E-08	pleuromutilin/lincosamide/streptogramin A transport system ATP-binding/permease protein
K19350	1.641E-06	1.02123E-06	4.29652E-06	1.96892E-06	lincosamide and streptogramin A transport system ATP-binding/permease protein
K19351	7.99523E-07	0	2.68927E-06	1.86346E-06	zinc metalloproteinase [EC:3.4.24.-]
K19353	8.889E-07	1.65101E-06	8.80685E-06	1.50454E-05	heptose-I-phosphate ethanolaminephosphotransferase [EC:2.7.8.-]
K19354	1.73338E-06	2.72697E-06	2.23434E-06	2.92536E-06	heptose III glucuronosyltransferase [EC:2.4.1.-]
K19355	1.83355E-07	8.66864E-07	4.42509E-07	1.4486E-06	mannan endo-1,4-beta-mannosidase [EC:3.2.1.78]
K19367	0	2.35848E-09	1.39543E-08	0	maspardin
K19405	1.31131E-06	4.79436E-07	3.79694E-06	4.36443E-08	protein arginine kinase [EC:2.7.14.1]
K19411	1.31131E-06	4.7777E-07	3.81875E-06	1.35192E-07	protein arginine kinase activator
K19416	0.000215447	0.000215692	0.000205107	0.000166126	modulator of FtsH protease
K19417	0	0	3.8928E-07	0	HTH-type transcriptional regulator, biofilm formation regulator
K19418	6.48218E-12	1.68226E-09	5.43904E-08	1.92016E-10	membrane protein EpsK
K19419	5.54378E-08	4.42717E-08	4.15858E-07	2.47028E-09	transmembrane protein EpsG
K19421	0.000218612	0.000216207	0.000194064	0.000165794	polysaccharide biosynthesis protein EpsC
K19422	3.29618E-07	9.54111E-07	1.20429E-06	3.40349E-06	glycosyltransferase EpsD [EC:2.4.-.-]
K19423	7.64712E-07	3.7649E-07	5.41616E-07	6.19775E-07	glycosyltransferase EpsE [EC:2.4.-.-]
K19424	1.50053E-06	1.43177E-06	4.34231E-06	3.29248E-07	glycosyltransferase EpsF [EC:2.4.-.-]
K19425	1.10256E-06	1.83335E-06	2.4949E-06	3.06812E-06	glycosyltransferase EpsH [EC:2.4.-.-]
K19426	4.80567E-07	3.06602E-09	2.75406E-07	8.26982E-09	pyruvyl transferase EpsI [EC:2.-.-.-]
K19427	1.99733E-06	3.10274E-06	2.35912E-06	3.72692E-06	glycosyltransferase EpsJ [EC:2.4.-.-]
K19428	0.000219942	0.000218013	0.000193629	0.000124791	sugar transferase EpsL [EC:2.-.-.-]
K19429	2.16253E-06	2.50282E-06	9.78449E-06	2.43713E-05	acetyltransferase EpsM [EC:2.3.1.-]
K19430	2.41122E-06	1.82716E-06	2.91051E-06	2.19312E-06	pyridoxal phosphate-dependent aminotransferase EpsN [EC:2.6.1.-]
K19431	1.20682E-08	5.95088E-07	1.63051E-07	9.05104E-08	pyruvyl transferase EpsO [EC:2.-.-.-]
K19449	1.07997E-06	1.36102E-07	9.08139E-07	8.06199E-08	XRE family transcriptional regulator, master regulator for biofilm formation
K19504	4.48952E-08	7.05362E-12	2.75415E-06	7.0144E-07	glucoselysine-6-phosphate deglycase
K19505	7.47644E-08	4.64174E-07	3.79885E-06	5.72232E-06	sigma-54 dependent transcriptional regulator, gfr operon transcriptional activator
K19506	2.39789E-08	1.02071E-07	6.29203E-09	5.94218E-07	PTS system, fructoselysine/glucoselysine-specific IIA component [EC:2.7.1.-]
K19507	3.4437E-08	1.32608E-07	2.73768E-06	7.71001E-07	PTS system, fructoselysine/glucoselysine-specific IIB component [EC:2.7.1.-]
K19508	1.55785E-07	1.68408E-07	2.91833E-06	4.27363E-06	PTS system, fructoselysine/glucoselysine-specific IIC component
K19509	2.9973E-07	3.50799E-07	2.96967E-06	4.3116E-06	PTS system, fructoselysine/glucoselysine-specific IID component
K19510	3.67143E-08	8.78875E-08	3.01486E-06	2.50664E-06	fructoselysine-6-phosphate deglycase
K19511	1.23365E-08	2.03663E-08	7.86211E-08	1.07717E-07	peroxidase [EC:1.11.1.7]
K19519	4.59441E-06	3.93185E-06	1.86195E-05	8.61179E-05	transforming growth factor-beta-induced protein
K19540	3.50588E-09	1.19949E-08	1.70469E-07	1.7152E-07	fructoselysine transporter
K19542	9.127E-08	3.03022E-08	5.23187E-07	1.41184E-07	MFS transporter, DHA2 family, tetracycline/oxytetracycline resistance protein
K19545	1.78643E-07	2.63991E-07	1.17387E-07	1.04703E-07	lincosamide nucleotidyltransferase A/C/D/E
K19546	0	0	0	8.03716E-09	prephenate decarboxylase [EC:4.1.1.100]
K19548	4.71761E-07	2.52459E-07	1.85858E-06	2.08037E-06	dihydroanticapsin dehydrogenase [EC:1.1.1.385]
K19549	3.58239E-08	0	3.98755E-08	2.56735E-08	bacilysin biosynthesis transaminase BacF [EC:2.6.1.-]
K19550	3.24676E-08	0	1.17963E-07	0	bacilysin biosynthesis oxidoreductase BacG [EC:1.3.1.-]

K19551	2.31084E-09	9.31083E-09	1.66157E-06	1.32599E-07	pectate lyase C [EC:4.2.2.2 4.2.2.10]
K19563	3.42099E-09	2.44811E-07	1.08162E-07	4.27891E-09	lysine---8-amino-7-oxononanoate aminotransferase [EC:2.6.1.105]
K19569	0	0	1.76979E-09	5.4955E-10	8-demethyl-8-(2,3-dimethoxy-alpha-L-rhamnosyl)tetracenomycin-C 4-O-methyltransferase [EC:2.1.1.307]
K19572	0	0	7.39687E-08	2.035E-09	adenosine deaminase CECR1 [EC:3.5.4.4]
K19575	1.35655E-07	1.06445E-08	5.32236E-07	2.37395E-08	MerR family transcriptional regulator, activator of bmr gene
K19576	9.38363E-07	0	2.81423E-06	3.03626E-08	MFS transporter, DHA1 family, quinolone resistance protein
K19577	0.000442362	0.000442105	0.000430129	0.000550148	MFS transporter, DHA1 family, inner membrane transport protein
K19578	9.57567E-07	0	2.97054E-06	2.17896E-08	MFS transporter, DHA1 family, multidrug resistance protein
K19580	0.000213813	0.000211798	0.000186188	0.000113205	1,3,6,8-tetrahydroxynaphthalene synthase [EC:2.3.1.233]
K19585	0.000220474	0.000221665	0.000227088	0.000256183	multidrug efflux pump
K19586	4.41754E-06	4.39327E-06	1.95758E-05	9.98427E-05	membrane fusion protein, multidrug efflux system
K19587	1.07397E-07	3.23305E-07	6.33051E-08	5.1531E-07	Rrf2 family transcriptional regulator, repressor of oqxAB
K19588	9.89254E-07	5.10378E-07	1.1474E-06	2.08664E-07	2,5-dioxopentanoate dehydrogenase [EC:1.2.1.26]
K19590	7.2217E-11	9.23501E-11	1.5078E-09	1.4263E-08	D-arabinose 1-dehydrogenase [EC:1.1.1.117]
K19591	0.000661666	0.000659821	0.000642727	0.000712817	MerR family transcriptional regulator, copper efflux regulator
K19592	0.000215191	0.000213324	0.000187387	0.000144202	MerR family transcriptional regulator, gold-responsive activator of gol and ges genes
K19593	0.000215159	0.000214913	0.000199992	0.000149737	outer membrane protein, multidrug efflux system
K19594	0.000223408	0.000222888	0.000228479	0.000304004	gold/copper resistance efflux pump
K19595	2.61989E-06	3.25496E-06	1.61363E-05	3.57228E-05	membrane fusion protein, gold/copper resistance efflux system
K19597	4.19641E-06	5.27192E-06	1.63012E-05	6.65936E-05	Au+-exporting ATPase [EC:3.6.1.-]
K19609	8.35019E-07	1.05771E-06	2.78661E-06	6.76791E-06	two-component system, OmpR family, sensor histidine kinase PfeS [EC:2.7.13.3]
K19610	3.63646E-07	5.8858E-07	2.81049E-06	1.19158E-06	two-component system, OmpR family, response regulator PfeR
K19611	2.54706E-06	4.82134E-06	2.08167E-05	8.11013E-05	ferric enterobactin receptor
K19615	0	1.47448E-09	9.17879E-09	3.21442E-08	insecticidal toxin
K19616	3.64011E-07	2.53087E-07	9.29654E-07	6.7258E-06	two-component system, sensor histidine kinase and response regulator FitF [EC:2.7.13.3]
K19617	4.46815E-09	2.4421E-08	0	4.89305E-06	two-component system, response regulator FitH
K19618	5.85846E-08	2.21369E-07	4.07182E-08	3.50613E-08	LysR family transcriptional regulator, activator of insecticidal toxin
K19620	0.000213765	0.000211792	0.000186082	0.000113222	extracellular factor (EF) 3-hydroxypalmitic acid methyl ester biosynthesis protein
K19621	0.000216228	0.000212094	0.000187085	0.000141223	two-component system, sensor histidine kinase PhcS [EC:2.7.13.3]
K19622	0.000213879	0.000211832	0.00018636	0.000113322	two-component system, response regulator PhcR
K19623	0.000213775	0.000211792	0.000186082	0.000113222	two-component system, probable response regulator PhcQ
K19624	0.000213765	0.000211774	0.000186082	0.000113222	LysR family transcriptional regulator, virulence genes transcriptional regulator
K19628	0	0	0	1.78604E-09	biflaviolin synthase [EC:1.14.21.7]
K19633	0	0	1.76381E-07	1.1358E-08	sorbose reductase [EC:1.1.1.289]
K19640	8.74959E-09	2.65757E-08	2.88433E-07	1.99997E-06	putative two-component system protein, hydrogenase maturation factor HypX/HoxX
K19641	0.000225107	0.000222105	0.000220892	0.000279483	two-component system, NtrC family, response regulator HupR/HoxA
K19644	0	0	0	8.93018E-10	dihydrofolate reductase (trimethoprim resistance protein) [EC:1.5.1.3]
K19645	6.48218E-12	1.02052E-08	8.21919E-10	1.92016E-10	dihydrofolate reductase (trimethoprim resistance protein) [EC:1.5.1.3]
K19646	0	0	8.75084E-09	2.33759E-08	MFS transporter, DHA2 family, florfenicol/chloramphenicol resistance protein
K19647	0	1.02051E-08	2.75737E-10	4.70805E-08	2-hydroxymethylglutarate dehydrogenase [EC:1.1.1.291]
K19659	0	0	1.95717E-07	2.50574E-06	enoyl-CoA hydratase [EC:4.2.1.119]
K19660	0	0	0	3.42313E-09	L-arabinose 1-dehydrogenase [NAD(P)+] [EC:1.1.1.376]
K19661	1.47972E-05	1.04724E-05	4.06262E-05	0.000234199	two-component system, NtrC family, sensor histidine kinase HupT/HoxJ [EC:2.7.13.3]
K19664	1.71257E-09	4.4543E-09	3.87428E-08	5.70522E-09	CDP-2,3-bis-(O-geranylgeranyl)-sn-glycerol synthase [EC:2.7.7.67]
K19666	0.000214423	0.000212262	0.00018792	0.000160362	LuxR family transcriptional regulator, quorum-sensing system regulator SolR
K19667	0.000213765	0.000211774	0.000186082	0.000113202	transcriptional activator of eps genes
K19668	0.000427609	0.00042368	0.000373284	0.000228365	cellulose 1,4-beta-cellobiosidase [EC:3.2.1.91]
K19669	0	1.17924E-09	6.97714E-09	0	phosphonopyruvate hydrolase [EC:3.11.1.3]
K19670	3.70495E-07	9.52997E-08	1.71526E-07	8.21104E-08	phosphonoacetate hydrolase [EC:3.11.1.2]
K19686	1.51682E-06	9.62904E-07	9.88839E-07	1.90259E-07	ribonuclease VapC [EC:3.1.-.-]
K19687	1.80796E-06	7.48691E-07	9.64856E-07	1.71987E-07	antitoxin VapB
K19688	2.83668E-08	1.95539E-08	5.50586E-08	7.29857E-07	biofilm regulator BssR
K19689	2.33168E-06	1.32518E-06	5.42285E-06	3.31871E-06	aminopeptidase [EC:3.4.11.-]
K19693	0	1.35781E-08	5.47089E-07	8.40941E-06	AraC family transcriptional regulator, chitin signaling transcriptional activator
K19694	0.000216381	0.000212869	0.000190675	0.000141746	two-component system, sensor histidine kinase ChiS
K19696	1.26897E-06	1.7413E-06	3.03161E-06	5.17526E-05	5-methylthioinosine phosphorylase [EC:2.4.2.44]
K19697	1.53243E-07	2.29866E-08	1.08264E-07	9.11339E-06	propionate kinase [EC:2.7.2.15]
K19700	8.36273E-09	5.5841E-09	0	6.35938E-10	3-succinoylsemialdehyde-pyridine dehydrogenase [EC:1.2.1.83]
K19701	9.80938E-07	5.02787E-07	1.61582E-06	8.14549E-06	aminopeptidase YwaD [EC:3.4.11.6 3.4.11.10]
K19702	1.74325E-07	8.05661E-07	3.12994E-06	6.56159E-06	aminopeptidase S [EC:3.4.11.24]
K19707	2.77026E-07	2.40495E-07	3.90519E-06	6.09989E-06	sigma-B regulation protein RsbQ
K19709	6.47838E-08	1.2639E-07	6.52527E-07	3.79824E-07	acetate CoA-transferase [EC:2.8.3.8]
K19710	1.56328E-06	4.09953E-06	9.54125E-06	4.03917E-06	ATP adenylyltransferase [EC:2.7.7.53]
K19711	1.70916E-06	4.86351E-07	4.12198E-06	5.1601E-05	L-serine 3-dehydrogenase (NAD+) [EC:1.1.1.387]
K19712	0	0	4.35362E-08	0	adenosylcobinamide-phosphate guanylyltransferase [EC:2.7.7.62]
K19713	0.000436296	0.000440082	0.000442561	0.000489325	thiosulfate dehydrogenase [EC:1.8.2.2]
K19714	0	0	3.85108E-08	1.44371E-06	3-deoxy-alpha-D-manno-octulosonate 8-oxidase [EC:1.1.3.48]
K19715	1.02955E-06	5.77149E-07	1.64599E-06	1.65557E-06	8-amino-3,8-dideoxy-alpha-D-manno-octulosonate transaminase [EC:2.6.1.109]
K19720	0	0	4.35362E-08	0	collagen, type III, alpha
K19721	3.00492E-07	1.71654E-07	5.94674E-07	1.34203E-07	collagen, type V/XI/XXIV/XXVII, alpha
K19731	3.47718E-08	2.83732E-08	9.31338E-07	2.64433E-08	LuxR family transcriptional regulator, quorum-sensing system regulator CciR
K19732	2.10288E-07	4.03924E-08	9.00935E-08	0	LuxR family transcriptional regulator, activator of conjugal transfer of Ti plasmids
K19733	0	5.03892E-08	2.33103E-07	3.07201E-06	LuxR family transcriptional regulator, quorum-sensing system regulator SinR
K19734	1.3509E-06	3.49413E-06	1.52318E-05	3.97338E-05	LuxR family transcriptional regulator, quorum-sensing system regulator ExpR
K19736	2.88939E-06	1.22275E-06	1.11588E-05	6.05413E-05	TetR/AcrR family transcriptional regulator, regulator of autoinduction and epiphytic fitness
K19737	3.72955E-08	6.5978E-09	1.4951E-09	0	type II protein arginine methyltransferase [EC:2.1.1.320]
K19742	0	9.82517E-09	6.18207E-08	2.24509E-06	1-pyrroline-2-carboxylate reductase [NAD(P)H] [EC:1.5.1.49]
K19743	0.00021506	0.000214812	0.000202234	0.000148036	1-piperideine-2-carboxylate/1-pyrroline-2-carboxylate reductase [NAD(P)H] [EC:1.5.1.1]
K19744	1.45465E-08	1.25035E-08	1.5483E-07	5.71517E-08	L-arginine dehydrogenase [EC:1.4.1.25]
K19745	0.000220257	0.00021807	0.000210512	0.000275535	acrylyl-CoA reductase (NADPH) [EC:1.3.1.-]
K19746	2.57505E-06	6.05446E-06	3.15047E-05	7.02223E-05	D-arginine dehydrogenase [EC:1.4.99.6]
K19761	0	2.35848E-09	2.96529E-08	3.42565E-07	gamma-glutamylaminecyclotransferase [EC:2.3.2.-]
K19775	1.22766E-07	3.54168E-07	4.01147E-07	7.04903E-06	GntR family transcriptional regulator, hexuronate regulon transcriptional repressor
K19776	2.81205E-08	1.89412E-08	4.26301E-08	7.64343E-07	GntR family transcriptional regulator, galactonate operon transcriptional repressor
K19777	1.6084E-08	1.44566E-07	1.60784E-06	3.21126E-07	acid stress chaperone HdeA
K19778	7.08422E-08	9.91163E-08	3.41891E-08	3.97077E-07	acid stress chaperone HdeB
K19779	7.81102E-10	1.94277E-09	5.62504E-09	2.3138E-08	endodeoxyribonuclease RalR [EC:3.1.-.-]

K19780	2.40387E-09	1.01872E-08	1.73113E-08	7.4468E-08	antisense regulator of RalR protein
K19784	0.00043434	0.000435964	0.000428337	0.000424644	chromate reductase, NAD(P)H dehydrogenase (quinone)
K19789	1.64422E-06	1.48118E-06	9.43489E-06	2.4622E-05	DNA repair protein RadD
K19793	1.97874E-06	1.40828E-06	3.36074E-06	5.2013E-05	(5-formylfuran-3-yl)methyl phosphate transaminase [EC:2.6.1.108]
K19794	2.86127E-08	2.32685E-08	4.13753E-07	1.98528E-06	barbiturase [EC:3.5.2.1]
K19795	9.70665E-07	8.11318E-09	4.03754E-08	1.76118E-06	ureidomalonase [EC:3.5.1.95]
K19802	4.67048E-06	3.25182E-06	8.35114E-06	7.86772E-05	L-Ala-D/L-Glu epimerase [EC:5.1.1.20]
K19803	2.33093E-07	3.72249E-07	1.12748E-06	3.90303E-06	Kdo2-lipid A phosphotransferase [EC:2.7.4.29]
K19804	0.00021648	0.000216436	0.000205476	0.000217335	lipopolysaccharide assembly protein B
K19810	2.65092E-06	1.71541E-06	3.01841E-06	6.45425E-05	L-lysine 2,3-aminomutase [EC:5.4.3.-]
K19811	2.40723E-11	3.07834E-11	6.57526E-09	1.52139E-08	3alpha-hydroxysteroid 3-dehydrogenase [EC:1.1.1.357]
K19813	0.000214563	0.000212567	0.000186978	0.000115335	glucose dehydrogenase [EC:1.1.5.9]
K19814	1.66016E-08	5.74947E-08	2.05239E-07	1.03659E-08	glutamate 2,3-aminomutase [EC:5.4.3.9]
K19817	0	0	2.86555E-06	7.65444E-10	coenzyme F420H2 oxidase [EC:1.5.3.22]
K19818	4.92514E-09	9.19807E-10	3.81855E-08	3.48277E-08	nicotine dehydrogenase subunit A [EC:1.5.99.4]
K19819	1.39162E-08	1.09738E-09	4.17983E-08	4.01858E-08	nicotine dehydrogenase subunit B [EC:1.5.99.4]
K19820	4.92514E-09	1.09738E-09	3.97335E-08	3.48277E-08	nicotine dehydrogenase subunit C [EC:1.5.99.4]
K19824	9.77243E-07	1.19596E-06	3.11537E-06	2.71835E-08	rubrerythrin
K19826	4.92514E-09	9.19807E-10	3.81855E-08	3.48277E-08	(S)-6-hydroxynicotine oxidase [EC:1.5.3.5]
K19837	1.0677E-06	1.48756E-07	3.59375E-07	6.35604E-07	biuret amidohydrolase [EC:3.5.1.84]
K19854	1.29156E-06	1.31832E-06	3.45009E-06	1.29285E-05	dTDP-3-amino-3,6-dideoxy-alpha-D-glucopyranose transaminase [EC:2.6.1.89]
K19855	0	0	2.06475E-09	2.56554E-08	dTDP-4-dehydro-6-deoxy-D-glucose 3-epimerase [EC:5.1.3.27]
K19856	3.97169E-09	0	1.23678E-07	6.75205E-07	3-O-methyltransferase [EC:2.1.1.-]
K19883	4.60155E-08	0	6.99596E-09	1.14065E-07	bifunctional aminoglycoside 6-N-acetyltransferase / aminoglycoside 2-phosphotransferase [EC:2.3.1.82 2.7.1.190]
K19941	0	4.42717E-08	2.80217E-08	0	synapsin
K19954	0.000219217	0.000217702	0.000208407	0.000162919	alcohol dehydrogenase [EC:1.1.1.-]
K19955	2.5966E-06	2.95152E-06	1.17266E-05	1.30201E-05	alcohol dehydrogenase [EC:1.1.1.-]
K19956	1.48514E-07	2.41151E-07	2.84621E-06	5.21073E-07	L-sorbose 1-phosphate reductase [EC:1.1.1.-]
K19961	1.29627E-08	1.22641E-08	0	1.88426E-09	6-hydroxyhexanoate dehydrogenase [EC:1.1.1.258]
K19965	6.46229E-08	0	7.03844E-08	1.05618E-08	dihydroneopterin triphosphate pyrophosphohydrolase [EC:3.6.1.67]
K19966	5.95746E-07	3.71121E-07	9.46444E-07	1.24935E-07	3-sulfinopropanoyl-CoA desulfinase [EC:3.13.1.4]
K19967	2.4877E-06	2.98104E-06	1.61965E-05	3.48565E-05	D-threonine aldolase [EC:4.1.2.42]
K19969	0	5.41496E-09	8.9741E-08	1.30882E-06	2-epi-5-epi-valiolone synthase [EC:4.2.3.152]
K19971	1.25083E-07	7.94201E-08	2.29408E-07	3.5006E-06	manganese/zinc transport system substrate-binding protein
K19972	6.24792E-08	7.99328E-08	2.28759E-07	2.90395E-06	manganese/zinc transport system permease protein
K19973	3.84242E-06	3.91083E-06	1.88278E-05	4.29099E-05	manganese/zinc transport system ATP-binding protein [EC:3.6.3.35]
K19974	0	0	1.32269E-08	0	2-epi-5-epi-valiolone 7-phosphate 2-epimerase [EC:5.1.3.35]
K19975	1.17458E-06	2.39212E-07	3.76097E-06	7.44633E-06	manganese/zinc transport system substrate-binding protein
K19976	1.74082E-06	1.15625E-06	9.83218E-06	1.1042E-05	manganese/zinc transport system permease protein
K19978	0	0	1.60675E-07	0	acarbose 7IV-phosphotransferase [EC:2.7.1.187]
K19979	0	0	6.06684E-08	1.12184E-06	2-epi-5-epi-valiolone 7-kinase [EC:2.7.1.188]
K19981	1.54063E-09	8.99401E-09	1.11332E-07	8.03716E-09	monodechloroaminopyrrolnitrin synthase
K19982	2.43131E-09	5.13337E-07	4.81468E-08	5.26376E-08	aminopyrrolnitrin oxygenase [EC:1.14.13.-]
K19997	5.4352E-07	1.31367E-06	1.82536E-06	5.82147E-07	GlcNAc-P-P-Und epimerase [EC:5.1.3.26]
K20021	0	0	3.09713E-08	0	L-cysteine desulfidase [EC:4.4.1.28]
K20023	1.57486E-06	3.22656E-06	1.55277E-05	4.659E-05	L-talarate/galactarate dehydratase [EC:4.2.1.156 4.2.1.42]
K20024	3.11512E-09	0	0	0	monoglucosyldiacylglycerol epimerase [EC:5.1.3.34]
K20025	3.2196E-07	1.32452E-07	4.78392E-07	3.80348E-09	(R)-2-hydroxyisocaproyl-CoA dehydratase alpha subunit [EC:4.2.1.157]
K20026	3.2196E-07	1.32452E-07	2.34108E-07	3.80348E-09	(R)-2-hydroxyisocaproyl-CoA dehydratase beta subunit [EC:4.2.1.157]
K20034	0.000218238	0.00021935	0.000222254	0.000195746	3-(methylthio)propionyl---CoA ligase [EC:6.2.1.44]
K20035	0.000655606	0.000651353	0.000626672	0.000523933	3-(methylthio)propanoyl-CoA dehydrogenase [EC:1.3.8.-]
K20036	0.000214016	0.000212085	0.000187943	0.000118928	(methylthio)acryloyl-CoA hydratase [EC:4.2.1.155]
K20037	2.46365E-08	8.91825E-08	3.12713E-06	1.81848E-06	choline trimethylamine-lyase activating enzyme [EC:1.97.1.-]
K20038	1.20691E-09	2.09074E-07	3.30431E-06	1.82396E-06	choline trimethylamine-lyase [EC:4.3.99.4]
K20073	9.83032E-08	7.94201E-08	2.49895E-07	3.45858E-06	mid-cell-anchored protein Z
K20074	0.000229244	0.000235345	0.000283606	0.00032297	PPM family protein phosphatase [EC:3.1.3.16]
K20075	0	0	2.22133E-08	0	tryptophan oxidase StaO [EC:1.4.3.-]
K20076	0	0	2.22133E-08	0	chromopyrrolic acid synthase
K20077	2.16728E-08	2.78476E-08	4.0533E-07	1.86974E-07	flavin-dependent monooxygenase StaC [EC:1.13.12.-]
K20078	0	0	1.08066E-07	7.33744E-08	cytochrome P450 StaP [EC:1.13.12.-]
K20079	3.97169E-09	1.30024E-08	1.39543E-08	0	N-glycosyltransferase StaG [EC:2.4.-.-]
K20080	0	0	4.35362E-08	0	cytochrome P450 StaN [EC:1.14.-.-]
K20081	0	0	4.35362E-08	0	N-methyltransferase StaMA [EC:2.1.1.-]
K20082	0	0	1.04657E-08	0	O-methyltransferase StaMB [EC:2.1.1.139]
K20086	3.24676E-08	6.5978E-09	0	0	tryptophan oxidase VioA [EC:1.4.3.-]
K20087	3.24676E-08	6.5978E-09	2.22133E-08	0	violacein biosynthesis protein VioB
K20088	3.24676E-08	6.5978E-09	0	0	violacein biosynthesis protein VioE
K20089	3.75525E-08	1.00268E-08	2.59514E-08	0	protodeoxyviolaceinate monooxygenase [EC:1.14.13.217]
K20090	3.24676E-08	6.5978E-09	0	0	violacein synthase [EC:1.14.13.224]
K20107	5.09299E-07	5.88853E-07	1.09135E-05	1.43909E-05	PTS system, maltose-specific IIB component [EC:2.7.1.208]
K20108	5.09299E-07	5.88853E-07	1.09135E-05	1.43909E-05	PTS system, maltose-specific IIC component
K20109	0	0	8.60025E-08	0	Pro-Pro endopeptidase [EC:3.4.24.89]
K20110	3.31529E-08	0	0	0	desampylase [EC:3.4.19.15]
K20112	1.80257E-07	9.885E-08	3.44383E-07	2.93143E-06	PTS system, galactose-specific IIA component [EC:2.7.1.204]
K20113	9.64869E-08	8.24747E-08	1.84419E-07	2.89856E-06	PTS system, galactose-specific IIB component [EC:2.7.1.204]
K20114	6.29151E-08	8.35589E-08	1.79851E-07	2.79068E-06	PTS system, galactose-specific IIC component
K20116	0.000218752	0.000215406	0.000218276	0.000145147	PTS system, glucose-specific IIA component [EC:2.7.1.199]
K20117	0.000221209	0.000216721	0.000227817	0.000157992	PTS system, glucose-specific IIB component [EC:2.7.1.199]
K20118	0.000221209	0.000216721	0.000227817	0.000157992	PTS system, glucose-specific IIC component
K20136	0	0	2.94883E-07	4.46004E-08	alpha-lytic endopeptidase [EC:3.4.21.12]
K20138	0	0	7.6371E-09	0	flavastacin [EC:3.4.24.76]
K20140	8.77617E-07	2.78434E-06	3.92062E-07	0	2-oxoglutarate carboxylase large subunit [EC:6.4.1.7]
K20141	1.03701E-07	2.34245E-07	1.55115E-07	7.24286E-07	2-oxoglutarate carboxylase small subunit [EC:6.4.1.7]
K20148	1.44434E-10	2.69767E-10	4.44994E-07	2.64433E-08	acetylacetone-cleaving enzyme [EC:1.13.11.50]
K20151	9.9505E-08	2.55747E-07	4.13676E-08	2.26894E-07	alpha(1,3/1,4) fucosyltransferase [EC:2.4.1.65 2.4.1.152]
K20155	4.92514E-09	9.19807E-10	0	3.48277E-08	4-methylaminobutanoate oxidase (methylamine-forming) [EC:1.5.3.21]
K20157	0	0	1.81612E-08	0	enduracididine beta-hydroxylase [EC:1.14.11.40]

K20158	1.44434E-10	1.847E-10	1.86888E-07	7.91166E-08	pseudooxynicotine oxidase [EC:1.4.3.24]
K20169	1.44434E-10	1.847E-10	1.08828E-08	4.98775E-09	nicotine oxidoreductase [EC:1.5.3.-]
K20170	7.35527E-07	7.75743E-07	5.13356E-06	1.89258E-08	nicotine oxidoreductase [EC:1.5.3.-]
K20199	0	6.49767E-07	5.07606E-08	5.79659E-09	4-hydroxybenzaldehyde dehydrogenase (NADP+) [EC:1.2.1.96]
K20200	0	0	2.15079E-09	0	4-cresol dehydrogenase [hydroxylating] cytochrome subunit
K20201	3.20455E-07	4.62652E-07	7.71419E-07	4.74457E-08	protein arginine phosphatase [EC:3.9.1.2]
K20202	0	0	7.15978E-09	1.4193E-07	sulfide dehydrogenase subunit alpha [EC:1.8.1.19]
K20203	3.31128E-07	1.51647E-07	2.34108E-07	3.80348E-09	sulfide dehydrogenase subunit beta [EC:1.8.1.19]
K20218	3.35971E-08	5.19079E-08	1.64645E-07	1.86974E-07	4-hydroxyisophthalate hydroxylase
K20219	7.94338E-09	0	2.45095E-08	0	o-aminophenol oxidase [EC:1.10.3.4]
K20248	0	3.8155E-08	4.69177E-09	0	acyl homoserine lactone synthase [EC:2.3.1.184]
K20249	0	4.99842E-09	0	0	acyl homoserine lactone synthase [EC:2.3.1.184]
K20250	2.64521E-07	1.38999E-08	6.47375E-08	1.27188E-09	acyl homoserine lactone synthase [EC:2.3.1.184]
K20252	0	0	4.69177E-09	0	LuxR family transcriptional regulator, quorum-sensing system regulator CinR
K20253	0	0	1.17294E-09	0	LuxR family transcriptional regulator, quorum-sensing system regulator BisR
K20257	1.03511E-09	8.82132E-09	6.3447E-08	0	2-aminobenzoylacetyl-CoA thioesterase [EC:3.1.2.32]
K20260	3.0693E-10	5.56836E-10	8.84915E-09	5.88675E-09	phenazine biosynthesis protein
K20261	1.71743E-07	7.09792E-09	1.92369E-07	3.9295E-06	trans-2,3-dihydro-3-hydroxyanthranilic acid synthase [EC:3.3.2.15]
K20262	3.0693E-10	1.48285E-08	4.33815E-09	2.15253E-07	dihydrophenazinedicarboxylate synthase [EC:1.10.3.16]
K20263	1.40336E-07	1.04559E-06	7.6488E-07	1.47519E-05	two-component system, NarL family, sensor histidine kinase FusK [EC:2.7.13.3]
K20264	1.32141E-07	7.14722E-07	9.90713E-07	1.44644E-05	two-component system, NarL family, response regulator FusR
K20265	1.08297E-06	1.2737E-06	8.43249E-06	2.12717E-06	glutamate:GABA antiporter
K20266	3.25237E-06	4.01107E-06	1.89204E-05	5.18921E-05	type IV secretion system protein TrbJ
K20267	2.01488E-07	4.03924E-08	8.07403E-08	0	type IV secretion system protein TrbH
K20268	1.44434E-10	1.42313E-09	1.81478E-08	5.28572E-10	rhizosphere induced protein
K20269	2.40676E-07	5.85752E-07	2.74082E-06	6.92149E-06	rhizosphere induced protein
K20271	0	0	3.82026E-08	3.42313E-09	DeoR family transcriptional regulator, repressor of opine catabolism and conjugal transfer
K20272	0	4.03924E-08	6.1171E-08	0	TraR antiactivator
K20273	8.8662E-08	1.46163E-08	8.97475E-07	1.10683E-07	zinc metalloprotease ZmpA
K20274	4.48477E-08	7.04575E-08	1.23073E-07	1.20228E-08	zinc metalloprotease ZmpB
K20275	2.50352E-09	3.34325E-09	2.49289E-08	0	nematocidal protein AidA
K20276	0.000431439	0.000426712	0.000394329	0.000258514	large repetitive protein
K20277	0.000213779	0.000211791	0.0001862	0.000113219	mannose-binding lectin
K20319	0	6.73516E-07	5.43437E-08	0	beta-lactamase class C ADC [EC:3.5.2.6]
K20320	1.44154E-07	6.98342E-10	9.36394E-07	7.90988E-07	beta-lactamase class C PDC [EC:3.5.2.6]
K20325	3.84746E-08	1.82082E-07	3.88358E-07	1.54195E-07	TetR/AcrR family transcriptional regulator, Clp-modulated transcription factor
K20326	3.75735E-07	4.94744E-08	3.64715E-07	1.10626E-07	protein XagA
K20327	2.19207E-06	2.75638E-07	5.71344E-07	1.96417E-06	glycosyltransferase XagB
K20328	2.997E-09	0	1.36678E-07	0	membrane protein XagC
K20329	0.000214037	0.000212499	0.0001904	0.000120362	IclR family transcriptional regulator, positive regulator for flagellar biogenesis
K20330	3.80343E-09	2.10967E-08	1.29599E-08	1.99964E-08	LuxR family transcriptional regulator, activator of tox operons
K20331	2.0579E-07	1.57573E-07	3.61029E-07	1.35567E-05	toxoflavin biosynthesis protein ToxA
K20332	6.00905E-09	7.89544E-09	6.80212E-08	8.03716E-09	toxoflavin biosynthesis protein ToxC
K20333	8.62246E-07	1.41188E-06	7.27662E-06	8.64493E-06	toxoflavin biosynthesis protein ToxD
K20334	0	0	0	1.65082E-08	LuxR family transcriptional regulator, quorum-sensing system regulator CviR
K20337	5.26162E-06	0	1.62794E-05	1.71459E-07	phenol-soluble modulin beta
K20338	9.36655E-07	0	2.74951E-06	3.03626E-08	MarR family transcriptional regulator, global regulator for virulence
K20341	7.77404E-08	0	4.04706E-08	1.78575E-07	immunity protein
K20342	1.38638E-07	1.5884E-07	1.22206E-07	2.95647E-06	HTH-type transcriptional regulator, regulator for ComX
K20343	7.29951E-08	0	3.93952E-08	1.87373E-07	peptide pheromone BlpC
K20344	6.50864E-06	4.20701E-06	2.09049E-05	8.15316E-05	ATP-binding cassette, subfamily C, bacteriocin exporter
K20345	5.26556E-07	5.69187E-07	3.30772E-07	4.8205E-07	membrane fusion protein, peptide pheromone/bacteriocin exporter
K20370	5.05029E-07	2.61814E-07	5.9225E-06	4.05387E-08	phosphoenolpyruvate carboxykinase (diphosphate) [EC:4.1.1.38]
K20373	6.03411E-08	7.94201E-08	1.41907E-07	2.7089E-06	HTH-type transcriptional regulator, SHP2-responsive activator
K20374	2.37263E-08	7.94201E-08	1.04304E-07	9.88845E-07	HTH-type transcriptional regulator, SHP3-responsive repressor
K20375	0	0	0	2.27374E-10	HTH-type transcriptional regulator, pheromone-responsive regulator
K20379	1.04582E-08	0	2.85396E-08	8.13677E-07	sex pheromone cAD1
K20381	0	0	0	1.68775E-07	regulatory protein
K20382	0	0	0	6.54524E-07	aggregation substance
K20384	0	0	0	3.27262E-07	CylL-S protein
K20385	0	0	0	1.87007E-07	CylM protein
K20386	0	0	0	1.87007E-07	ATP-binding cassette, subfamily B, bacterial CylB
K20388	0	0	0	1.40255E-07	HTH-type transcriptional regulator, cytolysin regulator
K20390	0	0	4.48531E-09	0	regulatory peptide PapR
K20391	0	0	4.48531E-09	0	HTH-type transcriptional regulator, pleiotropic regulator of extracellular virulence genes
K20415	2.91698E-11	5.07097E-09	2.10064E-10	3.18055E-08	ACP-SH:acetate ligase [EC:6.2.1.35]
K20418	0	0	4.48531E-09	0	L-isoleucine 4-hydroxylase [EC:1.14.11.45]
K20420	3.12928E-06	1.3606E-06	5.7083E-06	5.58459E-06	2-hydroxy-5-methyl-1-naphthoate 7-hydroxylase [EC:1.14.99.49]
K20421	1.78514E-08	2.81207E-08	5.05374E-07	3.17018E-08	2,7-dihydroxy-5-methyl-1-naphthoate 7-O-methyltransferase [EC:2.1.1.303]
K20422	5.50534E-08	1.55925E-07	1.0024E-06	1.80547E-07	neocarzinostatin naphthoate synthase [EC:2.3.1.237]
K20423	2.96526E-08	3.14909E-08	1.96949E-07	1.65966E-07	2-hydroxy-7-methoxy-5-methyl-1-naphthoate---CoA ligase [EC:6.2.1.43]
K20424	0	0	6.06684E-08	1.12184E-06	2-epi-valiolone-7-phosphate 1-reductase [EC:1.1.1.-]
K20425	0	0	1.27285E-08	0	cyclitol oxidoreductase
K20427	0	0	7.21251E-08	0	valienol-1-phosphate guanylyltransferase [EC:2.7.7.91]
K20428	0	8.50664E-11	6.41127E-08	0	dTDP-4-amino-4,6-dideoxy-D-glucose transaminase [EC:2.6.1.33]
K20429	0.000216103	0.000214446	0.000201462	0.000126108	dTDP-4-amino-4,6-dideoxy-D-glucose transaminase [EC:2.6.1.33]
K20430	0	0	5.76604E-08	0	glycosyltransferase AcbS [EC:2.4.-.-]
K20431	3.3965E-07	4.62596E-07	3.79789E-07	3.43989E-06	2-epi-5-epi-valiolone epimerase [EC:5.1.3.33]
K20432	0	0	7.21251E-08	0	5-epi-valiolone dehydratase [EC:4.2.1.-]
K20433	0	0	1.44647E-08	0	C7-cyclitol 7-kinase [EC:2.7.1.214]
K20434	0	0	1.2839E-07	0	cyclitol reductase
K20435	0	0	8.00804E-08	0	validone 7-phosphate aminotransferase [EC:2.6.1.-]
K20436	0	0	7.21251E-08	0	validamine 7-phosphate valienyltransferase [EC:2.5.1.135]
K20444	1.46516E-05	1.15111E-05	4.11497E-05	0.000150424	O-antigen biosynthesis protein [EC:2.4.1.-]
K20445	8.99101E-09	3.26743E-08	9.84717E-09	3.62376E-07	nicotinate dehydrogenase FAD-subunit [EC:1.17.1.5]
K20446	0	3.27506E-09	1.78808E-07	0	nicotinate dehydrogenase small FeS subunit [EC:1.17.1.5]
K20447	1.37567E-06	2.08352E-06	9.00163E-06	2.317E-05	nicotinate dehydrogenase large molybdopterin subunit [EC:1.17.1.5]

K20448	3.31683E-07	1.7378E-07	4.02196E-07	2.26764E-08	nicotinate dehydrogenase medium molybdopterin subunit [EC:1.17.1.5]
K20449	2.6472E-08	3.9023E-08	1.25996E-07	5.0875E-09	6-hydroxynicotinate reductase [EC:1.3.7.1]
K20451	2.6504E-07	1.62798E-07	6.69153E-07	1.71995E-05	methylitaconate Delta-isomerase [EC:5.3.3.6]
K20454	1.5306E-06	3.26777E-06	1.65064E-05	3.94351E-05	2,3-dimethylmalate lyase [EC:4.1.3.32]
K20455	0.000220366	0.000222284	0.00023836	0.000279245	2-methylcitrate dehydratase (2-methyl-trans-aconitate forming) [EC:4.2.1.117]
K20458	0	3.19642E-08	2.89098E-07	1.80347E-06	3-hydroxybenzoate/4-hydroxybenzoate---CoA ligase [EC:6.2.1.37 6.2.1.27]
K20459	1.63579E-06	2.72832E-06	3.76878E-06	4.60873E-06	lantibiotic transport system ATP-binding protein
K20460	4.44519E-08	7.72438E-07	8.71935E-07	2.0752E-07	lantibiotic transport system permease protein
K20461	4.52522E-07	9.90382E-07	1.08283E-06	2.69091E-07	lantibiotic transport system permease protein
K20466	0	1.45407E-07	2.34901E-07	9.51921E-08	heme transporter
K20467	0	1.55873E-07	1.54857E-08	2.76662E-08	heme-binding protein
K20468	0	5.96007E-07	2.44404E-06	1.76322E-06	putative heme transporter
K20469	1.61192E-06	4.15014E-06	9.59891E-06	7.82694E-06	putative heme transporter
K20470	9.04107E-08	5.96959E-07	2.99061E-06	3.78288E-06	trehalose monomycolate/heme transporter
K20480	3.0496E-07	1.29822E-07	6.56488E-07	1.00996E-06	HTH-type transcriptional regulator, quorum sensing regulator NprR
K20481	0	0	4.48531E-09	0	regulatory peptide NprX
K20482	0	0	5.84265E-09	4.93465E-09	lantibiotic bacteriocin
K20483	4.73325E-08	5.30111E-07	5.90292E-07	1.58268E-07	lantibiotic biosynthesis protein
K20484	4.39802E-08	7.24899E-08	2.32179E-07	8.64829E-08	lantibiotic biosynthesis protein
K20485	2.43854E-07	2.41537E-07	5.09362E-07	1.71641E-07	ATP-binding cassette, subfamily B, bacterial NisT
K20486	1.2654E-08	7.16519E-07	1.52054E-07	2.41088E-07	lantibiotic leader peptide-processing serine protease [EC:3.4.21.-]
K20487	7.69228E-07	5.65691E-07	1.77275E-06	1.01523E-05	two-component system, OmpR family, lantibiotic biosynthesis sensor histidine kinase NisK/SpaK [EC:2.7.13.3]
K20488	7.84923E-06	7.59881E-06	2.41517E-05	8.10892E-05	two-component system, OmpR family, lantibiotic biosynthesis response regulator NisR/SpaR
K20489	0	8.28414E-08	2.0605E-07	6.72885E-08	lantibiotic immunity protein
K20490	2.13616E-06	1.81656E-06	4.04583E-06	4.15975E-06	lantibiotic transport system ATP-binding protein
K20491	1.87656E-08	6.01245E-07	1.63711E-06	7.21361E-07	lantibiotic transport system permease protein
K20492	3.1635E-09	1.64816E-07	2.28039E-07	3.51574E-07	lantibiotic transport system permease protein
K20494	1.24386E-08	0	1.6748E-09	0	lantibiotic transport system permease protein
K20497	2.503E-07	1.56697E-06	2.28534E-06	6.00821E-06	methyl-branched lipid omega-hydroxylase [EC:1.14.15.14]
K20500	1.3865E-09	5.5865E-09	2.26103E-08	2.23974E-08	L-lysine cyclodeaminase [EC:4.3.1.28]
K20509	1.70432E-07	5.85641E-07	3.81585E-08	3.18055E-08	carboxybiotin decarboxylase [EC:4.3.99.2]
K20510	2.91698E-11	7.42945E-09	1.69162E-08	3.18055E-08	malonyl-S-ACP:biotin-protein carboxyltransferase subunit MadC [EC:2.1.3.10]
K20511	2.91698E-11	1.00694E-08	2.10064E-10	3.18055E-08	malonyl-S-ACP:biotin-protein carboxyltransferase subunit MadD [EC:2.1.3.10]
K20525	5.86926E-07	3.88023E-07	1.23428E-06	4.66726E-05	oligo-alginate lyase [EC:4.2.2.26]
K20527	0.000220217	0.000218963	0.00021964	0.00018428	type IV secretion system protein TrbB
K20528	3.21361E-06	3.96675E-06	1.88333E-05	5.03676E-05	type IV secretion system protein TrbC
K20529	3.22019E-06	3.97477E-06	1.8803E-05	5.0354E-05	type IV secretion system protein TrbD
K20530	3.39542E-06	4.36156E-06	1.98504E-05	5.13714E-05	type IV secretion system protein TrbE
K20531	3.23046E-06	3.98464E-06	1.88523E-05	5.03643E-05	type IV secretion system protein TrbF
K20532	0.000218214	0.000218716	0.000218663	0.000198253	type IV secretion system protein TrbG
K20533	3.39449E-06	4.00436E-06	2.00508E-05	5.18396E-05	type IV secretion system protein TrbI
K20534	0.000227322	0.000226784	0.00023088	0.000275116	polyisoprenyl-phosphate glycosyltransferase [EC:2.4.-.-]
K20539	4.64219E-07	1.43615E-07	2.51335E-07	1.10109E-08	IclR family transcriptional regulator, blcABC operon repressor
K20540	1.66099E-09	2.12405E-09	5.32955E-08	0	LuxR family transcriptional regulator, quorum-sensing system regulator CepR2
K20541	1.07514E-06	6.21196E-07	1.49513E-06	2.30389E-05	cellulose synthase operon protein B
K20542	9.78887E-07	5.91338E-07	1.76714E-06	2.55885E-05	endoglucanase [EC:3.2.1.4]
K20543	1.2572E-06	6.72239E-07	1.48645E-06	2.73906E-05	cellulose synthase operon protein C
K20547	4.96461E-09	2.18337E-09	4.44042E-07	1.13132E-05	basic endochitinase B [EC:3.2.1.14]
K20552	9.98255E-10	2.48287E-09	7.18885E-09	2.95705E-08	transcriptional regulator of LEE operons
K20555	0	4.03924E-08	4.83594E-08	0	type IV secretion system protein TrbK
K20584	0	0	5.23286E-09	0	6-hydroxyparomomycin oxidase [EC:1.1.3.-]
K20608	1.2579E-06	4.64046E-07	3.90144E-06	4.70871E-08	tetrahedral aminopeptidase [EC:3.4.11.-]
K20609	1.50089E-09	3.73304E-09	3.98957E-08	5.96737E-08	putative aminopeptidase [EC:3.4.11.-]
K20611	9.4765E-07	4.51132E-06	1.8726E-06	2.04779E-06	1-hydroxy-2-isopentenylcarotenoid 3,4-desaturase [EC:1.3.99.37]
K20616	1.58078E-08	5.01467E-08	3.09397E-07	3.80161E-06	lycopene elongase/1,2-hydratase
K20626	3.2196E-07	1.32452E-07	4.78392E-07	3.80348E-09	lactoyl-CoA dehydratase subunit alpha [EC:4.2.1.54]
K20627	3.2196E-07	1.32452E-07	4.78392E-07	3.80348E-09	lactoyl-CoA dehydratase subunit beta [EC:4.2.1.54]
K20628	0.000213808	0.00021181	0.000188831	0.000113458	expansin
K20656	0	0	0	8.03716E-09	transmembrane protein 189
K20708	1.72244E-07	1.83275E-08	2.94674E-07	0	isoleucine 2-epimerase [EC:5.1.1.21]
K20712	0.000215079	0.000214805	0.000200391	0.000147985	3-(hydroxyamino)phenol mutase [EC:5.4.4.3]
K20719	0	1.06439E-08	0	0	sigma non-opioid intracellular receptor
K20742	1.0663E-06	8.91529E-07	3.08491E-06	8.91828E-08	gamma-D-glutamyl-L-lysine dipeptidyl-peptidase [EC:3.4.14.13]
K20744	0	0	1.44647E-08	0	mycolysin [EC:3.4.24.31]
K20749	0	0	1.18611E-07	0	beta-lytic metalloendopeptidase [EC:3.4.24.32]
K20750	1.34865E-08	1.83275E-08	0	0	beta-peptidyl aminopeptidase [EC:3.4.11.25]
K20754	9.2481E-07	3.90208E-06	3.73491E-06	3.79396E-06	aqualysin 1 [EC:3.4.21.111]
K20755	6.14829E-07	4.15146E-07	2.31051E-06	4.52008E-05	Lys-Lys/Arg-Xaa endopeptidase [EC:3.4.21.121]
K20757	0.000215995	0.000215936	0.000201661	0.000149886	threo-3-hydroxy-D-aspartate ammonia-lyase [EC:4.3.1.27]
K20760	0	2.12879E-08	0	4.11447E-08	thiocyanate hydrolase subunit alpha [EC:3.5.5.8]
K20761	0	2.12879E-08	0	4.11447E-08	thiocyanate hydrolase subunit beta [EC:3.5.5.8]
K20762	6.22255E-07	5.19018E-07	3.98677E-07	8.12554E-06	thiocyanate hydrolase subunit gamma [EC:3.5.5.8]
K20765	1.00516E-06	6.9377E-08	2.94993E-07	2.05973E-07	6-oxocamphor hydrolase [EC:3.7.1.18]
K20766	5.39461E-08	2.83838E-08	4.09739E-08	5.65278E-09	arylacetonitrilase [EC:3.5.5.5]
K20786	0	0	2.60187E-07	1.78604E-09	niddamycin polyketide synthase 2
K20787	1.18913E-09	4.06295E-09	2.08932E-06	1.7011E-07	candicidin polyketide synthase FscC
K20788	4.40315E-08	2.94241E-07	2.52373E-06	4.85235E-07	myxalamid-type polyketide synthase MxaE
K20791	0	1.31002E-09	1.16286E-08	4.27891E-09	N-alpha-acetyltransferase 10/11 [EC:2.3.1.255]
K20801	1.34865E-07	6.72246E-08	6.32857E-07	3.19548E-06	L-allo-threonine aldolase [EC:4.1.2.49]
K20806	0	0	1.81612E-08	0	bromoxynil nitrilase [EC:3.5.5.6]
K20807	1.04554E-06	2.96476E-07	1.48502E-06	1.84473E-06	nitrile hydratase subunit beta [EC:4.2.1.84]
K20810	1.7982E-08	1.17924E-09	8.1308E-07	1.23811E-07	aminodeoxyfutalosine deaminase [EC:3.5.4.40]
K20811	5.82373E-08	1.22184E-08	3.01649E-07	6.69764E-09	inulosucrase [EC:2.4.1.9]
K20812	0	0	3.96562E-08	0	glycogen synthase [EC:2.4.1.242]
K20814	1.98395E-06	6.58754E-06	5.3299E-06	4.11646E-06	Pup amidohydrolase [EC:3.5.1.119]
K20816	0	0	3.48857E-09	0	streptothricin hydrolase [EC:3.5.2.19]
K20817	3.37013E-10	3.72233E-08	2.05853E-06	1.05773E-07	N-acyl-D-aspartate deacylase [EC:3.5.1.83]

K20829	0	0	6.2198E-08	0	cellulose 1,4-beta-cellobiosidase [EC:3.2.1.176]
K20832	0	0	3.85108E-08	1.53121E-08	galactan endo-beta-1,3-galactanase [EC:3.2.1.181]
K20844	3.11219E-08	1.99552E-08	6.4698E-07	5.12365E-08	non-reducing end alpha-L-arabinofuranosidase [EC:3.2.1.55]
K20847	0	0	6.97714E-09	0	glucan 1,6-alpha-isomaltosidase [EC:3.2.1.94]
K20859	5.54378E-08	1.63753E-08	2.1226E-07	2.35712E-09	phosphoribosyl 1,2-cyclic phosphate 1,2-diphosphodiesterase [EC:3.1.4.57]
K20861	2.60774E-06	1.59327E-06	1.75742E-05	7.83374E-06	FMN hydrolase / 5-amino-6-(5-phospho-D-ribitylamino)uracil phosphatase [EC:3.1.3.102 3.1.3.104]
K20862	2.71204E-06	4.97153E-06	2.00747E-05	0.000104656	FMN hydrolase / 5-amino-6-(5-phospho-D-ribitylamino)uracil phosphatase [EC:3.1.3.102 3.1.3.104]
K20866	1.57932E-06	1.76901E-06	5.22291E-06	5.35758E-05	glucose-1-phosphatase [EC:3.1.3.10]
K20881	2.53557E-07	7.38177E-07	1.5184E-06	8.8916E-06	5-nucleotidase [EC:3.1.3.5]
K20882	2.8804E-08	2.49671E-08	3.85262E-07	5.67606E-08	(R)-2-hydroxy-4-methylpentanoate CoA-transferase [EC:2.8.3.24]
K20883	3.24109E-11	3.87351E-08	2.33404E-10	2.12542E-07	alpha-1,2-colitosyltransferase [EC:2.4.1.341]
K20885	1.11097E-07	2.9152E-07	2.66828E-06	1.59576E-07	beta-1,2-mannobiose phosphorylase / 1,2-beta-oligomannan phosphorylase [EC:2.4.1.339 2.4.1.340]
K20895	8.64813E-07	0	2.88616E-06	3.89081E-08	formylaminopyrimidine deformylase [EC:3.5.1.-]
K20896	0	1.13298E-07	2.03153E-07	7.58314E-08	formylaminopyrimidine deformylase / aminopyrimidine aminohydrolase [EC:3.5.1.- 3.5.99.-]
K20900	1.24833E-06	3.19147E-06	1.39298E-05	3.46274E-05	D-threitol dehydrogenase (NAD+) [EC:1.1.1.403]
K20901	5.08037E-07	3.99092E-07	5.64799E-06	1.55232E-07	L-erythrulose 1-kinase [EC:2.7.1.209]
K20902	5.31825E-07	4.13527E-07	5.70175E-06	1.6928E-07	D-erythrulose 4-kinase [EC:2.7.1.210]
K20903	6.4392E-07	2.64904E-07	8.84334E-07	4.02645E-08	(R)-2-hydroxyglutaryl-CoA dehydratase subunit alpha [EC:4.2.1.167]
K20904	6.4392E-07	2.64904E-07	7.62449E-07	4.02645E-08	(R)-2-hydroxyglutaryl-CoA dehydratase subunit beta [EC:4.2.1.167]
K20906	1.1988E-08	0	6.97714E-09	6.33538E-08	2-hydroxyisobutanoyl-CoA mutase large subunit [EC:5.4.99.64]
K20907	8.14197E-08	0	0	6.33538E-08	2-hydroxyisobutanoyl-CoA mutase small subunit [EC:5.4.99.64]
K20918	3.24676E-08	7.21737E-07	4.0317E-07	1.6508E-05	LuxR family transcriptional regulator, positive regulator of biofilm formation
K20919	0.000214733	0.000212729	0.000190149	0.00013231	sigma-54 dependent transcriptional regulator, positive regulator of biofilm formation
K20920	4.0972E-08	3.65031E-07	7.41249E-08	1.25812E-05	polysaccharide biosynthesis protein VpsM
K20921	0	0	0	2.77766E-06	polysaccharide biosynthesis protein VpsD
K20922	0	0	4.30157E-09	2.77766E-06	polysaccharide biosynthesis protein VpsI
K20927	3.955E-08	1.23254E-07	3.78297E-07	8.34118E-08	2-methyl-1,2-propanediol dehydrogenase [EC:1.1.1.400]
K20928	8.73988E-08	2.83838E-08	7.1817E-08	3.49089E-08	2-hydroxy-2-methylpropanal dehydrogenase [EC:1.2.1.98]
K20937	7.94338E-09	4.50994E-07	2.18434E-06	4.58192E-07	1-butanol dehydrogenase (quinone) [EC:1.1.5.11]
K20938	1.28828E-05	1.13786E-05	3.50471E-05	0.000129904	long-chain alkane monooxygenase [EC:1.14.14.28]
K20940	3.53855E-06	5.78852E-06	2.88876E-05	6.5039E-05	5-methylphenazine-1-carboxylate 1-monooxygenase [EC:1.14.13.218]
K20941	8.86982E-08	1.22817E-07	1.67357E-06	5.48445E-07	gamma-resorcylate decarboxylase [EC:4.1.1.103]
K20942	1.2987E-08	3.30937E-08	1.5809E-06	6.2209E-07	resorcinol 4-hydroxylase (FADH2) [EC:1.14.14.27]
K20943	1.48175E-08	7.14482E-09	3.21446E-08	1.9113E-07	resorcinol 4-hydroxylase (NADPH) [EC:1.14.13.219]
K20945	0	0	1.57351E-08	5.49961E-06	tyrosine-protein phosphatase [EC:3.1.3.48]
K20946	0	0	0	1.26022E-06	polysaccharide biosynthesis protein VpsE
K20947	0	0	0	1.37463E-06	polysaccharide biosynthesis protein VpsF
K20948	3.1294E-10	5.01703E-09	0	8.31459E-06	polysaccharide biosynthesis protein VpsJ
K20949	0	0	0	3.81586E-06	polysaccharide biosynthesis protein VpsP
K20950	0	0	6.41787E-08	5.64441E-06	polysaccharide biosynthesis protein VpsQ
K20951	0	0	0	1.32753E-05	extracellular matrix protein
K20952	1.39069E-09	2.09172E-09	1.23329E-08	1.37781E-05	rugosity and biofilm structure modulator C
K20953	0	0	0	1.14939E-07	rugosity and biofilm structure modulator A
K20954	7.27844E-09	0	3.64302E-07	7.0726E-06	diguanylate cyclase [EC:2.7.7.65]
K20955	3.07608E-06	5.45927E-06	2.164E-05	0.000106072	diguanylate cyclase [EC:2.7.7.65]
K20956	4.17958E-07	2.63874E-07	1.33482E-06	1.43205E-05	diguanylate cyclase [EC:2.7.7.65]
K20957	2.3985E-08	4.73833E-07	6.50547E-07	1.26906E-05	diguanylate cyclase [EC:2.7.7.65]
K20958	3.4607E-07	5.93731E-08	4.96609E-07	2.23541E-05	diguanylate cyclase [EC:2.7.7.65]
K20959	5.27443E-07	4.77591E-07	1.35744E-06	5.07108E-05	diguanylate cyclase [EC:2.7.7.65]
K20960	0	0	4.42447E-09	5.90751E-06	diguanylate cyclase [EC:2.7.7.65]
K20961	9.99003E-07	2.62686E-06	1.46757E-05	2.90809E-05	diguanylate cyclase [EC:2.7.7.65]
K20962	0.000651454	0.000647943	0.000619038	0.000535297	c-di-GMP phosphodiesterase [EC:3.1.4.52]
K20964	1.34865E-07	1.35781E-08	1.11439E-07	3.72886E-06	c-di-GMP phosphodiesterase [EC:3.1.4.52]
K20965	8.00842E-06	7.82395E-06	4.58857E-05	0.000116019	c-di-GMP phosphodiesterase [EC:3.1.4.52]
K20966	0.000217047	0.000214843	0.000207358	0.000341805	c-di-GMP phosphodiesterase [EC:3.1.4.52]
K20967	0	2.55502E-09	2.26801E-07	1.91836E-06	GTP 3,8-cyclase / cyclic pyranopterin monophosphate synthase [EC:4.1.99.22 4.6.1.17]
K20968	0	8.28414E-08	5.76224E-08	1.84051E-06	AraC family transcriptional regulator, exoenzyme S synthesis regulatory protein ExsA
K20971	0.000222171	0.000222915	0.00024192	0.000336293	two-component system, sensor histidine kinase LadS
K20972	3.5727E-06	4.01114E-06	6.90776E-06	0.000125319	two-component system, sensor histidine kinase RetS
K20973	1.03279E-05	1.53228E-05	4.58388E-05	0.000152645	two-component system, sensor histidine kinase SagS [EC:2.7.13.3]
K20974	0.000448778	0.000444142	0.000436305	0.000575672	two-component system, sensor histidine kinase [EC:2.7.13.3]
K20975	0.000650123	0.000646432	0.000602443	0.000573893	two-component system, sensor histidine kinase [EC:2.7.13.3]
K20976	1.32646E-06	1.09857E-06	2.00637E-06	3.97361E-06	histidine phosphotransfer protein HptB
K20977	2.26194E-06	1.98515E-06	8.39828E-06	2.37423E-05	two-component system, HptB-dependent secretion and biofilm response regulator
K20978	8.13646E-08	1.14862E-07	1.23971E-06	1.09477E-05	HptB-dependent secretion and biofilm anti anti-sigma factor
K20987	0	6.2087E-08	3.23176E-07	2.7519E-08	polysaccharide biosynthesis/export protein PslD
K20988	4.0972E-08	3.65031E-07	1.27687E-07	1.18929E-05	polysaccharide biosynthesis/export protein VpsN
K20989	0	0	2.72753E-09	0	urea-proton symporter
K20990	3.97169E-09	9.82517E-09	3.48857E-08	5.70522E-08	4-sulfomuconolactone hydrolase [EC:3.1.1.92]
K20993	7.61656E-11	1.8944E-10	2.87421E-09	5.28196E-09	pyrethroid hydrolase [EC:3.1.1.88]
K20997	7.4189E-06	6.60227E-06	1.76267E-05	1.55284E-05	polysaccharide biosynthesis protein PslA
K20998	9.89526E-07	4.30163E-07	3.33679E-07	2.97448E-08	polysaccharide biosynthesis protein PslE
K20999	0	1.96672E-07	3.97968E-07	2.7519E-08	polysaccharide biosynthesis protein PslF
K21000	0	1.76564E-07	3.62384E-07	5.7095E-08	polysaccharide biosynthesis protein PslG
K21001	1.67994E-08	2.23824E-07	4.53163E-07	6.6826E-08	polysaccharide biosynthesis protein PslH
K21002	1.2987E-08	6.2087E-08	8.75959E-07	1.52597E-07	polysaccharide biosynthesis protein PslI
K21003	0	1.86028E-07	3.38662E-07	2.7519E-08	polysaccharide biosynthesis protein PslJ
K21004	0	6.2087E-08	3.23176E-07	2.7519E-08	polysaccharide biosynthesis protein PslK
K21005	1.82592E-07	5.58867E-07	5.33699E-07	3.61643E-08	polysaccharide biosynthesis protein PslL
K21006	0.000428916	0.000426665	0.000388403	0.000262323	polysaccharide biosynthesis protein PelA
K21007	0.00021513	0.000214806	0.000202267	0.000149006	polysaccharide biosynthesis protein PelB
K21008	0.00021513	0.000214806	0.000202264	0.000149001	polysaccharide biosynthesis protein PelC
K21009	0.000215134	0.000214847	0.000202292	0.000149026	polysaccharide biosynthesis protein PelD
K21010	0.00021513	0.000214846	0.000202291	0.000149026	polysaccharide biosynthesis protein PelE
K21011	0.000215562	0.000215979	0.00020318	0.000150969	polysaccharide biosynthesis protein PelF
K21012	0.000215252	0.00021485	0.0002023	0.000149236	polysaccharide biosynthesis protein PelG
K21014	4.81447E-11	1.24003E-07	1.86469E-07	1.73486E-06	trehalose 2-sulfotransferase [EC:2.8.2.37]

K21019	0.000667125	0.000675863	0.000726861	0.001039171	diguanylate cyclase [EC:2.7.7.65]
K21020	0.00065146	0.000653211	0.000641651	0.000606337	diguanylate cyclase [EC:2.7.7.65]
K21021	0.001536357	0.001533529	0.001535456	0.001634455	diguanylate cyclase [EC:2.7.7.65]
K21022	4.96129E-07	1.42126E-06	2.89061E-06	1.34848E-05	diguanylate cyclase [EC:2.7.7.65]
K21023	0.00372871	0.003707404	0.003669278	0.003941537	diguanylate cyclase [EC:2.7.7.65]
K21024	0.000668905	0.000665132	0.000695357	0.000900069	c-di-GMP phosphodiesterase [EC:3.1.4.52]
K21025	2.50191E-06	1.74149E-06	4.38888E-06	5.32495E-05	multidomain signaling protein FimX
K21028	7.0708E-08	1.67839E-07	2.7348E-07	4.48215E-06	molybdopterin synthase sulfurtransferase [EC:2.8.1.11]
K21029	0.000223707	0.000223724	0.00022955	0.000238903	molybdopterin-synthase adenylyltransferase [EC:2.7.7.80]
K21030	9.78788E-07	1.59146E-07	2.74079E-06	1.32967E-06	D-ribitol-5-phosphate cytidylyltransferase [EC:2.7.7.40]
K21033	3.97169E-09	2.12879E-08	1.22864E-07	6.76737E-09	cytochrome P450 family 103
K21034	3.52959E-08	1.43232E-08	1.28684E-07	2.31203E-07	cytochrome P450 family 104
K21036	0	0	2.89295E-08	0	validamycin A dioxygenase [EC:1.14.11.52]
K21039	3.14647E-08	1.2963E-07	5.14605E-08	2.88036E-07	enterobacteria phage integrase [EC:2.7.7.- 3.1.-.-]
K21053	0.00021622	0.000215623	0.000205439	0.00015868	adenine deaminase [EC:3.5.4.2]
K21054	1.06559E-06	1.91757E-07	4.2652E-07	3.39926E-06	D-erythritol 1-phosphate dehydrogenase [EC:1.1.1.402]
K21055	0	0	6.97714E-09	0	3-deoxy-D-glycero-D-galacto-nononate 9-phosphatase [EC:3.1.3.103]
K21056	5.03955E-08	2.45629E-08	3.08768E-07	5.20917E-09	alpha-D-ribose-1-phosphate 5-kinase (ADP) [EC:2.7.1.212]
K21059	1.19151E-08	3.0141E-07	1.24819E-07	5.6775E-08	4-hydroxyphenylalkanoate adenylyltransferase [EC:6.2.1.51]
K21060	1.71257E-09	3.11892E-09	2.96768E-07	2.84048E-06	D-hydroxyproline dehydrogenase [EC:1.5.99.-]
K21061	4.26928E-07	3.66265E-07	7.37841E-07	2.93408E-07	D-hydroxyproline dehydrogenase subunit beta [EC:1.5.99.-]
K21062	1.58236E-06	5.74437E-07	1.32642E-06	2.95419E-06	1-pyrroline-4-hydroxy-2-carboxylate deaminase [EC:3.5.4.22]
K21063	1.69364E-07	6.31137E-07	6.85175E-07	5.54947E-09	5-amino-6-(5-phospho-D-ribitylamino)uracil phosphatase [EC:3.1.3.104]
K21064	7.33314E-06	5.26338E-06	3.26259E-05	0.000109156	5-amino-6-(5-phospho-D-ribitylamino)uracil phosphatase [EC:3.1.3.104]
K21065	4.23211E-08	0	4.93331E-07	1.53089E-09	beta-1,2-mannosidase [EC:3.2.1.197]
K21071	7.00015E-06	1.17041E-05	3.6906E-05	9.07072E-05	ATP-dependent phosphofructokinase / diphosphate-dependent phosphofructokinase [EC:2.7.1.11 2.7.1.90]
K21084	0.000227009	0.000218141	0.000217211	0.000248401	diguanylate cyclase [EC:2.7.7.65]
K21085	0.000650518	0.000651689	0.0006322	0.000602868	diguanylate cyclase [EC:2.7.7.65]
K21086	2.38141E-07	8.64496E-07	1.28331E-07	1.63422E-06	c-di-GMP phosphodiesterase [EC:3.1.4.52]
K21087	1.08954E-07	3.40803E-07	1.45556E-07	8.12467E-07	flagellar brake protein
K21088	2.03546E-06	3.60045E-07	4.57274E-06	2.00237E-05	diguanylate cyclase [EC:2.7.7.65]
K21089	5.63803E-08	4.14961E-08	2.071E-07	9.8401E-06	MerR family transcriptional regulator, activator of the csg genes
K21090	6.51143E-06	1.5386E-05	6.01882E-05	0.00022142	c-di-GMP phosphodiesterase [EC:3.1.4.52]
K21104	3.50005E-08	2.88416E-08	6.49306E-07	1.20171E-07	poly(ethylene terephthalate) hydrolase [EC:3.1.1.101]
K21105	2.52854E-06	6.09672E-06	3.00569E-05	7.03122E-05	mono(ethylene terephthalate) hydrolase [EC:3.1.1.102]
K21113	6.97942E-08	1.66355E-07	3.57364E-07	5.86486E-07	cytochrome P450 family 106
K21114	0	0	4.48531E-09	8.03716E-09	steroid 15beta-monooxygenase [EC:1.14.15.8]
K21116	9.41916E-09	0	1.09509E-06	5.28866E-08	cytochrome P450 family 112 subfamily A
K21117	9.02714E-07	1.36268E-09	1.08537E-06	9.38673E-08	cytochrome P450 family 114
K21118	9.41916E-09	1.06439E-08	5.05358E-07	2.86415E-08	cytochrome P450 family 117 subfamily A
K21119	5.70858E-09	3.43678E-07	6.92781E-07	5.50799E-06	cytochrome P450 family 130
K21132	0	0	0	2.46092E-07	alpha-mannan endo-1,2-alpha-mannanase / glycoprotein endo-alpha-1,2-mannosidase [EC:3.2.1.198 3.2.1.130]
K21133	0.000222006	0.000218028	0.000210588	0.000241863	multidrug efflux pump
K21134	0.000220038	0.000219681	0.000218375	0.000241152	multidrug efflux pump
K21135	4.27983E-07	9.73598E-08	3.62787E-07	1.57309E-05	membrane fusion protein, multidrug efflux system
K21136	2.74213E-06	8.0007E-07	4.10885E-06	4.69513E-06	membrane fusion protein, multidrug efflux system
K21137	0.000215982	0.000212151	0.000187313	0.000113614	membrane fusion protein, multidrug efflux system
K21138	3.97169E-09	0	0	2.67905E-09	guanosine-3,5-bis(diphosphate) 3-pyrophosphohydrolase [EC:3.1.7.2]
K21140	7.6335E-08	3.75082E-07	6.86138E-07	1.92978E-07	[CysO sulfur-carrier protein]-S-L-cysteine hydrolase [EC:3.13.1.6]
K21142	9.15274E-07	5.03951E-09	3.03174E-06	2.76836E-08	MoaE-MoaD fusion protein [EC:2.8.1.12]
K21146	0	1.13519E-07	8.80811E-07	3.57207E-09	vitamin D 1,25-hydroxylase [EC:1.14.15.22]
K21147	0.000226285	0.000228816	0.000239026	0.000312177	sulfur-carrier protein adenylyltransferase/sulfurtransferase [EC:2.7.7.80 2.7.7.- 2.8.1.11 2.8.1.-]
K21148	1.26158E-06	1.16644E-06	1.87545E-06	3.805E-06	[CysO sulfur-carrier protein]-thiocarboxylate-dependent cysteine synthase [EC:2.5.1.113]
K21154	0	0	0	5.1331E-07	exopolysaccharide glucosyl ketal-pyruvate-transferase [EC:2.5.1.98]
K21159	1.663E-06	5.12885E-07	2.53092E-06	4.17634E-06	epoxide hydrolase
K21160	0	0	4.35362E-08	0	enediyne biosynthesis protein E2
K21161	0	0	6.57495E-08	0	enediyne biosynthesis protein E3
K21162	4.04966E-08	0	6.57495E-08	0	enediyne biosynthesis protein E4
K21163	0	0	6.57495E-08	8.03716E-09	enediyne biosynthesis protein E5
K21164	1.98225E-06	1.46153E-07	6.43405E-07	1.43022E-06	enediyne biosynthesis protein E7
K21165	0	0	4.35362E-08	0	enediyne biosynthesis protein E8
K21166	0	1.17924E-09	1.04857E-07	0	enediyne biosynthesis protein E9
K21167	0	1.48562E-07	3.10289E-07	1.81909E-06	enediyne biosynthesis protein E11
K21171	1.3865E-09	6.40947E-07	3.96977E-10	9.7156E-11	enediyne biosynthesis protein CalE4
K21173	0	4.44304E-09	0	8.03716E-09	methionine gamma-lyase
K21178	0	0	1.44647E-08	0	O-methyltransferase [EC:2.1.1.-]
K21181	0	0	3.05842E-07	1.36641E-08	MIO-dependent L-tyrosine 2,3-aminomutase [EC:5.4.3.6]
K21182	0	0	2.06475E-09	0	(S)-beta-tyrosine adenylation enzyme [EC:6.2.1.-]
K21184	1.16414E-07	3.09397E-07	4.0418E-07	2.23257E-06	two-component FAD-dependent monooxygenase [EC:1.14.14.15 1.14.14.-]
K21185	1.28028E-06	3.11125E-06	1.46999E-05	3.51605E-05	flavin reductase
K21186	0.000427531	0.00042355	0.000372415	0.000227367	condensation enzyme
K21188	9.0314E-08	5.11954E-07	3.44E-07	4.80743E-07	PLP-dependent transaminase
K21190	2.22254E-07	1.80324E-07	4.816E-07	1.40236E-05	dehydrogenase
K21191	9.62893E-11	9.08343E-08	5.04254E-08	1.64858E-08	CoA ligase [EC:6.2.1.-]
K21195	8.56287E-10	9.06995E-09	1.2582E-08	5.4955E-10	2-aminoethylphosphonate dioxygenase [EC:1.14.11.46]
K21196	2.56253E-08	1.20606E-07	4.15773E-07	7.70853E-07	2-amino-1-hydroxyethylphosphonate dioxygenase (glycine-forming) [EC:1.13.11.78]
K21199	0	1.06439E-08	9.57194E-08	6.65642E-08	cytochrome P450 family 128
K21200	0.000214422	0.000211982	0.000186911	0.000115396	cytochrome P450 family 144
K21201	0	0	1.0512E-07	2.39741E-08	cytochrome P450 family 119 [EC:1.11.1.7]
K21210	5.68427E-09	1.85547E-09	1.42055E-07	0	NDP-mannose synthase
K21211	1.39374E-08	4.35006E-09	1.29158E-07	9.46231E-09	NDP-hexose 4,6-dehydratase
K21214	0	1.22815E-08	2.64641E-07	1.46228E-08	NDP-hexose 4-ketoreductase
K21217	0	1.35781E-08	2.51348E-07	1.63345E-05	sodium-type polar flagellar protein MotX
K21218	2.72341E-07	8.80649E-07	2.04973E-06	2.57535E-05	sodium-type flagellar protein MotY
K21219	2.37835E-06	4.08059E-06	1.47149E-05	4.09481E-05	hydroxymethylpyrimidine kinase / phosphomethylpyrimidine kinase / thiamine-phosphate diphosphorylase [EC:2.7.1.4
K21224	0	0	4.35362E-08	0	radical SAM C-methyltransferase
K21231	0	4.59904E-09	0	1.52139E-08	3,8-divinyl protochlorophyllide a 8-vinyl-reductase (ferredoxin) [EC:1.3.7.13]

K21251	0	1.41622E-07	1.16517E-07	1.68455E-06	macrolide glycosyltransferase
K21252	1.04582E-08	1.99937E-08	3.00273E-08	3.17377E-09	fosfomycin resistance protein FosX
K21253	0	8.73348E-10	6.64408E-08	2.44286E-06	glutathione S-transferase fosA [EC:2.5.1.18]
K21254	6.16096E-09	1.41241E-07	5.40491E-07	1.58978E-07	orsellinic acid synthase [EC:2.3.1.-]
K21255	0	0	6.81974E-08	0	orsellinic acid C2-O-methyltransferase [EC:2.1.1.-]
K21256	1.03837E-09	7.24899E-08	1.09641E-08	2.44509E-09	flavin-dependent halogenase
K21259	0	0	5.76604E-08	0	calicheamicinone 4-hydroxyamino-4,6-dideoxy-alpha-D-glucosyltransferase [EC:2.4.1.-]
K21260	0	0	1.81612E-08	1.78604E-09	calicheamicin 4-deoxy-4-thio-alpha-D-digitoxosyltransferase [EC:2.4.1.-]
K21264	6.99937E-08	2.86917E-07	4.15154E-08	3.07503E-07	glutathione S-transferase fosA2 [EC:2.5.1.18]
K21265	9.39697E-08	2.86917E-07	1.19992E-07	3.38089E-06	glutathione S-transferase fosA5 [EC:2.5.1.18]
K21266	0	0	1.52269E-08	2.98446E-09	beta-lactamase class D OXA-286 [EC:3.5.2.6]
K21267	8.56287E-10	3.20722E-08	7.4747E-07	1.92399E-06	rifampicin monooxygenase [EC:1.14.13.211]
K21271	2.40676E-07	5.90195E-07	2.74082E-06	6.92149E-06	aurachin B dehydrogenase [EC:1.1.1.394]
K21272	1.49862E-07	5.7168E-08	2.33636E-07	2.88255E-08	aurachin C monooxygenase/isomerase [EC:1.14.13.222]
K21273	5.86449E-07	4.11211E-07	7.03545E-06	3.55652E-06	trans,polycis-polyprenyl diphosphate synthase [EC:2.5.1.88]
K21275	0	0	3.27286E-08	1.73885E-08	hexaprenyl-diphosphate synthase large subunit [EC:2.5.1.83]
K21276	0.0002138	0.000211816	0.000186262	0.000113256	beta-lactamase class D OXA-22 [EC:3.5.2.6]
K21277	6.21875E-08	1.17924E-09	2.24332E-08	2.46392E-08	beta-lactamase class D OXA-60 [EC:3.5.2.6]
K21279	0	6.23613E-08	7.97388E-09	0	3-deoxy-D-glycero-D-galacto-nononate 9-phosphate synthase [EC:2.5.1.132]
K21281	0	0	8.84135E-08	6.11582E-09	isobutylamine N-monooxygenase [EC:1.14.14.30]
K21284	0	0	1.06729E-07	0	3-hydroxy-4-methylanthranilate adenylyltransferase [EC:2.7.7.97]
K21285	1.0467E-06	1.19459E-07	3.1473E-06	6.43834E-08	teichoic acid glycerol-phosphate primase [EC:2.7.8.44]
K21288	5.6934E-07	4.35639E-07	1.25024E-06	2.9176E-05	rifampin ADP-ribosylating transferase
K21297	1.33358E-08	6.04244E-09	2.87031E-07	3.48277E-08	flavin reductase (NADH) [EC:1.5.1.36]
K21298	1.04582E-08	1.87982E-09	5.38018E-08	6.9957E-07	1,2-beta-oligoglucan phosphorylase [EC:2.4.1.333]
K21302	2.6973E-08	6.41879E-08	3.76527E-07	1.18262E-07	phosphatidylinositol-3-phosphatase [EC:3.1.3.64]
K21303	7.45817E-06	3.9937E-06	1.54122E-05	1.45256E-05	UDP-GalNAc:undecaprenyl-phosphate GalNAc-1-phosphate transferase [EC:2.7.8.40]
K21304	9.16819E-09	0	0	3.57207E-09	UDP-N-acetylglucosamine---dolichyl-phosphate N-acetylglucosaminyltransferase [EC:2.4.1.153]
K21305	5.54378E-08	0	1.83695E-08	0	dolichyl N-acetyl-alpha-D-glucosaminyl phosphate 3-beta-D-2,3-diacetamido-2,3-dideoxy-beta-D-glucuronosyltransfe
K21307	5.04673E-08	7.24542E-07	2.81061E-06	2.59908E-06	sulfite dehydrogenase (quinone) subunit SoeA [EC:1.8.5.6]
K21308	2.38521E-08	7.08292E-07	7.75272E-07	3.3582E-06	sulfite dehydrogenase (quinone) subunit SoeB
K21309	8.47372E-08	7.484E-07	3.15818E-07	9.47471E-08	sulfite dehydrogenase (quinone) subunit SoeC
K21310	7.7134E-08	9.46647E-08	1.46421E-07	4.29242E-08	methanethiol S-methyltransferase [EC:2.1.1.334]
K21323	2.11983E-07	3.16068E-07	5.58434E-07	4.27643E-07	tert-butyl alcohol monooxygenase / tert-amyl alcohol desaturase [EC:1.14.13.229 1.14.19.48]
K21324	1.54312E-07	8.82181E-08	3.72016E-07	5.46474E-07	tert-butyl alcohol monooxygenase/tert-amyl alcohol desaturase reductase
K21325	3.97169E-09	0	1.4461E-07	7.10032E-07	dTDP-rhamnose C3-O-methyltransferase [EC:2.1.1.-]
K21329	0	0	2.40492E-07	1.78604E-09	aminosugar N-oxygenase [EC:1.14.15.-]
K21332	0	0	1.16286E-08	0	dTDP-alpha-D-glucuronic acid decarboxylase [EC:1.1.1.-]
K21336	8.56287E-10	0	1.9665E-07	1.0991E-09	C-methyltransferase [EC:2.1.1.-]
K21342	0	1.7725E-08	5.28417E-08	0	demethyl-4-deoxygadusol synthase [EC:4.2.3.154]
K21344	0.000214353	0.000212625	0.000188933	0.000119842	D-glycero-beta-D-manno-heptose-7-phosphate kinase [EC:2.7.1.167]
K21345	0.000214064	0.000211969	0.000187421	0.000114035	D-glycero-beta-D-manno-heptose 1-phosphate adenylyltransferase [EC:2.7.7.70]
K21349	3.63164E-08	0	1.3724E-07	1.38356E-08	glucosylglycerate synthase [EC:2.4.1.268]
K21350	0	3.71095E-09	3.37188E-07	1.27133E-07	sucrose 6(F)-phosphate phosphorylase [EC:2.4.1.329]
K21355	2.27728E-09	2.29952E-09	3.75799E-08	1.49762E-09	1,2-alpha-glucosylglycerol phosphorylase [EC:2.4.1.332]
K21363	1.87983E-10	1.40457E-08	1.35374E-09	6.59467E-08	UDP-Glc:alpha-D-GlcNAc-glucosaminyl-diphosphoundecaprenol beta-1,3-glucosyltransferase [EC:2.4.1.305]
K21364	1.95018E-07	4.472E-07	1.6687E-08	8.00354E-07	UDP-Gal:alpha-D-GlcNAc-diphosphoundecaprenol beta-1,4-galactosyltransferase [EC:2.4.1.304]
K21365	3.88931E-11	9.67354E-11	2.80085E-10	5.86252E-09	UDP-GalNAc:alpha-D-GalNAc-diphosphoundecaprenol alpha-1,3-N-acetylgalactosaminyltransferase [EC:2.4.1.306]
K21366	2.65664E-08	6.45036E-07	5.24236E-08	7.1633E-08	N-acetylgalactosaminide beta-1,3-galactosyltransferase [EC:2.4.1.122]
K21367	2.45095E-07	8.67004E-07	1.79723E-06	2.52632E-07	GDP-Fuc:beta-D-Gal-1,3-alpha-D-GalNAc-1,3-alpha-GalNAc-diphosphoundecaprenol alpha-1,2-fucosyltransferase [E
K21377	0	0	9.0419E-08	0	3-hydroxy-5-methyl-1-naphthoate 3-O-methyltransferase [EC:2.1.1.302]
K21378	0	1.02051E-08	1.85576E-08	2.33942E-08	acetoacetyl-CoA synthase [EC:2.3.1.194]
K21379	9.81162E-07	3.48399E-06	2.18348E-06	8.88884E-06	dTDP-4-amino-4,6-dideoxy-D-glucose acyltransferase [EC:2.3.1.209]
K21393	9.66889E-06	1.59397E-05	8.53337E-05	0.000190983	TRAP-type transport system large permease protein
K21394	2.48856E-06	6.056E-06	3.87024E-05	7.15096E-05	TRAP-type transport system small permease protein
K21395	1.15459E-05	1.61611E-05	8.58541E-05	0.000194168	TRAP-type transport system periplasmic protein
K21397	4.88919E-06	1.46466E-05	2.63502E-05	4.24712E-05	ABC transport system ATP-binding/permease protein
K21399	5.60467E-07	2.61814E-07	5.94649E-06	6.84249E-08	2-amino-4-ketopentanoate thiolase alpha subunit [EC:2.3.1.263]
K21400	5.60467E-07	2.61814E-07	5.94649E-06	6.84249E-08	2-amino-4-ketopentanoate thiolase beta subunit [EC:2.3.1.263]
K21401	5.13793E-06	1.04058E-05	2.97586E-05	4.26107E-05	menaquinone-9 beta-reductase [EC:1.3.99.38]
K21402	1.84102E-08	1.22641E-08	2.09314E-08	0	2-O-methyltransferase [EC:2.1.1.-]
K21405	0.002608655	0.002607355	0.00249143	0.002279481	sigma-54 dependent transcriptional regulator, acetoin dehydrogenase operon transcriptional activator AcoR
K21416	2.09551E-06	2.2925E-06	7.7498E-06	9.85553E-06	acetoin:2,6-dichlorophenolindophenol oxidoreductase subunit alpha [EC:1.1.1.-]
K21417	0.000220012	0.00022195	0.000200658	0.000161634	acetoin:2,6-dichlorophenolindophenol oxidoreductase subunit beta [EC:1.1.1.-]
K21420	0.000219205	0.000216819	0.000207715	0.000216387	leucyl-tRNA---protein transferase [EC:2.3.2.29]
K21428	0.000213787	0.000211859	0.000186088	0.000113204	3-acetyloctanal synthase [EC:2.2.1.12]
K21429	0	4.44304E-09	5.64747E-08	1.85764E-06	immunomodulating metalloprotease
K21430	3.8183E-06	7.15684E-06	2.83222E-05	0.000114702	aldose sugar dehydrogenase [EC:1.1.5.-]
K21431	1.1167E-06	6.81166E-07	3.49863E-06	7.19984E-07	alpha-keto-acid decarboxylase [EC:4.1.1.-]
K21440	3.97169E-09	3.97012E-08	2.77051E-06	2.46128E-09	ankyrin repeat domain-containing protein 50
K21449	0.000222807	0.000223599	0.000203983	0.000318765	trimeric autotransporter adhesin
K21453	4.6021E-07	1.29628E-08	5.60932E-08	1.29957E-08	TetR/AcrR family transcriptional regulator, biofilm operon repressor
K21454	1.04703E-06	0	2.75621E-06	3.03626E-08	MarR family transcriptional regulator, teicoplanin-associated locus regulator
K21457	0	0	6.06506E-08	0	phenylpyruvate C(3)-methyltransferase [EC:2.1.1.281]
K21460	0	0	1.27285E-08	0	L-tyrosine C(3)-methyltransferase [EC:2.1.1.304]
K21461	3.17109E-08	0	3.3496E-09	0	poly-beta-1,6-N-acetyl-D-glucosamine synthesis protein
K21462	7.06132E-08	0	1.1399E-07	3.48652E-09	probable poly-beta-1,6-N-acetyl-D-glucosamine export protein
K21463	1.05031E-06	0	2.8318E-06	2.06426E-06	membrane-associated protein TcaA
K21464	3.14949E-06	1.78718E-06	1.18348E-05	5.1127E-06	penicillin-binding protein 2D [EC:2.4.1.129 3.4.16.4]
K21465	1.34629E-06	2.65794E-07	6.78027E-06	3.45813E-06	penicillin-binding protein A
K21466	1.08564E-06	0	3.07862E-06	1.94335E-07	penicillin-binding protein H
K21467	6.12994E-07	1.72377E-07	1.19799E-06	1.003E-05	penicillin-binding protein 3
K21468	4.52323E-07	4.83109E-07	5.56481E-07	2.24727E-08	penicillin-binding protein 4B
K21469	7.96546E-07	1.29304E-06	8.02421E-06	3.75107E-06	serine-type D-Ala-D-Ala carboxypeptidase [EC:3.4.16.4]
K21470	4.31163E-06	2.53831E-06	5.59966E-06	1.61058E-05	L,D-transpeptidase YcbB
K21471	0.000442408	0.000443783	0.000452909	0.000427483	peptidoglycan DL-endopeptidase CwlO [EC:3.4.-.-]
K21472	2.88764E-06	3.27637E-06	6.50558E-06	0.000103577	peptidoglycan LD-endopeptidase LytH [EC:3.4.-.-]

K21473	6.24897E-07	1.1909E-06	8.52281E-06	5.18457E-06	peptidoglycan DL-endopeptidase RipA [EC:3.4.-.-]
K21474	0	8.75822E-07	1.83137E-06	2.17016E-06	peptidoglycan DL-endopeptidase RipB [EC:3.4.-.-]
K21478	9.85127E-08	4.48362E-08	9.47015E-08	2.57841E-07	poly-beta-1,6-N-acetyl-D-glucosamine N-deacetylase [EC:3.5.1.-]
K21479	1.15275E-07	7.35717E-08	2.8219E-06	1.92599E-07	cobalt-factor III methyltransferase [EC:2.1.1.272]
K21480	1.27124E-07	1.67595E-07	8.82177E-07	3.77861E-06	heme oxygenase (biliverdin-producing, ferredoxin) [EC:1.14.15.20]
K21481	2.13472E-06	4.0017E-06	4.97148E-06	1.76981E-06	heme oxygenase (mycobilin-producing) [EC:1.14.99.57]
K21487	1.20362E-10	5.93339E-10	6.61295E-09	1.07593E-07	toxin YobL [EC:3.1.-.-]
K21488	0	0	1.31418E-08	0	antitoxin YobK
K21490	0	0	2.72753E-09	0	antitoxin YokJ
K21493	6.7364E-09	1.31827E-09	3.50848E-08	1.00361E-08	toxin YxiD [EC:3.1.-.-]
K21494	1.2654E-09	0	7.21284E-09	0	antitoxin YxxD
K21495	1.02457E-06	2.19405E-06	6.80444E-06	1.47812E-05	antitoxin FitA
K21498	5.32552E-06	8.18651E-06	3.50651E-05	0.000128322	antitoxin HigA-1
K21507	3.56854E-10	8.87571E-10	5.34933E-09	1.05708E-08	methyltransferase [EC:2.1.1.-]
K21511	6.76725E-07	6.53071E-07	3.13704E-06	9.12859E-06	capsid assembly protease [EC:3.4.21.-]
K21512	1.13248E-06	1.39399E-06	6.25188E-06	2.50695E-05	terminase, large subunit [EC:3.1.21.4]
K21514	0	0	2.45376E-07	2.2716E-08	23S rRNA (uridine2479-2-O)-methyltransferase [EC:2.1.1.208]
K21527	9.16042E-09	3.27693E-08	5.47052E-09	1.73717E-08	adenine modification enzyme [EC:2.3.1.-]
K21528	5.05642E-08	3.65214E-08	5.02629E-07	1.63111E-06	serine recombinase [EC:3.1.22.- 6.5.1.-]
K21555	2.04795E-08	2.01859E-08	3.95736E-09	7.05394E-09	CRP/FNR family transcriptional regulator, carbon monoxide oxidation system transcription regulator
K21556	1.70403E-07	7.03627E-07	2.02081E-06	5.54947E-09	CRP/FNR family transcriptional regulator, polysaccharide utilization system transcription regulator
K21557	3.15324E-07	3.92306E-07	6.52347E-07	5.8098E-09	HTH-type transcriptional regulator, polysaccharide utilization system transcription regulator
K21560	9.34164E-09	0	0	0	CRP/FNR family transcriptional regulator, listeriolysin regulatory protein
K21562	1.09752E-06	3.144E-06	1.16719E-06	6.8722E-07	CRP/FNR family transcriptional regulator, anaerobic regulatory protein
K21563	0.000215216	0.000216101	0.000203342	0.00014965	CRP/FNR family transcriptional regulator, dissimilatory nitrate respiration regulator
K21564	5.16343E-07	1.48157E-07	7.72765E-07	2.30991E-07	CRP/FNR family transcriptional regulator, nitrogen oxide reductase regulator
K21567	3.09889E-06	4.55602E-06	1.7453E-05	3.99757E-05	ferredoxin/flavodoxin---NADP+ reductase [EC:1.18.1.2 1.19.1.1]
K21568	0	0	1.74429E-08	0	pinoresinol/lariciresinol reductase [EC:1.23.1.1 1.23.1.2 1.23.1.3 1.23.1.4]
K21571	8.01051E-07	2.11518E-06	8.23497E-06	6.36031E-08	starch-binding outer membrane protein SusE/F
K21572	1.49581E-05	5.44467E-05	6.57028E-05	3.77754E-06	starch-binding outer membrane protein, SusD/RagB family
K21573	1.54384E-05	5.7549E-05	7.8758E-05	1.62059E-06	TonB-dependent starch-binding outer membrane protein SusC
K21574	1.76086E-06	2.75144E-06	7.39423E-06	4.20964E-05	glucan 1,4-alpha-glucosidase [EC:3.2.1.3]
K21575	9.11178E-07	1.57114E-06	2.13097E-06	8.73475E-06	neopullulanase [EC:3.2.1.135]
K21576	9.74318E-08	7.25515E-11	1.78331E-07	1.35977E-08	glycine/sarcosine/betaine reductase complex component C subunit alpha [EC:1.21.4.2 1.21.4.3 1.21.4.4]
K21577	9.74545E-08	1.2898E-10	1.78494E-07	1.42698E-08	glycine/sarcosine/betaine reductase complex component C subunit beta [EC:1.21.4.2 1.21.4.3 1.21.4.4]
K21578	0	0	1.78121E-07	1.27337E-08	betaine reductase complex component B subunit alpha [EC:1.21.4.4]
K21579	9.74027E-08	0	2.67181E-07	3.8201E-08	betaine reductase complex component B subunit beta [EC:1.21.4.4]
K21583	9.74318E-08	7.25515E-11	1.78331E-07	2.63314E-08	sarcosine reductase complex component B subunit alpha [EC:1.21.4.3]
K21584	9.74027E-08	0	1.78121E-07	1.27337E-08	sarcosine reductase complex component B subunit beta [EC:1.21.4.3]
K21591	4.3068E-08	2.44367E-08	1.6748E-09	1.07162E-08	teichoic acid glycerol-phosphate transferase [EC:2.7.8.45]
K21592	1.04582E-08	1.43137E-07	2.75737E-10	2.54375E-09	teichoic acid ribitol-phosphate primase [EC:2.7.8.46]
K21600	6.56876E-06	9.86629E-06	2.10849E-05	6.4339E-05	CsoR family transcriptional regulator, copper-sensing transcriptional repressor
K21601	0	6.64661E-08	1.25252E-07	1.27188E-09	DeoR family transcriptional regulator, copper-sensing transcriptional repressor
K21602	5.33837E-08	2.20074E-07	1.41489E-07	3.57133E-07	IclR family transcriptional regulator, carbohydrate utilization repressor
K21603	9.91739E-07	1.78817E-07	1.08586E-07	3.57629E-08	two-component system, OmpR family, flagellar system response regulator FtcR
K21606	1.41197E-07	3.16944E-07	7.65014E-07	3.56634E-05	endo-chitodextinase [EC:3.2.1.202]
K21607	4.49876E-06	1.10227E-05	4.69144E-05	0.00011827	tetrachlorobenzoquinone reductase [EC:1.1.1.404]
K21608	0	1.06439E-08	0	3.7621E-08	lipoprotein LpqS
K21609	0	0	0	2.08829E-08	metallothionein
K21610	2.36749E-07	1.83274E-08	2.75737E-10	3.98511E-08	Ni-sirohydrochlorin a,c-diamide reductive cyclase subunit CfbC [EC:6.3.3.7]
K21613	0.000441614	0.000440958	0.000428744	0.000373995	uncharacterized hydrolase [EC:3.-.-.-]
K21614	0	0	5.81429E-09	0	L-proline cis-4-hydroxylase [EC:1.14.11.56]
K21616	4.04966E-08	8.61351E-08	2.81312E-08	2.54375E-09	D-altritol 5-dehydrogenase [EC:1.1.1.407]
K21617	4.96461E-09	8.50664E-11	4.76771E-08	7.14415E-09	4-hydroxyproline betaine 2-epimerase [EC:5.1.1.22]
K21619	4.23211E-08	0	9.54637E-09	1.1358E-08	tagaturonate epimerase [EC:5.1.2.7]
K21620	1.52861E-06	6.75011E-07	3.84919E-06	4.02103E-05	galactitol 2-dehydrogenase [EC:1.1.1.16]
K21621	1.5663E-06	3.15466E-06	1.41355E-05	3.5309E-05	tagatose kinase [EC:2.7.1.101]
K21622	1.35421E-06	3.34726E-06	1.41984E-05	3.76213E-05	D-tagatose 6-phosphate 4-epimerase [EC:5.1.3.40]
K21624	2.95189E-08	7.41419E-09	2.79141E-07	1.71157E-08	cis-L-3-hydroxyproline dehydratase [EC:4.2.1.171]
K21636	0.000432563	0.000427147	0.000398063	0.000249811	ribonucleoside-triphosphate reductase (formate) [EC:1.1.98.6]
K21637	4.29029E-09	1.81542E-08	3.08962E-08	1.91792E-07	anti-adapter protein IraD
K21638	2.52239E-08	5.40311E-09	3.94021E-08	6.22439E-07	anti-adapter protein IraM
K21645	0.000223384	0.000233811	0.00029708	0.000390195	LysR family transcriptional regulator, hypochlorite-specific transcription factor HypT
K21672	5.09584E-07	1.59962E-06	6.77811E-06	4.03219E-06	2,4-diaminopentanoate dehydrogenase [EC:1.4.1.12 1.4.1.26]
K21676	4.85323E-07	1.18369E-06	5.4956E-06	1.3843E-05	lupanine 17-hydroxylase (cytochrome c) [EC:1.17.2.2]
K21677	1.45651E-06	1.24447E-07	1.22752E-06	2.07706E-07	hydroxysqualene dehydroxylase [EC:1.17.8.1]
K21678	1.33659E-06	1.03131E-07	3.83156E-07	5.53545E-08	presqualene diphosphate synthase [EC:2.5.1.103]
K21679	2.56115E-06	3.01439E-06	1.48083E-05	3.4834E-05	hydroxysqualene synthase [EC:4.2.3.156]
K21680	5.58266E-10	3.05459E-09	0	0	ribulose-5-phosphate 2-dehydrogenase [EC:1.1.1.137]
K21681	2.35591E-07	5.13014E-07	1.00548E-07	7.08656E-08	ribitol-5-phosphate 2-dehydrogenase (NADP+) / D-ribitol-5-phosphate cytidylyltransferase [EC:1.1.1.405 2.7.7.40]
K21684	1.44434E-10	1.847E-10	1.53613E-07	0	salicyloyl-CoA 5-hydroxylase [EC:1.14.13.209]
K21685	1.15218E-07	9.92934E-07	3.7394E-06	8.54686E-06	LuxR family transcriptional regulator, regulator of acetate metabolism
K21686	3.04577E-06	4.39182E-06	1.92066E-05	8.9868E-05	XRE family transcriptional regulator, fatty acid utilization regulator
K21687	2.01314E-06	6.21275E-06	9.65091E-06	6.28202E-05	resuscitation-promoting factor RpfA
K21688	1.46284E-06	3.3471E-06	1.91243E-05	1.41352E-05	resuscitation-promoting factor RpfB
K21689	0	2.83147E-07	1.61769E-07	3.50999E-06	resuscitation-promoting factor RpfC
K21690	0	1.06439E-08	0	2.26111E-09	resuscitation-promoting factor RpfD
K21691	8.99101E-09	1.22601E-06	2.58459E-06	7.9889E-06	resuscitation-promoting factor RpfE
K21694	2.04795E-08	9.59746E-09	3.67344E-08	7.05394E-09	N(2)-fixation sustaining protein CowN
K21695	6.11197E-07	6.61335E-07	1.04744E-06	3.35048E-05	protein AaeX
K21696	1.20362E-10	1.72795E-08	5.95036E-08	0	LytTR family transcriptional regulator, CO-responsive transcriptional regulator RcoM
K21697	3.99601E-09	7.63099E-08	0	0	LuxR family transcriptional regulator, quorum-sensing system regulator VjbR
K21698	0.000217501	0.0002186	0.000212252	0.000244584	LysR family transcriptional regulator, transcriptional activator for aaeXAB operon
K21699	0.000218014	0.000221717	0.000232226	0.000221199	LysR family transcriptional regulator, transcriptional activator for bauABCD operon
K21700	1.00244E-06	7.09048E-07	1.71928E-07	1.49107E-07	beta-alanine degradation protein BauB
K21701	6.14082E-07	1.17812E-06	4.60167E-06	3.4083E-06	AraC family transcriptional regulator, transcriptional activator for feuABC-ybbA operon
K21702	0	3.8155E-08	1.74429E-08	0	galactitol 2-dehydrogenase (L-tagatose-forming) [EC:1.1.1.406]

K21703	0.001737643	0.001736823	0.001682833	0.001539329	LysR family transcriptional regulator, low CO2-responsive transcriptional regulator
K21711	1.51759E-06	4.37118E-06	1.6044E-05	4.02897E-05	LysR family transcriptional regulator, putative pyruvate carboxylase regulator
K21712	1.44101E-07	4.7745E-07	6.72478E-07	7.88282E-06	N-acetylglucosamine-binding protein A
K21713	1.23169E-08	1.14316E-08	3.53658E-07	2.03044E-06	lytic chitin monoxygenase [EC:1.14.99.53]
K21721	0.000427609	0.000423613	0.000372878	0.000226424	N-[(2S)-2-amino-2-carboxyethyl]-L-glutamate dehydrogenase [EC:1.5.1.51]
K21722	0	1.01891E-08	3.81567E-08	3.34137E-09	methylxanthine N1-demethylase [EC:1.14.13.178]
K21723	0	1.01891E-08	3.81567E-08	3.34137E-09	methylxanthine N3-demethylase [EC:1.14.13.179]
K21724	7.94338E-09	5.68328E-08	1.27453E-07	3.34137E-09	7-methylxanthine demethylase [EC:1.14.13.128]
K21725	3.30974E-09	3.74292E-09	6.81884E-08	0	4-nitrocatechol/4-nitrophenol 4-monooxygenase [EC:1.14.13.166 1.14.13.167]
K21726	0	0	6.45236E-10	6.35938E-10	4-nitrophenol 2-monooxygenase / 4-nitrocatechol 4-monooxygenase, oxygenase component [EC:1.14.13.29 1.14.13.16
K21727	0	1.70133E-10	2.17868E-07	1.78604E-09	4-nitrophenol 2-monooxygenase / 4-nitrocatechol 4-monooxygenase, reductase component
K21728	0	0	4.65143E-09	0	(S)-dichlorprop dioxygenase (2-oxoglutarate) [EC:1.14.11.43]
K21730	6.24832E-08	2.14726E-08	9.30027E-08	5.57976E-07	(2,2,3-trimethyl-5-oxocyclopent-3-enyl)acetyl-CoA 1,5-monooxygenase [EC:1.14.13.160]
K21731	1.59354E-08	0	8.2995E-09	4.15646E-09	2,5-diketocamphane 1,2-monooxygenase [EC:1.14.13.162]
K21738	0	0	1.18611E-08	8.17748E-09	rubredoxin---NAD+ reductase [EC:1.18.1.1]
K21739	2.2623E-06	2.18477E-06	1.13295E-05	4.52843E-06	probable pyridine nucleotide-disulfide oxidoreductase
K21740	5.46934E-11	1.36034E-10	3.93869E-10	1.62014E-09	reactive chlorine resistance protein B
K21741	3.29507E-07	9.21469E-07	4.13531E-07	2.33471E-07	reactive chlorine resistance protein C
K21742	2.43929E-07	4.12936E-07	2.91532E-07	2.08551E-06	LysR family transcriptional regulator, transcriptional activator for ttdABT operon
K21743	1.02721E-08	2.78321E-09	2.17139E-07	5.08605E-07	MerR family transcriptional regulator, multidrug-efflux activator
K21744	1.58683E-06	2.07178E-06	5.89008E-06	7.41549E-06	MerR family transcriptional regulator, thiopeptide resistance regulator
K21745	1.98493E-07	1.20836E-07	4.01903E-07	4.41885E-07	MerR family transcriptional regulator, aldehyde-responsive regulator
K21746	3.16564E-08	1.29802E-07	5.0604E-08	1.93355E-07	AraC family transcriptional regulator, reactive chlorine species (RCS)-specific activator of rcl operon
K21747	1.84405E-06	9.47166E-07	3.42724E-06	4.44687E-05	AraC family transcriptional regulator, alkane utilization regulator
K21748	0	0	1.18611E-08	8.17748E-09	LuxR family transcriptional regulator, alkane degradation pathway regulator
K21749	1.19191E-08	1.2258E-07	7.70465E-08	4.01858E-09	N-acylneuraminate/3-deoxy-D-glycero-D-galacto-nononate cytidylyltransferase [EC:2.7.7.43 2.7.7.92]
K21755	0.000217845	0.000220058	0.000219778	0.000228619	LysR family transcriptional regulator, salicylic acid-responsive activator of bsdBCD
K21756	0	7.15054E-07	6.45236E-09	6.92573E-09	LysR family transcriptional regulator, cis,cis-muconate-responsive activator of cat and ben genes
K21757	0.001733791	0.001728955	0.001652055	0.001538659	LysR family transcriptional regulator, benzoate and cis,cis-muconate-responsive activator of ben and cat genes
K21758	0	1.44028E-09	3.58921E-08	2.3566E-08	benzoate transport porin
K21759	8.55673E-08	9.1658E-08	1.64042E-07	5.42788E-07	vanillate/4-hydroxybenzoate decarboxylase subunit D [EC:4.1.1.- 4.1.1.61]
K21778	0.000214104	0.00021188	0.000186131	0.000113204	3-acetyloctanal aminotransferase [EC:2.6.1.-]
K21779	2.11057E-08	8.50389E-08	6.04288E-09	1.47893E-09	2-methyl-3-n-amyl-dihydropyrrolel dehydrogenase
K21780	7.43254E-07	1.91294E-06	8.67879E-06	2.07832E-05	L-proline---[L-prolyl-carrier protein] ligase [EC:6.2.1.53]
K21781	2.11057E-08	8.50389E-08	6.04288E-09	3.26497E-09	peptidyl carrier protein
K21782	3.43066E-07	2.17491E-07	3.36734E-06	8.85308E-08	L-prolyl-PCP dehydrogenase [EC:1.3.8.14]
K21783	2.11057E-08	8.50389E-08	2.42041E-08	3.26497E-09	beta-ketoacyl ACP synthase [EC:2.3.1.-]
K21784	2.11057E-08	8.50389E-08	4.4701E-08	7.95331E-09	4-hydroxy-2,2-bipyrrole-5-methanol synthase
K21785	2.11057E-08	8.50389E-08	6.04288E-09	3.26497E-09	4-hydroxy-2,2-bipyrrole-5-methanol dehydrogenase
K21786	2.11057E-08	8.50389E-08	6.04288E-09	1.47893E-09	4-hydroxy-2,2-bipyrrole-5-carbaldehyde O-methyltransferase [EC:2.1.1.-]
K21787	2.11057E-08	1.98336E-07	4.85727E-08	4.03672E-07	prodigiosin/undecylprodigiosin synthetase
K21789	0	0	0	1.78604E-09	dodecanoy-ACP synthase
K21792	2.64796E-10	3.38617E-10	0	1.78604E-09	polyketide synthase
K21795	0.000213766	0.000211774	0.000186082	0.000113202	phenylalanine 2-monooxygenase [EC:1.13.12.9]
K21801	9.60198E-07	1.59281E-06	7.21747E-06	1.86461E-05	indoleacetamide hydrolase [EC:3.5.1.-]
K21802	0.000430255	0.000428536	0.000395955	0.000323599	vanillin dehydrogenase [EC:1.2.1.67]
K21814	0	0	4.82911E-07	0	serine/threonine-protein phosphatase CPPED1 [EC:3.1.3.16]
K21815	0.000213765	0.000211774	0.000186082	0.000113202	2-oxoglutarate dioxygenase / 2-oxoglutarate/L-arginine monooxygenase/decarboxylase [EC:1.13.12.19 1.14.20.7]
K21817	1.34865E-07	1.17924E-09	2.32571E-08	1.05673E-08	beta-carotene 15,15-dioxygenase [EC:1.13.11.63]
K21822	9.73043E-07	5.97605E-09	2.04064E-07	2.48083E-08	8-apo-carotenoid 13,14-cleaving dioxygenase [EC:1.13.11.82]
K21825	2.3032E-07	5.56692E-08	1.47479E-06	4.83669E-06	AraC family transcriptional regulator, L-arginine-responsive activator
K21826	2.78307E-06	7.81411E-07	4.41396E-06	6.54605E-06	AraC family transcriptional regulator, glycine betaine-responsive activator
K21827	5.39461E-08	2.83838E-08	4.63102E-08	7.59085E-07	IclR family transcriptional regulator, arginine deiminase pathway regulator
K21828	9.34097E-07	1.83275E-08	2.78002E-06	2.95881E-06	CRP/FNR family transcriptional regulator, arginine deiminase pathway regulator
K21829	1.32941E-07	4.06308E-07	1.11095E-06	2.57249E-07	D-arginine utilization repressor
K21830	1.41986E-07	2.5079E-08	2.33909E-07	7.97387E-07	phosphorylcholine phosphatase
K21831	1.52803E-08	2.18337E-09	2.04562E-07	3.66225E-08	cholinesterase
K21832	1.52122E-06	2.74683E-07	1.9027E-06	9.69443E-06	glycine betaine catabolism B
K21833	2.99775E-06	1.43908E-06	4.30272E-06	9.16455E-06	dimethylglycine catabolism A
K21834	4.09877E-07	1.43062E-06	3.53019E-06	3.10518E-06	dimethylglycine catabolism B
K21883	0.000433938	0.000431203	0.000409761	0.00036451	2-dehydro-3-deoxy-L-rhamnonate dehydrogenase (NAD+) [EC:1.1.1.401]
K21884	0	0	6.97714E-09	5.18522E-08	CRP/FNR family transcriptional regulator, cAMP and macrophage regulator
K21885	1.3045E-06	5.23477E-06	4.50204E-06	5.27772E-06	ArsR family transcriptional regulator, cadmium/lead-responsive transcriptional repressor
K21886	0	1.23941E-07	1.95419E-07	5.69368E-07	ArsR family transcriptional regulator, nickel/cobalt-responsive transcriptional repressor
K21887	2.95302E-06	5.94841E-06	1.8919E-06	2.73084E-06	cation-transporting P-type ATPase J [EC:3.6.3.-]
K21898	9.51378E-07	1.31519E-06	6.36475E-06	1.78706E-06	ornithine racemase [EC:5.1.1.12]
K21900	6.00635E-06	8.29461E-06	3.76308E-05	0.000129095	LysR family transcriptional regulator, transcriptional activator of the cysJI operon
K21901	4.37497E-09	1.83648E-08	3.15059E-08	1.943E-07	LuxR family transcriptional regulator, dicarboxylate transport regulator
K21902	6.49892E-07	1.18546E-06	9.98404E-07	2.35989E-07	MerR family transcriptional regulator, repressor of the yfmOP operon
K21903	6.35342E-06	7.639E-06	1.28708E-05	1.49322E-05	ArsR family transcriptional regulator, lead/cadmium/zinc/bismuth-responsive transcriptional repressor
K21904	2.13382E-06	9.81438E-07	3.7583E-06	4.83488E-05	metallothionein
K21905	4.37821E-09	1.83729E-08	3.15293E-08	1.94396E-07	AraC family transcriptional regulator, glutamate-dependent acid resistance regulator
K21906	5.6375E-09	2.1505E-08	4.05979E-08	2.31699E-07	AraC family transcriptional regulator, glutamate-dependent acid resistance regulator
K21907	4.35552E-09	1.83165E-08	3.13659E-08	1.93724E-07	LuxR family transcriptional regulator, glutamate-dependent acid resistance regulator
K21908	9.81992E-08	2.99822E-07	5.13925E-08	3.08143E-07	membrane protein HdeD
K21909	2.91698E-11	1.1555E-07	7.2173E-08	8.64073E-10	D-erythrulose 1-phosphate 3-epimerase [EC:5.1.3.38]
K21910	6.5562E-08	1.27346E-07	1.57733E-07	6.51762E-07	L-erythrulose 1-phosphate isomerase [EC:5.3.1.33]
K21911	1.59383E-07	2.91606E-07	1.99843E-07	3.53908E-07	D-erythrulose 4-phosphate isomerase [EC:5.3.1.34]
K21929	0.000224228	0.000223172	0.000243645	0.000260722	uracil-DNA glycosylase [EC:3.2.2.27]
K21935	0	2.13623E-08	4.25471E-08	0	beta-lysine N6-acetyltransferase [EC:2.3.1.264]
K21936	0.000220536	0.000222011	0.000225449	0.000215736	anaerobilin synthase [EC:2.1.1.342]
K21947	6.32744E-07	7.38274E-07	3.51575E-06	6.37433E-08	tRNA-5-methyluridine54 2-sulfurtransferase [EC:2.8.1.15]
K21948	2.70247E-06	3.75018E-06	1.4558E-05	4.11633E-05	3-dehydrotetronate 4-kinase [EC:2.7.1.217]
K21949	0.000643166	0.000639113	0.000570702	0.000341593	N-(2-amino-2-carboxyethyl)-L-glutamate synthase [EC:2.5.1.140]
K21959	1.15926E-06	1.63172E-07	4.49792E-07	1.59785E-07	LysR family transcriptional regulator, cell division regulator
K21960	1.20795E-07	1.93017E-07	5.59714E-07	7.58809E-06	LysR family transcriptional regulator, regulator of the ytmI operon
K21961	8.41161E-07	4.32355E-07	8.42831E-07	2.70044E-07	TetR/AcrR family transcriptional regulator, ethionamide resistance regulator

K21962	5.36338E-07	6.42536E-07	1.71826E-06	1.82466E-06	TetR/AcrR family transcriptional regulator, transcriptional repressor of aconitase
K21963	2.79394E-08	1.7341E-08	2.85417E-08	6.30718E-07	LuxR family transcriptional regulator, Mat/Ecp fimbriae transcriptional regulator
K21964	7.14028E-08	1.44091E-07	2.40886E-07	3.6892E-06	Mat/Ecp fimbriae major subunit
K21965	7.14125E-08	1.44115E-07	2.96913E-07	5.4862E-06	Mat/Ecp fimbriae periplasmic chaperone
K21966	2.54269E-07	2.66048E-07	7.16402E-07	1.65761E-05	Mat/Ecp fimbriae outer membrane usher protein
K21967	7.13375E-08	1.44074E-07	2.41283E-07	3.76174E-06	Mat/Ecp fimbriae adhesin
K21968	7.14125E-08	1.44115E-07	3.04447E-07	6.0854E-06	Mat/Ecp fimbriae periplasmic chaperone
K21970	1.1988E-08	0	0	0	5-methylcytosine rRNA methyltransferase NSUN4 [EC:2.1.1.-]
K21972	5.58326E-08	3.98711E-08	1.31622E-07	1.31789E-06	MerR family transcriptional regulator, repressor of blue light- and temperature-responsive genes
K21973	3.69353E-06	3.7767E-06	1.83381E-05	0.000118372	blue light- and temperature-responsive anti-repressor
K21974	2.8014E-08	1.98262E-08	4.67111E-08	7.48954E-07	probable RcsB/C two-component-system connector
K21975	2.3976E-08	1.14976E-09	2.82865E-08	5.74139E-07	probable RcsB/C two-component-system connector
K21976	2.92699E-08	2.6225E-08	6.641E-08	9.86254E-07	probable RcsB/C two-component-system connector, global regulator of biofilm formation and acid-resistance
K21977	2.00805E-07	2.99845E-07	3.27309E-07	3.49474E-06	phosphopantothenate---cysteine ligase (CTP) [EC:6.3.2.5]
K21990	1.26817E-06	3.37662E-07	3.27205E-07	9.17176E-07	formate-nitrite transporter family protein
K21992	0	1.13298E-07	1.21627E-07	6.06392E-08	lysine N-acyltransferase [EC:2.3.1.-]
K21993	1.03761E-06	1.01884E-06	8.47267E-06	1.62362E-05	formate transporter
K22003	2.98745E-06	3.59412E-06	1.63997E-05	3.53423E-05	aconitate Delta-isomerase [EC:5.3.3.7]
K22010	0.000221588	0.000223004	0.000224597	0.000216963	two-component system, response regulator PdtaR
K22012	0	0	1.06547E-08	0	Ni-sirohydrochlorin a,c-diamide synthase [EC:6.3.5.12]
K22014	1.19542E-08	4.49875E-08	1.04086E-07	4.36393E-07	terminase small subunit
K22015	8.75334E-07	9.37644E-08	1.47815E-06	7.67419E-06	formate dehydrogenase (acceptor) [EC:1.17.99.7]
K22024	5.02416E-06	7.51111E-06	3.71724E-05	7.24078E-05	4-phospho-D-threonate 3-dehydrogenase / 4-phospho-D-erythronate 3-dehydrogenase [EC:1.1.1.408 1.1.1.409]
K22025	3.26742E-06	6.12911E-06	1.60954E-05	3.91756E-05	D-erythronate 2-dehydrogenase [EC:1.1.1.410]
K22026	0	0	2.45589E-08	0	nucleoside kinase [EC:2.7.1.73 2.7.1.213 2.7.1.-]
K22027	1.97618E-07	4.84627E-07	1.65504E-06	4.65394E-06	indole-3-acetate monooxygenase [EC:1.14.13.235]
K22033	3.50005E-08	0	4.77419E-07	3.57207E-09	lytic cellulose monooxygenase (C4-dehydrogenating) [EC:1.14.99.56]
K22041	7.45591E-07	1.27869E-06	1.78303E-06	2.07552E-05	TetR/AcrR family transcriptional regulator, copper-responsive repressor
K22042	6.33646E-06	8.18623E-06	3.12152E-05	0.000133353	ArsR family transcriptional regulator, virulence genes transcriptional regulator
K22043	1.96525E-06	3.59626E-07	4.41352E-06	2.91187E-06	ArsR family transcriptional regulator, zinc-responsive transcriptional repressor
K22044	0.000217167	0.000214338	0.00020187	0.000126294	moderate conductance mechanosensitive channel
K22051	0.00021536	0.00021572	0.000202006	0.000157059	miniconductance mechanosensitive channel
K22067	0.000216361	0.000215048	0.00020438	0.000149371	two-component system, oxyanion-binding sensor
K22071	1.19394E-08	0	1.54415E-07	0	ferredoxin-2, mitochondrial
K22077	0	5.02175E-08	6.97714E-09	0	ganglioside-induced differentiation-associated protein 1
K22078	0	5.11004E-09	6.37754E-08	0	protein-glucosylgalactosylhydroxylysine glucosidase [EC:3.2.1.107]
K22081	4.48334E-07	2.06742E-07	4.43878E-07	2.34244E-07	methylamine---glutamate N-methyltransferase subunit A [EC:2.1.1.21]
K22082	4.50611E-07	2.09042E-07	4.39993E-07	2.34244E-07	methylamine---glutamate N-methyltransferase subunit B [EC:2.1.1.21]
K22083	1.38702E-06	3.12262E-06	1.02629E-06	3.05082E-06	methylamine---glutamate N-methyltransferase subunit C [EC:2.1.1.21]
K22084	0.000214357	0.0002119	0.000187331	0.000114492	methylglutamate dehydrogenase subunit A [EC:1.5.99.5]
K22085	4.30688E-07	6.81339E-10	1.22796E-07	1.64397E-08	methylglutamate dehydrogenase subunit B [EC:1.5.99.5]
K22086	4.37307E-07	1.61864E-07	5.60418E-07	8.88623E-07	methylglutamate dehydrogenase subunit C [EC:1.5.99.5]
K22087	4.30688E-07	6.81339E-10	1.00206E-07	1.64397E-08	methylglutamate dehydrogenase subunit D [EC:1.5.99.5]
K22103	2.55439E-07	5.57763E-08	3.26573E-06	4.61932E-06	DeoR family transcriptional regulator, carbon catabolite repression regulator
K22104	3.36993E-08	4.15321E-07	2.56377E-07	7.78639E-07	GntR family transcriptional regulator, sialic acid-inducible nan operon repressor
K22105	1.02211E-06	1.08108E-06	3.49247E-06	7.12316E-05	TetR/AcrR family transcriptional regulator, fatty acid biosynthesis regulator
K22106	5.89134E-08	2.82444E-07	4.74218E-07	1.1739E-07	TetR/AcrR family transcriptional regulator, repressor of fatR-cypB operon
K22107	4.13271E-07	4.68376E-07	5.50526E-07	7.12915E-06	TetR/AcrR family transcriptional regulator, cholesterol catabolism regulator
K22108	8.01686E-08	3.287E-07	6.71648E-07	6.22759E-06	TetR/AcrR family transcriptional regulator, cholesterol catabolism regulator
K22109	1.1242E-06	0	2.90782E-06	3.03626E-08	HTH-type transcriptional regulator, glycine betaine synthesis regulator
K22110	3.18163E-08	5.30292E-08	1.19574E-07	4.04062E-06	oligogalacturonate-specific porin family protein
K22111	1.58525E-08	4.69119E-08	1.1416E-07	5.3429E-07	9-O-acetyl-N-acetylneuraminic acid deacetylase
K22112	1.65557E-06	3.10606E-06	1.39961E-05	3.6341E-05	putative amide transporter protein
K22113	9.62893E-11	1.23133E-10	0	5.29948E-10	fumarate---(S)-2,3-diaminopropanoate ligase [EC:6.3.2.46]
K22116	1.24618E-06	1.80759E-07	3.11756E-06	6.62022E-08	gamma-polyglutamate biosynthesis protein CapC
K22129	7.78517E-07	6.04741E-07	3.0339E-06	2.22641E-06	D-threonate/D-erythronate kinase [EC:2.7.1.219 2.7.1.220]
K22130	2.52969E-06	4.029E-06	1.45924E-05	4.11429E-05	3-dehydro-4-phosphotetronate decarboxylase [EC:4.1.1.104]
K22131	0.000433052	0.000431464	0.000406592	0.000309687	2-dehydrotetronate isomerase [EC:5.3.1.35]
K22132	0.000221743	0.000219644	0.000218779	0.000237593	tRNA threonylcarbamoyladenosine dehydratase
K22135	1.13037E-06	2.32304E-07	3.43969E-06	5.52335E-08	N-acetylglucosamine malate deacetylase 2 [EC:3.5.1.-]
K22136	1.15646E-06	9.68694E-07	3.61484E-06	6.33538E-08	bacillithiol synthase
K22158	0	0	0	1.4263E-08	F420H2 dehydrogenase subunit A [EC:1.5.98.3]
K22159	0	0	5.8993E-09	0	F420H2 dehydrogenase subunit B [EC:1.5.98.3]
K22161	1.78726E-08	4.99842E-09	0	0	F420H2 dehydrogenase subunit D [EC:1.5.98.3]
K22163	0	5.02175E-08	2.09314E-08	0	F420H2 dehydrogenase subunit H [EC:1.5.98.3]
K22165	0	0	1.47223E-07	0	F420H2 dehydrogenase subunit J [EC:1.5.98.3]
K22166	0	0	1.13561E-08	2.035E-09	F420H2 dehydrogenase subunit K [EC:1.5.98.3]
K22167	1.70403E-07	7.79937E-07	6.44059E-07	1.60743E-08	F420H2 dehydrogenase subunit L [EC:1.5.98.3]
K22168	9.16819E-09	3.06768E-09	7.32079E-08	2.18663E-08	F420H2 dehydrogenase subunit M [EC:1.5.98.3]
K22169	6.27458E-08	7.8383E-07	5.64595E-08	7.43882E-09	F420H2 dehydrogenase subunit N [EC:1.5.98.3]
K22179	0	0	1.94645E-07	7.76174E-08	F420H2:quinone oxidoreductase subunit L [EC:1.1.98.4]
K22185	0.000222066	0.000221413	0.00022636	0.000205092	D-xylose 1-dehydrogenase [EC:1.1.1.175]
K22186	7.31319E-06	6.74061E-06	3.32794E-05	7.13252E-05	xylonate dehydratase [EC:4.2.1.82]
K22187	0.000655677	0.000661789	0.00064055	0.000620193	alpha-ketoglutaric semialdehyde dehydrogenase [EC:1.2.1.-]
K22199	0	0	0	2.28209E-08	phosphopentomutase [EC:5.4.2.7]
K22205	2.02785E-07	7.63653E-07	8.06853E-07	5.64678E-06	S-adenosyl-L-methionine hydroxide adenosyltransferase [EC:2.5.1.-]
K22206	7.41428E-10	9.48128E-10	4.30157E-09	6.35938E-10	XRE family transcriptional regulator, thiamine biosynthesis regulator
K22209	1.13885E-06	1.93611E-06	1.12355E-05	2.11112E-05	D(-)-tartrate dehydratase [EC:4.2.1.81]
K22212	1.2215E-06	9.23966E-07	1.23274E-05	2.99132E-06	malolactic enzyme [EC:4.1.1.101]
K22213	3.40525E-07	4.31248E-07	3.08812E-07	2.46593E-07	6-methylsalicylate decarboxylase [EC:4.1.1.52]
K22214	2.83558E-07	9.97998E-07	2.08339E-06	2.23777E-07	propionyl-CoA:succinyl-CoA transferase [EC:2.8.3.-]
K22215	0.000216375	0.000212628	0.000190187	0.000118075	galactose dehydrogenase [EC:1.1.1.48 1.1.1.120]
K22216	4.59498E-07	7.98754E-07	6.99975E-06	8.44274E-06	dehydratase ilvD1
K22217	2.45555E-07	2.12615E-07	3.99874E-07	7.49569E-06	sugar lactone lactonase
K22219	4.7873E-06	6.21999E-06	3.46968E-05	7.1443E-05	dibenzothiophene monooxygenase [EC:1.14.14.21]
K22220	1.15659E-06	4.67524E-07	6.47712E-07	2.14568E-06	dibenzothiophene sulfone monooxygenase [EC:1.14.14.22]
K22223	1.05037E-06	2.75169E-07	3.29204E-06	8.38671E-07	phosphoglycolate phosphatase [EC:3.1.3.18]

K22224	7.89632E-06	1.6605E-05	6.35516E-05	0.000163296	acetate---CoA ligase (ADP-forming) subunit beta [EC:6.2.1.13]
K22225	0	2.78321E-09	3.42385E-09	0	siroheme decarboxylase [EC:4.1.1.111]
K22226	1.9919E-07	4.2838E-07	3.5203E-07	9.09549E-08	Fe-coproporphyrin III synthase
K22227	1.09997E-06	1.37096E-06	6.55124E-06	1.92466E-06	heme synthase
K22229	1.96938E-06	3.31728E-06	1.52667E-05	3.49236E-05	2-ketogluconate reductase [EC:1.1.1.215]
K22230	1.37044E-06	2.47112E-06	7.95648E-06	1.62213E-05	scyllo-inositol 2-dehydrogenase (NADP+) [EC:1.1.1.-]
K22247	6.21208E-11	1.54508E-10	4.47358E-10	1.84016E-09	polysialic-acid O-acetyltransferase [EC:2.3.1.136]
K22249	1.1086E-07	6.99898E-08	7.51728E-07	9.41834E-06	poly(3-hydroxyoctanoate) depolymerase [EC:3.1.1.76]
K22250	4.24624E-08	1.04551E-08	1.4649E-06	2.57799E-07	poly(3-hydroxyoctanoate) depolymerase [EC:3.1.1.76]
K22251	1.21457E-06	3.02321E-06	1.37332E-05	3.46142E-05	glycerol dehydrogenase [EC:1.1.1.6]
K22252	2.68423E-07	5.54911E-07	1.04675E-06	4.7215E-06	GDP-6-deoxy-D-talose 4-dehydrogenase [EC:1.1.1.135]
K22253	0	6.88612E-08	1.54759E-07	1.33655E-08	glucan 1,4-alpha-maltotetraohydrolase [EC:3.2.1.60]
K22268	0	7.73089E-08	3.68066E-07	0	xylan 1,4-beta-xylosidase [EC:3.2.1.37]
K22270	1.66048E-06	5.00542E-06	1.93284E-05	4.32736E-05	3-hydroxybenzoate 6-monooxygenase [EC:1.14.13.24]
K22278	0.000223963	0.000225893	0.000230218	0.000261387	peptidoglycan-N-acetylglucosamine deacetylase [EC:3.5.1.104]
K22292	0.000218918	0.000217807	0.000214081	0.000208208	N-acetyl-D-muramate 6-phosphate phosphatase [EC:3.1.3.105]
K22293	1.78836E-05	2.01668E-05	8.10175E-05	0.000245759	GntR family transcriptional regulator, rspAB operon transcriptional repressor
K22294	0.00042882	0.000425575	0.00038163	0.000247488	TetR/AcrR family transcriptional regulator, upper aerobic nicotinate degradation pathway regulator
K22295	0	1.06439E-08	1.04657E-08	3.42745E-08	TetR/AcrR family transcriptional regulator, repressor for neighboring sulfatase
K22296	1.43895E-06	3.27038E-06	1.62441E-05	3.4853E-05	MarR family transcriptional regulator, lower aerobic nicotinate degradation pathway regulator
K22297	1.00394E-06	3.18089E-06	1.3613E-06	5.62498E-06	Fur family transcriptional regulator, stress-responsive regulator
K22298	1.35611E-07	1.27855E-06	3.47165E-06	5.69424E-06	ArsR family transcriptional regulator, zinc-responsive transcriptional repressor
K22299	3.2196E-07	1.32452E-07	6.08803E-07	3.91717E-09	HTH-type transcriptional regulator, competence development regulator
K22300	7.73198E-09	1.37244E-08	3.09525E-08	2.01776E-07	HTH-type transcriptional regulator, cell division transcriptional repressor
K22301	5.54378E-08	0	8.48566E-08	0	HTH-type transcriptional regulator, osmoprotectant uptake regulator
K22302	2.13101E-08	6.42747E-08	6.82107E-08	2.12971E-06	transcriptional repressor of cell division inhibition gene dicB
K22303	4.42142E-06	1.06949E-05	4.93896E-05	0.000127898	alpha-ketoglutarate-dependent sulfate ester dioxygenase [EC:1.14.11.-]
K22304	7.04002E-09	2.49934E-08	5.06981E-08	2.73245E-07	cell division inhibition protein DicB
K22305	1.45555E-06	2.13464E-06	3.3016E-06	5.16485E-05	phosphoserine phosphatase [EC:3.1.3.3]
K22306	1.70296E-06	1.57821E-06	2.01098E-05	6.43398E-06	glucosyl-3-phosphoglycerate phosphatase [EC:3.1.3.85]
K22307	1.39291E-07	3.1736E-09	6.26006E-08	4.24347E-08	1,3-alpha-isomaltosidase [EC:3.2.1.204]
K22308	0	0	0	3.48277E-08	isomaltose glucohydrolase [EC:3.2.1.205]
K22310	0.000215108	0.000211959	0.000186802	0.00011343	L-ornithine Nalpha-acyltransferase [EC:2.3.2.30]
K22311	1.08547E-06	7.39162E-07	3.36487E-06	1.98222E-06	phosphatidylinositol dimannoside acyltransferase [EC:2.3.1.265]
K22314	0	0	3.48857E-08	5.70522E-08	glucosinolate gamma-glutamyl hydrolase [EC:3.4.19.16]
K22316	1.06735E-06	3.65127E-06	3.93827E-06	2.19018E-06	ribonuclease H / adenosylcobalamin/alpha-ribazole phosphatase [EC:3.1.26.4 3.1.3.73]
K22317	1.9198E-06	4.27142E-06	2.37202E-06	5.47872E-05	acyl-CoA:acyl-CoA alkyltransferase [EC:2.3.3.20]
K22318	1.02833E-06	1.35494E-06	2.08202E-06	5.42662E-05	3-alkyl-4-acyloxetan-2-one decarboxylase
K22319	4.77527E-06	1.11467E-05	3.42749E-05	0.000137082	olefin beta-lactone synthetase [EC:6.1.3.1]
K22320	2.69643E-06	4.71368E-06	4.39743E-06	0.000105195	2-alkyl-3-oxoalkanoate reductase [EC:1.1.1.412]
K22322	3.00879E-06	4.82943E-06	2.17291E-05	4.09187E-05	A-factor type gamma-butyrolactone 1-reductase (1S-forming) [EC:1.1.1.413]
K22325	1.88948E-06	4.25499E-06	2.01225E-05	4.86801E-05	chloroacetanilide N-alkylformylase [EC:1.14.15.23]
K22330	2.4049E-08	1.24673E-08	2.59491E-07	6.03958E-07	tyrosine decarboxylase [EC:4.1.1.25]
K22332	2.59287E-11	6.44902E-11	1.86723E-10	1.20736E-06	beta-lactamase class D OXA-548 [EC:3.5.2.6]
K22333	0	0	2.6561E-08	4.82131E-10	beta-lactamase class D OXA-493 [EC:3.5.2.6]
K22335	0.000214969	0.000214753	0.00019993	0.000147812	beta-lactamase class D OXA-114 [EC:3.5.2.6]
K22336	9.85028E-09	2.79899E-07	1.00388E-06	3.47413E-06	bacterioferritin B [EC:1.16.3.1]
K22338	0	0	1.78121E-07	1.27337E-08	formate dehydrogenase (NAD+, ferredoxin) subunit A [EC:1.17.1.11]
K22339	0	4.03206E-08	9.26836E-08	1.36766E-07	formate dehydrogenase (NAD+, ferredoxin) subunit B [EC:1.17.1.11]
K22340	9.74027E-08	3.86545E-08	5.80788E-07	1.24032E-07	formate dehydrogenase (NAD+, ferredoxin) subunit C [EC:1.17.1.11]
K22341	0	3.74292E-09	4.2443E-07	2.54673E-08	formate dehydrogenase (NAD+, ferredoxin) subunit [EC:1.17.1.11]
K22342	4.51627E-07	2.21586E-07	2.92113E-07	1.71859E-07	dimethylamine monooxygenase subunit A [EC:1.14.13.238]
K22343	0.000215688	0.000215525	0.000203649	0.000151586	dimethylamine monooxygenase subunit B [EC:1.14.13.238]
K22344	4.39639E-07	1.81193E-07	2.6809E-07	1.41001E-07	dimethylamine monooxygenase subunit C [EC:1.14.13.238]
K22345	0.000215066	0.000212581	0.00018868	0.000126339	glucosaminate ammonia-lyase [EC:4.3.1.9]
K22347	9.80607E-07	6.2272E-07	5.46507E-07	3.58181E-06	4,4-dithiodibutanoate disulfide reductase [EC:1.8.1.20]
K22348	0.000220729	0.000219109	0.000217649	0.000228081	manganese oxidase [EC:1.16.3.3]
K22349	8.93218E-08	7.52879E-08	3.0788E-07	5.9282E-08	manganese oxidase [EC:1.16.3.3]
K22350	2.94158E-08	5.24316E-08	1.99622E-07	4.9107E-08	manganese oxidase [EC:1.16.3.3]
K22351	5.01007E-09	7.24899E-08	0	0	beta-lactamase class D OXA-209 [EC:3.5.2.6]
K22352	3.40633E-08	1.58053E-08	2.68152E-08	6.05995E-08	beta-lactamase class D OXA-29 [EC:3.5.2.6]
K22353	0	3.19318E-08	6.50889E-10	2.21801E-07	alkene monooxygenase alpha subunit [EC:1.14.13.69]
K22354	0	1.06439E-08	0	3.48277E-08	alkene monooxygenase beta subunit [EC:1.14.13.69]
K22355	0	2.12879E-08	0	3.48277E-08	alkene monooxygenase coupling protein
K22356	0	2.12879E-08	0	3.48277E-08	alkene monooxygenase reductase
K22363	0	1.06439E-08	0	6.96554E-08	2-hydroxypropyl-CoM lyase [EC:4.4.1.23]
K22364	0	3.19318E-08	0	3.48277E-08	2-oxopropyl-CoM reductase (carboxylating) [EC:1.8.1.5]
K22369	1.71257E-09	0	2.38659E-08	0	epoxide hydrolase 4 [EC:3.3.-.-]
K22373	1.06615E-06	1.91795E-07	2.84051E-06	1.86327E-07	lactate racemase [EC:5.1.2.1]
K22389	3.65706E-08	6.5978E-09	3.48857E-09	4.68835E-09	phospholipase A1 [EC:3.1.1.32]
K22391	1.1744E-06	4.31467E-07	3.38665E-06	4.14066E-08	GTP cyclohydrolase I [EC:3.5.4.16]
K22393	1.23418E-07	0	2.91639E-07	2.68243E-08	flavin reductase (NADH) subunit 1 [EC:1.5.1.36]
K22394	2.18558E-07	9.24084E-08	6.39782E-07	3.09634E-06	flavin reductase (NADH) subunit 2 [EC:1.5.1.36]
K22396	3.457E-08	3.89494E-08	2.28656E-07	1.39116E-07	xylonate dehydratase [EC:4.2.1.82]
K22397	4.21366E-09	3.64305E-08	4.80559E-07	1.88374E-07	2-dehydro-3-deoxy-D-pentonate aldolase [EC:4.1.2.28]
K22405	2.67359E-07	7.83045E-07	8.53376E-06	4.98961E-07	NADH oxidase (H2O-forming) [EC:1.6.3.4]
K22408	0	0	2.58094E-09	0	diacetylchitobiose deacetylase [EC:3.5.1.-]
K22409	6.49479E-06	2.36293E-09	1.97103E-05	1.80551E-06	N-acetylmuramoyl-L-alanine amidase [EC:3.5.1.28]
K22424	0	2.59535E-09	3.35565E-07	3.29608E-08	glutamine kinase [EC:2.7.3.13]
K22430	6.00765E-06	1.06969E-05	4.05832E-05	0.000126017	caffeyl-CoA reductase-Etf complex subunit CarC [EC:1.3.1.108]
K22431	2.3943E-06	1.15341E-06	4.95259E-06	7.26686E-08	caffeyl-CoA reductase-Etf complex subunit CarD [EC:1.3.1.108]
K22432	1.95694E-06	1.03366E-06	1.30686E-06	1.62055E-07	caffeyl-CoA reductase-Etf complex subunit CarE [EC:1.3.1.108]
K22441	2.25467E-06	8.13116E-07	9.5707E-06	5.76187E-05	diamine N-acetyltransferase [EC:2.3.1.57]
K22443	1.27386E-07	3.62373E-07	5.78923E-07	7.44798E-07	carnitine monooxygenase subunit [EC:1.14.13.239]
K22444	3.87018E-07	8.38818E-07	3.43108E-06	8.17552E-06	carnitine monooxygenase subunit [EC:1.14.13.239]
K22445	0.000217967	0.000217612	0.000210654	0.000236648	4,4-diapolycopenoate synthase [EC:1.2.99.10]
K22446	6.01589E-08	2.81046E-07	3.6441E-08	2.48118E-08	tRNA (cytosine49-C5)-methyltransferase [EC:2.1.1.-]

K22447	2.93706E-07	1.31002E-09	6.09774E-08	1.56369E-08	archaeal chaperonin
K22451	0	1.96503E-08	2.09314E-08	5.35811E-09	4-alpha-glucanotransferase [EC:2.4.1.25]
K22452	0.00021805	0.000217655	0.000212365	0.000219245	protein-glutamine gamma-glutamyltransferase [EC:2.3.2.13]
K22457	1.00376E-06	1.33072E-06	2.28079E-06	5.78356E-06	asparagine---oxo-acid transaminase [EC:2.6.1.14]
K22462	2.56886E-09	2.18337E-09	5.07264E-07	3.46615E-08	(4-alkanoyl-5-oxo-2,5-dihydrofuran-3-yl)methyl phosphate reductase [EC:1.3.1.113]
K22463	2.40723E-11	2.91737E-09	5.84388E-07	1.79162E-06	A-factor biosynthesis enzyme
K22465	1.44434E-10	5.68806E-08	0	1.70245E-08	5-hydroxybenzimidazole synthase [EC:4.1.99.23]
K22468	1.3354E-05	1.93778E-05	5.24087E-05	0.000140748	polyphosphate kinase [EC:2.7.4.1]
K22473	1.02237E-06	9.56154E-09	3.79128E-07	8.82837E-08	alcohol dehydrogenase (quinone), dehydrogenase subunit [EC:1.1.5.5]
K22474	0.000216548	0.000217195	0.000202423	0.00020754	alcohol dehydrogenase (quinone), cytochrome c subunit [EC:1.1.5.5]
K22476	1.0265E-07	7.12525E-07	1.32407E-06	1.79552E-06	N-acetylglutamate synthase [EC:2.3.1.1]
K22477	1.12535E-07	7.90177E-08	1.57864E-07	8.36974E-08	N-acetylglutamate synthase [EC:2.3.1.1]
K22478	1.06114E-06	1.2356E-06	1.68801E-06	5.14341E-05	bifunctional N-acetylglutamate synthase/kinase [EC:2.3.1.1 2.7.2.8]
K22479	0.000216799	0.000215673	0.000204779	0.000204646	N-acetyltransferase
K22480	0	1.85547E-09	6.54027E-09	3.42565E-07	heterodisulfide reductase subunit A1 [EC:1.8.7.3]
K22481	0	0	4.82911E-07	0	heterodisulfide reductase subunit B1 [EC:1.8.7.3]
K22486	3.49349E-09	2.44172E-08	3.29733E-08	1.74927E-07	transcriptional regulator HilA, main transcriptional regulator of SPI1
K22487	2.47661E-09	1.67008E-08	7.9089E-08	1.32735E-07	type III secretion system protein
K22488	2.97806E-09	2.16966E-08	8.68283E-08	2.0225E-07	type III secretion system protein
K22489	0.000213807	0.000211848	0.000186147	0.000113801	MarR family transcriptional regulator, temperature-dependent positive regulator of motility
K22490	0	0	3.13971E-08	0	CRP/FNR family transcriptional regulator, LitR-dependent transcriptional activator
K22491	3.812E-06	1.02009E-05	3.22676E-05	7.81577E-05	MerR family transcriptional regulator, light-induced transcriptional regulator
K22492	0	0	1.81612E-08	0	beta-carotene 3-hydroxylase [EC:1.14.15.24]
K22501	3.99859E-09	1.74287E-08	2.87955E-08	1.83151E-07	cytochrome bd-II ubiquinol oxidase subunit AppX [EC:1.10.3.14]
K22502	0	0	3.39781E-08	0	lycopene beta-cyclase [EC:5.5.1.19]
K22503	0	1.50352E-08	3.69959E-09	2.13946E-09	aspartyl-tRNA synthetase [EC:6.1.1.12]
K22504	3.43707E-09	1.29236E-08	7.72228E-08	3.22837E-08	type III secretion system outer membrane ring protein
K22505	2.28863E-09	1.67068E-08	8.18634E-08	1.27167E-07	type III secretion system inner membrane ring protein
K22506	0.000214041	0.000213065	0.000189412	0.000120301	type III secretion system ATPase [EC:3.6.3.14]
K22507	1.29153E-06	3.61168E-06	1.61314E-05	3.49814E-05	type III secretion system export apparatus protein
K22508	2.47564E-09	1.71719E-08	1.15719E-07	1.38982E-07	type III secretion system export apparatus protein
K22509	2.67659E-09	1.76717E-08	8.46573E-08	1.38659E-07	type III secretion system export apparatus protein
K22510	1.35527E-08	2.77175E-08	1.02657E-07	2.75924E-07	type III secretion system export apparatus switch protein
K22511	1.0876E-09	1.04445E-08	7.32143E-08	3.01453E-08	type III secretion system protein
K22512	3.72165E-10	8.0044E-09	6.3934E-08	2.16919E-09	type III secretion system chaperone
K22513	7.00004E-10	1.92821E-09	8.72808E-09	2.07356E-08	type III secretion system protein
K22514	9.00951E-10	2.24086E-09	6.48812E-09	2.66881E-08	type III secretion system protein
K22515	1.11032E-06	7.83349E-07	1.21828E-06	5.05227E-07	formate dehydrogenase beta subunit [EC:1.17.1.9]
K22516	1.35565E-06	8.48363E-07	2.05556E-06	6.31931E-06	formate dehydrogenase (coenzyme F420) alpha subunit [EC:1.17.98.3 1.8.98.6]
K22523	0	0	2.22133E-08	0	sulfotransferase 6B1 [EC:2.8.2.-]
K22539	6.74681E-08	1.10408E-08	2.57115E-07	1.78604E-09	pectate lyase [EC:4.2.2.2]
K22548	1.59327E-07	1.68746E-07	4.78206E-07	1.42821E-07	trans-L-3-hydroxyproline dehydratase [EC:4.2.1.77]
K22549	6.6929E-07	7.30808E-07	1.62857E-06	4.27932E-07	D-hydroxyproline dehydrogenase subunit alpha [EC:1.5.99.-]
K22550	5.73519E-07	4.65726E-07	1.39738E-06	3.2841E-07	D-hydroxyproline dehydrogenase subunit gamma
K22551	3.66724E-06	4.36343E-06	1.94217E-05	3.95243E-05	N-methyl-L-proline demethylase [EC:1.5.3.-]
K22552	0.000220907	0.000220614	0.000203068	0.000218374	multicopper oxidase [EC:1.16.3.1]
K22553	9.41544E-08	5.80213E-07	1.9464E-06	1.46534E-06	4-methoxybenzoate monooxygenase (O-demethylating) [EC:1.14.99.15]
K22579	0.000218357	0.000218234	0.000214473	0.000218376	rifampicin phosphotransferase [EC:2.7.9.6]
K22580	9.36655E-07	0	2.74951E-06	3.03626E-08	teichoic acid D-alanine hydrolase [EC:3.1.1.103]
K22589	0.000220239	0.000221691	0.000232858	0.00026916	threo-3-hydroxy-L-aspartate ammonia-lyase [EC:4.3.1.16]
K22597	5.40865E-08	2.15984E-08	1.09746E-07	6.64703E-07	glucosylglycerate phosphorylase [EC:2.4.1.352]
K22601	1.091E-06	1.47425E-07	3.834E-07	2.09009E-06	oxamate carbamoyltransferase [EC:2.1.3.5]
K22602	6.67046E-07	1.32382E-07	4.6867E-07	4.07058E-06	oxamate amidohydrolase [EC:3.5.1.126]
K22605	9.73328E-07	8.32656E-08	3.00658E-08	2.26965E-08	3alpha-hydroxycholanate dehydrogenase (NADP+) [EC:1.1.1.392]
K22606	0	0	2.12142E-09	0	3beta-hydroxycholanate 3-dehydrogenase (NAD+) [EC:1.1.1.391]
K22614	0.000427531	0.000423549	0.000372296	0.000226405	NLR family CARD domain-containing protein 3
K22616	2.08916E-07	4.20794E-07	4.4159E-07	1.94226E-06	ornithine lipid ester-linked acyl 2-hydroxylase [EC:1.14.11.58]
K22617	2.72905E-06	1.07957E-06	3.37995E-06	2.89582E-06	lyso-ornithine lipid:acyl-ACP O-acyltransferase
K22618	0.000214139	0.000211956	0.000186446	0.000114925	ornithine lipid hydroxylase
K22622	4.01519E-07	2.57255E-07	1.35716E-06	5.23463E-07	S-disulfanyl-L-cysteine oxidoreductase SoxD [EC:1.8.2.6]
K22650	9.82896E-08	3.02346E-07	7.5482E-08	7.70333E-07	luxR family transcriptional regulator, regulator of transport and utilization of aryl beta-glucosides
K22684	0	0	6.97714E-09	0	metacaspase-1 [EC:3.4.22.-]
K22686	0	0	2.09314E-08	0	pro-apoptotic serine protease NMA111 [EC:3.4.21.-]
K22690	9.16819E-09	8.88608E-09	0	0	exo-cleaving rubber dioxygenase [EC:1.13.11.85]
K22693	7.2217E-11	9.23501E-11	1.63332E-07	0	tryptophan 5-halogenase [EC:1.14.19.58]
K22694	2.67826E-07	1.36074E-07	8.00497E-07	5.8534E-07	tryptophan 6-halogenase [EC:1.14.19.59]
K22699	1.09404E-06	1.99804E-07	2.52122E-06	1.83984E-07	sulfide-dependent adenosine diphosphate thiazole synthase [EC:2.4.2.59]
K22704	4.48335E-09	0	2.67457E-08	1.78604E-09	hopanoid C-3 methylase [EC:2.1.1.-]
K22705	1.34494E-06	2.09556E-08	1.26521E-07	2.22578E-09	hopanoid C-2 methylase [EC:2.1.1.-]
K22708	1.89118E-07	6.64661E-08	4.54182E-08	1.52278E-06	poly(ribitol-phosphate) beta-N-acetylglucosaminyltransferase [EC:2.4.1.355]
K22709	7.70407E-09	0	0	0	poly(ribitol-phosphate) beta-glucosyltransferase [EC:2.4.1.53]
K22710	3.73159E-08	0	5.02441E-09	0	poly(ribitol-phosphate) alpha-N-acetylglucosaminyltransferase [EC:2.4.1.70]
K22711	2.58173E-07	1.56387E-07	4.91709E-07	1.86855E-05	LysR family transcriptional regulator, pyoluteorin biosynthesis regulator
K22712	0	0	6.16974E-08	0	1H-pyrrole-2-carbonyl-[peptidyl-carrier protein] chlorinase [EC:1.14.19.56]
K22714	0.000427678	0.000423942	0.000375727	0.000227119	polyketide synthase
K22718	3.46796E-10	8.62557E-10	1.31521E-08	3.60111E-08	divisome-associated membrane protein
K22719	2.1618E-06	2.41599E-06	4.76507E-06	7.00247E-05	murein hydrolase activator
K22720	2.8417E-08	1.96788E-08	4.6127E-08	6.78719E-07	surface composition regulator
K22721	1.20362E-10	7.65155E-09	9.26836E-08	1.90174E-09	inositol phosphorylceramide mannosyltransferase catalytic subunit [EC:2.4.1.-]
K22723	0	0	0	2.03996E-08	inositol phosphorylceramide synthase catalytic subunit [EC:2.7.1.-]
K22736	7.21796E-06	8.65849E-06	1.54005E-05	6.94268E-05	vacuolar iron transporter family protein
K22737	1.02076E-06	1.88077E-07	2.40427E-07	5.60643E-08	erythrin-vacuolar iron transport family protein
K22745	4.82798E-09	0	9.32766E-08	6.53358E-08	apoptosis-inducing factor 2
K22747	1.4172E-06	5.99204E-08	8.55233E-08	4.27317E-08	apoptosis-inducing factor 3
K22757	5.76343E-07	1.48003E-06	2.75347E-06	1.8616E-06	glutaminyl-peptide cyclotransferase [EC:2.3.2.5]
K22769	1.818E-06	4.42037E-06	3.77415E-06	6.20958E-05	NADPH-dependent stearoyl-CoA 9-desaturase [EC:1.14.19.-]
K22770	1.75084E-06	4.14022E-06	3.55363E-06	4.96163E-05	stearoyl-CoA 9-desaturase NADPH oxidoreductase

K22795	3.42515E-09	2.58527E-07	8.74958E-08	8.77497E-06	trehalose acyltransferase [EC:2.3.1.-]
K22796	0	1.98449E-07	1.54857E-08	4.37052E-08	diacyltrehalose/SL1278 acyltransferase [EC:2.3.1.-]
K22797	0	1.59578E-07	1.40844E-06	1.7338E-06	diglucosylglycerate octanoyltransferase [EC:2.3.1.-]
K22818	0.000223151	0.000230476	0.00026025	0.000291361	3-oxocholest-4-en-26-oyl-CoA dehydrogenase alpha subunit [EC:1.3.99.-]
K22819	1.21637E-06	3.84093E-06	1.53801E-05	4.30839E-05	3-oxocholest-4-en-26-oyl-CoA dehydrogenase beta subunit [EC:1.3.99.-]
K22820	3.58984E-06	8.6222E-06	3.02791E-05	8.25303E-05	3-oxochol-4-en-24-oyl-CoA dehydrogenase [EC:1.3.99.-]
K22821	0	1.34585E-07	2.23088E-07	6.94086E-06	3-oxo-4-pregnene-20-carboxyl-CoA dehydrogenase alpha subunit [EC:1.3.99.-]
K22822	0.000217073	0.000217059	0.000211825	0.000171522	3-oxo-4-pregnene-20-carboxyl-CoA dehydrogenase beta subunit [EC:1.3.99.-]
K22831	1.14262E-06	3.97163E-08	7.75517E-07	1.74323E-06	1-acylglycerol-3-phosphate O-acyltransferase [EC:2.3.1.51]
