# Supplementary material for: Multiscale Plant Defense Strategies against Ciprofloxacin Stress: From Chloroplast-Centered Adaptation to Microbiome Coordination
Source: Research (Wash D C). 2026 Jan 15;9:1082. doi: 10.34133/research.1082 (PMC12804602; doi:10.34133/research.1082)
Supplement: Supplementary 1 — Description of Supplemental Methods Figs. S1 to S4 Tables S1 to S9 [file research.1082.f1.zip › Supplemental Information Table S9.pdf]

Table S9. Pathway

pathway	CK	5 ppm	10 ppm	20 ppm	level1	level2	level3
ko00010	0.007388817	0.007392587	0.007554886	0.007589494	Glycolysis / Gluconeogenesis	Carbohydrate metabolism	Metabolism
ko00020	0.004989605	0.005001202	0.005019673	0.005091088	Citrate cycle (TCA cycle)	Carbohydrate metabolism	Metabolism
ko00030	0.003875439	0.003863184	0.003898128	0.003919496	Pentose phosphate pathway	Carbohydrate metabolism	Metabolism
ko00040	0.002360028	0.002351795	0.0023607	0.002235956	Pentose and glucuronate interconversions	Carbohydrate metabolism	Metabolism
ko00051	0.002907399	0.002918918	0.003198797	0.003563232	Fructose and mannose metabolism	Carbohydrate metabolism	Metabolism
ko00052	0.002010757	0.002011859	0.002082156	0.002055739	Galactose metabolism	Carbohydrate metabolism	Metabolism
ko00053	0.003188047	0.003183758	0.003100811	0.002791861	Ascorbate and aldarate metabolism	Carbohydrate metabolism	Metabolism
ko00061	0.006845931	0.00685865	0.006959196	0.007529272	Fatty acid biosynthesis	Lipid metabolism	Metabolism
ko00062	8.61277E-08	1.12843E-07	1.3801E-07	5.784E-08	Fatty acid elongation	Lipid metabolism	Metabolism
ko00071	0.007712837	0.007786211	0.007994249	0.008483399	Fatty acid degradation	Lipid metabolism	Metabolism
ko00072	0.002103257	0.002109715	0.002101105	0.002221951	Synthesis and degradation of ketone bodies	Lipid metabolism	Metabolism
ko00073	0	2.09517E-09	7.70609E-08	3.55439E-08	Cutin, suberine and wax biosynthesis	Lipid metabolism	Metabolism
ko00100	1.57328E-07	4.02014E-07	8.02266E-07	1.03099E-06	Steroid biosynthesis	Lipid metabolism	Metabolism
ko00120	0.000256532	0.000266318	0.000348148	0.000473642	Primary bile acid biosynthesis	Lipid metabolism	Metabolism
ko00121	6.24727E-06	7.81804E-06	3.99569E-05	0.000134519	Secondary bile acid biosynthesis	Lipid metabolism	Metabolism
ko00130	0.002950628	0.002941918	0.002805795	0.002742331	Ubiquinone and other terpenoid-quinone biosynthesis	Metabolism of cofactors and vitamins	Metabolism
ko00140	0.000616555	0.000622259	0.000629419	0.000608632	Steroid hormone biosynthesis	Lipid metabolism	Metabolism
ko00190	0.009262587	0.009216235	0.008949778	0.008444773	Oxidative phosphorylation	Energy metabolism	Metabolism
ko00195	0.001246637	0.001245314	0.001239965	0.001226366	Photosynthesis	Energy metabolism	Metabolism
ko00196	0	0	7.82655E-09	0	Photosynthesis - antenna proteins	Energy metabolism	Metabolism
ko00220	0.004215374	0.004217582	0.004157393	0.003778282	Arginine biosynthesis	Amino acid metabolism	Metabolism
ko00230	0.010274053	0.010274541	0.010416471	0.010594309	Purine metabolism	Nucleotide metabolism	Metabolism
ko00232	6.10402E-07	1.19154E-07	4.34101E-07	2.08478E-06	Caffeine metabolism	Biosynthesis of other secondary metabolites	Metabolism
ko00240	0.005802753	0.005805075	0.006012877	0.006354364	Pyrimidine metabolism	Nucleotide metabolism	Metabolism
ko00250	0.005720427	0.00574798	0.00577163	0.00587497	Alanine, aspartate and glutamate metabolism	Amino acid metabolism	Metabolism
ko00253	0.000123014	0.000125454	0.000128069	0.000121145	Tetracycline biosynthesis	Metabolism of terpenoids and polyketides	Metabolism
ko00260	0.008546656	0.008528568	0.008423649	0.007974473	Glycine, serine and threonine metabolism	Amino acid metabolism	Metabolism
ko00261	0.001366897	0.001375195	0.001367871	0.001402224	Monobactam biosynthesis	Biosynthesis of other secondary metabolites	Metabolism
ko00270	0.007098684	0.007100051	0.006985683	0.007154988	Cysteine and methionine metabolism	Amino acid metabolism	Metabolism
ko00280	0.010161069	0.010248643	0.010363134	0.010585986	Valine, leucine and isoleucine degradation	Amino acid metabolism	Metabolism
ko00281	0.002226688	0.002244791	0.002265382	0.002348596	Geraniol degradation	Metabolism of terpenoids and polyketides	Metabolism
ko00290	0.002487458	0.002482192	0.002488145	0.002467226	Valine, leucine and isoleucine biosynthesis	Amino acid metabolism	Metabolism
ko00300	0.003599624	0.003604985	0.003604194	0.00340973	Lysine biosynthesis	Amino acid metabolism	Metabolism
ko00310	0.005913918	0.005941828	0.005851658	0.005762366	Lysine degradation	Amino acid metabolism	Metabolism
ko00311	0.000246569	0.000244745	0.000246425	0.000242303	Penicillin and cephalosporin biosynthesis	Biosynthesis of other secondary metabolites	Metabolism
ko00330	0.007848158	0.007837882	0.00749068	0.006833553	Arginine and proline metabolism	Amino acid metabolism	Metabolism
ko00331	0	1.21574E-07	1.56488E-07	5.26554E-08	Clavulanic acid biosynthesis	Biosynthesis of other secondary metabolites	Metabolism
ko00332	0.000250635	0.000251146	0.000256333	0.000264399	Carbapenem biosynthesis	Biosynthesis of other secondary metabolites	Metabolism
ko00333	0.00372483	0.003720283	0.003738705	0.00396401	Prodigiosin biosynthesis	Biosynthesis of other secondary metabolites	Metabolism
ko00340	0.004324174	0.004328175	0.004278485	0.004149717	Histidine metabolism	Amino acid metabolism	Metabolism
ko00350	0.006028753	0.006040697	0.005917423	0.005618092	Tyrosine metabolism	Amino acid metabolism	Metabolism
ko00360	0.007378475	0.007403127	0.007238343	0.006668313	Phenylalanine metabolism	Amino acid metabolism	Metabolism
ko00361	0.001105984	0.001102776	0.00107141	0.001005067	Chlorocyclohexane and chlorobenzene degradation	Xenobiotics biodegradation and metabolism	Metabolism
ko00362	0.007267351	0.007324888	0.007338862	0.007285699	Benzoate degradation	Xenobiotics biodegradation and metabolism	Metabolism
ko00363	7.47677E-07	6.09404E-07	2.41129E-06	8.10446E-06	Bisphenol degradation	Xenobiotics biodegradation and metabolism	Metabolism
ko00364	0.000252122	0.000255541	0.000278783	0.000360875	Fluorobenzoate degradation	Xenobiotics biodegradation and metabolism	Metabolism
ko00365	6.96755E-06	4.17479E-06	1.76732E-05	5.63857E-05	Furfural degradation	Xenobiotics biodegradation and metabolism	Metabolism
ko00380	0.005431986	0.005456079	0.00541165	0.005412432	Tryptophan metabolism	Amino acid metabolism	Metabolism
ko00400	0.004829743	0.004821807	0.004731493	0.004517846	Phenylalanine, tyrosine and tryptophan biosynthesis	Amino acid metabolism	Metabolism
ko00401	0.001598507	0.001589369	0.001512893	0.001302093	Novobiocin biosynthesis	Biosynthesis of other secondary metabolites	Metabolism
ko00404	7.04627E-07	6.42302E-07	3.03641E-06	8.05455E-06	Staurosporine biosynthesis	Biosynthesis of other secondary metabolites	Metabolism
ko00405	0.000501433	0.000501827	0.000508861	0.000596151	Phenazine biosynthesis	Biosynthesis of other secondary metabolites	Metabolism
ko00410	0.00602286	0.0060498	0.005942404	0.005736691	beta-Alanine metabolism	Metabolism of other amino acids	Metabolism
ko00430	0.001003049	0.001007489	0.001050794	0.001141169	Taurine and hypotaurine metabolism	Metabolism of other amino acids	Metabolism
ko00440	0.00025637	0.000252054	0.000265214	0.000293867	Phosphonate and phosphinate metabolism	Metabolism of other amino acids	Metabolism
ko00450	0.002385478	0.002409806	0.002446425	0.002600439	Selenocompound metabolism	Metabolism of other amino acids	Metabolism
ko00460	0.000636229	0.000642187	0.000727497	0.000913805	Cyanoamino acid metabolism	Metabolism of other amino acids	Metabolism
ko00471	0.00074739	0.000749637	0.000748969	0.000726863	D-Glutamine and D-glutamate metabolism	Metabolism of other amino acids	Metabolism
ko00472	0.000124526	0.000124465	0.000139524	0.000108709	D-Arginine and D-ornithine metabolism	Metabolism of other amino acids	Metabolism
ko00473	0.000740321	0.000735887	0.000725283	0.000653154	D-Alanine metabolism	Metabolism of other amino acids	Metabolism
ko00480	0.004427825	0.004403516	0.004240955	0.004129443	Glutathione metabolism	Metabolism of other amino acids	Metabolism
ko00500	0.004960053	0.004936365	0.004929128	0.004456228	Starch and sucrose metabolism	Carbohydrate metabolism	Metabolism
ko00510	5.31082E-06	5.98989E-06	1.32523E-05	4.98613E-05	N-Glycan biosynthesis	Glycan biosynthesis and metabolism	Metabolism
ko00511	0.000140395	0.000150633	0.000204634	0.000240464	Other glycan degradation	Glycan biosynthesis and metabolism	Metabolism
ko00513	3.48231E-06	6.69554E-06	1.84432E-05	4.89279E-05	Various types of N-glycan biosynthesis	Glycan biosynthesis and metabolism	Metabolism
ko00514	2.20896E-06	5.3334E-06	2.01748E-05	3.81075E-05	Other types of O-glycan biosynthesis	Glycan biosynthesis and metabolism	Metabolism
ko00515	8.61855E-07	2.32374E-06	5.67848E-06	2.33067E-06	Mannose type O-glycan biosynthesis	Glycan biosynthesis and metabolism	Metabolism
ko00520	0.005410852	0.005402691	0.005566495	0.005566241	Amino sugar and nucleotide sugar metabolism	Carbohydrate metabolism	Metabolism
ko00521	0.001371812	0.001370971	0.001344381	0.001387064	Streptomycin biosynthesis	Biosynthesis of other secondary metabolites	Metabolism
ko00522	3.12765E-09	5.20666E-08	5.46174E-06	2.75295E-07	Biosynthesis of 12-, 14- and 16-membered macrolides	Metabolism of terpenoids and polyketides	Metabolism
ko00523	0.000625956	0.000631599	0.000634123	0.000702464	Polyketide sugar unit biosynthesis	Metabolism of terpenoids and polyketides	Metabolism
ko00524	0.000248349	0.000245718	0.000240662	0.00024531	Neomycin, kanamycin and gentamicin biosynthesis	Biosynthesis of other secondary metabolites	Metabolism
ko00525	0.000252119	0.000255377	0.000259056	0.000308266	Acarbose and validamycin biosynthesis	Biosynthesis of other secondary metabolites	Metabolism
ko00531	0.000131098	0.000137603	0.000172717	0.000217567	Glycosaminoglycan degradation	Glycan biosynthesis and metabolism	Metabolism
ko00532	0	2.48639E-08	9.72912E-08	1.94073E-06	Glycosaminoglycan biosynthesis - chondroitin sulfate	Glycan biosynthesis and metabolism	Metabolism
ko00534	0	2.48639E-08	9.58173E-08	1.93889E-06	Glycosaminoglycan biosynthesis - heparan sulfate / hc	Glycan biosynthesis and metabolism	Metabolism
ko00540	0.003422952	0.003399753	0.003175803	0.002832765	Lipopolysaccharide biosynthesis	Glycan biosynthesis and metabolism	Metabolism
ko00550	0.002541881	0.002536515	0.002679187	0.00297114	Peptidoglycan biosynthesis	Glycan biosynthesis and metabolism	Metabolism
ko00561	0.002849378	0.002843348	0.002884008	0.002776001	Glycerolipid metabolism	Lipid metabolism	Metabolism
ko00562	0.002683615	0.002671076	0.002495514	0.002092594	Inositol phosphate metabolism	Carbohydrate metabolism	Metabolism
ko00563	0	0	5.39869E-08	0	Glycosylphosphatidylinositol (GPI)-anchor biosynthes	Glycan biosynthesis and metabolism	Metabolism
ko00564	0.003236979	0.003225802	0.003258211	0.003420572	Glycerophospholipid metabolism	Lipid metabolism	Metabolism
ko00565	0.000487337	0.000482117	0.000449835	0.000405619	Ether lipid metabolism	Lipid metabolism	Metabolism
ko00571	6.63703E-06	1.27146E-05	4.17254E-05	3.18711E-05	Lipoarabinomannan (LAM) biosynthesis	Glycan biosynthesis and metabolism	Metabolism
ko00590	0.000248928	0.000246388	0.000242685	0.000309056	Arachidonic acid metabolism	Lipid metabolism	Metabolism
ko00591	2.66423E-06	4.23052E-06	1.3683E-05	9.01312E-05	Linoleic acid metabolism	Lipid metabolism	Metabolism
ko00592	0.000499886	0.000510105	0.000531985	0.000635005	alpha-Linolenic acid metabolism	Lipid metabolism	Metabolism
ko00600	0.000134688	0.0001449	0.000184608	0.00018017	Sphingolipid metabolism	Lipid metabolism	Metabolism
ko00601	0	1.38145E-07	9.74068E-07	1.74635E-08	Glycosphingolipid biosynthesis - lacto and neolacto sc	Glycan biosynthesis and metabolism	Metabolism
ko00603	5.11776E-06	9.42169E-06	3.10224E-05	6.21066E-05	Glycosphingolipid biosynthesis - globo and isoglobo s	Glycan biosynthesis and metabolism	Metabolism
ko00604	3.48135E-06	6.69554E-06	1.84109E-05	4.89279E-05	Glycosphingolipid biosynthesis - ganglio series	Glycan biosynthesis and metabolism	Metabolism
ko00620	0.010232025	0.010276037	0.010498389	0.010924029	Pyruvate metabolism	Carbohydrate metabolism	Metabolism
ko00621	0.001089195	0.001083559	0.000999264	0.000734536	Dioxin degradation	Xenobiotics biodegradation and metabolism	Metabolism
ko00622	0.001821922	0.001815211	0.001682033	0.001330431	Xylene degradation	Xenobiotics biodegradation and metabolism	Metabolism
ko00623	0.000497205	0.000502141	0.000510204	0.000611269	Toluene degradation	Xenobiotics biodegradation and metabolism	Metabolism
ko00624	0.000611565	0.00061777	0.000625318	0.000616109	Polycyclic aromatic hydrocarbon degradation	Xenobiotics biodegradation and metabolism	Metabolism
ko00625	0.003205227	0.003193683	0.003080788	0.002813767	Chloroalkane and chloroalkene degradation	Xenobiotics biodegradation and metabolism	Metabolism
ko00626	0.001364649	0.001363095	0.001347888	0.001308937	Naphthalene degradation	Xenobiotics biodegradation and metabolism	Metabolism
ko00627	0.004165022	0.004171692	0.004086418	0.003706189	Aminobenzoate degradation	Xenobiotics biodegradation and metabolism	Metabolism
ko00630	0.010082051	0.010091649	0.010192755	0.010187533	Glyoxylate and dicarboxylate metabolism	Carbohydrate metabolism	Metabolism
ko00633	0.001097818	0.001090508	0.001009746	0.000862932	Nitrotoluene degradation	Xenobiotics biodegradation and metabolism	Metabolism
ko00640	0.008851532	0.008911522	0.009079698	0.009442994	Propanoate metabolism	Carbohydrate metabolism	Metabolism
ko00642	0.000738109	0.000745085	0.000737046	0.000725453	Ethylbenzene degradation	Xenobiotics biodegradation and metabolism	Metabolism
ko00643	0.001482631	0.001477527	0.001463986	0.001443669	Styrene degradation	Xenobiotics biodegradation and metabolism	Metabolism
ko00650	0.009794087	0.009818346	0.009872976	0.010057203	Butanoate metabolism	Carbohydrate metabolism	Metabolism
ko00660	0.00186677	0.001862687	0.001865656	0.00178118	C5-Branched dibasic acid metabolism	Carbohydrate metabolism	Metabolism

ko00670	0.001875149	0.001877235	0.001904568	0.001964436	One carbon pool by folate	Metabolism of cofactors and vitamins	Metabolism
ko00680	0.004419462	0.004400278	0.004535486	0.004609887	Methane metabolism	Energy metabolism	Metabolism
ko00710	0.001644184	0.001644903	0.001707761	0.001780923	Carbon fixation in photosynthetic organisms	Energy metabolism	Metabolism
ko00720	0.005755641	0.005772214	0.0059201	0.00609581	Carbon fixation pathways in prokaryotes	Energy metabolism	Metabolism
ko00730	0.001877567	0.001878292	0.00190746	0.001854619	Thiamine metabolism	Metabolism of cofactors and vitamins	Metabolism
ko00740	0.001138051	0.001134445	0.001173677	0.001332122	Riboflavin metabolism	Metabolism of cofactors and vitamins	Metabolism
ko00750	0.00088323	0.000886512	0.000921664	0.001037396	Vitamin B6 metabolism	Metabolism of cofactors and vitamins	Metabolism
ko00760	0.004341767	0.004357053	0.004397665	0.004618759	Nicotinate and nicotinamide metabolism	Metabolism of cofactors and vitamins	Metabolism
ko00770	0.002626031	0.002626213	0.00266696	0.002678086	Pantothenate and CoA biosynthesis	Metabolism of cofactors and vitamins	Metabolism
ko00780	0.00557862	0.005566396	0.005542998	0.005810117	Biotin metabolism	Metabolism of cofactors and vitamins	Metabolism
ko00785	0.000251531	0.000253764	0.000253873	0.000262483	Lipoic acid metabolism	Metabolism of cofactors and vitamins	Metabolism
ko00790	0.003863431	0.003853693	0.003837221	0.003869934	Folate biosynthesis	Metabolism of cofactors and vitamins	Metabolism
ko00791	0.000991421	0.000994138	0.00099365	0.000900891	Atrazine degradation	Xenobiotics biodegradation and metabolism	Metabolism
ko00830	0.000632658	0.000635201	0.00065561	0.000746945	Retinol metabolism	Metabolism of cofactors and vitamins	Metabolism
ko00860	0.005939166	0.005913712	0.005897071	0.005257164	Porphyrin and chlorophyll metabolism	Metabolism of cofactors and vitamins	Metabolism
ko00900	0.002145714	0.002162461	0.002257512	0.002399105	Terpenoid backbone biosynthesis	Metabolism of terpenoids and polyketides	Metabolism
ko00901	7.48385E-08	1.9504E-07	8.8492E-07	1.2716E-06	Indole alkaloid biosynthesis	Biosynthesis of other secondary metabolites	Metabolism
ko00902	5.15986E-08	1.33219E-07	2.48892E-07	1.00027E-06	Monoterpenoid biosynthesis	Metabolism of terpenoids and polyketides	Metabolism
ko00903	0.002941911	0.002949049	0.002859434	0.002678409	Limonene and pinene degradation	Metabolism of terpenoids and polyketides	Metabolism
ko00906	0.000134228	0.000127755	0.000136322	0.000139647	Carotenoid biosynthesis	Metabolism of terpenoids and polyketides	Metabolism
ko00908	0.000125847	0.000126058	0.000130062	0.00013058	Zeatin biosynthesis	Metabolism of terpenoids and polyketides	Metabolism
ko00909	1.58145E-06	3.92852E-07	1.0407E-06	2.15793E-07	Sesquiterpenoid and triterpenoid biosynthesis	Metabolism of terpenoids and polyketides	Metabolism
ko00910	0.004440148	0.004426461	0.004346012	0.003803077	Nitrogen metabolism	Energy metabolism	Metabolism
ko00920	0.007297869	0.007290844	0.007140975	0.006623637	Sulfur metabolism	Energy metabolism	Metabolism
ko00930	0.002462332	0.002469079	0.002447759	0.002315317	Caprolactam degradation	Xenobiotics biodegradation and metabolism	Metabolism
ko00940	0.000249325	0.000248945	0.000258055	0.00032412	Phenylpropanoid biosynthesis	Biosynthesis of other secondary metabolites	Metabolism
ko00941	6.2956E-07	1.65841E-06	3.60774E-06	2.64627E-05	Flavonoid biosynthesis	Biosynthesis of other secondary metabolites	Metabolism
ko00943	7.78461E-08	1.47808E-08	6.00705E-07	3.5319E-06	Isoflavonoid biosynthesis	Biosynthesis of other secondary metabolites	Metabolism
ko00944	6.77438E-07	5.67397E-07	4.88869E-06	5.19575E-07	Flavone and flavonol biosynthesis	Biosynthesis of other secondary metabolites	Metabolism
ko00945	6.2956E-07	1.65841E-06	3.60774E-06	2.64627E-05	Stilbenoid, diarylheptanoid and gingerol biosynthesis	Biosynthesis of other secondary metabolites	Metabolism
ko00950	0.001100982	0.001095457	0.001021526	0.000864193	Isoquinoline alkaloid biosynthesis	Biosynthesis of other secondary metabolites	Metabolism
ko00960	0.001233729	0.001231127	0.00118909	0.001140703	Tropane, piperidine and pyridine alkaloid biosynthesis	Biosynthesis of other secondary metabolites	Metabolism
ko00965	0.000363924	0.00036077	0.000332555	0.00027055	Betalain biosynthesis	Biosynthesis of other secondary metabolites	Metabolism
ko00966	0.000126228	0.000125257	0.000133668	0.000128469	Glucosinolate biosynthesis	Biosynthesis of other secondary metabolites	Metabolism
ko00970	0.003878497	0.003873576	0.003931624	0.003803998	Aminoacyl-tRNA biosynthesis	Translation	Genetic Information Processing
ko00980	0.002225226	0.002212414	0.002165666	0.002204304	Metabolism of xenobiotics by cytochrome P450	Xenobiotics biodegradation and metabolism	Metabolism
ko00981	0.001586267	0.001588929	0.001519067	0.001355624	Insect hormone biosynthesis	Metabolism of terpenoids and polyketides	Metabolism
ko00982	0.002346372	0.00233216	0.002275922	0.002278506	Drug metabolism - cytochrome P450	Xenobiotics biodegradation and metabolism	Metabolism
ko00983	0.002487202	0.002479159	0.002460438	0.002533045	Drug metabolism - other enzymes	Xenobiotics biodegradation and metabolism	Metabolism
ko00984	0.000730835	0.000735746	0.000701199	0.000643474	Steroid degradation	Xenobiotics biodegradation and metabolism	Metabolism
ko01040	0.00372796	0.003726288	0.003725574	0.004157324	Biosynthesis of unsaturated fatty acids	Lipid metabolism	Metabolism
ko01051	0.000131532	0.000134757	0.00016484	0.000178048	Biosynthesis of ansamycins	Metabolism of terpenoids and polyketides	Metabolism
ko01052	0.001447087	0.001436139	0.001338265	0.000870477	Type I polyketide structures	Metabolism of terpenoids and polyketides	Metabolism
ko01053	0.004251245	0.0042268	0.003981205	0.003316587	Biosynthesis of siderophore group nonribosomal pepti	Metabolism of terpenoids and polyketides	Metabolism
ko01054	0.007135737	0.007090947	0.006588929	0.004862669	Nonribosomal peptide structures	Metabolism of terpenoids and polyketides	Metabolism
ko01055	0.000375163	0.000376603	0.000378209	0.000385409	Biosynthesis of vancomycin group antibiotics	Metabolism of terpenoids and polyketides	Metabolism
ko01056	0.000122813	0.000125004	0.000125013	0.000114034	Biosynthesis of type II polyketide backbone	Metabolism of terpenoids and polyketides	Metabolism
ko01057	0.00012305	0.000122443	0.000125173	0.000113121	Biosynthesis of type II polyketide products	Metabolism of terpenoids and polyketides	Metabolism
ko01059	0.000486944	0.000481947	0.000447093	0.000320154	Biosynthesis of enediyne antibiotics	Metabolism of terpenoids and polyketides	Metabolism
ko01062	0.000123574	0.000121873	0.000117301	8.72021E-05	Biosynthesis of terpenoids and steroids	Chemical structure transformation maps	Metabolism
ko01100	0.133589411	0.133552095	0.133173341	0.131041022	Metabolic pathways	Global and overview maps	Metabolism
ko01110	0.052063861	0.052083643	0.052153611	0.052090408	Biosynthesis of secondary metabolites	Global and overview maps	Metabolism
ko01120	0.057834926	0.057885624	0.057673893	0.05580569	Microbial metabolism in diverse environments	Global and overview maps	Metabolism
ko01130	0.045170596	0.045238775	0.045310659	0.0452829	Biosynthesis of antibiotics	Global and overview maps	Metabolism
ko01200	0.021384604	0.021423024	0.021800492	0.022348587	Carbon metabolism	Global and overview maps	Metabolism
ko01210	0.006062466	0.006052138	0.005950739	0.005597699	2-Oxocarboxylic acid metabolism	Global and overview maps	Metabolism
ko01212	0.011310874	0.011362445	0.011541677	0.012390779	Fatty acid metabolism	Global and overview maps	Metabolism
ko01220	0.005541917	0.005542546	0.005453092	0.005123182	Degradation of aromatic compounds	Global and overview maps	Metabolism
ko01230	0.022038071	0.022044088	0.022073318	0.021226985	Biosynthesis of amino acids	Global and overview maps	Metabolism
ko01501	0.005828338	0.005809613	0.005807014	0.006194507	beta-Lactam resistance	Drug resistance: Antimicrobial	Human Diseases
ko01502	0.000762596	0.000765402	0.00080625	0.000923687	Vancomycin resistance	Drug resistance: Antimicrobial	Human Diseases
ko01503	0.004938264	0.004913827	0.004814276	0.005206454	Cationic antimicrobial peptide (CAMP) resistance	Drug resistance: Antimicrobial	Human Diseases
ko01522	0	0	4.68103E-08	0	Endocrine resistance	Drug resistance: Antineoplastic	Human Diseases
ko01523	0.000629905	0.000630474	0.000663225	0.000729513	Antifolate resistance	Drug resistance: Antineoplastic	Human Diseases
ko01524	0.002096746	0.002083952	0.002017685	0.001994779	Platinum drug resistance	Drug resistance: Antineoplastic	Human Diseases
ko02010	0.041051901	0.041088081	0.04225092	0.040631848	ABC transporters	Membrane transport	Environmental Information Processing
ko02020	0.053739045	0.05360528	0.053647766	0.059177822	Two-component system	Signal transduction	Environmental Information Processing
ko02024	0.028241698	0.028155755	0.028269492	0.026403931	Quorum sensing	Cellular community - prokaryotes	Cellular Processes
ko02025	0.010849931	0.010819453	0.010671179	0.011538014	Biofilm formation - Pseudomonas aeruginosa	Cellular community - prokaryotes	Cellular Processes
ko02026	0.00717849	0.007187012	0.007270393	0.00855479	Biofilm formation - Escherichia coli	Cellular community - prokaryotes	Cellular Processes
ko02030	0.012984477	0.012923942	0.012631618	0.012617218	Bacterial chemotaxis	Cell motility	Cellular Processes
ko02040	0.008169443	0.008115889	0.007571338	0.007098519	Flagellar assembly	Cell motility	Cellular Processes
ko02060	0.003855506	0.003788866	0.004083393	0.003568704	Phosphotransferase system (PTS)	Membrane transport	Environmental Information Processing
ko03008	0.000249568	0.00025178	0.000257505	0.000287071	Ribosome biogenesis in eukaryotes	Translation	Genetic Information Processing
ko03010	0.006782112	0.00678156	0.006939918	0.006994839	Ribosome	Translation	Genetic Information Processing
ko03013	0.000368642	0.000367195	0.000346489	0.000287935	RNA transport	Translation	Genetic Information Processing
ko03015	0	0	0	1.60972E-08	mRNA surveillance pathway	Translation	Genetic Information Processing
ko03018	0.002877434	0.00288699	0.002949667	0.00315463	RNA degradation	Folding, sorting and degradation	Genetic Information Processing
ko03020	0.000506415	0.000505621	0.000525636	0.000532512	RNA polymerase	Transcription	Genetic Information Processing
ko03022	1.41427E-06	3.03213E-06	1.51732E-05	2.26341E-05	Basal transcription factors	Transcription	Genetic Information Processing
ko03030	0.001779723	0.001789671	0.001896376	0.00218329	DNA replication	Replication and repair	Genetic Information Processing
ko03040	0	2.48639E-08	7.82229E-08	1.15834E-09	Spliceosome	Transcription	Genetic Information Processing
ko03050	2.83213E-06	6.67953E-06	1.49414E-05	4.81966E-06	Proteasome	Folding, sorting and degradation	Genetic Information Processing
ko03060	0.002141255	0.002142351	0.002195154	0.00233988	Protein export	Folding, sorting and degradation	Genetic Information Processing
ko03070	0.009788739	0.009749281	0.009294326	0.008583315	Bacterial secretion system	Membrane transport	Environmental Information Processing
ko03320	0.001878936	0.001910645	0.002068612	0.002324987	PPAR signaling pathway	Endocrine system	Organismal Systems
ko03410	0.001879911	0.001887958	0.001915505	0.002074422	Base excision repair	Replication and repair	Genetic Information Processing
ko03420	0.001380361	0.001388535	0.00141917	0.001458646	Nucleotide excision repair	Replication and repair	Genetic Information Processing
ko03430	0.002390064	0.002389802	0.002467167	0.002666533	Mismatch repair	Replication and repair	Genetic Information Processing
ko03440	0.002532744	0.002539282	0.002665134	0.003010128	Homologous recombination	Replication and repair	Genetic Information Processing
ko03450	6.973E-06	1.0532E-05	3.28537E-05	0.000146776	Non-homologous end-joining	Replication and repair	Genetic Information Processing
ko03460	1.48817E-10	8.91604E-10	2.18604E-08	4.36256E-09	Fanconi anemia pathway	Replication and repair	Genetic Information Processing
ko04011	0.000128106	0.000130337	0.000138029	0.000195698	MAPK signaling pathway - yeast	Signal transduction	Environmental Information Processing
ko04013	0.000127219	0.000128591	0.000137002	0.000212624	MAPK signaling pathway - fly	Signal transduction	Environmental Information Processing
ko04014	6.94711E-07	3.15051E-07	1.06154E-06	1.61452E-05	Ras signaling pathway	Signal transduction	Environmental Information Processing
ko04016	0.000631024	0.000636789	0.000642268	0.000705641	MAPK signaling pathway - plant	Signal transduction	Environmental Information Processing
ko04020	2.50614E-09	1.36982E-08	1.36965E-08	1.9578E-07	Calcium signaling pathway	Signal transduction	Environmental Information Processing
ko04022	8.85597E-08	4.61743E-08	1.6024E-06	1.54509E-06	cGMP-PKG signaling pathway	Signal transduction	Environmental Information Processing
ko04024	1.34683E-06	2.33204E-06	3.65719E-06	2.06158E-05	cAMP signaling pathway	Signal transduction	Environmental Information Processing
ko04064	0	3.96447E-08	4.09027E-08	4.07868E-09	NF-kappa B signaling pathway	Signal transduction	Environmental Information Processing
ko04066	0.000506596	0.000508922	0.000510002	0.000523256	HIF-1 signaling pathway	Signal transduction	Environmental Information Processing
ko04068	0.000377013	0.000381988	0.00039147	0.000495567	FoxO signaling pathway	Signal transduction	Environmental Information Processing
ko04070	0.000378721	0.000378171	0.000384473	0.000454972	Phosphatidylinositol signaling system	Signal transduction	Environmental Information Processing
ko04071	8.26231E-07	4.50172E-07	3.39882E-06	1.85724E-05	Sphingolipid signaling pathway	Signal transduction	Environmental Information Processing
ko04072	0.000124135	0.000123185	0.000118972	0.000167037	Phospholipase D signaling pathway	Signal transduction	Environmental Information Processing
ko04080	2.25121E-06	1.32581E-06	3.84707E-06	4.32817E-05	Neuroactive ligand-receptor interaction	Signaling molecules and interaction	Environmental Information Processing
ko04112	0.005019563	0.00498431	0.005091636	0.006153243	Cell cycle - Caulobacter	Cell growth and death	Cellular Processes
ko04113	0.000135986	0.000133718	0.000151008	0.000249109	Meiosis - yeast	Cell growth and death	Cellular Processes

ko04115	0.000125921	0.000123676	0.000126785	0.000135403	p53 signaling pathway	Cell growth and death	Cellular Processes
ko04122	0.001751326	0.001745037	0.001771465	0.001844904	Sulfur relay system	Folding, sorting and degradation	Genetic Information Processing
ko04138	1.50804E-06	2.9092E-06	7.02523E-06	2.49643E-06	Autophagy - yeast	Transport and catabolism	Cellular Processes
ko04140	1.44564E-07	1.13065E-07	4.83044E-09	0	Autophagy - animal	Transport and catabolism	Cellular Processes
ko04141	0.000374459	0.000377676	0.000374203	0.000348444	Protein processing in endoplasmic reticulum	Folding, sorting and degradation	Genetic Information Processing
ko04142	0.000131387	0.000138122	0.000178611	0.000250902	Lysosome	Transport and catabolism	Cellular Processes
ko04144	9.04152E-07	3.16376E-07	1.15252E-06	1.7537E-05	Endocytosis	Transport and catabolism	Cellular Processes
ko04146	0.001649241	0.001682345	0.001904597	0.002412594	Peroxisome	Transport and catabolism	Cellular Processes
ko04151	0.000245763	0.000247035	0.000238544	0.000243831	PI3K-Akt signaling pathway	Signal transduction	Environmental Information Processing
ko04152	0.000492025	0.000492424	0.000478088	0.000467957	AMPK signaling pathway	Signal transduction	Environmental Information Processing
ko04210	0.000249679	0.000245321	0.000244111	0.000247132	Apoptosis	Cell growth and death	Cellular Processes
ko04211	0.000255325	0.000258928	0.000275032	0.000408322	Longevity regulating pathway	Aging	Organismal Systems
ko04212	0.001123149	0.001128097	0.001137277	0.001264701	Longevity regulating pathway - worm	Aging	Organismal Systems
ko04213	0.000884889	0.000888185	0.000913577	0.001126032	Longevity regulating pathway - multiple species	Aging	Organismal Systems
ko04214	0.000617964	0.000609144	0.000586382	0.000555428	Apoptosis - fly	Cell growth and death	Cellular Processes
ko04215	0.000125918	0.000123676	0.000126733	0.000135366	Apoptosis - multiple species	Cell growth and death	Cellular Processes
ko04216	0.000754838	0.000770575	0.000859736	0.000994676	Ferroptosis	Cell growth and death	Cellular Processes
ko04217	0.000745731	0.000748931	0.00074951	0.000734234	Necroptosis	Cell growth and death	Cellular Processes
ko04260	0.000369485	0.000365056	0.000351244	0.000373095	Cardiac muscle contraction	Circulatory system	Organismal Systems
ko04261	8.85597E-08	4.61743E-08	1.6024E-06	1.54509E-06	Adrenergic signaling in cardiomyocytes	Circulatory system	Organismal Systems
ko04310	5.45697E-12	1.23573E-09	1.72698E-07	1.54336E-08	Wnt signaling pathway	Signal transduction	Environmental Information Processing
ko04320	0	0	4.68103E-08	0	Dorso-ventral axis formation	Development	Organismal Systems
ko04330	5.45697E-12	1.23573E-09	2.19508E-07	1.54336E-08	Notch signaling pathway	Signal transduction	Environmental Information Processing
ko04370	0	3.96447E-08	4.09027E-08	4.07868E-09	VEGF signaling pathway	Signal transduction	Environmental Information Processing
ko04380	1.39577E-07	2.60436E-07	1.55008E-07	2.21189E-06	Osteoclast differentiation	Development	Organismal Systems
ko04611	0	0	2.44213E-08	0	Platelet activation	Immune system	Organismal Systems
ko04612	0.000123938	0.000123581	0.00012072	0.000156597	Antigen processing and presentation	Immune system	Organismal Systems
ko04614	2.42453E-06	3.12176E-06	7.18623E-06	6.76828E-05	Renin-angiotensin system	Endocrine system	Organismal Systems
ko04621	0.000864585	0.000871351	0.000856981	0.001006974	NOD-like receptor signaling pathway	Immune system	Organismal Systems
ko04622	2.27423E-06	2.73877E-06	1.57518E-05	2.56692E-05	RIG-I-like receptor signaling pathway	Immune system	Organismal Systems
ko04625	0	3.96447E-08	4.09027E-08	4.07868E-09	C-type lectin receptor signaling pathway	Immune system	Organismal Systems
ko04626	0.000867536	0.000863321	0.000846982	0.00093325	Plant-pathogen interaction	Environmental adaptation	Organismal Systems
ko04640	0	5.95813E-09	0	3.86114E-09	Hematopoietic cell lineage	Immune system	Organismal Systems
ko04657	0.000123938	0.00012362	0.000120761	0.000156602	IL-17 signaling pathway	Immune system	Organismal Systems
ko04658	0	0	4.68103E-08	0	Th1 and Th2 cell differentiation	Immune system	Organismal Systems
ko04659	0.000123938	0.000123581	0.00012072	0.000156597	Th17 cell differentiation	Immune system	Organismal Systems
ko04664	0	0	0	4.58852E-09	Fc epsilon RI signaling pathway	Immune system	Organismal Systems
ko04666	6.94711E-07	3.15051E-07	1.06154E-06	1.61452E-05	Fc gamma R-mediated phagocytosis	Immune system	Organismal Systems
ko04668	0	3.96447E-08	4.09027E-08	4.07868E-09	TNF signaling pathway	Signal transduction	Environmental Information Processing
ko04714	0.001251661	0.001263224	0.001342396	0.001518961	Thermogenesis	Environmental adaptation	Organismal Systems
ko04721	0	2.17091E-08	6.35292E-09	2.45394E-09	Synaptic vesicle cycle	Nervous system	Organismal Systems
ko04723	0.000120746	0.000119091	0.000104582	6.69414E-05	Retrograde endocannabinoid signaling	Nervous system	Organismal Systems
ko04724	0.000372348	0.000374766	0.000356683	0.000328799	Glutamatergic synapse	Nervous system	Organismal Systems
ko04726	0.000122152	0.000120387	0.000111998	7.69563E-05	Serotonergic synapse	Nervous system	Organismal Systems
ko04727	0.000371895	0.000375112	0.000356794	0.000324939	GABAergic synapse	Nervous system	Organismal Systems
ko04728	0.000122152	0.000120353	0.000111977	7.69599E-05	Dopaminergic synapse	Nervous system	Organismal Systems
ko04910	0.00024855	0.000251022	0.000256606	0.000250962	Insulin signaling pathway	Endocrine system	Organismal Systems
ko04911	8.85597E-08	4.61743E-08	1.6024E-06	1.54509E-06	Insulin secretion	Endocrine system	Organismal Systems
ko04912	6.94711E-07	3.15051E-07	1.06154E-06	1.61452E-05	GnRH signaling pathway	Endocrine system	Organismal Systems
ko04913	0	3.96447E-08	4.09027E-08	8.6672E-09	Ovarian steroidogenesis	Endocrine system	Organismal Systems
ko04914	0.000123938	0.000123581	0.00012072	0.000156597	Progesterone-mediated oocyte maturation	Endocrine system	Organismal Systems
ko04915	0.000123938	0.000123581	0.00012072	0.000156597	Estrogen signaling pathway	Endocrine system	Organismal Systems
ko04916	0.000240591	0.000238779	0.000214181	0.000148001	Melanogenesis	Endocrine system	Organismal Systems
ko04917	6.6503E-07	6.81085E-07	4.26225E-06	1.49231E-05	Prolactin signaling pathway	Endocrine system	Organismal Systems
ko04918	0.000494204	0.000489636	0.000475701	0.000491814	Thyroid hormone synthesis	Endocrine system	Organismal Systems
ko04919	0.000361802	0.000357572	0.000321403	0.000226309	Thyroid hormone signaling pathway	Endocrine system	Organismal Systems
ko04920	0.000632481	0.000650007	0.000744535	0.000861047	Adipocytokine signaling pathway	Endocrine system	Organismal Systems
ko04921	7.5795E-09	5.24719E-08	9.50924E-08	3.33897E-08	Oxytocin signaling pathway	Endocrine system	Organismal Systems
ko04922	0.000752602	0.000753956	0.000764931	0.000669146	Glucagon signaling pathway	Endocrine system	Organismal Systems
ko04923	0	3.96447E-08	4.09027E-08	4.07868E-09	Regulation of lipolysis in adipocytes	Endocrine system	Organismal Systems
ko04924	7.0797E-07	6.98432E-07	1.25729E-06	3.11132E-05	Renin secretion	Endocrine system	Organismal Systems
ko04925	8.85597E-08	4.61743E-08	1.6024E-06	1.54509E-06	Aldosterone synthesis and secretion	Endocrine system	Organismal Systems
ko04926	0	0	2.44213E-08	0	Relaxin signaling pathway	Endocrine system	Organismal Systems
ko04928	7.14343E-07	5.99778E-07	1.59029E-06	2.36873E-05	Parathyroid hormone synthesis, secretion and action	Endocrine system	Organismal Systems
ko04930	0.000246564	0.000246852	0.000235839	0.000205007	Type II diabetes mellitus	Endocrine and metabolic diseases	Human Diseases
ko04931	0.000253329	0.000259131	0.000291573	0.000336801	Insulin resistance	Endocrine and metabolic diseases	Human Diseases
ko04932	0.000495459	0.000488799	0.000476379	0.000506915	Non-alcoholic fatty liver disease (NAFLD)	Endocrine and metabolic diseases	Human Diseases
ko04933	0	0	2.44213E-08	0	AGE-RAGE signaling pathway in diabetic complicati	Endocrine and metabolic diseases	Human Diseases
ko04934	0.000127001	0.0001275	0.000140598	0.000151111	Cushing's syndrome	Endocrine and metabolic diseases	Human Diseases
ko04940	0.000127382	0.000128639	0.000135329	0.000145478	Type I diabetes mellitus	Endocrine and metabolic diseases	Human Diseases
ko04960	8.85597E-08	4.61743E-08	1.6024E-06	1.54509E-06	Aldosterone-regulated sodium reabsorption	Excretory system	Organismal Systems
ko04961	8.85597E-08	4.61743E-08	1.6024E-06	1.54509E-06	Endocrine and other factor-regulated calcium reabsorp	Excretory system	Organismal Systems
ko04962	0	2.17091E-08	6.35292E-09	0	Vasopressin-regulated water reabsorption	Excretory system	Organismal Systems
ko04964	0.000367064	0.0003708	0.000343556	0.000253156	Proximal tubule bicarbonate reclamation	Excretory system	Organismal Systems
ko04970	8.85597E-08	4.61743E-08	1.6024E-06	1.54509E-06	Salivary secretion	Digestive system	Organismal Systems
ko04971	8.85597E-08	4.61743E-08	1.6024E-06	1.54509E-06	Gastric acid secretion	Digestive system	Organismal Systems
ko04972	3.11836E-07	2.02908E-07	2.49767E-06	2.01127E-05	Pancreatic secretion	Digestive system	Organismal Systems
ko04973	1.55564E-06	3.23141E-06	1.47766E-05	2.719E-05	Carbohydrate digestion and absorption	Digestive system	Organismal Systems
ko04974	3.45934E-06	5.93222E-06	1.6689E-05	8.43313E-05	Protein digestion and absorption	Digestive system	Organismal Systems
ko04976	1.04994E-06	4.71915E-07	2.5108E-06	7.63478E-06	Bile secretion	Digestive system	Organismal Systems
ko04978	0.000126172	0.000126438	0.000128274	0.00011478	Mineral absorption	Digestive system	Organismal Systems
ko04979	2.02793E-06	1.16908E-06	2.9518E-06	2.4714E-05	Cholesterol metabolism	Digestive system	Organismal Systems
ko05010	0.000623263	0.000618552	0.000611063	0.000669098	Alzheimer's disease	Neurodegenerative diseases	Human Diseases
ko05012	0.000495315	0.000488686	0.000476374	0.000506918	Parkinson's disease	Neurodegenerative diseases	Human Diseases
ko05014	0.000377028	0.000376646	0.000382825	0.000477223	Amyotrophic lateral sclerosis (ALS)	Neurodegenerative diseases	Human Diseases
ko05016	0.000745538	0.00073991	0.000731447	0.000865708	Huntington's disease	Neurodegenerative diseases	Human Diseases
ko05020	0.000123004	0.000122633	0.000118109	0.000146159	Prion diseases	Neurodegenerative diseases	Human Diseases
ko05030	0.000122152	0.000120347	0.000111957	7.69477E-05	Cocaine addiction	Substance dependence	Human Diseases
ko05031	0.000122152	0.000120347	0.000111957	7.69477E-05	Amphetamine addiction	Substance dependence	Human Diseases
ko05034	0.000122152	0.000120347	0.000111989	7.95232E-05	Alcoholism	Substance dependence	Human Diseases
ko05100	1.1979E-06	1.05018E-06	4.19565E-06	1.28879E-05	Bacterial invasion of epithelial cells	Infectious diseases: Bacterial	Human Diseases
ko05110	0.000121168	0.000121589	0.000112316	0.000135377	Vibrio cholerae infection	Infectious diseases: Bacterial	Human Diseases
ko05111	0.008725652	0.008704258	0.008516746	0.00887372	Biofilm formation - Vibrio cholerae	Cellular community - prokaryotes	Cellular Processes
ko05120	0.00049641	0.000496173	0.000503803	0.000507005	Epithelial cell signaling in Helicobacter pylori infectio	Infectious diseases: Bacterial	Human Diseases
ko05130	7.75072E-09	1.92499E-08	5.57713E-08	2.34666E-07	Pathogenic Escherichia coli infection	Infectious diseases: Bacterial	Human Diseases
ko05131	4.54768E-09	3.51861E-08	1.7268E-07	1.68726E-06	Shigellosis	Infectious diseases: Bacterial	Human Diseases
ko05132	0.000984564	0.000985546	0.000972334	0.001035923	Salmonella infection	Infectious diseases: Bacterial	Human Diseases
ko05133	0.002082362	0.002074404	0.001974158	0.002006031	Pertussis	Infectious diseases: Bacterial	Human Diseases
ko05134	0.000997461	0.000994864	0.000993025	0.001019073	Legionellosis	Infectious diseases: Bacterial	Human Diseases
ko05140	0	3.96447E-08	4.09027E-08	4.07868E-09	Leishmaniasis	Infectious diseases: Parasitic	Human Diseases
ko05142	2.90326E-06	4.63448E-06	5.70967E-06	7.72321E-05	Chagas disease (American trypanosomiasis)	Infectious diseases: Parasitic	Human Diseases
ko05143	2.39122E-06	4.23527E-06	6.65737E-06	4.72083E-05	African trypanosomiasis	Infectious diseases: Parasitic	Human Diseases
ko05145	0.000125918	0.000123676	0.000126733	0.000135371	Toxoplasmosis	Infectious diseases: Parasitic	Human Diseases
ko05146	0.000242359	0.000240483	0.000222762	0.000182197	Amoebiasis	Infectious diseases: Parasitic	Human Diseases
ko05150	0.00049487	0.000483505	0.000483485	0.000400139	Staphylococcus aureus infection	Infectious diseases: Bacterial	Human Diseases
ko05152	0.000870971	0.000868465	0.000858025	0.000839498	Tuberculosis	Infectious diseases: Bacterial	Human Diseases
ko05160	1.44564E-07	1.13065E-07	4.83044E-09	0	Hepatitis C	Infectious diseases: Viral	Human Diseases
ko05161	0.000125918	0.000123676	0.000126733	0.000135366	Hepatitis B	Infectious diseases: Viral	Human Diseases

ko05162	1.44564E-07	1.13065E-07	4.83044E-09	0	Measles	Infectious diseases: Viral	Human Diseases
ko05163	0.000125918	0.000123716	0.000126774	0.00013537	Human cytomegalovirus infection	Infectious diseases: Viral	Human Diseases
ko05164	0.000126291	0.000123953	0.000127638	0.000154537	Influenza A	Infectious diseases: Viral	Human Diseases
ko05165	0.000246563	0.000246713	0.000235772	0.000207586	Human papillomavirus infection	Infectious diseases: Viral	Human Diseases
ko05166	2.02793E-06	1.16908E-06	2.9518E-06	2.4714E-05	HTLV-I infection	Infectious diseases: Viral	Human Diseases
ko05167	0.000125918	0.000123716	0.000126774	0.00013537	Kaposi's sarcoma-associated herpesvirus infection	Infectious diseases: Viral	Human Diseases
ko05168	0.000126062	0.000123789	0.000126746	0.000135375	Herpes simplex infection	Infectious diseases: Viral	Human Diseases
ko05169	0	0	7.76481E-09	8.94347E-09	Epstein-Barr virus infection	Infectious diseases: Viral	Human Diseases
ko05200	0.002092147	0.002077199	0.00202045	0.002080181	Pathways in cancer	Cancers: Overview	Human Diseases
ko05203	0.000246563	0.000246673	0.000235684	0.000207582	Viral carcinogenesis	Cancers: Overview	Human Diseases
ko05204	0.001725529	0.00171373	0.001660436	0.001712614	Chemical carcinogenesis	Cancers: Overview	Human Diseases
ko05205	0.000126174	0.000127173	0.000134682	0.0001486	Proteoglycans in cancer	Cancers: Overview	Human Diseases
ko05206	0.000370185	0.000370838	0.000350039	0.000284869	MicroRNAs in cancer	Cancers: Overview	Human Diseases
ko05210	0.000125918	0.000123676	0.000126733	0.000135366	Colorectal cancer	Cancers: Specific types	Human Diseases
ko05211	0.000127001	0.0001275	0.000140598	0.000151111	Renal cell carcinoma	Cancers: Specific types	Human Diseases
ko05215	0.000123938	0.000123581	0.00012072	0.000156597	Prostate cancer	Cancers: Specific types	Human Diseases
ko05219	2.92135E-06	2.92306E-06	7.27802E-06	1.62697E-05	Bladder cancer	Cancers: Specific types	Human Diseases
ko05220	5.45697E-12	1.23573E-09	1.72698E-07	1.54336E-08	Chronic myeloid leukemia	Cancers: Specific types	Human Diseases
ko05222	0.000125918	0.000123716	0.000126774	0.00013537	Small cell lung cancer	Cancers: Specific types	Human Diseases
ko05224	0	0	4.68103E-08	0	Breast cancer	Cancers: Specific types	Human Diseases
ko05225	0.00171529	0.001702401	0.001632138	0.001637087	Hepatocellular carcinoma	Cancers: Specific types	Human Diseases
ko05230	0.000875427	0.000878341	0.000853537	0.000758649	Central carbon metabolism in cancer	Cancers: Overview	Human Diseases
ko05231	0.000124743	0.000123427	0.000122237	0.000167147	Choline metabolism in cancer	Cancers: Overview	Human Diseases
ko05322	7.49742E-07	1.31335E-07	1.14194E-06	1.18036E-05	Systemic lupus erythematosus	Immune diseases	Human Diseases
ko05323	1.39577E-07	2.60436E-07	1.55008E-07	2.21189E-06	Rheumatoid arthritis	Immune diseases	Human Diseases
ko05340	0.000247153	0.000248954	0.000245603	0.000228894	Primary immunodeficiency	Immune diseases	Human Diseases
ko05410	1.19722E-06	1.48789E-06	4.64956E-06	4.43882E-05	Hypertrophic cardiomyopathy (HCM)	Cardiovascular diseases	Human Diseases
ko05414	4.89255E-07	7.8946E-07	3.39226E-06	1.3275E-05	Dilated cardiomyopathy (DCM)	Cardiovascular diseases	Human Diseases
ko05416	0.000125918	0.000123676	0.000126733	0.000135366	Viral myocarditis	Cardiovascular diseases	Human Diseases
ko05418	0.002458848	0.002454282	0.002376496	0.002451415	Fluid shear stress and atherosclerosis	Cardiovascular diseases	Human Diseases
