# Supplementary material for: Multiscale Plant Defense Strategies against Ciprofloxacin Stress: From Chloroplast-Centered Adaptation to Microbiome Coordination
Source: Research (Wash D C). 2026 Jan 15;9:1082. doi: 10.34133/research.1082 (PMC12804602; doi:10.34133/research.1082)
Supplement: Supplementary 1 — Description of Supplemental Methods Figs. S1 to S4 Tables S1 to S9 [file research.1082.f1.zip › Supplemental Materials.docx]

***Supplemental Materials***

**Multi-Scale Plant Defense Strategies Against Ciprofloxacin Stress: From Chloroplast-Centered Adaptation to Microbiome Coordination**

Chen Ling ^1,6^, Xiaohan Chen^1^, Jing Yang ^2^, Xinhua Zhan ^3^, Jason C. White ^4^, Melanie Kah ^5^, Yu Shen ^1,5*^, Baoshan Xing ^6*^

^1^ Co-Innovation Center for Sustainable Forestry in Southern China, College of Ecology and Environment, National Positioning Observation Station of Hung-tse Lake Wetland Ecosystem in Jiangsu Province, Nanjing, Jiangsu 210037, China; Nanjing Forestry University, Nanjing 210037, China;

^2^ Electron Microscope Lab, Advance Analysis and Test Center, Nanjing Forestry University, Nanjing 210037, China;

^3^ College of Resources and Environmental Sciences, Nanjing Agricultural University, Nanjing 210095, China;

^4^ The Connecticut Agricultural Experiment Station, New Haven, CT, 06504, USA;

^5^ School of Environment, University of Auckland, Private Bag 92019, Auckland, 1010, New Zealand;

^6^ Stockbridge School of Agriculture, University of Massachusetts, Amherst, MA, 01003, United States.

Corresponding authors

Yu Shen, Ph.D., Email: [sheyttmax@hotmail.com/yushen@njfu.edu.cn](mailto:sheyttmax@hotmail.com/yushen@njfu.edu.cn);

Baoshan Xing, Ph.D., Email: [bx@umass.edu](mailto:bx@umass.edu).

**Contents**

[**Section 1. Quantitative Proteomics 1**](#_Toc13530)

[**Table S1. Differential protein 5 mg L^-1^.vs.Control 1**](#_Toc4564)

[**Table S2. Differential protein 10 mg L^-1^.vs.Control 4**](#_Toc30584)

[**Table S3. Differential protein 20 mg L^-1^.vs.Control 10**](#_Toc32451)

[**Table S4. Differential protein subcellular localization 5 mg L^-1^.vs.Control 31**](#_Toc30314)

[**Table S5. Differential protein subcellular localization 10 mg L^-1^.vs.Control 32**](#_Toc1099)

[**Table S6. Differential protein subcellular localization 20 mg L^-1^.vs.Control 35**](#_Toc25558)

[**Table S7. Peptide fraction separation by liquid chromatography elution gradient table 44**](#_Toc12958)

[**Section 2. The Carotenoids of Leaves 45**](#_Toc9021)

[**Section 3. Influence of Ciprofloxacin on Media pH 46**](#_Toc2789)

**Section 4. The biodegradation of Ciprofloxacin in plant cells …………………………………………..49**

# Section 1. Quantitative Proteomics

## Table S1. Differential protein 5 mg L^-1^.vs.Control

| **Table S1. Differential protein 5 mg L^-1^.vs.Control** | | | | |
| --- | --- | --- | --- | --- |
| **No.** | **Protein** | **Description** | **Gene** | **Up/down** |
| 1 | Q339G9 | Ribulose bisphosphate carboxylase large chain | LOC_Os10g21280 | up |
| 2 | G8CTM8 | Ribulose bisphosphate carboxylase/oxygenase activase, chloroplastic |  | up |
| 3 | B9FJH8 | Os05g0460000 protein | Os05g0460000 | up |
| 4 | A0A5S6RD74 | Phenylalanine ammonia-lyase | Os04g0518100 | up |
| 5 | Q76FS2 | Tubulin beta-8 chain | TUBB8 | up |
| 6 | A3AW25 | Uncharacterized protein | OsJ_15655 | up |
| 7 | Q5Z790 | Os06g0531200 protein | Os06g0531200 | up |
| 8 | A3A985 | Phenylalanine ammonia-lyase | Os02g0626400 | up |
| 9 | Q75IZ9 | Glucose-6-phosphate 1-dehydrogenase | LOC_Os03g29950 | up |
| 10 | A0A0P0VKA8 | Os02g0554100 protein | Os02g0554100 | up |
| 11 | A0A5S6RBX8 | Eukaryotic translation initiation factor 3 subunit D | Os05g0566500 | up |
| 12 | A0A0P0WWY2 | 1,4-alpha-glucan branching enzyme | Os06g0367100 | up |
| 13 | Q6Z0I4 | Enoyl-[acyl-carrier-protein] reductase [NADH] 1, chloroplastic | Os08g0327400 | up |
| 14 | P49030 | Protein mago nashi homolog 2 | MAGO2 | up |
| 15 | Q9LGH8 | Histone H2B.8 | H2B.8 | up |
| 16 | P48489 | Serine/threonine-protein phosphatase PP1 | Os03g0268000 | up |
| 17 | D7PPK3 | ADH1 | adh1 | up |
| 18 | A0A8J8YN91 | Phospho-2-dehydro-3-deoxyheptonate aldolase | OsJ_11111 | up |
| 19 | Q2QYY2 | Leucine-rich repeat family protein, putative, expressed | LOC_Os12g01200 | up |
| 20 | Q0JLB5 | Os01g0611000 protein (Fragment) | Os01g0611000 | up |
| 21 | A0A8J8XXT2 | Os03g0116500 protein | Os03g0116500 | up |
| 22 | A3AAM6 | ArsA_ATPase domain-containing protein | OsJ_08118 | up |
| 23 | Q8GVF5 | Eukaryotic translation initiation factor 6 | EIF6 | up |
| 24 | Q10MC7 | RNA helicase | LOC_Os03g19960 | up |
| 25 | Q0DC50 | Immunophilin-like | Os06g0484500 | up |
| 26 | Q2QUI0 | Glutathione synthetase | LOC_Os12g16200 | up |
| 27 | B9FIA4 | Os05g0190300 protein | Os05g0190300 | up |
| 28 | Q6YVH6 | Os02g0705100 protein | Os02g0705100 | up |
| 29 | Q5N801 | peptidylprolyl isomerase | Os01g0915800 | up |
| 30 | Q8H367 | Os07g0413800 protein | Os07g0413800 | up |
| 31 | Q8LR34 | Iron-sulfur cluster assembly protein 1 | ISU1 | up |
| 32 | Q10MG3 | Os03g0309800 protein | LOC_Os03g19650 | up |
| 33 | B9FAM9 | Ferredoxin | OsJ_12142 | up |
| 34 | Q7XUY5 | Os04g0465600 protein | Os04g0465600 | up |
| 35 | A0A7S8BAT2 | 30S ribosomal protein S11, chloroplastic | rps11 | up |
| 36 | Q7XXR4 | Os08g0243600 protein | Os08g0243600 | up |
| 37 | Q0DEK1 | Os06g0148200 protein | P0036F10.45-1 | up |
| 38 | A0A0P0VGU6 | Peroxidase | Os02g0236800 | up |
| 39 | B9GE32 | Uncharacterized protein | OsJ_36748 | up |
| 40 | B9F6B9 | Os03g0784400 protein | OSJNBb0093E13.1 | up |
| 41 | Q7XR06 | Probable protein phosphatase 2C 45 | Os04g0659500 | up |
| 42 | Q0DJ33 | Guanine nucleotide-binding protein alpha-1 subunit | GPA1 | up |
| 43 | Q0IT01 | Os11g0433900 protein | OJ1612_A04.113 | up |
| 44 | Q5JNF3 | Os01g0730500 protein | Os01g0730500 | up |
| 45 | Q0DTG6 | Os03g0249200 protein | Os03g0249200 | up |
| 46 | A0A8J8Y991 | Os01g0839900 protein | Os01g0839900 | up |
| 47 | A3C4F7 | Uncharacterized protein | OsJ_31413 | up |
| 48 | Q2QX16 | Expressed protein | LOC_Os12g07500 | up |
| 49 | Q8HCQ0 | NADH-ubiquinone oxidoreductase chain 3 | nad3 | up |
| 50 | Q84LM2 | Vacuolar-processing enzyme beta-isozyme 1 | VPE1 | up |
| 51 | Q653G8 | Translation machinery associated TMA7 | P0623A10.17-1 | up |
| 52 | Q657X8 | Peroxisomal membrane protein PEX14 | P0005A05.25-1 | up |
| 53 | A0A0P0UZ45 | Os01g0182200 protein | Os01g0182200 | up |
| 54 | B9EVU0 | ABC1 atypical kinase-like domain-containing protein | OsJ_01512 | up |
| 55 | A3A3E9 | Phytocyanin domain-containing protein | OsJ_05484 | up |
| 1 | B9F6U6 | Glutamine amidotransferase domain-containing protein | OsJ_10181 | down |
| 2 | A3CCT6 | RNA helicase | OsJ_34439 | down |
| 3 | B9FKW6 | Proteasome subunit alpha type | OsJ_19022 | down |
| 4 | Q0ITU4 | Os11g0216100 protein | Os11g0216100 | down |
| 5 | Q10FT7 | Hsp20/alpha crystallin family protein, expressed | LOC_Os03g45340 | down |
| 6 | Q10G20 | DNA-directed RNA polymerase subunit beta | LOC_Os03g44484 | down |
| 7 | Q6K5H0 | Cytosolic Fe-S cluster assembly factor NBP35 | NBP35 | down |
| 8 | Q69U53 | Os08g0103500 protein | Os08g0103500 | down |
| 9 | Q0JF69 | Os04g0137500 protein (Fragment) | Os04g0137500 | down |
| 10 | B9F269 | RRM domain-containing protein | OsJ_05182 | down |
| 11 | Q0IZL5 | Os09g0562700 protein (Fragment) | Os09g0562700 | down |
| 12 | B9FLV6 | RNA polymerase sigma factor | OsJ_19735 | down |
| 13 | Q5Z414 | Os06g0730800 protein | Os06g0730800 | down |
| 14 | A0A0P0V1F4 | Os01g0259600 protein | Os01g0259600 | down |
| 15 | Q0JI70 | Os01g0819900 protein | Os01g0819900 | down |
| 16 | Q2QU14 | Expressed protein | LOC_Os12g17830 | down |
| 17 | Q94I55 | Probable dolichyl-diphosphooligosaccharide-protein glycosyltransferase subunit 3 | OST3 | down |
| 18 | A3BXT3 | Os09g0346700 protein | P0512H04.9-1 | down |
| 19 | Q10P76 | Expressed protein | LOC_Os03g14040 | down |
| 20 | A3A0G8 | Os01g0894500 protein | Os01g0894500 | down |
| 21 | Q5ZC82 | Cytokinin riboside 5-monophosphate phosphoribohydrolase LOG | LOG | down |
| 22 | Q6YZU8 | p53 binding protein-like | OJ1003_A09.35-2 | down |
| 23 | A0A0P0XKP6 | Chlorophyll a-b binding protein, chloroplastic | Os09g0296800 | down |
| 24 | Q6ES52 | TPR repeat-containing thioredoxin TDX | Os09g0401200 | down |
| 25 | B9EV51 | Rab proteins geranylgeranyltransferase component | OsJ_01236 | down |
| 26 | A3BWG0 | U4/U6 small nuclear ribonucleoprotein Prp3 | OsJ_28520 | down |
| 27 | A0A0P0WCB4 | OSJNBa0067K08.9 protein | Os04g0496400 | down |
| 28 | E3WF09 | Resistant protein | r | down |
| 29 | B9ETN3 | Protein kinase domain-containing protein | OsJ_00747 | down |
| 30 | Q7XRF0 | OSJNBa0006M15.17 protein | OSJNBa0006M15.17 | down |
| 31 | A0A8J8XD15 | Zinc finger protein-like | OsJ_22693 | down |
| 32 | Q5VR53 | Os01g0807900 protein | P0702B09.41-2 | down |
| 33 | Q0IW04 | chitinase | OSJNBb0015I11.16 | down |

## Table S2. Differential protein 10 mg L^-1^.vs.Control

| **Table S2. Differential protein 10 mg L^-1^.vs.Control** | | | | |
| --- | --- | --- | --- | --- |
| **No.** | **Protein** | **Description** | **Gene** | **Up/down** |
| 1 | A0A0P0Y838 | Ribulose bisphosphate carboxylase large chain | Os12g0207600 | up |
| 2 | G8CTM8 | Ribulose bisphosphate carboxylase/oxygenase activase, chloroplastic |  | up |
| 3 | B9FRD6 | phosphopyruvate hydratase | OsJ_20044 | up |
| 4 | B9FI18 | NAD-dependent epimerase/dehydratase domain-containing protein | OsJ_18396 | up |
| 5 | Q2RAP8 | 2-isopropylmalate synthase | LOC_Os11g04670 | up |
| 6 | Q65XK0 | Ketol-acid reductoisomerase, chloroplastic | Os05g0573700 | up |
| 7 | B9GCW1 | Ribulose bisphosphate carboxylase large subunit C-terminal domain-containing protein | OsJ_35903 | up |
| 8 | Q2QXR8 | Pyruvate kinase 2, cytosolic | Os12g0145700 | up |
| 9 | Q0J4W9 | Phospho-2-dehydro-3-deoxyheptonate aldolase | Os08g0484500 | up |
| 10 | Q8LJJ2 | threonine synthase | Os01g0693800 | up |
| 11 | A0A8J8YSG5 | Methyltransf_11 domain-containing protein | OsJ_19441 | up |
| 12 | B9FY88 | Eukaryotic translation initiation factor 5A | OsJ_24987 | up |
| 13 | Q0J2L4 | 26S proteasome non-ATPase regulatory subunit 2 homolog | Os09g0326800 | up |
| 14 | Q5NBJ3 | DNA gyrase subunit B, chloroplastic/mitochondrial | GYRB | up |
| 15 | Q0IRR5 | Os11g0603200 protein | Os11g0603200 | up |
| 16 | Q0IM13 | protein-synthesizing GTPase | Os12g0607100 | up |
| 17 | C6YXK3 | Fructose-bisphosphate aldolase | LOC_Os11g07020 | up |
| 18 | B9F6J9 | CCT-theta | Os03g0804800 | up |
| 19 | A3C107 | Drought-induced S-like ribonuclease | Os09g0537700 | up |
| 20 | Q7XTJ3 | Pyruvate dehydrogenase E1 component subunit alpha-3, chloroplastic | Os04g0119400 | up |
| 21 | A3A338 | TOG domain-containing protein | OsJ_05362 | up |
| 22 | Q0DFB6 | glycine--tRNA ligase | Os06g0103600 | up |
| 23 | Q2QVB4 | Aspartyl aminopeptidase, putative, expressed | LOC_Os12g13390 | up |
| 24 | A3AW25 | Uncharacterized protein | OsJ_15655 | up |
| 25 | Q2QNF7 | Diaminopimelate epimerase, chloroplastic | DAPF | up |
| 26 | Q5W6Z6 | Os05g0247100 protein | Os05g0247100 | up |
| 27 | Q75IM9 | Isovaleryl-CoA dehydrogenase, mitochondrial | Os05g0125500 | up |
| 28 | Q6H4V1 | 4-hydroxyphenylpyruvate dioxygenase | OSJNBa0085K21.52 | up |
| 29 | B9FJD7 | uracil phosphoribosyltransferase | OsJ_18781 | up |
| 30 | A0A0P0UYL6 | Os01g0143800 protein | Os01g0143800 | up |
| 31 | Q7XZF7 | Probable DNA gyrase subunit A, chloroplastic/mitochondrial | GYRA | up |
| 32 | Q5Z790 | Os06g0531200 protein | Os06g0531200 | up |
| 33 | Q53L28 | Acetolactate synthase small subunit, putative, expressed | LOC_Os11g14950 | up |
| 34 | Q75IZ9 | Glucose-6-phosphate 1-dehydrogenase | LOC_Os03g29950 | up |
| 35 | A0A0P0VKA8 | Os02g0554100 protein | Os02g0554100 | up |
| 36 | B9G1B5 | Glutamate decarboxylase | OsJ_27610 | up |
| 37 | A0A5S6RBX8 | Eukaryotic translation initiation factor 3 subunit D | Os05g0566500 | up |
| 38 | Q6Z6Y1 | glutaminase | Os02g0130100 | up |
| 39 | Q5JJI4 | Probable mitochondrial import receptor subunit TOM20 | TOM20 | up |
| 40 | A0A0P0WWY2 | 1,4-alpha-glucan branching enzyme | Os06g0367100 | up |
| 41 | Q0IZS0 | Nicotinate-nucleotide pyrophosphorylase [carboxylating], chloroplastic | Os09g0553600 | up |
| 42 | Q9LDN2 | Uridine 5-monophosphate synthase | UMPS1 | up |
| 43 | A0A0P0XN09 | phosphoribosylaminoimidazolesuccinocarboxamide synthase | Os09g0467100 | up |
| 44 | Q9LRE9 | Cytosolic aldehyde dehydrogenase | ALDH1a | up |
| 45 | Q10S55 | ATP phosphoribosyltransferase, chloroplastic | Os03g0134300 | up |
| 46 | Q6EPN6 | 2-C-methyl-D-erythritol 2,4-cyclodiphosphate synthase, chloroplastic | ISPF | up |
| 47 | Q0D557 | Os07g0580900 protein | Os07g0580900 | up |
| 48 | Q5JJM6 | Os01g0367100 protein | B1642C07.56 | up |
| 49 | A0A0P0XR10 | Os10g0113100 protein | Os10g0113100 | up |
| 50 | Q5JNJ1 | Trehalose 6-phosphate phosphatase | Os01g0749400 | up |
| 51 | A0A0N7KEK9 | methenyltetrahydrofolate cyclohydrolase | Os02g0120900 | up |
| 52 | Q0IM82 | adenine phosphoribosyltransferase | Os12g0589100 | up |
| 53 | Q6ZAA5 | D-3-phosphoglycerate dehydrogenase | Os08g0447000 | up |
| 54 | Q9XHY5 | Os01g0246400 protein | Os01g0246400 | up |
| 55 | A0A0P0WG50 | Os04g0665500 protein | Os04g0665500 | up |
| 56 | Q10SD6 | Anthranilate phosphoribosyltransferase, chloroplast, putative, expressed | LOC_Os03g03450 | up |
| 57 | Q657X6 | Protein EXECUTER 2, chloroplastic | EX2 | up |
| 58 | A3AAD9 | 26S proteosome regulatory subunit-like | Os02g0697600 | up |
| 59 | A0A0P0W629 | Os03g0850600 protein | Os03g0850600 | up |
| 60 | A0A0P0VC29 | Os01g0919700 protein | Os01g0919700 | up |
| 61 | Q0JLB5 | Os01g0611000 protein (Fragment) | Os01g0611000 | up |
| 62 | A0A0P0WV17 | Os06g0237502 protein | Os06g0237502 | up |
| 63 | Q5VND2 | Os06g0149900 protein | Os06g0149900 | up |
| 64 | A3AAM6 | ArsA_ATPase domain-containing protein | OsJ_08118 | up |
| 65 | A0A0P0WDN1 | Superoxide dismutase copper chaperone | Os04g0573200 | up |
| 66 | Q69LD2 | Os07g0170100 protein | Os07g0170100 | up |
| 67 | B9FYF5 | RRM domain-containing protein | OsJ_28175 | up |
| 68 | A0A0N7KFS8 | Chaperone protein dnaJ-like | Os02g0651300 | up |
| 69 | Q6ET01 | Putative aminopropyl transferase | P0463G12.43-1 | up |
| 70 | Q67VF5 | Os06g0238300 protein | Os06g0238300 | up |
| 71 | B9EV54 | Cysteine proteinase inhibitor | OsJ_01243 | up |
| 72 | P31673 | 17.4 kDa class I heat shock protein | HSP17.4 | up |
| 73 | Q10S62 | Uncharacterized protein | OJ1006F06.20 | up |
| 74 | Q6Z0D0 | Os08g0323000 protein | Os08g0323000 | up |
| 75 | A0A0P0Y0G8 | Os11g0183900 protein | Os11g0183900 | up |
| 76 | Q0JEQ2 | Probable L-ascorbate peroxidase 3, peroxisomal | APX3 | up |
| 77 | Q6Z8U4 | Os08g0492100 protein | Os08g0492100 | up |
| 78 | Q7XZG6 | Os03g0811700 protein | LOC_Os03g59710 | up |
| 79 | A3BMP5 | 10 kDa chaperonin | P0524G08.116 | up |
| 80 | A0A8J8XMH3 | Os12g0609500 protein | Os12g0609500 | up |
| 81 | A0A0P0VTN1 | Os03g0161200 protein | Os03g0161200 | up |
| 82 | Q0JHZ2 | Zinc finger CCCH domain-containing protein 11 | Os01g0834700 | up |
| 83 | Q6EPR4 | GrpE protein homolog | Os09g0284400 | up |
| 84 | B9F1F2 | Os02g0654100 protein | Os02g0654100 | up |
| 85 | Q8H2U6 | Os07g0661700 protein | Os07g0661700 | up |
| 86 | Q69SH3 | Os09g0533300 protein | Os09g0533300 | up |
| 87 | A3C6J4 | UBX domain-containing protein | OsJ_32183 | up |
| 88 | A3BDG7 | Calcium-binding EF hand-like protein | Os06g0609600 | up |
| 89 | Q7XHS7 | Os07g0249600 protein | Os07g0249600 | up |
| 90 | Q5ZCL8 | Chloroplastic group IIB intron splicing facilitator CRS2, chloroplastic | Os01g0132800 | up |
| 91 | Q67UK2 | Defective in cullin neddylation protein | Os06g0233400 | up |
| 92 | Q7XI54 | Os07g0632700 protein | Os07g0632700 | up |
| 93 | Q0JJ35 | Mitochondrial import inner membrane translocase subunit TIM50 | Os01g0762600 | up |
| 94 | A2ZVM0 | Protein kinase domain-containing protein | OsJ_02684 | up |
| 95 | Q0DTG6 | Os03g0249200 protein | Os03g0249200 | up |
| 96 | B9EXB9 | Pre-mRNA-processing protein 40A | OsJ_02081 | up |
| 97 | B9G1U9 | J domain-containing protein | OsJ_27983 | up |
| 98 | A0A0N7KI61 | Os03g0785200 protein | Os03g0785200 | up |
| 99 | B9FU89 | Os07g0618600 protein | Os07g0618600 | up |
| 100 | Q2R3H7 | Acyl carrier protein | LOC_Os11g31900 | up |
| 101 | A3C4F7 | Uncharacterized protein | OsJ_31413 | up |
| 102 | B9FC09 | C2H2-type domain-containing protein | OsJ_15807 | up |
| 103 | Q10RP2 | GCN5-related N-acetyltransferase, putative, expressed | LOC_Os03g05710 | up |
| 104 | B9FPG6 | CNNM transmembrane domain-containing protein | OsJ_18448 | up |
| 105 | Q8LHL4 | Os07g0623000 protein | Os07g0623000 | up |
| 106 | Q657X8 | Peroxisomal membrane protein PEX14 | P0005A05.25-1 | up |
| 107 | A0A0P0V6X6 | Os01g0692600 protein | Os01g0692600 | up |
| 108 | B9FU26 | Uncharacterized protein | OsJ_21920 | up |
| 109 | A0A0P0XHI8 | Geranylgeranyl transferase type-2 subunit beta | Os08g0512300 | up |
| 110 | B9EZ25 | Uncharacterized protein | OsJ_03189 | up |
| 111 | Q0JNS7 | Os01g0267800 protein | Os01g0267800 | up |
| 112 | B9F715 | Os03g0823400 protein | OSJNBb0081B07.13 | up |
| 113 | B9G419 | Os09g0465800 protein | Os09g0465800 | up |
| 1 | Q6ZD89 | Flavone 3-O-methyltransferase 1 | COMT | down |
| 2 | B9FMM1 | Uncharacterized protein | OsJ_17229 | down |
| 3 | A0A8J8Y8Q7 | Os03g0284400 protein | Os03g0284400 | down |
| 4 | A0A7S7YAD2 | 50S ribosomal protein L2, chloroplastic | rpl2 | down |
| 5 | Q10MB2 | 30S ribosomal protein S1, chloroplast, putative, expressed | LOC_Os03g20100 | down |
| 6 | A0A5S6RD74 | Phenylalanine ammonia-lyase | Os04g0518100 | down |
| 7 | Q0E446 | Os02g0137200 protein | Os02g0137200 | down |
| 8 | A0A0N7KDR4 | Os01g0749200 protein | Os01g0749200 | down |
| 9 | A3AI13 | Ribosomal protein L6 alpha-beta domain-containing protein | OsJ_10878 | down |
| 10 | Q850L8 | 50S ribosomal protein L6, putative, expressed | OSJNBa0032E21.12 | down |
| 11 | Q2R6V7 | Glycosyl hydrolase family 3 C terminal domain containing protein, expressed | LOC_Os11g18730 | down |
| 12 | Q8RYZ1 | Os01g0874700 protein | P0648C09.9 | down |
| 13 | Q75HX0 | Actin | Os05g0438800 | down |
| 14 | Q8L472 | Os07g0105600 protein | P0617C02.114 | down |
| 15 | Q84QA8 | Uncharacterized protein OJ1012B02.13 | OJ1012B02.13 | down |
| 16 | P0C443 | 50S ribosomal protein L16, chloroplastic | rpl16 | down |
| 17 | Q0DGR3 | Os05g0516600 protein | Os05g0516600 | down |
| 18 | Q0JP88 | Os01g0235900 protein | Os01g0235900 | down |
| 19 | A2ZWI7 | Os01g0678600 protein | Os01g0678600 | down |
| 20 | A0A0N9E0Z0 | 50S ribosomal protein L22, chloroplastic | rpl22 | down |
| 21 | Q10FT7 | Hsp20/alpha crystallin family protein, expressed | LOC_Os03g45340 | down |
| 22 | A0A8J8XZB2 | Putative carnitine/acylcarnitine translocase | OsJ_32559 | down |
| 23 | Q6Z4C0 | Os07g0510400 protein | Os07g0510400 | down |
| 24 | P12124 | NAD(P)H-quinone oxidoreductase subunit 1, chloroplastic | ndhA | down |
| 25 | B9F0E8 | Late embryogenesis abundant protein LEA-2 subgroup domain-containing protein | OsJ_07042 | down |
| 26 | B9FH86 | UspA domain-containing protein | OsJ_18198 | down |
| 27 | P12139 | 50S ribosomal protein L20, chloroplastic | rpl20 | down |
| 28 | B9FFD0 | Os04g0442200 protein | Os04g0442200 | down |
| 29 | Q6ZBZ8 | Os08g0459300 protein | Os08g0459300 | down |
| 30 | B9FLV6 | RNA polymerase sigma factor | OsJ_19735 | down |
| 31 | B9FPB7 | Uncharacterized protein | OsJ_18363 | down |
| 32 | Q8RVB1 | 50S ribosomal protein L31 | Os01g0633000 | down |
| 33 | Q5Z414 | Os06g0730800 protein | Os06g0730800 | down |
| 34 | Q10L97 | NAD dependent epimerase/dehydratase family protein, expressed | LOC_Os03g23980 | down |
| 35 | Q5KQD5 | Os05g0101400 protein | Os05g0101400 | down |
| 36 | A0A0P0VL01 | Small nuclear ribonucleoprotein Sm D1 | Os02g0586500 | down |
| 37 | Q8RZQ8 | Bidirectional sugar transporter SWEET1a | SWEET1A | down |
| 38 | Q7XUY5 | Os04g0465600 protein | Os04g0465600 | down |
| 39 | B9F7Z6 | Os03g0856400 protein | Os03g0856400 | down |
| 40 | Q8H046 | 1,3-beta-glucan synthase | OJ1263H11.9 | down |
| 41 | Q651F3 | Os09g0538800 protein | Os09g0538800 | down |
| 42 | A0A0N7KML5 | Peroxidase | P0547F09.20-1 | down |
| 43 | Q651X6 | Cellulose synthase-like protein E6 | CSLE6 | down |
| 44 | Q94I55 | Probable dolichyl-diphosphooligosaccharide--protein glycosyltransferase subunit 3 | OST3 | down |
| 45 | Q6H454 | Putative hexose transporter | B1040D06.2 | down |
| 46 | Q9AWU6 | Os01g0125800 protein | P0044F08.16 | down |
| 47 | A0A0P0WB26 | OSJNBa0072F16.12 protein | Os04g0461100 | down |
| 48 | B9F1E6 | MSP domain-containing protein | OsJ_07762 | down |
| 49 | P0C457 | 50S ribosomal protein L33, chloroplastic | rpl33 | down |
| 50 | A3A0G8 | Os01g0894500 protein | Os01g0894500 | down |
| 51 | B9F4P8 | 1,3-beta-glucan synthase | OsJ_09005 | down |
| 52 | Q5ZC82 | Cytokinin riboside 5-monophosphate phosphoribohydrolase LOG | LOG | down |
| 53 | Q6ZKB9 | Os08g0430600 protein | Os08g0430600 | down |
| 54 | B9FL15 | Calreticulin | OsJ_19137 | down |
| 55 | Q60E30 | Uncharacterized protein OSJNBb0012L23.7 | OSJNBb0012L23.7 | down |
| 56 | A3A2X0 | Os02g0135800 protein | Os02g0135800 | down |
| 57 | Q0IR61 | Os11g0673200 protein | Os11g0673200 | down |
| 58 | A0A7S8BBD6 | Photosystem II reaction center protein L | psbL | down |
| 59 | B9FFE3 | Signal peptidase complex subunit 2 | Os04g0446300 | down |
| 60 | A3A841 | Os02g0565000 protein | Os02g0565000 | down |
| 61 | B9FH79 | Uncharacterized protein | OsJ_18173 | down |
| 62 | Q5NB36 | Eukaryotic translation initiation factor 3 subunit L | Os01g0229100 | down |
| 63 | Q0DBR2 | Os06g0538200 protein (Fragment) | Os06g0538200 | down |
| 64 | A0A0P0XBZ8 | Os08g0159100 protein | P0498E12.115 | down |
| 65 | Q5Z408 | Uncharacterized protein B1206D04.20 | B1206D04.20 | down |
| 66 | B9F2E2 | RRM domain-containing protein | OsJ_08169 | down |
| 67 | A0A0P0WCB4 | OSJNBa0067K08.9 protein | Os04g0496400 | down |
| 68 | Q8GSC2 | Os07g0628900 protein | Os07g0628900 | down |
| 69 | B9FCX2 | OSJNBa0011F23.3 protein | Os04g0657900 | down |
| 70 | Q5Z8A1 | Os06g0338900 protein | Os06g0338900 | down |
| 71 | Q8RZ80 | Endopeptidase-like protein | B1065G12.15 | down |
| 72 | A0A8J8YE67 | Uncharacterized protein | OsJ_31918 | down |
| 73 | Q6ZGL9 | Signal peptide peptidase 1 | SPP1 | down |
| 74 | Q7XRF0 | OSJNBa0006M15.17 protein | OSJNBa0006M15.17 | down |
| 75 | B9FH99 | Putative beta 1,3-glucanase | OSJNBa0039O18.8 | down |
| 76 | A0A0P0WIM3 | Os05g0170800 protein | Os05g0170800 | down |
| 77 | A0A0N7KDR2 | Os01g0747700 protein | Os01g0747700 | down |
| 78 | A0A0P0V4T6 | Os01g0598600 protein | Os01g0598600 | down |
| 79 | B9FU28 | Uncharacterized protein | OsJ_21922 | down |
| 80 | A0A8J8YIS5 | Os03g0139000 protein | Os03g0139000 | down |
| 81 | A0A0N9E0S3 | Protein PsbN | psbN | down |
| 82 | A3BDU5 | Uncharacterized protein | OsJ_22074 | down |
| 83 | P0CD23 | NAD(P)H-quinone oxidoreductase subunit 2 B, chloroplastic | ndhB2 | down |
| 84 | Q8S3P2 | Uncharacterized protein 24K23.15 | 24K23.15 | down |
| 85 | B9FYD2 | SWI/SNF-like complex subunit BAF250 C-terminal domain-containing protein | OsJ_25084 | down |
| 86 | Q5ZCB0 | Putative Bowman Birk trypsin inhibitor | P0037C04.15 | down |
| 87 | A0A0P0WJN4 | Os05g0241200 protein | Os05g0241200 | down |
| 88 | Q0IW04 | chitinase | OSJNBb0015I11.16 | down |

## Table S3. Differential protein 20 mg L^-1^.vs.Control

| **Table S3. Differential protein 20 mg L^-1^.vs.Control** | | | | |
| --- | --- | --- | --- | --- |
| **No.** | **Protein** | **Description** | **Gene** | **Up/down** |
| 1 | G8CTM8 | Ribulose bisphosphate carboxylase/oxygenase activase, chloroplastic |  | up |
| 2 | Q0INX9 | Os12g0277500 protein | Os12g0277500 | up |
| 3 | Q69QQ6 | Heat shock protein 81-2 | HSP81-2 | up |
| 4 | Q0J4P2 | Heat shock protein 81-1 | HSP81-1 | up |
| 5 | B9F342 | Peptidase M16C associated domain-containing protein | OsJ_08475 | up |
| 6 | B7EA73 | Puromycin-sensitive aminopeptidase | Os08g0562700 | up |
| 7 | B9FGD1 | Elongation factor G, chloroplastic | OsJ_15609 | up |
| 8 | Q0DF58 | Os06g0114000 protein | Os06g0114000 | up |
| 9 | Q10M50 | Magnesium-chelatase subunit ChlH, chloroplastic | CHLH | up |
| 10 | B9G449 | Histidine kinase/HSP90-like ATPase domain-containing protein | OsJ_29732 | up |
| 11 | A0A8J8Y500 | Os03g0151800 protein | Os03g0151800 | up |
| 12 | A0A8J8YN06 | Cell division cycle protein 48 homolog | OsJ_31677 | up |
| 13 | A0A0P0XAE2 | Os07g0691200 protein | Os07g0691200 | up |
| 14 | Q6K8J4 | 4-hydroxy-3-methylbut-2-en-1-yl diphosphate synthase (ferredoxin), chloroplastic | ISPG | up |
| 15 | B9G1I1 | Histidine kinase/HSP90-like ATPase domain-containing protein | OsJ_27736 | up |
| 16 | B9FRD6 | phosphopyruvate hydratase | OsJ_20044 | up |
| 17 | B9FJH8 | Os05g0460000 protein | Os05g0460000 | up |
| 18 | Q8H903 | 60 kDa chaperonin | Os10g0462900 | up |
| 19 | B9FHQ0 | putative 6-phosphofructo-2-kinase | OSJNBa0027N19.4 | up |
| 20 | B9FI18 | NAD-dependent epimerase/dehydratase domain-containing protein | OsJ_18396 | up |
| 21 | Q943K7 | 70 kDa heat shock protein | Os01g0840100 | up |
| 22 | Q10MJ1 | Probable glutamyl endopeptidase, chloroplastic | GEP | up |
| 23 | B9FB06 | Os03g0143400 protein | Os03g0143400 | up |
| 24 | Q75GT3 | Chaperone protein ClpB2, chloroplastic | CLPB2 | up |
| 25 | Q5N8R3 | phosphoribosylformylglycinamidine synthase | B1099D03.28 | up |
| 26 | Q0E0Z3 | Acetolactate synthase | Os02g0510200 | up |
| 27 | Q65XK0 | Ketol-acid reductoisomerase, chloroplastic | Os05g0573700 | up |
| 28 | Q657P0 | Protein translocase subunit SecA | P0426D06.18 | up |
| 29 | Q0DM51 | DEAD-box ATP-dependent RNA helicase 3, chloroplastic | Os03g0827700 | up |
| 30 | B9GCW1 | Ribulose bisphosphate carboxylase large subunit C-terminal domain-containing protein | OsJ_35903 | up |
| 31 | Q5N725 | Fructose-bisphosphate aldolase 3, cytoplasmic | FBA3 | up |
| 32 | P17784 | Fructose-bisphosphate aldolase 1, cytoplasmic | FBA1 | up |
| 33 | A3C4S4 | GDP-mannose 3,5-epimerase 1 | GME-1 | up |
| 34 | A3B9A1 | Putative chaperonin 21 | P0528E04.36-1 | up |
| 35 | Q2QXR8 | Pyruvate kinase 2, cytosolic | Os12g0145700 | up |
| 36 | Q0JM17 | DEAD-box ATP-dependent RNA helicase 56 | AIP1 | up |
| 37 | Q7FAH2 | Glyceraldehyde-3-phosphate dehydrogenase 2, cytosolic | GAPC2 | up |
| 38 | B9FVM5 | valine--tRNA ligase | OsJ_23192 | up |
| 39 | Q0J908 | Pyruvate kinase | Os04g0677500 | up |
| 40 | Q6YZE2 | Glutamate-1-semialdehyde 2,1-aminomutase, chloroplastic | GSA | up |
| 41 | Q10LR9 | Uroporphyrinogen decarboxylase 2, chloroplastic | Os03g0337600 | up |
| 42 | Q337Y2 | Probable cinnamyl alcohol dehydrogenase 3 | CAD3 | up |
| 43 | A0A0P0X3K0 | methylmalonate-semialdehyde dehydrogenase (CoA acylating) | Os07g0188800 | up |
| 44 | Q2QS11 | proline--tRNA ligase | LOC_Os12g25710 | up |
| 45 | Q6Z6L4 | Aminopeptidase M1-A | Os02g0218200 | up |
| 46 | Q8S5T1 | Glutathione reductase | LOC_Os03g06740 | up |
| 47 | Q8W250 | 1-deoxy-D-xylulose 5-phosphate reductoisomerase, chloroplastic | DXR | up |
| 48 | Q6ER90 | threonine--tRNA ligase | Os02g0538000 | up |
| 49 | A2ZW27 | Ketol-acid reductoisomerase | OsJ_02845 | up |
| 50 | Q0J432 | glycine--tRNA ligase | Os08g0538000 | up |
| 51 | Q7F1F2 | peptidylprolyl isomerase | Os08g0525600 | up |
| 52 | Q6YV23 | Carbamoyl-phosphate synthase small chain, chloroplastic | CARA | up |
| 53 | A3ANX5 | Mitochondrial Rho GTPase | OsJ_13062 | up |
| 54 | Q10MQ2 | Probable LL-diaminopimelate aminotransferase, chloroplastic | AGD2 | up |
| 55 | Q0INR5 | 40S ribosomal protein S3a | LOC_Os12g21798 | up |
| 56 | Q2QTC2 | Phosphoglucan, water dikinase, chloroplastic | GWD3 | up |
| 57 | Q0DWH1 | Alcohol dehydrogenase class-3 | Os02g0815500 | up |
| 58 | Q2QQM3 | ubiquitinyl hydrolase 1 | LOC_Os12g30540 | up |
| 59 | Q6ZG77 | Probable diaminopimelate decarboxylase, chloroplastic | LYSA | up |
| 60 | A0A0P0VQ36 | Os02g0774300 protein | Os02g0774300 | up |
| 61 | Q6Z2Z3 | 26S proteasome non-ATPase regulatory subunit 2 homolog | Os02g0146700 | up |
| 62 | B9FQ68 | isoleucine--tRNA ligase | OsJ_22145 | up |
| 63 | A0A0P0YAS2 | Os12g0514500 protein | Os12g0514500 | up |
| 64 | Q0J4W9 | Phospho-2-dehydro-3-deoxyheptonate aldolase | Os08g0484500 | up |
| 65 | A0A8J8XQK2 | ornithine carbamoyltransferase | OsJ_08078 | up |
| 66 | Q652L6 | Monodehydroascorbate reductase 3, cytosolic | MDAR3 | up |
| 67 | A3ANA0 | Tubulin beta chain | OsJ_12810 | up |
| 68 | Q7XXS4 | Thiamine thiazole synthase, chloroplastic | THI1 | up |
| 69 | Q6ATS0 | Magnesium-chelatase subunit ChlD, chloroplastic | CHLD | up |
| 70 | Q40665 | Tubulin beta-3 chain | TUBB3 | up |
| 71 | Q8LJJ2 | threonine synthase | Os01g0693800 | up |
| 72 | Q65XA0 | Probable glutathione S-transferase DHAR1, cytosolic | DHAR1 | up |
| 73 | Q10R17 | Adenylosuccinate synthetase 1, chloroplastic | PURA1 | up |
| 74 | Q6L5I5 | Mitochondrial outer membrane protein porin 2 | VDAC2 | up |
| 75 | Q9AV81 | Pre-mRNA-processing factor 19 | PRP19 | up |
| 76 | Q0DJB7 | Guanosine nucleotide diphosphate dissociation inhibitor | Os05g0304400 | up |
| 77 | Q5NAI9 | Os01g0710000 protein | Os01g0710000 | up |
| 78 | Q0E4R7 | Os02g0103700 protein | Os02g0103700 | up |
| 79 | Q0DKH7 | arginine--tRNA ligase | Os05g0163000 | up |
| 80 | Q5NBJ3 | DNA gyrase subunit B, chloroplastic/mitochondrial | GYRB | up |
| 81 | Q0IRR5 | Os11g0603200 protein | Os11g0603200 | up |
| 82 | B9G059 | threonine--tRNA ligase | OsJ_26793 | up |
| 83 | Q6K5G8 | Glyceraldehyde-3-phosphate dehydrogenase 3, cytosolic | GAPC3 | up |
| 84 | Q8LHD1 | Os01g0966000 protein | Os01g0966000 | up |
| 85 | Q0IM13 | protein-synthesizing GTPase | Os12g0607100 | up |
| 86 | B9GC51 | protein-synthesizing GTPase | OsJ_35408 | up |
| 87 | Q948T6 | Lactoylglutathione lyase | GLYI-11 | up |
| 88 | B9FB88 | TOG domain-containing protein | OsJ_12383 | up |
| 89 | Q7Y0F2 | Probable nucleoredoxin 1-2 | Os03g0405900 | up |
| 90 | A0A8J8YE53 | CCT-beta | OsJ_11750 | up |
| 91 | B9F6J9 | CCT-theta | Os03g0804800 | up |
| 92 | A3C107 | Drought-induced S-like ribonuclease | Os09g0537700 | up |
| 93 | Q7XTJ3 | Pyruvate dehydrogenase E1 component subunit alpha-3, chloroplastic | Os04g0119400 | up |
| 94 | B9FU86 | alanine transaminase | OsJ_25140 | up |
| 95 | B9F279 | 3-isopropylmalate dehydratase | Os02g0125100 | up |
| 96 | Q8W426 | 21D7 | Os21D7 | up |
| 97 | Q0JJS8 | Fe-S cluster assembly factor HCF101, chloroplastic | HCF101 | up |
| 98 | Q6Z4K6 | DEAD-box ATP-dependent RNA helicase 52B | PL10B | up |
| 99 | Q0DSE5 | Adenylosuccinate lyase | Os03g0313600 | up |
| 100 | Q2R2Z0 | Glutamyl-tRNA(Gln) amidotransferase subunit B, chloroplastic/mitochondrial | GATB | up |
| 101 | P52428 | Proteasome subunit alpha type-1 | PAF1 | up |
| 102 | Q0J0N6 | leucine--tRNA ligase | Os09g0503400 | up |
| 103 | A3BVR4 | dihydroxy-acid dehydratase | Os08g0559600 | up |
| 104 | A0A0P0WFN9 | D-3-phosphoglycerate dehydrogenase | Os04g0650800 | up |
| 105 | Q0DFB6 | glycine--tRNA ligase | Os06g0103600 | up |
| 106 | Q9XEA8 | Cysteine synthase | RCS3 | up |
| 107 | Q75G91 | 40S ribosomal protein S3, putative, expressed | LOC_Os03g38000 | up |
| 108 | Q0J136 | Glucose-6-phosphate isomerase | Os09g0465600 | up |
| 109 | Q7X5X9 | Os04g0444600 protein | Os04g0444600 | up |
| 110 | Q6Z1J6 | Obg-like ATPase 1 | YCHF1 | up |
| 111 | A0A8J8YE90 | 4a-hydroxytetrahydrobiopterin dehydratase | Os03g0100200 | up |
| 112 | A3BNM4 | Lon protease homolog, mitochondrial | OsJ_25659 | up |
| 113 | A3BYB3 | leucine--tRNA ligase | Os09g0378300 | up |
| 114 | Q338N8 | alanine transaminase | LOC_Os10g25130 | up |
| 115 | A0A5S6R775 | ATP-dependent Clp protease proteolytic subunit | Os03g0308100 | up |
| 116 | Q7XJW1 | indole-3-glycerol-phosphate synthase | OSJNBa0016O02.9 | up |
| 117 | Q33AF5 | V-type proton ATPase subunit a | LOC_Os10g10500 | up |
| 118 | A0A0P0V3R1 | Os01g0542000 protein | Os01g0542000 | up |
| 119 | Q2QVB4 | Aspartyl aminopeptidase, putative, expressed | LOC_Os12g13390 | up |
| 120 | B9FXC6 | Malic enzyme | OJ1457_D07.117 | up |
| 121 | Q688Q9 | Glutamate--cysteine ligase A, chloroplastic | GSH1-1 | up |
| 122 | A0A0P0WCM6 | Isocitrate dehydrogenase [NADP] | Os04g0508200 | up |
| 123 | A3C117 | Os09g0539100 protein | Os09g0539100 | up |
| 124 | Q94CN9 | Dihydrolipoyl dehydrogenase | Os01g0337900 | up |
| 125 | A3BGL5 | Uncharacterized protein | OsJ_23104 | up |
| 126 | Q65XA1 | Os05g0116000 protein | Os05g0116000 | up |
| 127 | Q5Z5T3 | L-ascorbate oxidase | Os06g0567900 | up |
| 128 | Q10DU3 | 3,4-dihydroxy-2-butanone kinase, putative, expressed | LOC_Os03g51000 | up |
| 129 | A2ZYI3 | NAD(P)H dehydrogenase (quinone) | Os01g0784800 | up |
| 130 | Q5NAY4 | Histidinol dehydrogenase, chloroplastic | HDH | up |
| 131 | Q0DGP6 | Vesicle-fusing ATPase | NSF | up |
| 132 | Q0DLL5 | Os03g0858100 protein | Os03g0858100 | up |
| 133 | Q2QTQ1 | cysteine desulfurase | LOC_Os12g18900 | up |
| 134 | A2ZTZ7 | Os01g0531500 protein | Os01g0531500 | up |
| 135 | A0A0P0XDU4 | Putative aminoimidazolecarboximide ribonucleotide transformylase | OJ1119_C05.21-1 | up |
| 136 | Q67J09 | Os09g0460400 protein | Os09g0460400 | up |
| 137 | Q7XN11 | Gamma-aminobutyrate transaminase 1, mitochondrial | OSL2 | up |
| 138 | Q10LR5 | fumarate hydratase | LOC_Os03g21950 | up |
| 139 | Q2QNF7 | Diaminopimelate epimerase, chloroplastic | DAPF | up |
| 140 | Q10NY1 | Chorismate synthase | LOC_Os03g14990 | up |
| 141 | B9G4J9 | Proteasome subunit beta | OsJ_30005 | up |
| 142 | Q5W6Z6 | Os05g0247100 protein | Os05g0247100 | up |
| 143 | Q6Z3X5 | oxoglutarate dehydrogenase (succinyl-transferring) | Os07g0695800 | up |
| 144 | B9FD44 | Amidase domain-containing protein | OsJ_13590 | up |
| 145 | B9F0F0 | Myb-like domain-containing protein | OsJ_07047 | up |
| 146 | Q75IM9 | Isovaleryl-CoA dehydrogenase, mitochondrial | Os05g0125500 | up |
| 147 | Q6H4V1 | 4-hydroxyphenylpyruvate dioxygenase | OSJNBa0085K21.52 | up |
| 148 | Q10MW3 | Pyruvate decarboxylase 2 | PDC2 | up |
| 149 | Q6AV34 | Probable N-acetyl-gamma-glutamyl-phosphate reductase, chloroplastic | Os03g0617900 | up |
| 150 | B9FJD7 | uracil phosphoribosyltransferase | OsJ_18781 | up |
| 151 | Q2QX01 | Monothiol glutaredoxin-S12, chloroplastic | GRXS12 | up |
| 152 | A3B934 | Carboxypeptidase | Os06g0186400 | up |
| 153 | Q10BT5 | Serine/threonine-protein phosphatase PP2A-2 catalytic subunit | PP2A2 | up |
| 154 | A0A0P0UYL6 | Os01g0143800 protein | Os01g0143800 | up |
| 155 | Q84YK7 | Beta-glucosidase 27 | BGLU27 | up |
| 156 | A0A0P0WC35 | Os04g0504600 protein | Os04g0504600 | up |
| 157 | Q6Z329 | Os02g0728100 protein | Os02g0728100 | up |
| 158 | Q9SDD6 | Peroxiredoxin-2F, mitochondrial | PRXIIF | up |
| 159 | A0A0P0XM43 | Putative ribulose-1,5 bisphosphate carboxylase/oxygenase large subunit N-methyltransferase, chloroplast | OJ1294_G06.18 | up |
| 160 | A0A0N7KFQ1 | Lysine--tRNA ligase | Os02g0623500 | up |
| 161 | B9F3P2 | Clp R domain-containing protein | OsJ_05634 | up |
| 162 | Q0JFQ1 | Os01g0967900 protein | Os01g0967900 | up |
| 163 | A3AUK5 | Prohibitin | OsJ_15076 | up |
| 164 | Q7XZF7 | Probable DNA gyrase subunit A, chloroplastic/mitochondrial | GYRA | up |
| 165 | B9EUP7 | 5-nucleotidase domain-containing protein 4 | OsJ_01113 | up |
| 166 | A0A8J8XKV7 | Os03g0214600 protein | Os03g0214600 | up |
| 167 | Q84MQ0 | Uncharacterized protein OSJNBb0036F07.11 | OSJNBb0036F07.11 | up |
| 168 | B9G4B3 | Os09g0491772 protein | Os09g0491772 | up |
| 169 | Q5Z790 | Os06g0531200 protein | Os06g0531200 | up |
| 170 | B9FHI2 | 40S ribosomal protein S4 | OsJ_18278 | up |
| 171 | Q0D935 | Os07g0109500 protein | OJ1567_G09.119 | up |
| 172 | B9G3B3 | glucan endo-1,3-beta-D-glucosidase | OsJ_29181 | up |
| 173 | Q0D867 | Pyruvate kinase | Os07g0181000 | up |
| 174 | Q10RE5 | Acireductone dioxygenase 2 | ARD2 | up |
| 175 | Q9SMB1 | Spermidine synthase 1 | SPDSYN1 | up |
| 176 | Q53L28 | Acetolactate synthase small subunit, putative, expressed | LOC_Os11g14950 | up |
| 177 | A0A0P0Y344 | Os11g0546000 protein | Os11g0546000 | up |
| 178 | Q8S3Q3 | glucose-6-phosphate 1-epimerase | 24K23.3 | up |
| 179 | A0A0P0XC77 | transaldolase | OJ1066_B03.121-1 | up |
| 180 | A3A3M4 | Uncharacterized protein | OsJ_05566 | up |
| 181 | Q0JNK5 | Coatomer subunit beta-2 | Os01g0281400 | up |
| 182 | Q10NX8 | Beta-galactosidase 6 | Os03g0255100 | up |
| 183 | B9G1B5 | Glutamate decarboxylase | OsJ_27610 | up |
| 184 | Q0JLS8 | Os01g0574600 protein | Os01g0574600 | up |
| 185 | Q9AXB0 | Uroporphyrinogen decarboxylase 1, chloroplastic | Os01g0622300 | up |
| 186 | A3BVT3 | Os08g0562600 protein | Os08g0562600 | up |
| 187 | A0A0P0X7V0 | Os07g0573800 protein | Os07g0573800 | up |
| 188 | Q0JGY1 | 60S ribosomal protein L5-1 | RPL5A | up |
| 189 | A0A8J8Y8S8 | glutamine--tRNA ligase | Os01g0185200 | up |
| 190 | Q7XM29 | Molybdopterin biosynthesis protein CNX1 | OSJNBa0015K02.22 | up |
| 191 | B9F8W6 | Uncharacterized protein | OsJ_11170 | up |
| 192 | Q10G39 | Pyruvate dehydrogenase E1 component subunit beta-4, chloroplastic | Os03g0645100 | up |
| 193 | Q75LB3 | 4-coumarate--CoA ligase | LOC_Os03g62850 | up |
| 194 | Q69UI2 | 40S ribosomal protein S13-1 | Os08g0117200 | up |
| 195 | Q10BB7 | Putative chloroplast RNA processing protein | OSJNBb0081B07.6 | up |
| 196 | A0A8J8YAG9 | Prohibitin | Os03g0841700 | up |
| 197 | Q0DC89 | Peptide methionine sulfoxide reductase B1, chloroplastic | MSRB1 | up |
| 198 | Q75GB3 | ER membrane protein complex subunit 1 | Os05g0230600 | up |
| 199 | A0A0P0W5L4 | Os03g0843300 protein | OSJNBa0032G11.5 | up |
| 200 | A0A5S6RBX8 | Eukaryotic translation initiation factor 3 subunit D | Os05g0566500 | up |
| 201 | B9G0C7 | Eukaryotic translation initiation factor 3 subunit D | OsJ_26917 | up |
| 202 | Q0DXH5 | Annexin | Os02g0753800 | up |
| 203 | Q5JJI4 | Probable mitochondrial import receptor subunit TOM20 | TOM20 | up |
| 204 | A0A0P0WWY2 | 1,4-alpha-glucan branching enzyme | Os06g0367100 | up |
| 205 | Q6Z0I4 | Enoyl-[acyl-carrier-protein] reductase [NADH] 1, chloroplastic | Os08g0327400 | up |
| 206 | Q69J84 | Os07g0675000 protein | P0037D09.22-1 | up |
| 207 | B9F1V9 | Uncharacterized protein | OsJ_07974 | up |
| 208 | A0A8J8Y114 | Probable 6-phosphogluconolactonase | OsJ_30091 | up |
| 209 | B9FD11 | OSJNBb0004G23.6 protein | Os04g0105700 | up |
| 210 | P0C587 | Glutamyl-tRNA reductase, chloroplastic | Os10g0502400 | up |
| 211 | B9GDT7 | ATP citrate synthase | OsJ_36536 | up |
| 212 | Q6K623 | Os02g0612900 protein | Os02g0612900 | up |
| 213 | Q10LJ0 | Nuclear cap-binding protein subunit 1 | ABH1 | up |
| 214 | A3A253 | HECT-type E3 ubiquitin transferase | OsJ_04994 | up |
| 215 | B9GC70 | Dehydrogenase E1 component domain-containing protein | OsJ_35450 | up |
| 216 | Q0DDM2 | Os06g0215300 protein | Os06g0215300 | up |
| 217 | Q0IZS0 | Nicotinate-nucleotide pyrophosphorylase [carboxylating], chloroplastic | Os09g0553600 | up |
| 218 | Q53JF7 | Abscisic stress-ripening protein 5 | ASR5 | up |
| 219 | Q9SDG6 | 60S ribosomal protein L30 | RPL30 | up |
| 220 | A3AXT8 | Uncharacterized protein | OsJ_16326 | up |
| 221 | B9FDF9 | Protein transport protein Sec24-like | OsJ_13628 | up |
| 222 | A0A0P0W5P7 | Os03g0832600 protein | Os03g0832600 | up |
| 223 | Q9LDN2 | Uridine 5-monophosphate synthase | UMPS1 | up |
| 224 | Q5VND6 | Nucleosome assembly protein 11 | NAP1;1 | up |
| 225 | Q6ZDG3 | Os08g0475400 protein | Os08g0475400 | up |
| 226 | Q7XTL6 | inorganic diphosphatase | Os04g0687100 | up |
| 227 | Q2R4J4 | Zinc finger CCCH domain-containing protein 63 | Os11g0472000 | up |
| 228 | O22567 | 1-deoxy-D-xylulose-5-phosphate synthase 1, chloroplastic | CLA1 | up |
| 229 | Q9LST9 | Proteasome subunit beta | OsPBA1 | up |
| 230 | A0A0P0XN09 | phosphoribosylaminoimidazolesuccinocarboxamide synthase | Os09g0467100 | up |
| 231 | P49030 | Protein mago nashi homolog 2 | MAGO2 | up |
| 232 | Q655Y9 | fructose-bisphosphatase | Os06g0664200 | up |
| 233 | A0A0P0VAG8 | Os01g0851000 protein | Os01g0851000 | up |
| 234 | B7FAH8 | Os12g0541000 protein | Os12g0541000 | up |
| 235 | A0A0P0XGW9 | GTP cyclohydrolase II | Os08g0481950 | up |
| 236 | A0A0P0VAD1 | Os01g0850900 protein | Os01g0850900 | up |
| 237 | Q33E23 | Glutamate dehydrogenase 2, mitochondrial | GDH2 | up |
| 238 | A0A0N7KR67 | Os09g0538000 protein | P0229B10.19-2 | up |
| 239 | A0A0N7KJ23 | OSJNBb0088C09.10 protein | Os04g0423400 | up |
| 240 | Q10M57 | 14 kDa zinc-binding protein, putative, expressed | LOC_Os03g20630 | up |
| 241 | Q7XM43 | 4-hydroxy-tetrahydrodipicolinate synthase | OSJNBb0018J12.16 | up |
| 242 | Q9ZWR5 | Importin-beta1 | Os05g0353400 | up |
| 243 | Q9LRE9 | Cytosolic aldehyde dehydrogenase | ALDH1a | up |
| 244 | Q10S55 | ATP phosphoribosyltransferase, chloroplastic | Os03g0134300 | up |
| 245 | B9FZ31 | tyrosine--tRNA ligase | P0443G08.126 | up |
| 246 | B9FY56 | SUMO-activating enzyme subunit | OsJ_24922 | up |
| 247 | A0A0P0VTX8 | Os03g0182600 protein | Os03g0182600 | up |
| 248 | Q0DYK2 | Os02g0686600 protein | Os02g0686600 | up |
| 249 | K4FDV3 | Sucrose synthase |  | up |
| 250 | Q6EPN6 | 2-C-methyl-D-erythritol 2,4-cyclodiphosphate synthase, chloroplastic | ISPF | up |
| 251 | A2ZTY4 | Os01g0528800 protein | Os01g0528800 | up |
| 252 | Q0D557 | Os07g0580900 protein | Os07g0580900 | up |
| 253 | Q5JJM6 | Os01g0367100 protein | B1642C07.56 | up |
| 254 | A3BAG6 | Ubiquitin receptor RAD23 | Os06g0264300 | up |
| 255 | B9EXN4 | Protein ROOT HAIR DEFECTIVE 3 homolog | OsJ_02311 | up |
| 256 | A3BMP1 | Xylulose kinase | OJ1340_C08.135 | up |
| 257 | Q6ZA87 | Malate dehydrogenase | OsJ_27438 | up |
| 258 | A3BFX9 | Eukaryotic translation initiation factor 5A | OJ1118_G09.115 | up |
| 259 | Q10SX9 | Protein tyrosine phosphatase, putative, expressed | LOC_Os03g01750 | up |
| 260 | A0A0P0WYX1 | tryptophan synthase | P0505A04.10-2 | up |
| 261 | Q6YXT5 | L-gulonolactone oxidase | Os08g0114300 | up |
| 262 | Q0DGC8 | Os05g0540300 protein | Os05g0540300 | up |
| 263 | A0A0P0XPK4 | Os09g0509000 protein | Os09g0509000 | up |
| 264 | B9FRK1 | Mitogen-activated protein kinase | OsJ_20171 | up |
| 265 | P48489 | Serine/threonine-protein phosphatase PP1 | Os03g0268000 | up |
| 266 | A0A8J8XRD2 | Glycosyl hydrolase family 95 N-terminal domain-containing protein | OsJ_31136 | up |
| 267 | P55142 | Glutaredoxin-C6 | GRXC6 | up |
| 268 | A0A0P0XR10 | Os10g0113100 protein | Os10g0113100 | up |
| 269 | A0A0P0WPV1 | folate gamma-glutamyl hydrolase | Os05g0517500 | up |
| 270 | Q10G81 | Histone-binding protein MSI1 homolog | MSI1 | up |
| 271 | Q5QLQ5 | Os01g0667200 protein | Os01g0667200 | up |
| 272 | B9G741 | Haloacid dehalogenase-like hydrolase domain-containing protein Sgpp | OsJ_32524 | up |
| 273 | Q0DA15 | NADH:ubiquinone reductase (non-electrogenic) | Os06g0684000 | up |
| 274 | Q0JF58 | Protein argonaute 4B | AGO4B | up |
| 275 | Q67UF5 | Protein disulfide isomerase-like 2-3 | PDIL2-3 | up |
| 276 | D7PPK3 | ADH1 | adh1 | up |
| 277 | Q0JFT4 | Os01g0962600 protein | Os01g0962600 | up |
| 278 | A0A0N7KEK9 | methenyltetrahydrofolate cyclohydrolase | Os02g0120900 | up |
| 279 | A0A8J8YAS4 | Os03g0323800 protein | Os03g0323800 | up |
| 280 | A0A8U0WPK4 | peptidylprolyl isomerase | OsJ_02248 | up |
| 281 | B9EU02 | Peptide-N4-(N-acetyl-beta-glucosaminyl)asparagine amidase A | OsJ_00820 | up |
| 282 | Q0JM76 | Monothiol glutaredoxin-S4, mitochondrial | GRXS4 | up |
| 283 | Q0IWL9 | Monothiol glutaredoxin-S11 | GRXS11 | up |
| 284 | Q7XKV4 | Beta-glucosidase 12 | BGLU12 | up |
| 285 | A3BYC3 | 4-coumarate--CoA ligase | OsJ_29182 | up |
| 286 | Q0D6H5 | Carboxypeptidase | Os07g0479300 | up |
| 287 | Q5W6F1 | Trans-cinnamate 4-monooxygenase | C4HL | up |
| 288 | Q5ZCF2 | Os01g0778800 protein | Os01g0778800 | up |
| 289 | A0A0P0VLS3 | Os02g0622400 protein | Os02g0622400 | up |
| 290 | Q852G0 | ATP-dependent Clp protease proteolytic subunit | LOC_Os03g29810 | up |
| 291 | Q6ZAA5 | D-3-phosphoglycerate dehydrogenase | Os08g0447000 | up |
| 292 | B9FKW6 | Proteasome subunit alpha type | OsJ_19022 | up |
| 293 | B9G4B9 | NAD-dependent epimerase/dehydratase domain-containing protein | OsJ_29857 | up |
| 294 | Q84ZC0 | Probable V-type proton ATPase subunit H | Os07g0549700 | up |
| 295 | B9EW03 | Aspartokinase | OsJ_04629 | up |
| 296 | Q6Z671 | Os02g0720900 protein | Os02g0720900 | up |
| 297 | Q0IMT5 | Os12g0543600 protein | Os12g0543600 | up |
| 298 | Q6ZB58 | Mitochondrial pyruvate carrier | Os08g0344300 | up |
| 299 | A0A8J8XDX3 | UMP kinase | Os01g0965400 | up |
| 300 | Q8W3G2 | Glycerol-3-phosphate acyltransferase, chloroplastic | LOC_Os10g42720 | up |
| 301 | Q0DIQ4 | Os05g0371200 protein | Os05g0371200 | up |
| 302 | A3AAG5 | Methyltransferase type 11 domain-containing protein | OsJ_08055 | up |
| 303 | Q8LR54 | Glycylpeptide N-tetradecanoyltransferase | Os01g0708100 | up |
| 304 | Q6K456 | Os09g0132600 protein | Os09g0132600 | up |
| 305 | Q9XHY5 | Os01g0246400 protein | Os01g0246400 | up |
| 306 | B9FA50 | VOC domain-containing protein | OsJ_11974 | up |
| 307 | B9FQR0 | UDP-sugar pyrophosphorylase | OsJ_22533 | up |
| 308 | A0A0P0WG50 | Os04g0665500 protein | Os04g0665500 | up |
| 309 | A2ZVG7 | ATP-dependent zinc metalloprotease FTSH 9, chloroplastic/mitochondrial | FTSH9 | up |
| 310 | Q10SD6 | Anthranilate phosphoribosyltransferase, chloroplast, putative, expressed | LOC_Os03g03450 | up |
| 311 | B9FLK4 | Branched-chain-amino-acid aminotransferase | Os05g0558400 | up |
| 312 | Q5VSC2 | NAD(+) diphosphatase | Os06g0141166 | up |
| 313 | B9FQU4 | Putative arogenate dehydrogenase isoform 2 | OsJ_22617 | up |
| 314 | Q2QQX3 | Expressed protein | LOC_Os12g29550 | up |
| 315 | A0A0P0WS09 | Os06g0106800 protein | Os06g0106800 | up |
| 316 | Q6ZJ05 | Probable uridine nucleosidase 1 | URH1 | up |
| 317 | Q2QYY2 | Leucine-rich repeat family protein, putative, expressed | LOC_Os12g01200 | up |
| 318 | Q8H936 | COP9 signalosome complex subunit 5 | CSN5 | up |
| 319 | B9FSX0 | Nucleoporin_N domain-containing protein | OsJ_21091 | up |
| 320 | B9EV70 | Os01g0276200 protein | Os01g0276200 | up |
| 321 | A3AAD9 | 26S proteosome regulatory subunit-like | Os02g0697600 | up |
| 322 | A0A0P0W629 | Os03g0850600 protein | Os03g0850600 | up |
| 323 | A0A0P0W2C1 | Tetratricopeptide repeat protein 38 | Os03g0689900 | up |
| 324 | Q0JLB5 | Os01g0611000 protein (Fragment) | Os01g0611000 | up |
| 325 | A0A0P0WV17 | Os06g0237502 protein | Os06g0237502 | up |
| 326 | Q851K1 | Germin-like protein 3-6 | Os03g0694000 | up |
| 327 | Q69U05 | Os06g0623300 protein | Os06g0623300 | up |
| 328 | Q67TV5 | Os09g0266000 protein | Os09g0266000 | up |
| 329 | A0A8J8YHU0 | Os11g0629200 protein | Os11g0629200 | up |
| 330 | P35686 | 40S ribosomal protein S20 | RPS20 | up |
| 331 | A2ZY47 | Chorismate mutase | Os01g0764400 | up |
| 332 | Q851Y7 | Monothiol glutaredoxin-S7, chloroplastic | GRXS7 | up |
| 333 | A0A8J8XXT2 | Os03g0116500 protein | Os03g0116500 | up |
| 334 | A3AD30 | Dienelactone hydrolase domain-containing protein | OsJ_09019 | up |
| 335 | A3AAM6 | ArsA_ATPase domain-containing protein | OsJ_08118 | up |
| 336 | Q9LD61 | Aspartate carbamoyltransferase, chloroplastic | PYRB | up |
| 337 | A3C7X7 | Uncharacterized protein | OsJ_32699 | up |
| 338 | A0A0P0WDN1 | Superoxide dismutase copper chaperone | Os04g0573200 | up |
| 339 | Q8GVF5 | Eukaryotic translation initiation factor 6 | EIF6 | up |
| 340 | A0A0P0XBL6 | Os08g0127600 protein | Os08g0127600 | up |
| 341 | Q0DFD6 | Os05g0597100 protein | Os05g0597100 | up |
| 342 | Q6ZHK4 | Os07g0491900 protein | P0038F10.103 | up |
| 343 | Q7XNX6 | Sucrose synthase 7 | SUS7 | up |
| 344 | B9FUQ4 | Uncharacterized protein | OsJ_25474 | up |
| 345 | A3BFZ0 | Protein kinase domain-containing protein | OsJ_22867 | up |
| 346 | A3AT06 | 3-oxoacyl-[acyl-carrier-protein] reductase | OsJ_14493 | up |
| 347 | B7FA99 | Os02g0302200 protein | Os02g0302200 | up |
| 348 | B9FTK5 | Aminopropyl transferase | Os06g0528600 | up |
| 349 | A0A0P0VWN1 | Os03g0278200 protein | Os03g0278200 | up |
| 350 | Q6K9C1 | Protein TAB2 homolog, chloroplastic | Os02g0610800 | up |
| 351 | Q10MC7 | RNA helicase | LOC_Os03g19960 | up |
| 352 | Q8H3I4 | Peroxisomal (S)-2-hydroxy-acid oxidase GLO4 | GLO4 | up |
| 353 | Q10L56 | Glutathione peroxidase | LOC_Os03g24380 | up |
| 354 | B9FP43 | Os05g0357600 protein | Os05g0357600 | up |
| 355 | Q75LS2 | hydroxyisourate hydrolase | LOC_Os03g27320 | up |
| 356 | Q0DC50 | Immunophilin-like | Os06g0484500 | up |
| 357 | Q9FNU2 | ABC transporter B family member 25 | ABCB25 | up |
| 358 | A2ZPW5 | Os01g0178000 protein | P0509B06.2-1 | up |
| 359 | Q69XJ9 | Os06g0602600 protein | Os06g0602600 | up |
| 360 | A0A8J8XJB3 | Os03g0197400 protein | Os03g0197400 | up |
| 361 | B9F537 | Uncharacterized protein | OsJ_06316 | up |
| 362 | A0A0P0Y329 | Os11g0526200 protein | Os11g0526200 | up |
| 363 | Q6H541 | 40S ribosomal protein S24 | Os02g0229000 | up |
| 364 | Q0JDG5 | Os04g0405100 protein | Os04g0405100 | up |
| 365 | A3AU95 | OSJNBa0064H22.3 protein | Os04g0447500 | up |
| 366 | Q5Z8Y9 | Os06g0564500 protein | Os06g0564500 | up |
| 367 | Q2QUI0 | Glutathione synthetase | LOC_Os12g16200 | up |
| 368 | B9FL32 | Os05g0512200 protein | Os05g0512200 | up |
| 369 | B9FJP6 | Os05g0470800 protein | Os05g0470800 | up |
| 370 | Q2R176 | Os11g0615200 protein | LOC_Os11g40100 | up |
| 371 | A3A851 | Dirigent protein | OsJ_07186 | up |
| 372 | Q656J2 | Reticulon-like protein | Os06g0503400 | up |
| 373 | B9FR10 | Prolyl endopeptidase | OsJ_22744 | up |
| 374 | P50156 | Probable aquaporin TIP1-1 | TIP1-1 | up |
| 375 | Q6ZHS7 | Os02g0187500 protein | Os02g0187500 | up |
| 376 | B9F0B8 | SAM domain-containing protein | OsJ_06971 | up |
| 377 | A0A8J8Y0D4 | V-type proton ATPase subunit a | Os03g0251500 | up |
| 378 | B9FQ59 | Sucrose-phosphate synthase | OsJ_22133 | up |
| 379 | B9EUD2 | Ribonuclease II/R domain-containing protein | OsJ_04037 | up |
| 380 | A0A0N7KFS8 | Chaperone protein dnaJ-like | Os02g0651300 | up |
| 381 | Q0JD42 | Protein disulfide isomerase-like 5-2 | PDIL5-2 | up |
| 382 | Q850L6 | Putative oligopeptide transporter protein | OSJNBa0032E21.14 | up |
| 383 | Q6ET01 | Putative aminopropyl transferase | P0463G12.43-1 | up |
| 384 | A3BT95 | 3-oxoacyl-[acyl-carrier-protein] synthase | OsJ_27364 | up |
| 385 | Q7XVN7 | DnaJ protein ERDJ2 | ERDJ2 | up |
| 386 | Q9FRT3 | Thioredoxin h | RTRXH2 | up |
| 387 | Q0E2F5 | Peroxin-7 | Os02g0245100 | up |
| 388 | Q0DHL4 | ATP-dependent zinc metalloprotease FTSH 8, mitochondrial | FTSH8 | up |
| 389 | A3C6N2 | glutathione transferase | Os10g0525400 | up |
| 390 | Q0DFP8 | Peroxisomal membrane protein PEX14 | Os05g0578000 | up |
| 391 | B9EY90 | Peptide deformylase | OsJ_02752 | up |
| 392 | B9G765 | CS domain-containing protein | OsJ_32585 | up |
| 393 | A0A0P0X335 | Os07g0178800 protein | Os07g0178800 | up |
| 394 | Q0JBF1 | Os04g0535600 protein | Os04g0535600 | up |
| 395 | A0A6B7JG68 | Non-specific lipid-transfer protein |  | up |
| 396 | Q7XHP7 | Os07g0239400 protein | Os07g0239400 | up |
| 397 | Q75M67 | Co-chaperone protein p23 | LOC_Os03g26460 | up |
| 398 | A0A0N7KGD3 | Putative ABC transporter | P0452F04.18-1 | up |
| 399 | Q5VRG1 | Metaxin | OSJNBa0033B09.11 | up |
| 400 | Q0JEA6 | Glycosyltransferase (Fragment) | Os04g0305700 | up |
| 401 | Q6YVH6 | Os02g0705100 protein | Os02g0705100 | up |
| 402 | A3AI17 | Mitochondrial fission 1 protein | OsJ_10883 | up |
| 403 | B9F2K8 | Os02g0735100 protein | Os02g0735100 | up |
| 404 | A0A0P0X6V3 | Putative ATP-dependent proteinase BsgA | P0409B11.11 | up |
| 405 | A0A8J8YB53 | Non-specific lipid-transfer protein | Os12g0115300 | up |
| 406 | Q67VF5 | Os06g0238300 protein | Os06g0238300 | up |
| 407 | Q6EPQ1 | Os02g0678400 protein | Os02g0678400 | up |
| 408 | Q7XTJ6 | Os04g0118900 protein | Os04g0118900 | up |
| 409 | Q2QMK2 | Os12g0601200 protein | LOC_Os12g40880 | up |
| 410 | A3C6Q9 | glutathione transferase | OsJ_32247 | up |
| 411 | B9EV54 | Cysteine proteinase inhibitor | OsJ_01243 | up |
| 412 | P31673 | 17.4 kDa class I heat shock protein | HSP17.4 | up |
| 413 | Q0DAL3 | Methyltransferase-like | Os06g0646000 | up |
| 414 | Q8LR34 | Iron-sulfur cluster assembly protein 1 | ISU1 | up |
| 415 | A0A0P0XCP7 | Os08g0176100 protein | Os08g0176100 | up |
| 416 | Q7XZZ1 | Glutamyl-tRNA(Gln) amidotransferase subunit C, chloroplastic/mitochondrial | GATC | up |
| 417 | Q5SNI3 | Os01g0190000 protein | Os01g0190000 | up |
| 418 | A3A3U3 | Proteasome subunit beta | OsJ_05637 | up |
| 419 | Q7XVK0 | OSJNBa0069D17.1 protein | OSJNBa0069D17.1 | up |
| 420 | A3AYY0 | C2 domain-containing protein | OsJ_16741 | up |
| 421 | Q94GB7 | Uncharacterized protein OSJNBb0016M10.6 | OSJNBb0016M10.6 | up |
| 422 | Q60DX8 | Beta-glucosidase 22 | BGLU22 | up |
| 423 | B9G0Z9 | Uncharacterized protein | OsJ_27374 | up |
| 424 | Q8LR75 | Os01g0841600 protein | Os01g0841600 | up |
| 425 | Q6Z0D0 | Os08g0323000 protein | Os08g0323000 | up |
| 426 | B9FAM9 | Ferredoxin | OsJ_12142 | up |
| 427 | Q7XW88 | Prostaglandin E synthase 2 | Os04g0244400 | up |
| 428 | Q6ZH90 | Histone deacetylase complex subunit SAP18 | Os02g0122000 | up |
| 429 | Q6ZIB4 | Proteasome subunit beta | Os08g0529100 | up |
| 430 | Q6Z0Z1 | Os02g0135600 protein | Os02g0135600 | up |
| 431 | A3BCV5 | RRM domain-containing protein | OsJ_21732 | up |
| 432 | Q7XMG3 | ubiquitinyl hydrolase 1 | OSJNBa0028I23.19 | up |
| 433 | Q7XHC4 | Acyl transferase 15 | AT15 | up |
| 434 | Q6ESK5 | Peptidyl-prolyl cis-trans isomerase | Os09g0411700 | up |
| 435 | Q652V8 | 16.0 kDa heat shock protein, peroxisomal | HSP16.0 | up |
| 436 | Q10S29 | Expressed protein | LOC_Os03g04440 | up |
| 437 | A3BIG2 | GCN5-related N-acetyltransferase (GNAT) family-like protein | B1100H02.2 | up |
| 438 | A3AEW7 | Uroporphyrinogen-III synthase | OsJ_09696 | up |
| 439 | A0A5S6RBF7 | Os10g0542200 protein | OSJNBb0015I11.23 | up |
| 440 | A3ADZ1 | Os03g0141000 protein | Os03g0141000 | up |
| 441 | Q0J9U9 | Os04g0630400 protein | Os04g0630400 | up |
| 442 | Q5Z9Z3 | Thioredoxin-like protein Clot | Os06g0320000 | up |
| 443 | A3BYT1 | AB hydrolase-1 domain-containing protein | OsJ_29351 | up |
| 444 | A0A0P0Y0G8 | Os11g0183900 protein | Os11g0183900 | up |
| 445 | Q6ZKV8 | Bifunctional dethiobiotin synthetase/7,8-diamino-pelargonic acid aminotransferase, mitochondrial | BIO3-BIO1 | up |
| 446 | Q84RV9 | Os07g0595700 protein | Os07g0595700 | up |
| 447 | Q0JEQ2 | Probable L-ascorbate peroxidase 3, peroxisomal | APX3 | up |
| 448 | A0A8J8Y2Z1 | Uncharacterized protein | OsJ_34403 | up |
| 449 | Q84TB6 | Actin-depolymerizing factor 3 | ADF3 | up |
| 450 | Q6YW27 | Os08g0248900 protein | Os08g0248900 | up |
| 451 | Q8GVF6 | Os07g0639600 protein | Os07g0639600 | up |
| 452 | Q9AY76 | Actin-depolymerizing factor 2 | ADF2 | up |
| 453 | A0A8J8YI60 | Transmembrane CLPTM1 family protein | OsJ_14919 | up |
| 454 | A3BFD4 | Os06g0714500 protein | Os06g0714500 | up |
| 455 | Q0IYG0 | Os10g0321700 protein | Os10g0321700 | up |
| 456 | Q6K9C3 | Serine/arginine-rich splicing factor RSZ23 | RSZ23 | up |
| 457 | Q0ITL6 | Os11g0241700 protein | Os11g0241700 | up |
| 458 | Q0J094 | ATPase | Os09g0521500 | up |
| 459 | A3ALI3 | Os03g0685500 protein | Os03g0685500 | up |
| 460 | Q6K9X3 | Nicotinate N-methyltransferase 1 | NANMT1 | up |
| 461 | A3B8D7 | HesB-like domain-containing protein-like | Os06g0146400 | up |
| 462 | Q7EZ93 | Os08g0517300 protein | Os08g0517300 | up |
| 463 | Q0JBQ4 | Os04g0517300 protein | Os04g0517300 | up |
| 464 | Q0JD85 | Fibrillin protein 5 homolog | FBN5 | up |
| 465 | Q7G645 | Os10g0330400 protein | LOC_Os10g18370 | up |
| 466 | A0A5S6R7M2 | OSJNBb0034I13.9 protein | Os04g0636900 | up |
| 467 | Q2QT46 | Os12g0407500 protein | LOC_Os12g21890 | up |
| 468 | A3BMP5 | 10 kDa chaperonin | P0524G08.116 | up |
| 469 | A0A8J8XMH3 | Os12g0609500 protein | Os12g0609500 | up |
| 470 | B9ETE7 | Bicarbonate transporter-like transmembrane domain-containing protein | OsJ_00581 | up |
| 471 | A0A7S8BAT2 | 30S ribosomal protein S11, chloroplastic | rps11 | up |
| 472 | Q5N9P6 | Os01g0695300 protein | Os01g0695300 | up |
| 473 | A3AQB2 | ATP synthase subunit e, mitochondrial | Os04g0117100 | up |
| 474 | A3A0G8 | Os01g0894500 protein | Os01g0894500 | up |
| 475 | A0A0P0VTN1 | Os03g0161200 protein | Os03g0161200 | up |
| 476 | Q7XXR4 | Os08g0243600 protein | Os08g0243600 | up |
| 477 | B9FR42 | Uncharacterized protein | OsJ_19840 | up |
| 478 | Q6EPR4 | GrpE protein homolog | Os09g0284400 | up |
| 479 | A3ADQ4 | FAS1 domain-containing protein | OsJ_09260 | up |
| 480 | Q6F2U7 | Inositol-1-monophosphatase | LOC_Os03g39000 | up |
| 481 | Q5NB99 | Pre-mRNA-splicing factor SPF27 homolog | P0453A06.28 | up |
| 482 | A0A0P0WNJ6 | Os05g0468600 protein | Os05g0468600 | up |
| 483 | Q0DRP7 | Os03g0366000 protein | Os03g0366000 | up |
| 484 | A0A0N7KKA4 | Probable tRNA N6-adenosine threonylcarbamoyltransferase | GCP2 | up |
| 485 | A0A0P0VGU6 | Peroxidase | Os02g0236800 | up |
| 486 | Q10B63 | Probable acylpyruvase FAHD2, mitochondrial | FAHD2 | up |
| 487 | Q0IWJ3 | Os10g0505700 protein | Os10g0505700 | up |
| 488 | Q94GF1 | Anthranilate synthase alpha subunit 1, chloroplastic | ASA1 | up |
| 489 | Q7XTU0 | Os04g0619500 protein | Os04g0619500 | up |
| 490 | Q7XN53 | OSJNBb0103I08.16 protein | OSJNBb0103I08.16 | up |
| 491 | Q7XBX2 | GTPase activating protein, putative, expressed | LOC_Os10g42420 | up |
| 492 | Q0JEF5 | Flowering-promoting factor 1-like protein 4 | Os04g0282400 | up |
| 493 | Q8H2U6 | Os07g0661700 protein | Os07g0661700 | up |
| 494 | Q6YSF3 | Acidic leucine-rich nuclear phosphoprotein 32-related protein 1 | Os07g0607800 | up |
| 495 | B9FWI5 | Os07g0264100 protein | Os07g0264100 | up |
| 496 | B9F338 | Os02g0760500 protein | Os02g0760500 | up |
| 497 | B7EII4 | Os06g0634300 protein | Os06g0634300 | up |
| 498 | C7J3H1 | Os06g0149300 protein | Os06g0149300 | up |
| 499 | Q0DBT1 | Os06g0530300 protein (Fragment) | Os06g0530300 | up |
| 500 | Q0JPJ8 | Os01g0219000 protein | Os01g0219000 | up |
| 501 | A3C602 | Os10g0491000 protein | Os10g0491000 | up |
| 502 | B9FB84 | Uncharacterized protein | OsJ_12376 | up |
| 503 | Q2QNG2 | GDSL-like Lipase/Acylhydrolase family protein, expressed | LOC_Os12g37910 | up |
| 504 | Q69UI8 | Os08g0116500 protein | Os08g0116500 | up |
| 505 | Q6ESG1 | Probable sodium/metabolite cotransporter BASS4, chloroplastic | BASS4 | up |
| 506 | A0A0P0X6F8 | Os07g0485400 protein | Os07g0485400 | up |
| 507 | Q8S1V0 | Os01g0937100 protein | Os01g0937100 | up |
| 508 | B9G9U1 | DNA-directed RNA polymerases I, II, and III subunit RPABC5 | OsJ_33258 | up |
| 509 | B9G087 | Uncharacterized protein | OsJ_26844 | up |
| 510 | Q60F47 | Os05g0121200 protein | Os05g0121200 | up |
| 511 | A3C6J4 | UBX domain-containing protein | OsJ_32183 | up |
| 512 | A0A0P0WNP9 | Non-specific lipid-transfer protein | Os05g0477900 | up |
| 513 | A0A0P0V127 | NEDD8-activating enzyme E1 catalytic subunit | Os01g0271500 | up |
| 514 | A0A0N7KFF9 | Cytochrome b-c1 complex subunit 6 | Os02g0541700 | up |
| 515 | B9F6B9 | Os03g0784400 protein | OSJNBb0093E13.1 | up |
| 516 | Q5KQF4 | Os05g0334400 protein | Os05g0334400 | up |
| 517 | A0A0P0WL90 | Os05g0362500 protein | Os05g0362500 | up |
| 518 | Q0DJ33 | Guanine nucleotide-binding protein alpha-1 subunit | GPA1 | up |
| 519 | Q0IT01 | Os11g0433900 protein | OJ1612_A04.113 | up |
| 520 | Q0DU92 | Os03g0199000 protein (Fragment) | Os03g0199000 | up |
| 521 | A0A8J8YMH9 | Os12g0159600 protein | Os12g0159600 | up |
| 522 | B9FLZ7 | Translocon-associated protein subunit beta | OsJ_16788 | up |
| 523 | B9GCT1 | Uncharacterized protein | OsJ_35848 | up |
| 524 | Q67UK2 | Defective in cullin neddylation protein | Os06g0233400 | up |
| 525 | A0A8J8XJL9 | Transmembrane 9 superfamily member | Os12g0175700 | up |
| 526 | Q10G26 | Cell division protein ftsZ, putative, expressed | LOC_Os03g44420 | up |
| 527 | A0A8J8XUQ9 | Os03g0295500 protein | Os03g0295500 | up |
| 528 | A3AKN1 | Os03g0633900 protein | Os03g0633900 | up |
| 529 | Q0JJ35 | Mitochondrial import inner membrane translocase subunit TIM50 | Os01g0762600 | up |
| 530 | A0A0P0XUQ1 | Os10g0447100 protein (Fragment) | Os10g0447100 | up |
| 531 | Q75HK2 | Os03g0804200 protein | LOC_Os03g58940 | up |
| 532 | A3BTS5 | Os08g0455800 protein | Os08g0455800 | up |
| 533 | B9G3N6 | Uncharacterized protein | OsJ_29419 | up |
| 534 | Q0E3V2 | Protein YELLOW LEAF 1, choloroplastic | YL1 | up |
| 535 | A0A0P0W0Q9 | 5-nucleotidase | LOC_Os03g44660 | up |
| 536 | A0A0P0V9W5 | Os01g0826900 protein | Os01g0826900 | up |
| 537 | A2ZVM0 | Protein kinase domain-containing protein | OsJ_02684 | up |
| 538 | Q0DTG6 | Os03g0249200 protein | Os03g0249200 | up |
| 539 | A0A0P0XQD2 | Os09g0560300 protein | Os09g0560300 | up |
| 540 | B9EXB9 | Pre-mRNA-processing protein 40A | OsJ_02081 | up |
| 541 | B9G1U9 | J domain-containing protein | OsJ_27983 | up |
| 542 | Q0JD68 | COP9 signalosome complex subunit 8 | Os04g0428900 | up |
| 543 | Q2QXS4 | Os12g0145100 protein | LOC_Os12g05050 | up |
| 544 | B9EX81 | Alpha-galactosidase | OsJ_02009 | up |
| 545 | A0A0N7KI61 | Os03g0785200 protein | Os03g0785200 | up |
| 546 | B9FTN9 | Ribosomal protein | OsJ_21625 | up |
| 547 | C7J455 | sulfiredoxin | Os06g0174325 | up |
| 548 | Q69XV5 | Os06g0622300 protein | Os06g0622300 | up |
| 549 | B9FI55 | Uncharacterized protein ycf23 | Os05g0565400 | up |
| 550 | Q8S3S0 | Putative ring box-1 protein | 49D11.12 | up |
| 551 | B9GDQ4 | DUF4149 domain-containing protein | OsJ_36484 | up |
| 552 | Q0E3I4 | choline-phosphate cytidylyltransferase | Os02g0173500 | up |
| 553 | B0FFN1 | HR-like lesion-inducer family protein |  | up |
| 554 | Q2R3H7 | Acyl carrier protein | LOC_Os11g31900 | up |
| 555 | B9F9J1 | Uncharacterized protein | OsJ_11614 | up |
| 556 | Q60D95 | Os05g0188500 protein | Os05g0188500 | up |
| 557 | Q8S7W6 | Os03g0170300 protein | LOC_Os03g07420 | up |
| 558 | A3C4F7 | Uncharacterized protein | OsJ_31413 | up |
| 559 | Q69SK5 | Putative calcineurin B subunit | OsJ_08753 | up |
| 560 | B9FC09 | C2H2-type domain-containing protein | OsJ_15807 | up |
| 561 | A0A0P0WQN6 | Os05g0557200 protein | Os05g0557200 | up |
| 562 | Q10RP2 | GCN5-related N-acetyltransferase, putative, expressed | LOC_Os03g05710 | up |
| 563 | Q75ID5 | Os03g0581800 protein | Os03g0581800 | up |
| 564 | B9FPG6 | CNNM transmembrane domain-containing protein | OsJ_18448 | up |
| 565 | A0A8J8YCA6 | Os12g0170100 protein | Os12g0170100 | up |
| 566 | Q5WA91 | Uncharacterized protein P0681F10.12 | P0681F10.12 | up |
| 567 | Q8HCQ0 | NADH-ubiquinone oxidoreductase chain 3 | nad3 | up |
| 568 | Q5JLC8 | THO complex subunit 2 | P0413C03.13 | up |
| 569 | B9GCB6 | Protein kinase domain-containing protein | OsJ_35543 | up |
| 570 | Q5JJV3 | Os01g0966300 protein | Os01g0966300 | up |
| 571 | B9F6P7 | Endoplasmic reticulum transmembrane protein | OsJ_10088 | up |
| 572 | P49964 | Signal recognition particle 19 kDa protein | SRP19 | up |
| 573 | A2ZZT6 | AB hydrolase-1 domain-containing protein | OsJ_04157 | up |
| 574 | Q8L6H7 | Beta-glucosidase-like SFR2, chloroplastic | SFR2 | up |
| 575 | Q6K9B7 | Os02g0611400 protein | OJ1004_A05.9 | up |
| 576 | Q7Y0D5 | Transmembrane protein 147 | OSJNBa0079B15.14 | up |
| 577 | Q653G8 | Translation machinery associated TMA7 | P0623A10.17-1 | up |
| 578 | Q84Q90 | Uncharacterized protein OJ1041F02.15 | OJ1041F02.15 | up |
| 579 | Q657X8 | Peroxisomal membrane protein PEX14 | P0005A05.25-1 | up |
| 580 | Q7XIN7 | Putative MFP1 attachment factor 1 | OJ1710_H11.119 | up |
| 581 | A0A0P0XEX2 | Os08g0363800 protein | Os08g0363800 | up |
| 582 | A0A0P0UZ45 | Os01g0182200 protein | Os01g0182200 | up |
| 583 | B9EX70 | Trafficking protein particle complex subunit | OsJ_01989 | up |
| 584 | Q5ZD09 | Os01g0770500 protein | Os01g0770500 | up |
| 585 | Q0JQA5 | Os01g0173900 protein | Os01g0173900 | up |
| 586 | Q6K9R1 | Pumilio-like | OJ1202_E07.19 | up |
| 587 | A0A0P0V6X6 | Os01g0692600 protein | Os01g0692600 | up |
| 588 | B9FRA2 | Os06g0127500 protein | Os06g0127500 | up |
| 589 | Q6ZDC2 | Chloride channel protein | P0045D08.120 | up |
| 590 | A0A0P0W892 | OSJNBa0091C12.3 protein | Os04g0244800 | up |
| 591 | A0A0P0Y9E7 | Os12g0403800 protein | Os12g0403800 | up |
| 592 | B9F2K0 | Os02g0732500 protein | Os02g0732500 | up |
| 593 | B9G5S1 | NADP-dependent oxidoreductase domain-containing protein | OsJ_31541 | up |
| 594 | H2KW24 | Trans-2-enoyl-CoA reductase, mitochondrial, putative, expressed | LOC_Os11g01154 | up |
| 595 | C7IZZ8 | Os03g0612600 protein (Fragment) | Os03g0612600 | up |
| 596 | Q8HCR2 | Ribosomal protein S7 | rps7 | up |
| 597 | A0A0P0W8V9 | Os04g0306750 protein | Os04g0306750 | up |
| 598 | A0A0P0W1L8 | Os03g0687000 protein | Os03g0687000 | up |
| 599 | A3ABS9 | 1-aminocyclopropane-1-carboxylate oxidase | OJ1353_F08.16-1 | up |
| 600 | Q7JAI6 | Cytochrome c oxidase subunit 1 | cox1 | up |
| 601 | A3AB41 | Gnk2-homologous domain-containing protein | OsJ_08291 | up |
| 602 | Q0JIN2 | Os01g0788700 protein | Os01g0788700 | up |
| 603 | Q2QRN7 | Probable kinase CHARK | CHARK | up |
| 604 | B9F1M4 | Membrane bound O-acyl transferase-like | Os02g0676000 | up |
| 605 | A0A979HLD2 | Os01g0550800 protein | Os01g0550800 | up |
| 606 | A0A0P0WCM5 | Os04g0519925 protein | Os04g0519925 | up |
| 607 | B9ESZ8 | Uncharacterized protein | OsJ_00469 | up |
| 608 | B7EFX9 | cDNA clone:J023007E22, full insert sequence | OsJ_11907 | up |
| 609 | A0A8J8YNE2 | Reticulon-like protein | OsJ_19453 | up |
| 610 | B9F686 | Major facilitator superfamily (MFS) profile domain-containing protein | OsJ_09938 | up |
| 611 | Q84ZB9 | Os07g0549800 protein | Os07g0549800 | up |
| 612 | Q6L4S2 | Os05g0592000 protein | Os05g0592000 | up |
| 613 | A0A8J8Y9G4 | Glycosyltransferase | Os01g0697100 | up |
| 614 | A3C5W8 | C2H2-type domain-containing protein | OsJ_31952 | up |
| 615 | Q5Z8U4 | Putative LeOPT1 | P0018H04.22 | up |
| 616 | Q0JJD6 | Nuclear pore complex protein Nup85 | Os01g0746200 | up |
| 617 | Q7XXD6 | OSJNBa0039G19.13 protein | OSJNBa0039G19.13 | up |
| 618 | B9G419 | Os09g0465800 protein | Os09g0465800 | up |
| 619 | Q0DF13 | Succinate dehydrogenase subunit 8A, mitochondrial | SDH8A | up |
| 1 | Q943W1 | Os01g0501800 protein | Os01g0501800 | dowm |
| 2 | A0A0N9E0R7 | Photosystem II CP47 reaction center protein | psbB | dowm |
| 3 | Q7G3F1 | Cytochrome f | LOC_Os10g21290 | dowm |
| 4 | E9KIP0 | Photosystem I P700 chlorophyll a apoprotein A2 | psaB | dowm |
| 5 | Q6Z1U4 | Photosystem II D2 protein | OSJNBa0022A24.62 | dowm |
| 6 | Q6Z510 | Ribosomal protein S3 | OSJNBa0036E18.21 | dowm |
| 7 | Q6ER49 | Peroxidase | prx29 | dowm |
| 8 | A3BVS4 | Photosystem I reaction center subunit II, chloroplastic | Os08g0560900 | dowm |
| 9 | Q8S6G5 | Putative PSII 43kDa protein from chromosome 10 chloroplast insertion | OSJNBb0075K12.22 | dowm |
| 10 | Q6EUK5 | Os02g0234500 protein | Os02g0234500 | dowm |
| 11 | A3BKU8 | Uncharacterized protein | OsJ_24632 | dowm |
| 12 | A0A8J8Y8Q7 | Os03g0284400 protein | Os03g0284400 | dowm |
| 13 | B9FZJ0 | Photosystem II 10 kDa polypeptide, chloroplastic | OsJ_26372 | dowm |
| 14 | A0A7S7YAD2 | 50S ribosomal protein L2, chloroplastic | rpl2 | dowm |
| 15 | Q9ZST0 | 50S ribosomal protein L5, chloroplastic | RPL5 | dowm |
| 16 | A0A0P0W8S4 | Os04g0304200 protein | Os04g0304200 | dowm |
| 17 | B9FSL7 | Os06g0258900 protein | P0603C10.39-1 | dowm |
| 18 | Q60E59 | Ribosomal protein | Os05g0388500 | dowm |
| 19 | Q6Z3V7 | Putative Photosystem I reaction center subunit IV | OsJ_24033 | dowm |
| 20 | Q0J6R9 | Os08g0276100 protein | Os08g0276100 | dowm |
| 21 | A0A7S8BBD3 | Photosystem I P700 chlorophyll a apoprotein A1 | psaA | dowm |
| 22 | Q0DCJ6 | Chlorophyll a-b binding protein, chloroplastic | Os06g0320500 | dowm |
| 23 | Q0IPL2 | Os12g0189400 protein | Os12g0189400 | dowm |
| 24 | Q10MB2 | 30S ribosomal protein S1, chloroplast, putative, expressed | LOC_Os03g20100 | dowm |
| 25 | Q8S6H1 | Photosystem II protein D1 | LOC_Os10g21200 | dowm |
| 26 | Q0E446 | Os02g0137200 protein | Os02g0137200 | dowm |
| 27 | B9FRS2 | galactinol--sucrose galactosyltransferase | OsJ_20295 | dowm |
| 28 | Q10PV6 | 50S ribosomal protein L15, chloroplast, putative, expressed | LOC_Os03g12020 | dowm |
| 29 | A0A0N7KDR4 | Os01g0749200 protein | Os01g0749200 | dowm |
| 30 | Q850W6 | Os03g0452300 protein | LOC_Os03g34040 | dowm |
| 31 | A3BHI1 | Os07g0196200 protein | Os07g0196200 | dowm |
| 32 | A3AI13 | Ribosomal protein L6 alpha-beta domain-containing protein | OsJ_10878 | dowm |
| 33 | Q850L8 | 50S ribosomal protein L6, putative, expressed | OSJNBa0032E21.12 | dowm |
| 34 | Q69S39 | Cytochrome b6-f complex iron-sulfur subunit, chloroplastic | petC | dowm |
| 35 | A0A0N7KH18 | Os03g0281600 protein (Fragment) | Os03g0281600 | dowm |
| 36 | Q7DNB2 | NAD(P)H dehydrogenase subunit H | OSJNBa0061C08.3 | dowm |
| 37 | Q0J1V9 | Dihydrolipoamide acetyltransferase component of pyruvate dehydrogenase complex | Os09g0408600 | dowm |
| 38 | E9KIT0 | Photosystem I iron-sulfur center | psaC | dowm |
| 39 | Q653V9 | Os06g0683200 protein | Os06g0683200 | dowm |
| 40 | Q8RYZ1 | Os01g0874700 protein | P0648C09.9 | dowm |
| 41 | A0A0N9DZK6 | 30S ribosomal protein S2, chloroplastic | rps2 | dowm |
| 42 | Q6YXE9 | Eukaryotic translation initiation factor 3 subunit I | B1052H09.108 | dowm |
| 43 | P12153 | 30S ribosomal protein S19, chloroplastic | rps19-B | dowm |
| 44 | Q5U1N4 | Peroxidase | prx59 | dowm |
| 45 | A3A587 | Os02g0259600 protein | Os02g0259600 | dowm |
| 46 | Q7XIK0 | Os07g0565600 protein | Os07g0565600 | dowm |
| 47 | A0A7S8BCX7 | 30S ribosomal protein S4, chloroplastic | rps4 | dowm |
| 48 | Q6YUV4 | Os02g0189000 protein | Os02g0189000 | dowm |
| 49 | Q0IQR0 | Ferritin | Os12g0106000 | dowm |
| 50 | Q8L472 | Os07g0105600 protein | P0617C02.114 | dowm |
| 51 | Q8W0E6 | Chlorophyll a-b binding protein, chloroplastic | Os01g0720500 | dowm |
| 52 | A0A5S6R9G6 | 50S ribosomal protein L9, chloroplastic | OJ1119_A01.23-1 | dowm |
| 53 | A0A8J8YPA5 | Os03g0350300 protein | Os03g0350300 | dowm |
| 54 | A3CHK1 | J domain-containing protein | OsJ_36173 | dowm |
| 55 | Q84QA8 | Uncharacterized protein OJ1012B02.13 | OJ1012B02.13 | dowm |
| 56 | Q6Z6A0 | Os02g0754300 protein | Os02g0754300 | dowm |
| 57 | B9F5P8 | Os03g0196800 protein | Os03g0196800 | dowm |
| 58 | B9EW08 | Uncharacterized protein | OsJ_04636 | dowm |
| 59 | P0C443 | 50S ribosomal protein L16, chloroplastic | rpl16 | dowm |
| 60 | A3AP92 | Uncharacterized protein | OsJ_13194 | dowm |
| 61 | A0A8J8YMQ7 | DUF3700 domain-containing protein | OsJ_16640 | dowm |
| 62 | Q53RP2 | Remorin, C-terminal region family protein, expressed | LOC_Os10g36000 | dowm |
| 63 | B9F5W8 | 30S ribosomal protein S9 | OsJ_12727 | dowm |
| 64 | Q0DG05 | Photosystem I reaction center subunit VI, chloroplastic | PSAH | dowm |
| 65 | Q8SAY0 | 50S ribosomal protein L18, chloroplastic | RPL18 | dowm |
| 66 | B9EWK2 | Uncharacterized protein | OsJ_04839 | dowm |
| 67 | A2ZWI7 | Os01g0678600 protein | Os01g0678600 | dowm |
| 68 | A3BI67 | Os07g0243150 protein | P0418E08.140 | dowm |
| 69 | A0A0N9E0Z0 | 50S ribosomal protein L22, chloroplastic | rpl22 | dowm |
| 70 | Q10FT7 | Hsp20/alpha crystallin family protein, expressed | LOC_Os03g45340 | dowm |
| 71 | Q6ZGW6 | Delta-12 fatty acid desaturase | FAD2 | dowm |
| 72 | Q7XAM2 | Os07g0599000 protein | Os07g0599000 | dowm |
| 73 | A0A8J8XZB2 | Putative carnitine/acylcarnitine translocase | OsJ_32559 | dowm |
| 74 | B9FAJ0 | Os03g0122200 protein | Os03g0122200 | dowm |
| 75 | A0A0P0VKV9 | Os02g0581100 protein | Os02g0581100 | dowm |
| 76 | P12124 | NAD(P)H-quinone oxidoreductase subunit 1, chloroplastic | ndhA | dowm |
| 77 | Q10LH9 | Nuclear pore protein | LOC_Os03g22690 | dowm |
| 78 | H2KWR6 | PSI subunit V | LOC_Os12g23200 | dowm |
| 79 | Q8H656 | Putative plastid protein | OSJNBa0019F11.29-2 | dowm |
| 80 | B9F4N8 | Uncharacterized protein | OsJ_08984 | dowm |
| 81 | H2KWG3 | PWWP domain containing protein, expressed | LOC_Os11g19810 | dowm |
| 82 | Q69U53 | Os08g0103500 protein | Os08g0103500 | dowm |
| 83 | Q5VRM0 | Acyl-CoA-binding domain-containing protein 2 | ACBP2 | dowm |
| 84 | Q6ZKM2 | COP9 signalosome complex subunit 6 | CSN6 | dowm |
| 85 | Q6K973 | Sucrose synthase 6 | SUS6 | dowm |
| 86 | O65037 | 50S ribosomal protein L27, chloroplastic | RPL27 | dowm |
| 87 | P12139 | 50S ribosomal protein L20, chloroplastic | rpl20 | dowm |
| 88 | Q84SD6 | NADH dehydrogenase [ubiquinone] iron-sulfur protein 6, mitochondrial | Os08g0161700 | dowm |
| 89 | B9FFD0 | Os04g0442200 protein | Os04g0442200 | dowm |
| 90 | A0A0N9E0L8 | 30S ribosomal protein S7, chloroplastic | rps7 | dowm |
| 91 | Q69JU4 | Os02g0578400 protein | Os02g0578400 | dowm |
| 92 | B9F7D0 | Os03g0279950 protein | Os03g0279950 | dowm |
| 93 | A0A7S8BBD9 | Cytochrome b6 | petB | dowm |
| 94 | B9FPB7 | Uncharacterized protein | OsJ_18363 | dowm |
| 95 | Q8RVB1 | 50S ribosomal protein L31 | Os01g0633000 | dowm |
| 96 | Q6ZAA3 | Os08g0432500 protein | Os08g0432500 | dowm |
| 97 | Q5JPZ5 | OSJNBa0004L19.19 protein | OSJNBa0004L19.19 | dowm |
| 98 | P12151 | 30S ribosomal protein S16, chloroplastic | rps16 | dowm |
| 99 | Q0DQ67 | Os03g0627500 protein (Fragment) | Os03g0627500 | dowm |
| 100 | Q851F9 | Probable zinc metalloprotease EGY3, chloroplastic | EGY3 | dowm |
| 101 | A0A5S6R8K8 | p0035H10.3 protein | P0035H10.3 | dowm |
| 102 | Q10L97 | NAD dependent epimerase/dehydratase family protein, expressed | LOC_Os03g23980 | dowm |
| 103 | Q0DJC5 | Dolichyl-diphosphooligosaccharide--protein glycosyltransferase subunit 1A | OST1A | dowm |
| 104 | A0A0P0VLU4 | Os02g0610700 protein | OJ1476_F05.21-1 | dowm |
| 105 | Q10RN9 | Os03g0151900 protein | LOC_Os03g05740 | dowm |
| 106 | A0A0P0WV36 | Os06g0237300 protein | Os06g0237300 | dowm |
| 107 | Q5KQD5 | Os05g0101400 protein | Os05g0101400 | dowm |
| 108 | B7F7C1 | cDNA clone:J033007H10, full insert sequence |  | dowm |
| 109 | B9F7Z6 | Os03g0856400 protein | Os03g0856400 | dowm |
| 110 | Q0J032 | Lon protease homolog 2, peroxisomal | Os09g0533400 | dowm |
| 111 | Q0DGW5 | Os05g0508300 protein | Os05g0508300 | dowm |
| 112 | A3ANB5 | Actin-related protein 7 | ARP7 | dowm |
| 113 | Q5Z754 | Cyclin-dependent kinase F-1 | CDKF-1 | dowm |
| 114 | A0A0P0WB26 | OSJNBa0072F16.12 protein | Os04g0461100 | dowm |
| 115 | B9F1E6 | MSP domain-containing protein | OsJ_07762 | dowm |
| 116 | P0C457 | 50S ribosomal protein L33, chloroplastic | rpl33 | dowm |
| 117 | Q10GG7 | H1flk, putative, expressed | LOC_Os03g43010 | dowm |
| 118 | Q75K88 | Os05g0496500 protein | Os05g0496500 | dowm |
| 119 | A3BCW2 | Transmembrane 9 superfamily member | OsJ_21738 | dowm |
| 120 | B9FLK8 | TATA element modulatory factor 1 TATA binding domain-containing protein | OsJ_19523 | dowm |
| 121 | Q7X7T4 | Protoporphyrinogen oxidase | OSJNBa0076N16.4 | dowm |
| 122 | B9FL15 | Calreticulin | OsJ_19137 | dowm |
| 123 | B9FGH3 | Plastid lipid-associated protein/fibrillin conserved domain-containing protein | OsJ_15704 | dowm |
| 124 | Q5N9A0 | Chloroplast cytochrome b/f | P0557A01.12 | dowm |
| 125 | Q2R3B6 | Os11g0528500 protein | LOC_Os11g32500 | dowm |
| 126 | Q9FE64 | Elongation factor G, mitochondrial | Os03g0565500 | dowm |
| 127 | Q9MAX5 | Coatomer subunit zeta-1 | COPZ1 | dowm |
| 128 | A3BT01 | Os08g0407200 protein | P0453D01.16-1 | dowm |
| 129 | A0A7S8BBD6 | Photosystem II reaction center protein L | psbL | dowm |
| 130 | A3AY48 | Uncharacterized protein | OsJ_16441 | dowm |
| 131 | A2ZU58 | Peroxidase | OsJ_02142 | dowm |
| 132 | B9FFE3 | Signal peptidase complex subunit 2 | Os04g0446300 | dowm |
| 133 | B9FH79 | Uncharacterized protein | OsJ_18173 | dowm |
| 134 | Q2QPG9 | Probable histone H2AXb | Os12g0530000 | dowm |
| 135 | Q5NB36 | Eukaryotic translation initiation factor 3 subunit L | Os01g0229100 | dowm |
| 136 | A0A0P0X3U2 | Os07g0184800 protein | OJ1046_F10.127 | dowm |
| 137 | B9EWQ4 | Uncharacterized protein | OsJ_01749 | dowm |
| 138 | A0A7S7YC46 | NAD(P)H-quinone oxidoreductase chain 4, chloroplastic | ndhD | dowm |
| 139 | C7J2D7 | Os05g0497675 protein | Os05g0497675 | dowm |
| 140 | A0A0P0XBZ8 | Os08g0159100 protein | P0498E12.115 | dowm |
| 141 | B9FRC7 | 2-phosphotransferase | Os06g0134050 | dowm |
| 142 | A3C154 | glutathione transferase | OsJ_30203 | dowm |
| 143 | Q5Z408 | Uncharacterized protein B1206D04.20 | B1206D04.20 | dowm |
| 144 | Q0J4I3 | AT-hook motif nuclear-localized protein | Os08g0512400 | dowm |
| 145 | A3AYN2 | OSJNBa0064G10.22 protein | Os04g0678200 | dowm |
| 146 | A0A0P0XKP6 | Chlorophyll a-b binding protein, chloroplastic | Os09g0296800 | dowm |
| 147 | Q2QW75 | Os12g0204500 protein | LOC_Os12g10320 | dowm |
| 148 | A0A0P0VMW2 | Os02g0670500 protein | Os02g0670500 | dowm |
| 149 | Q9FE02 | Cytochrome c oxidase subunit | COX6b2 | dowm |
| 150 | C7IXN6 | Carbonic anhydrase | Os01g0640132 | dowm |
| 151 | Q0JC14 | OSJNBa0067K08.20 protein | Os04g0497900 | dowm |
| 152 | B9F0U0 | Cyclin G-associated kinase-like protein | OsJ_07336 | dowm |
| 153 | Q0J8W3 | Os04g0684500 protein | Os04g0684500 | dowm |
| 154 | A0A0P0WCB4 | OSJNBa0067K08.9 protein | Os04g0496400 | dowm |
| 155 | Q8GSC2 | Os07g0628900 protein | Os07g0628900 | dowm |
| 156 | Q5VRH4 | Homogentisate 1,2-dioxygenase | HGO | dowm |
| 157 | A0A0N7KMI0 | 50S ribosomal protein L35 | Os06g0647100 | dowm |
| 158 | B9FAD9 | THO complex subunit 1 | OsJ_09127 | dowm |
| 159 | Q5KQL0 | Rev interacting-like family protein, putative, expressed | LOC_Os03g63710 | dowm |
| 160 | Q8RZ80 | Endopeptidase-like protein | B1065G12.15 | dowm |
| 161 | Q6ZGL9 | Signal peptide peptidase 1 | SPP1 | dowm |
| 162 | Q69QQ2 | Os09g0482200 protein | Os09g0482200 | dowm |
| 163 | B7E324 | Os05g0186000 protein | Os05g0186000 | dowm |
| 164 | Q0IVZ4 | Os10g0545300 protein | Os10g0545300 | dowm |
| 165 | Q5Z5X5 | Os06g0713100 protein | Os06g0713100 | dowm |
| 166 | Q7XRF0 | OSJNBa0006M15.17 protein | OSJNBa0006M15.17 | dowm |
| 167 | A0A0N7KDR2 | Os01g0747700 protein | Os01g0747700 | dowm |
| 168 | Q5VR53 | Os01g0807900 protein | P0702B09.41-2 | dowm |
| 169 | Q0J0D1 | Os09g0516200 protein | Os09g0516200 | dowm |
| 170 | Q7F613 | Secretory carrier-associated membrane protein 2 | SCAMP2 | dowm |
| 171 | Q8GRJ3 | Glycosyl hydrolase family 17-like protein | OJ1634_B10.124 | dowm |
| 172 | B9FU28 | Uncharacterized protein | OsJ_21922 | dowm |
| 173 | Q10CP1 | COBRA-like protein 4, putative, expressed | LOC_Os03g54750 | dowm |
| 174 | A3AGV0 | Os03g0288700 protein | Os03g0288700 | dowm |
| 175 | A0A8J8YIS5 | Os03g0139000 protein | Os03g0139000 | dowm |
| 176 | A0A0N9E0S3 | Protein PsbN | psbN | dowm |
| 177 | Q6K9X2 | Os02g0823600 protein | Os02g0823600 | dowm |
| 178 | B9FVR5 | FRIGIDA-like protein | OsJ_23273 | dowm |
| 179 | Q0DHL9 | Os05g0457700 protein | Os05g0457700 | dowm |
| 180 | Q6Z5S4 | Os07g0268800 protein | Os07g0268800 | dowm |
| 181 | P0CD23 | NAD(P)H-quinone oxidoreductase subunit 2 B, chloroplastic | ndhB2 | dowm |
| 182 | Q8H7Y2 | Uncharacterized protein OJ1607A12.16 | OJ1607A12.16 | dowm |
| 183 | E9KIQ3 | Cytochrome b559 subunit beta | psbF | dowm |
| 184 | A3BKC8 | RRM domain-containing protein | OsJ_24455 | dowm |
| 185 | Q5ZCB0 | Putative Bowman Birk trypsin inhibitor | P0037C04.15 | dowm |
| 186 | A0A0P0WJN4 | Os05g0241200 protein | Os05g0241200 | dowm |
| 187 | A3ATQ2 | OJ991214_12.3 protein | Os04g0414700 | dowm |
| 188 | Q0DVC7 | Os03g0136800 protein | Os03g0136800 | dowm |
| 189 | Q0IW04 | chitinase | OSJNBb0015I11.16 | dowm |
| 190 | A0A0P0VG76 | Os02g0207900 protein | Os02g0207900 | dowm |
| 191 | Q5JKM1 | Os01g0940600 protein | Os01g0940600 | dowm |
| 192 | B9FVT2 | Uncharacterized protein | OsJ_23300 | dowm |

## Table S4. Differential protein subcellular localization 5 mg L^-1^.vs.Control

| **Table S4. Differential protein subcellular localization 5 mg L^-1^.vs.Control** | | | |
| --- | --- | --- | --- |
| **Protein id** | **Gene** | **Subcellular localization** | **Up/dowm** |
| A3A3E9 | OsJ_05484 | cell membrane protein | up |
| Q2QYY2 | LOC_Os12g01200 | cell membrane protein | up |
| Q69U53 | Os08g0103500 | cell membrane protein | down |
| Q339G9 | LOC_Os10g21280 | chloroplast protein | up |
| Q75IZ9 | LOC_Os03g29950 | chloroplast protein | up |
| A0A0P0WWY2 | Os06g0367100 | chloroplast protein | up |
| Q6Z0I4 | Os08g0327400 | chloroplast protein | up |
| A0A8J8YN91 | OsJ_11111 | chloroplast protein | up |
| Q2QUI0 | LOC_Os12g16200 | chloroplast protein | up |
| B9FAM9 | OsJ_12142 | chloroplast protein | up |
| A0A7S8BAT2 | rps11 | chloroplast protein | up |
| A0A0P0WCB4 | Os04g0496400 | chloroplast protein | down |
| B9F6U6 | OsJ_10181 | chloroplast protein | down |
| Q10G20 | LOC_Os03g44484 | chloroplast protein | down |
| Q6K5H0 | NBP35 | chloroplast protein | down |
| Q76FS2 | TUBB8 | cytoplasm protein | up |
| P48489 | Os03g0268000 | cytoplasm protein | up |
| D7PPK3 | adh1 | cytoplasm protein | up |
| A3CCT6 | OsJ_34439 | cytoplasm protein | down |
| Q6ES52 | Os09g0401200 | cytoplasm protein | down |
| E3WF09 | r | cytoplasm protein | down |
| B9ETN3 | OsJ_00747 | cytoplasm protein | down |
| B9FJH8 | Os05g0460000 | endoplasmic reticulum protein | up |
| A0A0P0UZ45 | Os01g0182200 | endoplasmic reticulum protein | up |
| Q0IZL5 | Os09g0562700 | endoplasmic reticulum protein | down |
| Q0DEK1 | P0036F10.45-1 | extracell protein | up |
| A0A8J8Y991 | Os01g0839900 | extracell protein | up |
| Q5JNF3 | Os01g0730500 | mitochondrion protein | up |
| Q8HCQ0 | nad3 | mitochondrion protein | up |
| Q0ITU4 | Os11g0216100 | mitochondrion protein | down |
| Q9LGH8 | H2B.8 | nucleus protein | up |
| A0A8J8XXT2 | Os03g0116500 | nucleus protein | up |
| Q10FT7 | LOC_Os03g45340 | nucleus protein | down |
| A0A0P0VGU6 | Os02g0236800 | peroxisome protein | up |
| Q7XUY5 | Os04g0465600 | vacuole protein | up |
| Q84LM2 | VPE1 | vacuole protein | up |
| Q0IW04 | OSJNBb0015I11.16 | vacuole protein | down |

## Table S5. Differential protein subcellular localization 10 mg L^-1^.vs.Control

| **Table S5. Differential protein subcellular localization 10 mg L^-1^.vs.Control** | | | |
| --- | --- | --- | --- |
| **Protein id** | **Gene** | **Subcellular localization** | **Up/down** |
| Q0JNS7 | Os01g0267800 | cell membrane protein | up |
| A3AAD9 | Os02g0697600 | cell membrane protein | up |
| A0A0P0Y0G8 | Os11g0183900 | cell membrane protein | up |
| A2ZVM0 | OsJ_02684 | cell membrane protein | up |
| A0A0P0V4T6 | Os01g0598600 | cell membrane protein | down |
| Q6H454 | B1040D06.2 | cell membrane protein | down |
| Q60E30 | OSJNBb0012L23.7 | cell membrane protein | down |
| Q8GSC2 | Os07g0628900 | cell membrane protein | down |
| A3C107 | Os09g0537700 | cell wall protein | up |
| B9FH99 | OSJNBa0039O18.8 | cell wall protein | down |
| A0A0P0Y838 | Os12g0207600 | chloroplast protein | up |
| B9FI18 | OsJ_18396 | chloroplast protein | up |
| Q2RAP8 | LOC_Os11g04670 | chloroplast protein | up |
| Q65XK0 | Os05g0573700 | chloroplast protein | up |
| Q0J4W9 | Os08g0484500 | chloroplast protein | up |
| Q8LJJ2 | Os01g0693800 | chloroplast protein | up |
| Q5NBJ3 | GYRB | chloroplast protein | up |
| Q0IRR5 | Os11g0603200 | chloroplast protein | up |
| C6YXK3 | LOC_Os11g07020 | chloroplast protein | up |
| Q75IZ9 | LOC_Os03g29950 | chloroplast protein | up |
| A0A0P0WWY2 | Os06g0367100 | chloroplast protein | up |
| Q9LRE9 | ALDH1a | chloroplast protein | up |
| Q6EPN6 | ISPF | chloroplast protein | up |
| Q0D557 | Os07g0580900 | chloroplast protein | up |
| Q6ZAA5 | Os08g0447000 | chloroplast protein | up |
| Q657X6 | EX2 | chloroplast protein | up |
| A0A0P0WDN1 | Os04g0573200 | chloroplast protein | up |
| A3BMP5 | P0524G08.116 | chloroplast protein | up |
| B9FU89 | Os07g0618600 | chloroplast protein | up |
| Q2R3H7 | LOC_Os11g31900 | chloroplast protein | up |
| A0A0P0WJN4 | Os05g0241200 | chloroplast protein | down |
| A0A7S7YAD2 | rpl2 | chloroplast protein | down |
| Q10MB2 | LOC_Os03g20100 | chloroplast protein | down |
| A0A0N7KDR4 | Os01g0749200 | chloroplast protein | down |
| Q84QA8 | OJ1012B02.13 | chloroplast protein | down |
| P0C443 | rpl16 | chloroplast protein | down |
| Q8RVB1 | Os01g0633000 | chloroplast protein | down |
| Q5KQD5 | Os05g0101400 | chloroplast protein | down |
| B9F1E6 | OsJ_07762 | chloroplast protein | down |
| A0A0P0WCB4 | Os04g0496400 | chloroplast protein | down |
| A0A0P0WIM3 | Os05g0170800 | chloroplast protein | down |
| B9FRD6 | OsJ_20044 | cytoplasm protein | up |
| Q2QXR8 | Os12g0145700 | cytoplasm protein | up |
| A0A8J8YSG5 | OsJ_19441 | cytoplasm protein | up |
| B9F6J9 | Os03g0804800 | cytoplasm protein | up |
| Q6H4V1 | OSJNBa0085K21.52 | cytoplasm protein | up |
| Q6Z6Y1 | Os02g0130100 | cytoplasm protein | up |
| Q0IM82 | Os12g0589100 | cytoplasm protein | up |
| Q5VND2 | Os06g0149900 | cytoplasm protein | up |
| Q8RYZ1 | P0648C09.9 | cytoplasm protein | down |
| Q0DGR3 | Os05g0516600 | cytoplasm protein | down |
| A0A0P0VC29 | Os01g0919700 | endoplasmic reticulum protein | up |
| B9FL15 | OsJ_19137 | endoplasmic reticulum protein | down |
| Q7XTJ3 | Os04g0119400 | mitochondrion protein | up |
| Q0DFB6 | Os06g0103600 | mitochondrion protein | up |
| Q75IM9 | Os05g0125500 | mitochondrion protein | up |
| A0A0P0UYL6 | Os01g0143800 | mitochondrion protein | up |
| Q7XZF7 | GYRA | mitochondrion protein | up |
| Q5JJI4 | TOM20 | mitochondrion protein | up |
| Q69LD2 | Os07g0170100 | mitochondrion protein | up |
| Q0JJ35 | Os01g0762600 | mitochondrion protein | up |
| B9G1U9 | OsJ_27983 | mitochondrion protein | up |
| P0CD23 | ndhB2 | mitochondrion protein | down |
| A3AI13 | OsJ_10878 | mitochondrion protein | down |
| Q850L8 | OSJNBa0032E21.12 | mitochondrion protein | down |
| A0A8J8XZB2 | OsJ_32559 | mitochondrion protein | down |
| A0A0P0WV17 | Os06g0237502 | nucleus protein | up |
| P31673 | HSP17.4 | nucleus protein | up |
| Q6Z8U4 | Os08g0492100 | nucleus protein | up |
| B9FC09 | OsJ_15807 | nucleus protein | up |
| Q75HX0 | Os05g0438800 | nucleus protein | down |
| Q10FT7 | LOC_Os03g45340 | nucleus protein | down |
| Q9AWU6 | P0044F08.16 | nucleus protein | down |
| A3A2X0 | Os02g0135800 | nucleus protein | down |
| Q0JEQ2 | APX3 | peroxisome protein | up |
| B9F1F2 | Os02g0654100 | peroxisome protein | up |
| A0A0N7KML5 | P0547F09.20-1 | peroxisome protein | down |
| Q0IM13 | Os12g0607100 | plastid protein | up |
| A0A8J8XMH3 | Os12g0609500 | plastid protein | up |
| A0A0N9E0Z0 | rpl22 | plastid protein | down |
| P12139 | rpl20 | plastid protein | down |
| A0A0P0WB26 | Os04g0461100 | plastid protein | down |
| P0C457 | rpl33 | plastid protein | down |
| Q5W6Z6 | Os05g0247100 | vacuole protein | up |
| Q5JNJ1 | Os01g0749400 | vacuole protein | up |
| A3BDG7 | Os06g0609600 | vacuole protein | up |
| Q0IW04 | OSJNBb0015I11.16 | vacuole protein | down |
| Q7XUY5 | Os04g0465600 | vacuole protein | down |

## Table S6. Differential protein subcellular localization 20 mg L^-1^.vs.Control

| **Table S6. Differential protein subcellular localization 20 mg L^-1^.vs.Control** | | | |
| --- | --- | --- | --- |
| **Protein id** | **Gene** | **Subcellular localization** | **Up/down** |
| A0A8J8Y500 | Os03g0151800 | cell membrane protein | up |
| A0A8J8YN06 | OsJ_31677 | cell membrane protein | up |
| Q0JIN2 | Os01g0788700 | cell membrane protein | up |
| Q2QRN7 | CHARK | cell membrane protein | up |
| B7EFX9 | OsJ_11907 | cell membrane protein | up |
| B9F686 | OsJ_09938 | cell membrane protein | up |
| Q5Z5T3 | Os06g0567900 | cell membrane protein | up |
| Q0DGP6 | NSF | cell membrane protein | up |
| Q6Z671 | Os02g0720900 | cell membrane protein | up |
| A2ZVG7 | FTSH9 | cell membrane protein | up |
| A0A0P0WS09 | Os06g0106800 | cell membrane protein | up |
| Q2QYY2 | LOC_Os12g01200 | cell membrane protein | up |
| A3AAD9 | Os02g0697600 | cell membrane protein | up |
| Q75LS2 | LOC_Os03g27320 | cell membrane protein | up |
| Q9FNU2 | ABCB25 | cell membrane protein | up |
| Q0DHL4 | FTSH8 | cell membrane protein | up |
| A0A0P0Y0G8 | Os11g0183900 | cell membrane protein | up |
| A3BFD4 | Os06g0714500 | cell membrane protein | up |
| A3ADQ4 | OsJ_09260 | cell membrane protein | up |
| Q75HK2 | LOC_Os03g58940 | cell membrane protein | up |
| A2ZVM0 | OsJ_02684 | cell membrane protein | up |
| Q69SK5 | OsJ_08753 | cell membrane protein | up |
| Q0JQA5 | Os01g0173900 | cell membrane protein | up |
| Q8GRJ3 | OJ1634_B10.124 | cell membrane protein | down |
| Q10CP1 | LOC_Os03g54750 | cell membrane protein | down |
| Q53RP2 | LOC_Os10g36000 | cell membrane protein | down |
| Q69U53 | Os08g0103500 | cell membrane protein | down |
| Q5JPZ5 | OSJNBa0004L19.19 | cell membrane protein | down |
| A0A5S6R8K8 | P0035H10.3 | cell membrane protein | down |
| Q0DGW5 | Os05g0508300 | cell membrane protein | down |
| Q8GSC2 | Os07g0628900 | cell membrane protein | down |
| Q69QQ2 | Os09g0482200 | cell membrane protein | down |
| A3AB41 | OsJ_08291 | cell wall protein | up |
| A3C107 | Os09g0537700 | cell wall protein | up |
| A0A0N7KR67 | P0229B10.19-2 | cell wall protein | up |
| A0A6B7JG68 |  | cell wall protein | up |
| A0A8J8YB53 | Os12g0115300 | cell wall protein | up |
| A0A0P0WNP9 | Os05g0477900 | cell wall protein | up |
| Q0INX9 | Os12g0277500 | chloroplast protein | up |
| B9FGD1 | OsJ_15609 | chloroplast protein | up |
| Q0DF58 | Os06g0114000 | chloroplast protein | up |
| B9FI18 | OsJ_18396 | chloroplast protein | up |
| Q75GT3 | CLPB2 | chloroplast protein | up |
| Q0E0Z3 | Os02g0510200 | chloroplast protein | up |
| Q65XK0 | Os05g0573700 | chloroplast protein | up |
| B9GCW1 | OsJ_35903 | chloroplast protein | up |
| A3B9A1 | P0528E04.36-1 | chloroplast protein | up |
| Q6YZE2 | GSA | chloroplast protein | up |
| Q10LR9 | Os03g0337600 | chloroplast protein | up |
| Q8W250 | DXR | chloroplast protein | up |
| A2ZW27 | OsJ_02845 | chloroplast protein | up |
| Q10MQ2 | AGD2 | chloroplast protein | up |
| Q2QTC2 | GWD3 | chloroplast protein | up |
| Q0J4W9 | Os08g0484500 | chloroplast protein | up |
| A0A8J8XQK2 | OsJ_08078 | chloroplast protein | up |
| Q6ATS0 | CHLD | chloroplast protein | up |
| Q8LJJ2 | Os01g0693800 | chloroplast protein | up |
| Q10R17 | PURA1 | chloroplast protein | up |
| Q5NBJ3 | GYRB | chloroplast protein | up |
| Q0IRR5 | Os11g0603200 | chloroplast protein | up |
| B9FU86 | OsJ_25140 | chloroplast protein | up |
| Q0JJS8 | HCF101 | chloroplast protein | up |
| A0A0P0WFN9 | Os04g0650800 | chloroplast protein | up |
| Q5NAY4 | HDH | chloroplast protein | up |
| Q2QTQ1 | LOC_Os12g18900 | chloroplast protein | up |
| Q10NY1 | LOC_Os03g14990 | chloroplast protein | up |
| Q10MW3 | PDC2 | chloroplast protein | up |
| Q2QX01 | GRXS12 | chloroplast protein | up |
| A0A0P0XM43 | OJ1294_G06.18 | chloroplast protein | up |
| B9F3P2 | OsJ_05634 | chloroplast protein | up |
| Q0D935 | OJ1567_G09.119 | chloroplast protein | up |
| Q0D867 | Os07g0181000 | chloroplast protein | up |
| A0A0P0Y344 | Os11g0546000 | chloroplast protein | up |
| Q9AXB0 | Os01g0622300 | chloroplast protein | up |
| Q10G39 | Os03g0645100 | chloroplast protein | up |
| A0A0P0WWY2 | Os06g0367100 | chloroplast protein | up |
| Q6Z0I4 | Os08g0327400 | chloroplast protein | up |
| P0C587 | Os10g0502400 | chloroplast protein | up |
| Q655Y9 | Os06g0664200 | chloroplast protein | up |
| Q7XM43 | OSJNBb0018J12.16 | chloroplast protein | up |
| Q9LRE9 | ALDH1a | chloroplast protein | up |
| Q6EPN6 | ISPF | chloroplast protein | up |
| Q0D557 | Os07g0580900 | chloroplast protein | up |
| P55142 | GRXC6 | chloroplast protein | up |
| Q0JM76 | GRXS4 | chloroplast protein | up |
| Q0IWL9 | GRXS11 | chloroplast protein | up |
| Q7XKV4 | BGLU12 | chloroplast protein | up |
| Q6ZAA5 | Os08g0447000 | chloroplast protein | up |
| B9EW03 | OsJ_04629 | chloroplast protein | up |
| A0A8J8XDX3 | Os01g0965400 | chloroplast protein | up |
| B9FLK4 | Os05g0558400 | chloroplast protein | up |
| A2ZY47 | Os01g0764400 | chloroplast protein | up |
| Q851Y7 | GRXS7 | chloroplast protein | up |
| Q9LD61 | PYRB | chloroplast protein | up |
| A0A0P0WDN1 | Os04g0573200 | chloroplast protein | up |
| B7FA99 | Os02g0302200 | chloroplast protein | up |
| A2ZPW5 | P0509B06.2-1 | chloroplast protein | up |
| Q2QUI0 | LOC_Os12g16200 | chloroplast protein | up |
| A3BT95 | OsJ_27364 | chloroplast protein | up |
| B9FAM9 | OsJ_12142 | chloroplast protein | up |
| Q6ZKV8 | BIO3-BIO1 | chloroplast protein | up |
| Q0JD85 | FBN5 | chloroplast protein | up |
| A3BMP5 | P0524G08.116 | chloroplast protein | up |
| A0A7S8BAT2 | rps11 | chloroplast protein | up |
| Q0DRP7 | Os03g0366000 | chloroplast protein | up |
| B9FWI5 | Os07g0264100 | chloroplast protein | up |
| B9G087 | OsJ_26844 | chloroplast protein | up |
| Q10G26 | LOC_Os03g44420 | chloroplast protein | up |
| B9FTN9 | OsJ_21625 | chloroplast protein | up |
| Q2R3H7 | LOC_Os11g31900 | chloroplast protein | up |
| Q8L6H7 | SFR2 | chloroplast protein | up |
| Q5ZD09 | Os01g0770500 | chloroplast protein | up |
| A3BKC8 | OsJ_24455 | chloroplast protein | down |
| A0A0P0WJN4 | Os05g0241200 | chloroplast protein | down |
| A0A7S7YAD2 | rpl2 | chloroplast protein | down |
| Q9ZST0 | RPL5 | chloroplast protein | down |
| Q60E59 | Os05g0388500 | chloroplast protein | down |
| Q10MB2 | LOC_Os03g20100 | chloroplast protein | down |
| Q10PV6 | LOC_Os03g12020 | chloroplast protein | down |
| A0A0N7KDR4 | Os01g0749200 | chloroplast protein | down |
| Q850W6 | LOC_Os03g34040 | chloroplast protein | down |
| Q7DNB2 | OSJNBa0061C08.3 | chloroplast protein | down |
| Q653V9 | Os06g0683200 | chloroplast protein | down |
| P12153 | rps19-B | chloroplast protein | down |
| A3A587 | Os02g0259600 | chloroplast protein | down |
| A0A5S6R9G6 | OJ1119_A01.23-1 | chloroplast protein | down |
| Q84QA8 | OJ1012B02.13 | chloroplast protein | down |
| Q6Z6A0 | Os02g0754300 | chloroplast protein | down |
| P0C443 | rpl16 | chloroplast protein | down |
| B9F5W8 | OsJ_12727 | chloroplast protein | down |
| Q0DG05 | PSAH | chloroplast protein | down |
| Q8SAY0 | RPL18 | chloroplast protein | down |
| Q8H656 | OSJNBa0019F11.29-2 | chloroplast protein | down |
| O65037 | RPL27 | chloroplast protein | down |
| Q8RVB1 | Os01g0633000 | chloroplast protein | down |
| P12151 | rps16 | chloroplast protein | down |
| Q5KQD5 | Os05g0101400 | chloroplast protein | down |
| B9F1E6 | OsJ_07762 | chloroplast protein | down |
| A0A7S7YC46 | ndhD | chloroplast protein | down |
| A0A0P0WCB4 | Os04g0496400 | chloroplast protein | down |
| A0A0N7KMI0 | Os06g0647100 | chloroplast protein | down |
| B7E324 | Os05g0186000 | chloroplast protein | down |
| B9FRD6 | OsJ_20044 | cytoplasm protein | up |
| Q0DM51 | Os03g0827700 | cytoplasm protein | up |
| Q5N725 | FBA3 | cytoplasm protein | up |
| P17784 | FBA1 | cytoplasm protein | up |
| Q2QXR8 | Os12g0145700 | cytoplasm protein | up |
| Q0JM17 | AIP1 | cytoplasm protein | up |
| Q7FAH2 | GAPC2 | cytoplasm protein | up |
| Q0J908 | Os04g0677500 | cytoplasm protein | up |
| Q337Y2 | CAD3 | cytoplasm protein | up |
| A3ANX5 | OsJ_13062 | cytoplasm protein | up |
| B9F2K0 | Os02g0732500 | cytoplasm protein | up |
| A3ABS9 | OJ1353_F08.16-1 | cytoplasm protein | up |
| Q0INR5 | LOC_Os12g21798 | cytoplasm protein | up |
| Q0DWH1 | Os02g0815500 | cytoplasm protein | up |
| Q652L6 | MDAR3 | cytoplasm protein | up |
| A3ANA0 | OsJ_12810 | cytoplasm protein | up |
| Q40665 | TUBB3 | cytoplasm protein | up |
| Q6K5G8 | GAPC3 | cytoplasm protein | up |
| A0A8J8YE53 | OsJ_11750 | cytoplasm protein | up |
| B9F6J9 | Os03g0804800 | cytoplasm protein | up |
| B9F279 | Os02g0125100 | cytoplasm protein | up |
| Q6Z4K6 | PL10B | cytoplasm protein | up |
| Q9XEA8 | RCS3 | cytoplasm protein | up |
| Q0J136 | Os09g0465600 | cytoplasm protein | up |
| A0A0P0WCM6 | Os04g0508200 | cytoplasm protein | up |
| Q6H4V1 | OSJNBa0085K21.52 | cytoplasm protein | up |
| Q10BT5 | PP2A2 | cytoplasm protein | up |
| A0A0N7KFQ1 | Os02g0623500 | cytoplasm protein | up |
| B9FHI2 | OsJ_18278 | cytoplasm protein | up |
| Q0DXH5 | Os02g0753800 | cytoplasm protein | up |
| A3A253 | OsJ_04994 | cytoplasm protein | up |
| Q7XTL6 | Os04g0687100 | cytoplasm protein | up |
| A0A0P0VTX8 | Os03g0182600 | cytoplasm protein | up |
| P48489 | Os03g0268000 | cytoplasm protein | up |
| Q5QLQ5 | Os01g0667200 | cytoplasm protein | up |
| Q67UF5 | PDIL2-3 | cytoplasm protein | up |
| D7PPK3 | adh1 | cytoplasm protein | up |
| A3AAG5 | OsJ_08055 | cytoplasm protein | up |
| Q8LR54 | Os01g0708100 | cytoplasm protein | up |
| B9FQR0 | OsJ_22533 | cytoplasm protein | up |
| Q851K1 | Os03g0694000 | cytoplasm protein | up |
| B9FUQ4 | OsJ_25474 | cytoplasm protein | up |
| Q5Z8Y9 | Os06g0564500 | cytoplasm protein | up |
| Q0JD42 | PDIL5-2 | cytoplasm protein | up |
| Q9FRT3 | RTRXH2 | cytoplasm protein | up |
| Q7XHP7 | Os07g0239400 | cytoplasm protein | up |
| Q8LR75 | Os01g0841600 | cytoplasm protein | up |
| Q84TB6 | ADF3 | cytoplasm protein | up |
| Q9AY76 | ADF2 | cytoplasm protein | up |
| P49964 | SRP19 | cytoplasm protein | up |
| Q6ER49 | prx29 | cytoplasm protein | down |
| A0A0P0W8S4 | Os04g0304200 | cytoplasm protein | down |
| Q8RYZ1 | P0648C09.9 | cytoplasm protein | down |
| B9F4N8 | OsJ_08984 | cytoplasm protein | down |
| Q10RN9 | LOC_Os03g05740 | cytoplasm protein | down |
| A3C154 | OsJ_30203 | cytoplasm protein | down |
| B9F0U0 | OsJ_07336 | cytoplasm protein | down |
| Q69QQ6 | HSP81-2 | endoplasmic reticulum protein | up |
| Q0J4P2 | HSP81-1 | endoplasmic reticulum protein | up |
| B9G449 | OsJ_29732 | endoplasmic reticulum protein | up |
| B9G1I1 | OsJ_27736 | endoplasmic reticulum protein | up |
| B9FJH8 | Os05g0460000 | endoplasmic reticulum protein | up |
| Q943K7 | Os01g0840100 | endoplasmic reticulum protein | up |
| A0A0P0YAS2 | Os12g0514500 | endoplasmic reticulum protein | up |
| Q65XA0 | DHAR1 | endoplasmic reticulum protein | up |
| Q65XA1 | Os05g0116000 | endoplasmic reticulum protein | up |
| Q84YK7 | BGLU27 | endoplasmic reticulum protein | up |
| A3A3M4 | OsJ_05566 | endoplasmic reticulum protein | up |
| Q5W6F1 | C4HL | endoplasmic reticulum protein | up |
| Q60DX8 | BGLU22 | endoplasmic reticulum protein | up |
| Q7Y0D5 | OSJNBa0079B15.14 | endoplasmic reticulum protein | up |
| A0A0P0UZ45 | Os01g0182200 | endoplasmic reticulum protein | up |
| A0A0N7KH18 | Os03g0281600 | endoplasmic reticulum protein | down |
| Q6ZGW6 | FAD2 | endoplasmic reticulum protein | down |
| B9FL15 | OsJ_19137 | endoplasmic reticulum protein | down |
| A3B934 | Os06g0186400 | extracell protein | up |
| A0A0P0WPV1 | Os05g0517500 | extracell protein | up |
| Q2QNG2 | LOC_Os12g37910 | extracell protein | up |
| A3C4S4 | GME-1 | Golgi apparatus protein | up |
| A0A0P0VWN1 | Os03g0278200 | Golgi apparatus protein | up |
| Q7XVN7 | ERDJ2 | Golgi apparatus protein | up |
| Q7XBX2 | LOC_Os10g42420 | Golgi apparatus protein | up |
| Q0DVC7 | Os03g0136800 | Golgi apparatus protein | down |
| Q5KQL0 | LOC_Os03g63710 | Golgi apparatus protein | down |
| B9F342 | OsJ_08475 | mitochondrion protein | up |
| Q8H903 | Os10g0462900 | mitochondrion protein | up |
| B9FB06 | Os03g0143400 | mitochondrion protein | up |
| A0A0P0X3K0 | Os07g0188800 | mitochondrion protein | up |
| Q8S5T1 | LOC_Os03g06740 | mitochondrion protein | up |
| Q6ER90 | Os02g0538000 | mitochondrion protein | up |
| Q0J432 | Os08g0538000 | mitochondrion protein | up |
| H2KW24 | LOC_Os11g01154 | mitochondrion protein | up |
| Q8HCR2 | rps7 | mitochondrion protein | up |
| Q7JAI6 | cox1 | mitochondrion protein | up |
| Q84ZB9 | Os07g0549800 | mitochondrion protein | up |
| A0A0P0VQ36 | Os02g0774300 | mitochondrion protein | up |
| Q6L5I5 | VDAC2 | mitochondrion protein | up |
| B9G059 | OsJ_26793 | mitochondrion protein | up |
| Q7XTJ3 | Os04g0119400 | mitochondrion protein | up |
| Q0DFB6 | Os06g0103600 | mitochondrion protein | up |
| A3BNM4 | OsJ_25659 | mitochondrion protein | up |
| A0A5S6R775 | Os03g0308100 | mitochondrion protein | up |
| B9FXC6 | OJ1457_D07.117 | mitochondrion protein | up |
| Q94CN9 | Os01g0337900 | mitochondrion protein | up |
| Q7XN11 | OSL2 | mitochondrion protein | up |
| Q10LR5 | LOC_Os03g21950 | mitochondrion protein | up |
| Q6Z3X5 | Os07g0695800 | mitochondrion protein | up |
| Q75IM9 | Os05g0125500 | mitochondrion protein | up |
| A0A0P0UYL6 | Os01g0143800 | mitochondrion protein | up |
| Q9SDD6 | PRXIIF | mitochondrion protein | up |
| Q7XZF7 | GYRA | mitochondrion protein | up |
| B9G4B3 | Os09g0491772 | mitochondrion protein | up |
| Q5JJI4 | TOM20 | mitochondrion protein | up |
| Q69J84 | P0037D09.22-1 | mitochondrion protein | up |
| B9GC70 | OsJ_35450 | mitochondrion protein | up |
| O22567 | CLA1 | mitochondrion protein | up |
| Q33E23 | GDH2 | mitochondrion protein | up |
| Q0DGC8 | Os05g0540300 | mitochondrion protein | up |
| A0A0P0XPK4 | Os09g0509000 | mitochondrion protein | up |
| Q0DA15 | Os06g0684000 | mitochondrion protein | up |
| A0A8J8YAS4 | Os03g0323800 | mitochondrion protein | up |
| Q5ZCF2 | Os01g0778800 | mitochondrion protein | up |
| Q852G0 | LOC_Os03g29810 | mitochondrion protein | up |
| Q6K456 | Os09g0132600 | mitochondrion protein | up |
| B9EV70 | Os01g0276200 | mitochondrion protein | up |
| Q7XVK0 | OSJNBa0069D17.1 | mitochondrion protein | up |
| Q0IYG0 | Os10g0321700 | mitochondrion protein | up |
| Q5N9P6 | Os01g0695300 | mitochondrion protein | up |
| A0A0P0X6F8 | Os07g0485400 | mitochondrion protein | up |
| A0A0N7KFF9 | Os02g0541700 | mitochondrion protein | up |
| Q0JJ35 | Os01g0762600 | mitochondrion protein | up |
| B9G1U9 | OsJ_27983 | mitochondrion protein | up |
| Q8HCQ0 | nad3 | mitochondrion protein | up |
| Q5JJV3 | Os01g0966300 | mitochondrion protein | up |
| P0CD23 | ndhB2 | mitochondrion protein | down |
| Q6EUK5 | Os02g0234500 | mitochondrion protein | down |
| A3AI13 | OsJ_10878 | mitochondrion protein | down |
| Q850L8 | OSJNBa0032E21.12 | mitochondrion protein | down |
| Q69S39 | petC | mitochondrion protein | down |
| Q0J1V9 | Os09g0408600 | mitochondrion protein | down |
| A0A8J8XZB2 | OsJ_32559 | mitochondrion protein | down |
| Q0J032 | Os09g0533400 | mitochondrion protein | down |
| Q7X7T4 | OSJNBa0076N16.4 | mitochondrion protein | down |
| Q9FE64 | Os03g0565500 | mitochondrion protein | down |
| Q6L4S2 | Os05g0592000 | nucleus protein | up |
| Q2QQM3 | LOC_Os12g30540 | nucleus protein | up |
| Q9AV81 | PRP19 | nucleus protein | up |
| Q5NAI9 | Os01g0710000 | nucleus protein | up |
| Q8W426 | Os21D7 | nucleus protein | up |
| Q7X5X9 | Os04g0444600 | nucleus protein | up |
| Q67J09 | Os09g0460400 | nucleus protein | up |
| Q0JGY1 | RPL5A | nucleus protein | up |
| B9F8W6 | OsJ_11170 | nucleus protein | up |
| Q53JF7 | ASR5 | nucleus protein | up |
| Q6ZDG3 | Os08g0475400 | nucleus protein | up |
| A0A0N7KJ23 | Os04g0423400 | nucleus protein | up |
| B9FY56 | OsJ_24922 | nucleus protein | up |
| A3BAG6 | Os06g0264300 | nucleus protein | up |
| B9FRK1 | OsJ_20171 | nucleus protein | up |
| Q10G81 | MSI1 | nucleus protein | up |
| Q0DIQ4 | Os05g0371200 | nucleus protein | up |
| Q8H936 | CSN5 | nucleus protein | up |
| A0A0P0WV17 | Os06g0237502 | nucleus protein | up |
| A0A8J8XXT2 | Os03g0116500 | nucleus protein | up |
| Q0DFD6 | Os05g0597100 | nucleus protein | up |
| A3BFZ0 | OsJ_22867 | nucleus protein | up |
| Q0E2F5 | Os02g0245100 | nucleus protein | up |
| P31673 | HSP17.4 | nucleus protein | up |
| A3BCV5 | OsJ_21732 | nucleus protein | up |
| Q6ESK5 | Os09g0411700 | nucleus protein | up |
| Q652V8 | HSP16.0 | nucleus protein | up |
| A0A5S6RBF7 | OSJNBb0015I11.23 | nucleus protein | up |
| A0A5S6R7M2 | Os04g0636900 | nucleus protein | up |
| A0A0P0V127 | Os01g0271500 | nucleus protein | up |
| A0A0P0XUQ1 | Os10g0447100 | nucleus protein | up |
| B9FC09 | OsJ_15807 | nucleus protein | up |
| B9GCB6 | OsJ_35543 | nucleus protein | up |
| Q0J0D1 | Os09g0516200 | nucleus protein | down |
| Q6YXE9 | B1052H09.108 | nucleus protein | down |
| Q10FT7 | LOC_Os03g45340 | nucleus protein | down |
| A3ANB5 | ARP7 | nucleus protein | down |
| Q5Z754 | CDKF-1 | nucleus protein | down |
| Q2QPG9 | Os12g0530000 | nucleus protein | down |
| A0A0P0X3U2 | OJ1046_F10.127 | nucleus protein | down |
| A0A0P0WC35 | Os04g0504600 | peroxisome protein | up |
| Q75LB3 | LOC_Os03g62850 | peroxisome protein | up |
| Q6ZA87 | OsJ_27438 | peroxisome protein | up |
| A3BYC3 | OsJ_29182 | peroxisome protein | up |
| Q8H3I4 | GLO4 | peroxisome protein | up |
| Q0JEQ2 | APX3 | peroxisome protein | up |
| A0A0P0VGU6 | Os02g0236800 | peroxisome protein | up |
| Q5U1N4 | prx59 | peroxisome protein | down |
| A2ZU58 | OsJ_02142 | peroxisome protein | down |
| Q0IM13 | Os12g0607100 | plastid protein | up |
| B9GC51 | OsJ_35408 | plastid protein | up |
| A3AT06 | OsJ_14493 | plastid protein | up |
| A0A8J8XMH3 | Os12g0609500 | plastid protein | up |
| Q6Z510 | OSJNBa0036E18.21 | plastid protein | down |
| A0A0N9DZK6 | rps2 | plastid protein | down |
| A0A7S8BCX7 | rps4 | plastid protein | down |
| Q0IQR0 | Os12g0106000 | plastid protein | down |
| A0A0N9E0Z0 | rpl22 | plastid protein | down |
| P12139 | rpl20 | plastid protein | down |
| A0A0N9E0L8 | rps7 | plastid protein | down |
| A0A0P0WB26 | Os04g0461100 | plastid protein | down |
| P0C457 | rpl33 | plastid protein | down |
| Q7F1F2 | Os08g0525600 | vacuole protein | up |
| A0A0P0W1L8 | Os03g0687000 | vacuole protein | up |
| Q5Z8U4 | P0018H04.22 | vacuole protein | up |
| Q5W6Z6 | Os05g0247100 | vacuole protein | up |
| B9F1V9 | OsJ_07974 | vacuole protein | up |
| Q0D6H5 | Os07g0479300 | vacuole protein | up |
| P50156 | TIP1-1 | vacuole protein | up |
| Q0JBF1 | Os04g0535600 | vacuole protein | up |
| A0A0N7KGD3 | P0452F04.18-1 | vacuole protein | up |
| B9FB84 | OsJ_12376 | vacuole protein | up |
| Q0IW04 | OSJNBb0015I11.16 | vacuole protein | down |

## Table S7. Peptide fraction separation by liquid chromatography elution gradients

| Time (min) | flow rate (mL/min) | mobile phaseA (%) | mobile phase B (%) |
| --- | --- | --- | --- |
| 0 | 1 | 97 | 3 |
| 10 | 1 | 95 | 5 |
| 30 | 1 | 80 | 20 |
| 48 | 1 | 60 | 40 |
| 50 | 1 | 50 | 50 |
| 53 | 1 | 30 | 70 |
| 54 | 1 | 0 | 100 |

# Section 2. The Carotenoids of Leaves.

Fresh leaf samples (0.1g) were finely chopped and thoroughly ground in 80% acetone. The homogenate was centrifuged, and the supernatant was collected and brought to a final volume of 10 mL. The extract was then analyzed using a UV-Vis spectrophotometer (UV-6100 spectrophotometer, Shanghai Mapada Instruments Co., Ltd., China), measuring absorbance at three specific wavelengths: 665 nm, 649 nm, and 470 nm.

The carotenoid content was calculated using the equation:

Carotenoid content (mg/g) = CC × V × N / (W × 1000), where CC = (1000 × A470 - 2851.304 × A649 + 811.7385 × A665) / 245 (mg/L), V is the extract volume (10 mL), N is the dilution factor, and W is the fresh leaf weight in g.


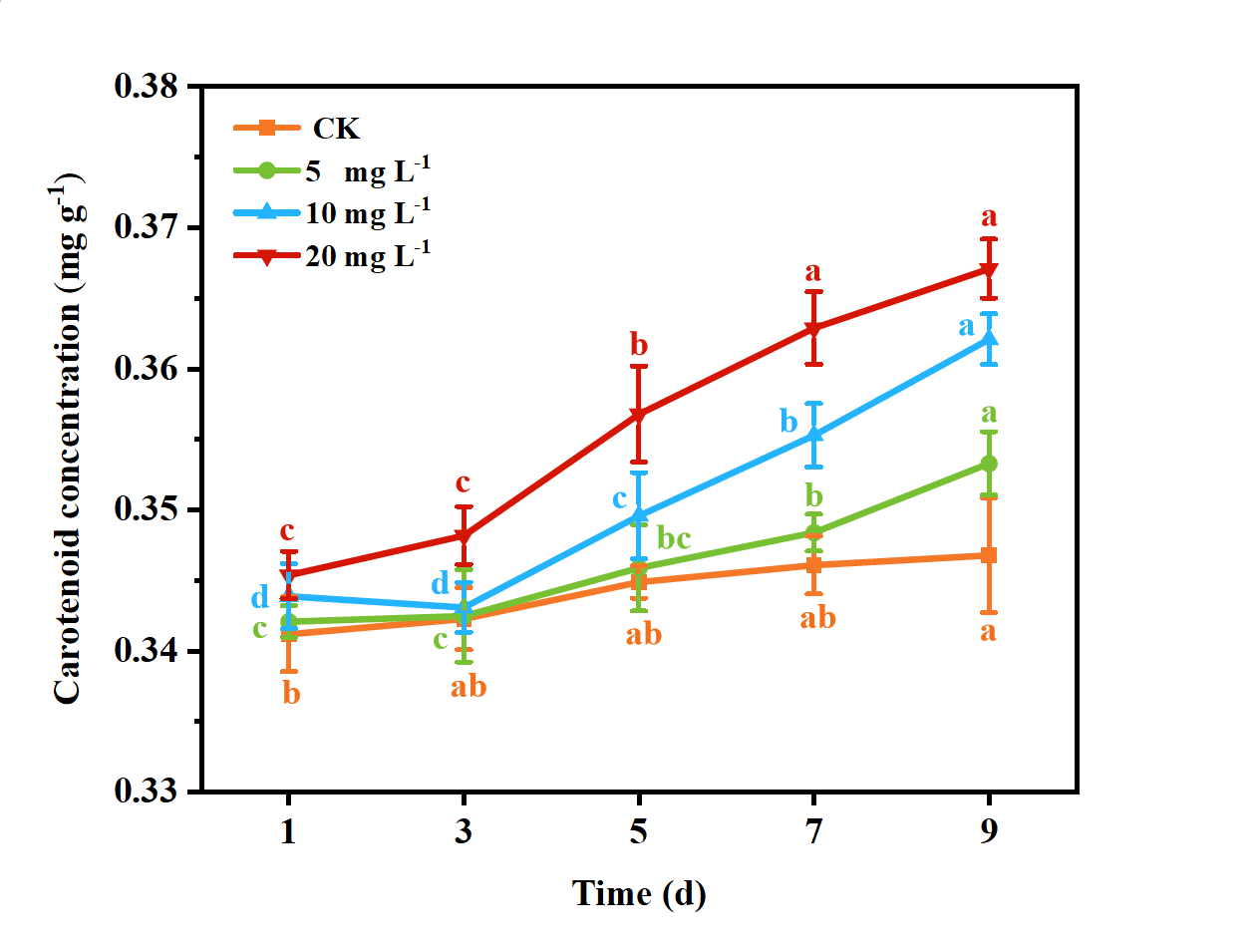


**Figure S1. Concentrations of Carotenoids in rice plants following CIP treatment, measured on day of 1st, 3rd, 5th, 7th, and 9^th^.**

# Section 3. Influence of Ciprofloxacin on Media pH

The internal environment of leaf cells was simulated using both organic and inorganic buffer conditions. The organic buffer consisted of a 0.35 mol L^-1^ glucose solution, based on the protoplast preparation solution described by Schlangstedt et al. (1992). For the inorganic buffer, a protoplast suspension buffer containing KCl, MgCl_2_·6H_2_O, and CaCl_2_·2H_2_O was used, as reported by Toriyama and Hinata (1985). Additionally, we included a control test of the CIP in deionized (DI) water.


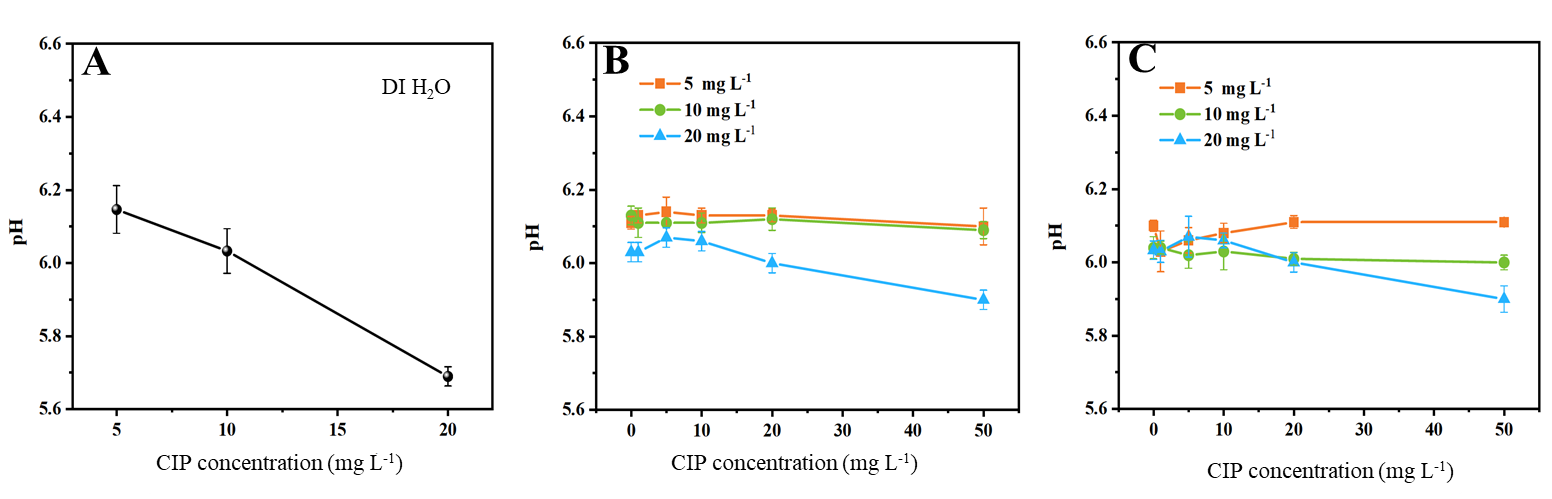


**Figure S2. Effect of CIP concentration on pH in different solutions. (A) pH changes in deionized water (DI H_2_O); (B) pH changes in glucose solution; and (C) pH changes in an inorganic solution containing at different CIP concentrations (5, 10, and 20 mg L⁻¹). Error bars represent standard deviation.**

**Reference**

Schlangstedt M, Hermans B, Zoglauer K, et al. Culture of sugar beet (Beta vulgaris L.) protoplasts in alginate—callus formation and root organogenesis. Journal of plant physiology, 1992, 140(3): 339-344.

Toriyama K, Hinata K. Cell suspension and protoplast culture in rice. Plant Science, 1985, 41(3): 179-183.

# Section 4. The Biodegradation of Ciprofloxacin in Plant Cells

Rice seedlings (Oryza sativa L. cv. Nanjing 9108) were obtained from Jiangsu Academy of Agricultural Sciences Seed Station. Seeds were surface-sterilized with 3% H₂O₂ for 5 minutes, thoroughly rinsed with ultrapure water, and imbibed overnight. After 7-day germination on deionized water-soaked gauze, seedlings were transferred to black plastic containers with Hoagland's nutrient solution for 14 days under controlled conditions (16/8 h light/dark photoperiod, 400 μmol m⁻² s⁻¹ light intensity, 25/20 °C Day/night temperature, 60% relative humidity). The nutrient solution was renewed every 4 days. Uniform seedlings were selected and exposed to 20 mg L⁻¹ CIP in Hoagland's solution (pH 5.5) for 9 days, with solution replenishment every two days. Control groups were maintained in CIP-free Hoagland's solution. All treatments were conducted in triplicate. Sample Preparation Twelve plants from the 20 mg L⁻¹ CIP treatment group were pooled and processed. Fresh leaves (approximately 0.5 g) were collected and immediately pulverized using a grinding mill. The homogenized sample (40 mg) was transferred to a 2 mL centrifuge tube and mixed with 400 μL of 75% (v/v) methanol aqueous solution. The mixture underwent ultrasonication for 30 minutes, followed by centrifugation (17,000 g, 20°C, 10 minutes). The supernatant was filtered through a 0.22 μm membrane and transferred to LC vials for analysis.

Chromatographic separation was performed using a Triple TOF™ 5600 system (AB SCIEX) equipped with an Acquity Uplc Hss T3 column (2.1 × 100 mm, 1.8 μm). The mobile phase consisted of 0.1% formic acid (v/v) (A) and acetonitrile (B). The gradient elution program was: 0-2 min: 90% A; 2-10 min: Linear decrease to 40% A; 10-20 min: Linear decrease to 10% A; 20-22 min: Hold at 10% A; 22-25 min: Return to 90% A; 25-27 min: Hold at 90% A. Operating conditions included flow rate at 0.4 mL min⁻¹, column temperature at 40°C, injection volume of 4 μL, DuoSpray™ ion source, source temperature at 550°C, ion spray voltage at +5500V (positive mode) and -4500V (negative mode), ion source gas at 60 psi, curtain gas at 35 psi, and mass range of m/z 50-500. And the related and potential chemicals were listed in **Figure S3**.

Data acquisition and processing were performed using MSDIAL ver 4.6 Software and PeakView® Software 2.2 (AB SCIEX). Metabolites were identified based on accurate mass measurements, isotopic patterns, and MS/MS fragmentation patterns.


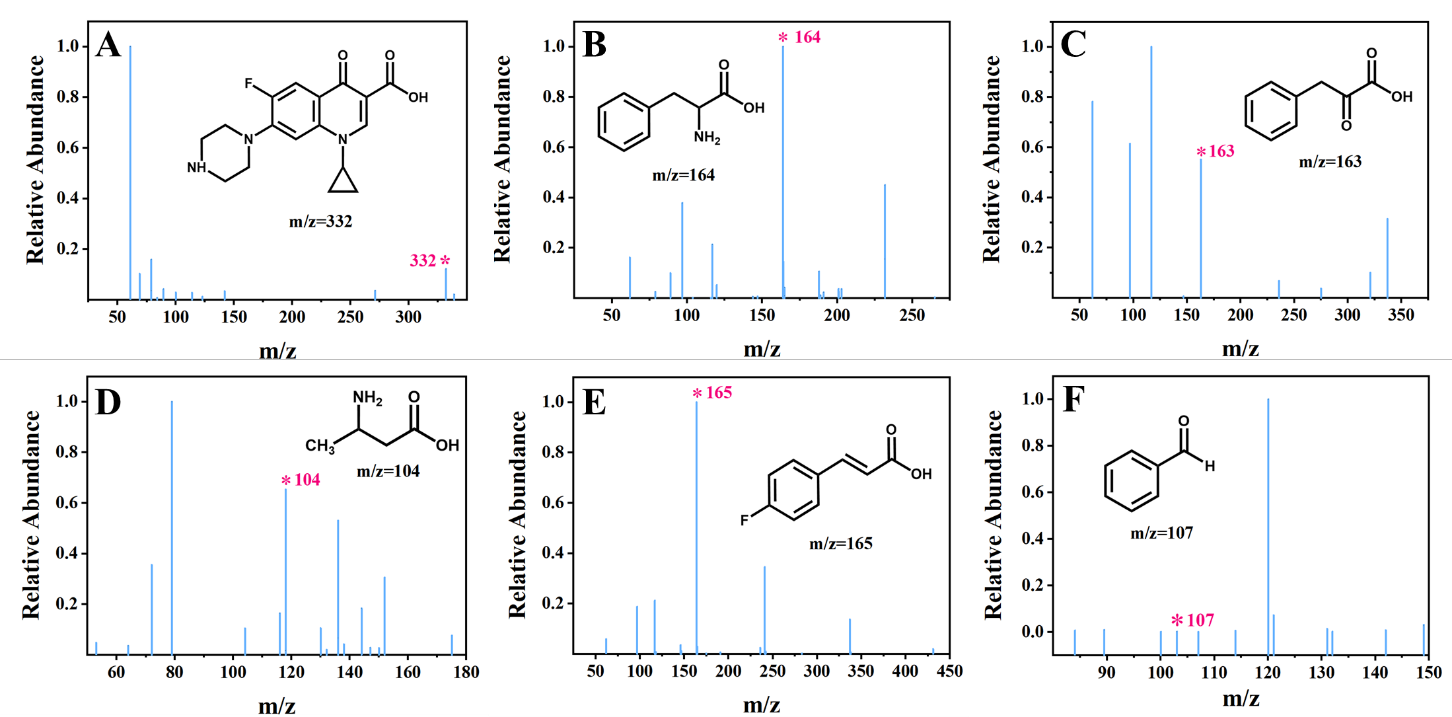


**Figure S3. Mass spectra and chemical structures of CIP and its transformation products in rice leaves.**

(Note, (A) Parent compound ciprofloxacin (m/z 332); (B) Phenylalanine (m/z 164); (C) Phenylpyruvic acid (m/z 163); (D) 3-Aminoisobutyric acid (m/z 104); (E) 4-Fluorocinnamic acid (m/z 165); and (F) Benzaldehyde (m/z 107). The relative abundance is shown on the y-axis, and mass-to-charge ratio (m/z) is shown on the x-axis. Pink asterisks (*) indicate the molecular ion peaks of identified compounds. The mass spectra were obtained by subtracting the corresponding control spectra to eliminate background signals.)

The transformation products of CIP in rice plant cells were identified and characterized (Table S1). Mass spectrometry analysis revealed five major metabolites: phenylalanine (m/z 164), phenylpyruvic acid (m/z 163), 3-aminoisobutyric acid (m/z 104), 4-fluorocinnamic acid (m/z 165), and benzaldehyde (m/z 107). The relative abundances and proposed structures of these metabolites are provided in Figure S1. The transformation pathway analysis suggested two primary degradation routes: ROS-mediated oxidation and deamination, as illustrated in **Figure S4**.


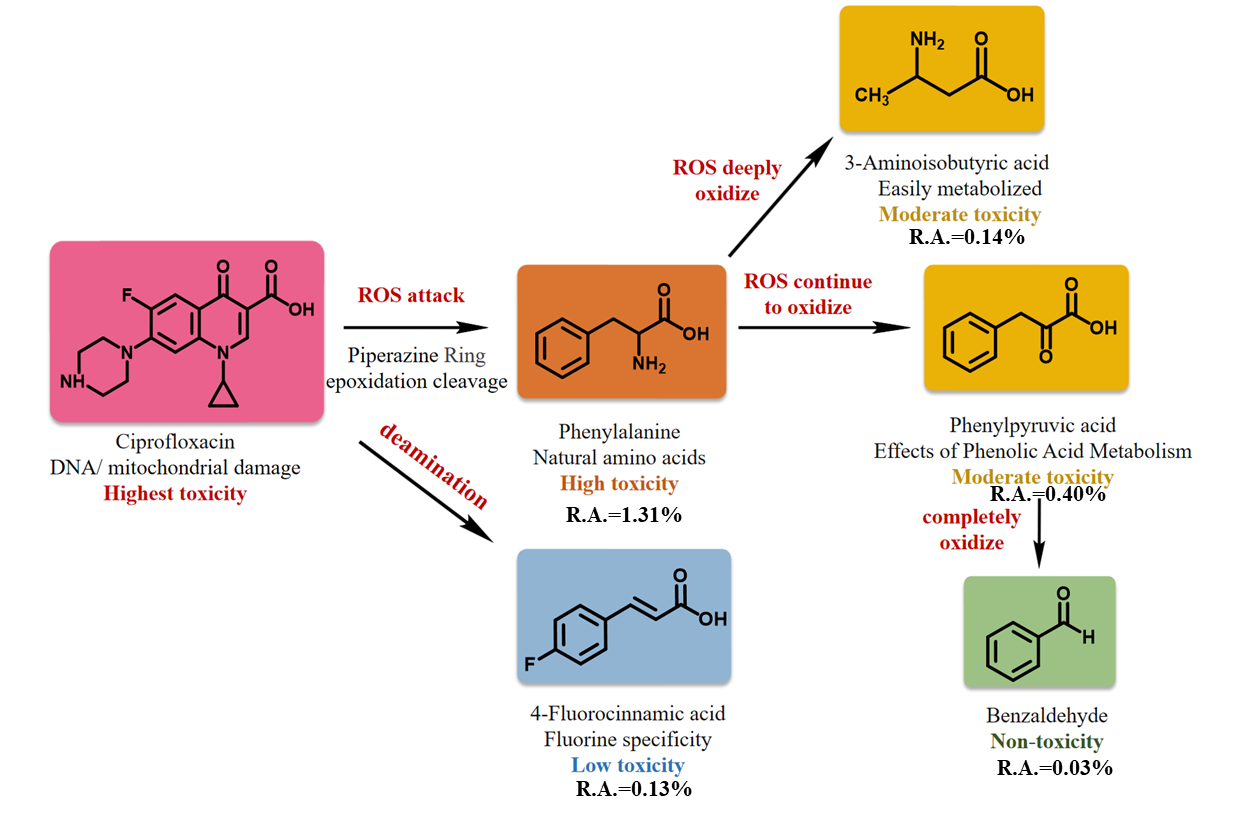


**Figure S4. Proposed transformation pathways of ciprofloxacin (CIP) in rice plants. CIP undergoes two major degradation routes: ROS-mediated oxidation and deamination.**

(Note, The ROS-mediated pathway begins with piperazine ring epoxidation cleavage, forming phenylalanine (relative abundance, RA=1.31%) which is further oxidized to phenylpyruvic acid (RA=0.40%) and other intermediates. The deamination pathway produces 4-fluorocinnamic acid (RA=0.13%). Both pathways lead to decreased toxicity of metabolites compared to the parent compound. Color coding indicates toxicity levels: pink (highest), orange (high), yellow (moderate), blue (low), and green (non-toxic).
